# Widely-used boronic esters as synthetically-versatile directing groups for C–H activation and hydrogen isotope exchange†

**DOI:** 10.1039/d5sc09113f

**Published:** 2026-01-14

**Authors:** J. Conor Townsley, Connor Smith, David M. Lindsay, Gemma M. Liwicki, Nicholas D. Measom, Laura C. Paterson, William J. Kerr

**Affiliations:** a Department of Pure and Applied Chemistry, University of Strathclyde 295 Cathedral Street Glasgow Scotland G1 1XL UK w.kerr@strath.ac.uk; b GSK Medicines Research Centre Gunnels Wood Road, Stevenage Hertfordshire England SG1 2NY UK

## Abstract

Herein we report the first use of accessible boron-containing compounds as highly effective directing groups for C–H activation and hydrogen isotope exchange. Selective *ortho*-activation and functionalisation at aromatic C-sp^2^ centres have been achieved across an array of aryl boronic ester species using an iridium-based, NHC/phosphine catalyst system at low loadings. The process is robust, with a wide scope of over 30 substrates, and delivers excellent levels of deuterium incorporation in a selective manner. Further utilisation of the resulting boron-containing isotopologues in cross-coupling chemistry has allowed the late-stage preparation of previously less accessible site-selectively labelled structures. This strategy has been exemplified *via* the preparation of an isotopically-labelled, biologically-active drug molecule.

## Abstract 2

We report the first use of accessible boronic esters as effective directing groups for C–H activation and hydrogen isotope exchange. A chelated NHC/phosphine Ir catalyst system delivers selective *ortho*-C-sp^2^ activation with a range of substrates.

## Graphical abstract

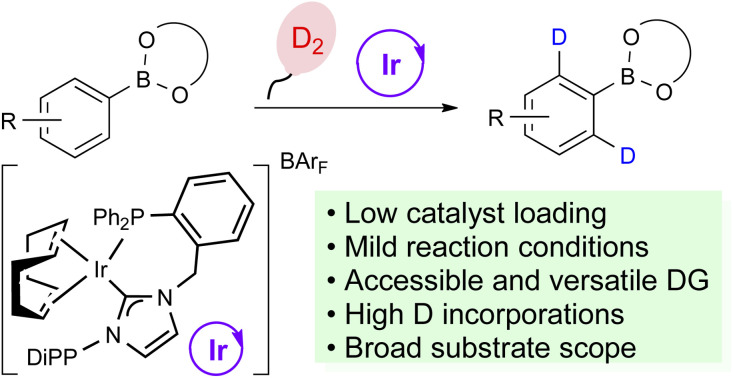

## Introduction

**Scheme 1:**
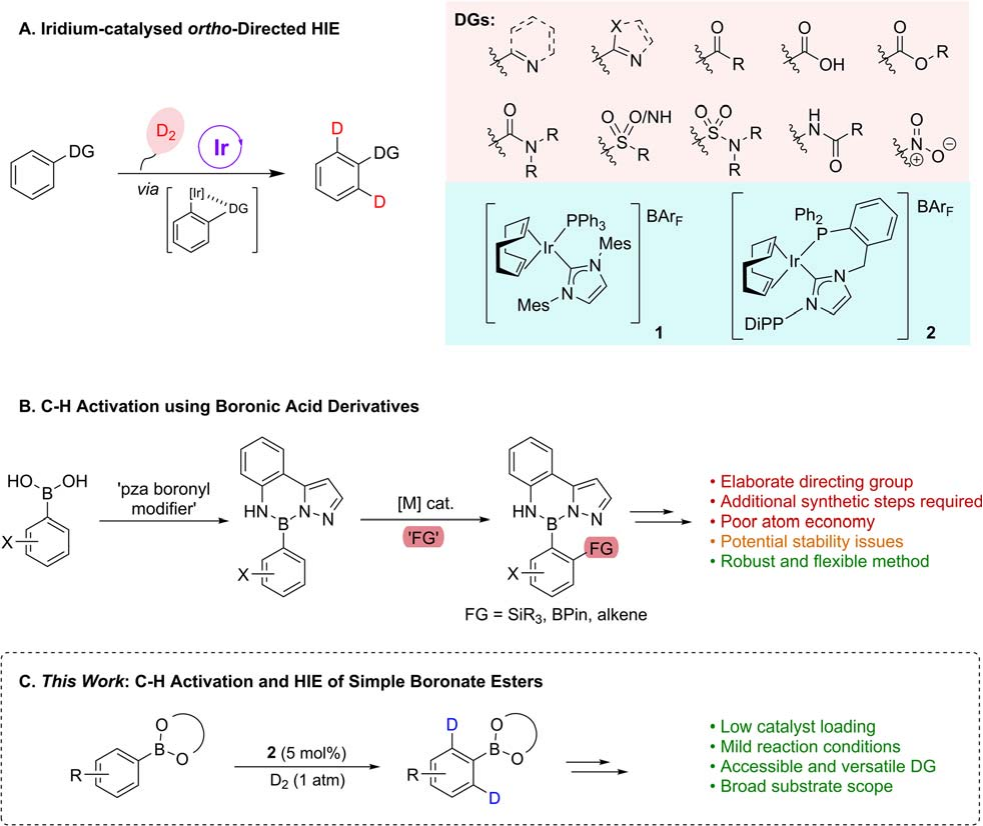
(A and B) Previous work; (C) this work.

Compounds incorporating heavy isotopes of hydrogen have proven invaluable both in advancing our understanding of chemical processes and in establishing novel therapeutic approaches.^1^ More specifically, deuterium-containing molecules have had widespread applications across various scientific domains, including in the elucidation of organic reaction mechanisms through the study of kinetic isotope effects;^2^ in adsorption, distribution, metabolism, excretion, and toxicity (ADMET) studies in pharmaceutical research;^1,3^ and, most recently, in developing deuterated marketed drugs.^4^ Of the many utilisable methods for the installation of deuterium labels, transition metal-catalysed hydrogen isotope exchange (HIE) has emerged as a leading technology that can rapidly deliver desired isotopologues from late-stage intermediates, or final target molecules themselves, and with site-selectivity.^5^ Within the field of HIE, research from our own laboratories has led to the development of a suite of iridium-based, homogeneous catalytic systems able to facilitate access to deuterated molecules *via* a selective, *ortho*-directed C–H activation and functionalisation pathway (Scheme 1A).^6^ In this regard, bulky mono- and bidentate phosphine/N-heterocyclic carbene (NHC) ligated iridium species, such as 1 and 2, are able to exploit an array of Lewis basic directing groups (DGs), including heterocycles, ketones, carboxylic acids, esters, amides, sulfur-based units, and the nitro moiety, to deliver selective C–H activation and hydrogen isotope exchange under mild conditions using an ambient pressure of deuterium gas as the isotopic source. These systems deliver high levels of isotopic labelling and have been successfully applied to multifunctional drug compounds, as well as being readily transferable to tritiation chemistry.^6*b*,*h*,*l*,*m*^

Despite this broad applicability, the Lewis base-directed C–H activation and HIE approach finds a limitation with weakly co-ordinating directing groups. A prime example of this is found with boron-containing compounds (BCCs), and, more specifically, with synthetically-versatile aryl boronate esters. These species are poor directing groups as a result of attenuation of the co-ordinating ability of the oxygen lone pairs due to the Lewis acidic boron. Related to this, the preparatively versatile nature of such BCCs means that they find application in a wide range of reactions,^7^ with notable examples including the Suzuki–Miyaura^8^ and Chan–Lam^9^ cross-coupling reactions, where boron-based reaction partners are essential components. Furthermore, novel BCCs are also emerging as robust and valuable tools in medicine and drug development, offering advantages such as enhanced drug binding, stability improvements, specific enzyme-inhibitor abilities, and neutron capture capabilities.^7*c*,10^ Such properties have led to the FDA-approval of five boron-containing drugs, with several further in various stages of clinical development. To the best of our knowledge, and despite this BCC prominence, there have been no examples to date of directed HIE using boron units. Indeed, more broadly, it is apparent that only very few C–H activation and subsequent functionalisation processes have been disclosed using BCCs as the directing functionality. Examples of such boron unit directed processes have been developed by Suginome and co-workers, whereby boronyl ‘modifiers’, such as anthranilamide (aam) or pyrazolylaniline (pza) groups, are employed to both protect the boron functionality, through suppression of transmetalation, as well as direct metal-mediated C–H silylation, borylation, or alkenylation, with these substrates being accessed *via* the parent boronic acids (Scheme 1B).^11^ Despite being effective in their desired function, both the aam and pza units represent relatively elaborated structures, with some issues relating to hydrolytic instability having also been noted.^11*a*,*b*^ As well as this, further manipulative steps are required to provide a boron-based unit which is applicable in desired subsequent cross-coupling processes. In this same overall area, the related work of Spivey and Cordier should be noted, whereby a nitrile unit tethered to an aryl *N*-methyliminodiacetic acid (MIDA) boronate effectively directs metal-catalysed *meta*-C(sp^2^)–H functionalisation.^12,13^

Based on this backdrop, it is apparent that there remains an unmet challenge with regards the use of simple boronate esters to directly facilitate C–H activation, whilst retaining the potential to readily further diversify what would be highly valuable functionalised boron-containing arenes. Accordingly, we envisioned that the iridium-based catalyst methods established within our laboratory could be further expanded to deliver unprecedented directed C–H activation/HIE methods for application with common boron-containing units (Scheme 1C). Moreover, we also hypothesised that a novel traceless labelling sequence could be established *via* further manipulation of the boron functional handle within the HIE products, to deliver subsequently produced molecular architectures with previously less accessible isotope patterns. Herein, we report the first examples of iridium-catalysed HIE using simple boronic esters as directing units, which also represents the first examples of these simple, readily accessible, and dynamic BCCs being utilised as a directing group within a C–H functionalisation process.

## Results and discussion

**Scheme 2:**
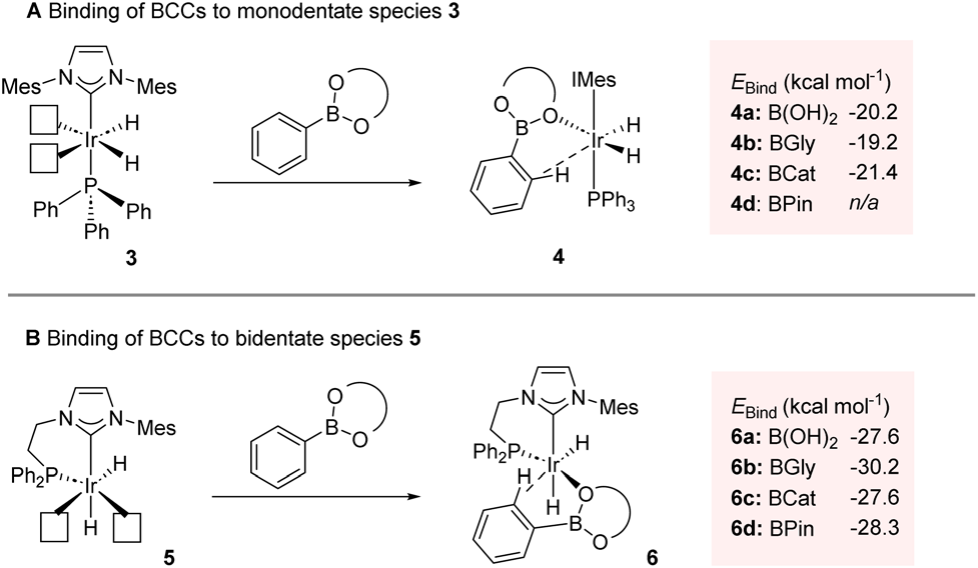
Binding energy (*E*_Bind_) considerations.

Consistent with our recent studies on catalyst development and application in HIE, our approach was fundamentally guided by computational methods. As related to this, we have previously employed substrate binding energy as a key principle in directing effective catalyst system design.^6*b*,*d*,*e*,*j*,*m*,14^ On this basis, the binding affinity of varying BCCs with catalytically-relevant^6*b*^ mono- and bidentate iridium(iii) dihydride species 3 and 5, respectively, was assessed *in silico* (Scheme 2).^15^ With regards the monodentate Ph_3_P/IMes species 3, significantly negative binding energy values (*E*_Bind_) were obtained for phenylboronic acid complex 4a, phenylboronic acid ethylene glycol ester (BGly) complex 4b, and phenylboronic acid catechol ester (BCat) complex 4c (Scheme 2A). Unfortunately, the binding energy of the key phenylboronic acid pinacol ester (BPin) agostic complex 4d, with the requisite interaction between the metal centre and the *ortho* C–H bond of the phenyl unit, could not be established, likely due to the steric combination of the four methyl groups within the pinacol unit and the bulky ligands on iridium. Comparing the calculated binding energies of species 3 with acetophenone (−23.1 kcal mol^−1^),^6*m*^ a substrate known for its exceptional performance under hydrogen isotope exchange conditions with such Ir catalyst species, it was concluded that the selected BCCs showed promise as viable substrates, albeit with potentially lower efficacy than acetophenone. When screening the same BCCs with the ethyl-linked bidentate complex 5, again, favourable *E*_Bind_ values were calculated (Scheme 2B). The free boronic acid- and BCat-bound complexes, 6a and 6c, respectively, both displayed a promising binding energy of −27.6 kcal mol^−1^, with the BGly-bound complex 6b displaying an even more favourable *E*_Bind_ value of −30.2 kcal mol^−1^. The enhanced binding energies observed in comparison to the monodentate complexes of type 4 can be attributed to the additional available space for substrate co-ordination around the iridium centre within the bidentate (chelated) species. This increased spatial accommodation also now facilitated the formation of the BPin-bound complex 6d, which exhibited a highly favourable binding energy of −28.3 kcal mol^−1^. Again, to gauge the potential HIE efficacy, positive comparisons were made with the *E*_Bind_ value of methylphenyl sulfone, a substrate which complexes to species 5 with a binding energy of −27.0 kcal mol^−1^,^6*m*^ despite the sterically challenging nature of the sulfone unit, and with this posited competence in HIE having also been verified experimentally with this sulfone species. Based on all of this, and with particular cognisance of the favorable binding energies of the studied BCCs within complex 6 compared with previously calculated species, it was envisaged that a bidentate catalyst system could effectively facilitate the labelling of synthetically tractable boron-containing compounds.

With the established computational evidence supporting our hypothesis, practical investigations were then initiated. The preliminary approach involved screening a selection of iridium-based catalysts developed in our laboratories against a small range of boron-containing starting substrates (Table 1). In this regard, at 5 mol% catalyst loading in methyl *tert*-butyl ether (MTBE) at 50 °C, the monodentate precatalyst 1 (precursor to species 3) performed poorly in the targeted HIE process, resulting in no observable deuterium incorporation with phenylboronic acid 10, phenyl-BGly 11, or phenyl-BPin, 12 (entry 1). Moving to the chelated precatalyst 7 (precursor to species 5), under the same conditions, low but encouraging levels of deuterium incorporation of 26% D and 8% D were observed with 11 and 12, respectively (entry 2). Manipulation of the steric environment within the catalyst sphere then allowed for improvements in the overall levels of labelling. Specifically, increasing the steric bulk on the NHC component by incorporating the di-iso-propylphenyl (DiPP) unit within 8 significantly enhanced the deuterium incorporation to 52% in BGly 11 and 18% in BPin 12 (entry 3). Benzyl-linked precatalysts 9 and 2 were also assessed (entries 4 and 5), with similar positive outcomes being observed, whereby deuterium incorporation was, again, most optimal with the larger DiPP-containing catalyst 2. Indeed, 62% D (11) and 26% D (12) represented the highest levels observed to this stage for these respective boron-containing species (entry 5).

**Table 1:** Catalyst screen with varying boron substrates

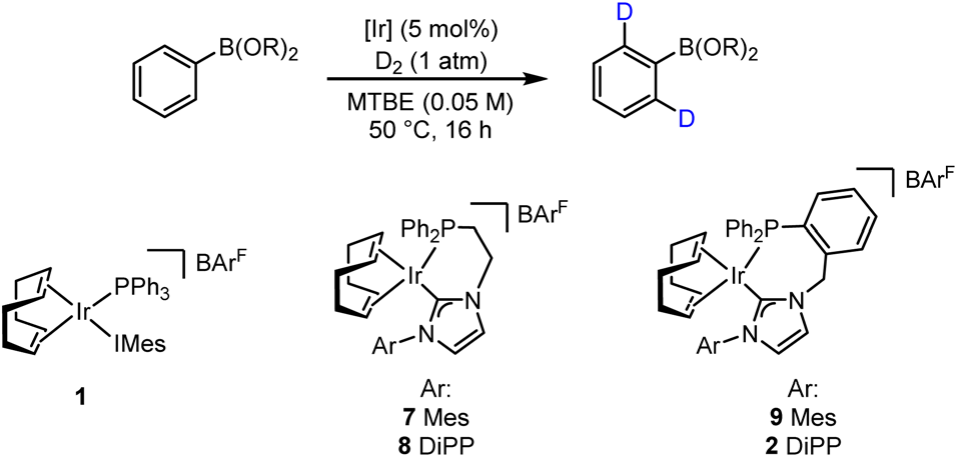				
Entry	Catalyst	Deuterium incorporation (%)		
		PhB(OH)_2_10^a^	PhBGly 11^a^	PhBPin 12^b^
1	1	<5	<5	<5
2	7	n.d.	26	8
3	8	—	52	18
4	9	—	22	5
5	2	—	62	26

a Deuterium incorporation is based on analysis of the ^1^H NMR spectrum of the crude reaction mixture and is an average of 2 runs.

b Deuterium incorporation is based on analysis of the ^1^H NMR spectrum of pure product and an average of 2 runs. n.d. denotes that no material was recovered.

Despite phenyl-BGly 11 displaying the highest levels of deuterium incorporation among the selected BCCs, due to the wider applicability, greater versatility, and significant utility of pinacol boronic esters for use in organic synthesis,^7*b*^ optimisation was continued with phenyl-BPin 12. Additionally, in a practical sense, phenyl-BPin 12 proved the most stable and easily handled of the starting substrates assessed in this initial optimisation phase. Accordingly, the effect of the reaction solvent, temperature, and concentration on the resulting deuterium incorporation level was next investigated with substrate 12. As shown in Table 2, toluene and fluorobenzene (PhF) were revealed as more effective reaction media than MTBE at 50 °C (entries 2 and 4), with PhF, in particular, delivering a significant 76% deuterium incorporation after 16 h. 1,2-Dichloroethane (1,2-DCE) proved to be an unsatisfactory solvent for this system (entry 3). Increasing the reaction temperature to 80 °C provided a further boost in the level of deuterium incorporation to 81% D in toluene (entry 5) and an excellent 92% D in PhF (entry 7). Due to toluene being a more widely utilised solvent in organic synthesis, further optimisation relating to the reaction concentration was conducted in this solvent. Pleasingly, increasing the concentration from 0.05 M to 0.1 M in toluene resulted in the deuterium incorporation level being elevated to 91% (entry 8). A reduction in concentration, to either 0.035 M (entry 9) or 0.02 M (entry 10), had the opposite effect, resulting in more moderate deuterium incorporations of 77% and 56%, respectively. Further assessment of the reaction time and catalyst loading revealed that high levels of deuterium incorporation could be delivered after just 1 h, with labelling reaching optimal levels after around 4 h. Additionally, catalyst loading levels as low as 1 mol% were tolerated within this system; however, in the ensuing substrate scope study, 5 mol% of catalyst 2 was employed to ensure the broadest possible range of substrates could be accommodated. Full details of these latter optimisation experiments are provided in the SI.

**Table 2:** Reaction optimisation using aryl boronic pinacol esters^a^

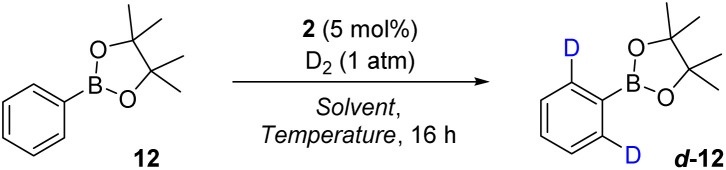				
Entry	Solvent	Temperature (°C)	Concentration (M)	% D
1	MTBE	50	0.05	26
2	Toluene	50	0.05	46
3	1,2-DCE	50	0.05	12
4	PhF	50	0.05	76
5	Toluene	80	0.05	81
6	1,2-DCE	80	0.05	26
7	PhF	80	0.05	92
8	Toluene	80	0.1	91
9	Toluene	80	0.035	77
10	Toluene	80	0.02	56

a Deuterium incorporation is based on analysis of the ^1^H NMR spectrum of the pure product and is an average of 3 reaction runs.

**Scheme 3:**
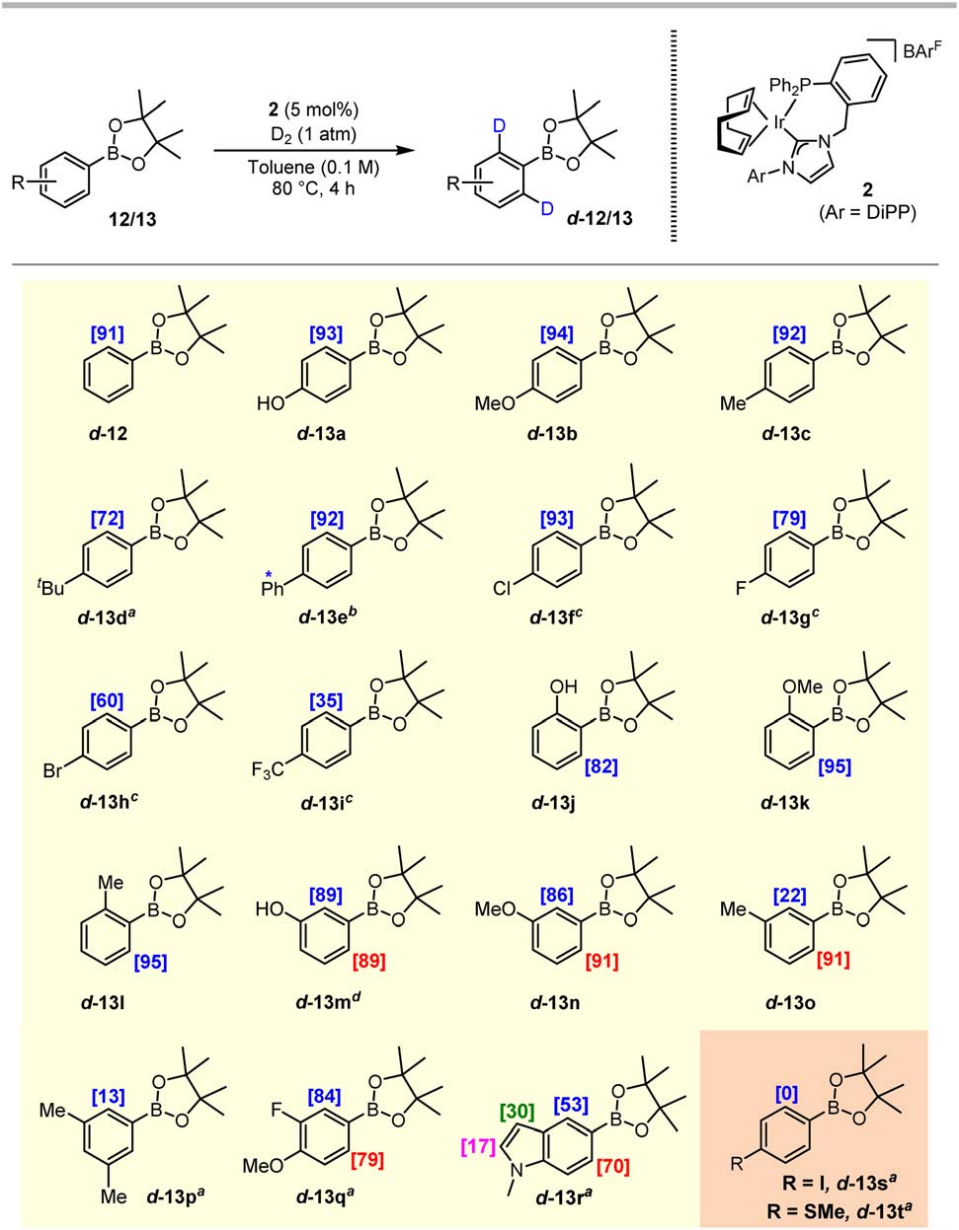
Aryl boronic pinacol ester substrate scope. Deuterium incorporation is based on analysis of the ^1^H NMR spectrum of the pure product and an average of 3 reaction runs. Isolated yields are provided within the SI. ^*a*^Reaction time: 16 h; ^*b*^16% D also observed on Ph ring (2′-position); ^*c*^reaction solvent: PhCF_3_, reaction time: 16 h; and ^*d*^deuterium incorporation is reported as an average of both *ortho*-sites.

With optimised reaction conditions in hand, the scope of our developing system was investigated through the application of an array of substituted aryl pinacol boronate esters exhibiting varying steric and electronic profiles (Scheme 3). Electron-rich arenes with substituents positioned *para* to the boron-based directing group resulted in excellent levels of deuterium incorporation, with >90% D achieved after just 4 h for products d-13a–d-13c, and an appreciable 72% D delivered for the more sterically hindered *t*-Bu analogue d-13d, despite an extended reaction time of 16 h being required. Alongside the excellent 92% D observed at the desired position in d-13e, concomitant labelling was also realised at the biphenyl-2′ position, albeit at a low 16%. Halide-substituted arenes (13f–13h) and electron-deficient substrates required more extended reaction times and the use of PhCF_3_ as the reaction medium (see the SI for the full solvent assessment for these particular substrates). Nonetheless, *para*-halide substituted compounds were labelled with good to excellent levels of incorporation; with the electron-poor 4-CF_3_ derivative, 13i, a lower deuterium incorporation of 35% was achieved. *ortho*-Substituents were also well-tolerated, delivering excellent levels of deuterium labelling in d-13j–d-13l under the standard reaction conditions. *meta*-Substituted products d-13m–d-13o were also obtained with good levels of labelling, with more favourable labelling at the least hindered *ortho*-position being observed with substrate 13o. Heavier substitution on the arene ring, such as in 3,5-dimethylphenyl-BPin 13p, led to a reduced labelling level of 13% D. In contrast, with 3,4-substituted arene 13q excellent levels of labelling were delivered despite the disubstitution. Finally, the successful labelling of the indole pinacol boronate ester species 13r extended the scope to this heterocyclic core, with exchange being favoured at the *ortho*-positions, and some labelling also being observed at the C-2 and C-3 positions of the indole unit. Isolated yields of the deuterated products shown in Scheme 3 were in the range 73–99% and, in every instance, no evidence of any C–B bond cleavage was observed. Substrates found to be less compatible with this process included the 4-iodo compound 13s and 4-thiomethyl derivative 13t, both of which resulted in full recovery of unlabelled starting material. In the case of 13t, it is anticipated that the sulfur atom may coordinate irreversibly to the catalyst, thus preventing required turnover. The outcome with substrate 13s is less readily explained; although, lower levels of labelling have been noted previously with such 4-iodoaryl substrates.^14^ In addition, the 2-furyl pinacol boronate ester was also investigated; however, no evidence of deuterium incorporation was observed, and the reaction exhibited poor mass balance with notable impurities.

**Scheme 4:**
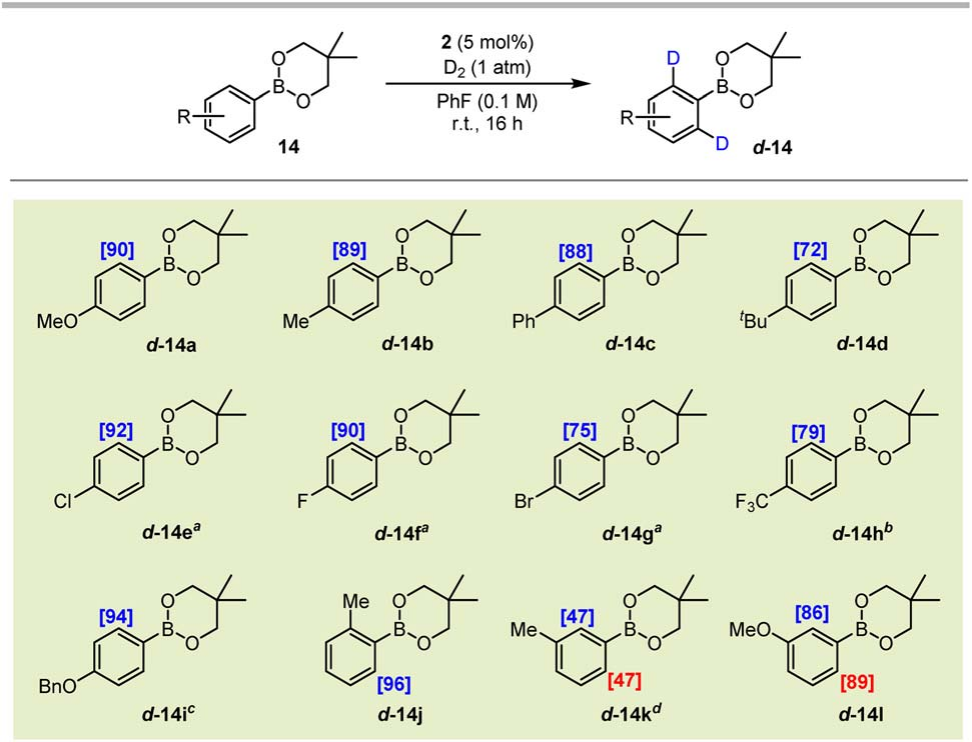
Hydrogen isotope exchange of aryl boronic neopentyl esters. Deuterium incorporation is based on analysis of the ^1^H NMR spectrum of the pure product and is an average of 3 reaction runs. ^*a*^Reaction temperature: 50 °C; ^*b*^reaction temperature: 80 °C; ^*c*^45% D also observed at the *ortho*-sites on the Ph ring of the OBn group; and ^*d*^deuterium incorporation is reported as an average of both *ortho*-sites.

Following successful labelling of a range of pinacol boronic esters, we next extended the application of the system to neopentyl glycol derivatives (BNeo). Pleasingly, after a short optimisation, aryl BNeo substrates of type 14 could be isotopically-labelled to excellent levels under even milder conditions than their corresponding BPin analogues (Scheme 4). Specifically, the electron-rich BNeo substrates 14a–14d and 14i–14l underwent HIE at room temperature in fluorobenzene, delivering up to 96% deuterium incorporation. Notably, and in contrast to its BPin analogue, the 4-phenyl substrate 14c displayed no off-site labelling on the 4-phenyl ring, indicating that the non-directed labelling pathway is likely dependent on an elevated temperature to proceed. Less electron-rich substrates 14e–14g also labelled with excellent incorporation in PhF, albeit requiring a reaction temperature of 50 °C to reach such levels of labelling. Pleasingly, the electron-deficient CF_3_-containing boronate substrate 14h could also be labelled to an appreciable 79% incorporation at 80 °C, far outperforming the corresponding BPin analogue 13i, which labelled to a maximum of 35% D.

**Scheme 5:**
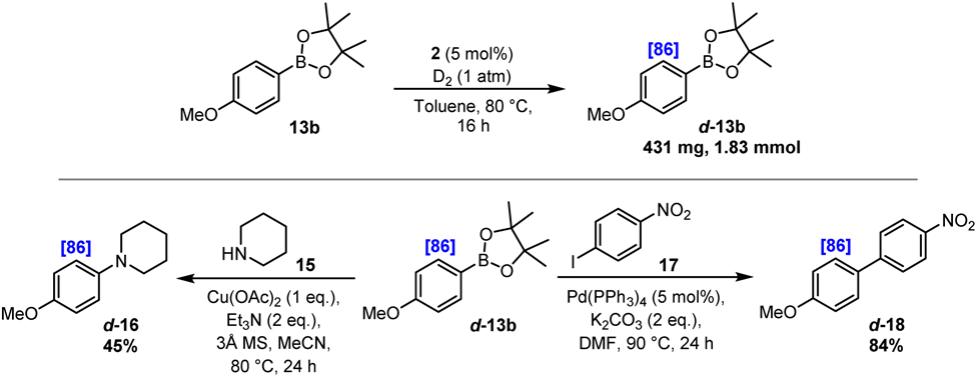
Access to tracelessly labelled arenes.

As described, specific iridium-based catalysis protocols have been established for efficient C–H activation and selective HIE directed by common and readily accessible boronic ester units. In addition, the isotopically labelled products delivered by this method possess a preparatively flexible synthetic boron-based handle that is primed for further diversification. Related to this, we envisioned that the boronic ester functionality could be utilised in subsequent cross-coupling reactions to deliver further valuable products with unique labelling patterns and, indeed, with isotopic substitution in positions that would normally be less accessible due to the absence of any directing functionality, *i.e.* leading to an overall labelling procedure that could be considered as ‘traceless’. To demonstrate this, *para*-methoxy derivative d-13b was prepared on approx. 2 mmol scale with 86% D (see the SI for full details). Subsequently and as described in Scheme 5, a Chan–Lam cross-coupling reaction was performed with piperidine 15 to deliver the aminated product d-16 in moderate yield, yet with the high level of deuterium incorporation completely preserved. In a similar vein, d-13b was reacted under Suzuki–Miyaura conditions with aryl iodide 17, delivering the biaryl product d-18 in 84% yield. Again, this transformation was accomplished with complete retention of the deuterium label, and with specific isotopic substitution within only one of the two arene units, which would be appreciably more challenging to achieve in such a direct and selective fashion using existing methods.

**Scheme 6:**
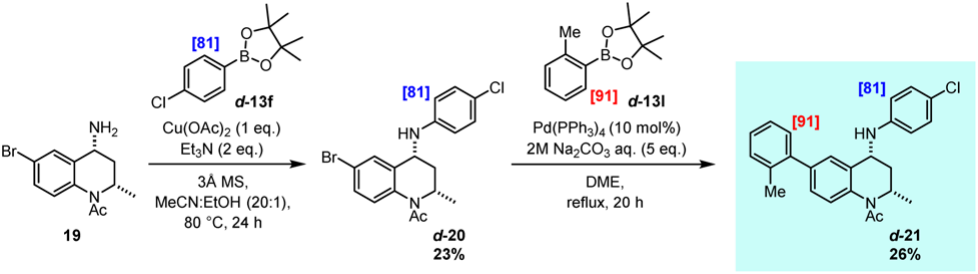
Synthesis of a labelled drug molecule.

Finally, given the recent focus on the strategic placement of deuterium within drug molecules, the practical application of the developed methodology to the preparation of an isotopically labelled active pharmaceutical was demonstrated (Scheme 6). In this regard, the bromodomain inhibitor 21^16^ was identified as an ideal target for this approach, with the synthetic pathway involving late-stage Chan–Lam and Suzuki–Miyaura cross-coupling reactions as the final two steps in the preparation of a specifically labelled analogue. Accordingly, intermediate 19 was subjected to Chan–Lam amination with previously prepared isotopologue d-13f, providing the aryl amine product, d-20, with levels of deuterium labelling preserved. The ensuing Suzuki–Miyaura reaction utilised isotopologue d-13l to deliver the polydeuterated target molecule d-21, selectively labelled at otherwise less accessible sites. This preparative scheme further demonstrates both the effectiveness of this directed deuteration method and the utility of the accessed specifically labelled boronic ester units within modern cross-coupling reactions as employed towards the efficient synthesis of biologically relevant structures.

## Conclusions

In conclusion, a novel system for the HIE of boronic ester substrates has been developed, representing, to the best of our knowledge, the first example of a C–H activation protocol utilising common and synthetically versatile boron-containing compounds as the directing functionality. The methodology has a broad scope that is tolerant to substituents across a range of steric and electronic parameters, and which delivers excellent levels of deuterium incorporation. Modifications to the boronic ester backbone are also tolerated, with neopentyl boronate esters also providing active boron-based functionality, alongside the corresponding pinacol units. Finally, the applicability of this process was demonstrated *via* the synthesis of a number of tracelessly labelled products, including an isotopically-enriched biologically active molecule, in which deuterium incorporation is retained through the subsequent synthetic transformations to provide products with previously less accessible site-specific labelling patterns.

## Author contributions

JCT: methodology, formal analysis, investigation, data curation. CS: methodology, formal analysis, investigation, data curation. DML: conceptualisation, supervision. GML: resources, supervision, project administration. NDM: resources, supervision, project administration. LCP: writing – original draft, supervision, project administration. WJK: conceptualisation, resources, supervision, funding acquisition, writing – editing.

## Conflicts of interest

There are no conflicts to declare.

## Supplementary Material

SC-OLF-D5SC09113F-s001

## Data Availability

The data that support the findings of this study are available in the supplementary information (SI) of this article. Supplementary information: full experimental and computational details. See DOI: https://doi.org/10.1039/d5sc09113f.
