# Supplementary material for: Widely-used boronic esters as synthetically-versatile directing groups for C–H activation and hydrogen isotope exchange
Source: Chem Sci. 2026 Jan 14;17(10):5213–9. doi: 10.1039/d5sc09113f (PMC12817062; doi:10.1039/d5sc09113f)
Supplement: SC-017-D5SC09113F-s001 [file SC-017-D5SC09113F-s001.pdf]

This journal is © The Royal Society of Chemistry 2026

Supporting Information

Accessible Boronic Esters as Dynamic Directing Groups for C-H Activation and Hydrogen Isotope Exchange

J. Conor Townsley,[a] Connor Smith,[a] David M. Lindsay,[a] Gemma M. Liwicki,[b] Nicholas D. Measom,[b] Laura C. Paterson[a] and William J. Kerr[a]*

[a] Department of Pure and Applied Chemistry, University of Strathclyde, 295 Cathedral Street, Glasgow, Scotland, G1 1XL (UK). *Corresponding author. E-mail: w.kerr@strath.ac.uk; Homepage: https://williamjkerr.com/.

[b] GSK, Medicines Research Centre, Gunnels Wood Road, Stevenage, Hertfordshire, England, SG1 2NY (UK).

Table of Contents

1 Experimental *4*

1.1 General Information **4**

1.2 List of General Procedures **6**

1.3 Computational Details **9**

1.3.1 General considerations 9

1.3.2 Counterpoise method for binding energy calculations 9

1.4 Catalyst Synthesis **11**

1.4.1 Towards [Ir(COD)(PPh_3)(IMes)]BAr^F **1** 11

1.4.2 Towards η^4-cycloocta-1,5-diene(3-(2-(diphenylphosphanyl)ethyl)-1-mesitylimidazole-2-ylidene)iridium tetrakis[3,5-bis(trifluoromethyl)phenyl]borate **7** 13

1.4.3 Towards η^4-cycloocta-1,5-diene(1-(2,6-diisopropylphenyl)-3-(2-(diphenylphosphanyl)ethyl) imidazole-2-ylidene)iridium BAr^F **8** 17

1.4.4 Towards η^4-cycloocta-1,5-diene(1-(2,6-diisopropylphenyl)-3-(2-(diphenylphosphanyl)benzyl) imidazole-2-ylidene)iridium BAr^F **2** 22

1.5 Catalyst Screening **27**

1.5.1 Substrate synthesis 27

1.5.2 Catalyst screening of phenylboronic acid **10** (Manuscript, Table 1) 28

1.5.3 Catalyst screening of 2-phenyl-1,3,2-dioxaborolane **11** (Manuscript, Table 1) 29

1.5.4 Catalyst screening of 4,4,5,5-tetramethyl-2-phenyl-1,3,2-dioxaborolane **12** (Manuscript, Table 1). 30

1.6 Optimisation **32**

1.6.1 Solvent and temperature screen (Manuscript, Table 2) 32

1.6.2 Concentration screen (Manuscript, Table 2) 33

1.6.3 Time study 33

1.6.4 Catalyst loading investigation 35

1.7 Substrate Scope – Aryl Boronic Pinacol Esters (Aryl-BPins) **37**

1.7.1 Substrate synthesis 37

1.7.2 Hydrogen isotope exchange of aryl-BPins (Manuscript, Scheme 3) 53

1.8 Substrate Scope – Aryl Boronic Neopentyl Esters (Aryl-BNeos) **68**

1.8.1 Substrate synthesis 68

1.8.2 Hydrogen isotope exchange of aryl-BNeos (Manuscript, Scheme 4) 78

1.9 Application of Labelled Boronates **87**

1.9.1 Large scale synthesis of ***d*-13b** (Manuscript, Scheme 5) 87

1.9.2 Chan-Lam coupling of ***d*-13b** (Manuscript, Scheme 5) 89
1.9.3 Suzuki-Miyaura cross-coupling of ***d*-13b** (Manuscript, Scheme 5) 90
1.9.4 Synthesis of a labelled drug molecule (Manuscript, Scheme 6) 91
1.10 References 101
1.11 Appendix 107
1.11.1 Scanned Spectra 107
1.11.2 Cartesian coordinates (Å) for all Optimised Structures 266

1 Experimental

1.1 General Information

All reagents were obtained from commercial suppliers (Alfa Aesar, Sigma-Aldrich, Apollo Scientific, Fluorochem, or Strem) and were used without further purification, unless otherwise stated. If purification was required, this was carried out using standard laboratory methods.[1] All glassware was flame-dried under vacuum and allowed to cool under a stream of argon, unless otherwise stated. Catalyst **9** was obtained in-house, having been prepared using a previously published procedure.[2]

Tetrahydrofuran was purified either by using a PureSolv SPS-400-5 Solvent Purification System (SPS) or by heating to reflux over sodium wire with benzophenone ketyl as the indicator, before being distilled under a nitrogen atmosphere. All other solvents were distilled (if required) over calcium hydride under an argon atmosphere and stored over molecular sieves. Petroleum ether refers to this solvent with a boiling point range of 40–60 °C.

Thin layer chromatography was carried out using Camlab silica plates coated with fluorescent indicator UV254. Plates were analysed using a Mineralight UVGL-25 lamp or developed using vanillin solution. Flash chromatography was carried out using Prolab silica gel (230-400 µm mesh).

IR spectra were obtained on a Perkin Elmer Spectrometer 1. All samples were analysed neat, unless otherwise states, and peaks are reported in cm^{-1}.

^{1}H, ^{13}C, ^{11}B, ^{19}F, and ^{31}P NMR spectra were recorded on a Bruker DPX 400 spectrometer at 400 MHz, 101 MHz, 128 MHz, 376 MHz, and 162 MHz, respectively. It should be noted that NMR spectra were run in borosilicate glass tubes and, as such, this led to peak broadening in ^{11}B NMR spectra. Chemical shifts (δ) are reported in ppm relative to the residual protic solvent. Coupling constants (J) are reported in Hz and refer to $^{3}J_{HH}$ interactions, unless stated otherwise. Analysis of deuterated material was carried out by ^{1}H NMR with an extended relaxation time (D1 = 20

seconds). Where a result was recorded as an average, this refers to an average value from at least two runs, unless stated otherwise.

High resolution mass spectrometry (HRMS) was carried out at the Mass Spectrometry Facility, University of Strathclyde or the Mass Spectrometry Facility, University of Edinburgh.

Density functional theory (DFT) calculations were conducted using the ARCHIE-WeSt High-Performance Computer (www.archie-west.ac.uk) based at the University of Strathclyde.

1.2 List of General Procedures

General Procedure A – Synthesis of Boronic Esters

Adapted from a literature procedure.[3] To a round-bottom flask under air was added the respective arylboronic acid (1 eq.) and DCM. To the suspension was added the respective diol (1.1 eq.) and $MgSO_4$ (1 eq.), and the reaction mixture was stirred at room temperature for 16 h. After this time, the reaction mixture was filtered, washed with DCM, and concentrated *in vacuo*. The crude product was purified by flash chromatography, with 0-20% EtOAc in petroleum ether as the eluent, to provide the desired boronic ester.

General Procedure B – Catalyst Screen

Reactions were performed using a Radley's 12 plus reaction station. Each of the carousel tubes were dried overnight in an oven at 180 °C and allowed to cool under vacuum before use. The tubes were subjected to an argon atmosphere before being charged with the relevant boron-containing compound (0.107 mmol) and catalyst (5 mol%). To each tube was added MTBE (2 mL, 0.05 M) and the solutions were cooled to -78 °C using dry ice/acetone. The atmosphere was exchanged with three vacuum/D_2(g) cycles introduced *via* balloon, the tubes were then sealed and immediately placed in a heating block, pre-heated to 50 °C. The reactions were stirred for 16 h, after which time, MeCN (0.2 mL) was added to the tubes to deactivate the catalyst. The reaction mixtures were transferred to a vial and concentrated *in vacuo*. The residue was then either analysed crude or, in the case of pinacol boronic esters, was purified by passing through a short silica gel column, with 20% Et_2O/petroleum ether as the eluent, to afford the deuterated product. The level of deuterium incorporation was determined by ^{1}H NMR spectroscopic analysis of the products, with the integrals of the anticipated labeling positions measured against a peak corresponding to a position where labeling was not expected. The level of deuteration was then calculated using Equation **1** displayed below:

$$Deuteration\ (\%) = 100 - \left[100 \times \left(\frac{residual\ integral}{expected\ integral}\right)\right] \qquad \mathbf{1}$$

General Procedure C – HIE Optimization

Reactions were performed using a Radley's 12 plus reaction station. Each of the carousel tubes were dried overnight in an oven at 180 °C and allowed to cool under vacuum before use. Each tube was charged with 4,4,5,5-tetramethyl-2-phenyl-1,3,2-dioxaborolane **12** (21.8 mg, 0.107 mmol) and the pre-catalyst **2**. To each tube was added the indicated solvent and the solutions were cooled to -78 °C using dry ice/acetone. The atmosphere was exchanged with three vacuum/D_2(g) cycles introduced *via* balloon, the tubes were sealed, and immediately placed in a heating block, pre-heated to the indicated temperature. The reactions were stirred for the indicated time period, after which time MeCN (0.2 mL) was added to the tubes to deactivate the catalyst. The reaction mixtures were transferred to a vial and concentrated *in vacuo*. The residue was then purified by passing through a short silica gel column, with 20% Et_2O/petroleum ether as the eluent, to afford the deuterated product. The level of deuterium incorporation was determined by ^{1}H NMR spectroscopic analysis of the products, with the integrals of the anticipated labeling positions measured against a peak corresponding to a position where labeling was not expected. The level of deuteration was then calculated using Equation **1** displayed below:

$$Deuteration\ (\%) = 100 - \left[100 \times \left(\frac{residual\ integral}{expected\ integral}\right)\right] \qquad \mathbf{1}$$

General Procedure D – HIE Substrate Scope

Reactions were performed using a Radley's 12 plus reaction station. Each of the carousel tubes were dried overnight in an oven at 180 °C and allowed to cool under vacuum before use. Each tube was charged with the appropriate boron-containing substrate (0.107 mmol) and the pre-catalyst **2** (8.9 mg, 0.00535 mmol, 5 mol%). To each tube was added the indicated solvent (1 mL, 0.1 M) and the solutions were cooled to -78 °C using dry ice/acetone. The atmosphere was exchanged with three vacuum/D_2(g) cycles introduced *via* balloon, the tubes were sealed, and immediately placed in a heating block, pre-heated to the indicated temperature. The reactions were stirred for the indicated time period, after which time MeCN (0.2 mL) was added to the tubes to deactivate the catalyst. The reaction mixtures were transferred to a vial and

concentrated *in vacuo*. The residue was then purified by passing through a short silica gel column, with 20% Et_2O/petroleum ether as the eluent, to afford the deuterated product. The level of deuterium incorporation was determined by ^{1}H NMR spectroscopic analysis of the products, with the integrals of the anticipated labelled positions measured against a peak corresponding to a position where labeling was not expected. The level of deuteration was then calculated using Equation **1** displayed below:

$$Deuteration\ (\%) = 100 - \left[100 \times \left(\frac{residual\ integral}{expected\ integral}\right)\right] \qquad \mathbf{1}$$

1.3 Computational Details

1.3.1 General considerations

DFT calculations were conducted using the ARCHIE-WeSt regional High Performance Supercomputer centre based at the University of Strathclyde (www.archie-west.ac.uk). Gaussian 16 software was used for all DFT calculations to determine the gas-phase structures and energies of all species detailed within this manuscript.[4] Geometries were constructed in GaussView 6 and the optimization was conducted using the meta-GGA exchange correlation functional M06L in combination with the 6-311G(d,p) basis set for all geometry optimization and transition states (TS) considering main group atoms, with iridium described by the Stuttgart RSC effective core potential and all its associated basis sets.[5] The same level of theory was also used to calculate the harmonic vibrational frequencies of respective minima (no imaginary frequencies) and first order saddle points (TSs, one imaginary frequency). Coordinates for optimized structures are provided in the Appendix in a multi-structure XYZ format with titles matching the compound number denoted in the manuscript.

1.3.2 Counterpoise method for binding energy calculations

Counterpoise method for binding energy calculation requires optimized structures of the free complex (**A**/**A'**), free substrate (**B**) and the catalyst substrate complex (**AB**/**A'B**),[6] as shown below.

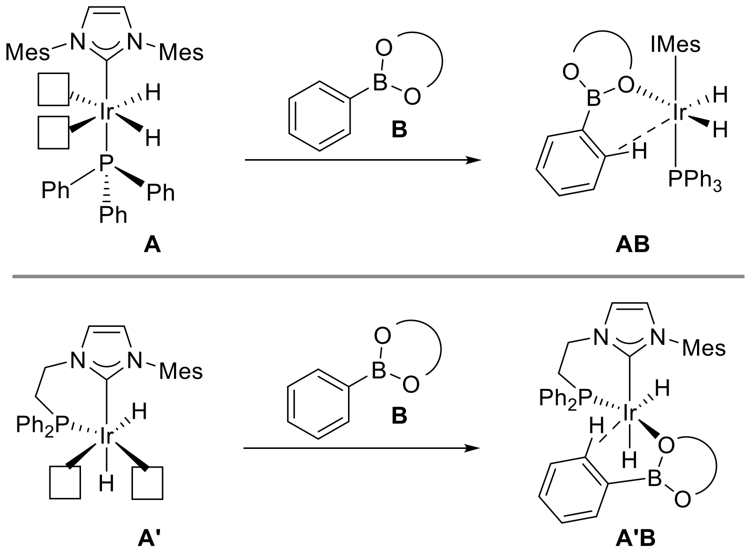

The electronic energy for each of these structures was then used in Equation 2, in order to deliver the binding energy (E_{bind}).

$$E_{bind} = \left[E_{AB}^{\alpha\beta}(AB) - E_{AB}^{\alpha\beta}(A) - E_{AB}^{\alpha\beta}(B)\right] + \left[\left(E_{AB}^{\alpha}(A) - E_{A}^{\alpha}(A)\right) + \left(E_{AB}^{\beta}(B) - E_{B}^{\beta}(B)\right)\right] \quad \mathbf{2}$$

Key: $E_{geometry}^{basis\ set}(structure)$

Equation **2** can be simplified into terms describing the counterpoise corrected interaction energy (E_{int}) and the sum of distortion energies (E_{dist}), as shown in Equation **3**.

$$E_{bind} = E_{int} + E_{dist} \quad \mathbf{3}$$

1.4 Catalyst Synthesis

1.4.1 Towards [Ir(COD)(PPh_3)(IMes)]BAr^F **1**

[Ir(COD)(IMes)Cl] S1[7]

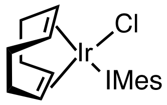

S1

To a flame-dried Schlenk tube, under an argon atmosphere, was added $[Ir(COD)Cl]_2$ (1.00 g, 1.49 mmol, 0.5 eq.) and KO^{t}Bu (335 mg, 2.98 mmol, 1 eq.). The solids were stirred under vacuum for 10 min. After this time, dry THF (25 mL) was then added and the mixture was stirred under argon for a further 10 min. 1,3-Dimesityl-1*H*-imidazol-3-ium chloride (1.02 g, 2.98 mmol, 1 eq.) was then added and the reaction mixture was stirred under argon for 4 h. The solvent was then removed *in vacuo* and the crude residue was purified by column chromatography, eluting with 50% EtOAc/petroleum ether, to afford [Ir(COD)(IMes)Cl] **S1** (1.47 g, 2.30 mmol, 77%) as a yellow solid.

Melting point: 196 – 198 °C (Lit.: decomp. >200 °C).[8]

FTIR (neat): 2970, 2932, 2874, 1612, 1463, 1354, 1273, 1136, 1119, 885, 712 cm^{-1}

^{1}H NMR (400 MHz, $CDCl_3$): δ 7.04 - 6.96 (m, 2H, NC$\underline{H}$=C$\underline{H}$N), 6.95 (s, 4H, Ar$\underline{H}$), 4.19 - 4.12 (m, 2H, COD C$\underline{H}$), 3.01 - 2.94 (m, 2H, COD C$\underline{H}$), 2.36 (s, 12H, *o*-C$\underline{H}_3$), 2.16 (s, 6H, *p*-C$\underline{H}_3$), 1.79 - 1.59 (m, 4H, COD C$\underline{H}_2$), 1.40 - 1.20 (m, 4H, COD C$\underline{H}_2$) ppm.

^{13}C NMR (101 MHz, $CDCl_3$): δ 180.9, 138.8, 137.5, 136.2, 134.5, 129.7, 128.3, 123.4, 82.7, 51.6, 33.7, 29.1, 21.3, 19.8, 18.4 ppm.

[Ir(COD)(PPh$_3$)(IMes)]BAr^F 1[8]

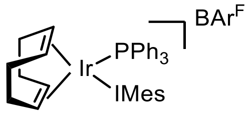

1

To a flame-dried Schlenk tube, under an argon atmosphere, was added [Ir(COD)(IMes)Cl] **S1** (200 mg, 0.31 mmol, 1.0 eq.) and NaBAr^F (276 mg, 0.31 mmol, 1 eq.). The mixture was dissolved in dry DCM (10 mL) and stirred at room temperature for 30 min. After this time, PPh$_3$ (82 mg, 0.31 mmol, 1 eq.) was added, resulting in an orange to red colour change, and the reaction mixture was stirred for a further 30 min at room temperature. The reaction mixture was then concentrated *in vacuo* and purified by column chromatography, eluting with 50% DCM/petroleum ether, to afford [Ir(COD)(PPh$_3$)(IMes)]BAr^F **1** (379 mg, 0.22 mmol, 71%) as a red crystalline solid.

Melting point: 152 – 154 °C (Lit.: decomp. >156 °C).[8]

FTIR (neat): 2957, 2916, 1609, 1483, 1437, 1350, 1277, 1157, 1121, 885, 683 cm^{-1}.

^{1}H NMR (400 MHz, CDCl$_3$): δ 7.73 (s, 8H, BAr^F Ar$\underline{H}$), 7.53 (s, 4H, BAr^F Ar$\underline{H}$), 7.49 - 7.40 (m, 3H, Ar$\underline{H}$), 7.38 - 7.25 (m, 8H, Ar$\underline{H}$ and NC$\underline{H}$=C$\underline{H}$N), 7.18 - 7.09 (m, 6H, Ar$\underline{H}$), 7.05 (s, 2H, Ar$\underline{H}$), 6.69 (s, 2H, Ar$\underline{H}$), 4.41 - 4.34 (m, 2H, 2 × COD C$\underline{H}$), 3.42 - 3.32 (m, 2H, 2 × COD C$\underline{H}$), 2.36 (s, 6H, 2 × ArCH$_3$), 2.10 (s, 6H, 2 × ArC$\underline{H}_3$), 1.77 (s, 6H, 2 × ArC$\underline{H}_3$), 1.72 - 1.47 (m, 6H, COD C$\underline{H}_2$), 1.35 - 1.25 (m, 2H, COD C$\underline{H}_2$) ppm.

^{13}C NMR (101 MHz, CDCl$_3$): δ 178.1 (d, $^3J_{C\text{-}P}$ = 8.1 Hz), 161.7 (q, $^1J_{C\text{-}B}$ = 50.4 Hz), 140.2, 135.6, 135.2, 134.9, 134.8, 131.4, 131.3, 130.8, 130.6, 129.9, 129.0 (d, $^2J_{C\text{-}F}$ = 31.2 Hz), 128.7 (d, $^2J_{C\text{-}P}$ = 10.0 Hz), 124.7 (d, $^1J_{C\text{-}F}$ = 272.3 Hz), 117.6, 80.6, 80.5, 78.7, 31.9, 21.2, 20.9, 19.0 ppm.

^{31}P NMR (162 MHz, CDCl$_3$): δ 16.38 ppm.

^{19}F NMR (376 MHz, CDCl$_3$): δ -62.43 ppm.

^{11}B NMR (128 MHz, CDCl$_3$): δ -6.69 ppm.

1.4.2 Towards η^4-cycloocta-1,5-diene(3-(2-(diphenylphosphanyl)ethyl)-1-mesitylimidazole-2-ylidene)iridium tetrakis[3,5-bis(trifluoromethyl)phenyl]borate **7**

1-Mesityl-1*H*-imidazole S2[9]

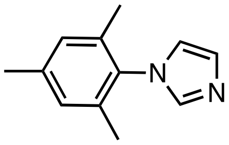

S2

To a 500 mL, three-necked, round-bottom flask equipped with a condenser and addition funnel, was added glyoxal (40% wt. in water, 16.3 mL, 142 mmol, 1.1 eq.), formaldehyde (37% wt. in water, 10.8 mL, 145 mmol, 1.1 eq.), and acetic acid (35.0 mL). The reaction mixture was heated to 80 °C. In a separate conical flask was added NH_4OAc (10.9 g, 140 mmol, 1.1 eq.), 2,4,6-trimethylaniline (18.3 mL, 130 mmol, 1 eq.), acetic acid (35 mL), and water (3.00 mL), and the mixture was stirred until a viscous solution formed. The resulting solution was transferred to the addition funnel and slowly added over a period of 10 minutes. After this time, the reaction was stirred for 16 h at 80 °C before being cooled to room temperature and transferred to a dropping funnel. The solution was then added to an excess amount of aqueous saturated sodium bicarbonate solution with very vigorous stirring. Once the quench was completed, the aqueous suspension was then filtered and the filter cake washed with water and allowed to dry in air. The filtrand was transferred to a beaker and petroleum ether was added. The suspension was heated to boiling and the hot solution decanted. This process was repeated until petroleum ether remained clear upon boiling, at which point, the remaining solid residue was discarded. The decanted liquid was concentrated *in vacuo* to yield mesityl imidazole **S2** (10.27 g, 55.1 mmol, 38%) as a brown solid.

Melting point: 112 – 114 °C (Lit.: 112 – 113 °C).[9]]

FTIR (neat): 3093, 2978, 2920, 2860, 1593, 1498 cm^{-1}.

^{1}H NMR (400 MHz, $CDCl_3$): δ 7.44 (t, ^4J = 1.1 Hz, 1H, ArH), 7.23 (t, ^4J = 1.1 Hz, 1H, ArH), 6.97 (s, 2H, ArH), 6.89 (t, ^4J = 1.3 Hz, 1H, ArH), 2.34 (s, 3H, $ArCH_3$), 1.99 (s, 6H, $ArCH_3$) ppm.

^{13}C NMR (101 MHz, $CDCl_3$): δ 139.0, 137.6, 135.5, 133.5, 129.7, 129.1, 120.2, 21.1, 17.4 ppm.

3-(2-Hydroxyethyl)-1-mesityl-1*H*-imidazol-3-ium bromide S3[10]

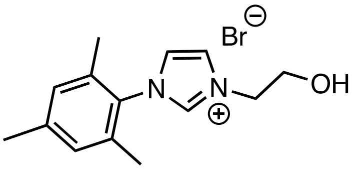

S3

To a 100 mL round-bottom flask was added mesityl imidazole **S2** (5 g, 26.8 mmol, 1 eq.). Toluene (80 mL) was added to dissolve the brown solid, followed by 2-bromoethanol (2.4 mL, 33.5 mmol, 1.25 eq.). The resulting reaction mixture was heated to reflux for 16 h, after which time the reaction was cooled to 0 °C and the resulting precipitate was allowed to settle. The solvent was then removed by pipette and the solid product washed with diethyl ether, yielding 3-(2-hydroxyethyl)-1-mesitylimidazolium bromide **S3** (6.88 g, 22.1 mmol, 82%) as a brown solid.

Melting point: 161 – 163 °C (Lit.: 164 – 166 °C).[11]
FTIR (neat): 3307, 3057, 3026, 2980, 2945, 2906, 2358, 1710, 1436 cm^{-1}.
^{1}H NMR (400 MHz, *d*$_6$-DMSO): δ 9.44 – 9.38 (m, 1H, NCHN), 8.06 (q, ^{4}J = 1.6 Hz, 1H, ArH), 7.92 (t, ^{4}J = 1.8 Hz, 1H, ArH), 7.14 (s, 2H, ArH), 4.34 (t, *J* = 5.1 Hz, 2H, CH_2), 3.81 (t, *J* = 5.1 Hz, 2H, CH_2), 2.33 (s, 3H, $ArCH_3$), 2.02 (s, 6H, $ArCH_3$) ppm.
^{13}C NMR (101 MHz, *d*$_6$-DMSO): δ 140.2, 137.7, 134.3, 131.2, 129.2, 123.7, 123.4, 59.1, 52.0, 20.6, 16.9 ppm.

3-(2-Bromoethyl)-1-mesityl-1*H*-imidazol-3-ium bromide S4[11]

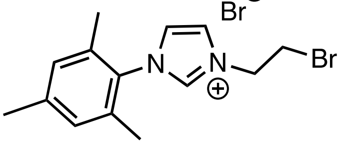

S4

To a 100 mL round-bottom flask was added 3-(2-hydroxyethyl)-1-mesityl imidazolium bromide **S3** (3 g, 9.62 mmol, 1 eq.) and DCM (50 mL). The solution was cooled to 0 °C and PBr_3 (0.84 mL,

8.34 mmol, 0.87 eq.) was added dropwise. The reaction was stirred at room temperature for 16 h, and then cooled to 0 °C and quenched with saturated aqueous sodium bicarbonate solution. The mixture was then extracted with DCM and the organic layers were collected, dried with Na_2SO_4, and concentrated *in vacuo* to yield 3-(2-bromoethyl)-1-mesityl imidazolium bromide **S4** (2.06 g, 5.52 mmol, 57%) as a brown solid.

Melting point: 165 – 167 °C (Lit.: 154 – 156 °C).[11]

FTIR (neat): 3118, 3032, 2970, 1862, 1546, 1444 cm^{-1}.

^{1}H NMR (400 MHz, $CDCl_3$): δ 10.26 (t, $^4J_{HH}$ = 1.5 Hz, 1H, NCHN), 7.94 (t, ^4J = 1.6 Hz, 1H, ArH), 7.15 (t, ^4J = 1.6 Hz, 1H, ArH), 7.03 (s, 2H, ArH), 5.26 (t, J = 5.4 Hz, 2H, CH_2), 4.06 (t, J = 5.2 Hz, 2H, CH_2), 2.36 (s, 3H, $ArCH_3$), 2.10 (s, 6H, $ArCH_3$) ppm.

^{13}C NMR (101 MHz, $CDCl_3$): δ 141.9, 138.4, 134.4, 130.6, 130.1, 124.1, 122.7, 52.2, 32.1, 21.3, 17.8 ppm.

3-(2-(Diphenylphosphanyl)ethyl)-1-mesitylimidazolium BAr^F S5[2,11]

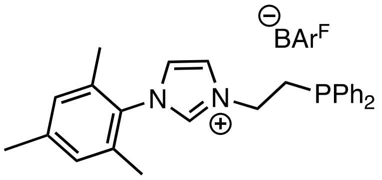

S5

A flame-dried 10 mL round-bottom flask was placed under an inert atmosphere with three vacuum/argon cycles. The flask was charged with potassium *tert*-butoxide (224 mg, 2 mmol, 1 eq.) and DMSO (3 mL). To this was added diphenylphosphine (0.34 mL, 2.1 mmol, 1.05 eq.) and the mixture was stirred for 1 h. A separate flame-dried 10 mL round-bottom flask was placed under an inert atmosphere with three vacuum/argon cycles and, subsequently, charged with 3-(2-bromoethyl)-1-mesitylimidazolium bromide **S4** (750 mg, 2 mmol, 1 eq.) and DMSO (3 mL). The DMSO solution of potassium diphenylphosphide was then added dropwise, *via* syringe, to the **S4** solution and the resultant mixture was stirred for a further 2 h. Water (30 mL) was then added to the reaction mixture and the product was extracted with DCM. The organic layer was separated, transferred to a round-bottom flask, and placed under an inert atmosphere. NaBAr^F

(1.95 g, 2.2 mmol, 1.1 eq.) was added and the reaction was stirred for 16 h at room temperature. After this time, the reaction mixture was concentrated *in vacuo*, and the product purified by column chromatography, eluting with 50% DCM/petrol, to yield 3-(2-(diphenylphosphanyl)ethyl)-1-mesitylimidazolium BAr^F, **S5** (867 mg, 0.69 mmol, 37%) as a tan solid.

Melting point: 96 – 98 °C (Lit.: 84 – 86 °C).[11]

FTIR (neat): 3126, 2978, 1608, 1546, 1438, 1354, 1273, 1117 cm^{-1}.

^{1}H NMR (400 MHz, CDCl$_3$): δ 8.08 (bs, 1H, NC<u>H</u>N), 7.68 (br s, 8H, Ar<u>H</u>, BAr^F), 7.50 (s, 4H, Ar<u>H</u> BAr^F), 7.45 - 7.27 (m, 11H, Ar<u>H</u>), 7.14 (d, J = 1.7 Hz, 1H, Ar<u>H</u>), 7.03 (s, 2H, Ar<u>H</u>), 4.34 - 4.19 (m, 2H, PC<u>H</u>$_2$), 2.61 (bs, 2H, C<u>H</u>$_2$), 2.36 (s, 3H, ArC<u>H</u>$_3$), 1.93 (s, 6H, ArC<u>H</u>$_3$) ppm.

^{13}C NMR (101 MHz, CDCl$_3$): δ 161.8 (q, $^1J_{C\text{-}B}$ = 50.2 Hz), 142.9, 134.9, 134.4, 133.8, 133.4, 132.8, 132.6, 130.4, 129.7, 129.4 (q, $^2J_{C\text{-}F}$ = 20.8 Hz), 128.9, 128.6, 125.1, 124.7 (q, $^1J_{C\text{-}F}$ = 273.3 Hz), 123.1, 117.6, 48.7, 29.2 (d, $^2J_{C\text{-}P}$ = 17.0 Hz), 21.1, 17.2 ppm.

^{31}P NMR (162 MHz, CDCl$_3$): δ -25.53 ppm.

^{11}B NMR (128 MHz, CDCl$_3$): δ -6.59 ppm.

η^4-Cycloocta-1,5-diene(3-(2-(diphenylphosphanyl)ethyl)-1-mesitylimidazole-2-ylidene)iridium tetrakis[3,5-bis(trifluoromethyl)phenyl]borate 7[2,11]

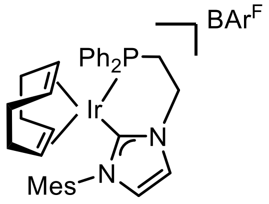

7

To a flame-dried Schlenk tube was added [Ir(COD)Cl]$_2$ (161 mg, 0.24 mmol, 0.5 eq.) and 3-(2-(diphenylphosphanyl)ethyl)-1-mesitylimidazolium BAr$_F$, **S5** (606 mg, 0.48 mmol, 1 eq.). The mixture was then dissolved in THF (4.5 mL) and ^{t}BuOK (56 mg, 0.5 mmol, 1 eq.) was added. The reaction was stirred at room temperature for 2 h, after which time the solvent was removed *in vacuo*. The resulting residue was purified by column chromatography, eluting with 50%

DCM/petrol. The resulting oil was then triturated with petrol yielding complex **7** (436 mg, 0.28 mmol, 58%) as a dusky pink solid.

Melting point: 158 – 160 °C (Lit.: 150 – 152 °C).[11]

FTIR (neat): 3126, 2978, 1608, 1546, 1438 , 1354, 1273, 1117 cm^{-1}.

^{1}H NMR (400 MHz, $CDCl_3$): δ 7.74 (bs, 8H, ArH BAr^F), 7.54 (s, 4H, ArH), 7.52 - 7.44 (m, 6H, ArH), 7.40 -7.33 (m, 4H, ArH), 6.98 (s, 2H, ArH), 6.96 (d, *J* = 1.9 Hz, 1H, ArH), 6.78 (d, *J* = 1.9 Hz, 1H, ArH), 4.64 - 4.50 (m, 2H, PCH_2), 4.40 (s, 2H, COD CH), 3.60 (s, 2H, COD CH), 2.54 - 2.45 (m, 2H, CH_2), 2.36 (s, 3H, ArCH_3), 2.02-1.77 (m, 14H, ArCH_3 + COD CH_2) ppm.

^{13}C NMR (101 MHz, $CDCl_3$): δ 170.0 (d, $^2J_{C-P}$ = 11.4 Hz), 161.9 (q, $^1J_{C-B}$ = 49.9 Hz), 140.4, 135.1, 135.0, 132.7 (d, $^2J_{C-P}$ = 10.5 Hz), 131.9, 131.6, 131.1, 129.4 (d, *J* = 8.6 Hz), 129.3, 129.0 - 128.8 (m), 128.7 - 128.5 (m), 124.7 (q, $^1J_{C-F}$ = 272.4 Hz), 124.6, 121.9, 117.6, 89.5 (d, $^2J_{C-P}$ = 11.2 Hz), 78.5, 50.6 (d, $^4J_{C-P}$ = 4.2 Hz), 31.4, 31.0, 25.5 (d, $^1J_{C-P}$ = 36.7 Hz), 21.1, 18.5 ppm.

^{31}P NMR (162 MHz, $CDCl_3$): δ 10.18 ppm.

^{11}B NMR (128 MHz, $CDCl_3$): δ -6.58 ppm.

1.4.3 Towards η^4-cycloocta-1,5-diene(1-(2,6-diisopropylphenyl)-3-(2-(diphenylphosphanyl)ethyl) imidazole-2-ylidene)iridium BAr^F 8

1-(2,6-Diisopropylphenyl)-1*H*-imidazole S6[12,13]

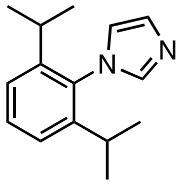

S6

To a 500 mL, three-necked, round-bottom flask equipped with a condenser and addition funnel was added glyoxal (40% wt. in water, 16.3 mL, 142 mmol, 1.10 eq.), formaldehyde (37% wt. in water, 10.8 mL, 145 mmol, 1.10 eq.) and acetic acid (35.0 mL). The reaction mixture was heated to 80 °C. In a separate conical flask was added NH_4OAc (10.9 g, 140 mmol, 1.10 eq.), 2,6-diisopropylaniline (24.5 mL, 23.05 g, 130 mmol, 1.00 eq.), acetic acid (35.0 mL) and water (3.00

mL), and the mixture was stirred until a viscous solution formed. The resulting solution was transferred to the addition funnel and slowly added over a period of 10 minutes. The reaction was stirred for 16 h at 80 °C. After this time, the reaction mixture was cooled to room temperature and transferred to a dropping funnel. The solution was then added to an excess amount of aqueous saturate sodium bicarbonate solution, with very vigorous stirring. Once the quench was completed, the aqueous suspension was then filtered, and the filter cake was washed with water and allowed to dry in air. The filtrand was transferred to a beaker and petroleum ether was added. The suspension was heated to boiling and the hot solution decanted. This process was repeated until petroleum ether remained clear upon boiling, at which point, the remaining solid residue was discarded. The decanted liquid was concentrated *in vacuo* to yield 2,6-diisopropylphenylimidazole **S6** (13.37 g, 75.4 mmol, 58%) as a brown solid.

Melting point: 122 – 124 °C (Lit.: 123 – 125 °C).[13]

FTIR (neat): 3094, 2963, 2868, 1638, 1495, 1458, 1364, 1321, 1225, 1094, 1067, 908, 810, 669 cm^{-1}.

^{1}H NMR (400 MHz, $CDCl_3$): δ 7.49 (app t, ^4J = 1.2 Hz, 1H, ArH), 7.45 (t, J = 7.8 Hz, 1H, ArH), 7.30 - 7.25 (m, 3H, ArH), 6.96 (t, J = 1.3 Hz, 1H, ArH), 2.42 (sept, J = 6.9 Hz, 2H, 2 x CH), 1.15 (d, J = 6.9 Hz, 12H, 4 x CH_3) ppm.

^{13}C NMR (101 MHz, $CDCl_3$): δ 146.8, 138.7, 133.1, 130.0, 129.6, 124.0, 121.8, 28.3, 24.7, 24.6 ppm.

1-(2,6-Diisopropylphenyl)-3-(2-hydroxyethyl)-1*H*-imidazol-3-ium bromide S7[14]

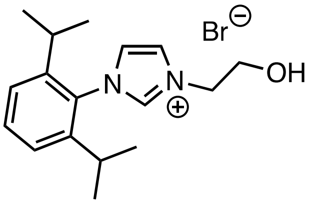

S7

To a flame-dried, 100 mL round bottomed flask was added 1-(2,6-diisopropylphenyl)-1*H*-imidazole **S6** (3.00 g, 13.1 mmol, 1 eq.). Toluene (45 mL) was added to dissolve the solid and 2-bromoethanol (1.2 mL 16.4 mmol, 1.25 eq.) was subsequently added. The resulting reaction

mixture was heated to reflux, after which time the reaction mixture was cooled to 0 °C and the resulting precipitate was allowed to settle. The solvent was then removed by pipette and the product was washed with Et_2O and filtered to give 1-(2,6-diisopropylphenyl)-3-(2-hydroxyethyl)-1*H*-imidazol-3-ium bromide **S7** (2.88 g, 8.1 mmol, 62%) as a brown solid.

Melting point: 162 – 164 °C (Lit.: 169 – 170 °C).[14]

FTIR (neat): 3285, 3048, 2963, 2866, 1562, 1543, 1460, 1186, 1074, 973, 864, 812, 764 cm^{-1}.

^{1}H NMR (400 MHz, DMSO): δ 9.54 (s, 1H, NCH=N), 8.12 (app s, 1H, NCH=CHN), 8.08 (t, *J* = 1.8 Hz, 1H, NCH=CHN), 7.62 (t, *J* = 7.7 Hz, 1H, ArH), 7.45 (d, *J* = 7.8 Hz, 2H, ArH), 5.22 (t, *J* = 5.0 Hz, 1H, OH), 4.36 (t, *J* = 5.0 Hz, 2H, CH_2), 3.82 (q, *J* = 5.1 Hz, 2H, CH_2), 2.27 (sept, *J* = 6.8 Hz, 2H, 2 x CH), 1.14 (d, *J* = 6.8 Hz, 12H, 4 × CH_3) ppm.

^{13}C NMR (101 MHz, DMSO): δ 145.2, 138.1, 131.4, 130.6, 124.9, 124.3, 123.4, 59.0, 52.0, 28.0, 23.8, 23.7 ppm.

HRMS *m/z* (ESI): Calc. for $C_{17}H_{25}N_2O$ [M^+]: 273.19614. Found 273.1951.

3-(2-Bromoethyl)-1-(2,6-diisopropylphenyl)-1*H*-imidazol-3-ium bromide S8[14]

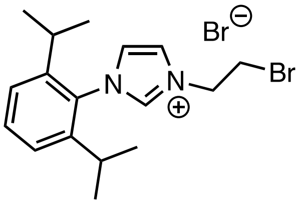

S8

To a flame-dried, 100 mL round-bottom flask was added 1-(2,6-diisopropylphenyl)-3-(2-hydroxyethyl)-1*H*-imidazol-3-ium bromide **S7** (2.00 g, 5.66 mmol, 1.0 eq.) and DCM (30 mL). The solution was cooled to 0 °C and PBr_3 (0.46 mL, 4.92 mmol, 0.87 eq.) was added dropwise. The reaction mixture was stirred at room temperature for 16 h, cooled to 0 °C, and quenched with saturated aqueous sodium bicarbonate before extracting with DCM. The organics were dried over Na_2SO_4, filtered, and concentrated *in vacuo* to yield 3-(2-bromoethyl)-1-(2,6-diisopropylphenyl)-1*H*-imidazol-3-ium bromide **S8** (1.42 g, 3.4 mmol, 60%) as an off-white solid.

Melting point: 162 – 164 °C (Lit.: 73 – 75 °C).[14]

FTIR (neat): 3034, 2963, 2868, 1547, 1458, 1366, 1192, 1059, 939, 806, 758, 640 cm^{-1}.

^{1}H NMR (400 MHz, $CDCl_3$): δ 10.07 (t, ^4J = 1.6 Hz, 1H, NC<u>H</u>=N), 8.47 (t, J = 1.7 Hz, 1H, NC<u>H</u>=CHN), 7.54 (t, J = 7.8 Hz, 1H, Ar<u>H</u>), 7.31 (d, J = 7.9 Hz, 2H, Ar<u>H</u>), 7.19 (t, J = 1.8 Hz, 1H, NCH=C<u>H</u>N), 5.31 (t, J = 5.5 Hz, 2H, C<u>H</u>$_2$), 4.08 (t, J = 5.4 Hz, 2H, C<u>H</u>$_2$), 2.34 (sept, J = 6.8 Hz, 2H, 2 x C<u>H</u>), 1.22 (d, J = 6.8 Hz, 6H, 2 × C<u>H</u>$_3$), 1.16 (d, J = 6.9 Hz, 6H, 2 × C<u>H</u>$_3$) ppm.

^{13}C NMR (101 MHz, $CDCl_3$): δ 145.6, 138.3, 132.2, 130.1, 124.9, 124.4, 123.9, 51.6, 32.3, 28.8, 24.6, 24.3 ppm.

HRMS *m/z* (ESI): Calc. for $C_{17}H_{24}{}^{79}BrN_2$ [M+]: 335.11174. Found 335.1109.

1-(2,6-Diisopropylphenyl)-3-(2-(diphenylphosphaneyl)ethyl)-1*H*-imidazol-3-ium BAr^F S9[2]

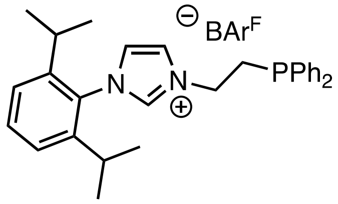

S9

To a flame-dried, 10 mL, round-bottom flask under argon was added ^{t}BuOK (224 mg, 2.0 mmol, 1.0 eq). and DMSO (3.0 mL). To this was added diphenylphosphine (0.340 mL, 2.10 mmol, 1.05 eq.) and the resulting reaction mixture was stirred for 1 h. To a separate flame dried, 10 mL round-bottom flask under argon, was added 3-(2-bromoethyl)-1-(2,6-diisopropylphenyl)-1*H*-imidazol-3-ium bromide **S8** (833 mg, 2.0 mmol, 1.00 eq.) and DMSO (3.0 mL). The DMSO solution of potassium diphenylphosphide was then added dropwise, *via* syringe, to the **S8** solution and the resulting mixture stirred for a further 2 h. Water (30.0 mL) was then added to the reaction mixture and the product was extracted with DCM. The organic layer was separated, transferred to a round-bottom flask, and placed under an inert atmosphere. NaBAr^F (1.95 g, 2.20 mmol, 1.10 eq) was added and the mixture was stirred for 16 h at room temperature. After this time, the reaction mixture was concentrated *in vacuo*, and the product purified by column chromatography, eluting with 50% DCM/petrol, to yield 1-(2,6-diisopropylphenyl)-3-(2-(diphenylphosphaneyl)ethyl)-1*H*-imidazol-3-ium BAr^F **S9** (1.47 g, 1.13 mmol, 57%) as an off-white solid.

FTIR (neat): 2967, 2870, 1545, 1460, 1354, 1275, 1109, 885, 837, 667 cm^{-1}.

^{1}H NMR (400 MHz, CDCl$_3$): δ 8.16 - 8.13 (m, 1H, NC$\underline{H}$=N), 7.69 (bs, 8H, BAr^F Ar$\underline{H}$), 7.60 (t, *J* = 7.9 Hz, 1H, Ar$\underline{H}$), 7.51 (bs, 4H, BAr^F Ar$\underline{H}$), 7.44 - 7.28 (m, 14H, Ar$\underline{H}$), 4.30 (dt, $^{2}J_{H-P}$ = 11.9 Hz, *J* = 6.9 Hz, 2H, PC$\underline{H}_2$), 2.64 - 2.58 (m, 2H, NC$\underline{H}_2$), 2.22 (sept, *J* = 6.8 Hz, 2H, 2 x C$\underline{H}$), 1.16 (d, *J* = 6.7 Hz, 6H, 2 × C$\underline{H}_3$), 1.13 (d, *J* = 6.8 Hz, 6H, 2 × C$\underline{H}_3$) ppm.

^{13}C NMR (101 MHz, CDCl$_3$): δ 161.8 (d, $^{1}J_{C-B}$ = 49.9 Hz), 145.2, 134.9, 134.4 (d, $^{2}J_{C-P}$ = 9.6 Hz), 133.1, 132.6 (d, $^{1}J_{C-P}$ = 19.8 Hz), 130.4, 129.5 (d, $^{3}J_{C-P}$ = 7.4 Hz), 129.1 (q, *J* = 30.0 Hz), 126.1, 125.4, 125.3, 124.7 (q, $^{1}J_{C-F}$ = 272.6 Hz), 122.8, 117.7, 113.4, 48.5 (d, $^{2}J_{C-P}$ = 18.9 Hz), 29.2 (d, $^{1}J_{C-P}$ = 16.7 Hz), 28.9, 24.2 ppm.

^{31}P NMR (162 MHz, CDCl$_3$): δ -26.79 ppm.

^{19}F NMR (376 MHz, CDCl$_3$): δ -62.35 ppm.

^{11}B NMR (128 MHz, CDCl$_3$): δ -6.70 ppm.

η^4-Cycloocta-1,5-diene(1-(2,6-diisopropylphenyl)-3-(2-(diphenylphosphanyl)ethyl) imidazole-2-ylidene)iridium BAr^F 8[2]

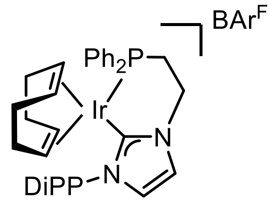

8

To a flame-dried Schlenk tube was added [Ir(COD)Cl]$_2$ (161 mg, 0.24 mmol, 0.5 eq.) and 1-(2,6-diisopropylphenyl)-3-(2-(diphenylphosphaneyl)ethyl)-1*H*-imidazol-3-ium BAr^F **S9** (626 mg, 0.48 mmol, 1 eq.). The mixture was then dissolved in THF (4.5 mL) and ^{t}BuOK (56 mg, 0.5 mmol, 1 eq.) was added. The reaction was stirred at room temperature for 2 h, after which time the solvent was removed *in vacuo*. The resulting residue was purified by column chromatography, eluting with 50% DCM/petrol. The resulting oil was then triturated with petrol yielding complex (117 mg, 0.07 mmol, 15%) as a dusky pink solid.

Melting point: 156 – 158 °C (Lit.: 158 – 160 °C).[2]

FTIR (neat): 2967, 2889, 1612, 1464, 1354, 1273, 1118, 885, 839, 667 cm^{-1}.

^{1}H NMR (400 MHz, CDCl$_3$): δ 7.71 (bs, 8H, BAr^F ArH), 7.56 - 7.44 (m, 11H, BAr^F ArH + ArH), 7.41 - 7.32 (m, 4H, ArH), 7.29 - 7.24 (m, 2H, ArH), 6.98 (d, J = 1.9 Hz, 1H NCH=CHN), 6.88 (d, J = 1.9 Hz, 1H, NCH=CHN), 4.56 - 4.45 (m, 2H, PCH$_2$), 4.33 (bs, 2H, 2 x COD CH), 3.76 (bs, 2H, 2 x COD CH), 2.51 (sept, J = 6.8 Hz, 2H, 2 x CH), 2.46 - 2.40 (m, 2H, CH$_2$), 1.97 - 1.71 (m, 8H, 4 x COD CH$_2$), 1.16 (d, J = 6.8 Hz, 6H, 2 × CH$_3$), 0.86 (d, J = 6.9 Hz, 6H, 2 × CH$_3$) ppm.

^{13}C NMR (101 MHz, CDCl$_3$): δ ^{13}C NMR (101 MHz, CDCl$_3$) δ 161.9 (q, $^1J_{C\text{-}B}$ = 49.8 Hz), 145.5, 135.0, 132.3 (d, $^2J_{C\text{-}P}$ = 10.3 Hz), 131.9, 131.8, 131.4, 131.0, 129.6 – 128.9 (m), 126.5, 124.7 (q, $^1J_{C\text{-}F}$ = 272.5 Hz), 124.6, 121.5, 117.7, 87.3 (d, $^2J_{C\text{-}P}$ = 11.4 Hz), 80.1, 50.0 (d, $^4J_{C\text{-}P}$ = 3.7 Hz), 31.3 (d, $^1J_{C\text{-}P}$ = 45.5 Hz), 29.1, 25.3, 22.7 ppm.

^{31}P NMR (162 MHz, CDCl$_3$): δ 9.00 ppm.

^{19}F NMR (376 MHz, CDCl$_3$): δ -62.40 ppm.

^{11}B NMR (128 MHz, CDCl$_3$): δ -6.58 ppm.

1.4.4 Towards η^4-cycloocta-1,5-diene(1-(2,6-diisopropylphenyl)-3-(2-(diphenylphosphanyl)benzyl) imidazole-2-ylidene)iridium BAr^F 2

1-(2-(Diphenylphosphanyl)phenyl)-*N*,*N*-dimethylmethanamine S10[15]

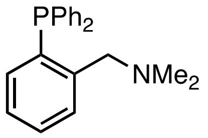

S10

A flame-dried, 3-neck, 250 mL round-bottom flask was placed under inert an atmosphere and then charged with *N,N*-dimethylbenzylamine (2.50 g, 18.5 mmol, 1.00 eq.) and Et$_2$O (60.0 mL). The solution was cooled to 0 °C and ^{n}BuLi (2.1 M in hexanes, 10.6 mL, 22.2 mmol, 1.20 eq.) was added, dropwise. The reaction mixture was then allowed to stir for 24 h at room temperature, after which time the reaction was again cooled to 0 °C. Chlorodiphenylphosphine (3.98 mL, 22.2 mmol, 1.20 eq.) was added, dropwise, and the reaction mixture was stirred at room temperature for 1 h. The reaction mixture was then quenched by the addition of MeOH (1 mL) and the mixture was transferred into a separating funnel. The product was extracted into an aqueous solution of HCl (2 M) and separated from the organic phase. The aqueous phase was then basified by

addition of NaOH, and the resulting suspension was then transferred to a separating funnel and extracted into DCM. The organic phase was dried over Na_2SO_4, filtered, and concentrated *in vacuo*. The residue was then loaded onto silica and purified by column chromatography, eluting with 50% EtOAc/hexane, to afford 1-(2-(diphenylphosphanyl)phenyl)-*N,N*-dimethylmethanamine **S10** (5.23 g, 16.4 mmol, 89%) as a colourless oil, which was stored under an inert atmosphere to prevent any oxidation.

FTIR (neat): 3051, 2968, 2938, 2770, 1433, 1252, 1175, 1026, 743, 694 cm^{-1}.

^{1}H NMR (400 MHz, $CDCl_3$): δ 7.46 (dd, *J* = 7.6 Hz, $^4J_{H\text{-}P}$ = 4.3 Hz, 1H, ArH), 7.34 - 7.22 (m, 11H, ArH), 7.15 (td, *J* = 7.5, $^4J_{H\text{-}H}$ = 1.4 Hz, 1H, ArH), 6.90 (ddd, *J* = 7.6 Hz, $^4J_{H\text{-}P}$ = 4.3 Hz, $^4J_{H\text{-}H}$ = 1.4 Hz, 1H, ArH), 3.61 (d, *J* = 2.1 Hz, 2H, CH_2), 2.06 (s, 6H, 2 x CH_3) ppm.

^{13}C NMR (101 MHz, $CDCl_3$): δ 144.2 (d, $^1J_{C\text{-}P}$ = 22.4 Hz), 137.9 (d, $^2J_{C\text{-}P}$ = 10.2 Hz), 136.8 (d, $^2J_{C\text{-}P}$ = 15.4 Hz), 134.0, 133.8, 129.2 (d, $^3J_{C\text{-}P}$ = 5.2 Hz), 128.7, 128.5, 128.4, 127.2, 62.3 (d, $^3J_{C\text{-}P}$ = 17.9 Hz), 44.8 ppm.

^{31}P NMR (162 MHz, $CDCl_3$) δ -15.26 ppm.

(2-(Chloromethyl)phenyl)diphenylphosphane S11[2]

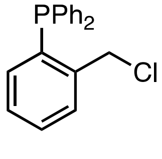

S11

To a 100 mL round-bottom flask equipped with a condenser was added 1-(2-(diphenylphosphanyl)phenyl)-*N,N*-dimethylmethanamine **S10** (5.23 g, 16.4 mmol, 1.00 eq.) and toluene (41 mL). To this, was added ethyl chloroformate (1.90 mL, 2.14 g, 19.7 mmol, 1.20 eq.) and the reaction mixture was heated to 80 °C and stirred for 2 h. The reaction mixture was then cooled to room temperature and concentrated *in vacuo*. The residue was then loaded onto silica and purified by column chromatography, eluting with DCM, to afford (2-(chloromethyl)phenyl)diphenylphosphane **S11** (4.47 g, 14.4 mmol, 88%) as a white, air stable, solid.

Melting point: 146 – 148 °C (Lit.: 147 – 149 °C).[2]

FTIR (neat): 3051, 3005, 2963, 1582, 1468, 1431, 1263, 822, 773, 675, 669 cm^{-1}.

^{1}H NMR (400 MHz, $CDCl_3$): δ 7.53 (ddd, *J* = 7.7 Hz, $^{4}J_{H\text{-}P}$ = 4.5 Hz, $^{4}J_{H\text{-}H}$ = 1.3 Hz, 1H, Ar<u>H</u>), 7.21 – 7.41 (m, 12H, Ar<u>H</u>), 6.96 (ddd, *J* = 7.7 Hz, $^{4}J_{H\text{-}P}$ = 4.3 Hz, $^{4}J_{H\text{-}H}$ = 1.3 Hz, 1H, Ar<u>H</u>), 4.89 (d, $^{4}J_{H\text{-}P}$ = 1.8 Hz, 2H, C<u>H</u>$_2$) ppm.

^{13}C NMR (101 MHz, $CDCl_3$): δ 142.0 (d, $^{1}J_{C\text{-}P}$ = 24.8 Hz), 136.5 (d, $^{2}J_{C\text{-}P}$ = 15.1 Hz), 136.3 (d, $^{2}J_{C\text{-}P}$ = 9.4 Hz), 134.3, 134.0 (d, $^{1}J_{C\text{-}P}$ = 19.8 Hz), 130.1 (d, $^{3}J_{C\text{-}P}$ = 4.4 Hz), 129.7, 129.0, 128.8, 128.7, 44.8 (d, $^{1}J_{C\text{-}P}$ = 28.7 Hz) ppm.

^{31}P NMR (162 MHz, $CDCl_3$): δ -16.93 ppm.

1-(2,6-Diisopropylphenyl)-3-(2-(diphenylphosphanyl)benzyl)-1*H*-imidazol-3-ium BAr^F S12[2]

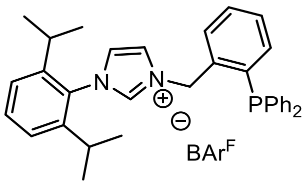

S12

To a 100 mL round-bottom flask equipped with a condenser was added 1-(2,6-di-*iso*-propylphenyl) imidazole **S6** (1.1 g, 4.82 mmol, 1.00 eq.), (2-(chloromethyl)phenyl)diphenylphosphane **S11** (1.5 g, 4.82 mmol, 1.00 eq.), and potassium iodide (0.400 g, 2.41 mmol, 0.50 eq.). The mixture was suspended in acetonitrile (36 mL) and subsequently heated to reflux for 2 h, after which time it was cooled to room temperature and passed through a silica plug, eluting with DCM. The filtrate was then concentrated *in vacuo*. The residue was redissolved in DCM (45 mL), NaBAr^F (4.27 g, 4.82 mmol, 1.00 eq.) was added, and the reaction mixture was stirred at room temperature overnight. The resulting suspension was concentrated *in vacuo* and the residue loaded onto silica and purified by column chromatography, eluting with 50% DCM/petroleum ether. 1-(2,6-Diisopropylphenyl)-3-(2-(diphenylphosphanyl)benzyl)-1*H*-imidazol-3-ium BAr^F **S12** (2.26 g, 1.65 mmol, 34%) was obtained as an off-white solid.

Melting point: 180 – 182 °C (Lit.: 170 - 172 °C).[2]

FTIR (neat): 2967, 1609, 1547, 1352, 1275, 1161, 887, 745, 681 cm^{-1}.

^{1}H NMR (400 MHz, CDCl$_3$): δ 8.15 (d, J = 1.5 Hz, 1H NC<u>H</u>N), 7.69 (bs, 8H, BAr^F Ar<u>H</u>), 7.57 (t, J = 7.9 Hz, 1H, Ar<u>H</u>), 7.50 (bs, 4H, BAr^F Ar<u>H</u>), 7.45 - 7.27 (m, 12H, Ar<u>H</u>), 7.23 (t, J = 1.8 Hz, 1H, Ar<u>H</u>), 7.20 - 7.12 (m, 4H, Ar<u>H</u>), 7.06 - 7.01 (m, 1H, Ar<u>H</u>), 5.44 (s, 2H, C<u>H</u>$_2$), 2.24 (sept, J = 6.8 Hz, 2H, 2 x C<u>H</u>), 1.13 - 1.05 (m, 12H, 4 × C<u>H</u>$_3$) ppm.
^{13}C NMR (101 MHz, CDCl$_3$): δ 161.8 (q, $^1J_{C\text{-}B}$ = 49.8 Hz), 145.2, 138.0 (d, $^2J_{C\text{-}P}$ = 16.3 Hz), 135.8 (d, $^3J_{C\text{-}P}$ = 4.5 Hz), 135.5, 135.0, 134.2 (d, $^1J_{C\text{-}P}$ = 23.4 Hz), 133.7 (d, $^2J_{C\text{-}P}$ = 19.7 Hz), 133.4 (d, $^3J_{C\text{-}P}$ = 6.1 Hz), 133.0, 131.7, 131.3 (d, $^3J_{C\text{-}P}$ = 4.4 Hz), 130.8, 130.3, 129.6 (d, $^2J_{C\text{-}P}$ = 7.4 Hz), 129.1 (q, $^2J_{C\text{-}F}$ = 32.8 Hz), 125.5, 125.3, 124.7 (q, $^1J_{C\text{-}F}$ = 272.5 Hz), 122.9, 117.6, 53.7 (d, $^3J_{C\text{-}P}$ = 17.8 Hz), 28.9, 24.2 ppm.
^{31}P NMR (162 MHz, CDCl$_3$): δ -16.36 ppm.
^{19}F NMR (376 MHz, CDCl$_3$): δ -62.33 ppm.
^{11}B NMR (128 MHz, CDCl$_3$): δ -6.58 ppm.

η^4-Cycloocta-1,5-diene(1-(2,6-diisopropylphenyl)-3-(2-(diphenylphosphanyl)benzyl) imidazole-2-ylidene)iridium BAr^F 2[2]

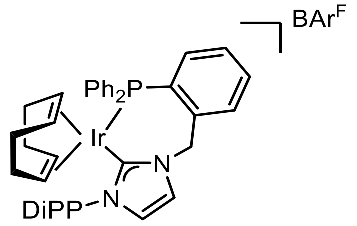

2

To a flame-dried Schlenk tube was added [Ir(COD)Cl]$_2$ (309 mg, 0.46 mmol, 0.5 eq.) and 1-(2,6-diisopropylphenyl)-3-(2-(diphenylphosphanyl)benzyl)-1*H*-imidazol-3-ium BAr^F **S12** (1.25 g, 0.91 mmol, 1.00 eq.). The mixture was then dissolved in THF (42 mL) and ^{t}BuOK (102 mg, 0.91 mmol, 1.00 eq.) was added. The reaction mixture was stirred at room temperature for 2 h, after which time the solvent was removed *in vacuo*. The resulting residue was purified by column chromatography, eluting with 50% DCM/petroleum ether, to give the complex **2** (1.26 g, 0.76 mmol, 83%) as a red solid.

Melting point: 186 – 188 °C (Lit.: 186 - 188 °C).[2]
FTIR (neat): 2970, 2027, 1608, 1437, 1352, 1273, 1117, 885, 839, 712, 681 cm^{-1}.

^{1}H NMR (400 MHz, CDCl$_3$): δ 7.92 - 7.84 (m, 2H, ArH), 7.71 (bs, 8H, BAr^F ArH), 7.56 - 7.43 (m, 10H, BAr^F ArH + ArH), 7.41 - 7.21 (m, 9H, ArH), 7.15 (d, *J* = 2.0 Hz, 1H, ArH), 6.97 (d, *J* = 2.0 Hz, 1H, ArH), 6.26 (d, $^2J_{H-H}$ = 14.5 Hz, 1H, CH_2), 5.50 (t, *J* = 7.2 Hz, 1H, COD CH), 4.59 (d, $^2J_{H-H}$ = 14.5 Hz, 1H, CH_2), 3.77 (t, *J* = 7.2 Hz, 1H, COD CH), 3.42 - 3.31 (m, 2H, COD CH), 3.17 (sept, *J* = 6.8 Hz, 1H, C$H(CH_3)_2$), 2.54 - 2.39 (m, 1H, COD CH_2), 2.24 (sept, *J* = 6.8 Hz, 1H, C$H(CH_3)_2$), 2.04 - 1.82 (m, 3H, COD CH_2), 1.78 - 1.64 (m, 1H, COD CH_2), 1.51 - 1.32 (m, 2H, COD CH_2), 1.25 (d, *J* = 6.7 Hz, 3H, CH_3), 1.21 (d, *J* = 6.7 Hz, 3H, CH_3), 1.16 (d, *J* = 6.8 Hz, 3H, CH_3), 1.13 - 1.04 (m, 1H, COD CH_2), 0.50 (d, *J* = 6.7 Hz, 3H, CH_3) ppm.

^{13}C NMR (101 MHz, CDCl$_3$): δ 171.1, 161.9 (d, $^1J_{C-B}$ = 50.1 Hz), 145.0 (d, $^1J_{C-P}$ = 112.6 Hz), 140.4 (d, $^3J_{C-P}$ = 12.2 Hz), 136.2, 135.0, 133.3, 133.1 (d, $^3J_{C-P}$ = 10.4 Hz), 132.6, 132.0, 131.6, 130.7, 130.6, 130.4 (d, $^3J_{C-P}$ = 7.2 Hz), 130.1, 129.9 (d, $^3J_{C-P}$ = 6.9 Hz), 129.43, 129.36 129.32, 129.26, 128.8, 128.7 (d, $^2J_{C-P}$ = 14.6 Hz), 125.3, 124.7 (q, $^1J_{C-F}$ = 272.6 Hz), 123.8, 120.4, 117.6, 87.4 (d, $^3J_{C-P}$ = 6.6 Hz), 86.9, 79.3 (d, $^2J_{C-P}$ = 17.3 Hz), 53.6 (d, $^3J_{C-P}$ = 9.8 Hz), 37.3, 34.7, 31.4, 28.5, 27.1, 26.6, 26.1, 23.8, 23.3, 22.2 ppm.

^{31}P NMR (162 MHz, CDCl$_3$): δ 3.93 ppm.

^{19}F NMR (376 MHz, CDCl$_3$): δ -62.42 ppm.

^{11}B NMR (128 MHz, CDCl$_3$): δ -6.58 ppm.

1.5 Catalyst Screening

1.5.1 Substrate synthesis

2-Phenyl-1,3,2-dioxaborolane 11[16]

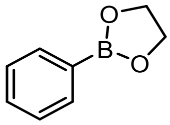

11

Prepared according to *General Procedure A*. Data presented as: (a) amount of phenylboronic acid, (b) volume of DCM, (c) amount of ethylene glycol, (d) amount of $MgSO_4$, and (e) yield.

(a) 1.00 g, 8.2 mmol, 1 eq., (b) 10 mL, (c) 560 mg, 9.0 mmol, 1.1 eq., (d) 985 mg, 8.2 mmol, 1 eq., and (e) 1.17 g, 7.93 mmol, 97%.

Appearance: colourless liquid.

FTIR (neat): 2978, 2906, 1600, 1498, 1438, 1394, 1332, 1211, 1091 cm^{-1}.

^{1}H NMR (400 MHz, $CDCl_3$): δ 7.82 (dd, J = 8.3 Hz, $^4J_{H\text{-}H}$ = 1.4 Hz, 2H, ArH), 7.49 (tt, J = 7.4 Hz, $^4J_{H\text{-}H}$ = 2.1 Hz, 1H, ArH), 7.39 (tt, J = 7.1 Hz, $^4J_{H\text{-}H}$ = 1.2 Hz, 2H, ArH), 4.38 (s, 4H, 2 × CH_2) ppm.

^{13}C NMR (101 MHz, $CDCl_3$): δ ^{13}C NMR (101 MHz, $CDCl_3$) δ 135.0, 131.6, 128.0, 66.2 ppm. *To note: the carbon directly attached to the boron atom was not observed, likely due to quadrupolar relaxation.*

^{11}B NMR (128 MHz, $CDCl_3$): δ 31.9 ppm.

4,4,5,5-Tetramethyl-2-phenyl-1,3,2-dioxaborolane 12[17,18]

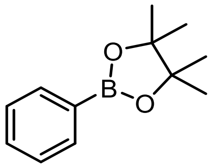

12

Prepared according to *General Procedure A*. Data presented as: (a) amount of phenylboronic acid, (b) volume of DCM, (c) amount of pinacol, (d) amount of $MgSO_4$, and (e) yield.

(a) 1.00 g, 8.2 mmol, 1 eq., (b) 10 mL, (c) 1.06 g, 9.0 mmol, 1.1 eq., (d) 985 mg, 8.2 mmol, 1 eq., and (e) 1.62 g, 7.94 mmol, 97%.

Melting point: 28 – 30 °C (Lit.: 29 – 30 °C).[18]
FTIR (neat): 2978, 2929, 1603, 1499, 1439, 1356, 1323, 1142, 1090 cm^{-1}.
^{1}H NMR (500 MHz, $CDCl_3$): δ 7.81 (d, *J* = 8.1 Hz, 2H, ArH), 7.45 (t, *J* = 7.5 Hz, 1H, ArH), 7.37 (t, *J* = 7.4 Hz, 2H, ArH), 1.35 (s, 12H, 4 × CH_3) ppm.
^{13}C NMR (126 MHz, $CDCl_3$): δ 134.9, 131.4, 127.8, 83.9, 25.0 ppm. *To note: the carbon directly attached to the boron atom was not observed, likely due to quadrupolar relaxation.*
^{11}B NMR (160 MHz, $CDCl_3$): δ 31.05 ppm.

1.5.2 Catalyst screening of phenylboronic acid **10** (Manuscript, Table 1)

Following *General Procedure B*, commercially available phenylboronic acid **10** (13.0 mg, 0.107 mmol, 1 eq.) was labelled using pre-catalysts **1** (9.3 mg, 0.00535 mmol, 5 mol%) and **7** (8.4 mg, 0.00535 mmol, 5 mol%) in MTBE (2 mL, 0.05 M). Each catalyst was applied over two runs and the deuterium incorporation was calculated based on analysis of the ^{1}H NMR spectra of the crude reaction mixtures.

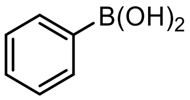

10

^{1}H NMR (400 MHz, CDCl3): δ 8.25 (d, *J* = 7.2 Hz, 2H, ArH), 7.61 (t, *J* = 7.2 Hz, 1H, ArH), 7.52 (t, *J* = 7.6 Hz, 2H, ArH) ppm.

Deuterium incorporation expected at δ 8.25 ppm and measured against the signal at δ 7.52 ppm.

Entry	Catalyst	Run 1 (%D)	Run 2 (%D)	Avg (%D)
**1**	**1**	<5	<5	<5
**2**	**7**	n.d.	n.d.	n.d.

n.d. denotes that no material was recovered.

1.5.3 Catalyst screening of 2-phenyl-1,3,2-dioxaborolane **11** (Manuscript, Table 1)

Following *General Procedure B*, prepared 2-phenyl-1,3,2-dioxaborolane **11** (15.8 mg, 0.107 mmol, 1 eq.) was labelled using pre-catalysts **1** (9.3 mg, 0.00535 mmol, 5 mol%), **7** (8.4 mg, 0.00535 mmol, 5 mol%), **8** (8.7 mg, 0.00535 mmol, 5 mol%), **9** (8.7 mg, 0.00535 mmol, 5 mol%), and **2** (8.9 mg, 0.00535 mmol, 5 mol%) in MTBE (2 mL, 0.05 M). Each catalyst was applied over two runs and the deuterium incorporation was calculated based on analysis of the ^{1}H NMR spectra of the crude reaction mixtures.

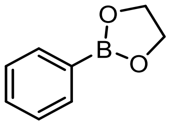

11

^{1}H NMR (400 MHz, CDCl$_3$): δ 7.82 (dd, *J* = 8.3 Hz, $^4J_{H\text{-}H}$ = 1.4 Hz, 2H, ArH), 7.49 (tt, *J* = 7.4 Hz, $^4J_{H\text{-}H}$ = 2.1 Hz, 1H, ArH), 7.39 (tt, *J* = 7.1 Hz, $^4J_{H\text{-}H}$ = 1.2 Hz, 2H, ArH), 4.38 (s, 4H, 2 × CH$_2$) ppm.

Deuterium incorporation expected at δ 7.82 ppm and measured against the signal at δ 7.39 ppm.

Entry	Catalyst	Run 1 (%)	Run 2 (%)	Avg (%)
**1**	**1**	<5	<5	<5
**2**	**7**	26	25	26
**3**	**8**	51.5	51.5	52
**4**	**9**	21	22.5	22
**5**	**2**	62.5	62	62

1.5.4 Catalyst screening of 4,4,5,5-tetramethyl-2-phenyl-1,3,2-dioxaborolane 12 (Manuscript, Table 1)

Following *General Procedure B*, prepared 4,4,5,5-tetramethyl-2-phenyl-1,3,2-dioxaborolane **12** (21.8 mg, 0.107 mmol, 1 eq.) was labelled using pre-catalyst **1** (9.3 mg, 0.00535 mmol, 5 mol%), **7** (8.4 mg, 0.00535 mmol, 5 mol%), **8** (8.7 mg, 0.00535 mmol, 5 mol%), **9** (8.7 mg, 0.00535 mmol, 5 mol%), and **2** (8.9 mg, 0.00535 mmol, 5 mol%) in MTBE (2 mL, 0.05 M). Each catalyst was applied over two runs and the deuterium incorporation was calculated based on analysis of the ^{1}H NMR spectra of pure products following silica gel chromatography.

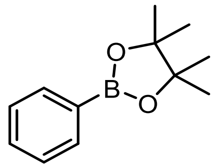

12

^{1}H NMR (500 MHz, CDCl$_3$): δ 7.81 (d, *J* = 8.1 Hz, 2H, ArH), 7.45 (t, *J* = 7.5 Hz, 1H, ArH), 7.37 (t, *J* = 7.4 Hz, 2H, ArH), 1.35 (s, 12H, 4 × CH$_3$) ppm.

Deuterium incorporation expected at δ 7.81 ppm and measured against the signal at 7.37 ppm.

Entry	Catalyst	Run 1 (%)	Run 2 (%)	Avg (%)
**1**	**1**	<5	<5	<5
**2**	**7**	7.5	7.5	8
**3**	**8**	18	18.5	18
**4**	**9**	5	5.5	5
**5**	**2**	24.5	27	26

1.6 Optimisation

1.6.1 Solvent and temperature screen (Manuscript, Table 2)

Following *General Procedure C*, prepared 4,4,5,5-tetramethyl-2-phenyl-1,3,2-dioxaborolane **12** (21.8 mg, 0.107 mmol, 1 eq.) was labelled using pre-catalyst **2** (8.9 mg, 0.00535 mmol, 5 mol%) in the indicated solvent (2 mL, 0.05 M) and temperature for 16 h. Each experiment was carried out over three runs and the deuterium incorporation was calculated based on analysis of the ^{1}H NMR spectra of pure products following silica gel chromatography.

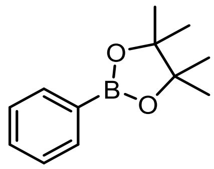

12

^{1}H NMR (500 MHz, CDCl$_3$): δ 7.81 (d, *J* = 8.1 Hz, 2H, ArH), 7.45 (t, *J* = 7.5 Hz, 1H, ArH), 7.37 (t, *J* = 7.4 Hz, 2H, ArH), 1.35 (s, 12H, 4 × CH$_3$) ppm.

Deuterium incorporation expected at δ 7.81 ppm and measured against the signal at 7.37 ppm.

Entry	Solvent	Temp. (°C)	Run 1 (%)	Run 2 (%)	Run 3 (%)	Avg (%)
**2**	Toluene	50	45	47	45.5	46
**3**	1,2-DCE	50	12	12.5	10.5	12
**4**	PhF	50	79	77	70.5	76
**5**	Toluene	80	81	79.5	82	81
**6**	1,2-DCE	80	28	27	23.5	26
**7**	PhF	80	92	92.5	92.5	92

1.6.2 Concentration screen (Manuscript, Table 2)

Following *General Procedure C*, prepared 4,4,5,5-tetramethyl-2-phenyl-1,3,2-dioxaborolane **12** (21.8 mg, 0.107 mmol 1 eq.) was labelled using pre-catalyst **2** (8.9 mg, 0.00535 mmol, 5 mol%) in toluene (varying concentration) at 80 °C for 16 h. Each experiment was carried out over three runs and the deuterium incorporation was calculated based on analysis of the ^{1}H NMR spectra of pure products following silica gel chromatography.

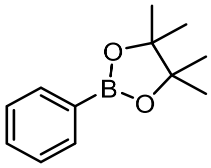

12

^{1}H NMR (500 MHz, $CDCl_3$): δ 7.81 (d, *J* = 8.1 Hz, 2H, ArH), 7.45 (t, *J* = 7.5 Hz, 1H, ArH), 7.37 (t, *J* = 7.4 Hz, 2H, ArH), 1.35 (s, 12H, 4 × CH_3) ppm.

Deuterium incorporation expected at δ 7.81 ppm and measured against the signal at 7.37 ppm.

Entry	Conc. (M)	Solvent Volume (mL)	Run 1 (%)	Run 2 (%)	Run 3 (%)	Avg (%)
**8**	0.1	1	91.5	88.5	92	91
**9**	0.035	3	79.5	76.5	74	77
**10**	0.02	5	51.5	57.5	58	56

1.6.3 Time study

Following *General Procedure C*, prepared 4,4,5,5-tetramethyl-2-phenyl-1,3,2-dioxaborolane **12** (21.8 mg, 0.107 mmol, 1 eq.) was labelled using pre-catalyst **2** (8.9 mg, 0.00535 mmol, 5 mol%) in the toluene (1 mL, 0.1 M) at 80 °C for the indicated time. Each experiment was carried out

over two runs and the deuterium incorporation was calculated based on analysis of the ^{1}H NMR spectra of pure products following silica gel chromatography.

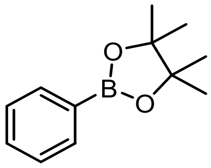

12

^{1}H NMR (500 MHz, CDCl$_3$): δ 7.81 (d, *J* = 8.1 Hz, 2H, ArH), 7.45 (t, *J* = 7.5 Hz, 1H, ArH), 7.37 (t, *J* = 7.4 Hz, 2H, ArH), 1.35 (s, 12H, 4 × CH$_3$) ppm.

Deuterium incorporation expected at δ 7.81 ppm and measured against the signal at 7.37 ppm.

Entry	Time (h)	Run 1 (%)	Run 2 (%)	Avg (%)
**1**	1	88.5	87.5	88
**2**	2	92	92	92
**3**	4	92	92.5	92
**4**	6	93	92.5	93
**5**	8	92.5	92.5	93
**6**	24	90.5	90	90

The data from this table was used to generate **Chart 1** below.

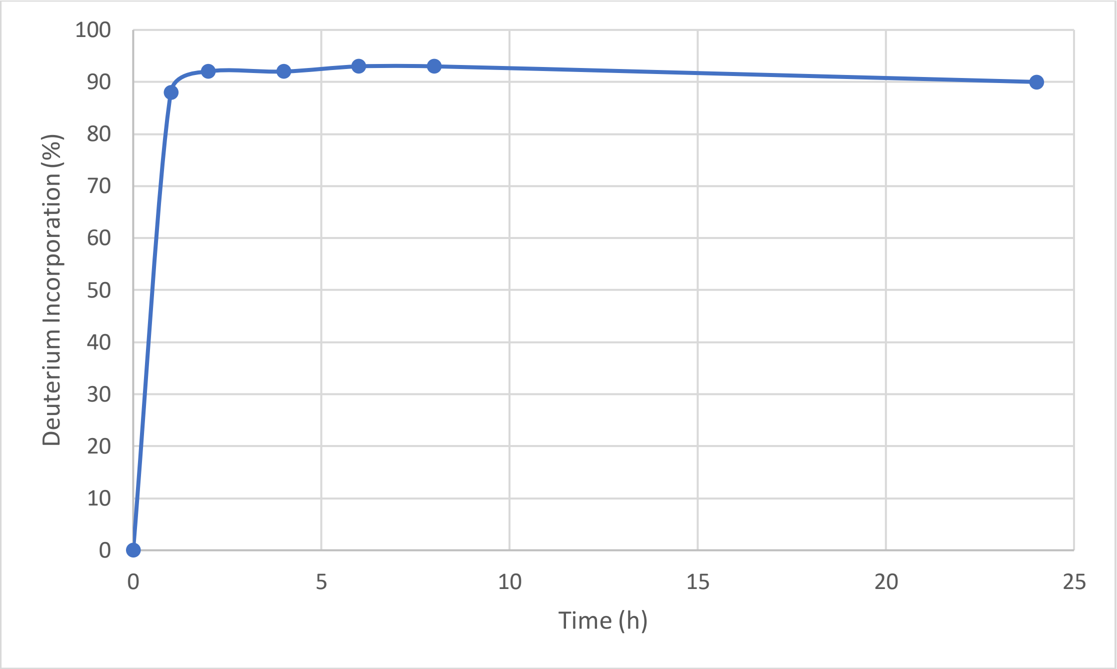

Chart 1

1.6.4 Catalyst loading investigation

Following *General Procedure C*, prepared 4,4,5,5-tetramethyl-2-phenyl-1,3,2-dioxaborolane **12** (21.8 mg, 0.107 mmol, 1 eq.) was labelled using pre-catalyst **2** at the indicated loading in toluene (1 mL, 0.1 M) at 80 °C for 4 h. Each experiment was carried out over three runs and the deuterium incorporation was calculated based on analysis of the ^{1}H NMR spectra of pure products following silica gel chromatography.

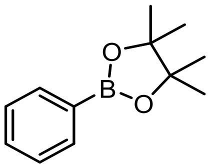

12

^{1}H NMR (500 MHz, CDCl$_3$): δ 7.81 (d, *J* = 8.1 Hz, 2H, ArH), 7.45 (t, *J* = 7.5 Hz, 1H, ArH), 7.37 (t, *J* = 7.4 Hz, 2H, ArH), 1.35 (s, 12H, 4 × CH$_3$) ppm.

Deuterium incorporation expected at δ 7.81 ppm and measured against the signal at 7.37 ppm.

Entry	Catalyst Loading (mol%)	Catalyst Mass (mg)	Run 1 (%)	Run 2 (%)	Run 3 (%)	Avg (%)
**1**	1	1.8	69.5	72	71.5	71
**2**	2	3.6	85.5	84.5	84	85
**3**	3	5.3	90	91	88.5	90
**4**	7.5	13.4	92	92	91.5	92

1.7 Substrate Scope – Aryl Boronic Pinacol Esters (Aryl-BPins)

1.7.1 Substrate synthesis

4-(4,4,5,5-Tetramethyl-1,3,2-dioxaborolan-2-yl)phenol 13a[19–21]

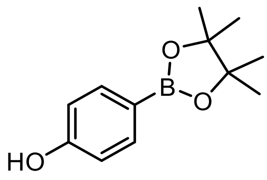

13a

Prepared according to *General Procedure A*. Data presented as: (a) amount of 4-hydroxybenzeneboronic acid, (b) volume of DCM, (c) amount of pinacol, (d) amount of $MgSO_4$, and (e) yield.

(a) 276 mg, 2.0 mmol, 1 eq., (b) 2.5 mL, (c) 260 mg, 2.2 mmol, 1.1 eq., (d) 241 mg, 2.0 mmol, 1 eq., and (e) 338 mg, 1.54 mmol, 77%.

Melting point: 114-116 °C (Lit.: 110-111 °C).[21]
FTIR (neat): 3306, 2984, 1607, 1580, 1429, 1356, 1308, 1261, 1138, 1078 cm^{-1}.
^{1}H NMR (400 MHz, $CDCl_3$): δ 7.71 (d, *J* = 8.6 Hz, 2H, ArH), 6.82 (d, *J* = 8.5 Hz, 2H, ArH), 1.33 (s, 12H, 4 × CH_3) ppm.
^{13}C NMR (101 MHz, $CDCl_3$): δ 158.4, 136.9, 114.9, 83.8, 25.0 ppm. *To note: the carbon directly attached to the boron atom was not observed, likely due to quadrupolar relaxation.*
^{11}B NMR (128 MHz, $CDCl_3$): δ 31.01 ppm.

2-(4-Methoxyphenyl)-4,4,5,5-tetramethyl-1,3,2-dioxaborolane 13b[22–23]

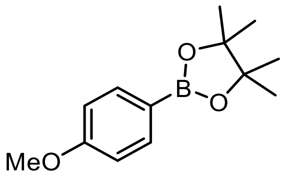

13b

Prepared according to *General Procedure A*. Data presented as: (a) amount of 4-methoxybenzeneboronic acid, (b) volume of DCM, (c) amount of pinacol, (d) amount of $MgSO_4$, and (e) yield.

(a) 1.5 g, 9.9 mmol, 1 eq., (b) 15 mL, (c) 1.29 g, 10.9 mmol, 1.1 eq., (d) 1.19 g, 9.9 mmol, 1 eq., and (e) 2.17 g, 9.28 mmol, 94%.

Melting point: obtained as a liquid (Lit.: 33.6 – 35.8 °C).[23]
FTIR (neat): 2976, 2934, 2837, 1603, 1396, 1356, 1277, 1244, 1140, 1090, 1028, 858, 654 cm^{-1}.
^{1}H NMR (400 MHz, $CDCl_3$): δ 7.75 (d, *J* = 8.7 Hz, 2H, Ar$\underline{H}$), 6.90 (d, *J* = 8.7 Hz, 2H, Ar$\underline{H}$), 3.83 (s, 3H, OC$\underline{H}_3$), 1.33 (s, 12H, 4 × C$\underline{H}_3$) ppm.
^{13}C NMR (101 MHz, $CDCl_3$): δ 162.3, 136.7, 113.5, 83.7, 55.2, 25.0 ppm. *To note: the carbon directly attached to the boron atom was not observed, likely due to quadrupolar relaxation.*
^{11}B NMR (128 MHz, $CDCl_3$): δ 31.09 ppm.

4,4,5,5-Tetramethyl-2-(*p*-tolyl)-1,3,2-dioxaborolane 13c[17,18,24]

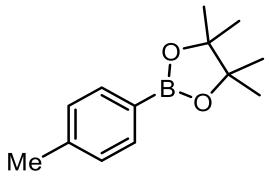

13c

Prepared according to *General Procedure A*. Data presented as: (a) amount of 4-methylbenzeneboronic acid, (b) volume of DCM, (c) amount of pinacol, (d) amount of $MgSO_4$, and (e) yield.

(a) 272 mg, 2.0 mmol, 1 eq., (b) 2.5 mL, (c) 260 mg, 2.2 mmol, 1.1 eq., (d) 241 mg, 2.0 mmol, 1 eq., and (e) 367 mg, 1.68 mmol, 84%.

Melting point: 49-51 °C (Lit.: 48-49 °C).[24]
FTIR (neat): 2976, 2924, 2357, 1611, 1516, 1396, 1358, 1142, 1088 cm^{-1}.
^{1}H NMR (400 MHz, $CDCl_3$): δ 7.70 (d, *J* = 7.9 Hz, 2H, Ar<u>H</u>), 7.19 (d, *J* = 7.5 Hz, 2H, Ar<u>H</u>), 1.55 (s, 3H, ArC<u>H</u>$_3$), 1.34 (s, 12H, 4 × C<u>H</u>$_3$) ppm.
^{13}C NMR (101 MHz, $CDCl_3$): δ 140.9, 134.3, 128.0, 83.1, 24.4, 21.2 ppm. *To note: the carbon directly attached to the boron atom was not observed, likely due to quadrupolar relaxation.*
^{11}B NMR (128 MHz, $CDCl_3$): δ 31.21 ppm.

2-(4-(*Tert*-butyl)phenyl)-4,4,5,5-tetramethyl-1,3,2-dioxaborolane 13d[25]

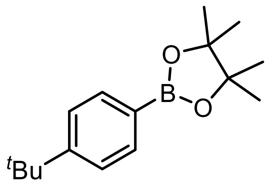

13d

Prepared according to *General Procedure A*. Data presented as: (a) amount of 4-*tert*-butylbenzeneboronic acid, (b) volume of DCM, (c) amount of pinacol, (d) amount of $MgSO_4$, and (e) yield.

(a) 356 mg, 2.0 mmol, 1 eq., (b) 2.5 mL, (c) 260 mg, 2.2 mmol, 1.1 eq., (d) 241 mg, 2.0 mmol, 1 eq., and (e) 509 mg, 1.96 mmol, 98%.

Melting point: 140-142 °C (lit. 138 – 139 °C).[25]
FTIR (neat): 2967, 2868, 1611, 1462, 1393, 1360, 1321, 1144, 1117 cm^{-1}.
^{1}H NMR (400 MHz, $CDCl_3$): δ 7.77 (d, *J* = 8.3 Hz, 2H, ArH), 7.41 (d, *J* = 8.3 Hz, 2H, ArH), 1.34 (s, 12H, 4 × CH_3), 1.33 (s, 9H, 3 × CH_3 (^{t}Bu)) ppm.
^{13}C NMR (101 MHz, $CDCl_3$): δ 154.6, 134.8, 124.8, 83.7, 35.0, 31.4, 25.0 ppm. *To note: the carbon directly attached to the boron atom was not observed, likely due to quadrupolar relaxation.*
^{11}B NMR (128 MHz, $CDCl_3$): δ 31.16 ppm.

2-([1,1'-Biphenyl]-4-yl)-4,4,5,5-tetramethyl-1,3,2-dioxaborolane 13e[17,18,26]

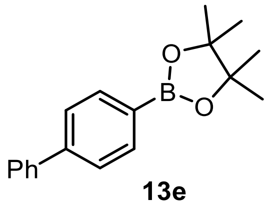

13e

Prepared according to *General Procedure A*. Data presented as: (a) amount of 4-biphenylboronic acid, (b) volume of DCM, (c) amount of pinacol, (d) amount of $MgSO_4$, and (e) yield.

(a) 396 mg, 2.0 mmol, 1 eq., (b) 2.5 mL, (c) 260 mg, 2.2 mmol, 1.1 eq., (d) 241 mg, 2.0 mmol, 1 eq., and (e) 498 mg, 1.78 mmol, 89%.

Melting point: 112 – 114 °C (Lit.: 111 – 112 °C).[26]
FTIR (neat): 3080, 3032, 2974, 2930, 2361, 1608, 1551, 1522, 1396, 1358, 1323, 1140, 1092 cm^{-1}.
^{1}H NMR (400 MHz, $CDCl_3$): δ 7.89 (d, *J* = 8.3 Hz, 2H, ArH), 7.65 - 7.59 (m, 4H, ArH + ArH (Ph)), 7.45 (t, *J* = 7.4 Hz, 2H, ArH (Ph)), 7.36 (t, *J* = 7.3 Hz, 1H, ArH (Ph)), 1.37 (s, 12H, 4 × CH_3) ppm.
^{13}C NMR (101 MHz, $CDCl_3$): δ 144.0, 141.2, 135.4, 128.9, 127.7, 127.4, 126.6, 84.0, 25.0 ppm. *To note: the carbon directly attached to the boron atom was not observed, likely due to quadrupolar relaxation.*
^{11}B NMR (128 MHz, $CDCl_3$): δ 31.41 ppm.

2-(4-Chlorophenyl)-4,4,5,5-tetramethyl-1,3,2-dioxaborolane 13f[17,21,27]

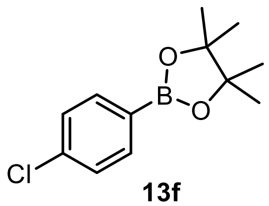

13f

Prepared according to *General Procedure A*. Data presented as: (a) amount of 4-chlorobenzeneboronic acid, (b) volume of DCM, (c) amount of pinacol, (d) amount of $MgSO_4$, and (e) yield.

(a) 313 mg, 2.0 mmol, 1 eq., (b) 2.5 mL, (c) 260 mg, 2.2 mmol, 1.1 eq., (d) 241 mg, 2.0 mmol, 1 eq., and (e) 362 mg, 1.52 mmol, 76%.

Melting point: 49 – 51 °C (Lit.: 50 – 51 °C).[21]
FTIR (neat): 2976, 2936, 1595, 1560, 1470, 1391, 1358, 1140, 1092 cm^{-1}.
^{1}H NMR (400 MHz, $CDCl_3$): δ 7.73 (d, *J* = 8.3 Hz, 2H, ArH) 7.34 (d, *J* = 8.4 Hz, 2H, ArH), 1.34 (s, 12H, 4 × CH_3) ppm.

^{13}C NMR (101 MHz, CDCl$_3$): δ 137.7, 136.3, 128.2, 84.2, 25.0, ppm. *To note: the carbon directly attached to the boron atom was not observed, likely due to quadrupolar relaxation.*
^{11}B NMR (128 MHz, CDCl$_3$): δ 30.79 ppm.

2-(4-Fluorophenyl)-4,4,5,5-tetramethyl-1,3,2-dioxaborolane 13g[18,28]

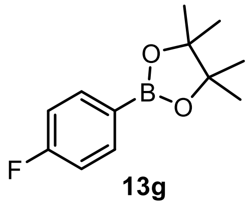

13g

Prepared according to *General Procedure A*. Data presented as: (a) amount of 4-fluorobenzeneboronic acid, (b) volume of DCM, (c) amount of pinacol, (d) amount of $MgSO_4$, and (e) yield.

(a) 280 mg, 2.0 mmol, 1 eq., (b) 2.5 mL, (c) 260 mg, 2.2 mmol, 1.1 eq., (d) 241 mg, 2.0 mmol, 1 eq., and (e) 355 mg, 1.60 mmol, 80%.

Melting point: obtained as a liquid.
FTIR (neat): 2978, 2932, 2361, 1603, 1541, 1398, 1356, 1142, 1086 cm^{-1}.
^{1}H NMR (400 MHz, CDCl$_3$): δ 7.80 (dd, J = 8.7 Hz, $^{4}J_{H\text{-}F}$ = 6.2 Hz, 2H, Ar<u>H</u>), 7.05 (dd, J = 8.7 Hz, $^{3}J_{H\text{-}F}$ = 9.2 Hz, 2H, Ar<u>H</u>), 1.34 (s, 12H, 4 × C<u>H</u>$_3$) ppm.
^{13}C NMR (101 MHz, CDCl$_3$): δ 165.3 (d, $^{1}J_{C\text{-}F}$ = 250.5 Hz), 137.1 (d, $^{3}J_{C\text{-}F}$ = 8.1 Hz), 115.0 (d, $^{2}J_{C\text{-}F}$ = 20.1 Hz), 84.1, 25.0 ppm. *To note: the carbon directly attached to the boron atom was not observed, likely due to quadrupolar relaxation.*
^{11}B NMR (128 MHz, CDCl$_3$): δ 30.78 ppm.
^{19}F NMR (376 MHz, CDCl$_3$): δ -108.5 ppm.

2-(4,4,5,5-Tetramethyl-1,3,2-dioxaborolan-2-yl)phenol 13j[27,29]

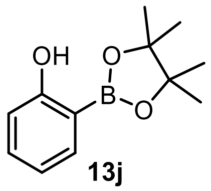

13j

Prepared according to *General Procedure A*. Data presented as: (a) amount of 2-hydroxybenzeneboronic acid, (b) volume of DCM, (c) amount of pinacol, (d) amount of $MgSO_4$, and (e) yield.

(a) 276 mg, 2.0 mmol, 1 eq., (b) 2.5 mL, (c) 260 mg, 2.2 mmol, 1.1 eq., (d) 241 mg, 2.0 mmol, 1 eq., and (e) 365 mg, 1.66 mmol, 83%.

Melting point: obtained as a liquid.
FTIR (neat): 3445, 2978, 2930, 1618, 1574, 1485, 1460, 1389, 1356, 1213, 1138, 1069 cm^{-1}.
^{1}H NMR (400 MHz, $CDCl_3$): δ 7.80 (s, 1H, ArOH), 7.61 (dd, *J* = 7.6 Hz, $^4J_{H\text{-}H}$ = 1.8 Hz, 1H, ArH), 7.37 (td, *J* = 7.6 Hz, $^4J_{H\text{-}H}$ = 1.7 Hz, 1H, ArH), 6.93 - 6.84 (m, 2H, ArH), 1.37 (s, 12H, 4 × CH_3) ppm.
^{13}C NMR (101 MHz, $CDCl_3$): δ 163.8, 135.9, 134.0, 119.7, 115.6, 84.6, 25.0 ppm. *To note: the carbon directly attached to the boron atom was not observed, likely due to quadrupolar relaxation.*
^{11}B NMR (128 MHz, $CDCl_3$): δ 30.87 ppm.

2-(2-Methoxyphenyl)-4,4,5,5-tetramethyl-1,3,2-dioxaborolane 13k[17,27,30]

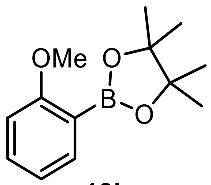

13k

Prepared according to *General Procedure A*. Data presented as: (a) amount of 2-methoxybenzeneboronic acid, (b) volume of DCM, (c) amount of pinacol, (d) amount of $MgSO_4$, and (e) yield.

(a) 304 mg, 2.0 mmol, 1 eq., (b) 2.5 mL, (c) 260 mg, 2.2 mmol, 1.1 eq., (d) 241 mg, 2.0 mmol, 1 eq., and (e) 400 mg, 1.71 mmol, 86%.

Melting point: 83 – 85 °C (Lit.: 76 – 77 °C).[30]
FTIR (neat): 2974, 2932, 1597, 1574, 1489, 1429, 1350, 1246, 1142, 1069 3345, cm^{-1}.
^{1}H NMR (400 MHz, $CDCl_3$): δ 7.67 (dd, J = 7.3 Hz, $^4J_{H\text{-}H}$ =1.9 Hz, 1H, ArH), 7.39 (ddd, J = 8.3, 7.3 Hz, $^4J_{H\text{-}H}$ = 1.9 Hz, 1H, ArH), 6.94 (td, J = 7.3 Hz, $^4J_{H\text{-}H}$ = 0.9 Hz, 1H, ArH), 6.85 (d, J = 8.2 Hz, 1H, ArH), 3.83 (s, 3H, OCH_3), 1.36 (s, 12H, 4 × CH_3) ppm.
^{13}C NMR (101 MHz, $CDCl_3$): δ 164.3, 136.8, 132.6, 120.3, 110.6, 83.6, 56.0, 25.0 ppm. *To note: the carbon directly attached to the boron atom was not observed, likely due to quadrupolar relaxation.*
^{11}B NMR (128 MHz, $CDCl_3$): δ 31.17 ppm.

4,4,5,5-Tetramethyl-2-(*o*-tolyl)-1,3,2-dioxaborolane 13l[17,27]

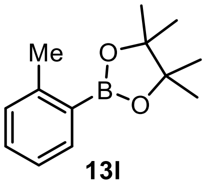

13l

Prepared according to *General Procedure A*. Data presented as: (a) amount of 2-methylbenzeneboronic acid, (b) volume of DCM, (c) amount of pinacol, (d) amount of $MgSO_4$, and (e) yield.

(a) 272 mg, 2.0 mmol, 1 eq., (b) 2.5 mL, (c) 260 mg, 2.2 mmol, 1.1 eq., (d) 241 mg, 2.0 mmol, 1 eq., and (e) 414 mg, 1.90 mmol, 95%.

Melting point: obtained as a liquid (Lit.: 32 – 34 °C).[27]
FTIR (neat): 2976, 2928, 1601, 1570, 1489, 1437, 1379, 1344, 1310, 1144, 1072 cm^{-1}.
^{1}H NMR (400 MHz, $CDCl_3$): δ 7.76 (dd, *J* = 7.8 Hz, $^{4}J_{H-H}$ = 1.6 Hz, 1H, ArH), 7.31 (td, *J* = 7.5 Hz, $^{4}J_{H-H}$ = 1.5 Hz, 1H, ArH), 7.19 - 7.13 (m, 2H, ArH), 2.54 (s, 3H, $ArCH_3$), 1.35 (s, 12H, 4 × CH_3) ppm.
^{13}C NMR (101 MHz, $CDCl_3$): δ 144.3, 135.4, 130.3, 129.3, 124.2, 82.9, 24.4, 21.7 ppm. *To note: the carbon directly attached to the boron atom was not observed, likely due to quadrupolar relaxation.*
^{11}B NMR (128 MHz, $CDCl_3$): δ 31.47 ppm.

3-(4,4,5,5-Tetramethyl-1,3,2-dioxaborolan-2-yl)phenol 13m[31,32]

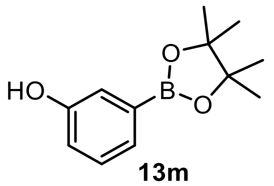

13m

Prepared according to *General Procedure A*. Data presented as: (a) amount of 3-hydroxybenzeneboronic acid, (b) volume of DCM, (c) amount of pinacol, (d) amount of $MgSO_4$, and (e) yield.

(a) 276 mg, 2.0 mmol, 1 eq., (b) 2.5 mL, (c) 260 mg, 2.2 mmol, 1.1 eq., (d) 241 mg, 2.0 mmol, 1 eq., and (e) 366 mg, 1.66 mmol, 83%.

Melting point: 86 – 88 °C.
FTIR (neat): 3397, 2974, 2932, 1585, 1431, 1356, 1138, 1086 cm^{-1}.
^{1}H NMR (400 MHz, $CDCl_3$): δ 7.36 (dt, *J* = 7.3 Hz, $^{4}J_{H\text{-}H}$ = 1.0 Hz, 1H, ArH), 7.25 - 7.21 (m, 2H, ArH), 6.93 (ddd, *J* = 8.1 Hz, $^{4}J_{H\text{-}H(OH)}$ = 2.8 Hz, $^{4}J_{H\text{-}H}$ = 1.1 Hz, 1H, ArH), 4.83 (s, 1H, ArOH), 1.32 (s, 12H, 4 × CH_3) ppm.
^{13}C NMR (101 MHz, $CDCl_3$): δ 155.2, 129.4, 127.3, 121.2, 118.5, 84.1, 25.0 ppm. *To note: the carbon directly attached to the boron atom was not observed, likely due to quadrupolar relaxation.*
^{11}B NMR (128 MHz, $CDCl_3$): δ 30.92 ppm.

2-(3-Methoxyphenyl)-4,4,5,5-tetramethyl-1,3,2-dioxaborolane 13n[17,18]

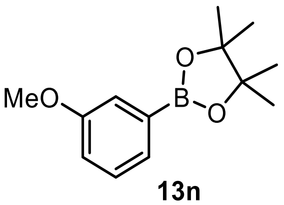

13n

Prepared according to *General Procedure A*. Data presented as: (a) amount of 3-methoxybenzeneboronic acid, (b) volume of DCM, (c) amount of pinacol, (d) amount of $MgSO_4$, and (e) yield.

(a) 304 mg, 2.0 mmol, 1 eq., (b) 2.5 mL, (c) 260 mg, 2.2 mmol, 1.1 eq., (d) 241 mg, 2.0 mmol, 1 eq., and (e) 454 mg, 1.94 mmol, 97%.

Melting point: obtained as a liquid.

FTIR (neat): 2976, 2934, 1599, 1576, 1449, 1420, 1350, 1238, 1142, 1043 cm^{-1}.

^{1}H NMR (400 MHz, $CDCl_3$): δ 7.40 (dt, *J* = 7.2 Hz, $^{4}J_{H-H}$ = 1.1 Hz, 1H, Ar<u>H</u>), 7.33 (d, $^{4}J_{H-H}$ = 2.7 Hz, 1H, Ar<u>H</u>), 7.29 (t, *J* = 7.3 Hz, 1H, Ar<u>H</u>), 7.01 (ddd, *J* = 8.2 Hz, $^{4}J_{H-H}$ = 2.7 Hz, $^{4}J_{H-H}$ = 1.2 Hz, 1H, Ar<u>H</u>), 3.83 (s, 3H, OC<u>H</u>$_3$), 1.35 (s, 12H, 4 × C<u>H</u>$_3$) ppm.

^{13}C NMR (101 MHz, $CDCl_3$): δ 159.2, 129.1, 127.3, 118.9, 118.1, 84.0, 55.4, 25.0 ppm. *To note: the carbon directly attached to the boron atom was not observed, likely due to quadrupolar relaxation.*

^{11}B NMR (128 MHz, $CDCl_3$): δ 31.21 ppm.

4,4,5,5-Tetramethyl-2-(*m*-tolyl)-1,3,2-dioxaborolane 13o[17,27]

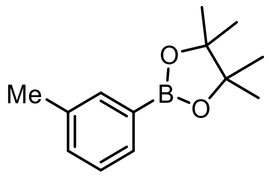

13o

Prepared according to *General Procedure A*. Data presented as: (a) amount of 3-methylbenzeneboronic acid, (b) volume of DCM, (c) amount of pinacol, (d) amount of $MgSO_4$, and (e) yield.

(a) 272 mg, 2.0 mmol, 1 eq., (b) 2.5 mL, (c) 260 mg, 2.2 mmol, 1.1 eq., (d) 241 mg, 2.0 mmol, 1 eq., and (e) 412 mg, 1.89 mmol, 95%.

Melting point: 32 – 34 °C (Lit.: 33 – 34 °C).[27]

FTIR (neat): 2978, 2928, 1607, 1584, 1414, 1354, 1314, 1207, 1144, 1078 cm^{-1}.

^{1}H NMR (400 MHz, $CDCl_3$): δ 7.64 (s, 1H, ArH), 7.63 - 7.59 (m, 1H, ArH), 7.28 - 7.26 (m, 2H, ArH), 2.36 (s, 3H, ArCH_3), 1.35 (s, 12H, 4 × CH_3) ppm.

^{13}C NMR (101 MHz, $CDCl_3$): δ 137.3, 135.5, 132.2, 131.9, 127.8, 83.9, 25.0, 21.4 ppm. *To note: the carbon directly attached to the boron atom was not observed, likely due to quadrupolar relaxation.*

^{11}B NMR (128 MHz, $CDCl_3$): δ 31.22 ppm.

2-(3,5-Dimethylphenyl)-4,4,5,5-tetramethyl-1,3,2-dioxaborolane 13p[33,34]

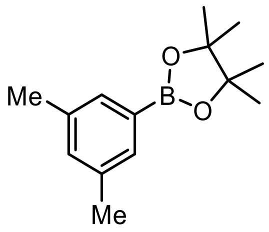

13p

Prepared according to *General Procedure A*. Data presented as: (a) amount of 3,5-dimethylbenzeneboronic acid, (b) volume of DCM, (c) amount of pinacol, (d) amount of $MgSO_4$, and (e) yield.

(a) 300 mg, 2.0 mmol, 1 eq., (b) 2.5 mL, (c) 260 mg, 2.2 mmol, 1.1 eq., (d) 241 mg, 2.0 mmol, 1 eq., and (e) 464 mg, 1.99 mmol, 99%.

Melting point: 96 – 98 °C (Lit.: 91 – 93 °C).[33]
FTIR (neat): 2976, 2922, 1599, 1420, 1385, 1356, 1240, 1138, 1115 cm^{-1}.
^{1}H NMR (400 MHz, $CDCl_3$): δ 7.44 (s, 2H, Ar$\underline{H}$), 7.10 (s, 1H, Ar$\underline{H}$), 2.32 (s, 6H, 2 × ArC$\underline{H}_3$), 1.35 (s, 12H, 4 × C$\underline{H}_3$) ppm.
^{13}C NMR (101 MHz, $CDCl_3$): δ 136.7, 132.5, 131.9, 83.2, 24.4, 20.6 ppm. *To note: the carbon directly attached to the boron atom was not observed, likely due to quadrupolar relaxation.*
^{11}B NMR (128 MHz, $CDCl_3$): δ 31.23 ppm.

2-(3-Fluoro-4-methoxyphenyl)-4,4,5,5-tetramethyl-1,3,2-dioxaborolane 13q[34]

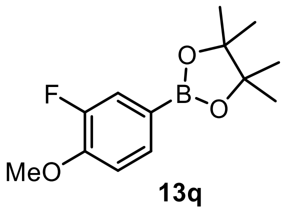

13q

Prepared according to *General Procedure A*. Data presented as: (a) amount of (3-fluoro-4-methoxyphenyl)boronic acid, (b) volume of DCM, (c) amount of pinacol, (d) amount of $MgSO_4$, and (e) yield.

(a) 340 mg, 2.0 mmol, 1 eq., (b) 2.5 mL, (c) 260 mg, 2.2 mmol, 1.1 eq., (d) 241 mg, 2.0 mmol, 1 eq., and (e) 268 mg, 1.06 mmol, 53%.

Melting point: 86 – 88 °C (Lit.: 125 – 128 °C).[34]

FTIR (neat): 2976, 2849, 2361, 1614, 1524, 1422, 1354, 1269, 1130, 1080, 1026 cm^{-1}.

^{1}H NMR (400 MHz, $CDCl_3$): δ 7.53 (dt, J = 8.2 Hz, $^{4}J_{H\text{-}H}$ = 1.0 Hz, 1H, Ar-$\underline{H}$), 7.49 (dd, $^{3}J_{H\text{-}F}$ = 11.7 Hz, $^{4}J_{H\text{-}H}$ = 1.4 Hz, 1H, Ar-$\underline{H}$), 6.95 (t, J = 8.1 Hz, 1H, Ar-$\underline{H}$), 3.91 (s, 3H, -OC$\underline{H}_3$), 1.33 (s, 12H, 4 × C$\underline{H}_3$) ppm.

^{13}C NMR (101 MHz, $CDCl_3$): δ 152.2 (d, $^{1}J_{C\text{-}F}$ = 245.9 Hz), 150.4 (d, $^{2}J_{C\text{-}F}$ = 10.5 Hz), 131.6 (d, $^{3}J_{C\text{-}F}$ = 3.8 Hz), 121.9 (d, $^{2}J_{C\text{-}F}$ = 16.3 Hz), 112.7, 84.0, 56.2, 25.0 ppm. *To note: the carbon directly attached to the boron atom was not observed, likely due to quadrupolar relaxation.*

^{11}B NMR (128 MHz, $CDCl_3$): δ 30.68 ppm.

^{19}F NMR (376 MHz, $CDCl_3$): δ -137.10 (dd, $^{3}J_{H\text{-}F}$ = 11.8 Hz, $^{4}J_{H\text{-}F}$ = 8.2 Hz) ppm.

HRMS *m/z* (ESI): Calc. for $C_{13}H_{18}BFO_3$ [M^+]: 252.13330. Found 252.1323.

1-Methyl-5-(4,4,5,5-tetramethyl-1,3,2-dioxaborolan-2-yl)-1*H*-indole 13r[35-37]

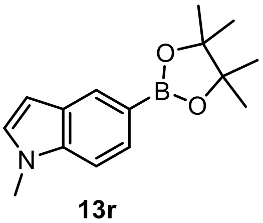

13r

Prepared according to *General Procedure A*. Data presented as: (a) amount of (1-methyl-1*H*-indol-5-yl)boronic acid, (b) volume of DCM, (c) amount of pinacol, (d) amount of $MgSO_4$, and (e) yield.

(a) 350 mg, 2.0 mmol, 1 eq., (b) 2.5 mL, (c) 260 mg, 2.2 mmol, 1.1 eq., (d) 241 mg, 2.0 mmol, 1 eq., and (e) 501 mg, 1.95 mmol, 98%.

Melting point: 108 – 110 °C (Lit.: 107 – 109 °C).[36]

FTIR (neat): 2918, 2829, 1608, 1516, 1540, 1369, 1350, 1136, 1089, 1070, 854 cm^{-1}.

^{1}H NMR (400 MHz, $CDCl_3$): δ 8.17 (s, 1H, ArH), 7.68 (dd, *J* = 8.3 Hz, $^4J_{H\text{-}H}$ = 1.0 Hz, 1H, ArH), 7.33 (d, *J* = 8.3 Hz, 1H, ArH), 7.04 (d, *J* = 3.1 Hz, 1H, ArH), 6.51 (dd, *J* = 3.1 Hz, $^4J_{H\text{-}H}$ = 0.9 Hz, 1H, ArH), 3.79 (s, 3H, NC$\underline{H}_3$), 1.38 (s, 12H, 4 x C$\underline{H}_3$) ppm.

^{13}C NMR (101 MHz, $CDCl_3$): δ 138.8, 129.02, 128.95, 128.4, 127.8, 108.7, 101.8, 83.5, 32.9, 25.0 ppm. *To note: the carbon directly attached to the boron atom was not observed, likely due to quadrupolar relaxation.*

^{11}B NMR (128 MHz, $CDCl_3$): δ 31.74 ppm.

2-(4-Iodophenyl)-4,4,5,5-tetramethyl-1,3,2-dioxaborolane 13s[27,38,39]

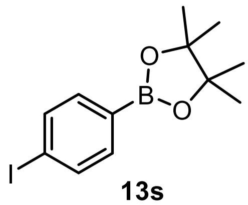

13s

Prepared according to *General Procedure A*. Data presented as: (a) amount of 4-iodobenzeneboronic acid, (b) volume of DCM, (c) amount of pinacol, (d) amount of $MgSO_4$, and (e) yield.

(a) 496 mg, 2.0 mmol, 1 eq., (b) 2.5 mL, (c) 260 mg, 2.2 mmol, 1.1 eq., (d) 241 mg, 2.0 mmol, 1 eq., and (e) 658 mg, 1.99 mmol, 99%.

Melting point: 89 – 91 °C (Lit.: 96 – 98 °C).[27]
FTIR (neat): 2974, 2930, 1584, 1385, 1356, 1323, 1140, 1086 cm^{-1}.
^{1}H NMR (400 MHz, $CDCl_3$): δ 7.72 (d, *J* = 8.1 Hz, 2H, Ar$\underline{H}$), 7.51 (d, *J* = 8.1 Hz, 2H, Ar$\underline{H}$), 1.33 (s, 12H, 4 × C$\underline{H}_3$) ppm.
^{13}C NMR (101 MHz, $CDCl_3$): δ 137.1, 136.4, 99.0, 84.2, 25.0 ppm. *To note: the carbon directly attached to the boron atom was not observed, likely due to quadrupolar relaxation.*
^{11}B NMR (128 MHz, $CDCl_3$): δ 31.10 ppm.

4,4,5,5-Tetramethyl-2-(4-(methylthio)phenyl)-1,3,2-dioxaborolane 13t[27,28,40]

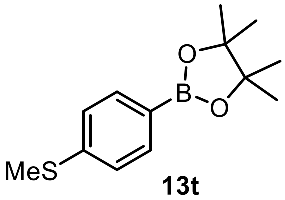

13t

Prepared according to *General Procedure A*. Data presented as: (a) amount of (4-(methylthio)phenyl)boronic acid, (b) volume of DCM, (c) amount of pinacol, (d) amount of $MgSO_4$, and (e) yield.

(a) 336 mg, 2.0 mmol, 1 eq., (b) 2.5 mL, (c) 260 mg, 2.2 mmol, 1.1 eq., (d) 241 mg, 2.0 mmol, 1 eq., and (e) 456 mg, 1.82 mmol, 91%.

Melting point: 31 – 33 °C (Lit.: 35 – 36 °C).[40]
FTIR (neat): 2976, 2924, 2361, 1595, 1544, 1458, 1393, 1356, 1142, 1101 cm^{-1}.
^{1}H NMR (400 MHz, $CDCl_3$): δ 7.71 (d, *J* = 8.2 Hz, 2H, ArH), 7.23 (d, *J* = 8.3 Hz, 2H, ArH), 2.49 (s, 3H, SCH_3), 1.34 (s, 12H, 4 × CH_3) ppm.
^{13}C NMR (101 MHz, $CDCl_3$): δ 142.7, 135.2, 125.2, 83.9, 25.0, 15.2 ppm. *To note: the carbon directly attached to the boron atom was not observed, likely due to quadrupolar relaxation.*
^{11}B NMR (128 MHz, $CDCl_3$): δ 31.17 ppm.

1.7.2 Hydrogen isotope exchange of aryl-BPins (Manuscript, Scheme 3)

All reactions were run as described in *General Procedure D.* Each experiment was carried out over three runs and the deuterium incorporation was calculated based on analysis of the ^{1}H NMR spectra of pure products following silica gel chromatography. Representative reaction yields are also provided for each substrate.

4-(4,4,5,5-Tetramethyl-1,3,2-dioxaborolan-2-yl)phenol *d*-13a

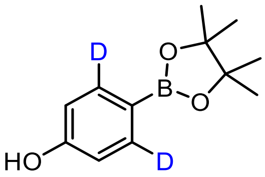

***d*-13a**

^{1}H NMR (400 MHz, $CDCl_3$): δ 7.71 (d, *J* = 8.6 Hz, 2H, ArH), 6.82 (d, *J* = 8.5 Hz, 2H, ArH), 1.33 (s, 12H, 4 × CH_3) ppm.

Deuterium incorporation expected at δ 7.71 ppm and measured against the signal at 6.82 ppm.

Substrate mass (mg)	Solvent	Temp. (°C)	Time (h)	Run 1 (%)	Run 2 (%)	Run 3 (%)	Avg (%)
**23.5**	Toluene	80	16	88	91	88	89
		Reaction yield (mg, %)		22.6, 95	20.6, 87	23.5, 99	22.2, 94
**23.5**	Toluene	80	4	93	93.5	92.5	93

2-(4-Methoxyphenyl)-4,4,5,5-tetramethyl-1,3,2-dioxaborolane *d*-13b

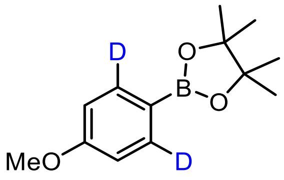

***d*-13b**

^{1}H NMR (400 MHz, $CDCl_3$): δ 7.75 (d, *J* = 8.7 Hz, 2H ArH), 6.90 (d, *J* = 8.7 Hz, 2H, ArH), 3.83 (s, 3H, OCH_3), 1.33 (s, 12H, 4 × CH_3) ppm.

Deuterium incorporation expected at δ 7.75 ppm and measured against the signal at δ 6.90 ppm.

Substrate mass (mg)	Solvent	Temp. (°C)	Time (h)	Run 1 (%)	Run 2 (%)	Run 3 (%)	Avg (%)
**25.0**	Toluene	80	16	93	92.5	93	93
		Reaction yield (mg, %)		24.1, 95	24.7, 98	25.0, 99	24.6, 97
**25.0**	Toluene	80	4	94	94	94	94

4,4,5,5-Tetramethyl-2-(*p*-tolyl)-1,3,2-dioxaborolane 13c

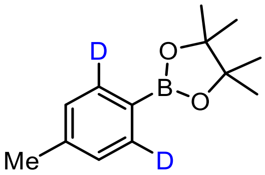

***d*-13c**

^{1}H NMR (400 MHz, $CDCl_3$): δ 7.70 (d, J = 7.9 Hz, 2H, ArH), 7.19 (d, J = 7.5 Hz, 2H, ArH), 1.55 (s, 3H, Ar-CH_3), 1.34 (s, 12H, 4 × CH_3) ppm.

Deuterium incorporation expected at δ 7.70 ppm and measured against the signal at δ 7.19 ppm.

Substrate mass (mg)	Solvent	Temp. (°C)	Time (h)	Run 1 (%)	Run 2 (%)	Run 3 (%)	Avg (%)
**23.3**	Toluene	80	16 h	90.5	91	91	91
		Reaction yield (mg, %)		20.5, 87	20.5, 87	18.8, 80	19.9, 85
**23.3**	Toluene	80	4	91	93	92	92

2-(4-(*Tert*-butyl)phenyl)-4,4,5,5-tetramethyl-1,3,2-dioxaborolane *d*-13d

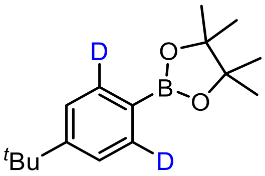

***d*-13d**

^{1}H NMR (400 MHz, $CDCl_3$): δ 7.77 (d, J = 8.3 Hz, 2H, ArH), 7.41 (d, J = 8.3 Hz, 2H, ArH), 1.34 (s, 12H, 4 × CH_3), 1.33 (s, 9H, 3 × CH_3 (^{t}Bu)) ppm.

Deuterium incorporation expected at δ 7.77 ppm and measured against the signal at δ 7.41 ppm.

**Substrate mass (mg)**	**Solvent**	**Temp. (°C)**	**Time (h)**	**Run 1 (%)**	**Run 2 (%)**	**Run 3 (%)**	**Avg (%)**
**27.8**	Toluene	80	16	70	72.5	73	72
		Reaction yield (mg, %)		23.1, 83	26.5, 95	26.8, 96	25.5, 91

2-([1,1'-Biphenyl]-4-yl)-4,4,5,5-tetramethyl-1,3,2-dioxaborolane *d*-13e

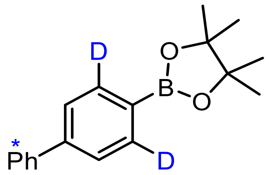

***d*-13e**

^{1}H NMR (400 MHz, CDCl$_3$): δ 7.89 (d, *J* = 8.3 Hz, 2H, ArH), 7.65 - 7.59 (m, 4H, ArH + ArH (Ph)), 7.45 (t, *J* = 7.4 Hz, 2H, Ar-H (Ph)), 7.36 (t, *J* = 7.3 Hz, 1H, ArH (Ph)), 1.37 (s, 12H, 4 × CH$_3$) ppm.

Deuterium incorporation expected at δ 7.89 ppm and measured against the signal at δ 7.36 ppm.

**Substrate mass (mg)**	**Solvent**	**Temp. (°C)**	**Time (h)**	**Run 1 (%)[a]**	**Run 2 (%)[b]**	**Run 3 (%)[c]**	**Avg (%)**
**30.0**	Toluene	80	16	90	92	91.5	91[a]
		Reaction yield (mg, %)		25.6, 85	-	-	-
**30.0**	Toluene	80	4	91.5	92	92	92[b]

[a]Average of 6%D on Ph ring at *ortho*-position; b Average of 16%D on Ph ring at *ortho*-position.

2-(4-Chlorophenyl)-4,4,5,5-tetramethyl-1,3,2-dioxaborolane *d*-13f

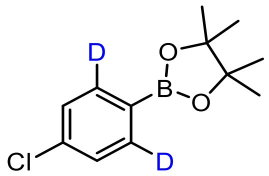

***d*-13f**

^{1}H NMR (400 MHz, CDCl$_3$): δ 7.73 (d, *J* = 8.3 Hz, 2H, ArH) 7.34 (d, *J* = 8.4 Hz, 2H, ArH), 1.34 (s, 12H, 4 × CH$_3$) ppm.

Deuterium incorporation expected at δ 7.73 ppm and measured against the signal at δ 7.34 ppm.

Substrate mass (mg)	Solvent	Temp. (°C)	Time (h)	Run 1 (%)	Run 2 (%)	Run 3 (%)	Avg (%)
**25.5**	Toluene	80	16	73	70.5	71	72
		Reaction yield (mg, %)		25.8, 100	-	23.8, 93	24.8, 97
**25.5**	PhF	80	16	89.5	90	89.5	90
**25.5**	$PhCF_3$	80	16	92.5	93	93	93

2-(4-Fluorophenyl)-4,4,5,5-tetramethyl-1,3,2-dioxaborolane *d*-13g

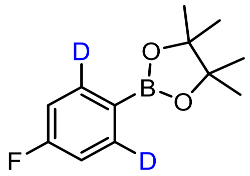

***d*-13g**

^{1}H NMR (400 MHz, CDCl$_3$): δ 7.80 (dd, *J* = 8.7 Hz, $^4J_{H-F}$ = 6.2 Hz, 2H, ArH), 7.05 (dd, *J* = 8.7 Hz, $^3J_{H-F}$ = 9.2 Hz, 2H, ArH), 1.34 (s, 12H, 4 × CH$_3$) ppm.

Deuterium incorporation expected at δ 7.80 ppm and measured against the signal at δ 7.05 ppm.

Substrate mass (mg)	Solvent	Temp. (°C)	Time (h)	Run 1 (%)	Run 2 (%)	Run 3 (%)	Avg (%)
**23.8**	Toluene	80	16	47.5	47.5	53	49
		Reaction yield (mg %)		18.9, 79	18.1, 76	16.9, 71	18.0, 75
**23.8**	PhF	80	16	75	71.5	78	75
**23.8**	$PhCF_3$	80	16	79	78	80	79

2-(4-Bromophenyl)-4,4,5,5-tetramethyl-1,3,2-dioxaborolane *d*-13h[41]

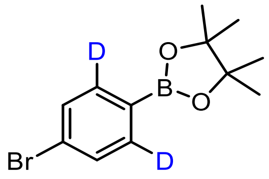

***d*-13h**

*Note: the starting substrate **13h** was purchased from a commercial supplier; CAS: [68716-49-4].*

^{1}H NMR (400 MHz, $CDCl_3$): δ 7.66 (d, *J* = 8.3 Hz, 2H, ArH), 7.50 (d, *J* = 8.3 Hz, 2H, ArH),1.34 (s, 12H, 4 × CH_3) ppm.

Deuterium incorporation expected at δ 7.66 ppm and measured against the signal at δ 7.50 ppm.

Substrate mass (mg)	Solvent	Temp. (°C)	Time (h)	Run 1 (%)	Run 2 (%)	Run 3 (%)	Avg (%)
**30.3**	Toluene	80	16	31.5	31.5	32	32
		Reaction yield (mg, %)		30.0, 99	30.0, 99	29.7, 98	99
**30.3**	PhF	80	16	47.5	47.5	47	47
**30.3**	$PhCF_3$	80	16	59.5	59.5	61	60

4,4,5,5-Tetramethyl-2-(4-(trifluoromethyl)phenyl)-1,3,2-dioxaborolane *d*-13i[17]

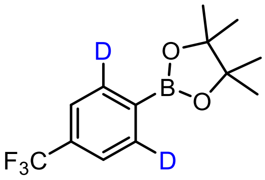

***d*-13i**

*Note: the starting substrate **13i** was purchased from a commercial supplier; CAS: [214360-65-3].*

^{1}H NMR (500 MHz, CDCl$_3$): δ 7.91 (d, *J* = 8.0 Hz, 2H, ArH), 7.61 (d, *J* = 8.0 Hz, 2H, ArH), 1.36 (s, 12 H, 4 × CH$_3$) ppm.

Deuterium incorporation expected at δ 7.91 ppm and measured against the signal at δ 7.61 ppm.

Substrate mass (mg)	Solvent	Temp. (°C)	Time (h)	Run 1 (%)	Run 2 (%)	Run 3 (%)	Avg (%)
**29.1**	Toluene	80	16 h	20	20.5	17.5	19
		Reaction yield (mg, %)		25.5, 87	25.7, 88	26.0, 89	25.7, 88
**29.1**	PhF	80	16	28.5	30	27	29
**29.1**	PhCF$_3$	80	16	35	35	36	35

2-(4,4,5,5-Tetramethyl-1,3,2-dioxaborolan-2-yl)phenol *d*-13j

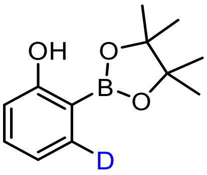

***d*-13j**

^{1}H NMR (400 MHz, CDCl$_3$): δ 7.80 (s, 1H, ArOH), 7.61 (dd, *J* = 7.6 Hz, $^4J_{H\text{-}H}$ = 1.8 Hz, 1H, ArH), 7.37 (td, *J* = 7.6 Hz, $^4J_{H\text{-}H}$ = 1.7 Hz, 1H, ArH), 6.93 - 6.84 (m, 2H, ArH), 1.37 (s, 12H, 4 × CH$_3$) ppm.

Deuterium incorporation expected at δ 7.61 ppm and measured against the signal at δ 7.37 ppm.

**Substrate mass (mg)**	**Solvent**	**Temp. (°C)**	**Time (h)**	**Run 1 (%)**	**Run 2 (%)**	**Run 3 (%)**	**Avg (%)**
**23.5**	Toluene	80	4	82	82	82	82
		Reaction yield (mg, %)		21.2, 90	20.5, 87	22.1, 94	21.3, 90

2-(2-Methoxyphenyl)-4,4,5,5-tetramethyl-1,3,2-dioxaborolane *d*-13k

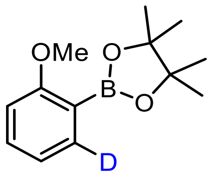

***d*-13k**

^{1}H NMR (400 MHz, CDCl$_3$): δ 7.67 (dd, J = 7.3 Hz, $^4J_{H\text{-}H}$ =1.9 Hz, 1H, ArH), 7.39 (ddd, J = 8.3, 7.3 Hz, $^4J_{H\text{-}H}$ = 1.9 Hz, 1H, ArH), 6.94 (td, J = 7.3 Hz, $^4J_{H\text{-}H}$ = 0.9 Hz, 1H, ArH), 6.85 (d, J = 8.2 Hz, 1H, ArH), 3.83 (s, 3H, OCH$_3$), 1.36 (s, 12H, 4 × CH$_3$) ppm.

Deuterium incorporation expected at δ 7.67 ppm and measured against signal at δ 6.85 ppm.

**Substrate mass (mg)**	**Solvent**	**Temp. (°C)**	**Time (h)**	**Run 1 (%)**	**Run 2 (%)**	**Run 3 (%)**	**Avg (%)**
**25.0**	Toluene	80	4	95	95	95	95
		Reaction yield (mg, %)		23.7, 94	23.3, 93	22.0, 87	23.0, 91

4,4,5,5-Tetramethyl-2-(*o*-tolyl)-1,3,2-dioxaborolane *d*-13l

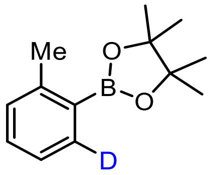

***d*-13l**

^{1}H NMR (400 MHz, CDCl$_3$): δ 7.76 (dd, J = 7.8 Hz, $^4J_{H-H}$ = 1.6 Hz, 1H, Ar$\underline{H}$), 7.31 (td, J = 7.5 Hz, $^4J_{H-H}$ = 1.5 Hz, 1H, Ar$\underline{H}$), 7.19 - 7.13 (m, 2H, Ar$\underline{H}$), 2.54 (s, 3H, ArC$\underline{H}_3$), 1.35 (s, 12H, 4 × C$\underline{H}_3$) ppm.

Deuterium incorporation expected at δ 7.76 ppm and measured against signal at δ 7.31 ppm.

**Substrate mass (mg)**	**Solvent**	**Temp. (°C)**	**Time (h)**	**Run 1 (%)**	**Run 2 (%)**	**Run 3 (%)**	**Avg (%)**
**23.3**	Toluene	80	4	95	94	95	95
		**Reaction yield** (mg, %)		17.2, 74	16.0, 70	17.4, 74	16.9, 73

3-(4,4,5,5-Tetramethyl-1,3,2-dioxaborolan-2-yl)phenol *d*-13m

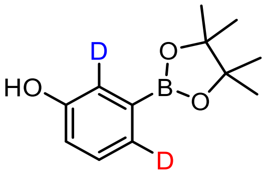

***d*-13m**

^{1}H NMR (400 MHz, DMSO): δ 9.31 (s, 1H, ArO$\underline{H}$), 7.18 (t, J = 8.0 Hz, 1H, Ar$\underline{H}$), 7.10 - 7.06 (m, 2H, Ar$\underline{H}$), 6.86 (ddd, J = 8.0, $^4J_{H-H(OH)}$ = 2.7, $^4J_{H-H}$ = 1.1 Hz, 1H, Ar$\underline{H}$), 1.28 (s, 12H, 4 × C$\underline{H}_3$) ppm.

Deuterium incorporation expected at δ 7.10 - 7.06 ppm and measured against the signal at δ 6.86 ppm.

Substrate mass (mg)	Solvent	Temp. (°C)	Time (h)	Run 1 (%)	Run 2 (%)	Run 3 (%)	Avg (%)
**23.5**	Toluene	80	4	89	89	89	89
		Reaction yield (mg, %)		20.2, 85	23.0, 97	19.4, 82	20.9, 88

To note: due to an overlap of anticipated labelled peaks, incorporation is reported as an average over both ortho-sites and the reaction yield is based on an 89% D2 product.

2-(3-Methoxyphenyl)-4,4,5,5-tetramethyl-1,3,2-dioxaborolane *d*-13n

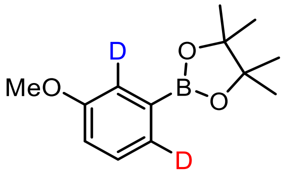

***d*-13n**

^{1}H NMR (400 MHz, CDCl$_3$): δ 7.40 (dt, J = 7.2 Hz, $^{4}J_{H\text{-}H}$ = 1.1 Hz, 1H, ArH), 7.33 (d, $^{4}J_{H\text{-}H}$ = 2.7 Hz, 1H, ArH), 7.29 (t, J = 7.3 Hz, 1H, ArH), 7.01 (ddd, J = 8.2 Hz, $^{4}J_{H\text{-}H}$ = 2.7 Hz, $^{4}J_{H\text{-}H}$ = 1.2 Hz, 1H, ArH), 3.83 (s, 3H, OCH$_3$), 1.35 (s, 12H, 4 × CH$_3$) ppm.

Deuterium incorporation expected at δ 7.40 (D) and δ 7.33 (D) ppm, and measured against the signal at δ 7.01 ppm.

Substrate mass (mg)	Solvent	Temp. (°C)	Time (h)	Run 1 (%)		Run 2 (%)		Run 3 (%)		Avg (%)	
				D	D	D	D	D	D	D	D
**25.0**	Toluene	80	4	86	91	85	91	86	91	86	91
		Reaction yield (mg, %)		20.3, 80		21.0, 83		24.0, 95		21.8, 86	

4,4,5,5-Tetramethyl-2-(*m*-tolyl)-1,3,2-dioxaborolane *d*-13o

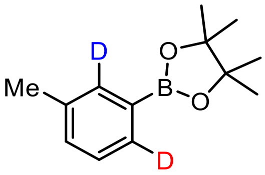

***d*-13o**

^{1}H NMR (400 MHz, CDCl$_3$): δ 7.64 (s, 1H, ArH), 7.63 - 7.59 (m, 1H, ArH), 7.28 - 7.26 (m, 2H, ArH), 2.36 (s, 3H, ArCH$_3$), 1.35 (s, 12H, 4 × CH$_3$) ppm.

Deuterium incorporation expected at δ 7.64 (D) and δ 7.63 - 7.59 (D) ppm, and measured against the signal at δ 7.28 - 7.26 ppm.

**Substrate mass (mg)**	**Solvent**	**Temp. (°C)**	**Time (h)**	**Run 1 (%)**		**Run 2 (%)**		**Run 3 (%)**		**Avg (%)**	
				**D**	**D**	**D**	**D**	**D**	**D**	**D**	**D**
**23.3**	Toluene	80	4	23	91	21	91	22	91	22	91
			Reaction yield (mg, %)	17.2, 73		18.7, 80		22.5, 94		82	

2-(3,5-Dimethylphenyl)-4,4,5,5-tetramethyl-1,3,2-dioxaborolane *d*-13p

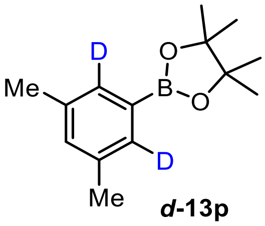

***d*-13p**

^{1}H NMR (400 MHz, CDCl$_3$): δ 7.44 (s, 2H, ArH), 7.10 (s, 1H, ArH), 2.32 (s, 6H, 2 × ArCH$_3$), 1.35 (s, 12H, 4 × CH$_3$) ppm.

Deuterium incorporation expected at δ 7.44 ppm and measured against the signal at δ 7.10 ppm.

Substrate mass (mg)	Solvent	Temp. (°C)	Time (h)	Run 1 (%)	Run 2 (%)	Run 3 (%)	Avg (%)
**24.8**	Toluene	80	16	12	16	12	13
		Reaction yield (mg, %)		23.4, 94	22.5, 90	22.6, 91	22.8, 92

2-(3-Fluoro-4-methoxyphenyl)-4,4,5,5-tetramethyl-1,3,2-dioxaborolane *d*-13q

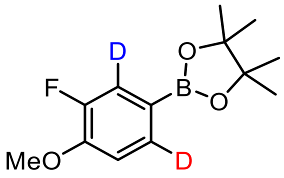

***d*-13q**

^{1}H NMR (400 MHz, CDCl$_3$): δ 7.53 (dt, J = 8.2 Hz, $^4J_{H\text{-}H}$ = 1.0 Hz, 1H, ArH), 7.49 (dd, $^3J_{H\text{-}F}$ = 11.7 Hz, $^4J_{H\text{-}H}$ = 1.4 Hz, 1H, ArH), 6.95 (t, J = 8.1 Hz, 1H, ArH), 3.91 (s, 3H, OCH$_3$), 1.33 (s, 12H, 4 × CH$_3$) ppm.

Deuterium incorporation expected at δ 7.53 (D) and δ 7.49 (D) ppm, and measured against the signal at δ 6.95 ppm.

Substrate mass (mg)	Solvent	Temp. (°C)	Time (h)	Run 1 (%)		Run 2 (%)		Run 3 (%)		Avg (%)	
				D	D	D	D	D	D	D	D
**27.0**	Toluene	80	16	83	77	86	83	84	78	84	79
		Reaction yield (mg, %)		25.8, 95		26.2, 96		25.1, 93		25.7, 95	

N-Methyl-5-(4,4,5,5-tetramethyl-1,3,2-dioxaborolan-2-yl)-1H-indole d-13r

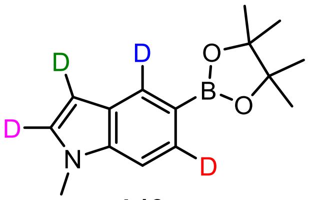

***d*-13r**

^{1}H NMR (400 MHz, $CDCl_3$): δ 8.17 (s, 1H, ArH), 7.68 (dd, J = 8.3 Hz, $^4J_{H\text{-}H}$ = 1.0 Hz, 1H, ArH), 7.33 (d, J = 8.3 Hz, 1H, ArH), 7.04 (d, J = 3.1 Hz, 1H, ArH), 6.51 (dd, J = 3.1 Hz, $^4J_{H\text{-}H}$ = 0.9 Hz, 1H, ArH), 3.79 (s, 3H, NC$\underline{H}_3$), 1.38 (s, 12H, 4 x C$\underline{H}_3$) ppm.

Deuterium incorporation expected at δ 8.17 (D) and δ 7.68 (D) ppm, and measured against the signal at δ 7.33 ppm.

**Substrate mass (mg)**	**Solvent**	**Temp. (°C)**	**Time (h)**	**Run 1 (%)**		**Run 2 (%)**		**Run 3 (%)**		**Avg (%)**	
				**D**	**D**	**D**	**D**	**D**	**D**	**D**	**D**
**27.5**	Toluene	r.t.	16	55	72	52	70	52	69	53	70
		Reaction yield (mg, %)		21.7, 78		20.0, 72		20.9, 76		20.8, 75	

Note: 30%D avg. observed on the indole C-3 position, and 17%D avg. observed on the indole C-2 position.

2-(4-Iodophenyl)-4,4,5,5-tetramethyl-1,3,2-dioxaborolane *d*-13s

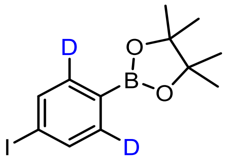

***d*-13s**

^{1}H NMR (400 MHz, $CDCl_3$): δ 7.72 (d, *J* = 8.1 Hz, 2H, ArH), 7.51 (d, *J* = 8.1 Hz, 2H, ArH), 1.33 (s, 12H, 4 × CH_3) ppm.

Deuterium incorporation expected at δ 7.72 ppm and measured against the signal at δ 7.51 ppm.

Substrate mass (mg)	Solvent	Temp. (°C)	Time (h)	Run 1 (%)	Run 2 (%)	Run 3 (%)	Avg (%)
**35.3**	Toluene	80	16	0	0	0	0
	Returned starting material (mg, %)			34.6, 98	35.0, 99	34.7, 97	34.8, 98

4,4,5,5-Tetramethyl-2-(4-(methylthio)phenyl)-1,3,2-dioxaborolane *d*-13t

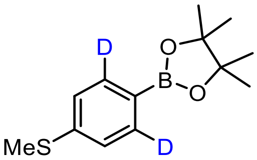

***d*-13t**

^{1}H NMR (400 MHz, $CDCl_3$): δ 7.71 (d, *J* = 8.2 Hz, 2H, ArH), 7.23 (d, *J* = 8.3 Hz, 2H, ArH), 2.49 (s, 3H, SCH_3), 1.34 (s, 12H, 4 × CH_3) ppm.

Deuterium incorporation expected at δ 7.71 ppm and measured against the signal at δ 7.23 ppm.

**Substrate mass (mg)**	**Solvent**	**Temp. (°C)**	**Time (h)**	**Run 1 (%)**	**Run 2 (%)**	**Run 3 (%)**	**Avg (%)**
**26.8**	Toluene	80	16	0	0	0	0
	Returned starting material (mg, %)			22.1, 82	21.5, 80	21.1, 79	21.6, 80

1.8 Substrate Scope – Aryl Boronic Neopentyl Esters (Aryl-BNeos)

1.8.1 Substrate synthesis

2-(4-Methoxyphenyl)-5,5-dimethyl-1,3,2-dioxaborinane 14a[17,42,43]

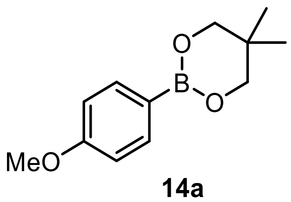

14a

Prepared according to *General Procedure A*. Data presented as: (a) amount of 4-methoxybenzeneboronic acid, (b) volume of DCM, (c) amount of neopentyl glycol, (d) amount of $MgSO_4$, and (e) yield.

(a) 304 mg, 2.0 mmol, 1 eq., (b) 2.5 mL, (c) 229 mg, 2.2 mmol, 1.1 eq., (d) 241 mg, 2.0 mmol, 1 eq., and (e) 432 mg, 1.96 mmol, 98%.

Melting point: 56 – 58 °C (Lit.: 57 – 59 °C).[43]

FTIR (neat): 2955, 2899, 2837, 2359, 1601, 1568, 1481, 1342, 1296, 1242, 1173, 1128, 1026 cm^{-1}.

^{1}H NMR (400 MHz, $CDCl_3$): δ 7.75 (d, *J* = 8.7 Hz, 2H, ArH), 6.89 (d, *J* = 8.7 Hz, 2H, ArH), 3.82 (s, 3H, OCH_3), 3.75 (s, 4H, 2 × CH_2), 1.02 (s, 6H, 2 × CH_3) ppm.

^{13}C NMR (101 MHz, $CDCl_3$): δ 161.9, 135.7, 113.3, 72.4, 55.2, 32.0, 22.1 ppm. *To note: the carbon directly attached to the boron atom was not observed, likely due to quadrupolar relaxation.*

^{11}B NMR (128 MHz, $CDCl_3$): δ 26.90 ppm.

5,5-Dimethyl-2-(*p*-tolyl)-1,3,2-dioxaborinane 14b[17,42,43]

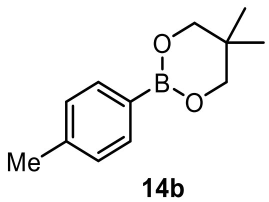

14b

Prepared according to *General Procedure A*. Data presented as: (a) amount of 4-methylbenzeneboronic acid, (b) volume of DCM, (c) amount of neopentyl glycol, (d) amount of $MgSO_4$, and (e) yield.

(a) 245 mg, 1.8 mmol, 1 eq., (b) 2.5 mL, (c) 206 mg, 1.98 mmol, 1.1 eq., (d) 217 mg, 1.8 mmol, 1 eq., and (e) 330 mg, 1.62 mmol, 90%.

Melting point: 93 – 94 °C (lit.: 94 – 95 °C).[43]
FTIR (neat): 2960, 2939, 2905, 2872, 2357, 1587, 1477, 1421, 1375, 1340, 1305, 1247, 1128 1014 cm^{-1}.
^{1}H NMR (400 MHz, $CDCl_3$): δ 7.70 (d, *J* = 8.0 Hz, 2H, ArH), 7.17 (d, *J* = 8.0 Hz, 2H, ArH), 3.76 (s, 4H, 2 × CH_2), 2.36 (s, 3H, $ArCH_3$), 1.02 (s, 6H, 2 × CH_3) ppm.
^{13}C NMR (101 MHz, $CDCl_3$): δ 140.8, 134.0, 128.6, 72.4, 32.0, 22.1, 21.8 ppm. *To note: the carbon directly attached to the boron atom was not observed, likely due to quadrupolar relaxation.*
^{11}B NMR (128 MHz, $CDCl_3$): δ 27.07 ppm.

2-([1,1'-Biphenyl]-4-yl)-5,5-dimethyl-1,3,2-dioxaborinane 14c[38,44,45]

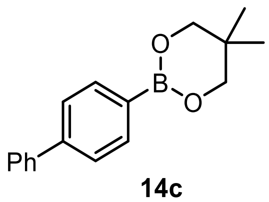

14c

Prepared according to *General Procedure A*. Data presented as: (a) amount of 4-phenylbenzeneboronic acid, (b) volume of DCM, (c) amount of neopentyl glycol, (d) amount of $MgSO_4$, and (e) yield.

(a) 396 mg, 2.0 mmol, 1 eq., (b) 5 mL, (c) 229 mg, 2.2 mmol, 1.1 eq., (d) 240 mg, 2.0 mmol, 1 eq., and (e) 490 mg, 1.84 mmol, 92%.

Melting Point : 106 – 108 °C (Lit.: 107.7 – 108.3 °C).[45]

FTIR (neat): 2958, 2931, 1597, 1516, 1475, 1420, 1377, 1342, 1311, 1265 1246, 1182, 1132, 1072, 1022, 1006, 931, 910, 835, 815, 765, 734, 695, 667 cm^{-1}.

^{1}H NMR (400 MHz, $CDCl_3$): δ 8.06 (d, *J* = 8.2 Hz, 2H, ArH), 7.78 - 7.72 (m, 4H, ArH), 7.55 (t, *J* = 7.5 Hz, 2H, ArH), 7.45 (t, *J* = 7.4 Hz, 1H, ArH), 3.88 (s, 4H, 2 × CH_2), 1.13 (s, 6H, 2 × CH_3) ppm.

^{13}C NMR (101 MHz, $CDCl_3$): δ 143.3, 141.3, 134.5, 128.8, 127.5, 127.3, 126.4, 72.3, 31.9, 21.9 ppm. *To note: the carbon directly attached to the boron atom was not observed, likely due to quadrupolar relaxation.*

^{11}B NMR (128 MHz, $CDCl_3$): δ 27.38 ppm.

2-(4-(*Tert*-butyl)phenyl)-5,5-dimethyl-1,3,2-dioxaborinane 14d[46,47]

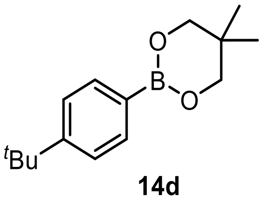

14d

Prepared according to *General Procedure A*. Data presented as: (a) amount of 4-*tert*-butylbenzeneboronic acid, (b) volume of DCM, (c) amount of neopentyl glycol, (d) amount of $MgSO_4$, and (e) yield.

(a) 356 mg, 2.0 mmol, 1 eq., (b) 5 mL, (c) 229 mg, 2.2 mmol, 1.1 eq., (d) 240 mg, 2.0 mmol, 1 eq., and (e) 426 mg, 1.73 mmol, 87%.

Melting Point : 74 – 76 °C (Lit.: 78 – 78.5 °C).[47]
FTIR (neat): 1604, 1477, 1406, 1346, 1317, 1134, 1115, 669, 644 cm^{-1}.
^{1}H NMR (400 MHz, $CDCl_3$): δ 7.81 (d, *J* = 8.3 Hz, 2H, ArH), 7.44 (d, *J* = 8.3 Hz, 2H, ArH), 3.80 (s, 4H, 2 × CH_2), 1.38 (s, 9H, $C(CH_3)_3$), 1.05 (s, 6H, 2 × CH_3) ppm.
^{13}C NMR (101 MHz, $CDCl_3$): δ 153.9, 133.9, 124.7, 72.4, 34.9, 32.0, 31.4, 22.0 ppm. *To note: the carbon directly attached to the boron atom was not observed, likely due to quadrupolar relaxation.*
^{11}B NMR (128 MHz, $CDCl_3$): δ 27.06 ppm.

2-(4-Chlorophenyl)-5,5-dimethyl-1,3,2-dioxaborinane 14e[44,46,48]

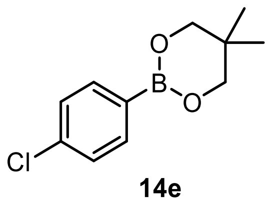

14e

Prepared according to *General Procedure A*. Data presented as: (a) amount of 4-chlorobenzeneboronic acid, (b) volume of DCM, (c) amount of neopentyl glycol, (d) amount of $MgSO_4$, and (e) yield.

(a) 313 mg, 2.0 mmol, 1 eq., (b) 2.5 mL, (c) 229 mg, 2.2 mmol, 1.1 eq., (d) 241 mg, 2.0 mmol, 1 eq., and (e) 374 mg, 1.67 mmol, 84%.

Melting point: 97 – 99 °C (Lit.: 97 – 98 °C).[48]

FTIR (neat): 2959, 2940, 2903, 1587, 1476, 1421, 1308, 1250, 1130, 1084, 1015, 826, 741, 725, 637 cm^{-1}.

^{1}H NMR (400 MHz, $CDCl_3$): δ 7.72 (d, *J* = 8.3 Hz, 2H, ArH), 7.32 (d, *J* = 8.4 Hz, 2H, ArH), 3.76 (s, 4H, 2 × CH_2), 1.02 (s, 6H, 2 × CH_3) ppm.

^{13}C NMR (101 MHz, $CDCl_3$): δ 137.0, 135.4, 128.0, 72.5, 32.0, 22.0 ppm. *To note: the carbon directly attached to the boron atom was not observed, likely due to quadrupolar relaxation.*

^{11}B NMR (128 MHz, $CDCl_3$): δ 26.87 ppm.

2-(4-Fluorophenyl)-5,5-dimethyl-1,3,2-dioxaborinane 14f[42,43,46]

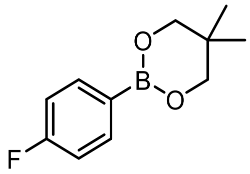

14f

Prepared according to *General Procedure A*. Data presented as: (a) amount of 4-fluorobenzeneboronic acid, (b) volume of DCM, (c) amount of neopentyl glycol, (d) amount of $MgSO_4$, and (e) yield.

(a) 280 mg, 2.0 mmol, 1 eq., (b) 5 mL, (c) 229 mg, 2.2 mmol, 1.1 eq., (d) 240 mg, 2.0 mmol, 1 eq., and (e) 358 mg, 1.72 mmol, 86%.

Melting Point: 64 – 66 °C (Lit.: 66 – 67 °C).[43]
FTIR (neat): 2964, 1595, 1479, 1305, 1247, 1128, 831, 640 cm^{-1}.
^{1}H NMR (400 MHz, $CDCl_3$): δ 7.85 (dd, J = 8.5 Hz, $^4J_{H\text{-}F}$ = 6.3 Hz, 2H, Ar<u>H</u>), 7.07 (dd, $^3J_{H\text{-}F}$ = 9.5 Hz, J = 8.7 Hz, 2H, Ar<u>H</u>), 3.78 (s, 4H, 2 × C<u>H</u>$_2$), 1.03 (s, 6H, 2 × C<u>H</u>$_3$) ppm.
^{13}C NMR (101 MHz, $CDCl_3$): δ 165.0 (d, $^1J_{C\text{-}F}$ = 249.2 Hz), 136.2 (d, $^3J_{C\text{-}F}$ = 8.0 Hz), 114.6 (d, $^2J_{C\text{-}F}$ = 19.9 Hz), 72.4, 31.9, 21.9 ppm. *To note: the carbon directly attached to the boron atom was not observed, likely due to quadrupolar relaxation.*
^{11}B NMR (128 MHz, $CDCl_3$): δ 26.68 ppm.
^{19}F NMR (376 MHz, $CDCl_3$): δ -109.73 ppm.

2-(4-Bromophenyl)-5,5-dimethyl-1,3,2-dioxaborinane 14g[49,50]

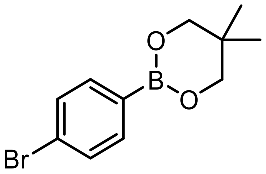

14g

Prepared according to *General Procedure A*. Data presented as: (a) amount of 4-bromobenzeneboronic acid, (b) volume of DCM, (c) amount of neopentyl glycol, (d) amount of $MgSO_4$, and (e) yield.

(a) 401 mg, 2.0 mmol, 1 eq., (b) 5 mL, (c) 229 mg, 2.2 mmol, 1.1 eq., (d) 240 mg, 2.0 mmol, 1 eq., and (e) 494 mg, 1.84 mmol, 92%.

Melting Point: 108 – 110 °C (Lit.: 107 – 110 °C).[49]
FTIR (neat): 2958, 2903, 1581, 1477, 1422 cm^{-1}.
^{1}H NMR (400 MHz, $CDCl_3$): δ 7.65 (d, *J* = 8.3 Hz, 2H, ArH), 7.48 (d, *J* = 8.4 Hz, 2H, ArH), 3.76 (s, 4H, 2 × CH_2), 1.02 (s, 6H, 2 × CH_3) ppm.
^{13}C NMR (101 MHz, $CDCl_3$): δ 135.6, 130.9, 125.7, 72.5, 32.0, 22.0 ppm. *To note: the carbon directly attached to the boron atom was not observed, likely due to quadrupolar relaxation.*
^{11}B NMR (128 MHz, $CDCl_3$): δ 26.70 ppm.

5,5-Dimethyl-2-(4-(trifluoromethyl)phenyl)-1,3,2-dioxaborinane 14h[17,42,43]

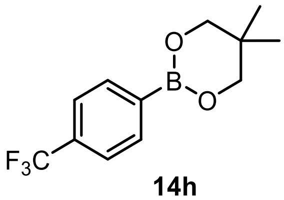

14h

Prepared according to *General Procedure A*. Data presented as: (a) amount of 4-trifluoromethylbenzeneboronic acid, (b) volume of DCM, (c) amount of neopentyl glycol, (d) amount of $MgSO_4$, and (e) yield.

(a) 380 mg, 2.0 mmol, 1 eq., (b) 2.5 mL, (c) 229 mg, 2.2 mmol, 1.1 eq., (d) 241 mg, 2.0 mmol, 1 eq., and (e) 435 mg, 1.69 mmol, 85%.

Melting point: 112 – 114 °C (Lit.: 110 – 113 °C).[43]

FTIR (neat): 2963, 2913, 2876, 1517, 1481, 1423, 1292, 1252, 1155, 1113, 1101, 1062, 839 cm^{-1}.

^{1}H NMR (400 MHz, $CDCl_3$): δ 7.92 (d, *J* = 8.3 Hz, 2H, Ar<u>H</u>), 7.62 (d, *J* = 8.3 Hz, 2H, Ar<u>H</u>), 3.81 (s, 4H, 2 × $C\underline{H}_2$), 1.06 (s, 6H, 2 × $C\underline{H}_3$) ppm.

^{13}C NMR (101 MHz, $CDCl_3$): δ 134.3, 132.4 (q, $^2J_{C-F}$ = 32.1 Hz), 124.5 (q, $^1J_{C-F}$ = 272.4 Hz), 124.3 (q, $^3J_{C-F}$ = 3.8 Hz), 72.6, 32.1, 22.0 ppm. *To note: the carbon directly attached to the boron atom was not observed, likely due to quadrupolar relaxation.*

^{11}B NMR (128 MHz, $CDCl_3$): δ 26.75 ppm.

^{19}F NMR (376 MHz, $CDCl_3$): δ -62.90 ppm.

2-(4-(Benzyloxy)phenyl)-5,5-dimethyl-1,3,2-dioxaborinane 14i[51]

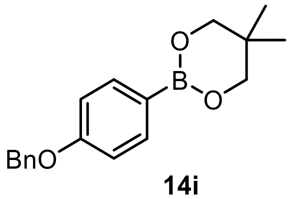

14i

Prepared according to *General Procedure A*. Data presented as: (a) amount of 4-benzyloxybenzeneboronic acid, (b) volume of DCM, (c) amount of neopentyl glycol, (d) amount of $MgSO_4$, and (e) yield.

(a) 456 mg, 2.0 mmol, 1 eq., (b) 5 mL, (c) 229 mg, 2.2 mmol, 1.1 eq., (d) 240 mg, 2.0 mmol, 1 eq., and (e) 492 mg, 1.66 mmol, 83%.

Melting point: 92 – 94 °C.

FTIR (neat): 3037, 2951, 1600, 1477, 1342, 1311, 1242, 1174, 1130, 1109, 1022, 1006, 914, 831, 812, 734, 698, 648 cm^{-1}.

^{1}H NMR (400 MHz, CDCl$_3$): δ 7.75 (d, *J* = 8.7 Hz, 2H, ArH), 7.46 - 7.42 (m, 2H, ArH), 7.38 (ddd, *J* = 8.1, 6.9 Hz, $^4J_{H-H}$ = 1.0 Hz, 2H, ArH), 7.34 - 7.28 (m, 1H, ArH), 6.96 (d, *J* = 8.8 Hz, 2H, ArH), 5.09 (s, 2H, OCH$_2$), 3.75 (s, 4H, 2 × BOCH$_2$), 1.02 (s, 6H, 2 × CH$_3$,) ppm.

^{13}C NMR (101 MHz, CDCl$_3$): δ 161.1, 137.1, 135.7, 128.7, 128.1, 127.6, 114.2, 72.4, 69.9, 32.0, 22.1 ppm. *To note: the carbon directly attached to the boron atom was not observed, likely due to quadrupolar relaxation.*

^{11}B NMR (128 MHz, CDCl$_3$): δ 27.03 ppm.

5,5-Dimethyl-2-(*o*-tolyl)-1,3,2-dioxaborinane 14j[42,52]

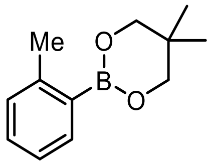

14j

Prepared according to *General Procedure A*. Data presented as: (a) amount of 2-methylbenzeneboronic acid, (b) volume of DCM, (c) amount of neopentyl glycol, (d) amount of MgSO$_4$, and (e) yield.

(a) 272 mg, 2.0 mmol, 1 eq., (b) 5 mL, (c) 229 mg, 2.2 mmol, 1.1 eq., (d) 240 mg, 2.0 mmol, 1 eq., and (e) 313 mg, 1.53 mmol, 77%.

Melting Point : 87 – 89 °C.

FTIR (neat): 2958, 1598, 1440, 1377, 1242, 1134, 813, 644 cm^{-1}.

^{1}H NMR (400 MHz, CDCl$_3$): δ 7.78 (d, *J* = 7.3 Hz, 1H, ArH), 7.32 (td, *J* = 7.6, 1.6 Hz, 1H, ArH), 7.19 (app. t, 2H, ArH), 3.81 (s, 4H, 2 × CH$_2$), 2.57 (s, 3H, ArCH$_3$), 1.07 (s, 6H, 2 × CH$_3$) ppm.

^{13}C NMR (101 MHz, CDCl$_3$): δ 144.0, 135.0, 130.1, 130.1, 124.8, 72.4, 31.7, 22.5, 22.0 ppm. *To note: the carbon directly attached to the boron atom was not observed, likely due to quadrupolar relaxation.*

^{11}B NMR (128 MHz, CDCl$_3$): δ 27.62 ppm.

5,5-Dimethyl-2-(*m*-tolyl)-1,3,2-dioxaborinane 14k[47,53]

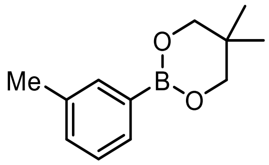

14k

Prepared according to *General Procedure A*. Data presented as: (a) amount of 3-methylbenzeneboronic acid, (b) volume of DCM, (c) amount of neopentyl glycol, (d) amount of $MgSO_4$, and (e) yield.

(a) 272 mg, 2.0 mmol, 1 eq., (b) 5 mL, (c) 229 mg, 2.2 mmol, 1.1 eq., (d) 240 mg, 2.0 mmol, 1 eq., and (e) 323 mg, 1.58 mmol, 79%.

Melting Point : 91 – 93 °C (Lit.: 94 – 95 °C).[47]
FTIR (neat): 2954, 1581, 1477, 1423 1338, 1249, 1128, 813, 651 cm^{-1}.
^{1}H NMR (400 MHz, $CDCl_3$): δ 7.66 - 7.57 (m, 2H, Ar$\underline{H}$), 7.31 - 7.21 (m, 2H, Ar$\underline{H}$), 3.78 (s, 4H, 2 × C$\underline{H}_2$), 2.36 (s, 3H, ArC$\underline{H}_3$), 1.03 (s, 6H, 2 × C$\underline{H}_3$) ppm.
^{13}C NMR (101 MHz, $CDCl_3$): δ 137.0, 134.6, 131.6, 131.0, 127.7, 72.5, 32.0, 22.0, 21.5 ppm. *To note: the carbon directly attached to the boron atom was not observed, likely due to quadrupolar relaxation.*
^{11}B NMR (128 MHz, $CDCl_3$): δ 27.04 ppm.

2-(3-Methoxyphenyl)-5,5-dimethyl-1,3,2-dioxaborinane 14l[42,52,54]

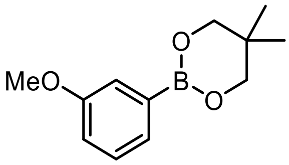

14l

Prepared according to *General Procedure A*. Data presented as: (a) amount of 3-methoxybenzeneboronic acid, (b) volume of DCM, (c) amount of neopentyl glycol, (d) amount of $MgSO_4$, and (e) yield.

(a) 304 mg, 2.0 mmol, 1 eq., (b) 5 mL, (c) 229 mg, 2.2 mmol, 1.1 eq., (d) 240 mg, 2.0 mmol, 1 eq., and (e) 398 mg, 1.81 mmol, 91%.

Melting Point: 69 – 71 °C (Lit.: 69 – 72 °C).[54]
FTIR (neat): 2941, 1573, 1479, 1409, 1296, 1120, 1085, 1033, 790, 673 cm^{-1}.
^{1}H NMR (400 MHz, $CDCl_3$): δ 7.40 (dt, *J* = 7.3 Hz, $^4J_{H-H}$ = 1.1 Hz, 1H, Ar<u>H</u>), 7.34 (d, $^4J_{H-H}$ = 2.8 Hz, 1H, Ar<u>H</u>), 7.28 (t, *J* = 7.9 Hz, 1H, Ar<u>H</u>), 6.98 (ddd, *J* = 8.1 Hz, $^4J_{H-H}$ = 2.8, 1.1 Hz, 1H, Ar<u>H</u>), 3.83 (s, 3H, $OC\underline{H}_3$), 3.77 (s, 4H, 2 × $C\underline{H}_2$), 1.03 (s, 6H, 2 × $C\underline{H}_3$) ppm.
^{13}C NMR (101 MHz, $CDCl_3$): δ 159.2, 128.9, 126.4, 118.1, 117.4, 72.5, 55.3, 32.0, 22.1 ppm. *To note: the carbon directly attached to the boron atom was not observed, likely due to quadrupolar relaxation.*
^{11}B NMR (128 MHz, $CDCl_3$): δ 26.98 ppm.

1.8.2 Hydrogen isotope exchange of aryl-BNeos (Manuscript, Scheme 4)

All reactions were run as described in *General Procedure D.* Each experiment was carried out over three runs and the deuterium incorporation was calculated based on analysis of the ^{1}H NMR spectra of pure products following silica gel chromatography. Representative reaction yields are also provided for selected substrates.

2-(4-Methoxyphenyl)-5,5-dimethyl-1,3,2-dioxaborinane *d*-14a

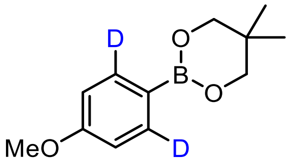

***d*-14a**

^{1}H NMR (400 MHz, CDCl$_3$): δ 7.75 (d, *J* = 8.7 Hz, 2H, ArH), 6.89 (d, *J* = 8.7 Hz, 2H, ArH), 3.82 (s, 3H, OCH$_3$), 3.75 (s, 4H, 2 × CH$_2$), 1.02 (s, 6H, 2 × CH$_3$) ppm.

Deuterium incorporation expected at δ 7.75 ppm and measured against the signal at δ 6.89 ppm.

Substrate mass (mg)	Solvent	Temp. (°C)	Time (h)	Run 1 (%)	Run 2 (%)	Run 3 (%)	Avg (%)
**23.5**	PhF	r.t.	16	90	90.5	89.5	90
**23.5**	Toluene	80	1	95.5	95.5	95.5	95.5
		**Reaction yield (mg, %)**		21.2, 89	21.7, 91	21.4, 91	21.4, 90

5,5-Dimethyl-2-(*p*-tolyl)-1,3,2-dioxaborinane *d*-14b

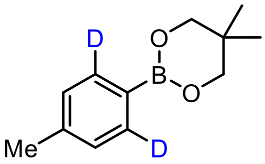

***d*-14b**

^{1}H NMR (400 MHz, CDCl$_3$): δ 7.70 (d, *J* = 8.0 Hz, 2H, ArH), 7.17 (d, *J* = 8.0 Hz, 2H, ArH), 3.76 (s, 4H, 2 × CH$_2$), 2.36 (s, 3H, ArCH$_3$), 1.02 (s, 6H, 2 × CH$_3$) ppm.

Deuterium incorporation expected at δ 7.70 ppm and measured against the signal at δ 7.17 ppm.

Substrate mass (mg)	Solvent	Temp. (°C)	Time (h)	Run 1 (%)	Run 2 (%)	Run 3 (%)	Avg (%)
**21.8**	PhF	r.t.	16	87.5	89.5	88.5	89
**21.8**	Toluene	80	4	95	94.5	95	95
		Reaction yield (mg, %)		19.8, 90	20.0, 90	20.2, 91	20.0, 90

2-([1,1'-Biphenyl]-4-yl)-5,5-dimethyl-1,3,2-dioxaborinane *d*-14c

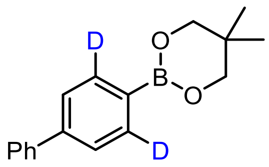

***d*-14c**

^{1}H NMR (400 MHz, CDCl$_3$): δ 8.06 (d, *J* = 8.2 Hz, 2H, ArH), 7.78 - 7.72 (m, 4H, ArH), 7.55 (t, *J* = 7.5 Hz, 2H, ArH), 7.45 (t, *J* = 7.4 Hz, 1H, ArH), 3.88 (s, 4H, 2 × CH_2), 1.13 (s, 6H, 2 × CH_3) ppm.

Deuterium incorporation expected at δ 8.06 ppm and measured against the signal at 7.45 ppm.

Substrate mass (mg)	Solvent	Temp. (°C)	Time (h)	Run 1 (%)	Run 2 (%)	Run 3 (%)	Avg (%)
**28.5**	PhF	r.t.	16	87	88	88	88

Note: 12%D avg. observed on the methylene and methyl positions.

2-(4-(*Tert*-butyl)phenyl)-5,5-dimethyl-1,3,2-dioxaborinane *d*-14d

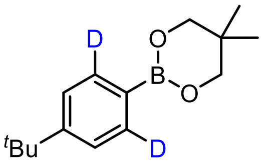

***d*-14d**

^{1}H NMR (400 MHz, CDCl$_3$): δ 7.81 (d, *J* = 8.3 Hz, 2H, ArH), 7.44 (d, *J* = 8.3 Hz, 2H, ArH), 3.80 (s, 4H, 2 × CH$_2$), 1.38 (s, 9H, C(CH$_3$)$_3$), 1.05 (s, 6H, 2 × CH$_3$) ppm.

Deuterium incorporation expected at δ 7.81 ppm and measured against the signal at δ 7.44 ppm.

Substrate mass (mg)	Solvent	Temp. (°C)	Time (h)	Run 1 (%)	Run 2 (%)	Run 3 (%)	Avg (%)
**26.3**	PhF	r.t.	16	70	74	71	72

2-(4-Chlorophenyl)-5,5-dimethyl-1,3,2-dioxaborinane *d*-14e

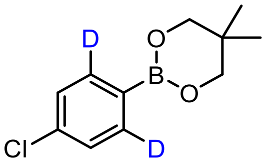

***d*-14e**

^{1}H NMR (400 MHz, CDCl$_3$): δ 7.72 (d, *J* = 8.3 Hz, 2H, ArH), 7.32 (d, *J* = 8.4 Hz, 2H, ArH), 3.76 (s, 4H, 2 × CH$_2$), 1.02 (s, 6H, 2 × CH$_3$) ppm.

Deuterium incorporation expected at δ 7.72 ppm and measured against the signal at δ 7.32 ppm.

Substrate mass (mg)	Solvent	Temp. (°C)	Time (h)	Run 1 (%)	Run 2 (%)	Run 3 (%)	Avg (%)
**24.0**	PhF	50	16	91	91	94	92
**24.0**	Toluene	80	4	91.5	91	91.5	91
		**Reaction yield (mg, %)**		23.8, 98	23.5, 97	23.2, 96	23.5, 97

2-(4-Fluorophenyl)-5,5-dimethyl-1,3,2-dioxaborinane *d*-14f

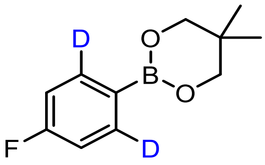

***d*-14f**

^{1}H NMR (400 MHz, CDCl$_3$): δ 7.85 (dd, *J* = 8.5 Hz, $^{4}J_{H\text{-}F}$ = 6.3 Hz, 2H, ArH), 7.07 (dd, $^{3}J_{H\text{-}F}$ = 9.5 Hz, *J* = 8.7 Hz, 2H, ArH), 3.78 (s, 4H, 2 × CH$_2$), 1.03 (s, 6H, 2 × CH$_3$) ppm.

Deuterium incorporation expected at δ 7.85 ppm and measured against the signal at δ 7.07 ppm.

Substrate mass (mg)	Solvent	Temp. (°C)	Time (h)	Run 1 (%)	Run 2 (%)	Run 3 (%)	Avg (%)
**22.3**	PhF	50	16	90.5	89.5	88.5	90

2-(4-Bromophenyl)-5,5-dimethyl-1,3,2-dioxaborinane *d*-14g

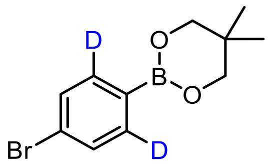

***d*-14g**

^{1}H NMR (400 MHz, CDCl$_3$): δ 7.65 (d, *J* = 8.3 Hz, 2H, ArH), 7.48 (d, *J* = 8.4 Hz, 2H, ArH), 3.76 (s, 4H, 2 × CH$_2$), 1.02 (s, 6H, 2 × CH$_3$) ppm.

Deuterium incorporation expected at δ 7.65 ppm and measured against the signal at δ 7.48 ppm.

Substrate mass (mg)	Solvent	Temp. (°C)	Time (h)	Run 1 (%)	Run 2 (%)	Run 3 (%)	Avg (%)
**28.8**	PhF	50	16	76	74.5	73.5	75

5,5-Dimethyl-2-(4-(trifluoromethyl)phenyl)-1,3,2-dioxaborinane *d*-14h

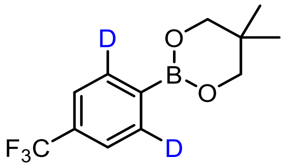

***d*-14h**

^{1}H NMR (400 MHz, CDCl$_3$): δ 7.92 (d, *J* = 8.3 Hz, 2H, ArH), 7.62 (d, *J* = 8.3 Hz, 2H, ArH), 3.81 (s, 4H, 2 × CH$_2$), 1.06 (s, 6H, 2 × CH$_3$) ppm.

Deuterium incorporation expected at δ 7.92 ppm and measured against the signal at δ 7.62 ppm.

Substrate mass (mg)	Solvent	Temp. (°C)	Time (h)	Run 1 (%)	Run 2 (%)	Run 3 (%)	Avg (%)
**27.6**	PhF	80	16	79.5	78.5	77.5	79

2-(4-(Benzyloxy)phenyl)-5,5-dimethyl-1,3,2-dioxaborinane *d*-14i

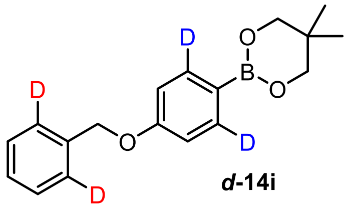

***d*-14i**

^{1}H NMR (400 MHz, CDCl$_3$): δ 7.75 (d, *J* = 8.7 Hz, 2H, ArH), 7.46 - 7.42 (m, 2H, ArH), 7.38 (ddd, *J* = 8.1, 6.9 Hz, $^4J_{H-H}$ = 1.0 Hz, 2H, ArH), 7.34 - 7.28 (m, 1H, ArH), 6.96 (d, *J* = 8.8 Hz, 2H, ArH), 5.09 (s, 2H, OCH$_2$), 3.75 (s, 4H, 2 × BOCH$_2$), 1.02 (s, 6H, 2 × CH$_{3,}$) ppm.

Deuterium incorporation expected at δ 7.75 (D) and δ 7.46 - 7.42 (D) ppm, measured against the signal at δ 6.96 ppm.

Substrate mass (mg)	Solvent	Temp. (°C)	Time (h)	Run 1 (%)		Run 2 (%)		Run 3 (%)		Avg (%)	
				D	D	D	D	D	D	D	D
**31.7**	PhF	r.t.	16	94.5	46	92.5	42	94	46	94	45

Note: 14%D avg. observed on the methylene, and 12%D avg. observed on the methyl groups.

5,5-Dimethyl-2-(*o*-tolyl)-1,3,2-dioxaborinane *d*-14j

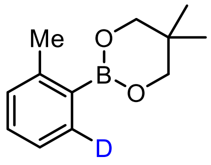

***d*-14j**

^{1}H NMR (400 MHz, $CDCl_3$): δ 7.78 (d, J = 7.3 Hz, 1H, ArH), 7.32 (td, J = 7.6, 1.6 Hz, 1H, ArH), 7.19 (app. t, 2H, ArH), 3.81 (s, 4H, 2 × CH_2), 2.57 (s, 3H, ArCH_3), 1.07 (s, 6H, 2 × CH_3) ppm.

Deuterium incorporation expected at δ 7.78 ppm and measured against the signal at δ 7.19 ppm.

Substrate mass (mg)	Solvent	Temp. (°C)	Time (h)	Run 1 (%)	Run 2 (%)	Run 3 (%)	Avg (%)
**21.8**	PhF	r.t.	16	96	96	95	96

5,5-Dimethyl-2-(*m*-tolyl)-1,3,2-dioxaborinane *d*-14k

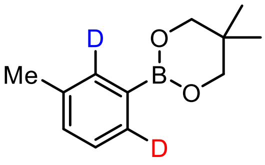

***d*-14k**

^{1}H NMR (400 MHz, $CDCl_3$): δ 7.66 - 7.57 (m, 2H, ArH), 7.31 - 7.21 (m, 2H, ArH), 3.78 (s, 4H, 2 × CH_2), 2.36 (s, 3H, ArCH_3), 1.03 (s, 6H, 2 × CH_3) ppm.

Deuterium incorporation expected at δ 7.66 - 7.57 ppm and measured against the signal at δ 2.36 ppm.

Substrate mass (mg)	Solvent	Temp. (°C)	Time (h)	Run 1 (%)	Run 2 (%)	Run 3 (%)	Avg (%)
**21.8**	PhF	r.t.	16	50	45	46	47

To note: due to an overlap of the anticipated labelled peaks, incorporation is reported as an average over both ortho-sites.

2-(3-Methoxyphenyl)-5,5-dimethyl-1,3,2-dioxaborinane *d*-14l

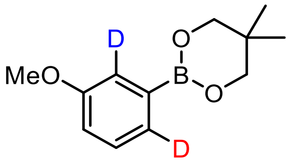

***d*-14l**

^{1}H NMR (400 MHz, CDCl$_3$): δ 7.40 (dt, *J* = 7.3 Hz, $^{4}J_{H\text{-}H}$ = 1.1 Hz, 1H, ArH), 7.34 (d, $^{4}J_{H\text{-}H}$ = 2.8 Hz, 1H, ArH), 7.28 (t, *J* = 7.9 Hz, 1H, ArH), 6.98 (ddd, *J* = 8.1 Hz, $^{4}J_{H\text{-}H}$ = 2.8, 1.1 Hz, 1H, ArH), 3.83 (s, 3H, OCH$_3$), 3.77 (s, 4H, 2 × CH$_2$), 1.03 (s, 6H, 2 × CH$_3$) ppm.

Deuterium incorporation expected at δ 7.40 (D) and δ 7.34 (D) ppm, measured against the signal at 6.98 ppm.

Substrate mass (mg)	Solvent	Temp. (°C)	Time (h)	Run 1 (%)		Run 2 (%)		Run 3 (%)		Avg (%)	
				D	D	D	D	D	D	D	D
**23.5**	PhF	r.t.	16	87	89	85	88	86	89	86	89

1.9 Application of Labelled Boronates

1.9.1 Large scale synthesis of *d*-13b (Manuscript, Scheme 5)

2-(4-Methoxyphenyl)-4,4,5,5-tetramethyl-1,3,2-dioxaborolane *d*-13b

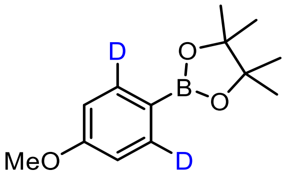

***d*-13b**

Reactions were performed using a Radley's 12 plus reaction station. Each of the carousel tubes were dried overnight in an over at 180 °C and allowed to cool under vacuum before use. Each of the nine tubes were charged with 2-(4-methoxyphenyl)-4,4,5,5-tetramethyl-1,3,2-dioxaborolane **13b** (75.5 mg, 0.3225 mmol) and the pre-catalyst **2** (26.9 mg, 0.01613 mmol, 5 mol%). To each tube was added toluene (3.2 mL, 0.1 M) and the solutions were cooled to -78 °C using dry ice/acetone. The atmosphere was exchanged with three vacuum/D_2 cycles introduced *via* balloon, the tubes were sealed, and then immediately placed in a heating block pre-heated to 80 °C. The reactions were stirred for the 4 h, after which MeCN (0.5 mL) was added to the tubes to deactivate the catalyst. The reaction mixtures were transferred to a vial and concentrated *in vacuo*. The residue was then purified by passing through a short silica gel column with 20% Et_2O/petroleum ether to afford the deuterated boronate product. Deuterium incorporation was determined by ^1H NMR spectroscopy of the products obtained with the integrals of the labelled positions measured against a peak corresponding to a position where labeling was not expected. The level of deuteration was then calculated using Equation **1** (*cf.* Section 1.2) and reported in the table below (entries 1 to 9). The deuterated products were then dissolved in DCM, combined and concentrated *in vacuo* to afford deuterated 2-(4-methoxyphenyl)-4,4,5,5-tetramethyl-1,3,2-dioxaborolane ***d*-13b** (591 mg, 77.5%D, 87% yield) as a white solid.

The labelled material, after this one deuteration cycle, was subjected to a second cycle of deuteration as follows:

Reactions were performed using a Radley's 12 plus reaction station. Each of the carousel tubes were dried overnight in an over at 180 °C and allowed to cool under vacuum before use. Each of the seven tubes were charged with 2-(4-methoxyphenyl)-4,4,5,5-tetramethyl-1,3,2-dioxaborolane ***d*-13b** (75.5 mg, 0.3225 mmol, 77.5%D) and the pre-catalyst **2** (26.9 mg, 0.01613 mmol, 5 mol%). To each tube was added toluene (3.2 mL, 0.1 M) and the solutions were cooled to -78 °C using dry ice/acetone. The atmosphere was exchanged with three vacuum/D_2 cycles introduced *via* balloon, the tubes were sealed, and then immediately placed in a heating block pre-heated to 80 °C. The reactions were stirred for the 16 h, after which time MeCN (0.5 mL) was added to the tubes to deactivate the catalyst. The reaction mixtures were transferred to a vial and concentrated *in vacuo*. The residue was then purified by passing through a short silica gel column with 20% Et_2O/petroleum ether to afford the deuterated boronate product. Deuterium incorporation was determined by ^{1}H NMR spectroscopy of the products obtained with the integrals of the labelled positions measured against a peak corresponding to a position where labeling was not expected. The level of deuteration was then calculated using Equation **1** (*cf.* Section 1.2) and reported in the table below (entries 10 to 16). The deuterated products were then dissolved in DCM, combined and concentrated *in vacuo* to afford deuterated 2-(4-methoxyphenyl)-4,4,5,5-tetramethyl-1,3,2-dioxaborolane ***d*-13b** (431 mg, 86%D, 81% yield) as a white solid.

^{1}H NMR (400 MHz, $CDCl_3$): δ 7.75 (d, *J* = 8.7 Hz, 2H, Ar$\underline{H}$), 6.90 (d, *J* = 8.7 Hz, 2H, Ar$\underline{H}$), 3.83 (s, 3H, OC$\underline{H}_3$), 1.33 (s, 12H, 4 × C$\underline{H}_3$) ppm.

Labeling expected against signal at 7.75 ppm and measured against the signal at 6.90 ppm.

Entry	Time (h)	Starting %D	Final %D	Avg %D	Combined Mass (mg)	Combined Yield (%)
**1**	4	0	76.5	77.5	591	87
**2**	4	0	76.5			
**3**	4	0	76.5			
**4**	4	0	77			
**5**	4	0	82.5			
**6**	4	0	77.5			
**7**	4	0	77.5			
**8**	4	0	76.5			
**9**	4	0	76			
**10**	16	77.5	86	86	431	81
**11**	16	77.5	87.5			
**12**	16	77.5	84			
**13**	16	77.5	87.5			
**14**	16	77.5	87.5			
**15**	16	77.5	85.5			
**16**	16	77.5	87			

1.9.2 Chan-Lam coupling of ***d*-13b** (Manuscript, Scheme 5)

1-(4-Methoxyphenyl-2,6-*d*$_2$)piperidine *d*-16[55]

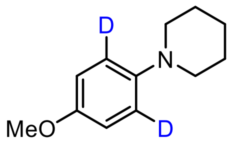

***d*-16**

Prepared according to an adapted literature procedure.[55] A solution of deuterated 2-(4-methoxyphenyl)-4,4,5,5-tetramethyl-1,3,2-dioxaborolane ***d*-13b** (86%D, 71 mg, 0.3 mmol, 1 eq.), piperidine **15** (0.06 mL, 51 mg, 1.2 mmol, 2 eq.), $Cu(OAc)_2$ (54 mg, 0.3 mmol, 1 eq.), Et_3N (0.06

mL, 89 mg, 0.6 mmol, 2 eq.), and powdered 3 Å molecular sieves (100 mg, activated by flame drying and stored in oven at 180 °C until use) in MeCN (0.6 mL) was sealed in a flame-dried microwave vial under air and stirred at 80 °C for 24 h. The reaction mixture was then allowed to cool to room temperature and filtered through celite. The filtrate was concentrated *in vacuo* and the residue was purified by column chromatography, eluting with 0-5% EtOAc/petroleum ether with 1% Et_3N modifier, to provide 1-(4-methoxyphenyl-2,6-d_2)piperidine ***d*-16** (86%D, 26 mg, 0.135 mmol, 45%) as a colourless oil.

FTIR (neat): 2980, 2930, 1508, 1464, 1238, 1180, 918, 820 cm^{-1}.

^{1}H NMR (400 MHz, $CDCl_3$): δ 6.92 (dt, J = 9.1 Hz, $^4J_{H-H}$ = 2.4 Hz, 0.29H, ArH), 6.83 (dt, J = 9.1 Hz, $^4J_{H-H}$ = 2.4 Hz, 2H, ArH), 3.77 (s, 3H, OCH_3), 3.03 (t, J = 5.6 Hz, 4H, 2 × NCH_2CH_2), 1.69 – 1.77 (m, 4H, 2 × CH_2), 1.51 – 1.59 (m, 2H, CH_2),ppm.

^{13}C NMR (101 MHz, $CDCl_3$): δ 153.7, 146.9, 118.6 (t, $^2J_{C-D}$ = 23.9 Hz), 114.4, 55.7, 52.4, 26.3, 24.3 ppm.

Deuterium incorporation expected against signal at δ 6.92 ppm and measured against the signal at δ 6.83 ppm.

1.9.3 Suzuki-Miyaura cross-coupling of ***d*-13b** (Manuscript, Scheme 5)

4-Methoxy-4'-nitro-1,1'-biphenyl-2,6-d_2 *d*-18[35,56]

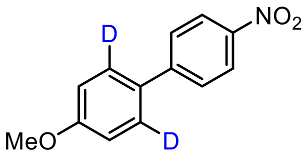

***d*-18**

Prepared according to literature procedure.[35] To a flame-dried 50 mL round bottomed flask was added 4-iodonitrobenzene **17** (249 mg, 1.0 mmol, 2 eq.) and DMF (5 mL). To this solution, was added deuterated 2-(4-methoxyphenyl)-4,4,5,5-tetramethyl-1,3,2-dioxaborolane ***d*-13b** (86%D, 119 mg, 0.5 mmol, 1 eq.), $Pd(PPh_3)_4$ (28.9 mg, 0.025 mmol, 5 mol%), and, subsequently, K_2CO_3

(138 mg, 1.0 mmol, 2 eq.). The reaction mixture was heated to 90 °C and stirred at this temperature for 24 h. After this time, the reaction mixture was cooled to room temperature, diluted with water, and extracted with Et_2O. The organics were combined, dried over Na_2SO_4, filtered, and concentrated *in vacuo*. The crude material was then purified by column chromatography, eluting with 0-10% Et_2O/petroleum ether, to deliver 4-methoxy-4'-nitro-1,1'-biphenyl-2,6-d_2 ***d*-18** (97 mg, 0.42 mmol, 84%, 86%D) as a yellow solid.

Melting point: 104 – 106 °C (Lit.: 107 – 109 °C).[56]

FTIR (neat): 2968, 2930, 2913, 2835, 1593, 1574, 1522, 1341, 1250 cm^{-1}.

^{1}H NMR (400 MHz, CDCl$_3$): δ 8.27 (dt, J = 8.9 Hz, $^4J_{H-H}$ = 2.1 Hz, 2H, Ar<u>H</u>), 7.69 (dt, J = 9.0 Hz, $^4J_{H-H}$ = 2.1 Hz, 2H, Ar<u>H</u>), 7.58 (dt, J = 8.9 Hz, $^4J_{H-H}$ = 2.1 Hz, 0.28H, Ar<u>H</u>), 7.02 (dt, J = 9.0 Hz, $^4J_{H-H}$ = 2.1 Hz, 2H, Ar<u>H</u>), 3.88 (s, 3H, OC<u>H</u>$_3$) ppm.

^{13}C NMR (101 MHz, CDCl$_3$): δ 160.6, 147.3, 146.7, 131.0, 128.4 (t, $^2J_{C-D}$ = 23.8 Hz), 127.2, 124.3, 114.6, 55.6 ppm.

HRMS *m/z* (ESI): Calc. for $C_{13}H_9D_2NO_3$ [M$^+$]: 231.98645. Found 231.0859.

Deuterium incorporation expected against signal at δ 7.58 ppm and measured against signal at δ 7.02 ppm.

1.9.4 Synthesis of a labelled drug molecule (Manuscript, Scheme 6)

***N*-(1-(1*H*-Benzo[*d*][1,2,3]triazol-1-yl)ethyl)-4-bromoaniline S13**[57]

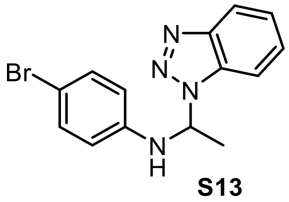

S13

Prepared according to a modified literature procedure.[57] To a flame-dried 3-neck round bottomed flask under argon was suspended benzotriazole (6.92 g, 58.1 mmol, 1 eq.) in toluene (100 mL). 4-Bromoaniline (10 g, 58.1 mmol, 1 eq.) in toluene (15 mL) was added to the suspension *via* syringe. Acetaldehyde (3.3 mL, 2.56 g, 58.7 mmol, 1.01 eq.) in toluene (10 mL) was added

dropwise *via* a dropping funnel. The solution became progressively more homogeneous during the addition and, after the addition was complete, the reaction was allowed to stir at room temperature under argon for 16 h, after which time a precipitate had formed. The reaction mixture was then filtered to give *N*-(1-(1*H*-benzo[*d*][1,2,3]triazol-1-yl)ethyl)-4-bromoaniline **S13** (14.1 g, 44.4 mmol, 76%) as an off white solid, which was used directly in the next step without any further purification.

Melting point: 94 – 96 °C.

FTIR (neat): 3318, 3102, 3046, 2984, 1595, 1524, 1487, 1452, 1385, 1310, 1290, 1179, 1157, 1076, 812, 745, 704 cm^{-1}.

^{1}H NMR (400 MHz, DMSO): δ 8.10 (d, *J* = 8.4 Hz, 1H, ArH (benzotriazole)), 8.00 (d, *J* = 8.4 Hz, 1H, ArH (benzotriazole)), 7.54 - 7.46 (m, 2H, ArH (benzotriazole)), 7.38 - 7.32 (m, 1H, CH), 7.16 (d, *J* = 8.9 Hz, 2H, ArH), 6.64 (d, *J* = 8.9 Hz, 2H, ArH), 1.86 (d, *J* = 6.4 Hz, 3H, CH_3) ppm. *To note: additional peaks were observed in the crude ^{1}H NMR but were not assigned.*

^{13}C NMR (101 MHz, DMSO): δ 145.7, 144.6, 131.5, 130.7, 127.1, 123.9, 119.3, 114.8, 111.2, 108.8, 66.0, 21.1 ppm. To *note: additional peaks were observed in the crude ^{13}C NMR but were not assigned.*

HRMS *m/z* (ESI): Calc. for $C_{14}H_{13}{}^{79}BrN_4$ [M^+]: 316.03236. Found 316.0339.

***N*-((2*S**,4*R**)-6-Bromo-2-methyl-1,2,3,4-tetrahydroquinolin-4-yl)formamide S14**[57]

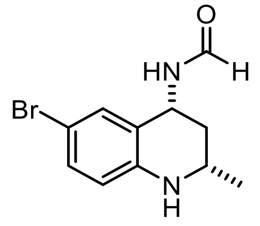

S14

Prepared according to literature procedure.[57] To a flame-dried 3-neck round bottomed flask under argon was added *N*-vinylformamide (4.42 mL, 4.48 g, 63 mmol, 2 eq.) and dry THF (26.6 mL), and the solution was cooled to -5 °C. BF•OEt_2 (15.6 mL, 17.9 g, 126 mmol, 4 eq.) was added dropwise *via* syringe pump with the temperature carefully monitored. The resulting mixture was stirred for 15 min before *N*-(1-(1*H*-benzo[*d*][1,2,3]triazol-1-yl)ethyl)-4-bromoaniline **S13** (10 g,

31.5 mmol, 1 eq.) was added as a solution in THF (67 mL) *via* dropping funnel. The reaction mixture was stirred at -5 °C for a further 2 h. After this time, the reaction mixture was poured slowly into sat. aqueous $NaHCO_3$ solution (350 mL) and extracted with EtOAc (150 mL). The organic layer was further extracted with water and brine, respectively, before being dried over Na_2SO_4, filtered, and concentrated *in vacuo*. The crude mixture was triturated with Et_2O whereby the desired product became insoluble; the solid was collected and dried under vacuum to give *N*-((2*S**,4*R**)-6-bromo-2-methyl-1,2,3,4-tetrahydroquinolin-4-yl)formamide **S14** (5.13 g, 19.1 mmol, 61%) as an off white solid.

To note: rotamers were observed by NMR; the data for the major rotameric product has been reported.

Melting point: 184 – 186 °C.
FTIR (neat): 3395, 3244, 3959, 2864, 1647, 1597, 1541, 1489, 1381, 1298, 1236, 815, 738 cm^{-1}.
^{1}H NMR (400 MHz, DMSO): δ 8.38 (d, *J* = 8.8 Hz, 1H, N<u>H</u>), 8.21 (s, 1H, C(O)<u>H</u>), 7.05 (dd, *J* = 8.5, 2.3 Hz, 1H, Ar<u>H</u>), 6.98 (s, 1H, Ar<u>H</u>), 6.45 (d, *J* = 8.6 Hz, 1H, Ar<u>H</u>), 5.94 (s, 1H, N<u>H</u>), 5.16 - 5.05 (m, 1H, C<u>H</u>), 3.42 - 3.39 (m, 1H, C<u>H</u>), 2.01 - 1.90 (m, 1H, C<u>H</u>$_2$), 1.40 (app. q, *J* = 11.8 Hz, 1H, C<u>H</u>$_2$), 1.14 (d, *J* = 6.3 Hz, 3H, C<u>H</u>$_3$) ppm.
^{13}C NMR (101 MHz, DMSO): δ 161.1, 144.9, 130.0, 128.6, 122.9, 115.3, 105.9, 45.8, 43.7, 36.5, 21.6 ppm.
HRMS *m/z* (ESI): Calc. for $C_{11}H_{14}{}^{79}BrN_2O$ [M+H]: 269.02840. Found 269.0278.

N-((2*S**,4*R**)-1-Acetyl-6-bromo-2-methyl-1,2,3,4-tetrahydroquinolin-4-yl)formamide S15[57]

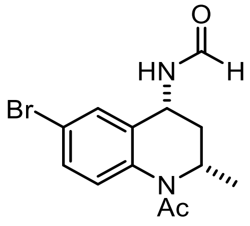

S15

Prepared according to literature procedure.[57] To a flame-dried round bottomed flask under argon was added *N*-((2*S**,4*R**)-6-bromo-2-methyl-1,2,3,4-tetrahydroquinolin-4-yl)formamide

S14 (4.8 g, 17.8 mmol, 1 eq.) in DCM (71 mL), followed by pyridine (25 mL). The mixture was cooled to 0 °C and acetyl chloride (1.42 mL 1.56 g, 19.9 mmol, 1.12 eq.) was added dropwise. The reaction mixture was stirred at 0 °C for 2 h before being poured into a mixture of conc. HCl (32 mL) and ice. The product was extracted with DCM and washed once with brine. The organics were dried over Na_2SO_4, filtered, and concentrated *in vacuo* to give *N*-((2*S**,4*R**)-1-acetyl-6-bromo-2-methyl-1,2,3,4-tetrahydroquinolin-4-yl)formamide **S15** (4.8 g, 15.4 mmol, 87%) as a white solid without any further purification required.

To note: Rotamers were observed by NMR; the data for the major rotameric product has been reported. Variable temperature NMR was run to confirm the rotameric structures.

Melting point: 162 – 164 °C.

FTIR (neat): 3262, 2980, 2868, 1630, 1528, 1477, 1369, 1323, 1248, 1143, 1082, 964, 822 cm^{-1}.

^{1}H NMR (400 MHz, DMSO): δ 8.53 (d, *J* = 8.4 Hz, 1H, C<u>H</u>O), 8.26 (s, 1H, Ar<u>H</u>), 7.48 (dd, *J* = 8.4 Hz, $^4J_{H-H}$ = 2.3 Hz, 1H, Ar<u>H</u>), 7.34 - 7.28 (m, 2H, Ar<u>H</u> + N<u>H</u>), 4.76 - 4.60 (m, 2H, 2 x C<u>H</u>), 2.45 (ddd, $^2J_{H-H}$ = 12.8 Hz, *J* = 8.3, 4.7 Hz, 1H, C<u>H</u>$_2$), 2.06 (s, 3H, C(O)C<u>H</u>$_3$), 1.25 (td, $^2J_{H-H}$ = 12.4 Hz, *J* = 8.8 Hz, 1H, C<u>H</u>$_2$), 1.04 (d, *J* = 6.3 Hz, 3H, C<u>H</u>$_3$) ppm.

^{13}C NMR (101 MHz, $CDCl_3$): δ 169.3, 160.9, 137.4, 135.6, 130.7, 127.8, 126.3, 119.7, 47.1, 44.1, 40.5, 23.0, 21.3 ppm.

HRMS *m/z* (ESI): Calc. for $C_{13}H_{15}{}^{79}BrN_2O_2$ [M^+]: 310.03169. Found 310.0318.

1-((2*S,4*R**)-4-Amino-6-bromo-2-methyl-3,4-dihydroquinolin-1(2*H*)-yl)ethan-1-one 19**[57]

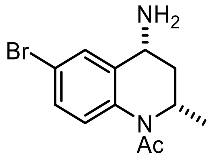

19

Prepared according to a modified literature procedure.[57] To a suspension of *N*-((2*S**,4*R**)-1-acetyl-6-bromo-2-methyl-1,2,3,4-tetrahydroquinolin-4-yl)formamide **S15** (3.0 g, 9.64 mmol, 1 eq.) in MeOH (32 mL) was added 6 M HCl (3.2 mL, 19.3 mmol 2 eq.). The resulting mixture was stirred at reflux for 1 h. After this time, the reaction mixture was cooled to r.t. and basified to pH 9 with 2 M NaOH (aq.). The MeOH was evaporated from the mixture and the organic material

was extracted with EtOAc. The combined organics were washed once with brine, dried over Na_2SO_4, and concentrated *in vacuo* to give 1-((2*S**,4*R**)-4-amino-6-bromo-2-methyl-3,4-dihydroquinolin-1(2*H*)-yl)ethan-1-one **19** (2.7 g, 9.46 mmol, 98%) as a brown oil, which was used without any further purification.

FTIR (neat): 3372, 3302, 2968, 2930, 2866, 2250, 1641, 1475, 1369, 1323, 1194, 1138, 1082, 880, 818 cm^{-1}.

^{1}H NMR (400 MHz, $CDCl_3$): δ 7.65 (dd, $^4J_{H-H}$ = 2.4 Hz, $^5J_{H-H}$ = 1.1 Hz, 1H, ArH), 7.40 (ddd, J = 8.3 Hz, $^4J_{H-H}$ = 2.3 Hz, $^5J_{H-H}$ = 0.7 Hz, 1H, ArH), 6.98 (d, J = 8.3 Hz, 1H, ArH), 4.88 - 4.73 (m, 1H, CH), 3.71 (dd, $^2J_{H-H}$ = 12.3 Hz, J = 4.4 Hz, 1H, CH_2), 2.52 (ddd, $^2J_{H-H}$ = 12.6 Hz, J = 8.5, 4.5 Hz, 1H, CH_2), 2.09 (s, 3H, C(O)CH_3), 1.50 (s, 2H, NH_2), 1.16 - 1.05 (m, 4H, CH_3 + CH) ppm.

^{13}C NMR (101 MHz, $CDCl_3$): δ 169.3, 142.1, 135.3, 129.8, 127.3, 126.1, 119.8, 47.6, 47.4, 44.1, 22.8, 21.4 ppm.

HRMS *m/z* (ESI): Calc. for $C_{12}H_{16}{}^{79}BrN_2O$ [M+H]: 283.04405. Found 283.0429.

2-(4-Chlorophenyl)-4,4,5,5-tetramethyl-1,3,2-dioxaborolane *d*-13f

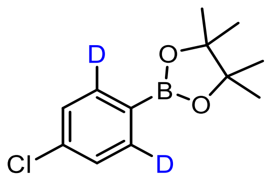

***d*-13f**

Reactions were performed using a Radley's 12 plus reaction station. Each of the carousel tubes were dried overnight in an over at 180 °C and allowed to cool under vacuum before use. Each tube was charged with 2-(4-chlorophenyl)-4,4,5,5-tetramethyl-1,3,2-dioxaborolane **13f** (51.3 mg, 0.215 mmol) and the pre-catalyst **2** (17.8 mg, 0.01075 mmol, 5 mol%). To each tube was added PhF (2.1 mL, 0.1 M) and the solutions were cooled to -78 °C using dry ice/acetone. The atmosphere was exchanged with three vacuum/D_2 cycles introduced *via* balloon, the tubes were sealed, and immediately placed in a heating block pre-heated to 80 °C. The reactions were stirred for 16 h, after which time MeCN was added to deactivate the catalyst. The reaction mixtures were transferred to a vial and concentrated *in vacuo*. The residue was then purified by passing

through a short silica gel column with 20% Et_2O/petroleum ether to afford the deuterated boronate product. Deuterium incorporation was determined by ^{1}H NMR spectroscopy of the products obtained, with the integrals of the anticipated labelled positions measured against a peak corresponding to a position where labeling was not expected. The level of deuteration was then calculated using Equation **1** (*cf.* Section 1.2).

^{1}H NMR (400 MHz, $CDCl_3$): δ 7.73 (d, *J* = 8.3 Hz, 2H, ArH) 7.34 (d, *J* = 8.4 Hz, 2H, ArH), 1.34 (s, 12H, 4 × CH_3) ppm.

Deuterium incorporation expected against signal at δ 7.73 ppm and measured against the signal at δ 7.34 ppm.

Entry	%D	Avg %D	Combined Mass (mg)	Combined Yield (%)
**1**	80	81	306	84
**2**	81.5			
**3**	82			
**4**	81			
**5**	81			
**6**	79.5			
**7**	81			

1-((2*S,4*R**)-6-Bromo-4-((4-chlorophenyl-2,6-*d*$_2$)amino)-2-methyl-3,4-dihydroquinolin-1(2*H*)-yl)ethan-1-one *d*-20**

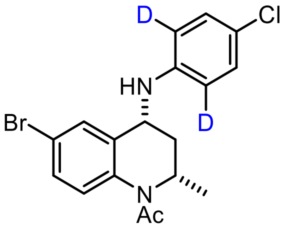

***d*-20**

Prepared according to an adapted literature procedure.[55] A solution of deuterated 2-(4-chlorophenyl)-4,4,5,5-tetramethyl-1,3,2-dioxaborolane ***d*-13f** (81%D, 238 mg, 1.0 mmol, 1 eq.), 1-((2*S**,4*R**)-4-amino-6-bromo-2-methyl-3,4-dihydroquinolin-1(2*H*)-yl)ethan-1-one **19** (566 mg, 2.0 mmol, 2 eq.), $Cu(OAc)_2$ (182 mg, 1.0 mmol, 1 eq.), Et_3N (0.28 mL, 202 mg, 2.0 mmol, 2 eq.), and powdered 3 Å molecular sieves (200 mg, activated by flame drying and stored in oven at 180 °C until use) in MeCN/EtOH (20:1, 3.0 mL) was sealed in a flame-dried microwave vial under air and stirred at 80 °C for 24 h. The reaction mixture was then allowed to cool to room temperature and filtered through celite. The filtrate was concentrated *in vacuo* and the residue was purified by column chromatography, eluting with 0-100% EtOAc in petrol, to give 1-((2*S**,4*R**)-6-bromo-4-((4-chlorophenyl-2,6-*d*$_2$)amino)-2-methyl-3,4-dihydroquinolin-1(2*H*)-yl)ethan-1-one ***d*-20** (89 mg, 0.23 mmol, 23%, 81%D) as a brown oil.

FTIR (neat): 3310 (pinacol and NH), 2976, 2930, 2868, 1636, 1585, 1476, 1445, 1369, 1325, 1144, 1092, 949, 818, 731 cm^{-1}.

^{1}H NMR (400 MHz, $CDCl_3$): δ 7.37 – 7.45 (m, 2H, Ar<u>H</u>), 7.14 (dd, *J* = 8.9, 2.1 Hz, 2H, Ar<u>H</u>), 7.02 (d, *J* = 8.2 Hz, 1H, Ar<u>H</u>), 6.54 (dd, *J* = 8.9, 2.1 Hz, 0.39H, Ar<u>H</u>), 4.86 (bs, 1H, C<u>H</u>), 4.11 (dd, *J* = 11.9, 4.1 Hz, 1H, C<u>H</u>), 3.83 (s, 1H, N<u>H</u>), 2.63 (ddd, $^2J_{H-H}$ = 12.3 Hz, *J* = 8.3, 4.3 Hz, 1H, C<u>H$_2$</u>), 2.17 (s, 3H C(O)C<u>H$_3$</u>), 1.14 (d, *J* = 6.3 Hz, 3H, C<u>H$_3$</u>) ppm. *To note: a significant amount of HBPin and pinacol were observed, which could not be separated from the product. Undesired peaks at δ 1.23 and 1.26 ppm obscure one methylene proton from within the product.*[58,59]

^{13}C NMR (101 MHz, $CDCl_3$): δ 169.4, 145.4, 145.3, 139.7, 135.4, 130.4, 129.4, 129.3, 127.6, 127.2, 122.9, 119.8, 114.1 (t, $^2J_{C-D}$ = 23.7 Hz), 50.0, 41.0, 29.1, 22.9, 21.3 ppm. *To note: a significant amount of HBPin and pinacol were observed, which could not be separated from the product.*

Undesired ^{13}C peaks at δ 24.6 and 82.9 ppm (HBPin), and δ 24.9 and 75.1 ppm (pinacol) denote their presence.[57,58]

HRMS *m/z* (ESI): Calc. for $C_{18}H_{16}{}^{79}Br^{37}ClD_2N_2NaO$ [M+Na]: 417.03088. Found 417.0296.

Deuterium incorporation expected against signal at δ 6.54 ppm and measured against the signal at δ 4.86 ppm.

4,4,5,5-Tetramethyl-2-(*o*-tolyl)-1,3,2-dioxaborolane *d*-13l

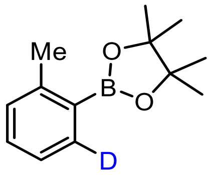

***d*-13l**

Reactions were performed using a Radley's 12 plus reaction station. Each of the carousel tubes were dried overnight in an over at 180 °C and allowed to cool under vacuum before use. Each tube was charged with 2-(2-methylphenyl)-4,4,5,5-tetramethyl-1,3,2-dioxaborolane **13l** (23.3 mg, 0.107 mmol) and the pre-catalyst **2** (8.9 mg, 0.00535 mmol, 5 mol%). To each tube was added toluene (1 mL, 0.1 M) and the solutions were cooled to -78 °C using dry ice/acetone. The atmosphere was exchanged with three vacuum/D_2 cycles introduced *via* balloon, the tubes were sealed, and immediately placed in a heating block pre-heated to 80 °C. The reactions were stirred for 16 h, after which time MeCN was added to deactivate the catalyst. The reaction mixtures were transferred to a vial and concentrated *in vacuo*. The residue was then purified by passing through a short silica gel column with 20% Et_2O/petroleum ether to afford the deuterated boronate product. Deuterium incorporation was determined by ^1H NMR spectroscopy of the products obtained, with the integrals of the anticipated labelled positions measured against a peak corresponding to a position where labeling was not expected. The level of deuteration was then calculated using Equation **1** (*cf.* Section 1.2).

^{1}H NMR (400 MHz, CDCl$_3$): δ 7.76 (dd, J = 7.8 Hz, $^4J_{H-H}$ = 1.6 Hz, 1H, ArH), 7.31 (td, J = 7.5 Hz, $^4J_{H-H}$ = 1.5 Hz, 1H, ArH), 7.19 - 7.13 (m, 2H, ArH), 2.54 (s, 3H, ArCH$_3$), 1.35 (s, 12H, 4 × CH$_3$) ppm.

Deuterium incorporation expected against signal at δ 7.76 ppm and measured against the signal at δ 7.31 ppm.

Entry	%D	Avg %D	Combined Mass (mg)	Combined Yield (%)
**1**	93			
**2**	92			
**3**	90	91	68	57
**4**	87			
**5**	93			

1-((2*S,4*R**)-4-((4-Chlorophenyl-2,6-d_2)amino)-2-methyl-6-(2-methylphenyl-6-*d*)-3,4-dihydroquinolin-1(2*H*)-yl)ethan-1-one *d*-21**

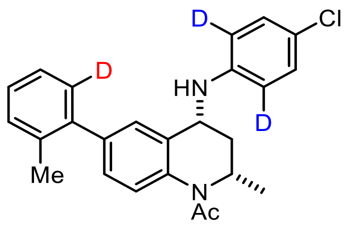

***d*-21**

To a flame-dried 10 mL round bottomed flask fitted, with a condenser, was added 1-((2*S**,4*R**)-6-bromo-4-((4-chlorophenyl-2,6-d_2)amino)-2-methyl-3,4-dihydroquinolin-1(2*H*)-yl)ethan-1-one ***d*-20** (81%D, 79 mg, 0.2 mmol, 1 eq.) as a solution in 1,2-dimethoxyethane (2.0 mL). Labelled 4,4,5,5-tetramethyl-2-(*o*-tolyl)-1,3,2-dioxaborolane ***d*-13l** (91%D, 65 mg, 0.3 mmol, 1.5 eq.), 2 M Na_2CO_3 aq. (0.5 mL 5 eq.), and $Pd(PPh_3)_4$ (23.1 mg, 0.02 mmol, 10 mol%) were added, and the reaction mixture was heated to reflux for 20 h. After this time, the solvent was removed *in vacuo* and the residue was extracted with EtOAc. The combined organics were washed with brine, dried over Na_2SO_4, filtered, and concentrated *in vacuo*. The crude product was then purified by column chromatography, eluting with 0-100% EtOAc in petrol, and delivering the desired product contaminated with pinacol. To remove the pinacol, the material was purified by SCX column eluting with DCM, MeOH, and 2 M NH_3/MeOH. The product was present in the MeOH fractions,

which were concentrated *in vacuo* to deliver 1-((2*S**,4*R**)-4-((4-chlorophenyl-2,6-d_2)amino)-2-methyl-6-(2-methylphenyl-6-*d*)-3,4-dihydroquinolin-1(2*H*)-yl)ethan-1-one ***d*-21** (21 mg, 0.051 mmol, 26%, 81%D, 91%D) as an off white solid. Trace amounts of pinacol were still visible by ^{1}H and ^{13}C NMR analysis.

Melting point: 106 – 108 °C.

FTIR (neat): 3327, 2968, 2929, 2864, 1637, 1585, 1497, 1445, 1369, 1323, 1144, 1020, 891, 760, 648 cm^{-1}.

^{1}H NMR (400 MHz, CD_3CN): δ 7.36 - 7.07 (m, 8.09H, ArH), 6.67 (dt, *J* = 8.9 Hz, $^4J_{H-H}$ = 2.2 Hz, 0.38H, ArH), 4.92 - 4.74 (m, 2H, CH and NH), 4.30 (ddd, *J* = 12.2, 7.9, 4.2 Hz, 1H, CH), 2.65 (ddd, $^2J_{H-H}$ = 12.3 Hz, *J* = 8.3, 4.2 Hz, 1H, CH_2), 2.15 (s, 3H, CH_3), 2.13 (s, 3H, CH_3), 1.27 (td, $^2J_{H-H}$ = 12.3 Hz, *J* = 9.0 Hz, 1H, CH_2), 1.14 (d, *J* = 6.3 Hz, 3H, CH_3) ppm. *To note: a small amount of pinacol was observed, which could not be separated from the product. The ^{1}H peak at δ 1.14 ppm, slightly obscures a peak from the product.*

^{13}C NMR (101 MHz, CD_3CN): δ 170.1, 147.5, 142.1, 139.8, 138.1, 136.7, 136.2, 131.5, 129.9, 129.8, 128.4, 128.3, 127.1, 126.8, 125.4, 122.2, 115.4, 50.4, 48.5, 41.4, 23.3, 21.7, 20.6 ppm.

HRMS *m/z* (ESI): Calc. for $C_{25}H_{22}{}^{37}ClD_3N_2NaO$ [M+Na]: 430.1741. Found 430.1719.

Deuterium incorporation expected against signal at δ 6.67 and δ 7.36 - 7.07 ppm, and measured against the signal at δ 4.30 ppm.

1.10 References

1. W. L. F. Armarego, C. L. L. Chai, "Purification of Laboratory Chemicals" 7th Edition, Elsevier Butterworth-Heinemann, Oxford, 2013.

2. W. J. Kerr, G. J. Knox, M. Reid, T. Tuttle, J. Bergare and R. A. Bragg, "Computationally-Guided Development of a Chelated NHC-P Iridium(I) Complex for the Directed Hydrogen Isotope Exchange of Aryl Sulfones", *ACS Catal.,* 2020, **10**, 11120–11126.

3. Y. Iwai, K. M. Gligorich and M. S. Sigman, "Aerobic Alcohol Oxidation Coupled to Palladium-Catalyzed Alkene Hydroarylation with Boronic Esters", *Angew. Chem. Int. Ed.*, 2008, **47**, 3219–3222.

4. Gaussian 16, Revision C.01, M. J. Frisch, G. W. Trucks, H. B. Schlegel, G. E. Scuseria, M. A. Robb, J. R. Cheeseman, G. Scalmani, V. Barone, G. A. Petersson, H. Nakatsuji, X. Li, M. Caricato, A. V. Marenich, J. Bloino, B. G. Janesko, R. Gomperts, B. Mennucci, H. P. Hratchian, J. V. Ortiz, A. F. Izmaylov, J. L. Sonnenberg, D. Williams-Young, F. Ding, F. Lipparini, F. Egidi, J. Goings, B. Peng, A. Petrone, T. Henderson, D. Ranasinghe, V. G. Zakrzewski, J. Gao, N. Rega, G. Zheng, W. Liang, M. Hada, M. Ehara, K. Toyota, R. Fukuda, J. Hasegawa, M. Ishida, T. Nakajima, Y. Honda, O. Kitao, H. Nakai, T. Vreven, K. Throssell, J. A. Montgomery, Jr., J. E. Peralta, F. Ogliaro, M. J. Bearpark, J. J. Heyd, E. N. Brothers, K. N. Kudin, V. N. Staroverov, T. A. Keith, R. Kobayashi, J. Normand, K. Raghavachari, A. P. Rendell, J. C. Burant, S. S. Iyengar, J. Tomasi, M. Cossi, J. M. Millam, M. Klene, C. Adamo, R. Cammi, J. W. Ochterski, R. L. Martin, K. Morokuma, O. Farkas, J. B. Foresman and D. J. Fox, "Gaussian" Gaussian, Inc., Wallingford CT, 2016.

5. a) Y. Zhao and D. G. Truhlar, "Applications and Validations of the Minnesota Density Functionals", *Chem. Phys. Lett.*, 2011, **502**, 1-13.; b) A. D. McLean and G. S. Chandler, "Contracted Gaussian Basis Sets for Molecular Calculations. I. Second Row Atoms, Z=11-18", *J. Chem. Phys.*, 1980, **72**, 5639-5648; c) R. Krishnan, J. S. Binkley, R. Seeger and J. A. Pople, "Self-Consistent Molecular Orbital Method. XX. A Basis Set for Correlated Wave Functions", *J. Chem. Phys.*, 1980, **72**, 650-654.

6. S. F. Boys and F. Bernardi, "The Calculation of Small Molecular Interactions by the Differences of Separate Total Energies. Some Procedures with Reduced Errors", *Mol. Phys.*, 1970, **19**, 553-566.

7. R. A. Kelly III, H. Clavier, S. Giudice, N. M. Scott, E. D. Stevens, J. Bordner, I. Samardjiev, C. D. Hoff, L. Cavallo and S. Nolan, "Determination of *N*-Heterocyclic Carbene (NHC) Steric and Electronic Parameters Using the [(NHC)Ir(CO)$_2$Cl] System", *Organometallics*, 2008, **27**, 202-210.

8. A. R. Kennedy, W. J. Kerr, R. Moir and M. Reid, "Anion Effects to Deliver Enhanced Iridium Catalysts for Hydrogen Isotope Exchange Processes", *Org. Biomol. Chem.*, 2014, **12**, 7927–7931.

9. M. G. Gardiner, W. A. Herrmann, C.-P. Reisinger, J. Schwarz and M. Spiegler, "Dicationic Chelating *N*-Heterocyclic Carbene Complexes of Palladium: New Catalysts for the Copolymerisation of C_2H_4 and CO", *J. Organomet. Chem.*, 1999, **572**, 239-247.

10. J. Wolf, A. Labande, J.-C. Daran and R. Poli, "Nickel(II) Complexes with Bifunctional Phosphine–Imidazolium Ligands and Their Catalytic Activity in the Kumada–Corriu Coupling Reaction", *J. Organomet. Chem.,* 2006, **691**, 433–443.

11. a) R. Zorzatto, P. T. Mulrainey, M. Reid, T. Tuttle, D. M. Lindsay and W. J. Kerr, "C–H Activation and Hydrogen Isotope Exchange of Aryl Carbamates Using Iridium(I) Complexes Bearing Chelating NHC-Phosphine Ligands", *Chem. Eur. J.*, 2024, **30**, e202403090; b) R. Zorzatto, PhD Thesis, University of Strathclyde, 2018.

12. K. Schröder, S. Enthaler, B. Bitterlich, T. Schulz, A. Spannenberg, M. K. Tse, K. Junge and M. Beller, "Design of and Mechanistic Studies on a Biomimetic Iron-Imidazole Catalyst System for Epoxidation of Olefins with Hydrogen Peroxide", *Chem. Eur. J.*, 2009, **15**, 5471–5481.

13. M. R. Kumar, K. Park and S. Lee, "Synthesis of Amido-*N*-Imidazolium Salts and Their Applications as Ligands in Suzuki-Miyaura Reactions: Coupling of Hetero-Aromatic Halides and the Synthesis of Milrinone and Irbesartan", *Adv. Synth. Catal.*, 2010, **352**, 3255–3266.

14. J. Wolf, A. Labande, M. Natella, J.-C. Daran and R. Poli, "Aryl Grignard Cross-Coupling of Aryl Chlorides Catalyzed by New, Highly Active Phosphine/Imidazolium Nickel(II) Complexes", *J. Mol. Catal. A: Chem.*, 2006, **259**, 205–212.

15. K. Ohmatsu, M. Ito, T. Kunieda and T. Ooi, "Ion-Paired Chiral Ligands for Asymmetric Palladium Catalysis", *Nature Chem.*, 2012, **4**, 473–477.

16. G. Ranjani and R. Nagarajan, "Insight into Copper Catalysis: In Situ Formed Nano Cu_2O in Suzuki–Miyaura Cross-Coupling of Aryl/Indolyl Boronates", *Org. Lett.*, 2017, **19**, 3974–3977.

17. Y.-M. Tian, X.-N. Guo, I. Krummenacher, Z. Wu, J. Nitsch, H. Braunschweig, U. Radius and T. B. Marder, "Visible-Light-Induced Ni-Catalyzed Radical Borylation of Chloroarenes", *J. Am. Chem. Soc.*, 2020, **142**, 18231–18242.

18. W. Srimontree, L. Guo and M. Rueping, "Hydride Transfer Enables the Nickel-Catalyzed *ipso*-Borylation and Silylation of Aldehydes", *Chem. Eur. J.*, 2020, **26**, 423–427.

19. A. D. Chavez, A. M. Evans, N. C. Flanders, R. P. Bisbey, E. Vitaku, L. X. Chen and W. R. Dichtel, "Equilibration of Imine-Linked Polymers to Hexagonal Macrocycles Driven by Self-Assembly", *Chem. Eur. J.*, 2018, **24**, 3989–3993.

20. J. Ratniyom, N. Dechnarong, S. Yotphan and S. Kiatisevi, "Convenient Synthesis of Arylboronates through a Synergistic Pd/Cu-Catalyzed Miyaura Borylation Reaction under Atmospheric Conditions", *Eur. J. Org. Chem.*, 2014, **2014**, 1381–1385.

21. Y.-J. Niu, G.-H. Sui, H.-X. Zheng, X.-H. Shan, L. Tie, J.-L. Fu, J.-P. Qu and Y.-B. Kang, "Competing Dehalogenation versus Borylation of Aryl Iodides and Bromides under Transition-Metal-Free Basic Conditions", *J. Org. Chem.*, 2019, **84**, 10805–10813.

22. Y. Liang, R. Steinbock, L. Yang and L. Ackermann, "Continuous Visible-Light Photoflow Approach for a Manganese-Catalyzed (Het)Arene C−H Arylation", *Angew. Chem. Int. Ed.*, 2018, **57**, 10625–10629.

23. S. Geng, J. Zhang, S. Chen, Z. Liu, X. Zeng, Y. He and Z. Feng, "Development and Mechanistic Studies of Iron-Catalyzed Construction of Csp^2 –B Bonds via C–O Bond Activation", *Org. Lett.*, 2020, **22**, 5582–5588.

24. K. M. M. Huihui, J. A. Caputo, Z. Melchor, A. M. Olivares, A. M. Spiewak, K. A. Johnson, T. A. DiBenedetto, S. Kim, L. K. G. Ackerman and D. J. Weix, "Decarboxylative Cross-Electrophile Coupling of *N*-Hydroxyphthalimide Esters with Aryl Iodides", *J. Am. Chem. Soc.*, 2016, **138**, 5016–5019.

25. J. D. Firth, L. A. Hammarback, T. J. Burden, J. B. Eastwood, J. R. Donald, C. S. Horbaczewskyj, M. T. McRobie, A. Tramaseur, I. P. Clark, M. Towrie, A. Robinson, J. Krieger, J. M. Lynam and I. J. S. Fairlamb, "Light- and Manganese-Initiated Borylation of Aryl Diazonium Salts: Mechanistic Insight on the Ultrafast Time-Scale Revealed by Time-Resolved Spectroscopic Analysis", *Chem. Eur. J.*, 2021, **27**, 3979–3985.

26. T. Niwa, H. Ochiai, Y. Watanabe and T. Hosoya, "Ni/Cu-Catalyzed Defluoroborylation of Fluoroarenes for Diverse C–F Bond Functionalizations", *J. Am. Chem. Soc.*, 2015, **137**, 14313–14318.

27. D. Qiu, L. Jin, Z. Zheng, H. Meng, F. Mo, X. Wang, Y. Zhang and J. Wang, "Synthesis of Pinacol Arylboronates from Aromatic Amines: A Metal-Free Transformation", *J. Org. Chem.*, 2013, **78**, 1923–1933.

28. L. Kuehn, M. Huang, U. Radius and T. B. Marder, "Copper-Catalysed Borylation of Aryl Chlorides", *Org. Biomol. Chem.*, 2019, **17**, 6601–6606.

29. M. M. Mahamudul Hassan, B. Mondal, S. Singh, C. Haldar, J. Chaturvedi, R. Bisht, R. B. Sunoj and B. Chattopadhyay, "Ir-Catalyzed Ligand-Free Directed C–H Borylation of Arenes and Pharmaceuticals: Detailed Mechanistic Understanding", *J. Org. Chem.*, 2022, **87**, 4360–4375.

30. K. L. Billingsley and S. L. Buchwald, "An Improved System for the Palladium-Catalyzed Borylation of Aryl Halides with Pinacol Borane", *J. Org. Chem.*, 2008, **73**, 5589–5591.

31. M. T. Mihai, B. D. Williams and R. J. Phipps, "*para*-Selective C–H Borylation of Common Arene Building Blocks Enabled by Ion-Pairing with a Bulky Countercation", *J. Am. Chem. Soc.*, 2019, **141**, 15477–15482.

32. J. R. Montero Bastidas, T. J. Oleskey, S. L. Miller, M. R. Smith III and R. E. Maleczka, Jr., "*para*-Selective, Iridium-Catalyzed C–H Borylations of Sulfated Phenols, Benzyl Alcohols,

and Anilines Directed by Ion-Pair Electrostatic Interactions", *J. Am. Chem. Soc.* 2019, **141**, 15483–15487.

33. C. R. K. Jayasundara, J. M. Gil-Negrete, J. R. Montero Bastidas, A. Chhabra, M. M. Martínez, J. Pérez Sestelo, M. R. Smith, R. E. Maleczka, "Merging Iridium-Catalyzed C–H Borylations with Palladium-Catalyzed Cross-Couplings Using Triorganoindium Reagents" *J. Org. Chem.*, 2022, **87**, 751–759.

34. S. Jin, H. T. Dang, G. C. Haug, R. He, V. D. Nguyen, V. T. Nguyen, H. D. Arman, K. S. Schanze and O. V. Larionov, "Visible Light-Induced Borylation of C–O, C–N, and C–X Bonds", *J. Am. Chem. Soc.*, 2020, **142**, 1603–1613.

35. E. Yamamoto, K. Izumi, Y. Horita and H. Ito, "Anomalous Reactivity of Silylborane: Transition-Metal-Free Boryl Substitution of Aryl, Alkenyl, and Alkyl Halides with Silylborane/Alkoxy Base Systems", *J. Am. Chem. Soc.*, 2012, **134**, 19997–20000.

36. B. J. Stokes, S. M. Opra and M. S. Sigman, "Palladium-Catalyzed Allylic Cross-Coupling Reactions of Primary and Secondary Homoallylic Electrophiles", *J. Am. Chem. Soc.*, 2012, **134**, 11408–11411.

37. Y. Lee, S.-Y. Baek, J. Park, S.-T. Kim, S. Tussupbayev, J. Kim, M.-H. Baik and S. H. Cho, "Chemoselective Coupling of 1,1-Bis[(pinacolato)boryl]alkanes for the Transition-Metal-Free Borylation of Aryl and Vinyl Halides: A Combined Experimental and Theoretical Investigation", *J. Am. Chem. Soc.*, 2017, **139**, 976–984.

38. C. Fricke, K. Deckers and F. Schoenebeck, "Orthogonal Stability and Reactivity of Aryl Germanes Enables Rapid and Selective (Multi)Halogenations", *Angew. Chem. Int. Ed.*, 2020, **59**, 18717–18722.

39. S. K. Bose and T. B. Marder, "Synthesis of Aryl Boronates via Zinc-Catalyzed Cross-Coupling of Alkoxy Diboron Reagents with Aryl Halides at Room Temperature", *Org. Lett.*, 2014, **16**, 4562–4565.

40. H. Marom, S. Antonov, Y. Popowski and M. Gozin, "Selective Sulfoxidation of Thioethers and Thioaryl Boranes with Nitrate, Promoted by a Molybdenum–Copper Catalytic System", *J. Org. Chem.*, 2011, **76**, 5240–5246.

41. P. Dai, X. Ning, H. Wang, X. Cui, J. Liu, J. Qu and Y. Kang, "Cleavage of C(Aryl)−CH_3 Bonds in the Absence of Directing Groups under Transition Metal Free Conditions", *Angew. Chem. Int. Ed.*, 2019, **58**, 5392–5395.

42. J. Hu, Y. Zhao, J. Liu, Y. Zhang and Z. Shi, "Nickel-Catalyzed Decarbonylative Borylation of Amides: Evidence for Acyl C−N Bond Activation", *Angew. Chem. Int. Ed.*, 2016, **55**, 8718–8722.

43. T. O. Ronson, E. Renders, B. F. Van Steijvoort, X. Wang, C. C. D. Wybon, H. Prokopcová, L. Meerpoel and B. U. W. Maes, "Ruthenium-Catalyzed Reductive Arylation of *N*-(2-Pyridinyl)Amides with Isopropanol and Arylboronate Esters", *Angew. Chem. Int. Ed.*, 2019, **58**, 482–487.

44. C. A. Malapit, J. R. Bour, S. R. Laursen and M. S. Sanford, "Mechanism and Scope of Nickel-Catalyzed Decarbonylative Borylation of Carboxylic Acid Fluorides", *J. Am. Chem. Soc.*, 2019, **141**, 17322–17330.

45. H. Ochiai, Y. Uetake, T. Niwa and T. Hosoya, "Rhodium-Catalyzed Decarbonylative Borylation of Aromatic Thioesters for Facile Diversification of Aromatic Carboxylic Acids", *Angew. Chem. Int. Ed.*, 2017, **56**, 2482–2486.

46. T. L. Andersen, M. W. Frederiksen, K. Domino and T. Skrydstrup, "Direct Access to α,α-Difluoroacylated Arenes by Palladium-Catalyzed Carbonylation of (Hetero)Aryl Boronic Acid Derivatives", *Angew. Chem. Int. Ed.*, 2016, **55**, 10396–10400.

47. S. Yasuike, K. Nakata, W. Qin, M. Matsumura, N. Kakusawa and J. Kurita, "Synthesis of Arylboronates by Boron-Induced *ipso*-Deantimonation of Triarylstibanes with Boron Trihalides and Its Application in One-Pot Two-Step Transmetallation/Cross-Coupling Reactions", *J. Organomet. Chem.*, 2015, **788**, 9–16.

48. K. Ito, H. Tamashima, N. Iwasawa and H. Kusama, "Photochemically Promoted Transition Metal-Free Cross-Coupling of Acylsilanes with Organoboronic Esters", *J. Am. Chem. Soc.*, 2011, **133**, 3716–3719.

49. S. De Ornellas, J. M. Slattery, R. M. Edkins, A. Beeby, C. G. Baumann and I. J. S. Fairlamb, "Design and Synthesis of Fluorescent 7-Dezaadenosine Nucleosides Containing π-Extended Diarylacetylene Motifs", *Org. Biomol. Chem.*, 2015, **13**, 68–72.

50. J. Takaya, S. Tadami, K. Ukai and N. Iwasawa, "Copper(I)-Catalyzed Carboxylation of Aryl- and Alkenylboronic Esters", *Org. Lett.*, 2008, **10**, 2697–2700.

51. This substance is commercially available from BLD Pharmatech Ltd.; CAS: 2096997-26-9.

52. M. Tobisu, H. Kinuta, Y. Kita, E. Rémond and N. Chatani, "Rhodium(I)-Catalyzed Borylation of Nitriles through the Cleavage of Carbon–Cyano Bonds", *J. Am. Chem. Soc.*, 2012, **134**, 115–118.

53. M. Huang, Z. Wu, J. Krebs, A. Friedrich, X. Luo, S. A. Westcott, U. Radius and T. B. Marder, "Ni-Catalyzed Borylation of Aryl Sulfoxides", *Chem. Eur. J.*, 2021, **27**, 8149–8158.

54. D. A. Wilson, C. J. Wilson, C. Moldoveanu, A.-M. Resmerita, P. Corcoran, L. M. Hoang, B. M. Rosen and V. Percec, "Neopentylglycolborylation of Aryl Mesylates and Tosylates Catalyzed by Ni-Based Mixed-Ligand Systems Activated with Zn", *J. Am. Chem. Soc.*, 2010, **132**, 1800–1801.

55. J. C. Vantourout, R. P. Law, A. Isidro-Llobet, S. J. Atkinson and A. J. B. Watson, "Chan–Evans–Lam Amination of Boronic Acid Pinacol (BPin) Esters: Overcoming the Aryl Amine Problem", *J. Org. Chem.*, 2016, **81**, 3942–3950.

56. S. Bernhardt, Z.-L. Shen and P. Knochel, "Preparation of Functionalized Organoindium Reagents by Means of Magnesium Insertion into Organic Halides in the Presence of $InCl_3$ at Room Temperature", *Chem. Eur. J.*, 2013, **19**, 828–833.

57. R. Gosmini, V. L. Nguyen, J. Toum, C. Simon, J.-M. G. Brusq, G. Krysa, O. Mirguet, A. M. Riou-Eymard, E. V. Boursier, L. Trottet, P. Bamborough, H. Clark, C. Chung, L. Cutler, E. H. Demont, R. Kaur, A. J. Lewis, M. B. Schilling, P. E. Soden, S. Taylor, A. L. Walker and M. D. Walker, "The Discovery of I-BET726 (GSK1324726A), a Potent Tetrahydroquinoline ApoA1 Up-Regulator and Selective BET Bromodomain Inhibitor", *J. Med. Chem.*, 2014, **57**, 8111–8131.

58. A. W. M. Cummins, S. Li, D. R. Willcox, T. Muilu, J. H. Docherty and S. P. Thomas, "Synthesis of DBpin Using Earth-Abundant Metal Catalysis", *Tetrahedron*, 2020, **76**, 131084.

59. T. F. Brewer and C. J. Chang, "An Aza-Cope Reactivity-Based Fluorescent Probe for Imaging Formaldehyde in Living Cells", *J. Am Chem Soc.*, 2015*,* **127***,* 10886-10889.

1.11 Appendix

1.11.1 Scanned Spectra

[Ir(COD)(IMes)Cl] S1

^{1}H NMR (400 MHz, $CDCl_3$)

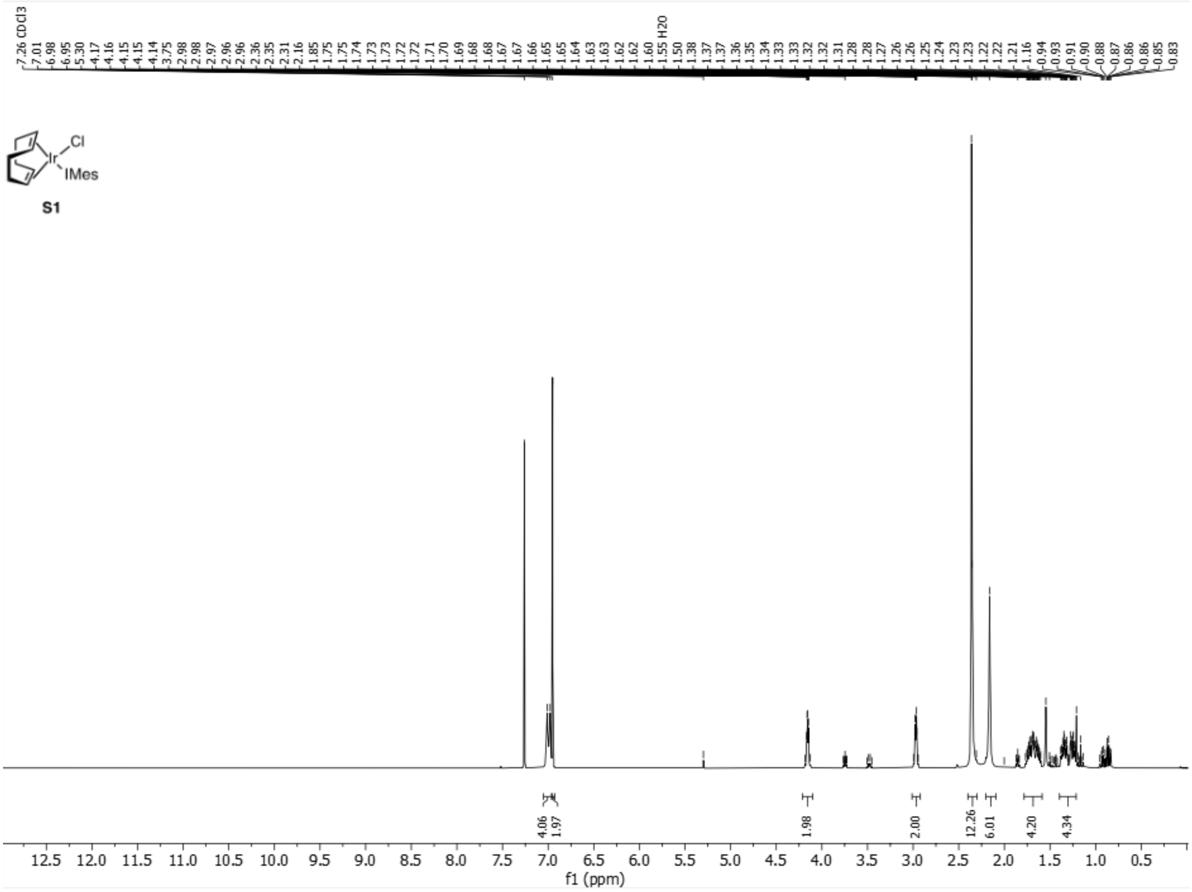

^{13}C NMR (101 MHz, $CDCl_3$)

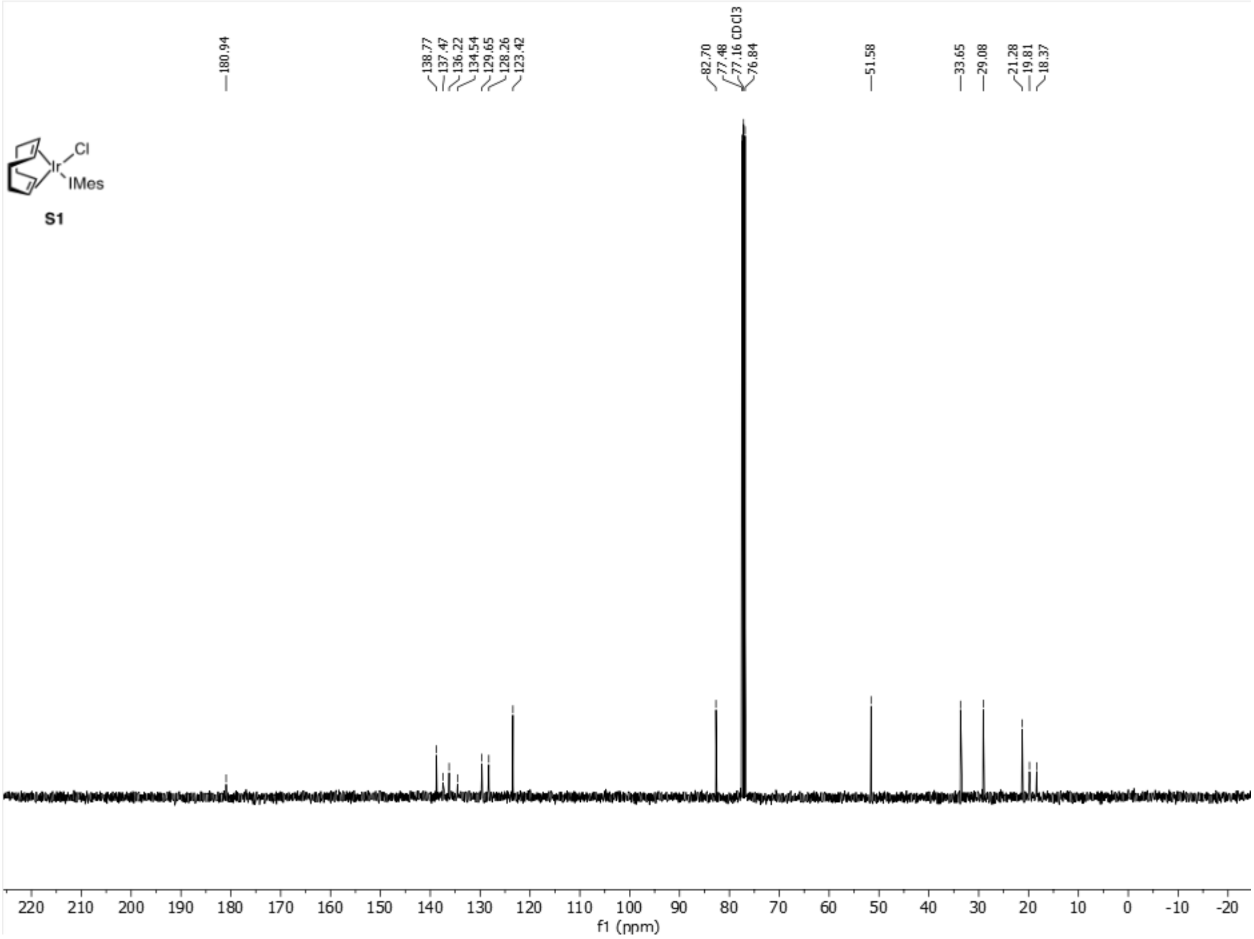

[Ir(COD)(PPh$_3$)(IMes)]BAr^F 1

^{1}H NMR (400 MHz, CDCl$_3$)

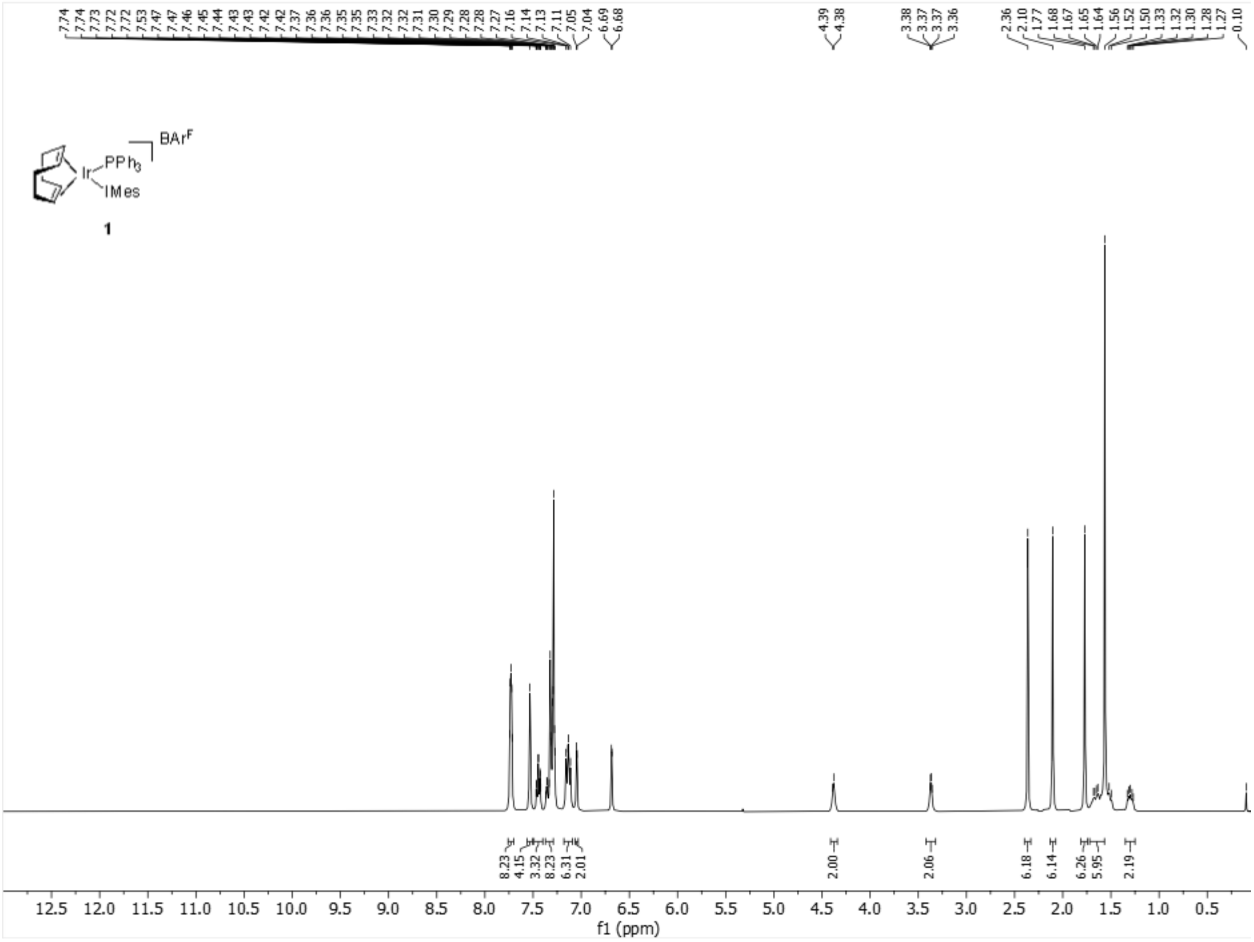

^{13}C NMR (101 MHz, $CDCl_3$)

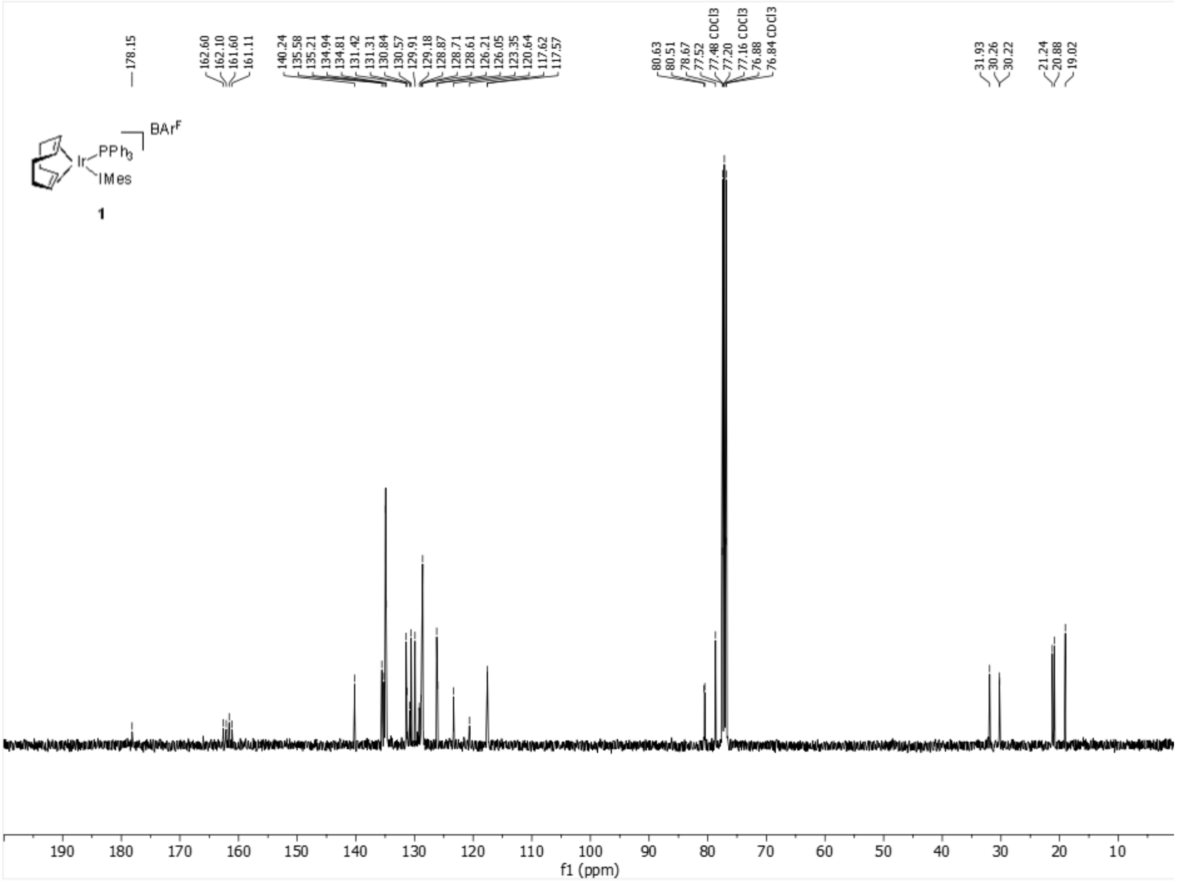

^{31}P NMR (162 MHz, $CDCl_3$)

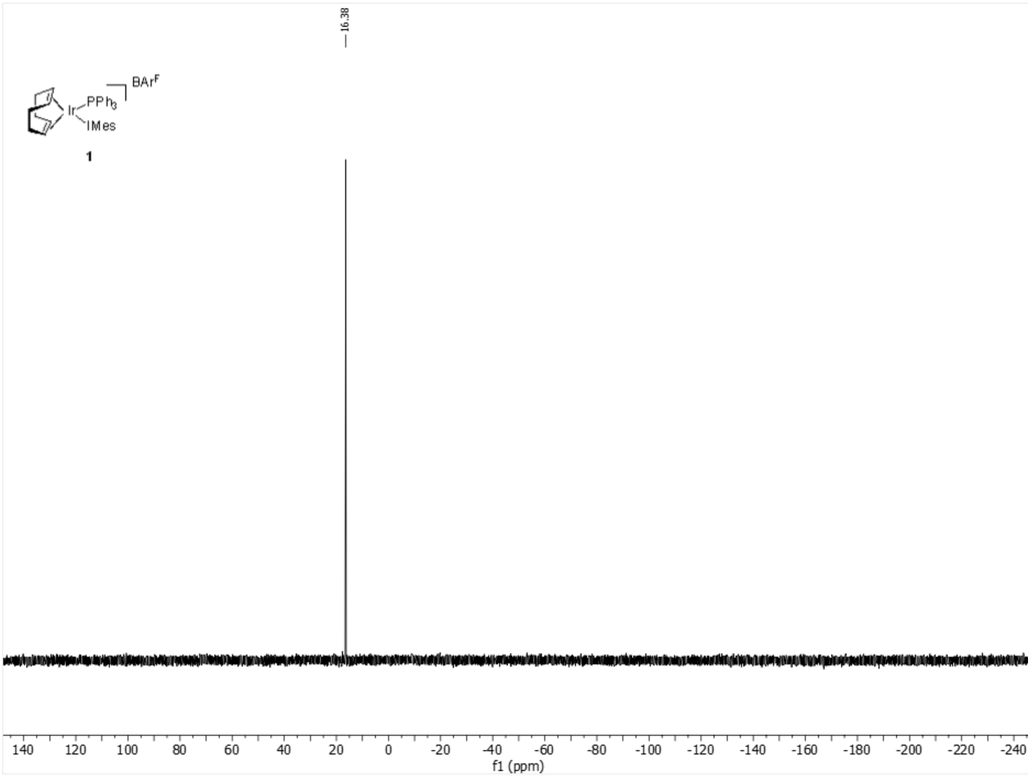

^{19}F NMR (376 MHz, $CDCl_3$)

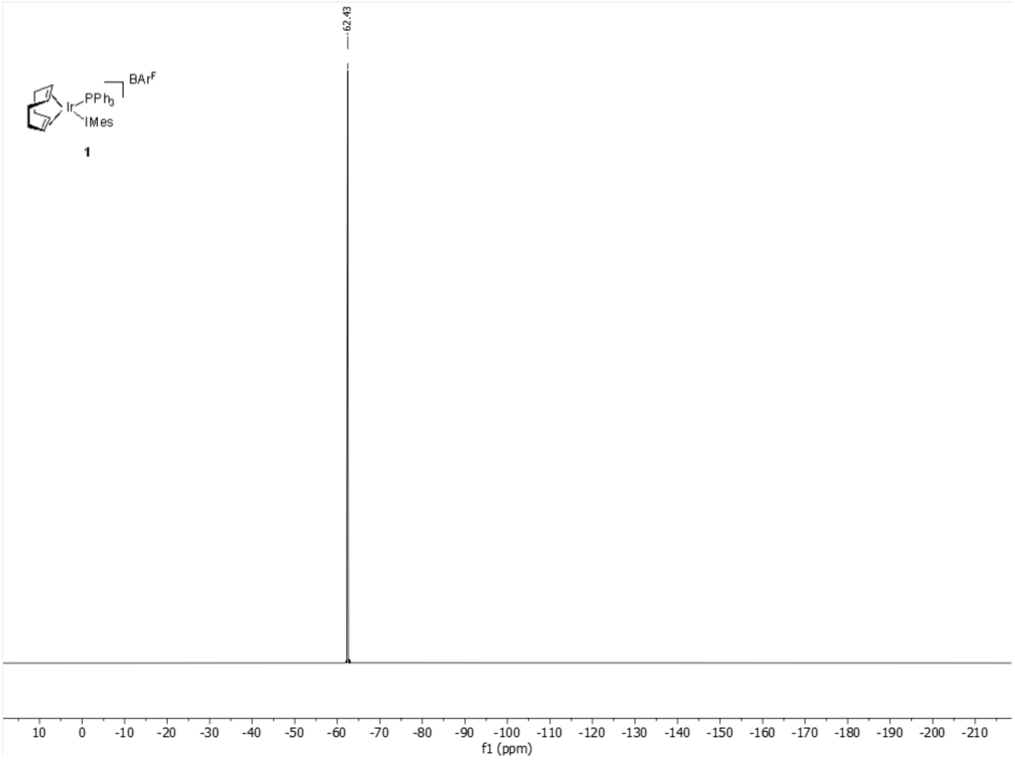

^{11}B NMR (128 MHz, $CDCl_3$)

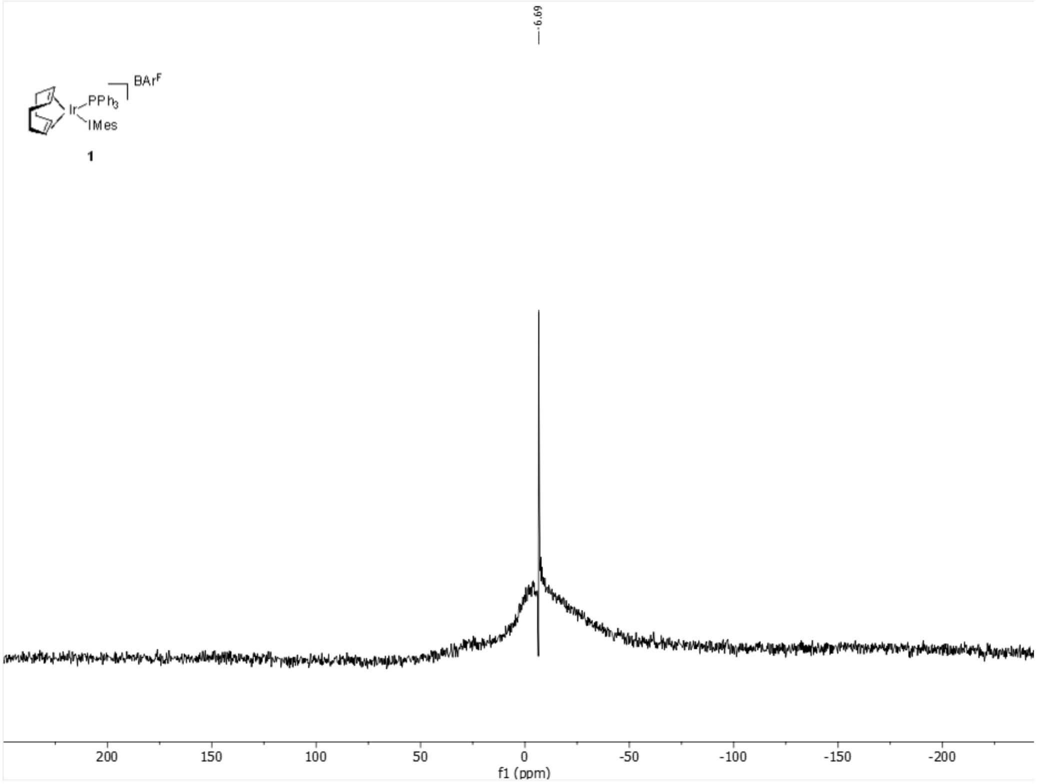

1-Mesityl-1*H*-imidazole S2

^{1}H NMR (400 MHz, $CDCl_3$)

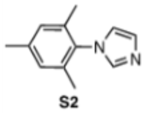

S2

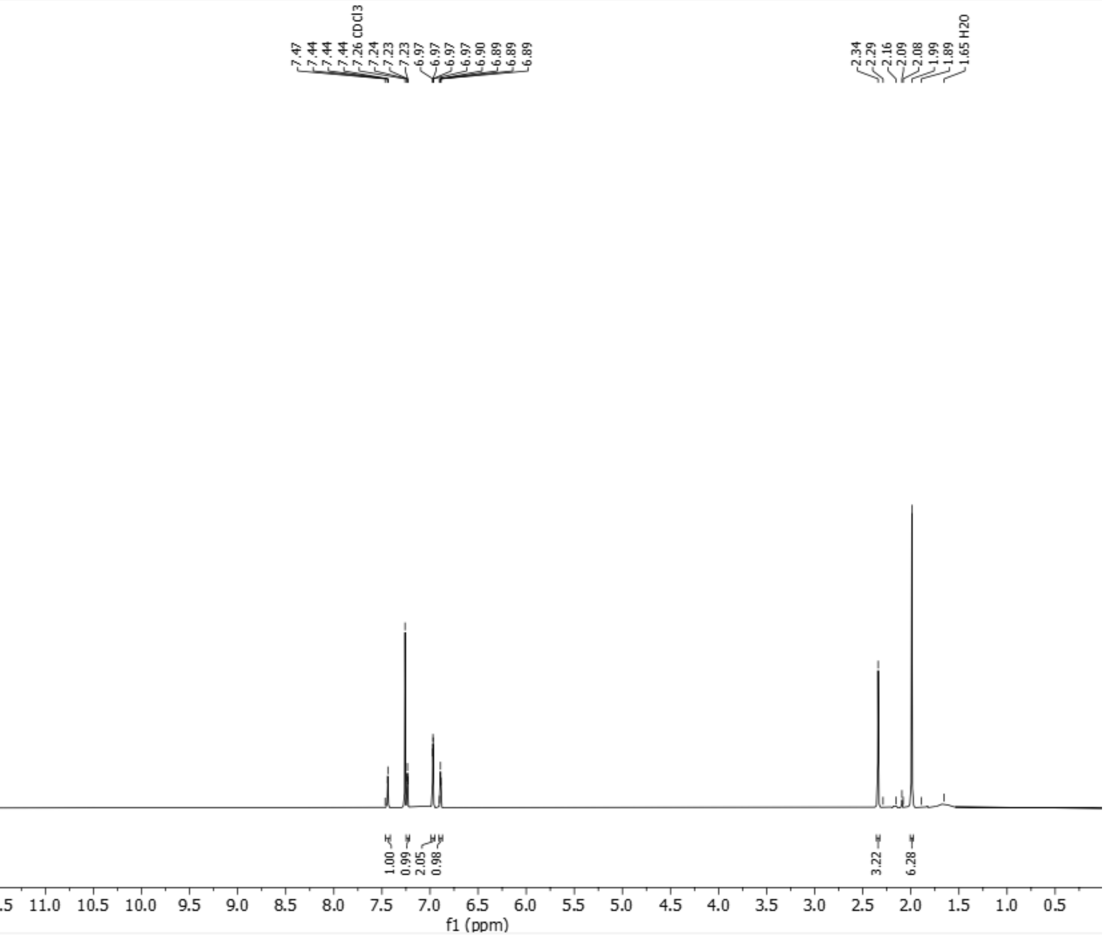

^{13}C NMR (101 MHz, $CDCl_3$)

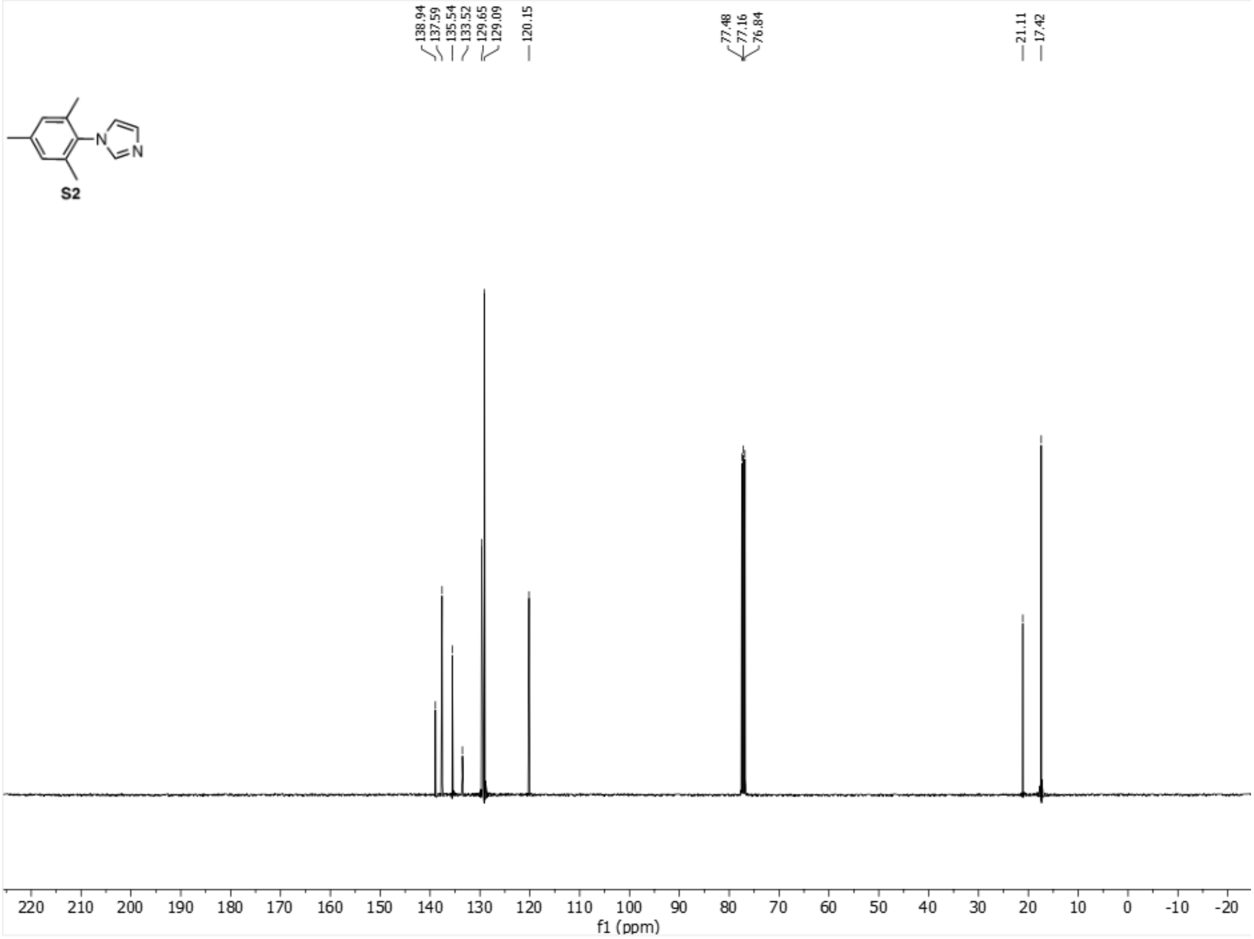

3-(2-Hydroxyethyl)-1-mesityl-1*H*-imidazol-3-ium bromide S3

^1H NMR (400 MHz, $CDCl_3$)

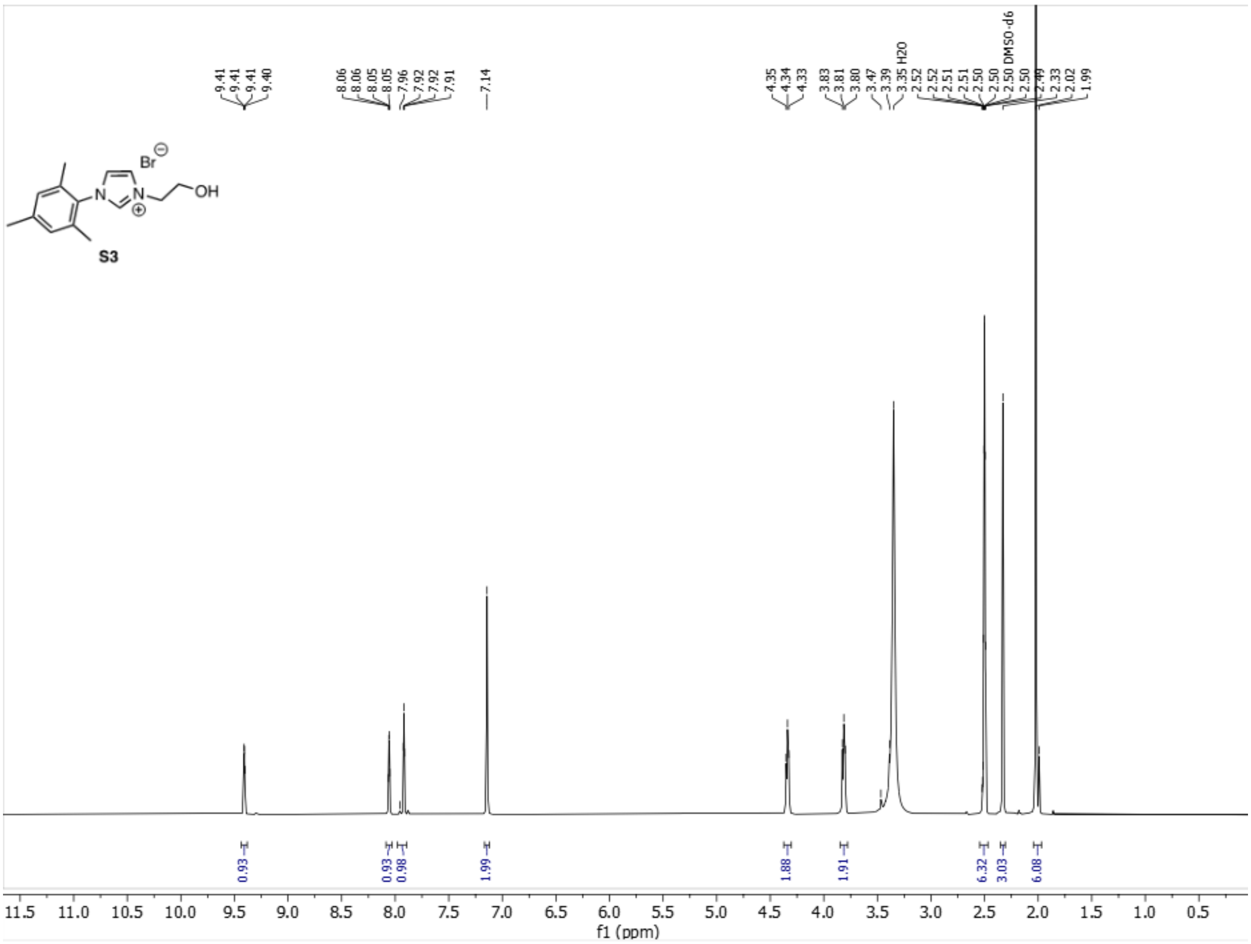

^{13}C NMR (101 MHz, d_6-DMSO)

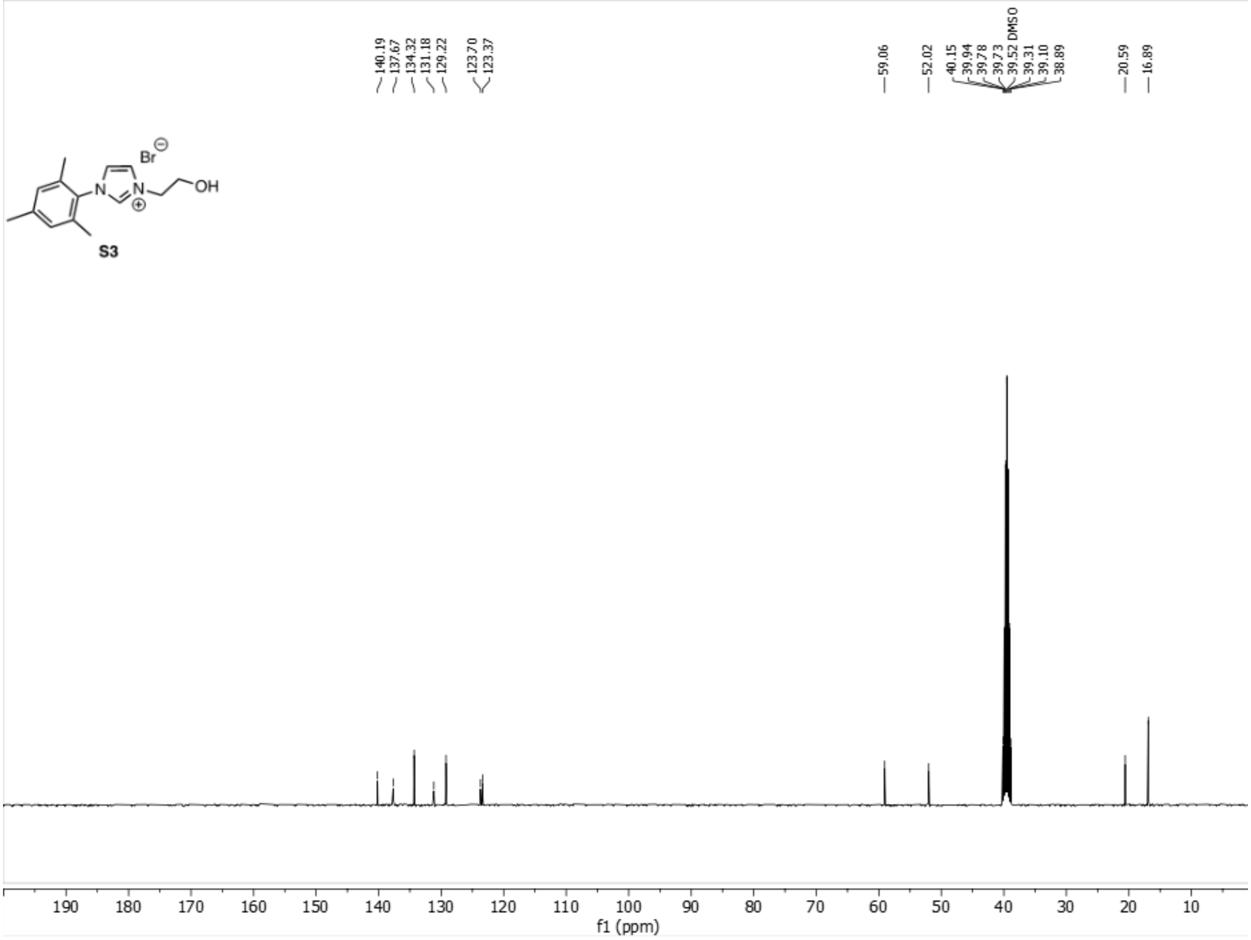

3-(2-Bromoethyl)-1-mesityl-1*H*-imidazol-3-ium bromide S4

^{1}H NMR (400 MHz, $CDCl_3$)

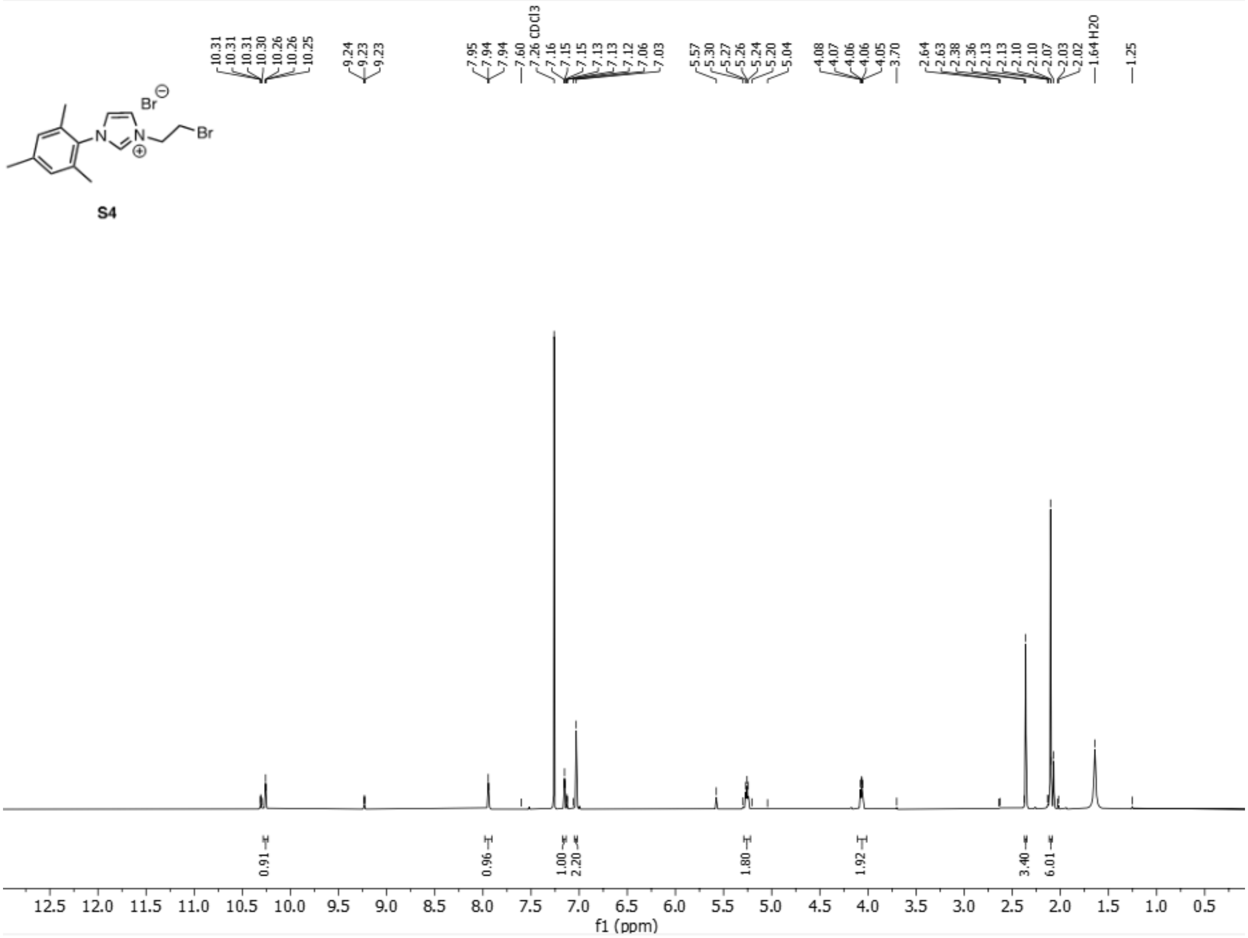

^{13}C NMR (101 MHz, $CDCl_3$)

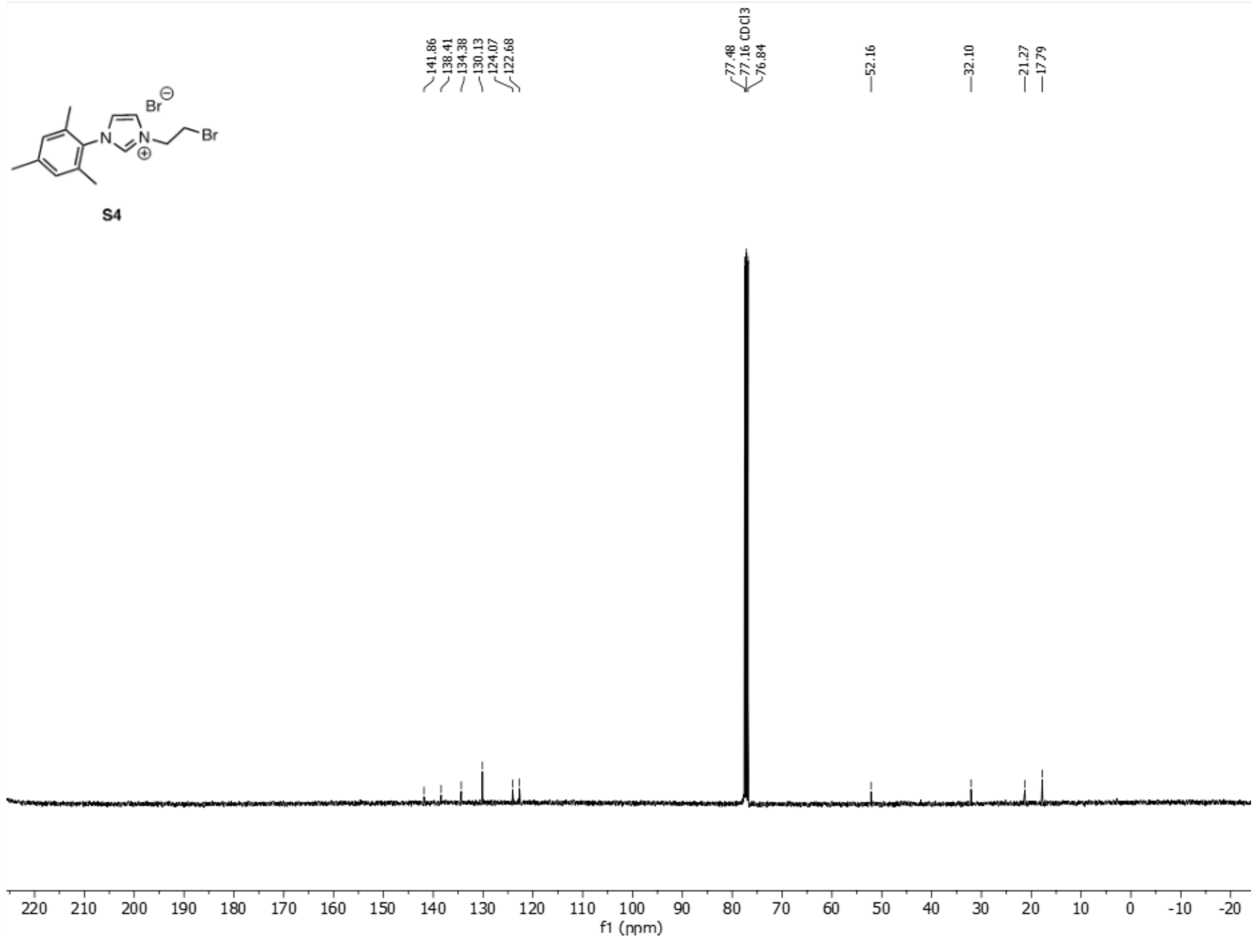

3-(2-(Diphenylphosphanyl)ethyl)-1-mesitylimidazolium BAr^F S5

^{1}H NMR (400 MHz, $CDCl_3$)

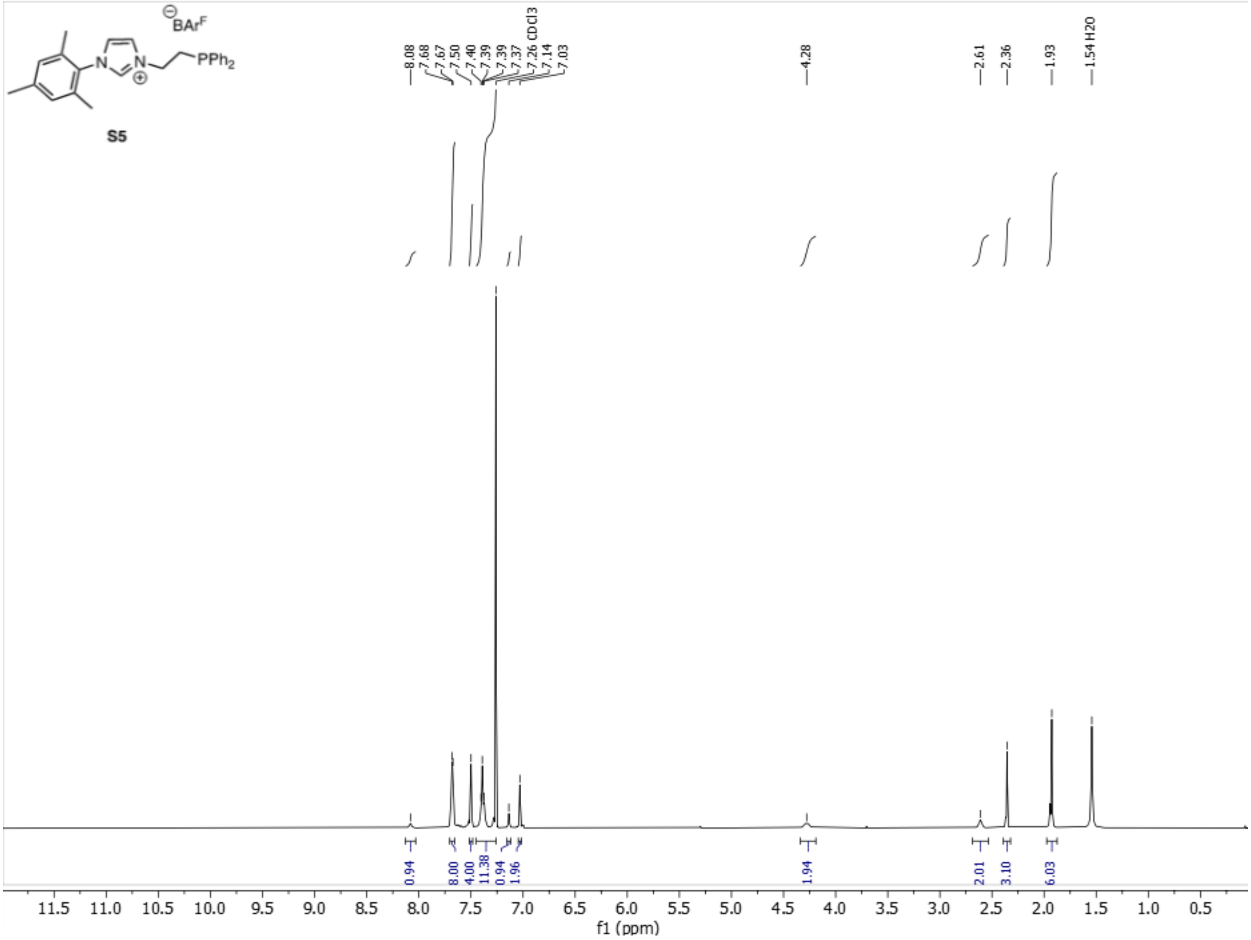

^{13}C NMR (101 MHz, $CDCl_3$)

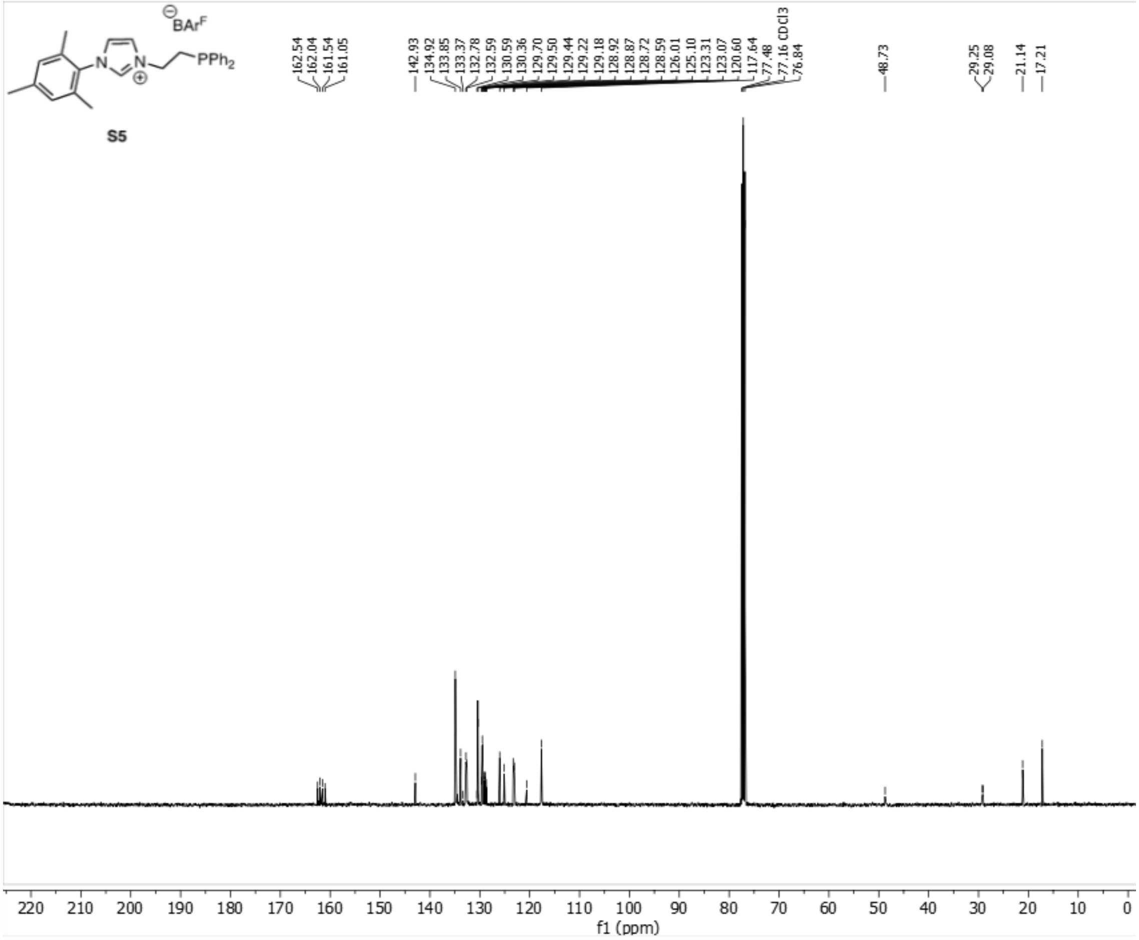

^{31}P NMR (162 MHz, $CDCl_3$)

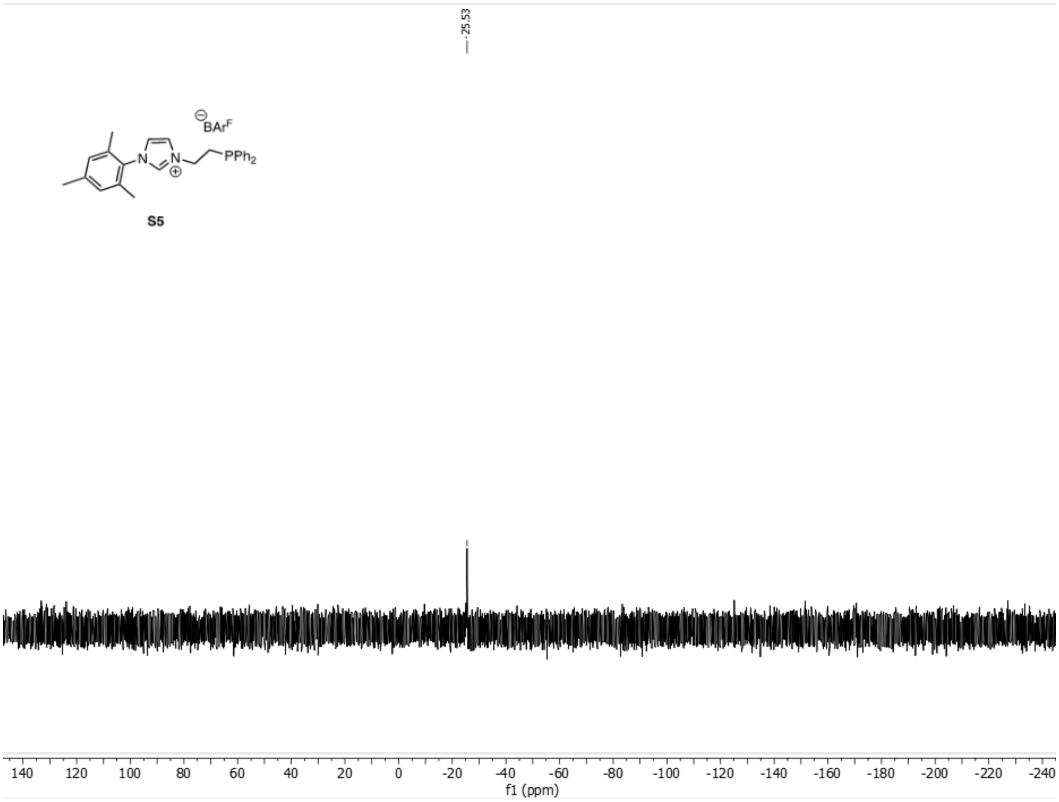

^{11}B NMR (128 MHz, $CDCl_3$)

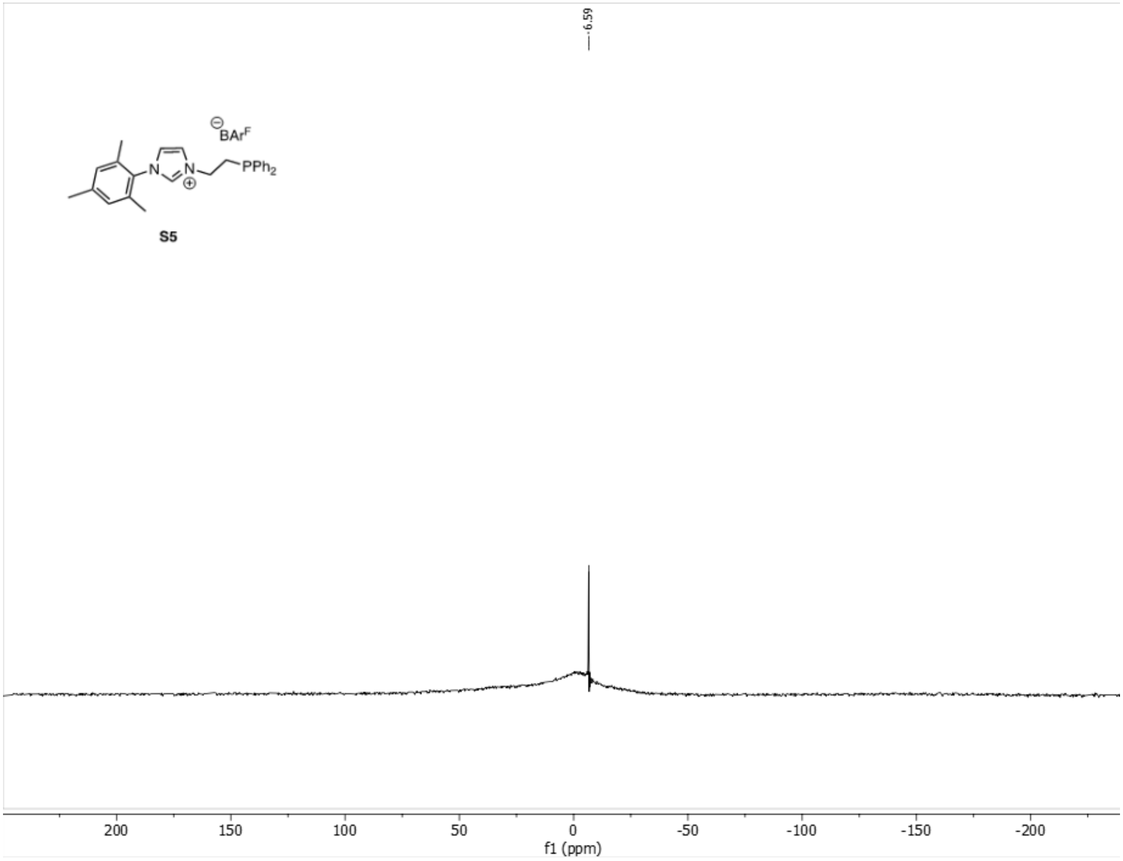

η^4-Cycloocta-1,5-diene(3-(2-(diphenylphosphanyl)ethyl)-1-mesitylimidazole-2-ylidene)iridium tetrakis[3,5-bis(trifluoromethyl)phenyl]borate 7

^{1}H NMR (400 MHz, $CDCl_3$)

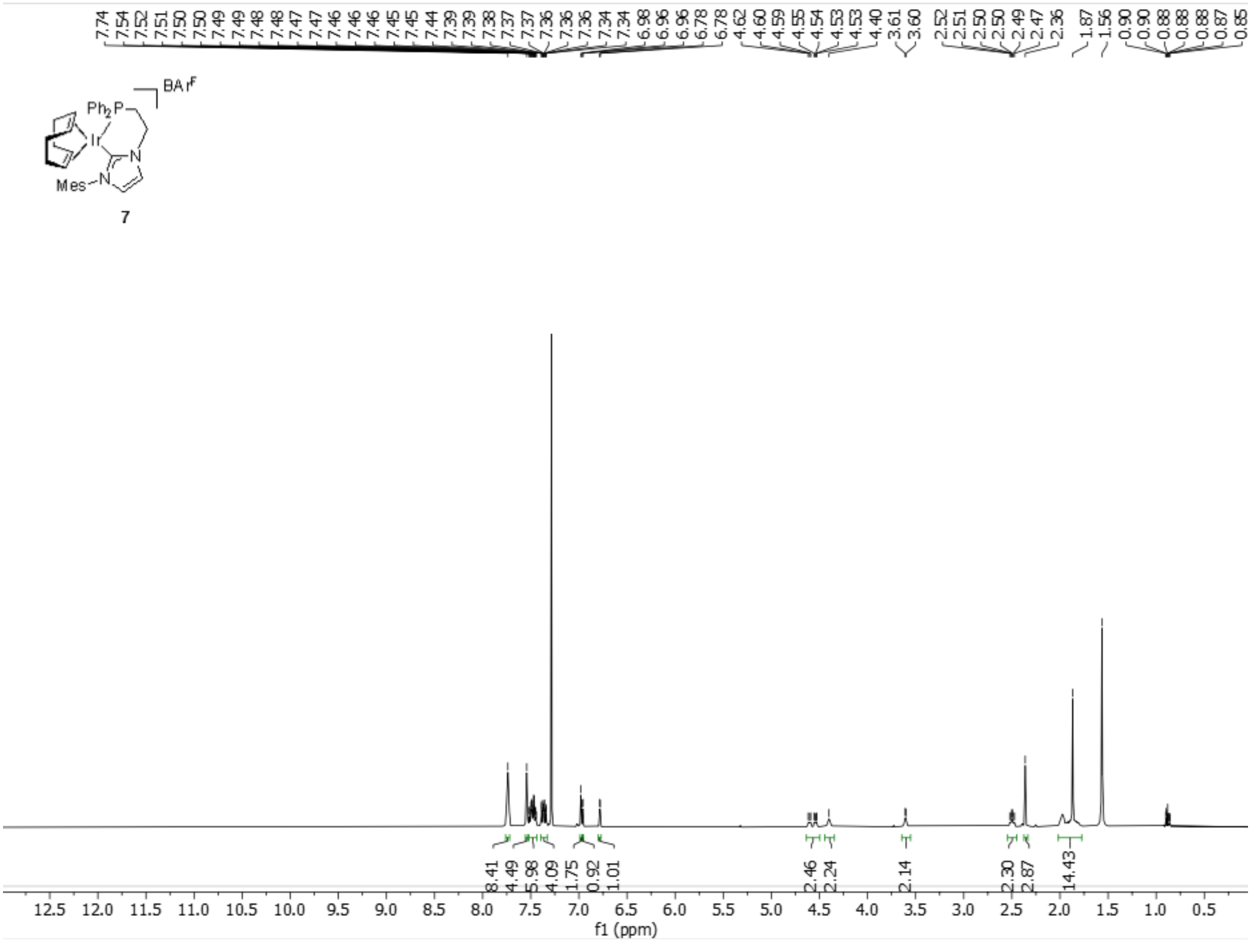

^{13}C NMR (101 MHz, $CDCl_3$)

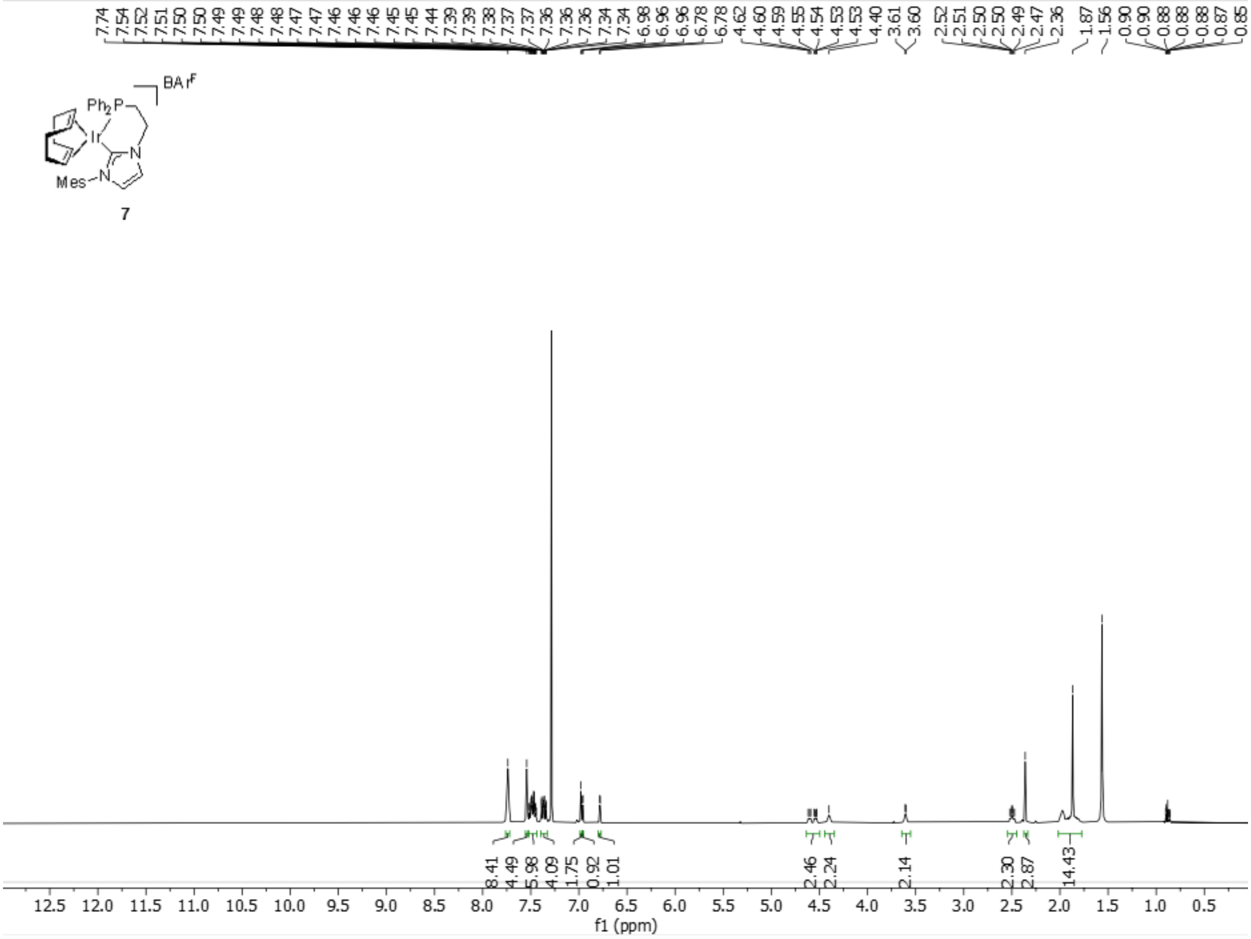

^{31}P NMR (162 MHz, $CDCl_3$)

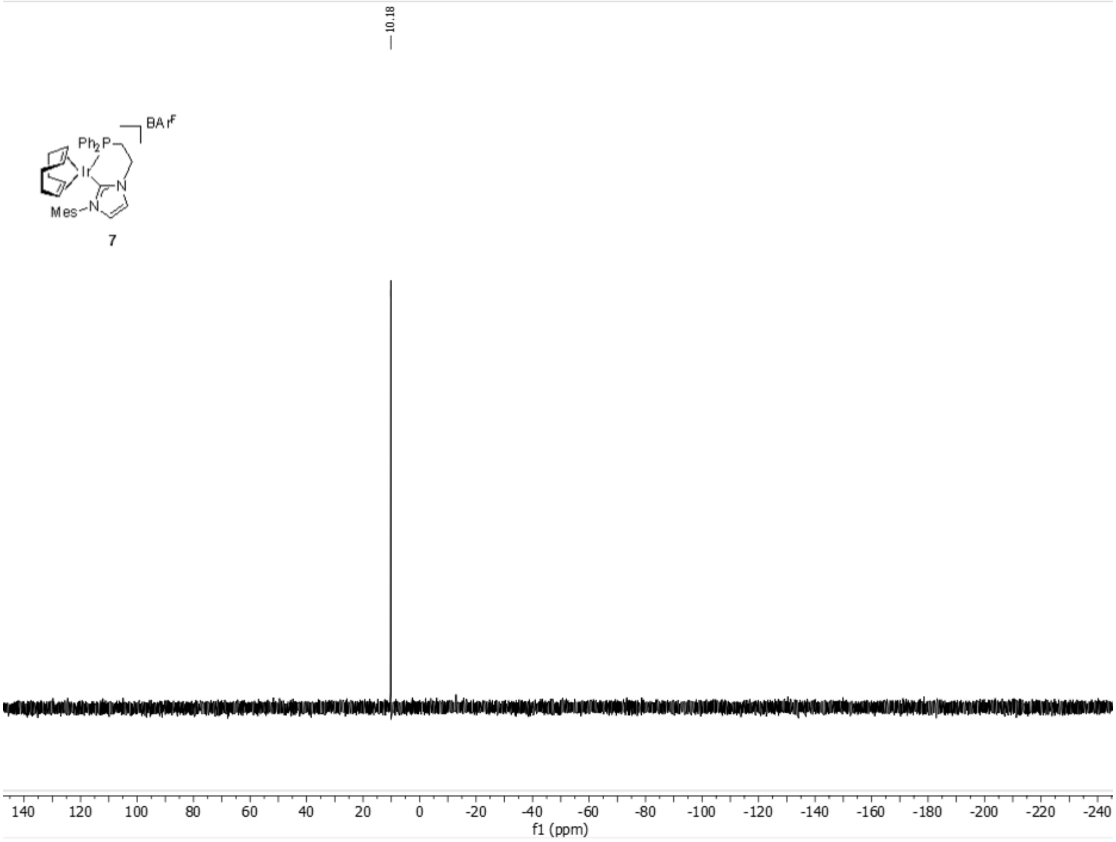

^{11}B NMR (128 MHz, $CDCl_3$)

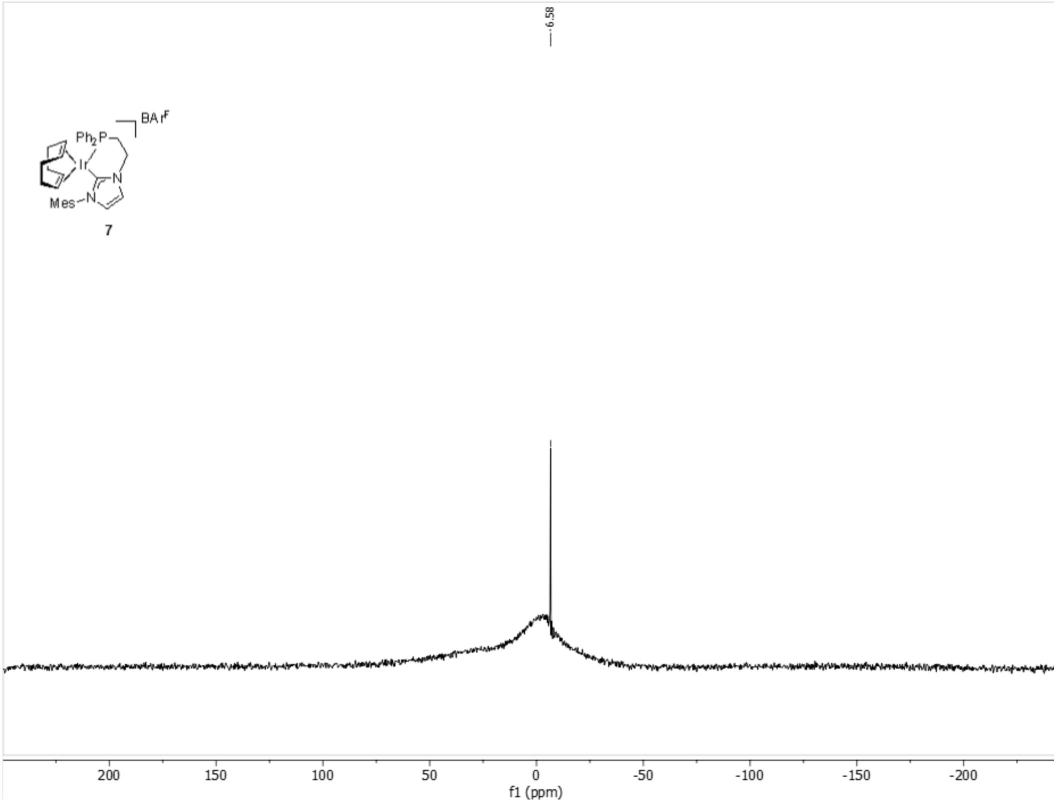

1-(2,6-Diisopropylphenyl)-1*H*-imidazole S6

^{1}H NMR (400 MHz, $CDCl_3$)

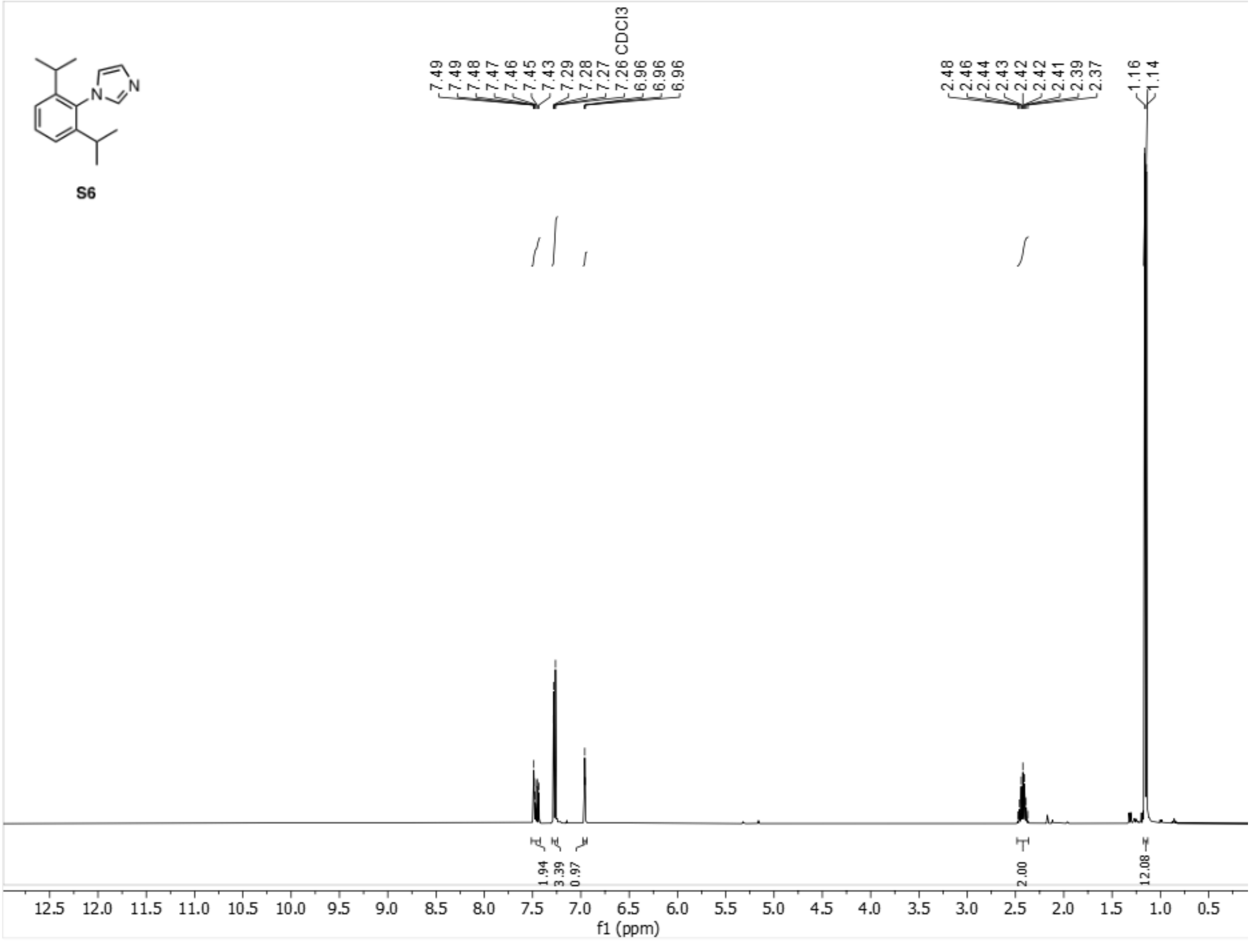

^{13}C NMR (101 MHz, $CDCl_3$)

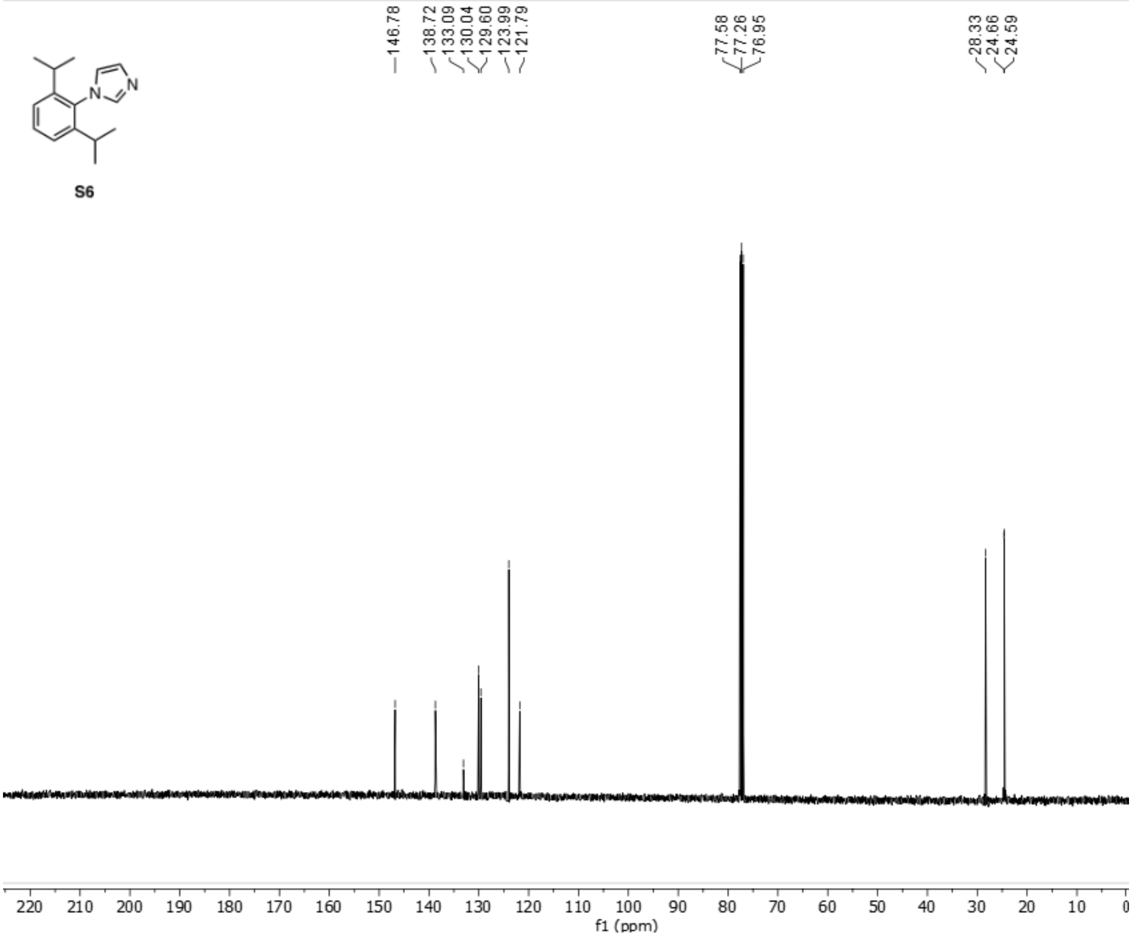

1-(2,6-Diisopropylphenyl)-3-(2-hydroxyethyl)-1*H*-imidazol-3-ium bromide S7

^1H NMR (400 MHz, DMSO)

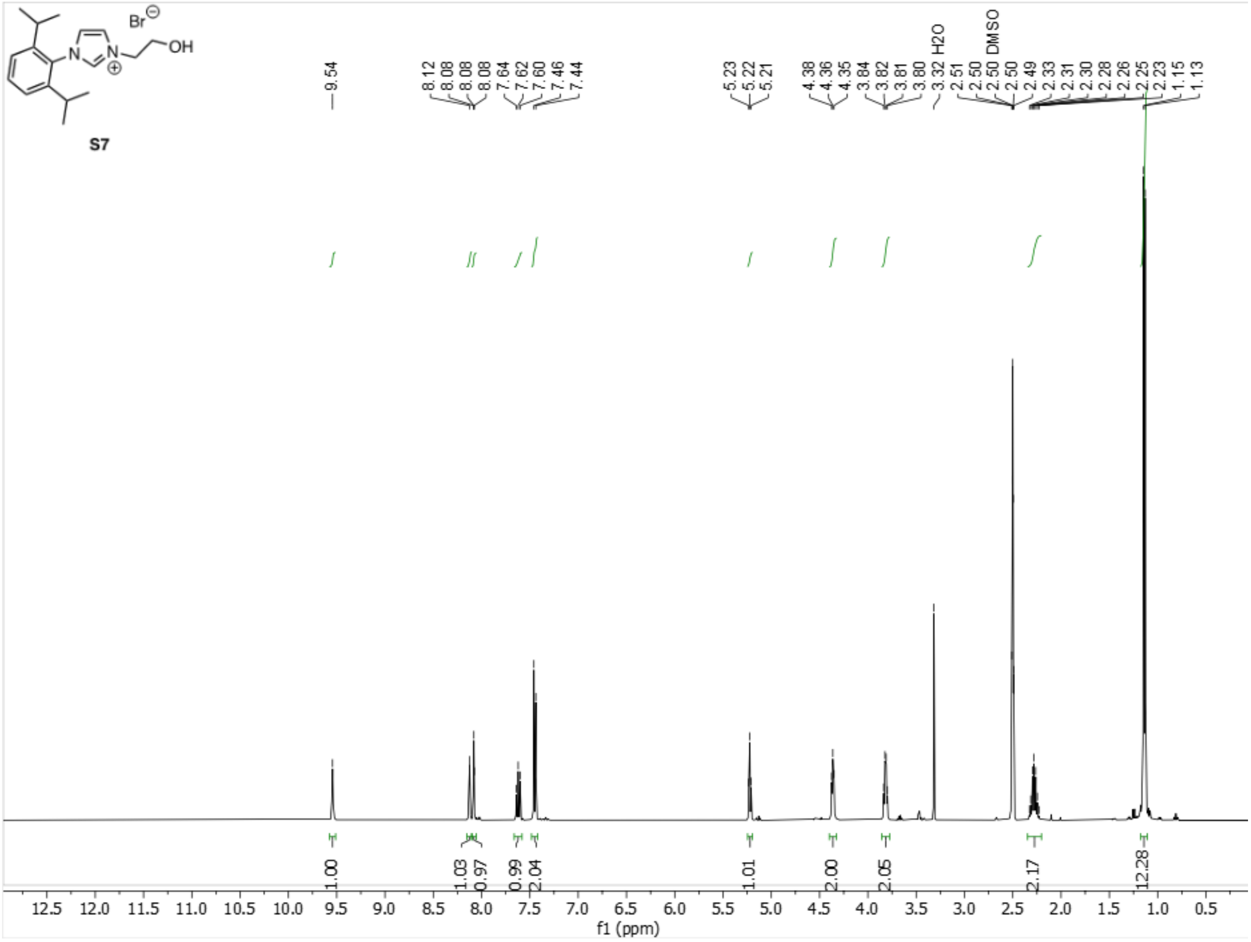

^{13}C NMR (101 MHz, DMSO)

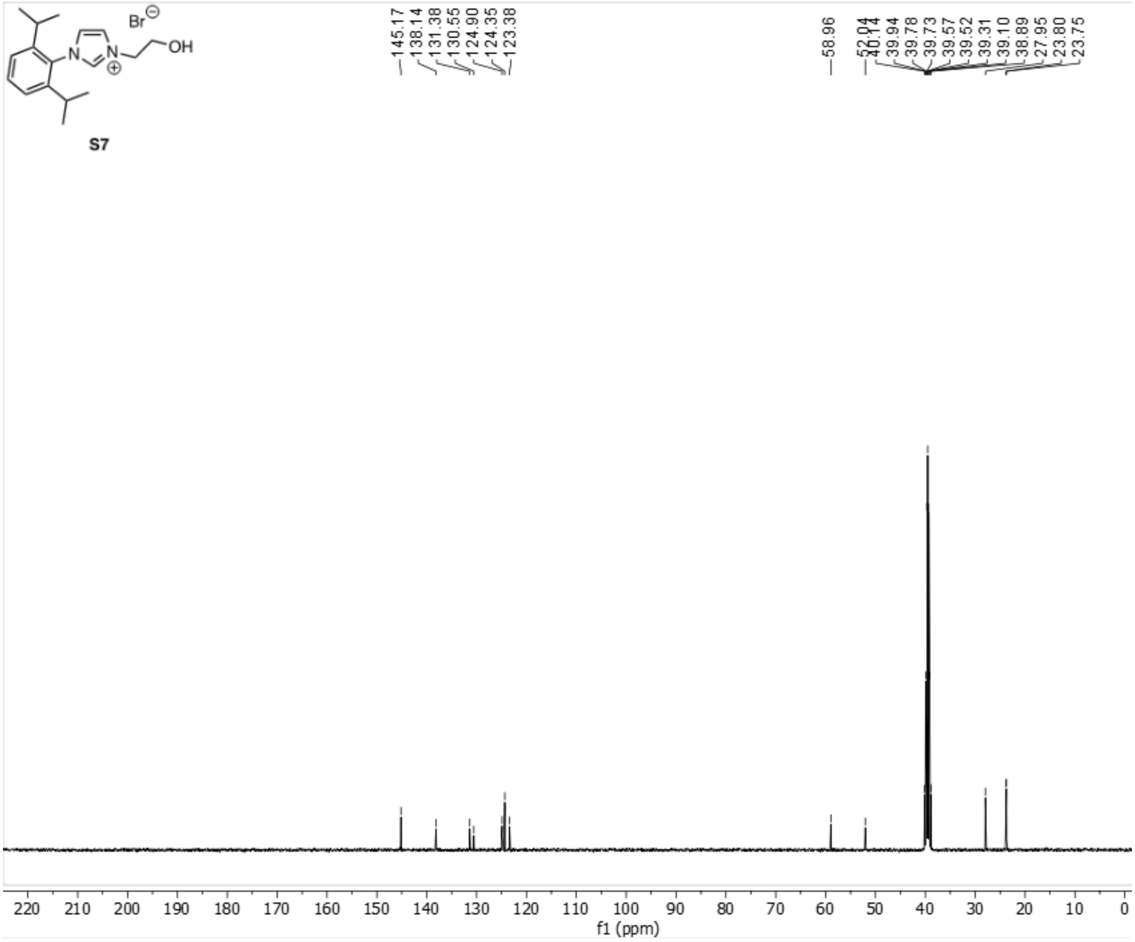

3-(2-Bromoethyl)-1-(2,6-diisopropylphenyl)-1*H*-imidazol-3-ium bromide S8

^{1}H NMR (400 MHz, $CDCl_3$)

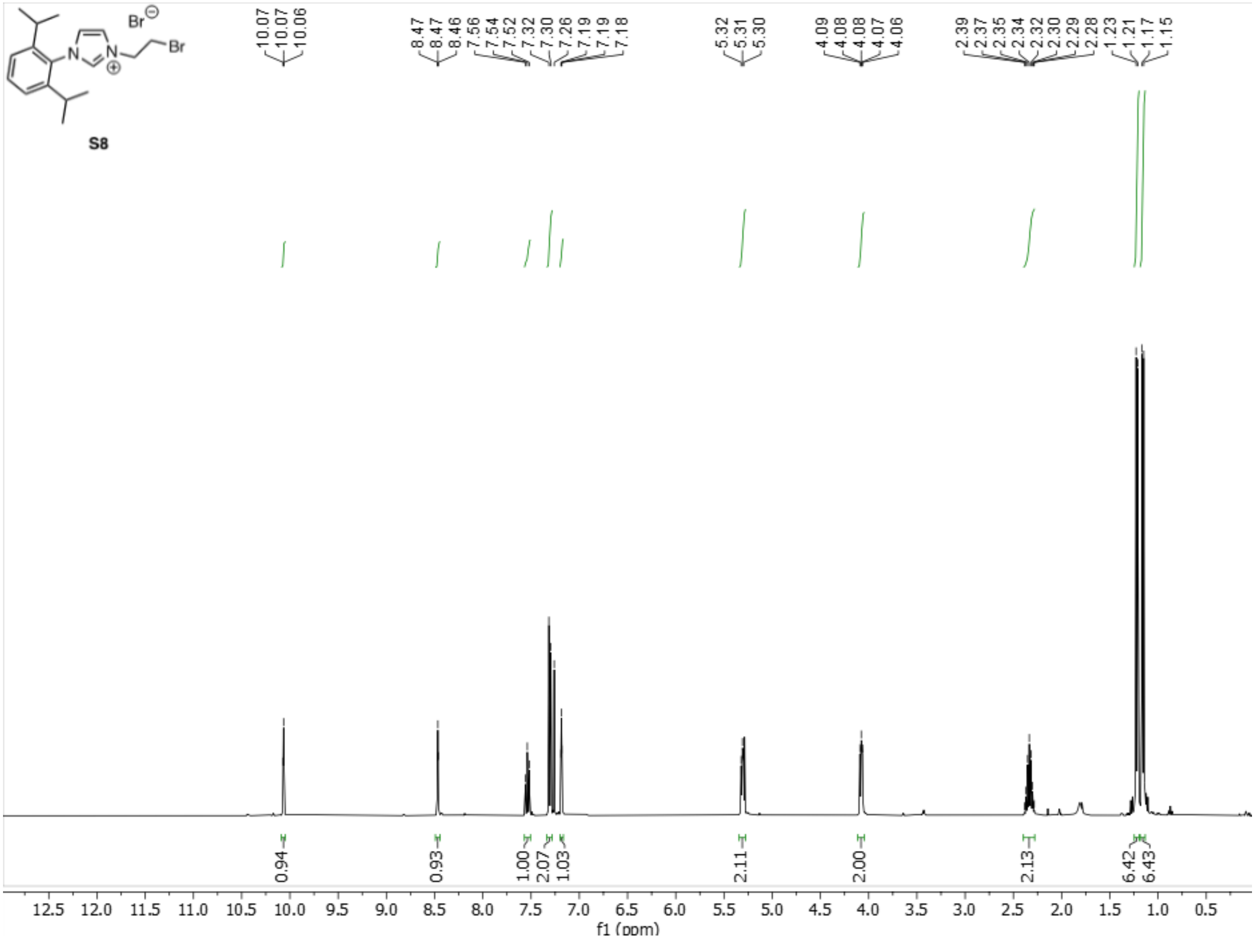

^{13}C NMR (101 MHz, $CDCl_3$)

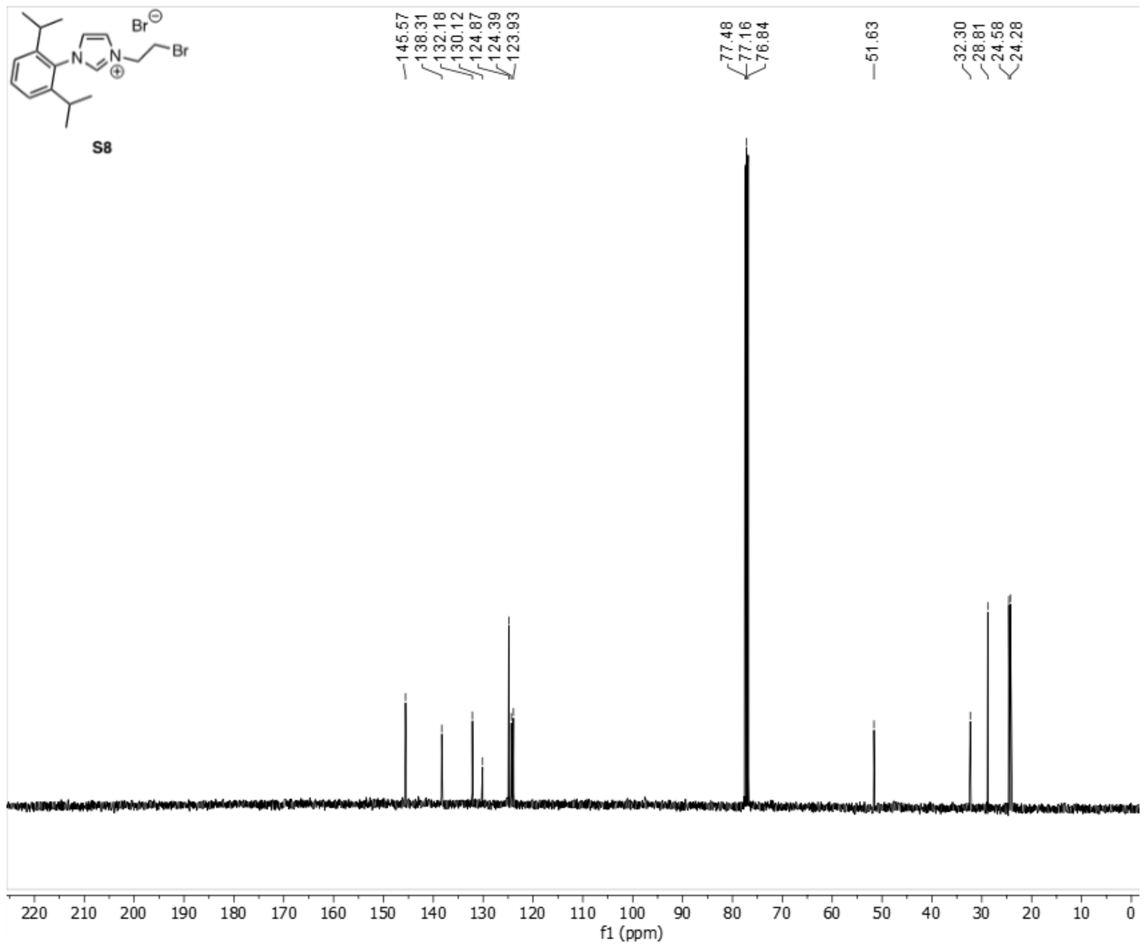

1-(2,6-Diisopropylphenyl)-3-(2-(diphenylphosphaneyl)ethyl)-1*H*-imidazol-3-ium BAr^F S9

^{1}H NMR (400 MHz, $CDCl_3$)

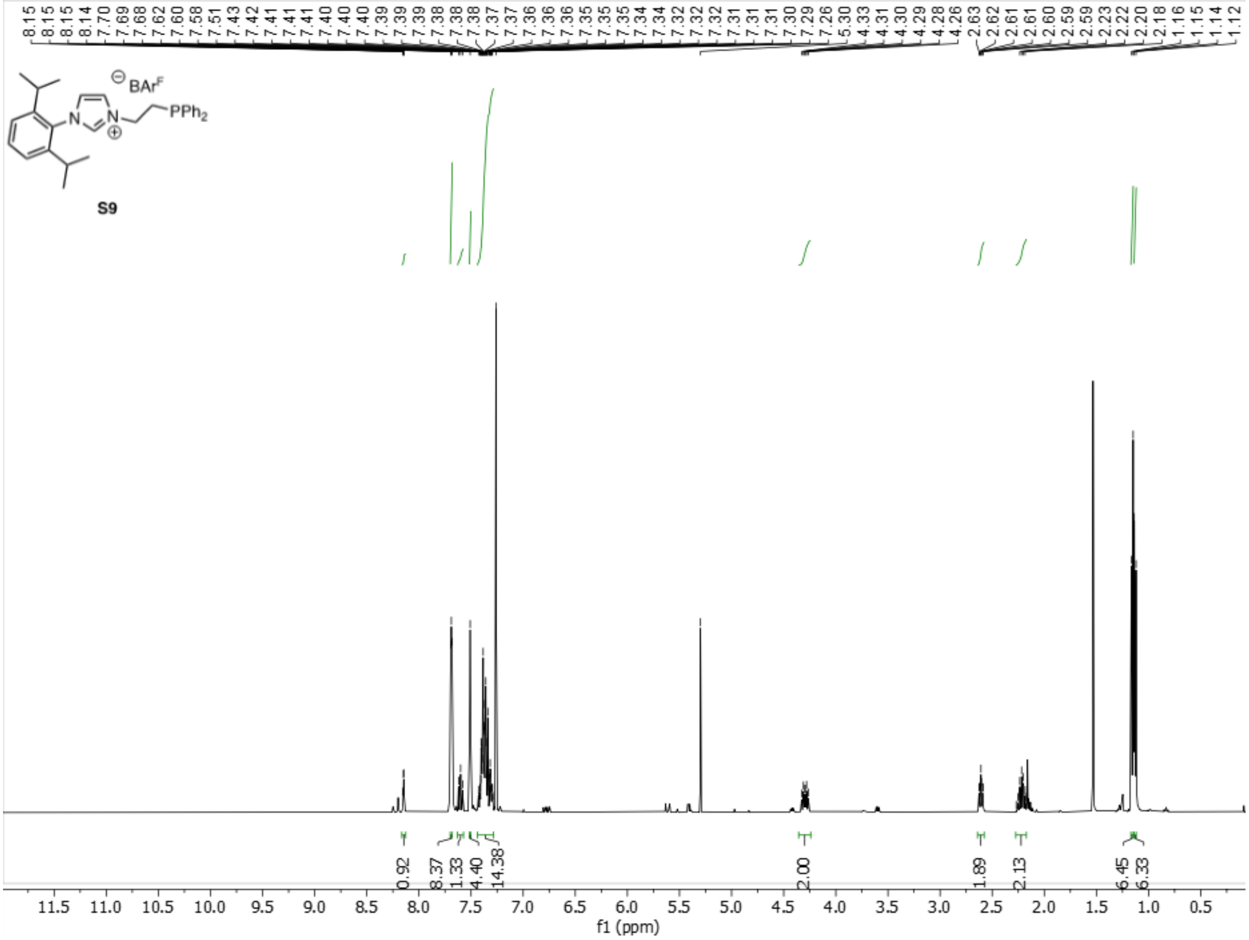

^{13}C NMR (101 MHz, $CDCl_3$)

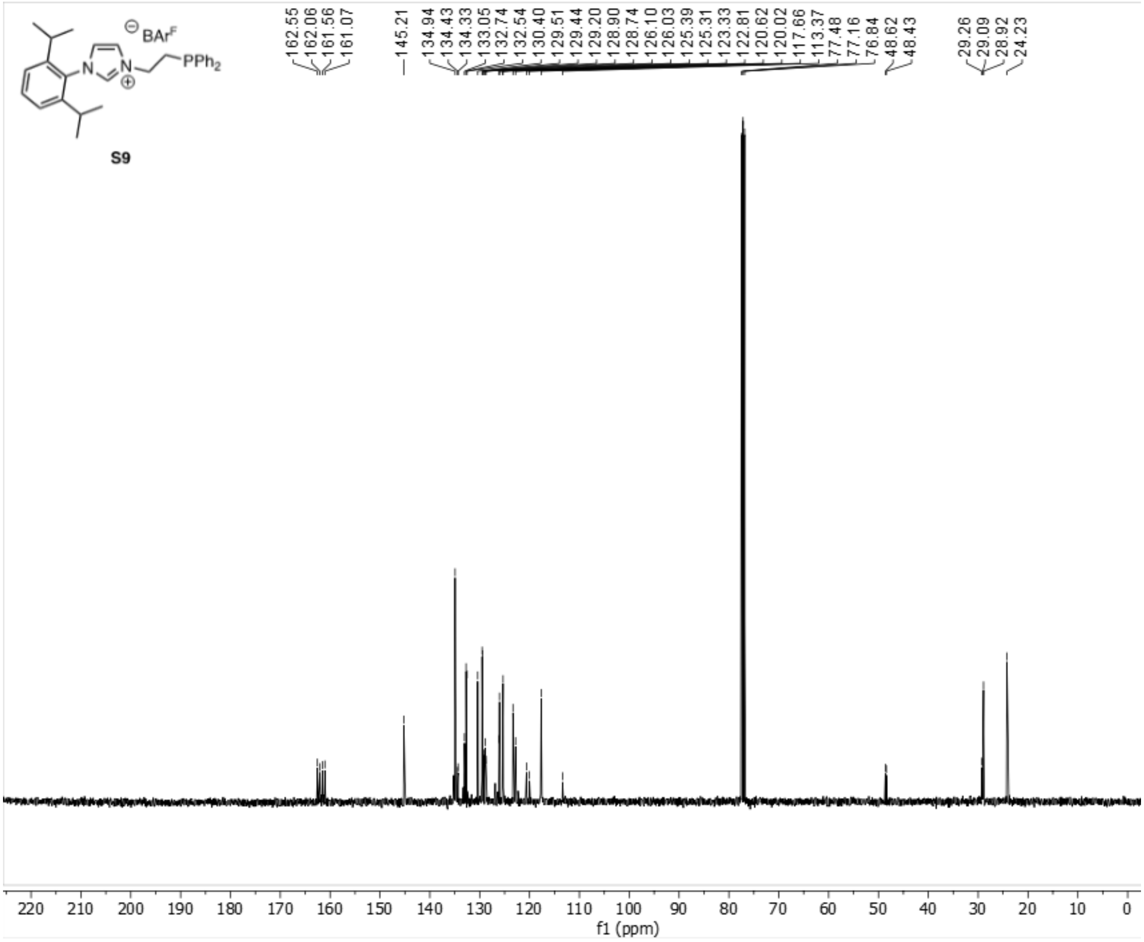

^{31}P NMR (162 MHz, $CDCl_3$)

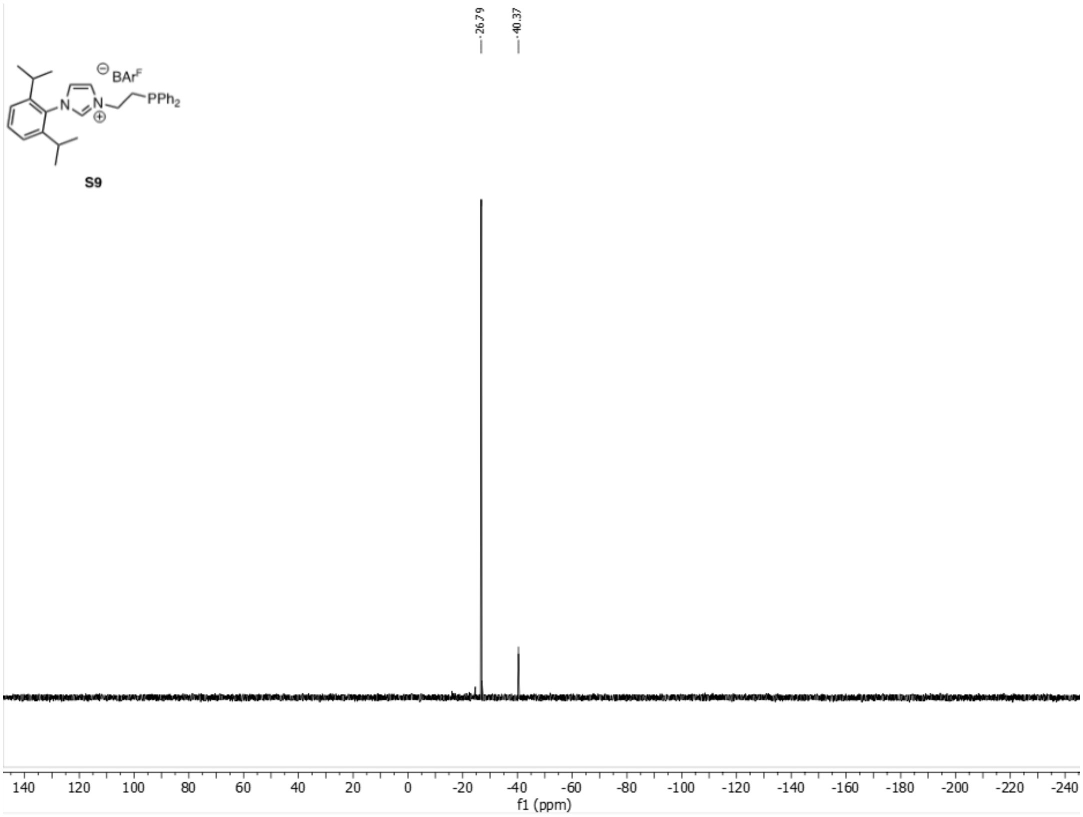

^{19}F NMR (376 MHz, $CDCl_3$)

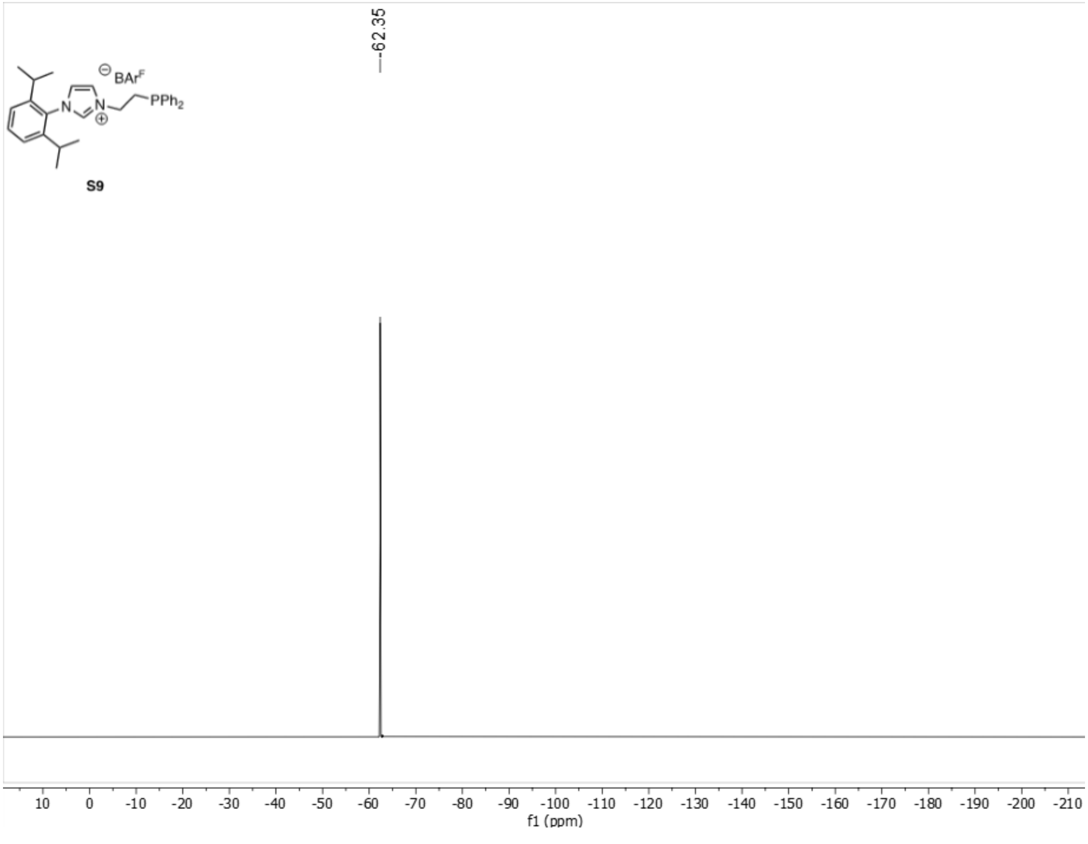

^{11}B NMR (128 MHz, $CDCl_3$)

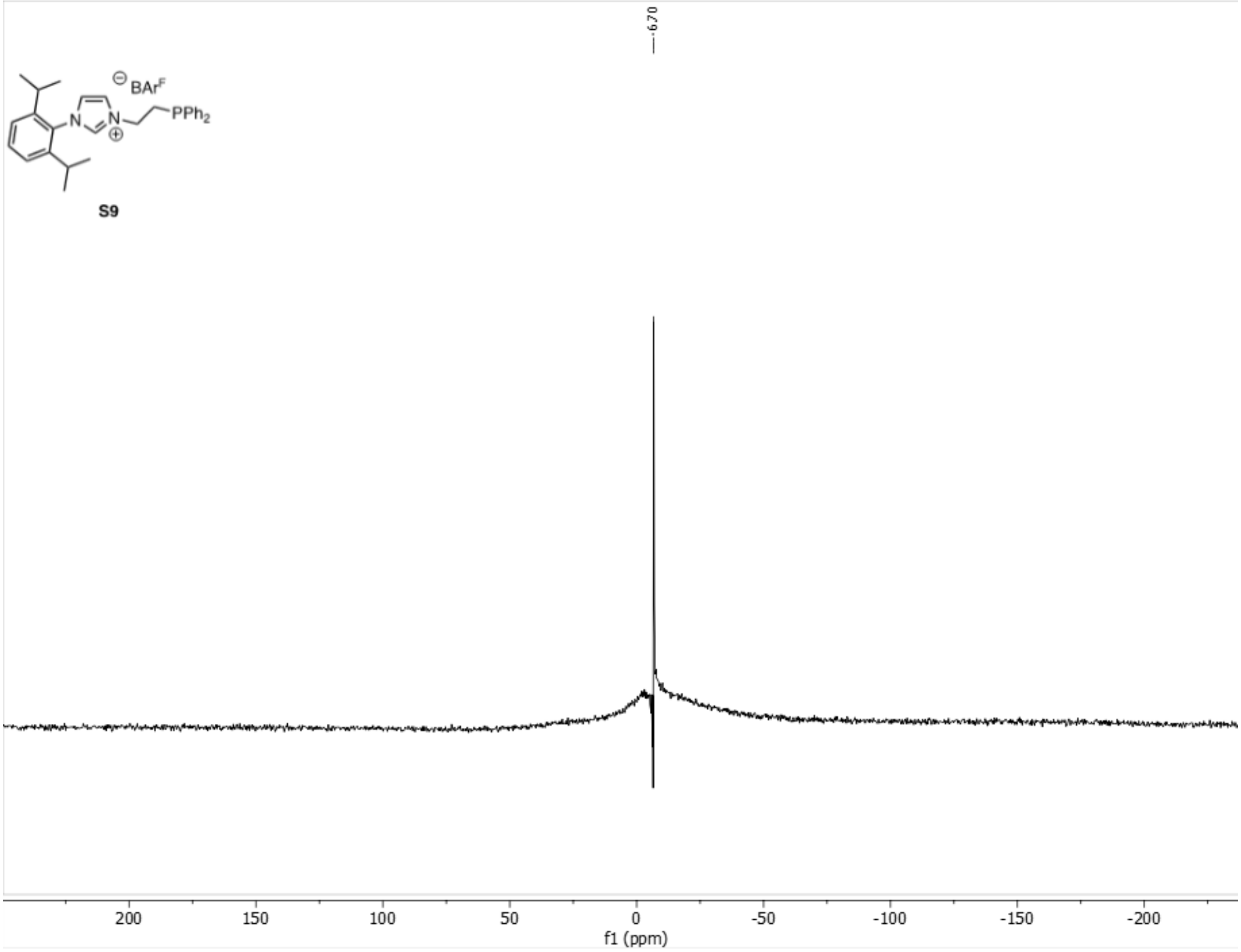

η[4]-Cycloocta-1,5-diene(1-(2,6-diisopropylphenyl)-3-(2-(diphenylphosphanyl)ethyl) imidazole-2-ylidene)iridium BAr^F 8

^{1}H NMR (400 MHz, $CDCl_3$)

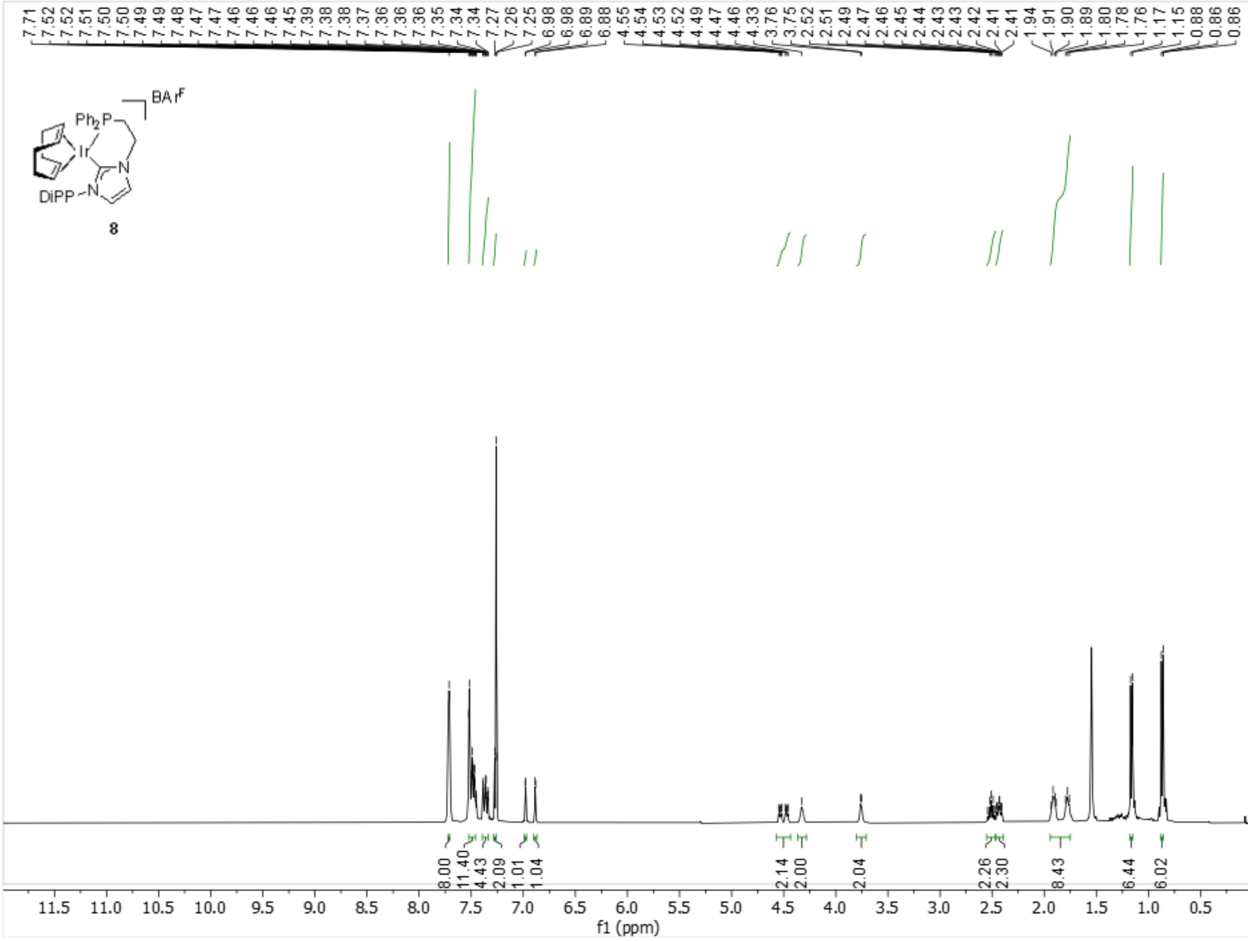

^{13}C NMR (101 MHz, $CDCl_3$):

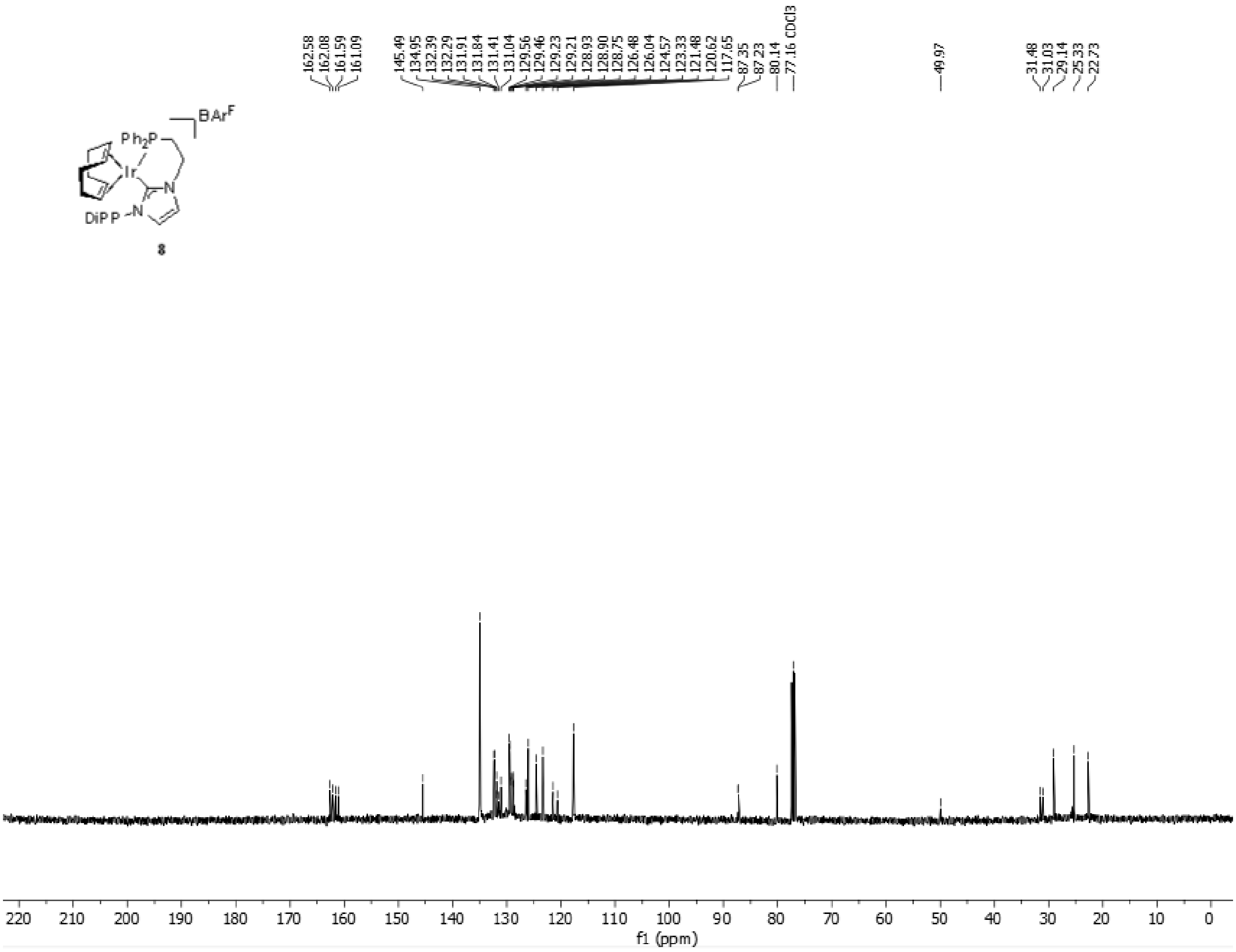

8

^{31}P NMR (162 MHz, $CDCl_3$)

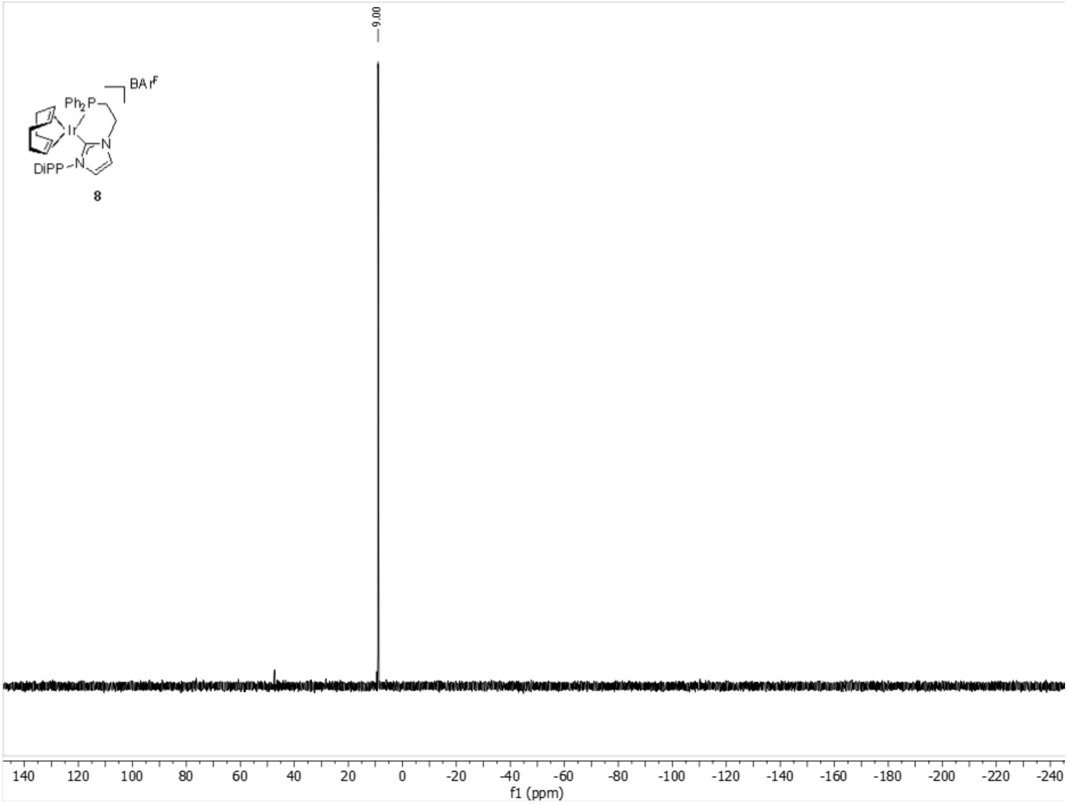

^{19}F NMR (376 MHz, $CDCl_3$)

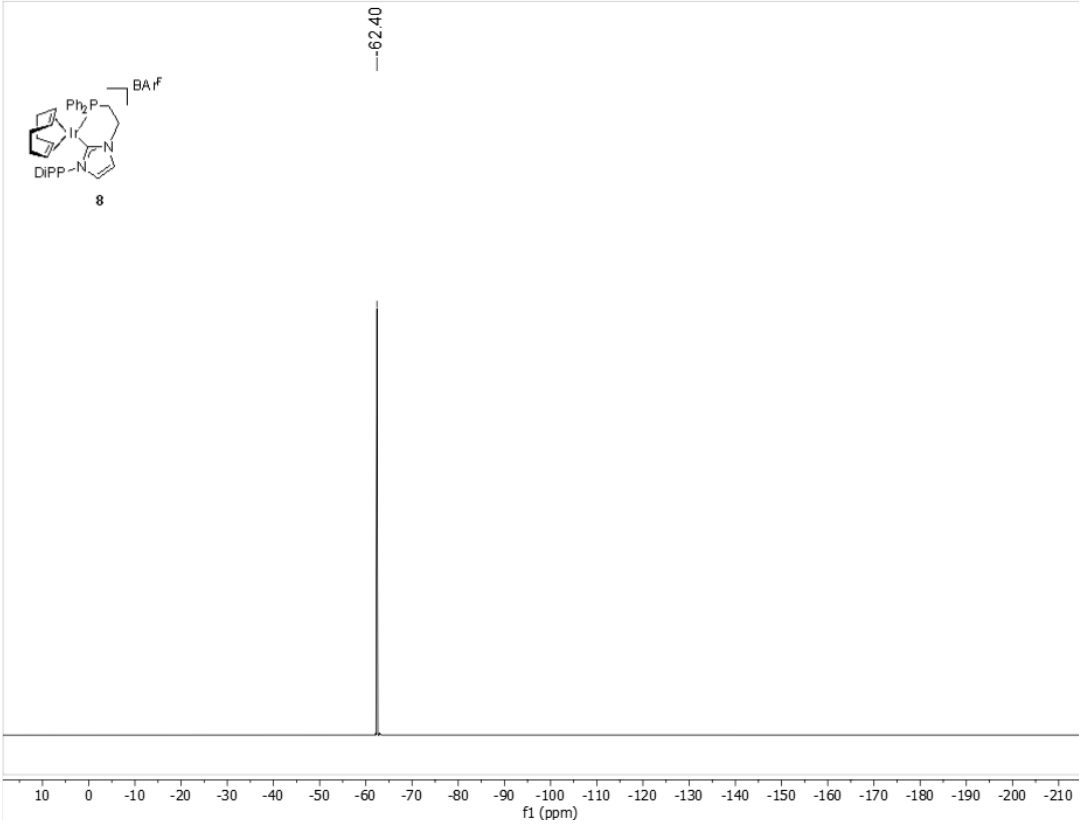

^{11}B NMR (128 MHz, $CDCl_3$)

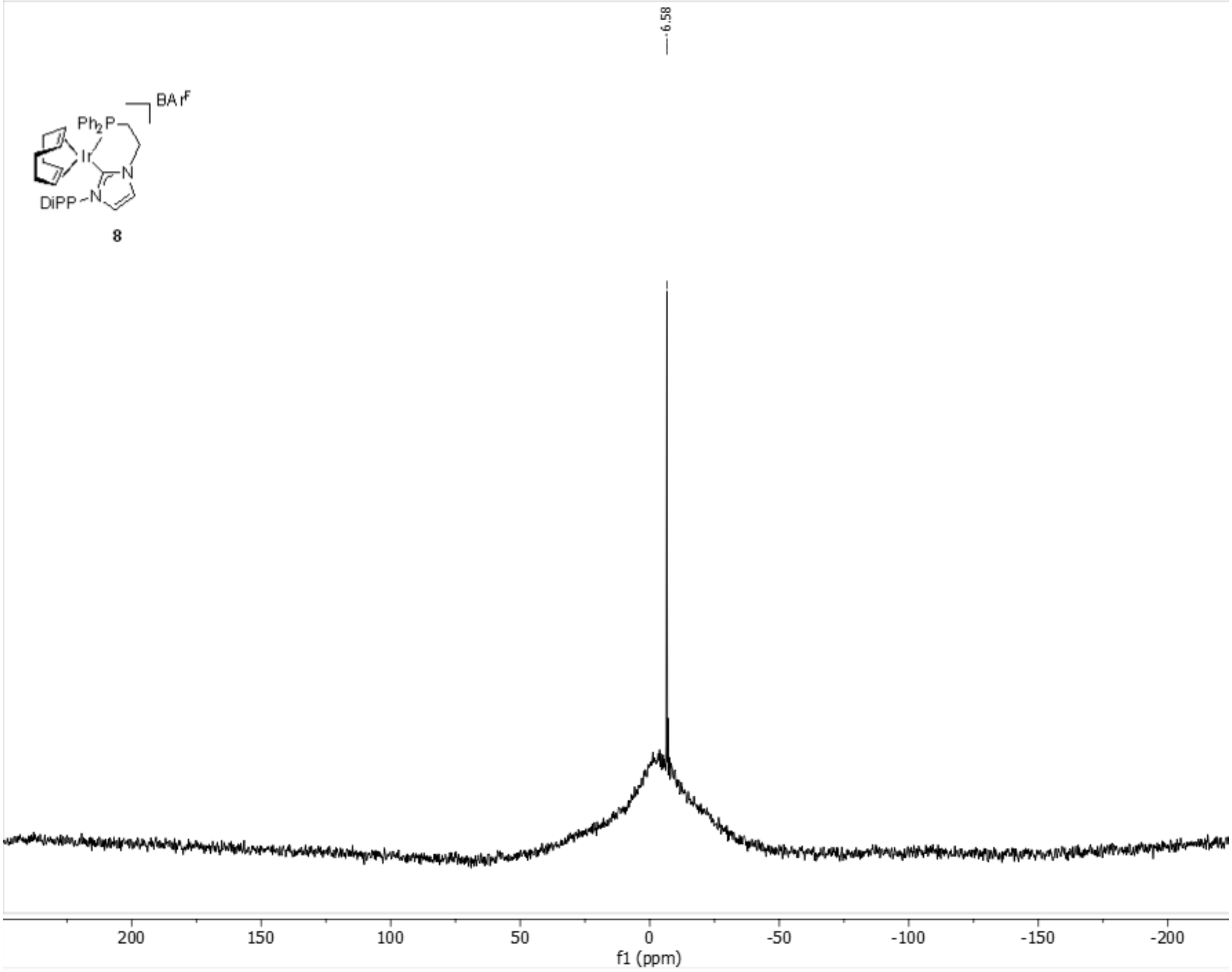

1-(2-(Diphenylphosphanyl)phenyl)-*N*,*N*-dimethylmethanamine S10

^{1}H NMR (400 MHz, $CDCl_3$)

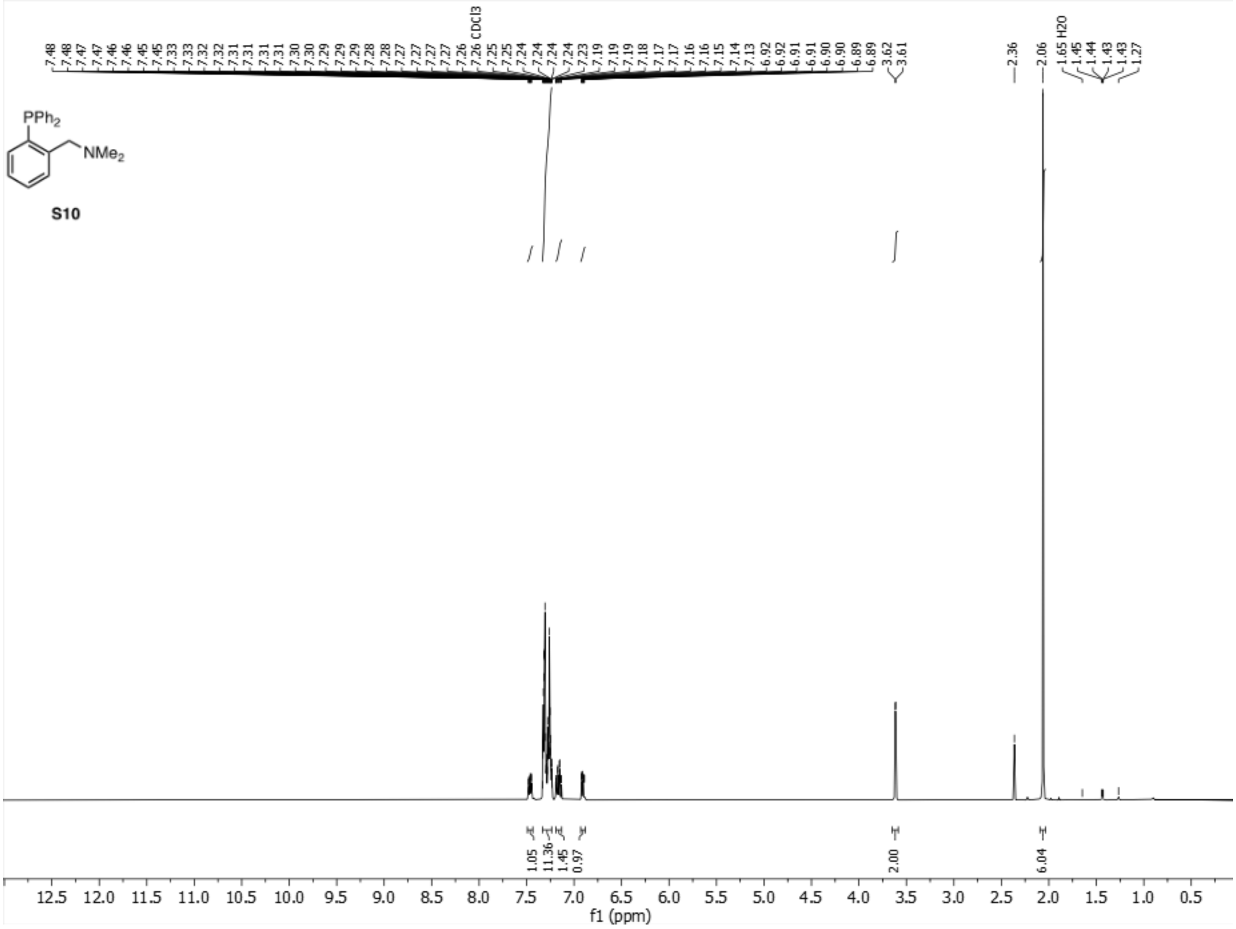

^{13}C NMR (101 MHz, $CDCl_3$)

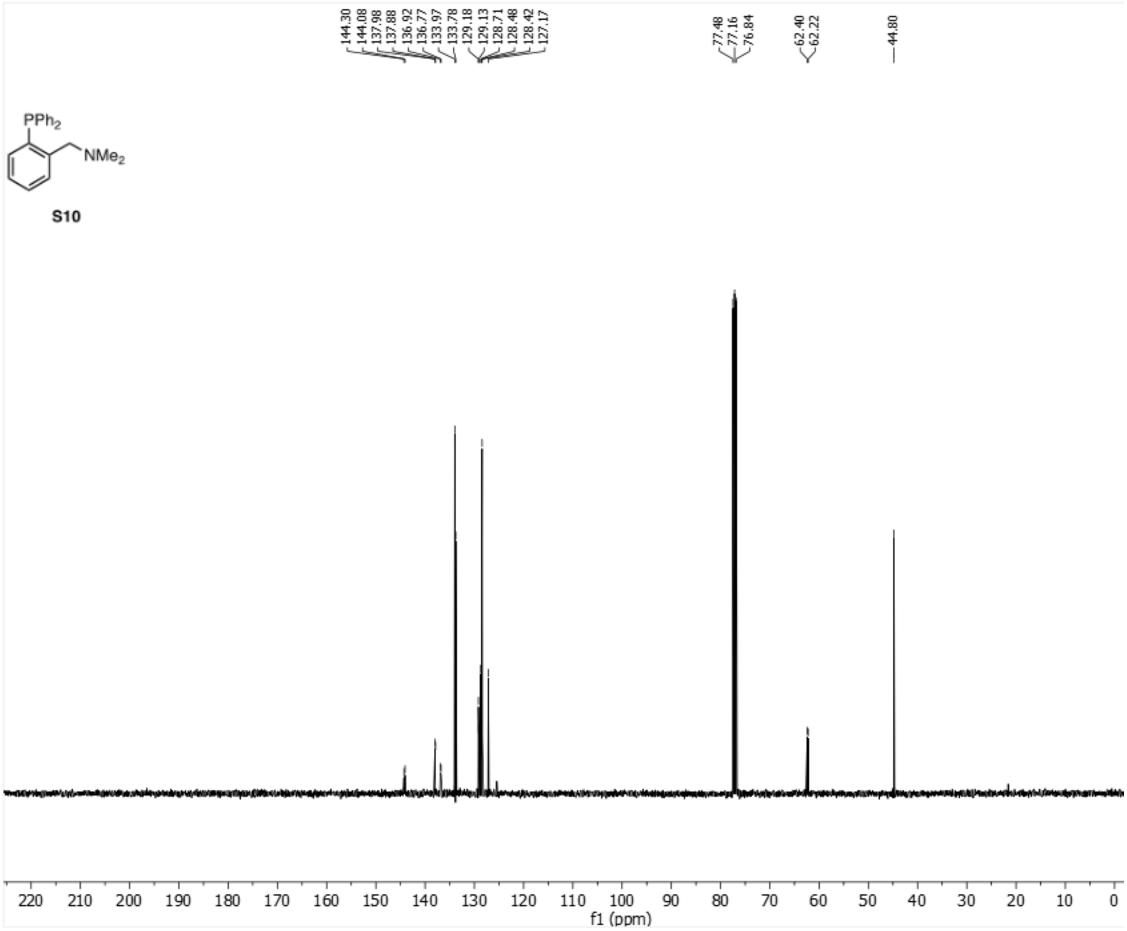

^{31}P NMR (162 MHz, $CDCl_3$)

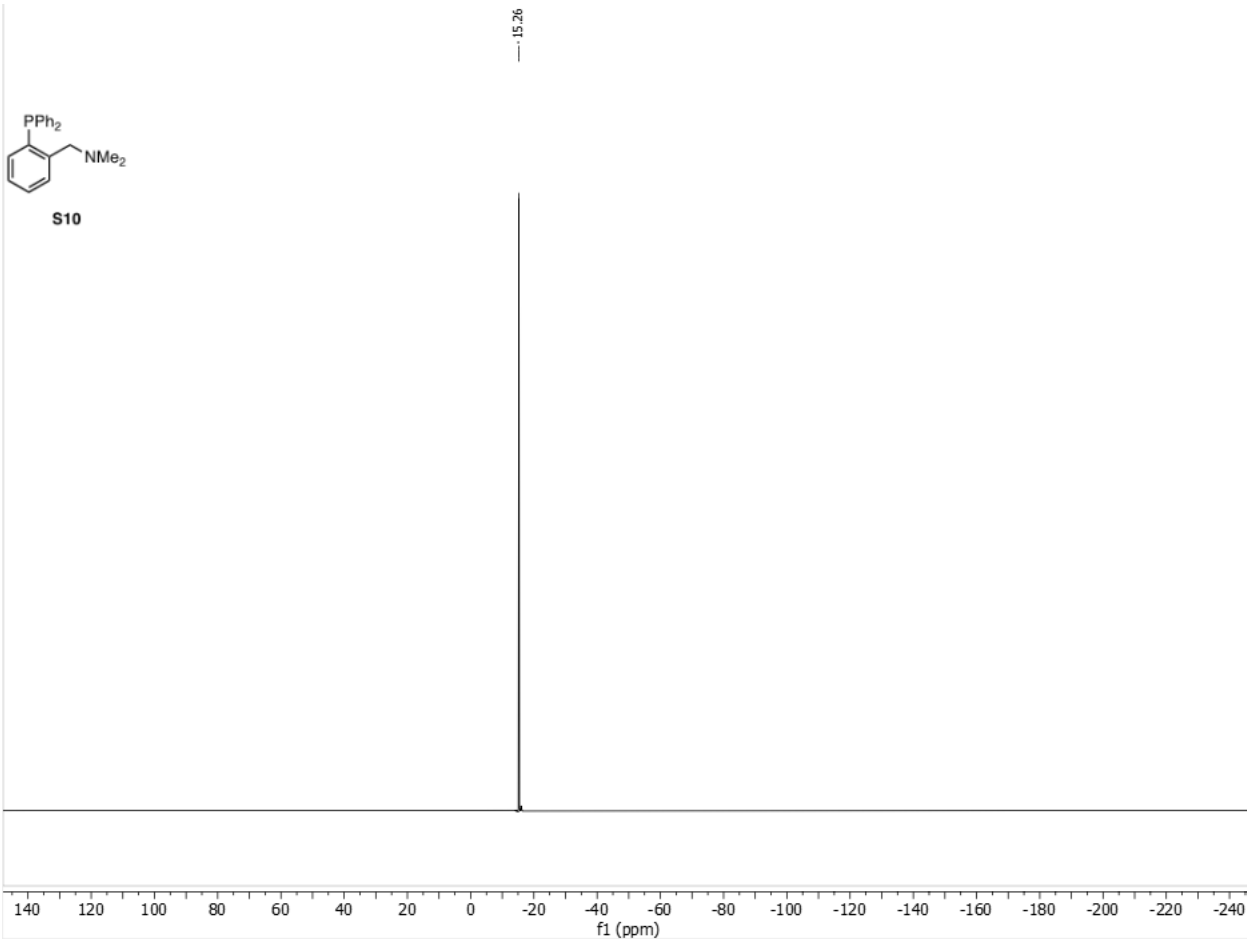

(2-(Chloromethyl)phenyl)diphenylphosphane S11

^1H NMR (400 MHz, $CDCl_3$)

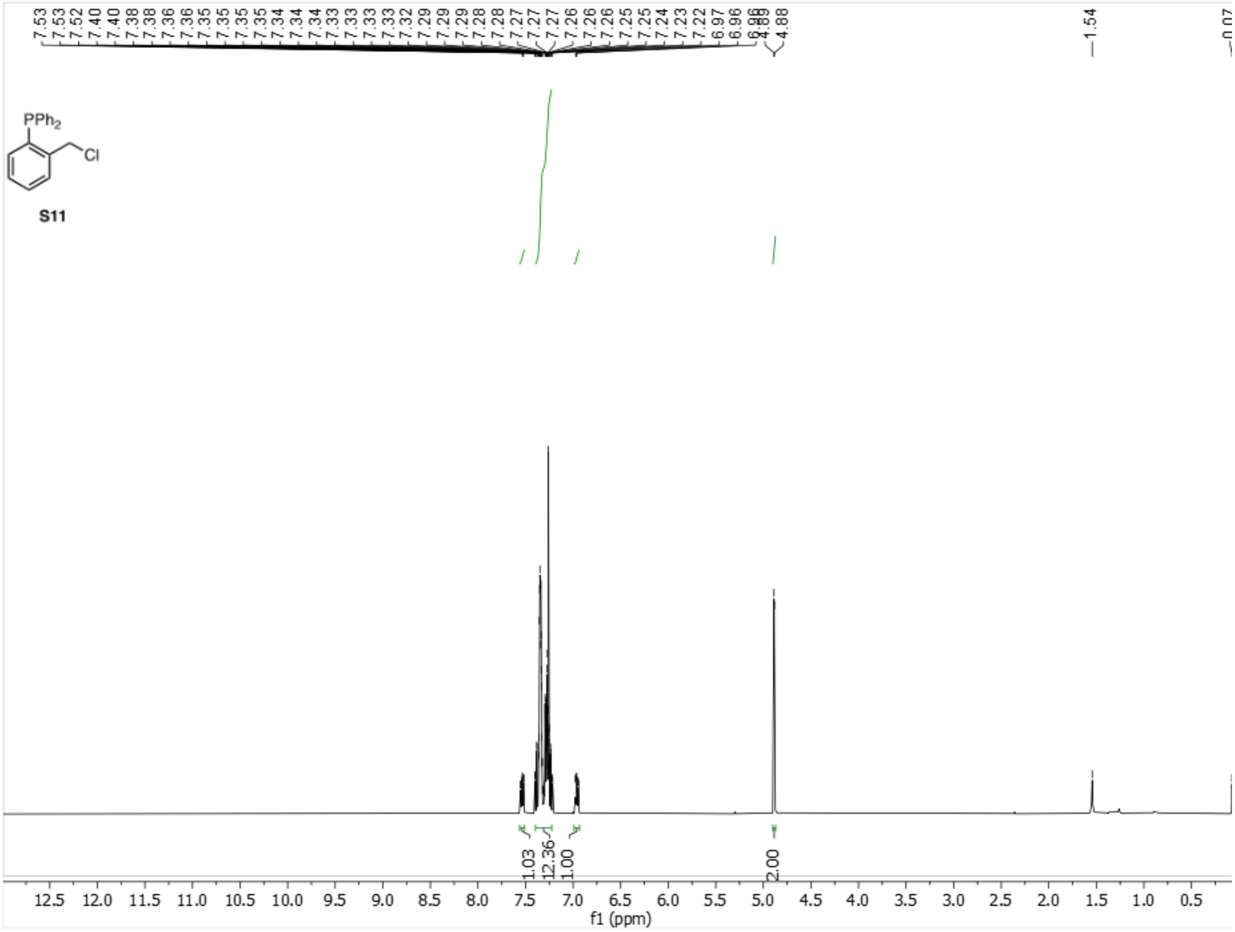

^{13}C NMR (101 MHz, $CDCl_3$)

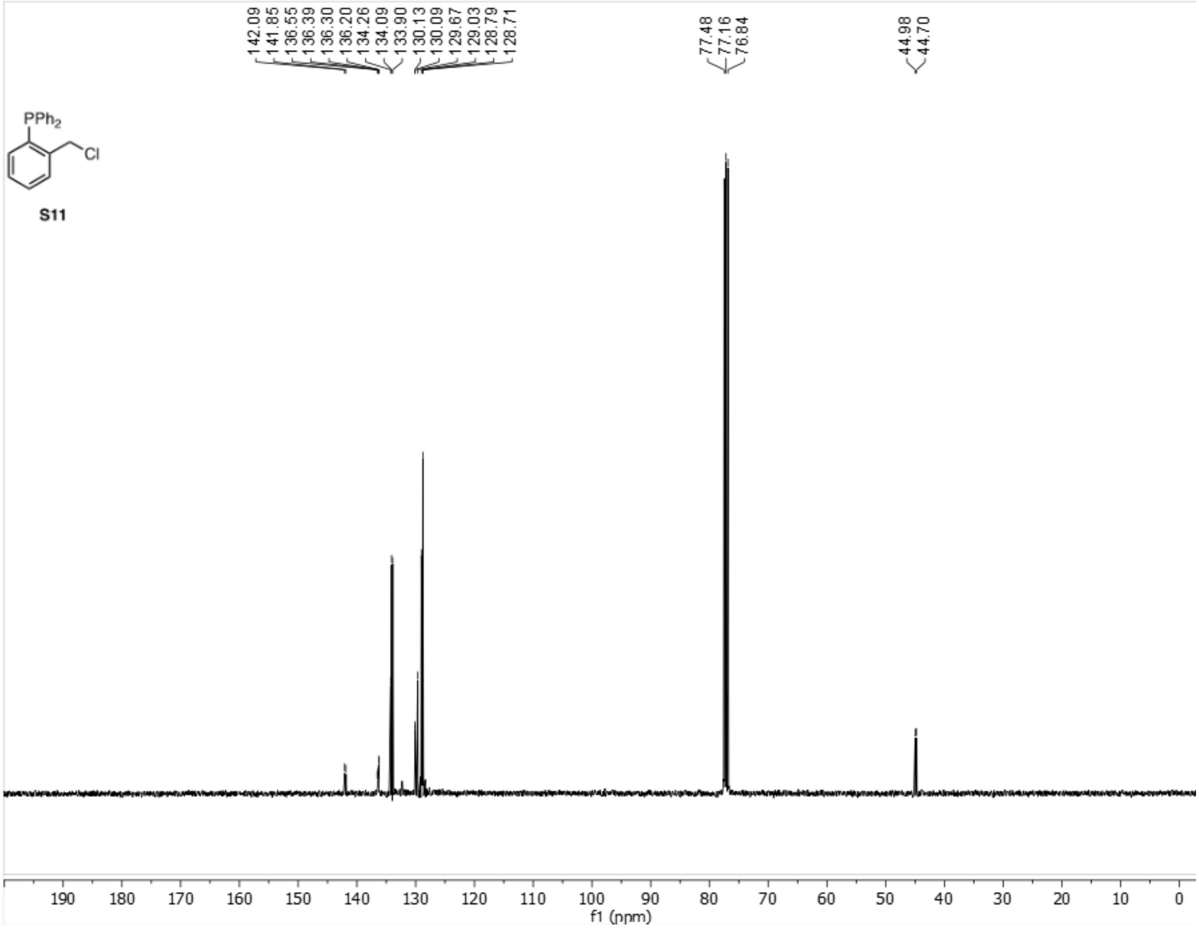

^{31}P NMR (162 MHz, $CDCl_3$)

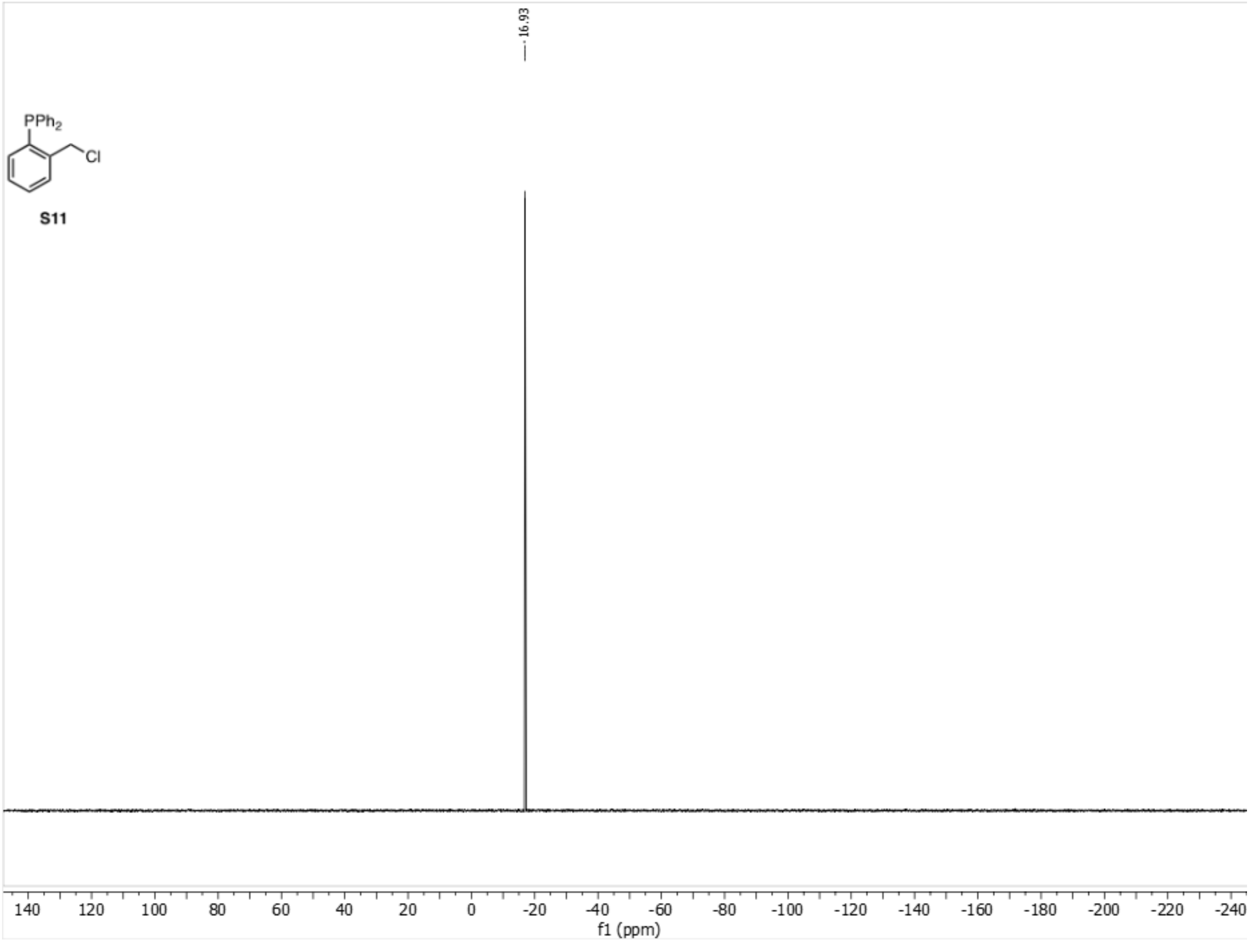

1-(2,6-Diisopropylphenyl)-3-(2-(diphenylphosphanyl)benzyl)-1*H*-imidazol-3-ium BAr^F S12

^{1}H NMR (400 MHz, $CDCl_3$)

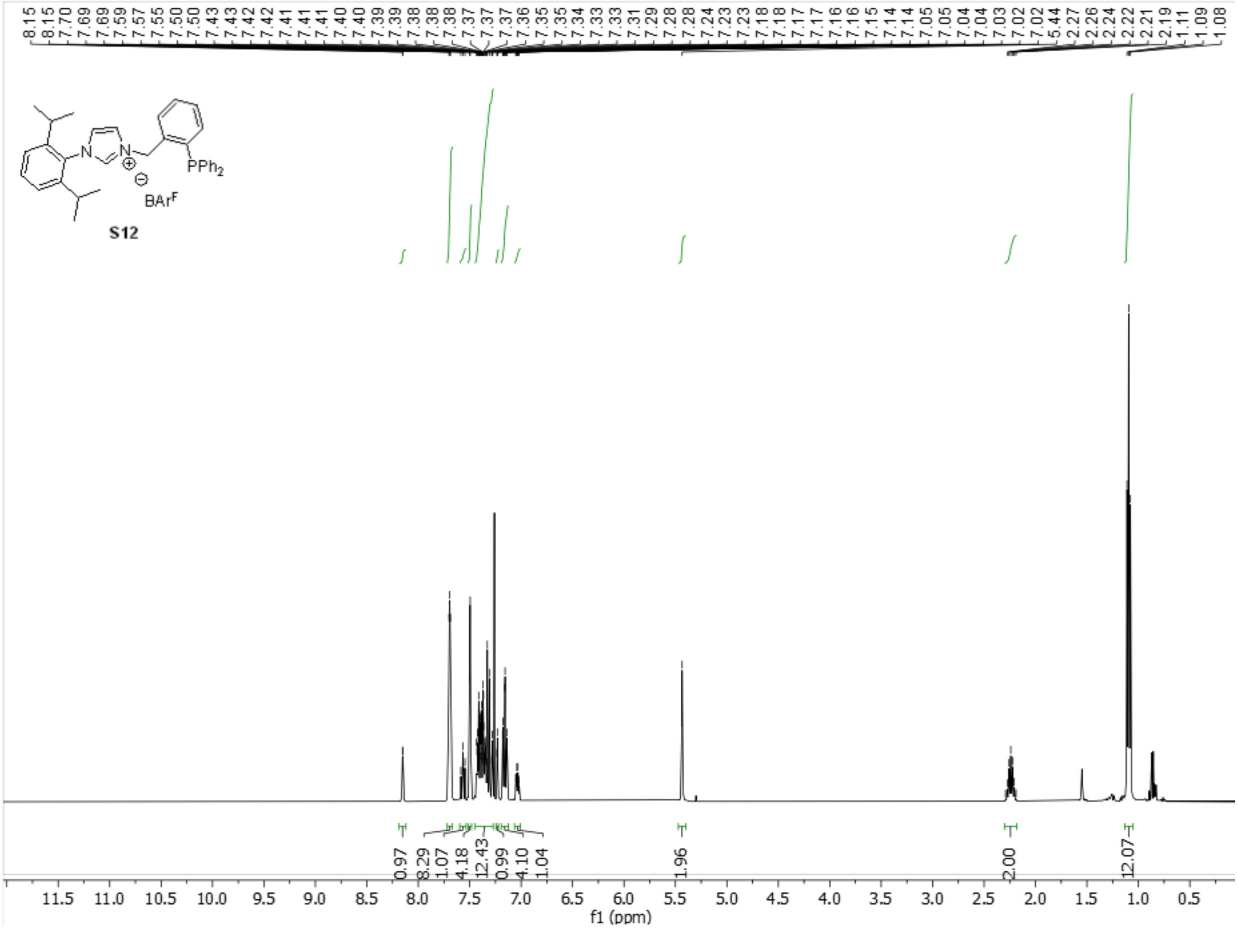

^{13}C NMR (101 MHz, $CDCl_3$)

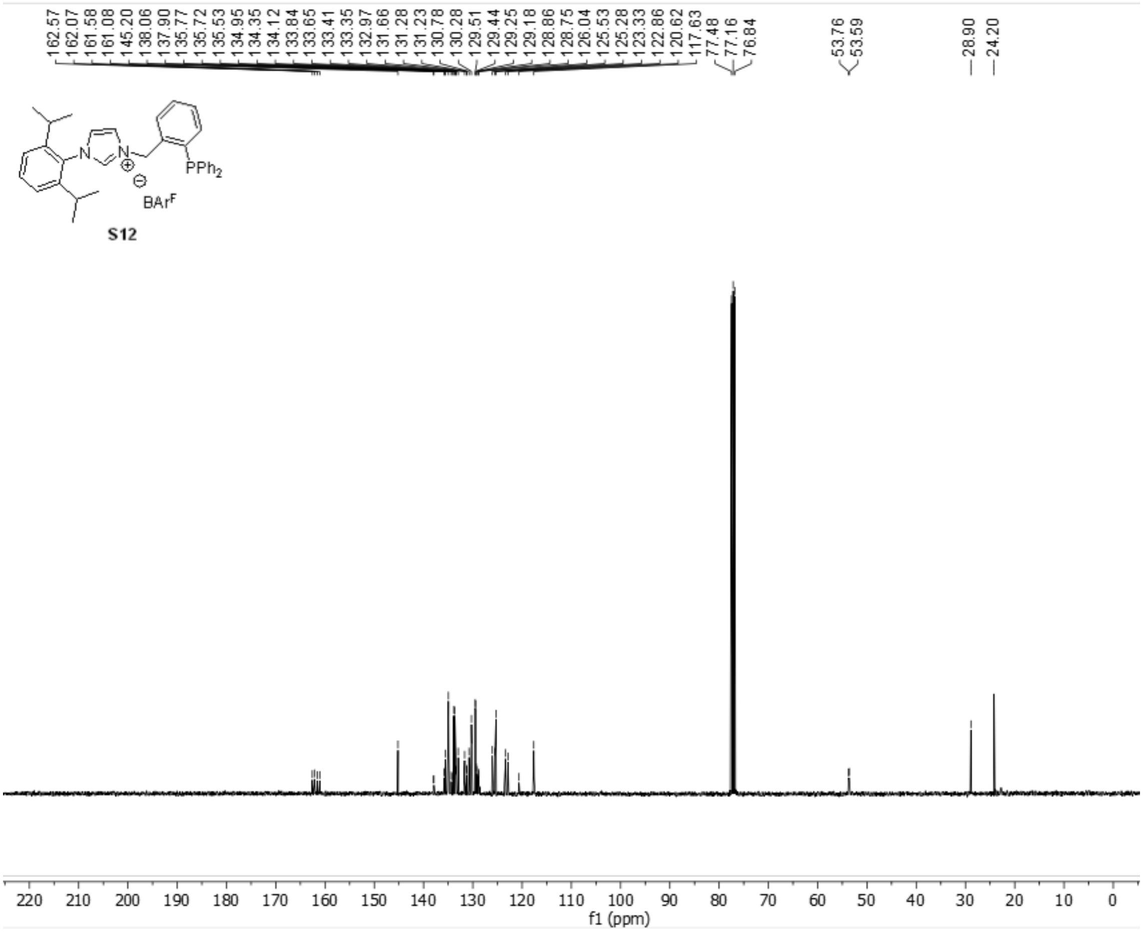

^{31}P NMR (162 MHz, $CDCl_3$)

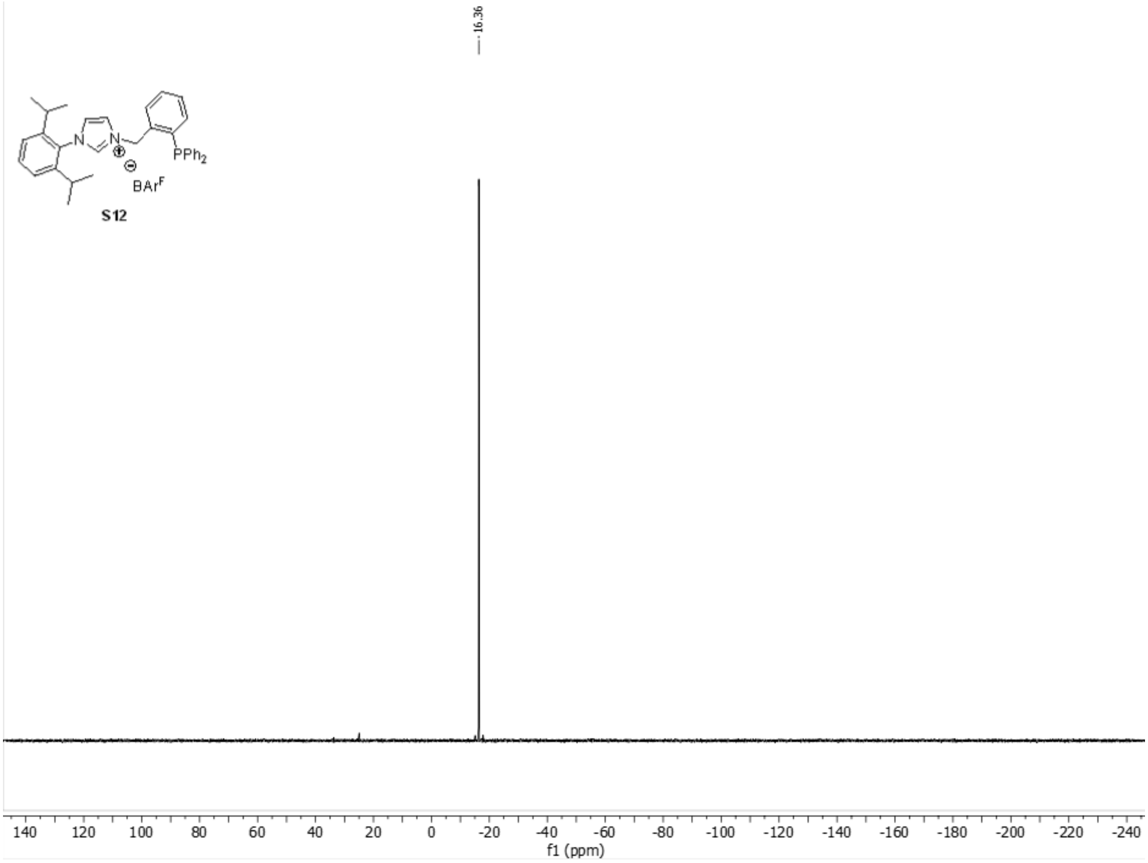

^{19}F NMR (376 MHz, $CDCl_3$)

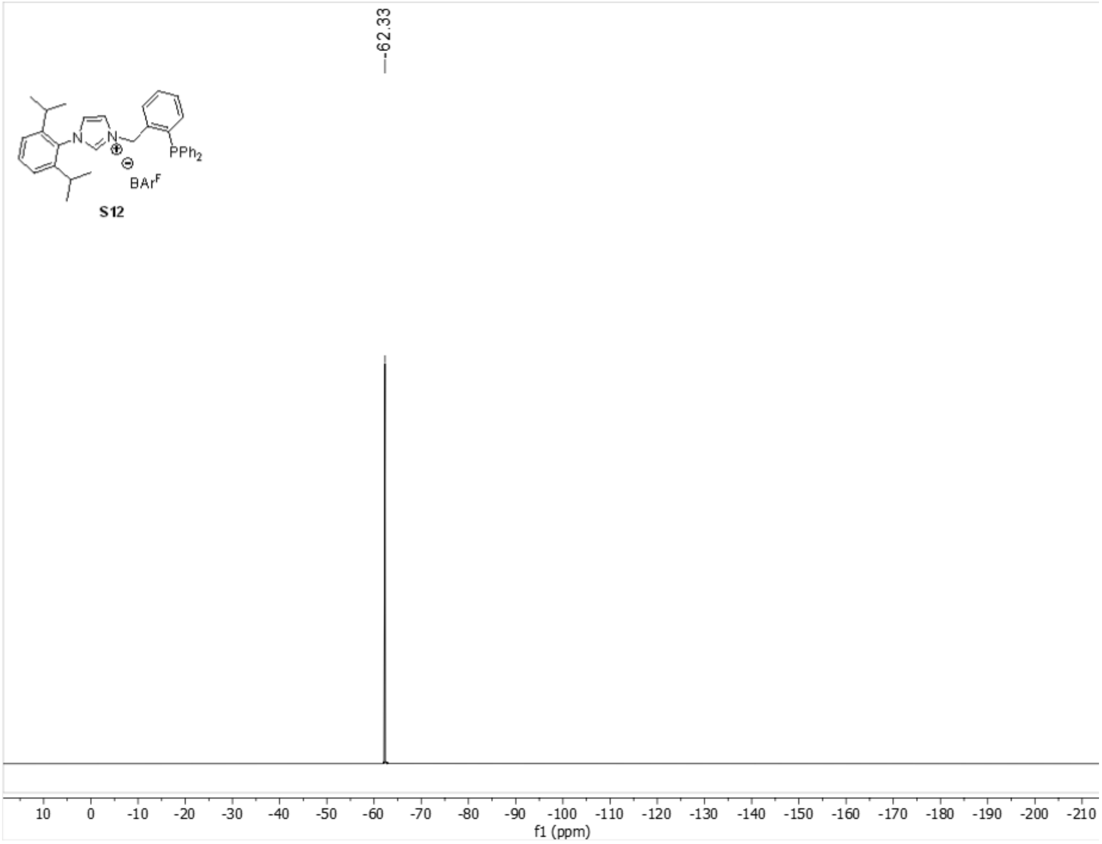

^{11}B NMR (128 MHz, $CDCl_3$)

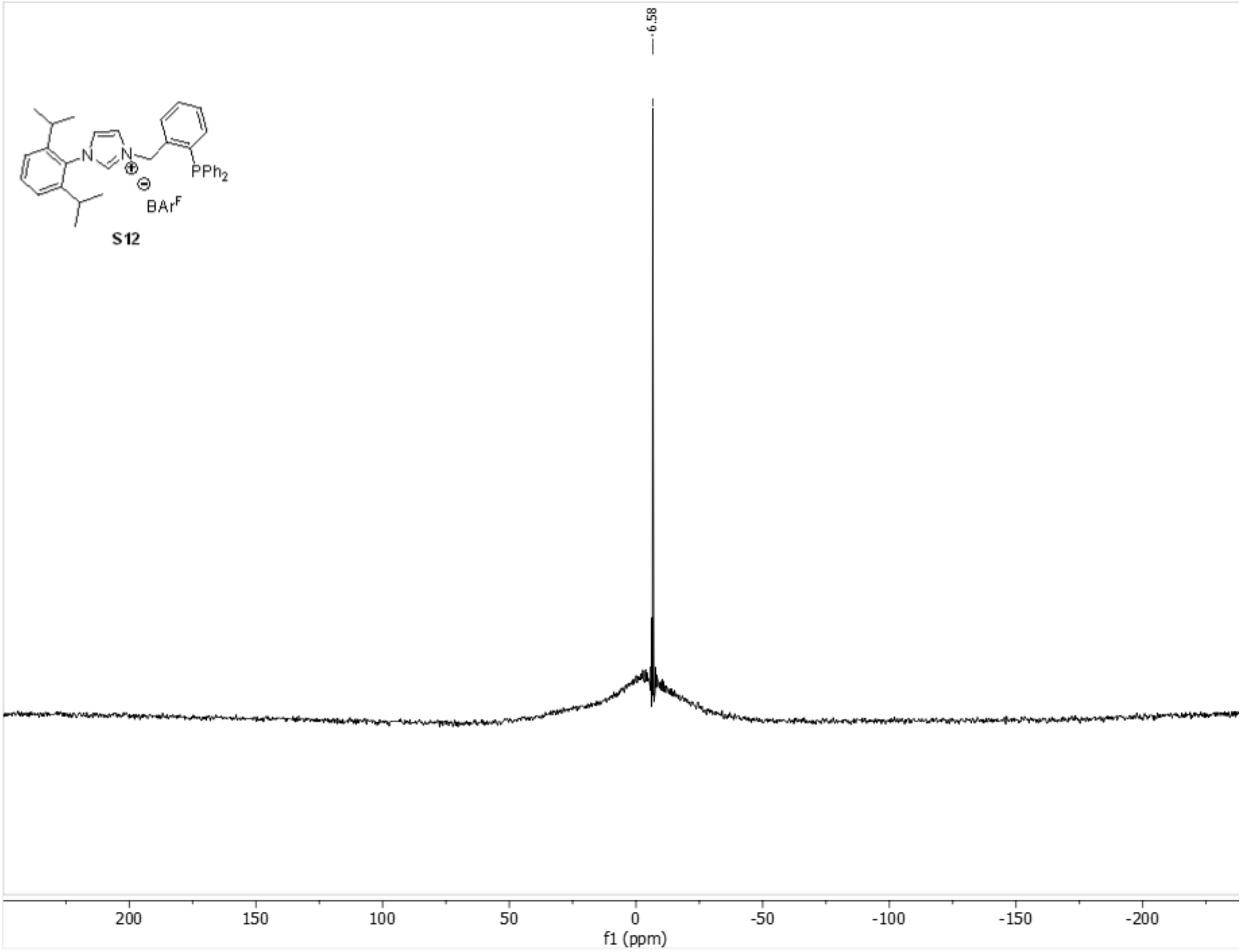

η⁴-Cycloocta-1,5-diene(1-(2,6-diisopropylphenyl)-3-(2-(diphenylphosphanyl)benzyl) imidazole-2-ylidene)iridium BArF 2

^{1}H NMR (400 MHz, $CDCl_3$)

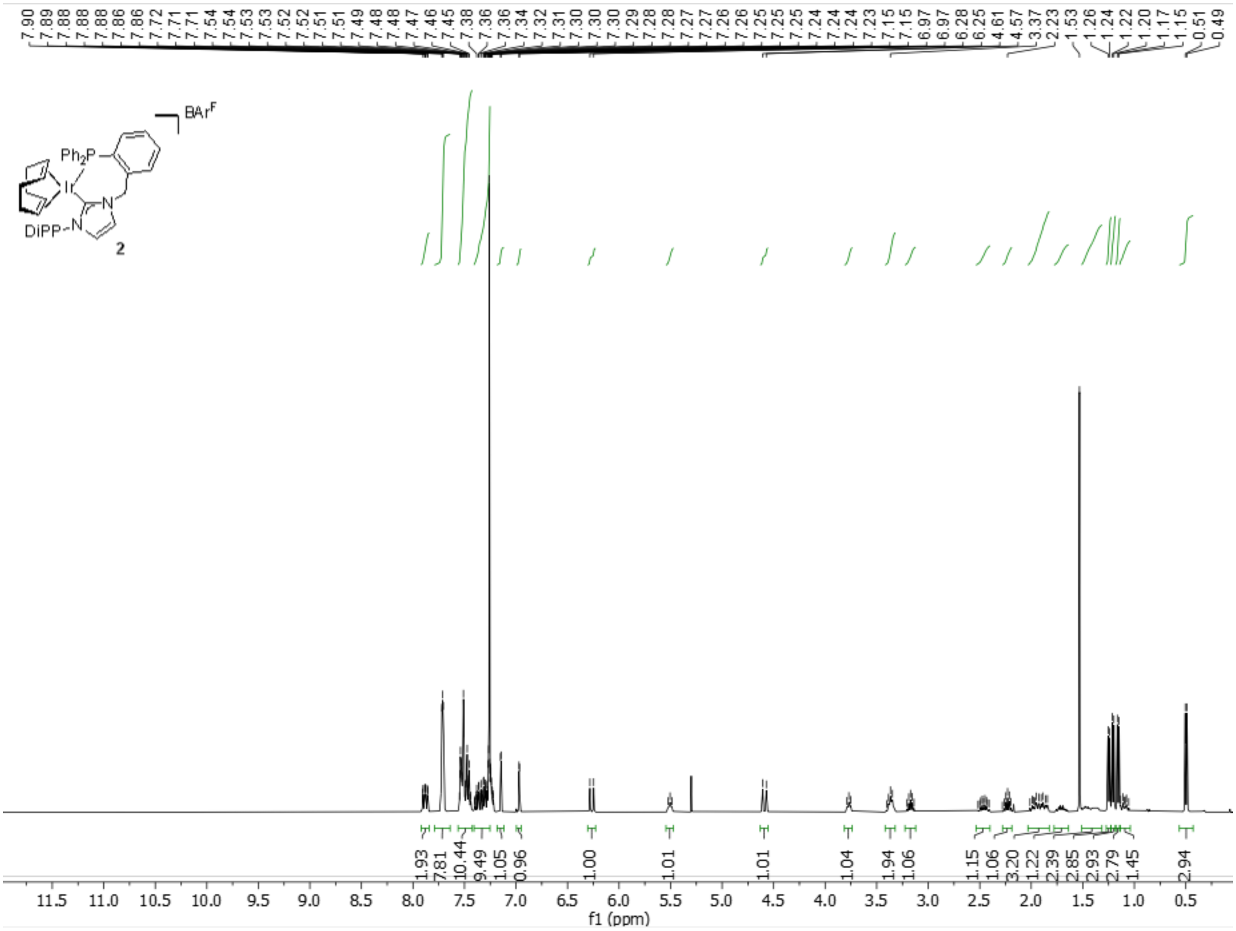

^{13}C NMR (101 MHz, $CDCl_3$)

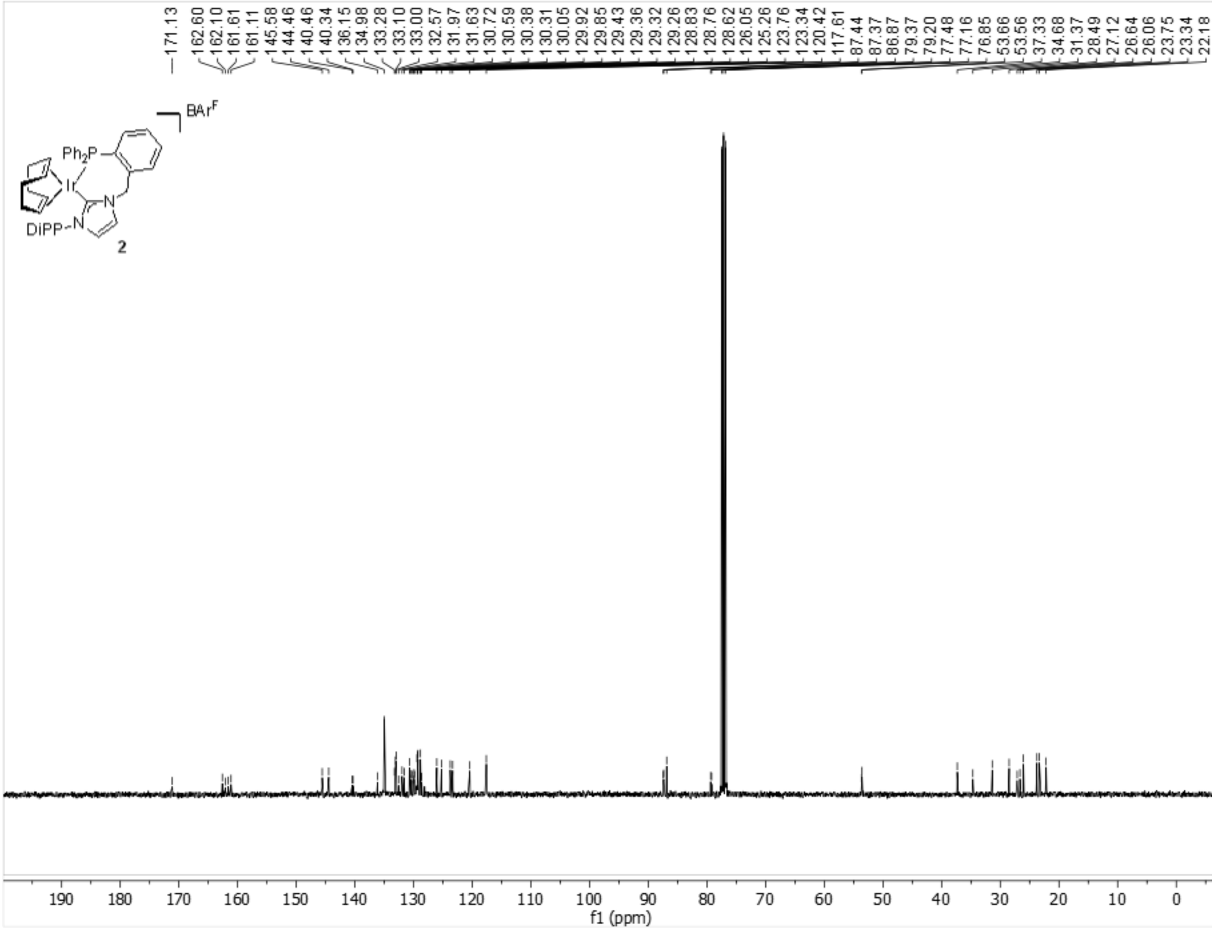

^{31}P NMR (162 MHz, $CDCl_3$)

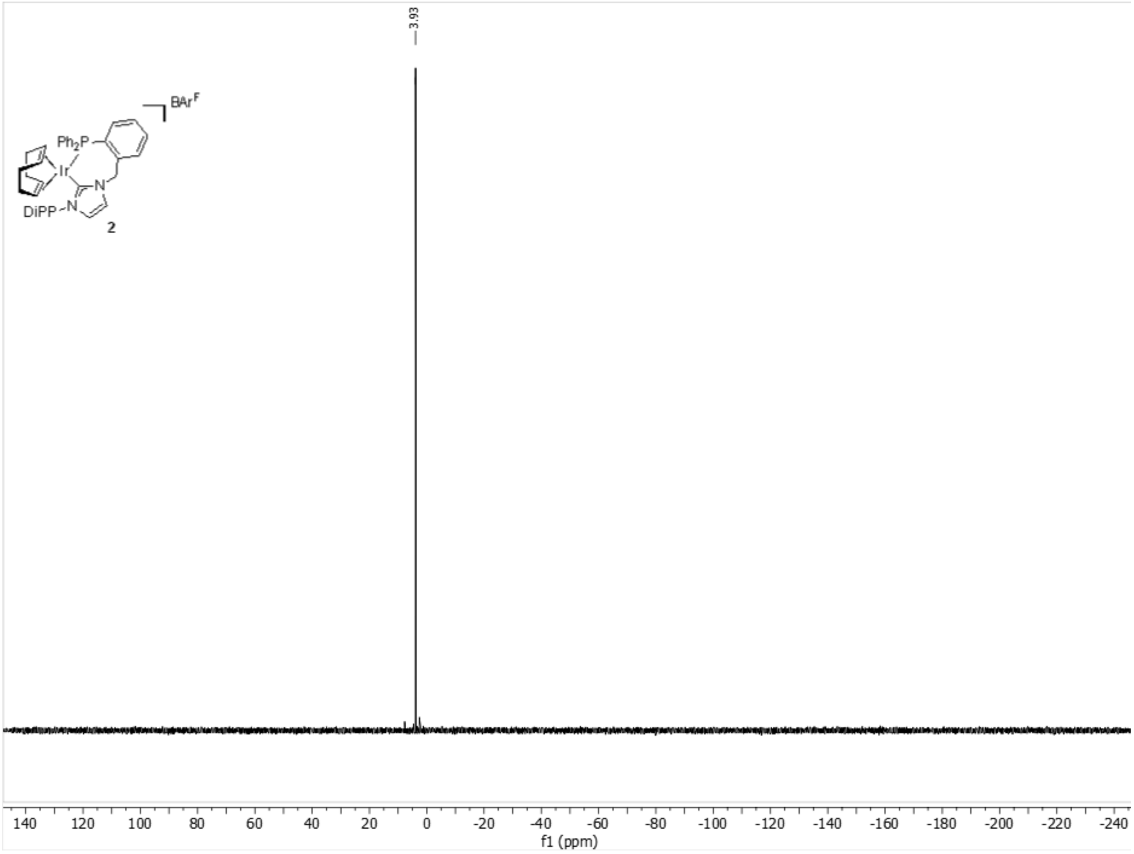

^{19}F NMR (376 MHz, $CDCl_3$)

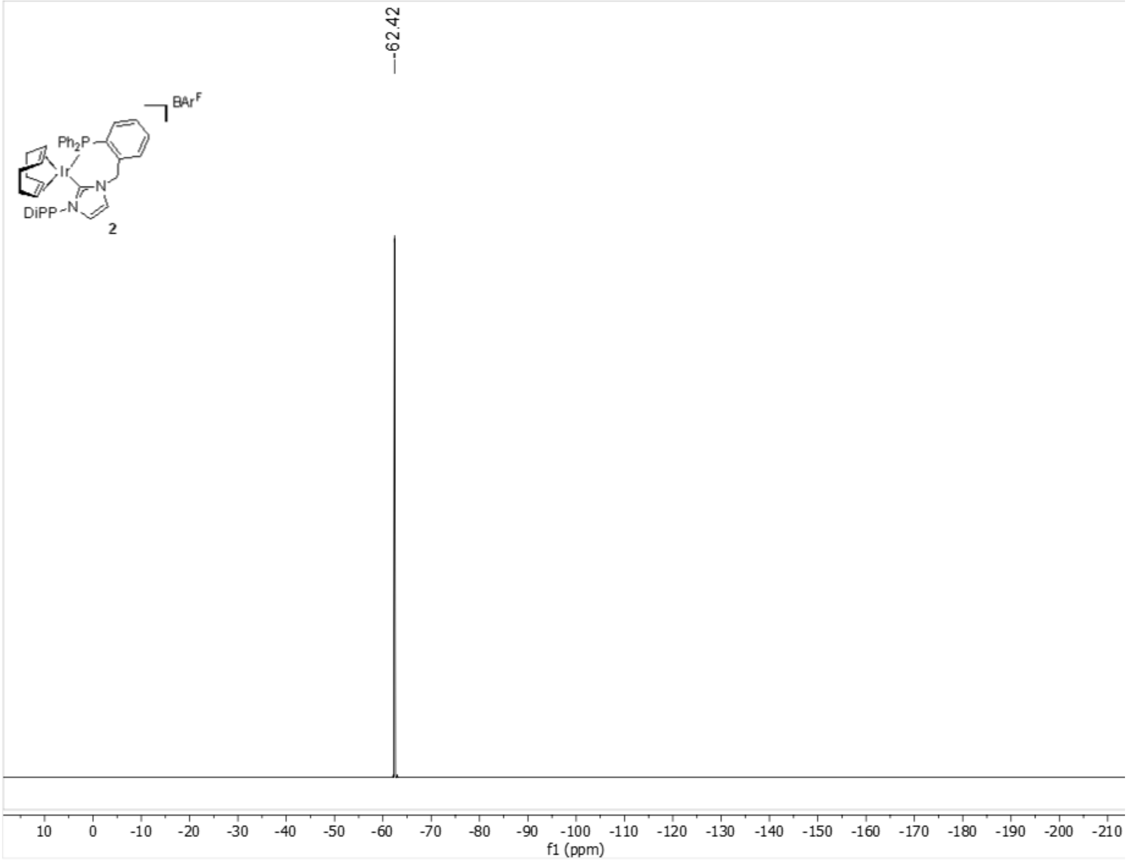

^{11}B NMR (128 MHz, $CDCl_3$)

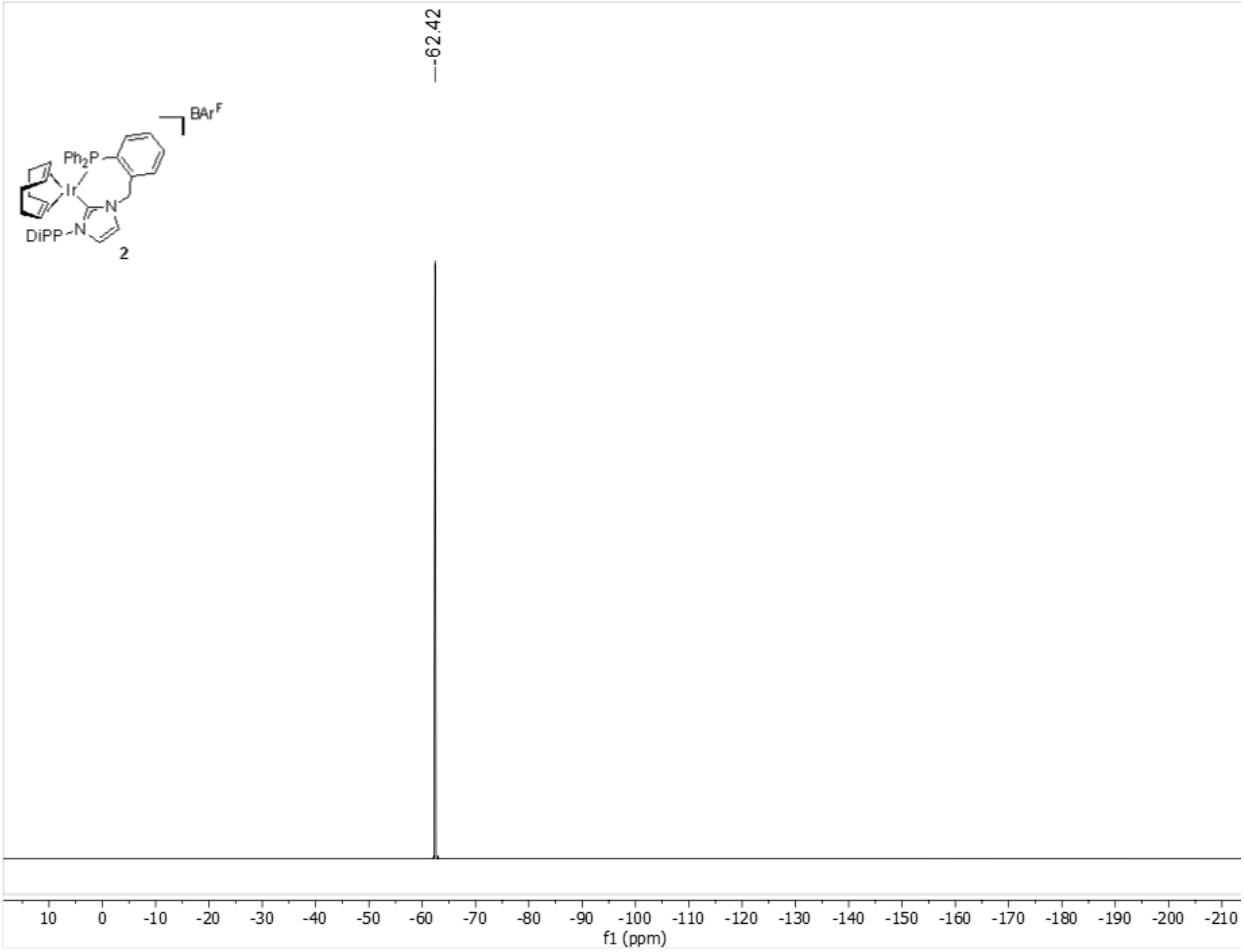

2-Phenyl-1,3,2-dioxaborolane 11

^1H NMR (400 MHz, $CDCl_3$)

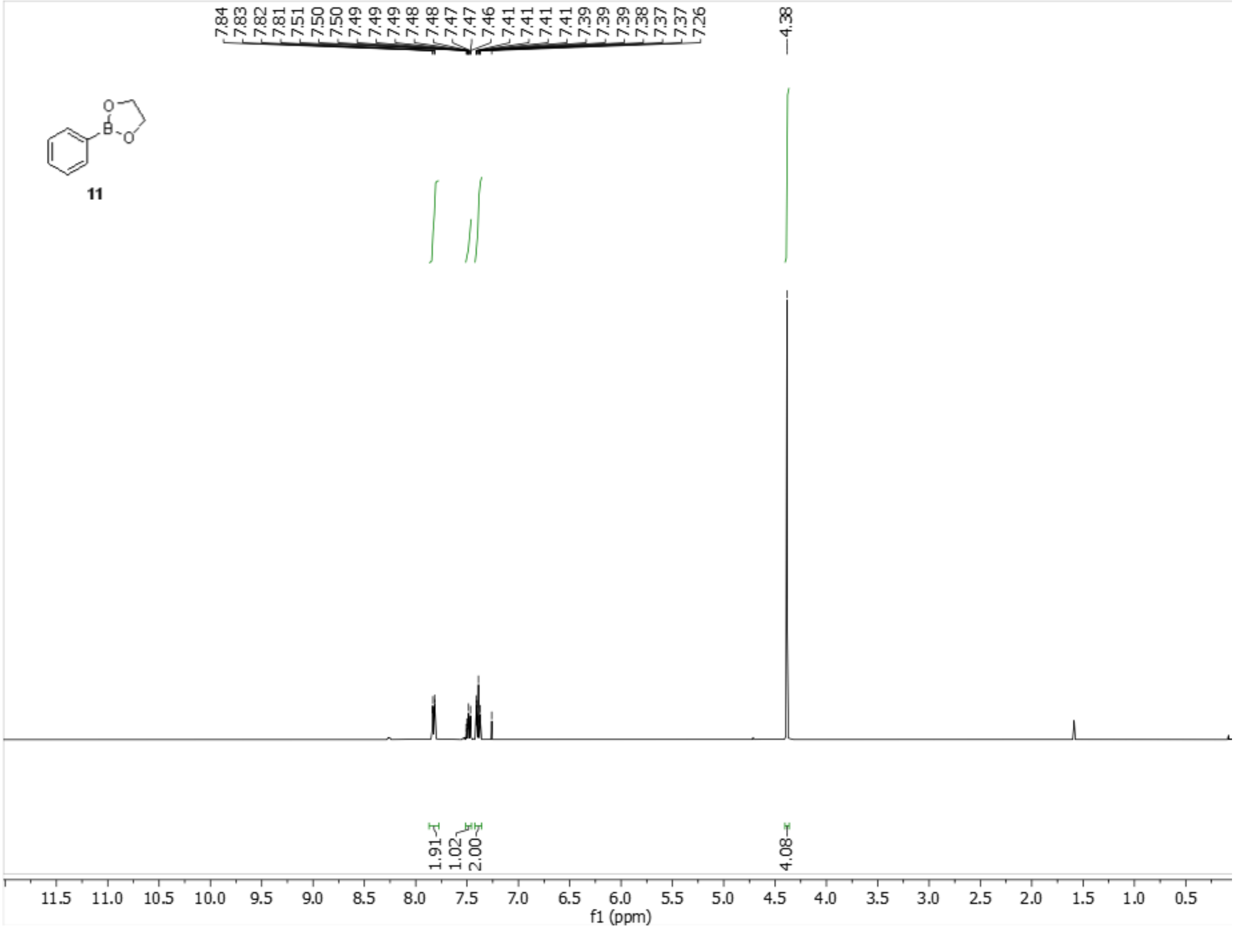

^{13}C NMR (101 MHz, $CDCl_3$)

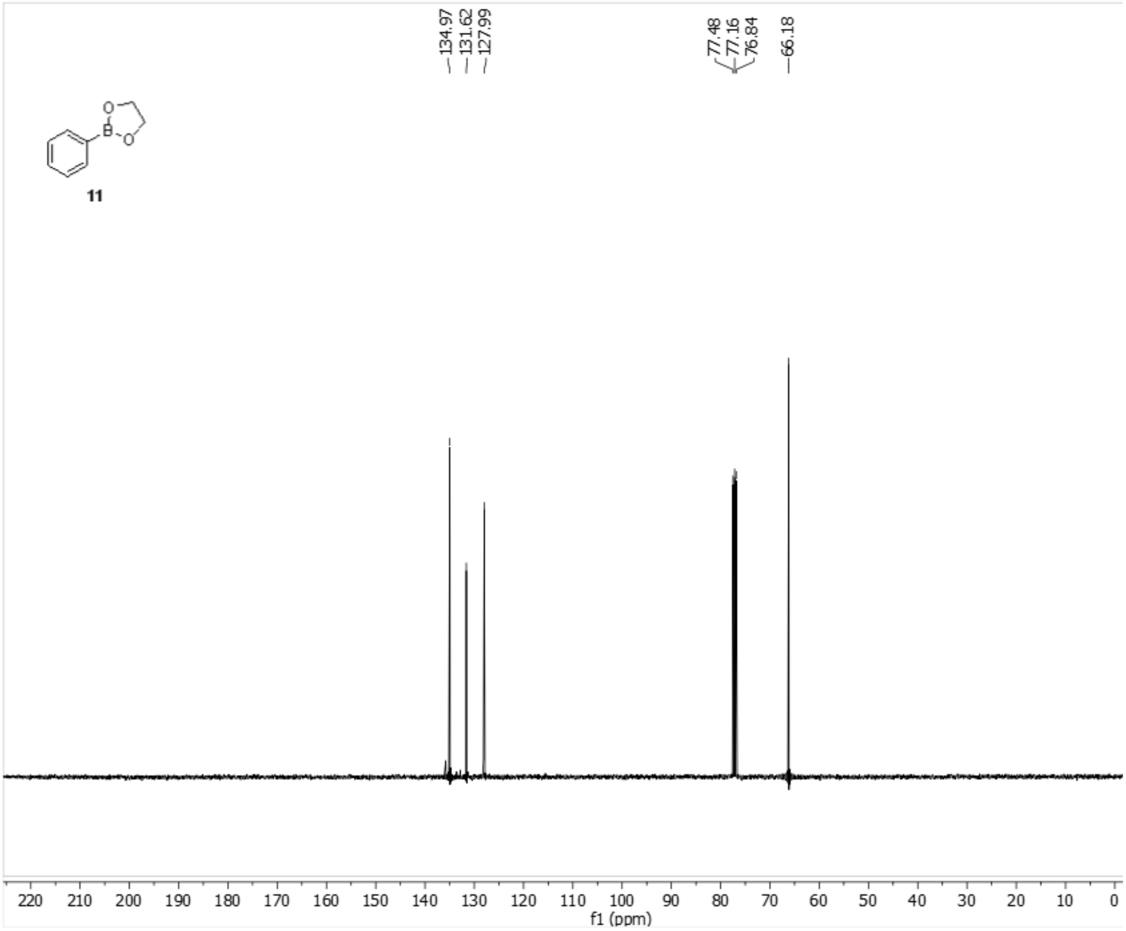

^{11}B NMR (128 MHz, $CDCl_3$)

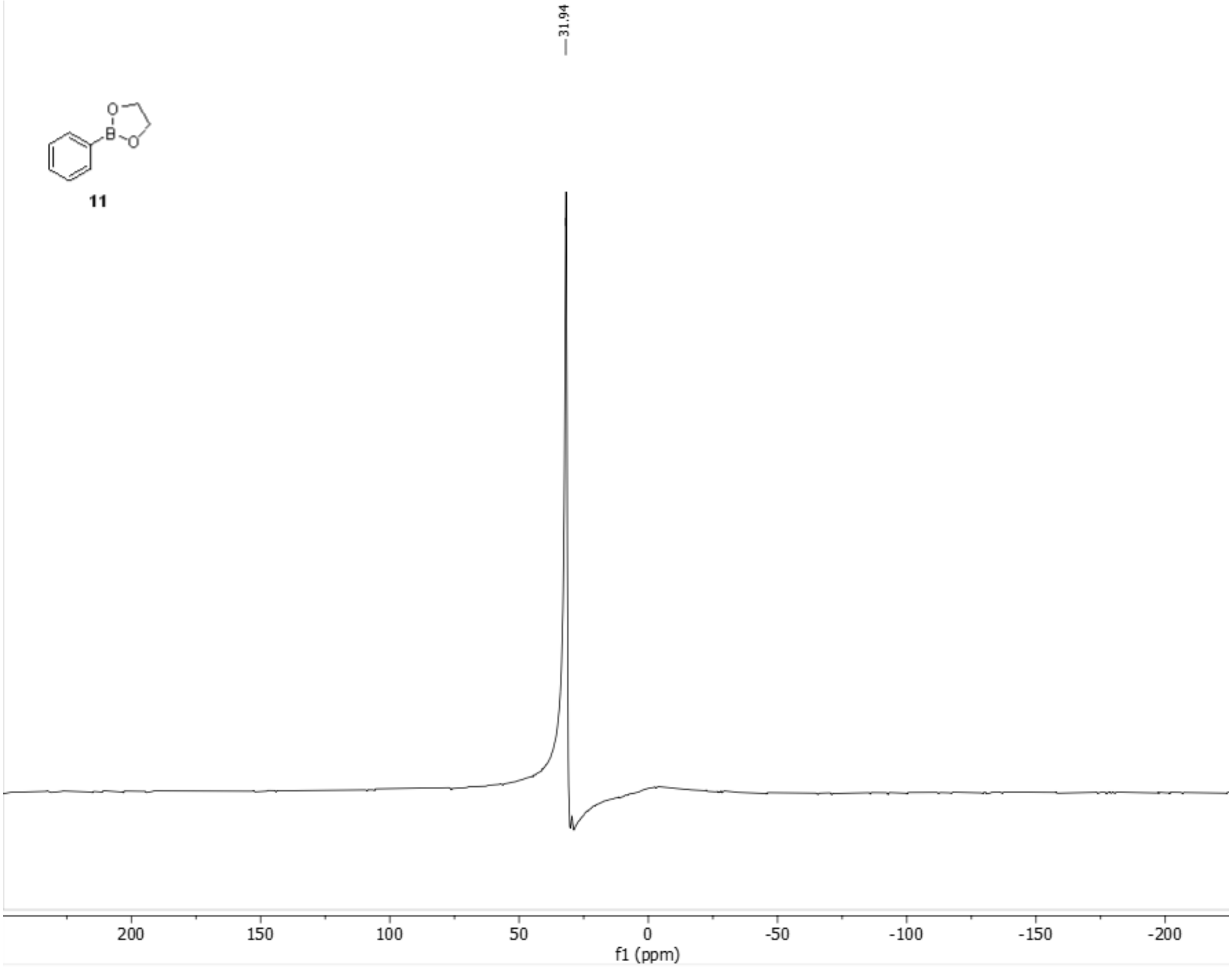

4,4,5,5-Tetramethyl-2-phenyl-1,3,2-dioxaborolane 12

^{1}H NMR (500 MHz, $CDCl_3$)

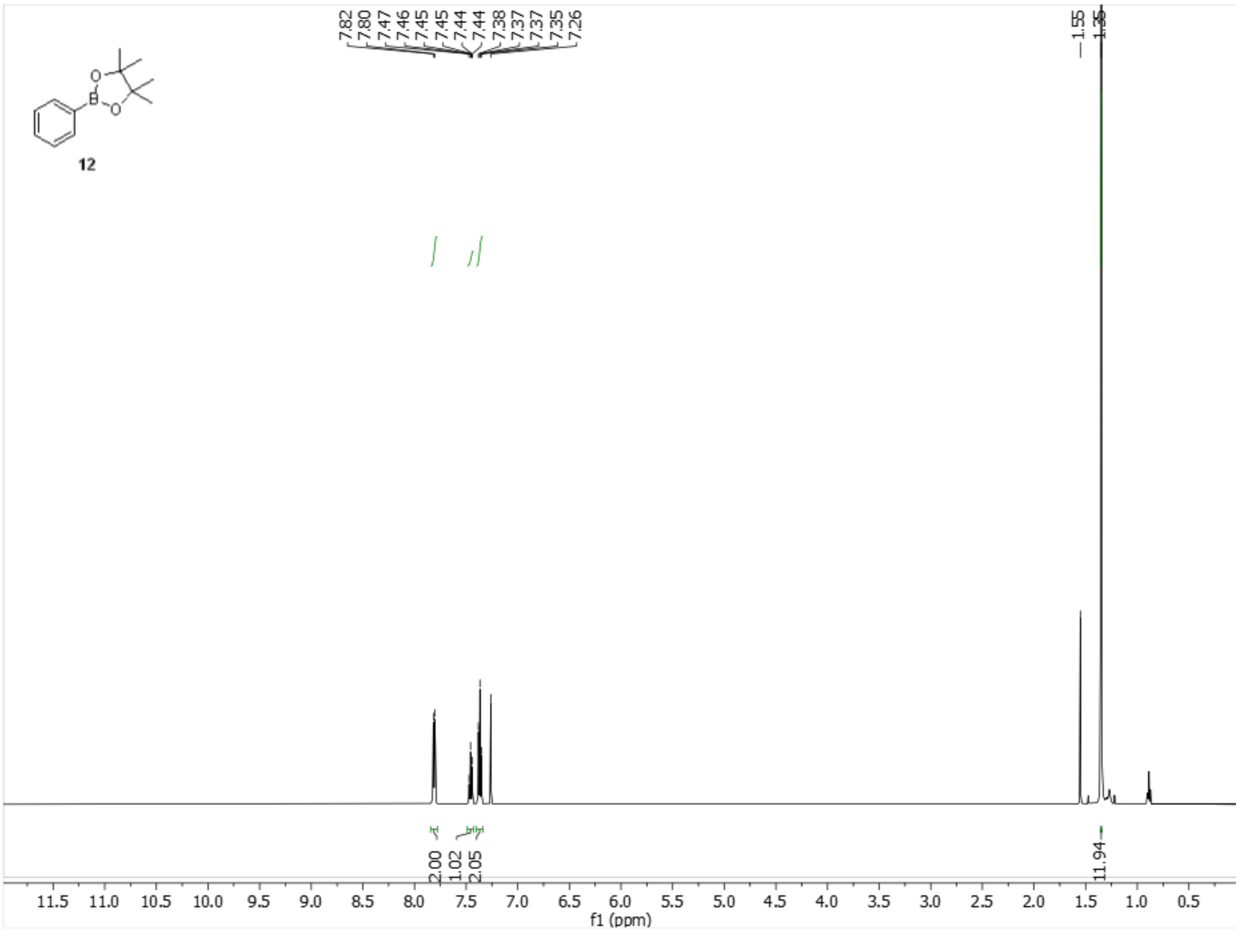

^{13}C NMR (126 MHz, $CDCl_3$)

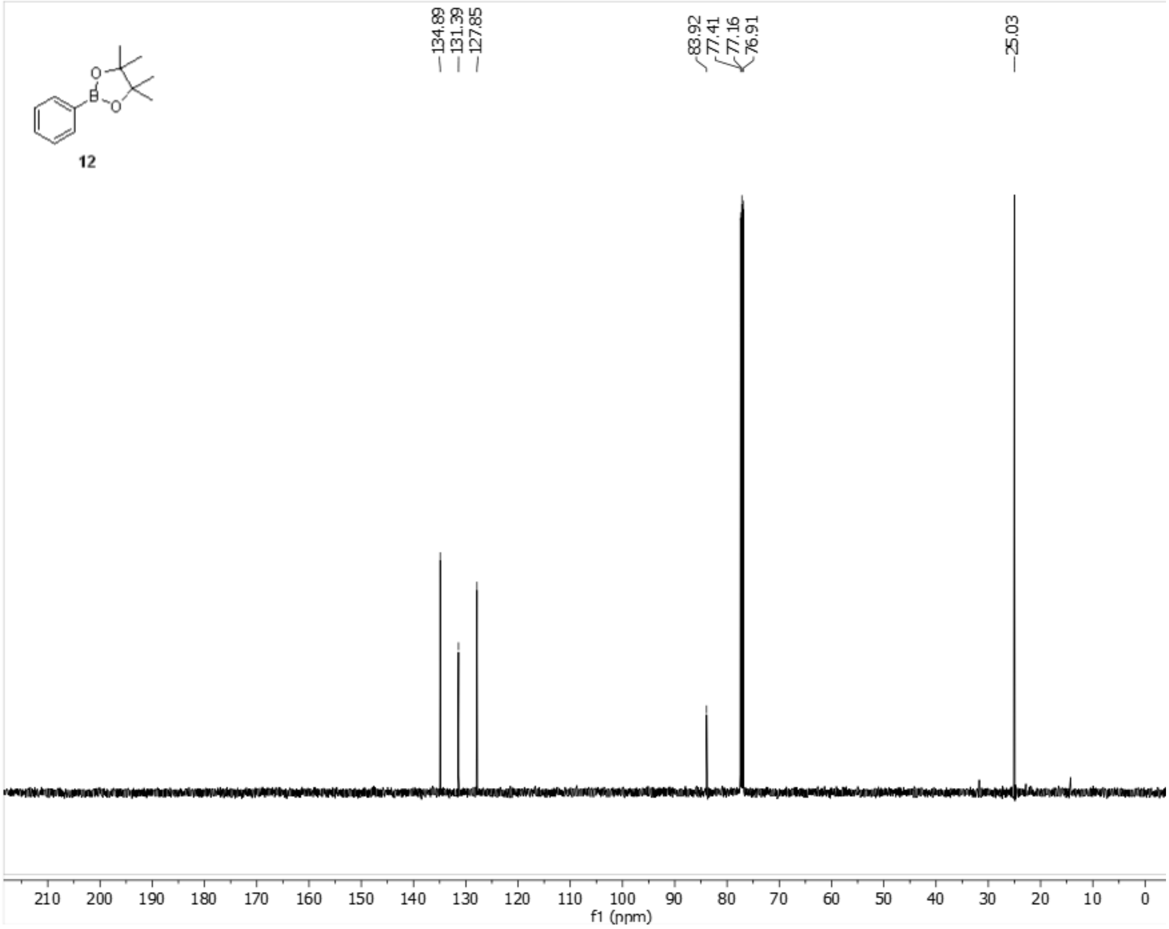

^{11}B NMR (160 MHz, $CDCl_3$)

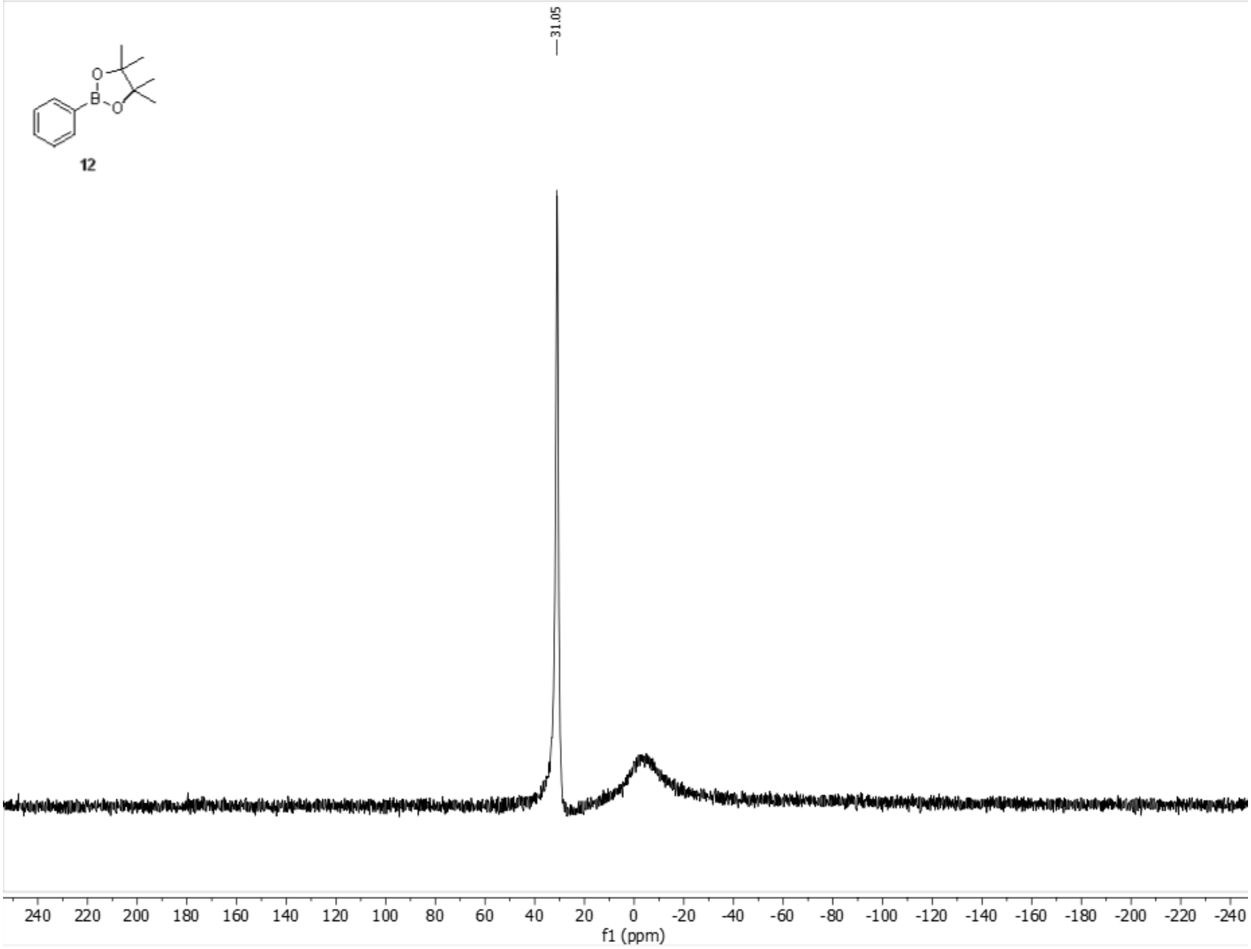

4-(4,4,5,5-Tetramethyl-1,3,2-dioxaborolan-2-yl)phenol 13a

^{1}H NMR (400 MHz, $CDCl_3$)

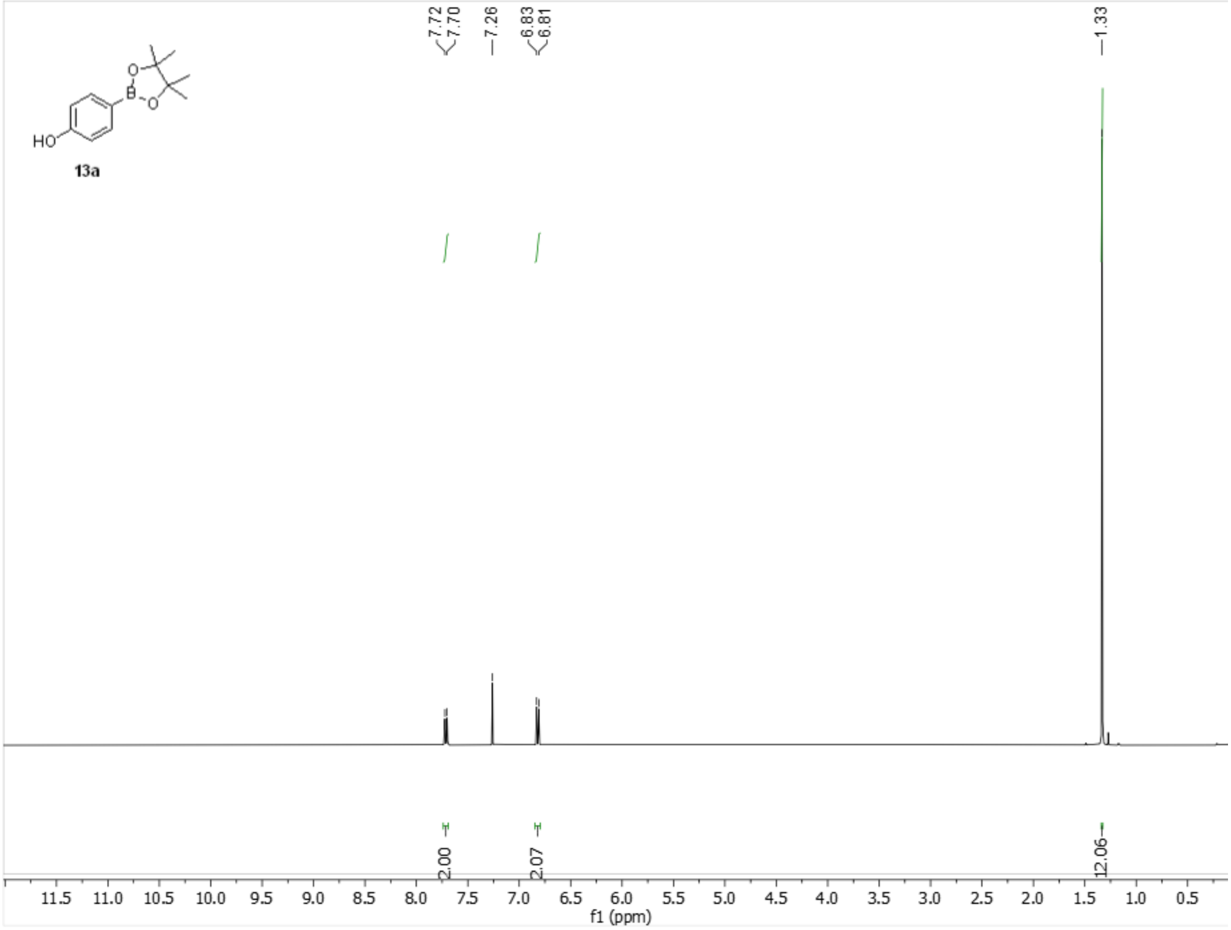

^{13}C NMR (101 MHz, $CDCl_3$)

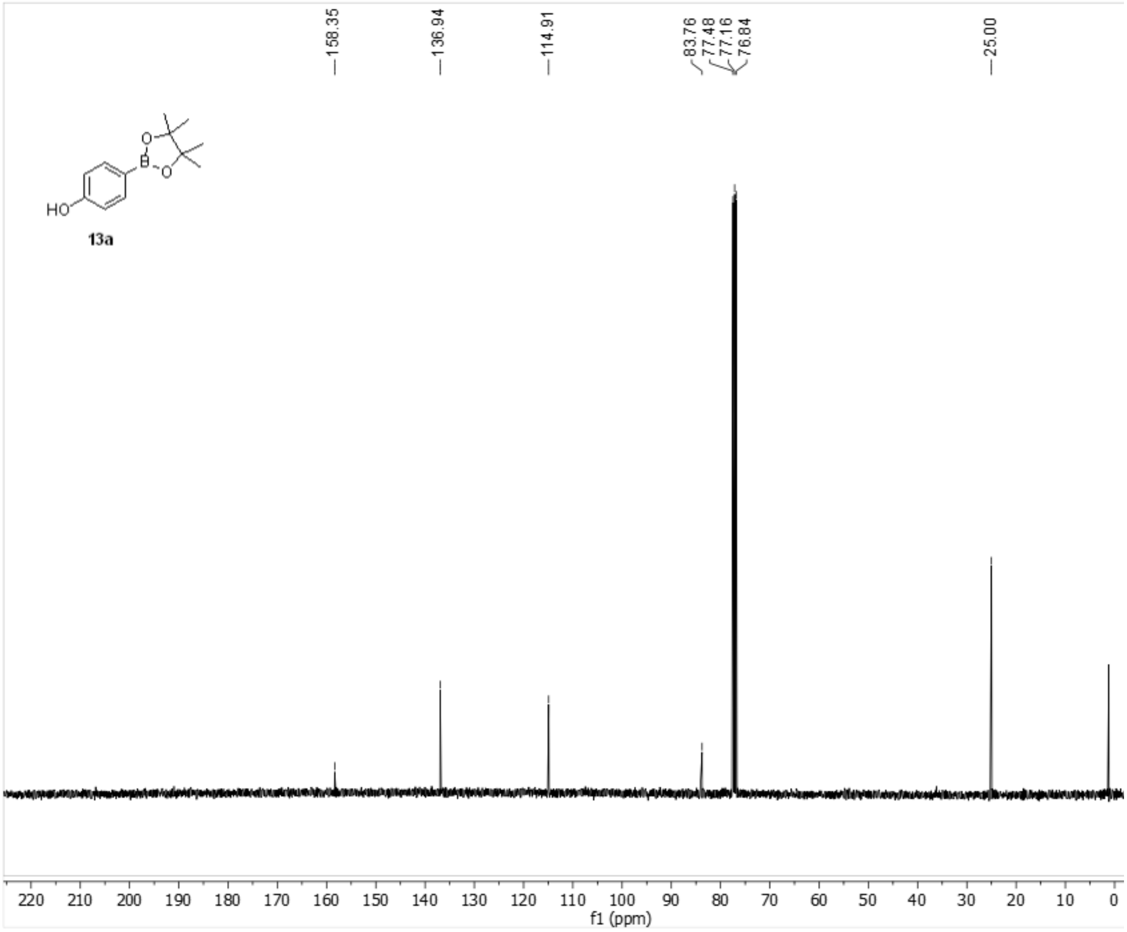

^{11}B NMR (128 MHz, $CDCl_3$)

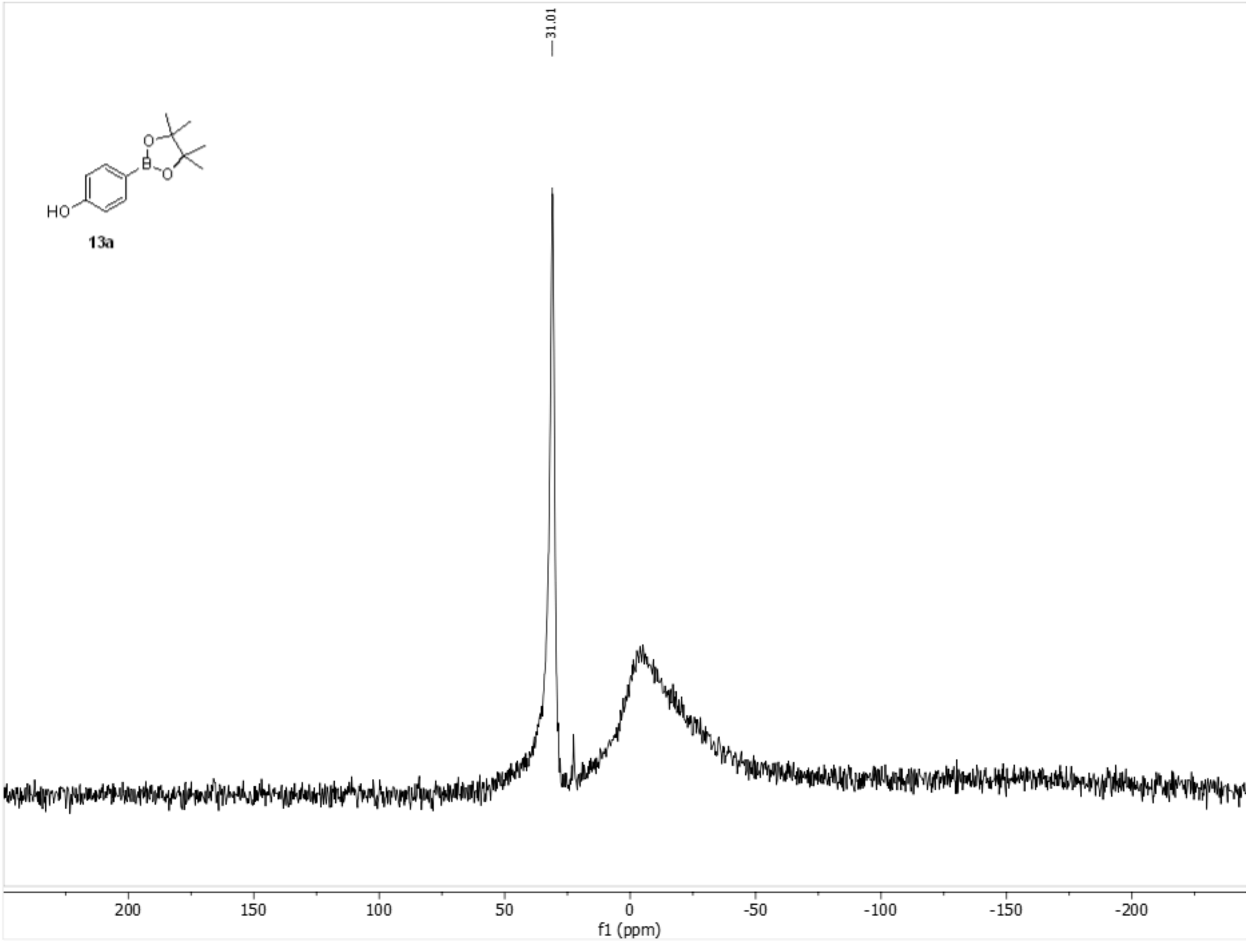

2-(4-Methoxyphenyl)-4,4,5,5-tetramethyl-1,3,2-dioxaborolane 13b

^{1}H NMR (400 MHz, $CDCl_3$)

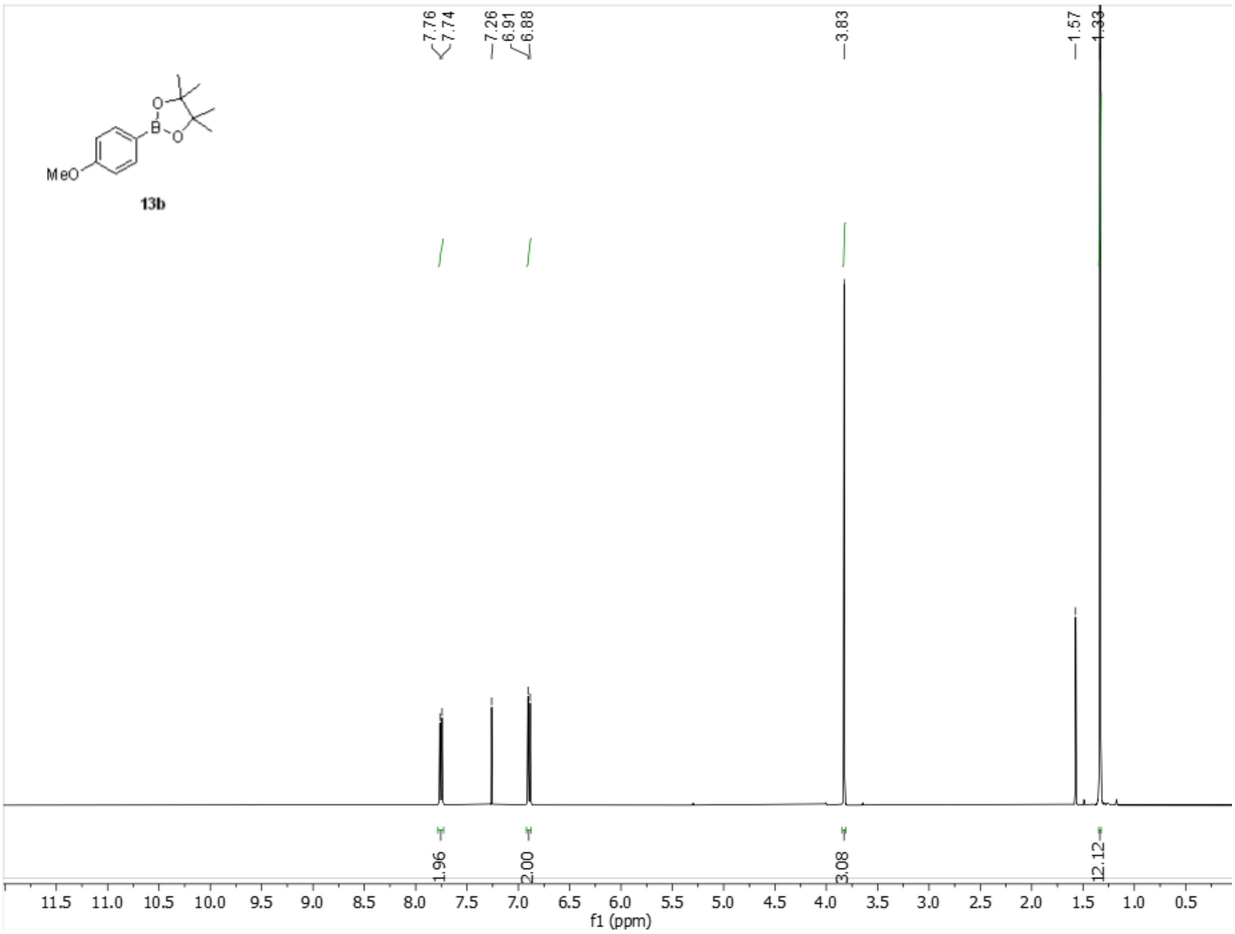

^{13}C NMR (101 MHz, $CDCl_3$)

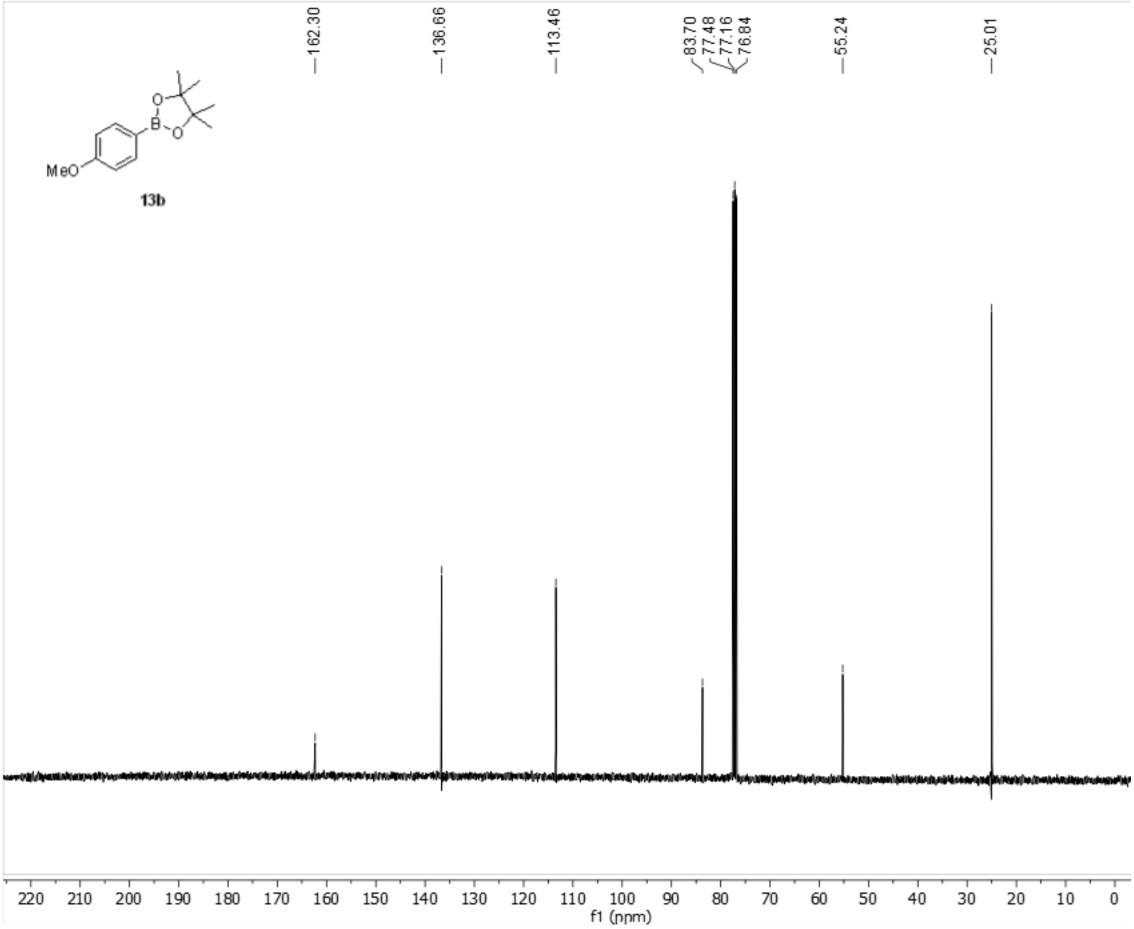

^{11}B NMR (128 MHz, $CDCl_3$)

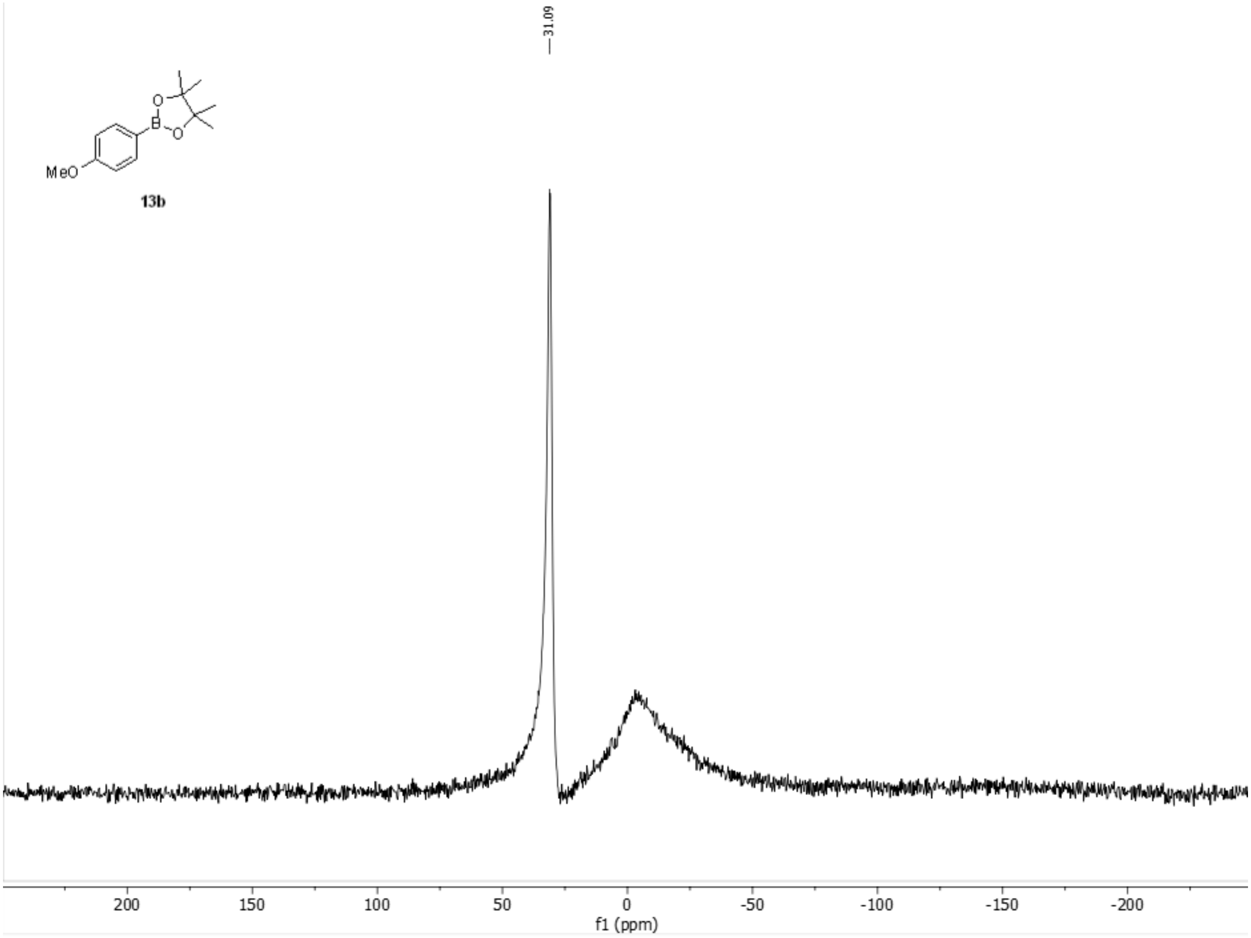

4,4,5,5-Tetramethyl-2-(*p*-tolyl)-1,3,2-dioxaborolane 13c

^{1}H NMR (400 MHz, $CDCl_3$)

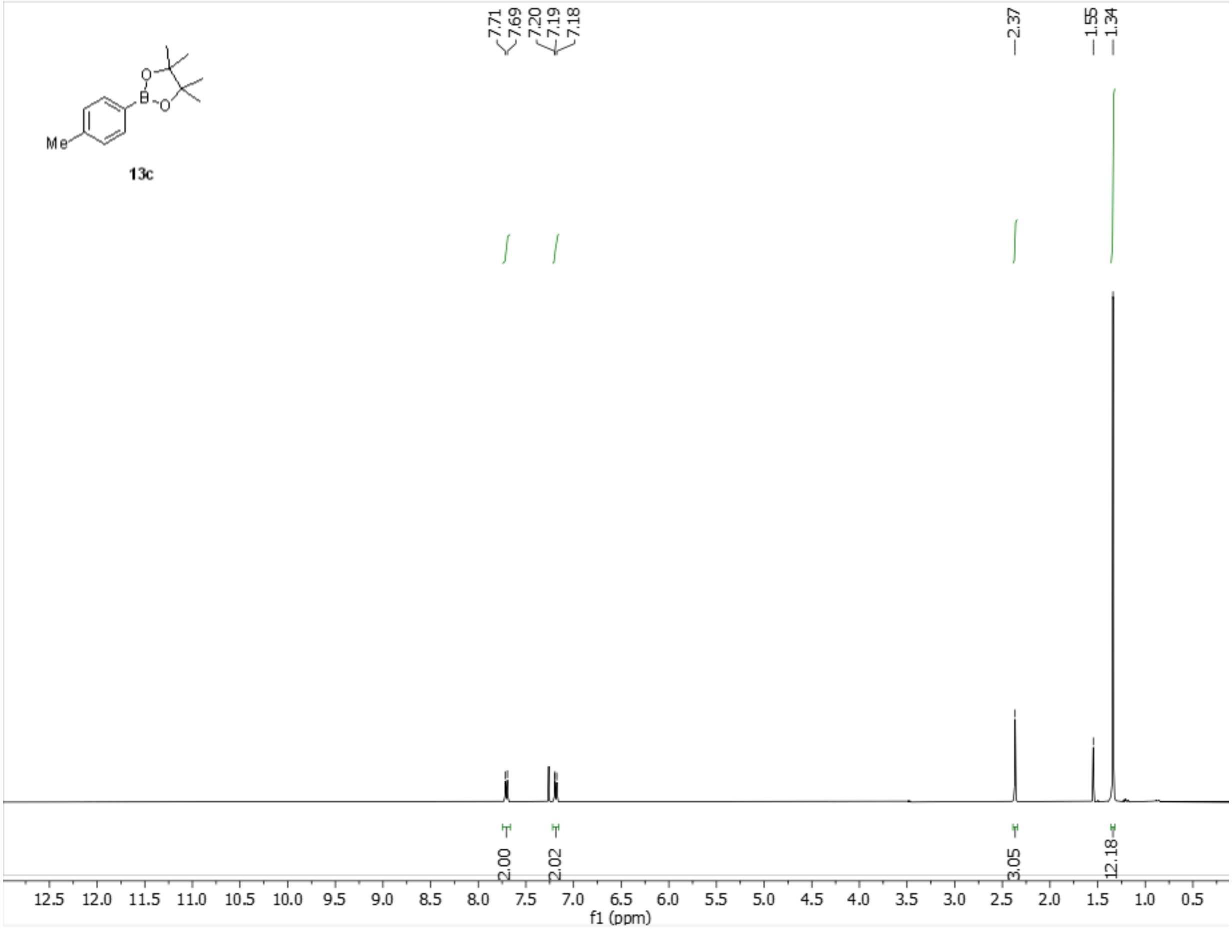

^{13}C NMR (101 MHz, $CDCl_3$)

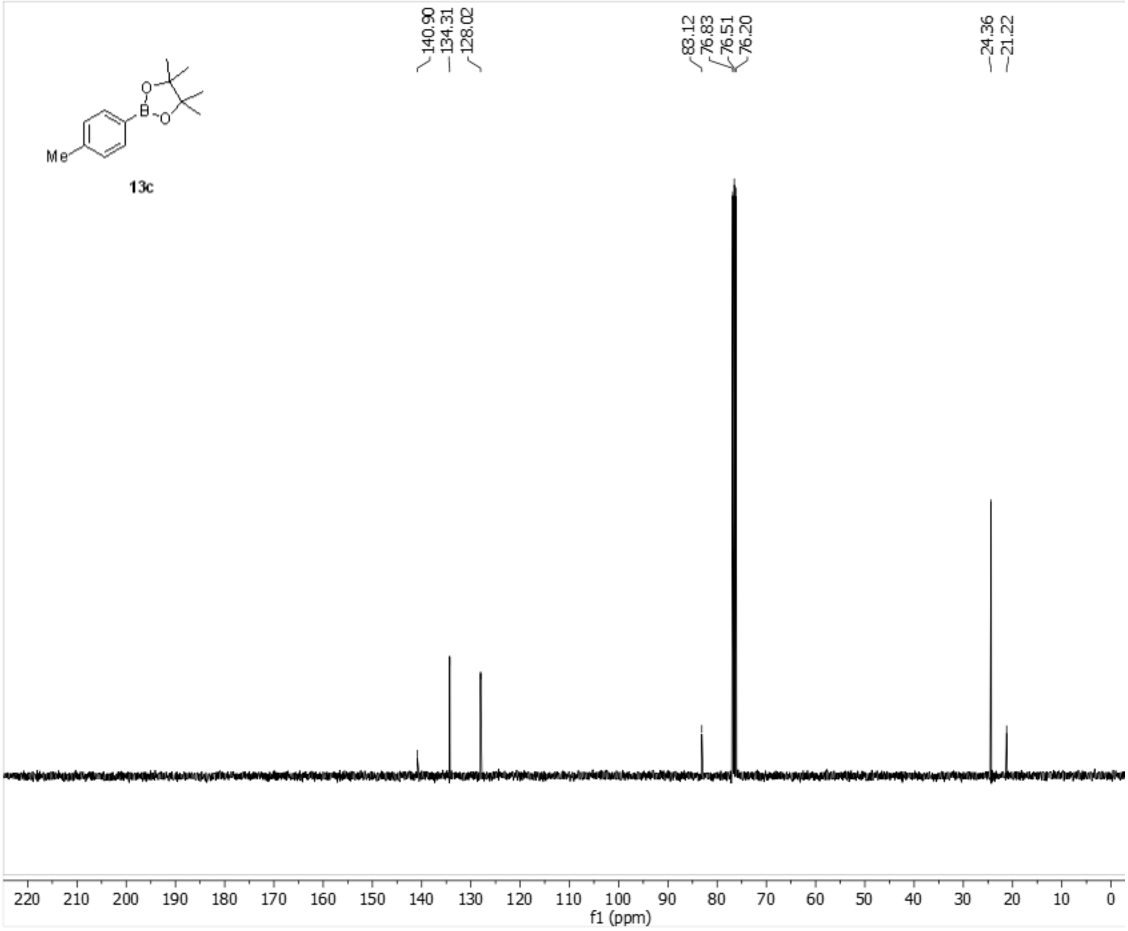

^{11}B NMR (128 MHz, $CDCl_3$)

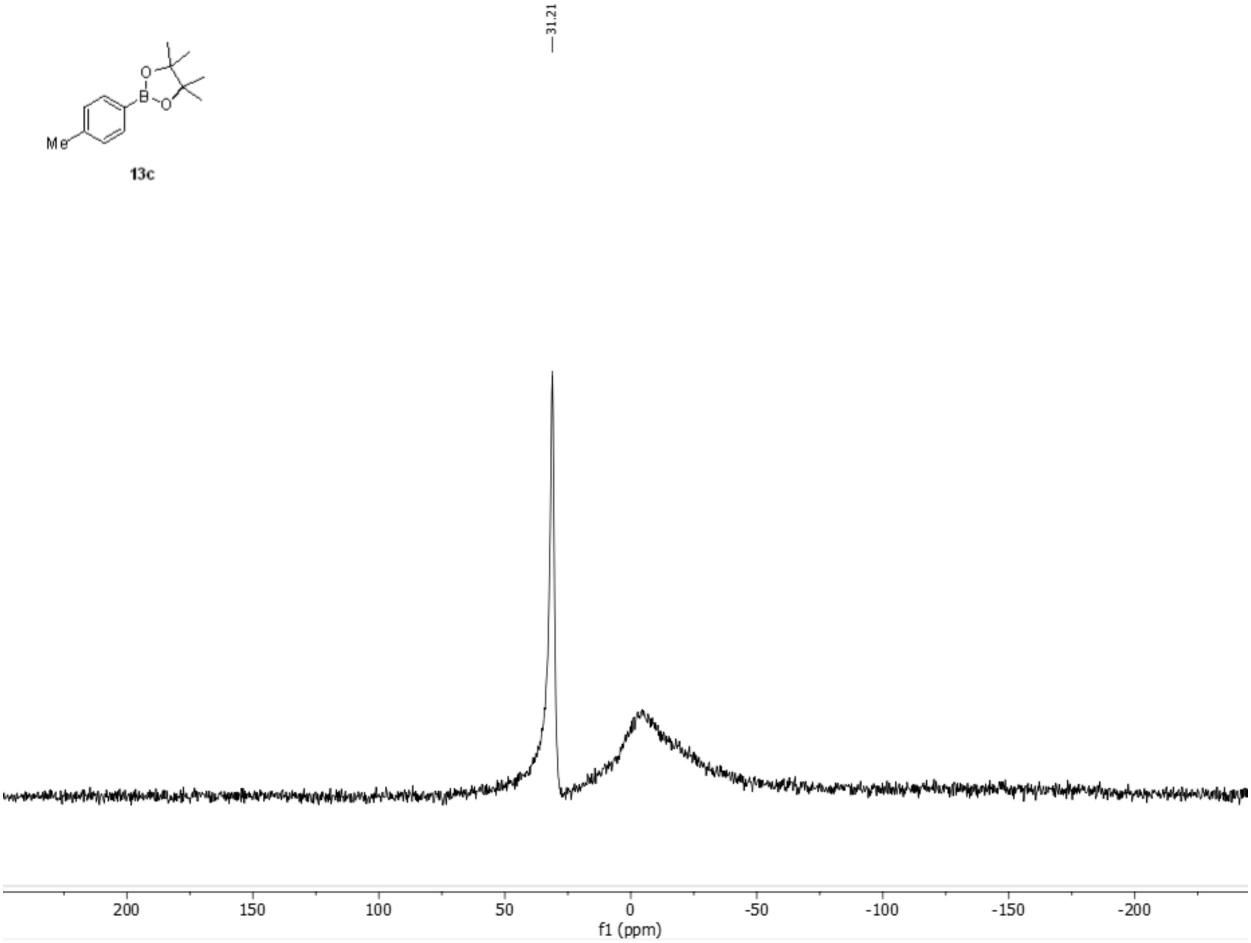

2-(4-(*Tert*-butyl)phenyl)-4,4,5,5-tetramethyl-1,3,2-dioxaborolane 13d

^{1}H NMR (400 MHz, $CDCl_3$)

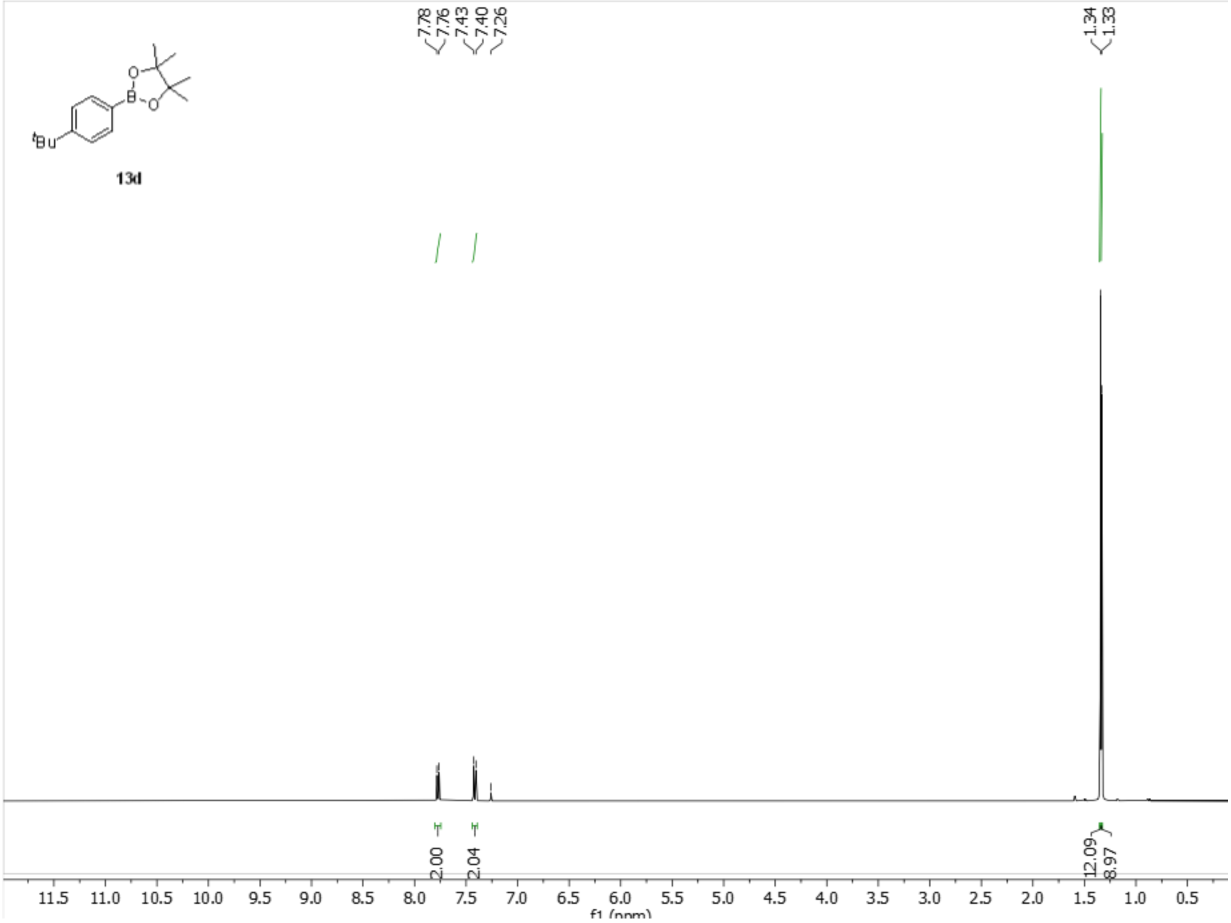

^{13}C NMR (101 MHz, $CDCl_3$)

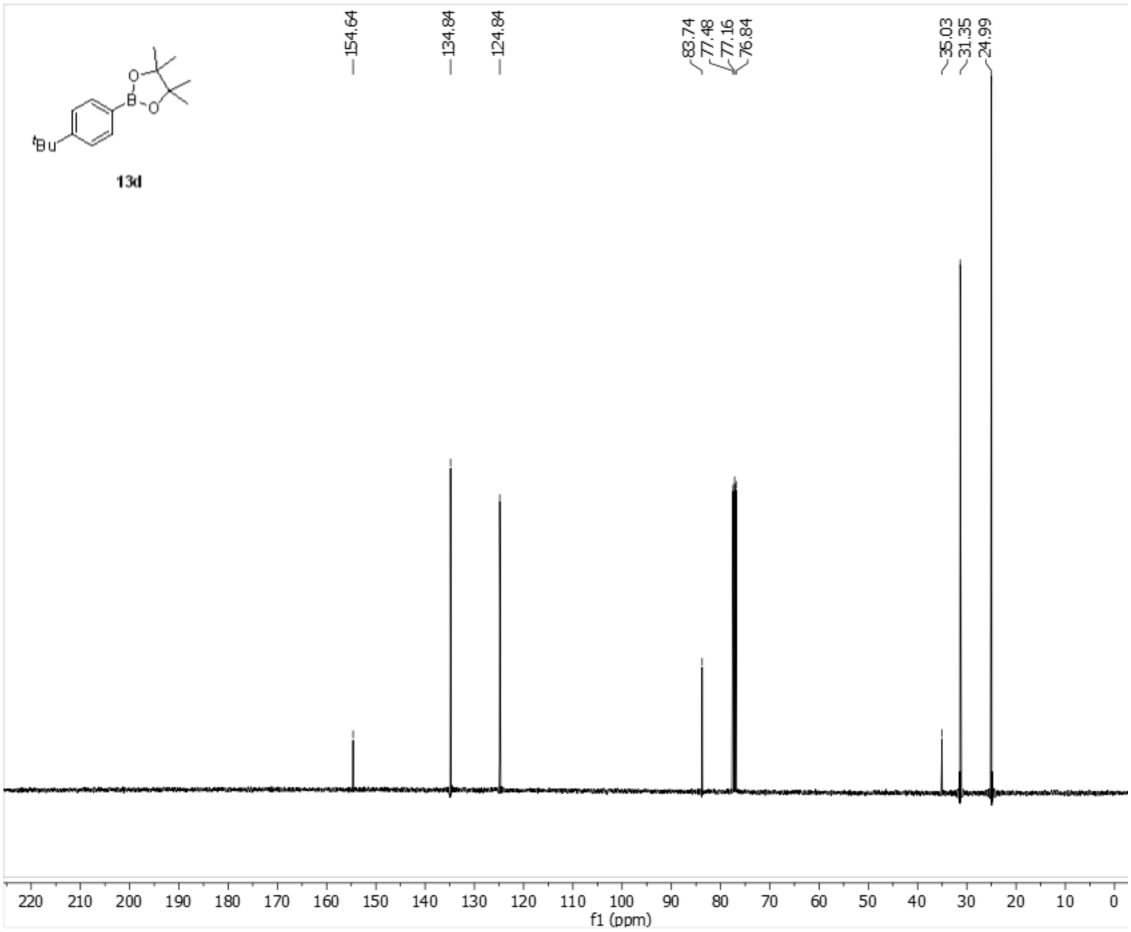

^{11}B NMR (128 MHz, $CDCl_3$)

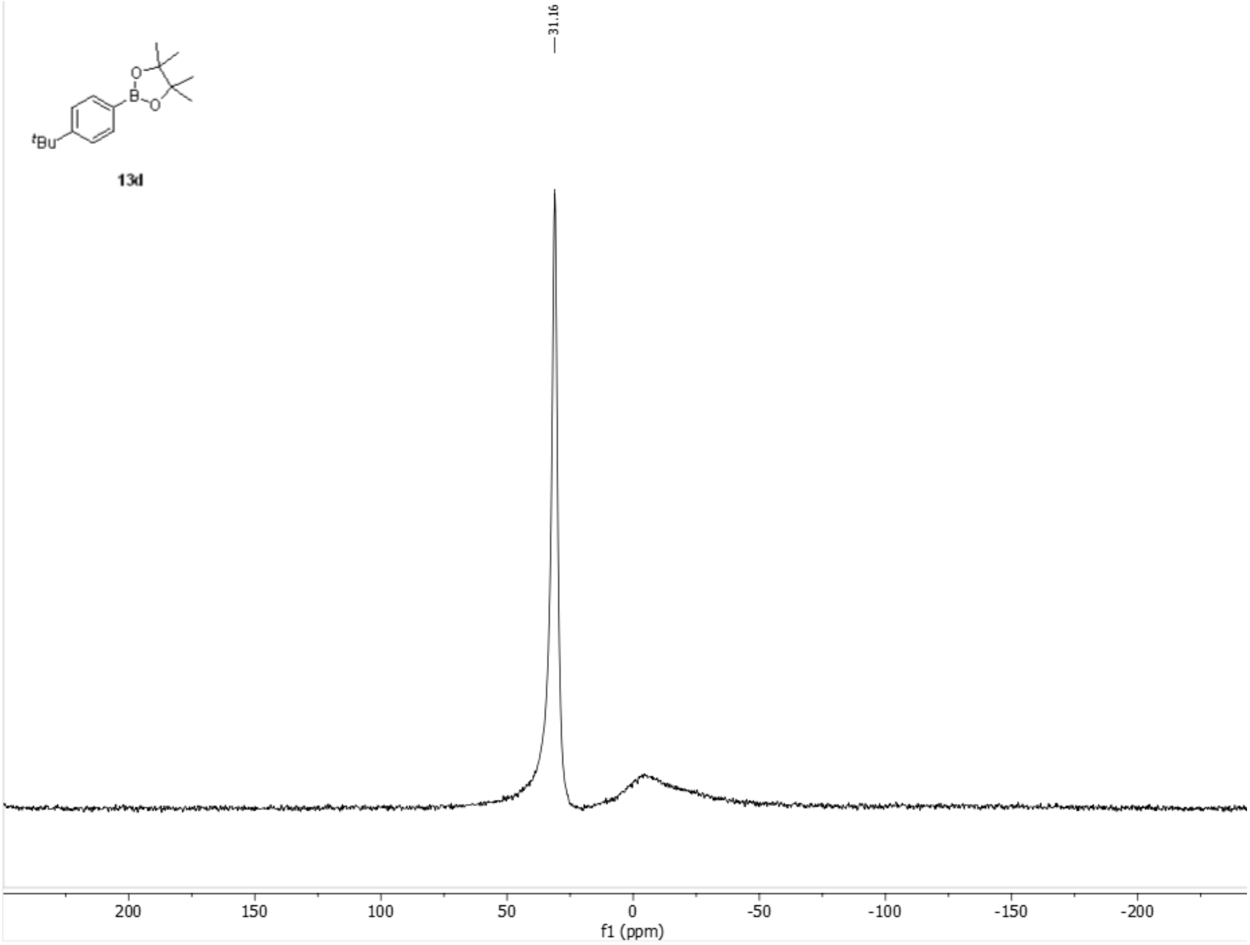

2-([1,1'-Biphenyl]-4-yl)-4,4,5,5-tetramethyl-1,3,2-dioxaborolane 13e

^{1}H NMR (400 MHz, $CDCl_3$)

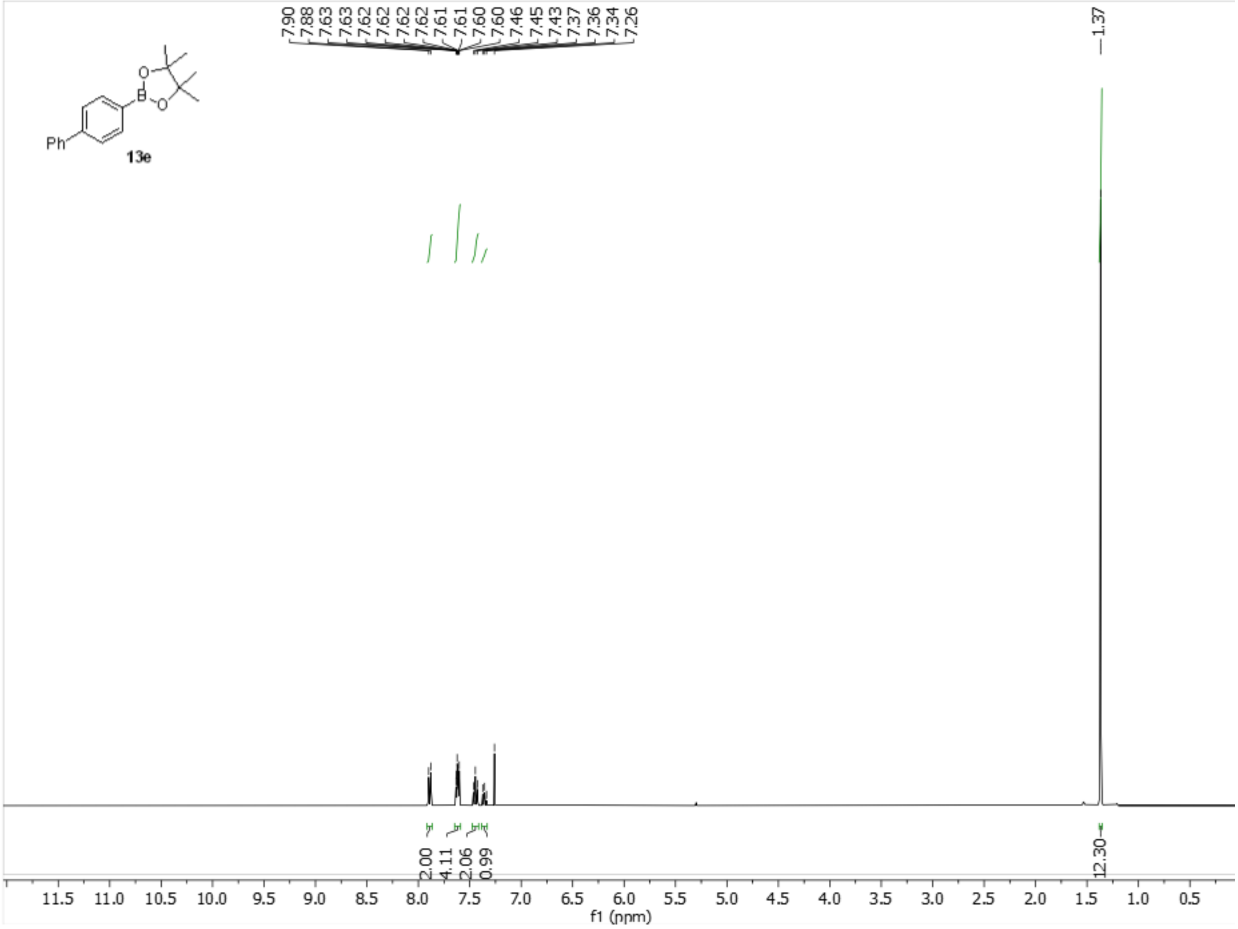

^{13}C NMR (101 MHz, $CDCl_3$)

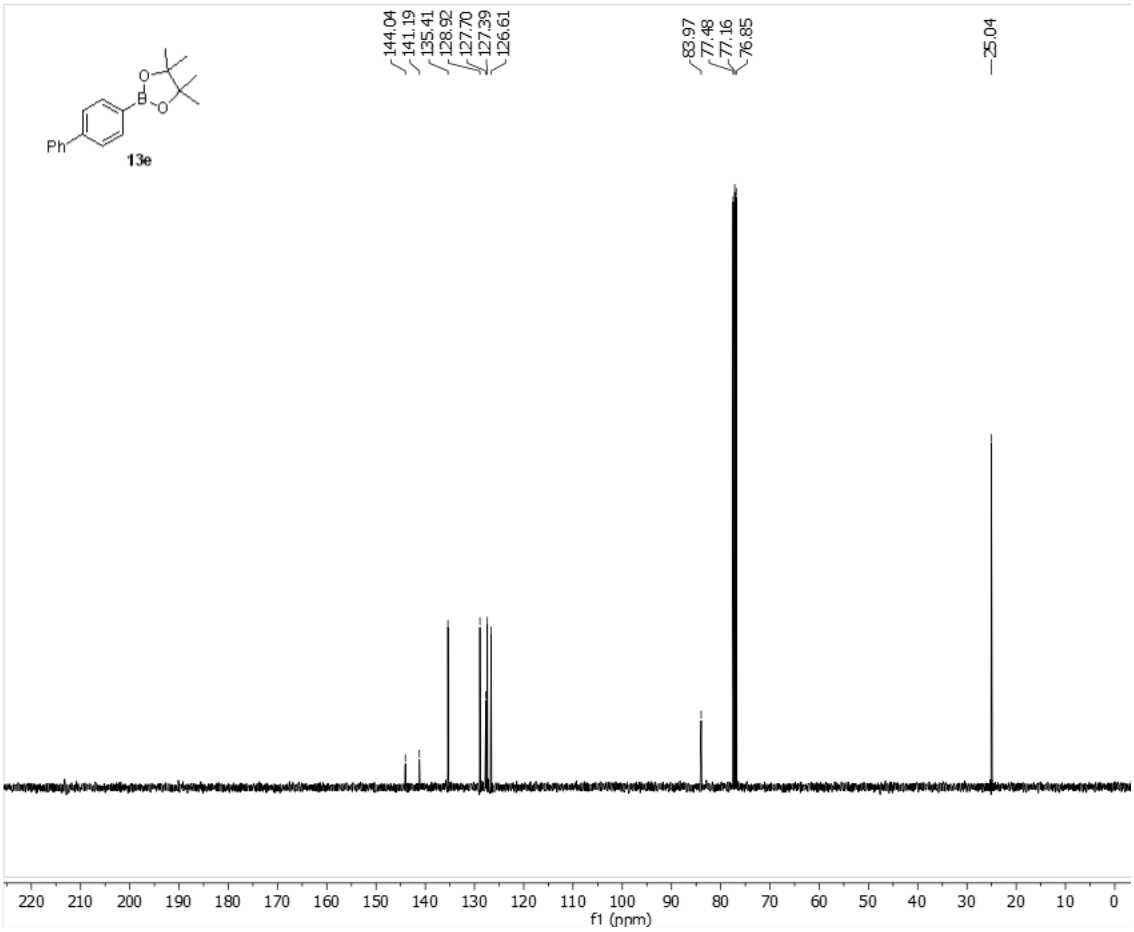

^{11}B NMR (128 MHz, $CDCl_3$)

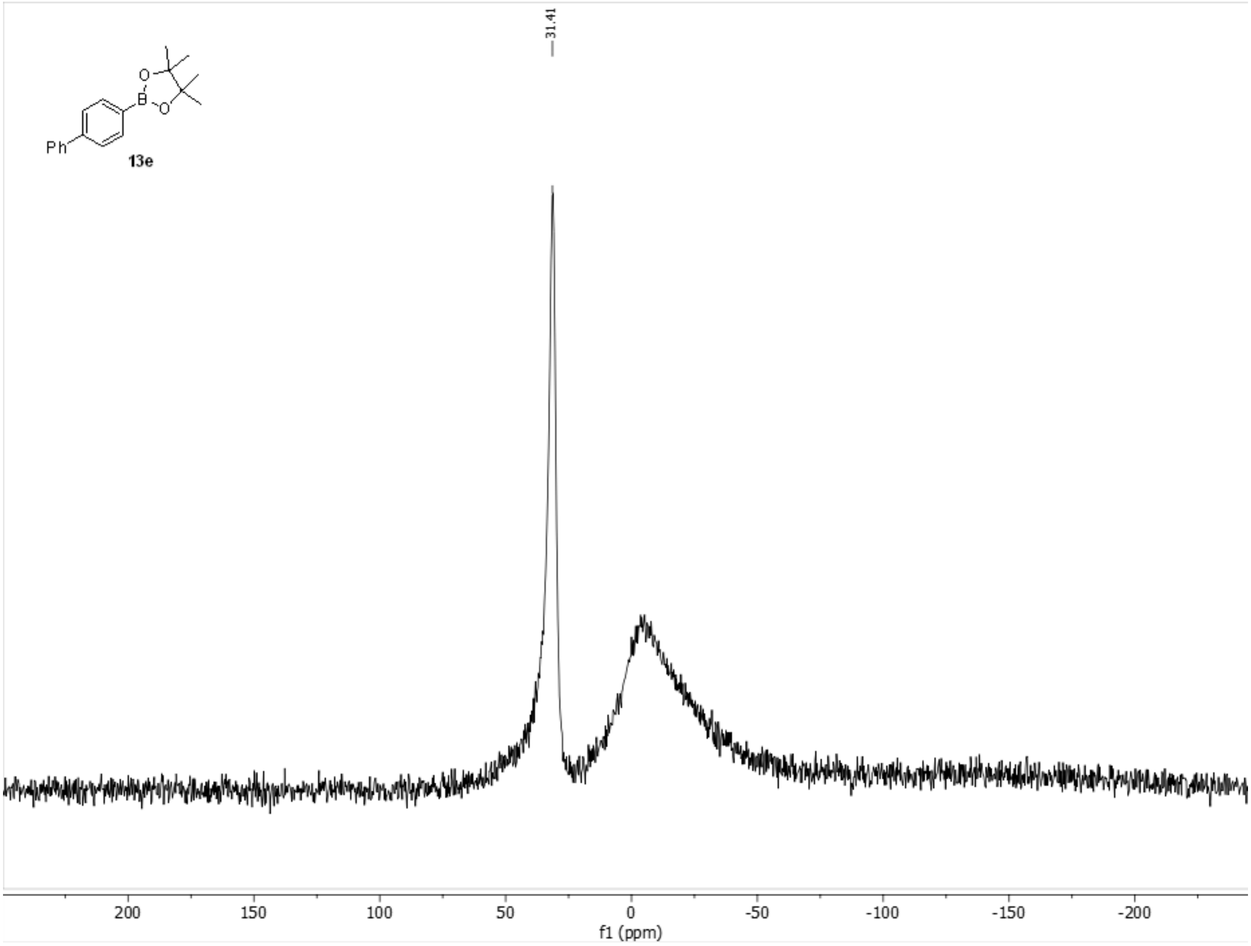

2-(4-Chlorophenyl)-4,4,5,5-tetramethyl-1,3,2-dioxaborolane 13f

^{1}H NMR (400 MHz, $CDCl_3$)

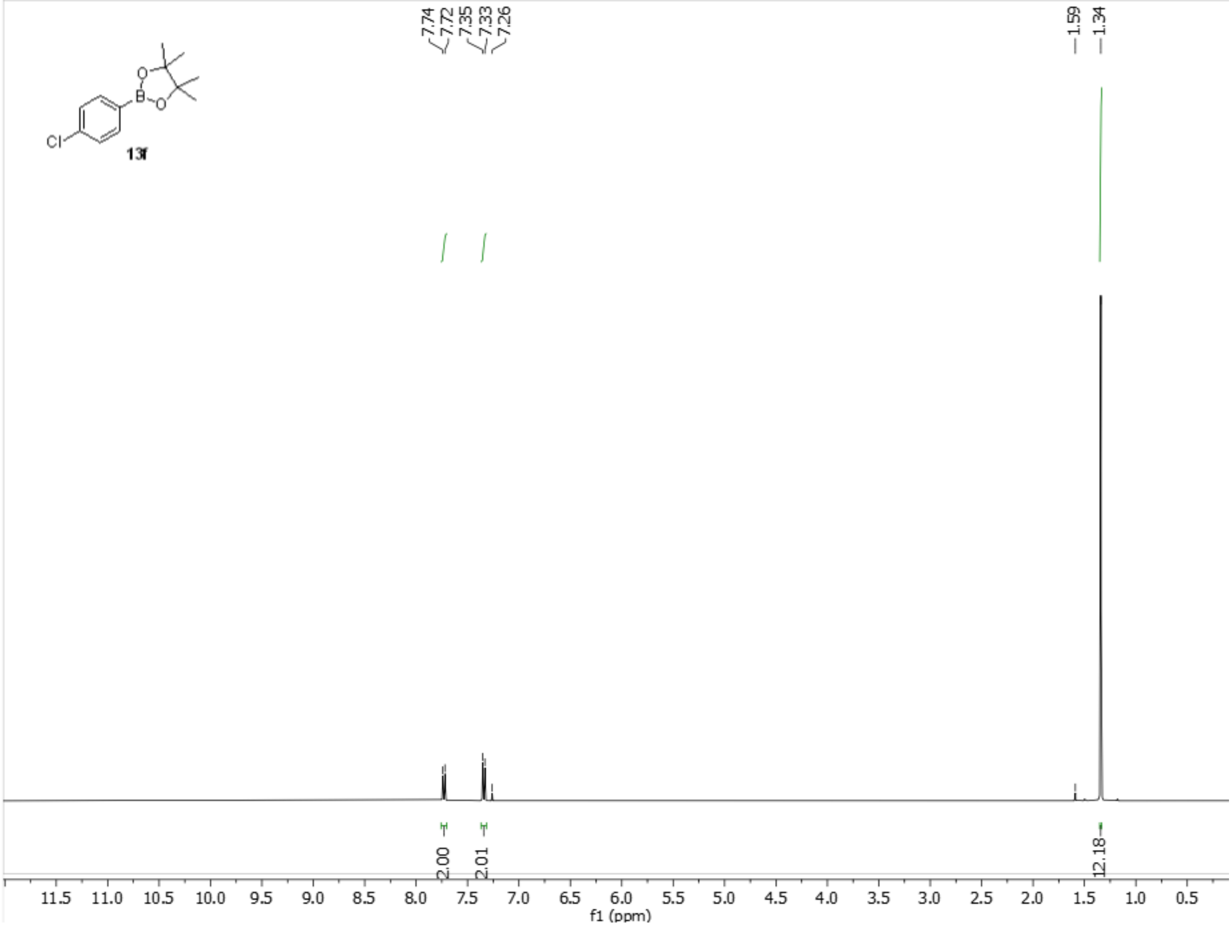

^{13}C NMR (101 MHz, $CDCl_3$)

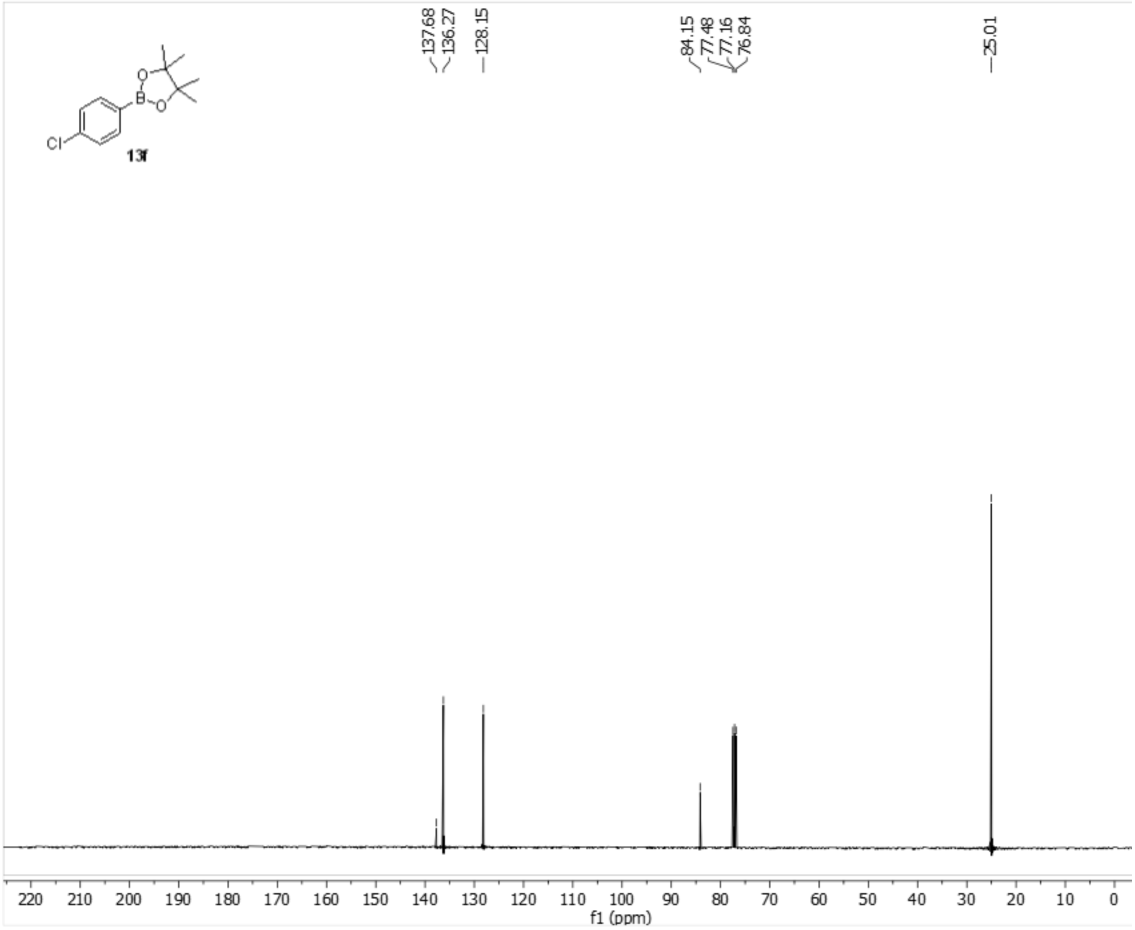

^{11}B NMR (128 MHz, $CDCl_3$)

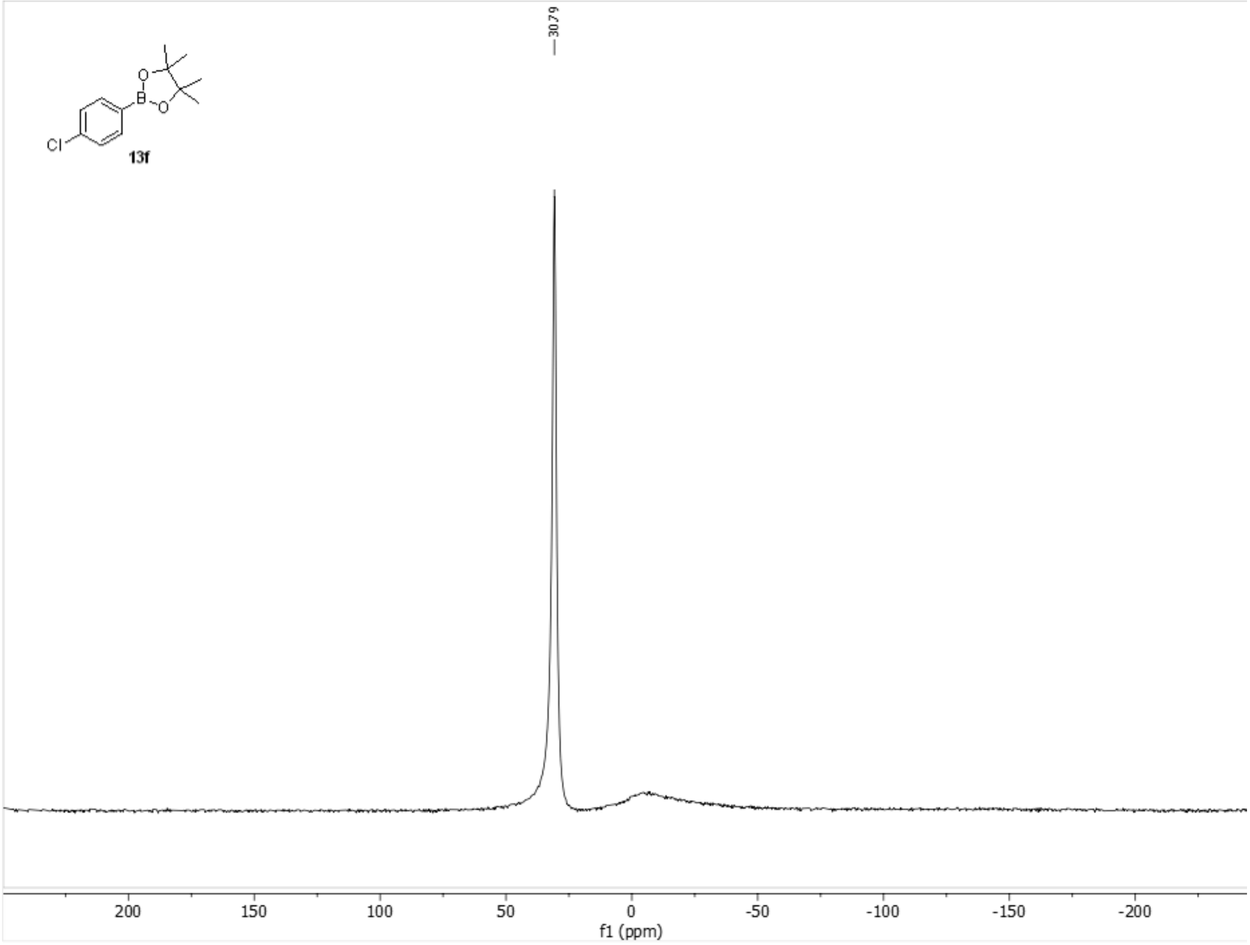

2-(4-Fluorophenyl)-4,4,5,5-tetramethyl-1,3,2-dioxaborolane 13g

^1H NMR (400 MHz, $CDCl_3$)

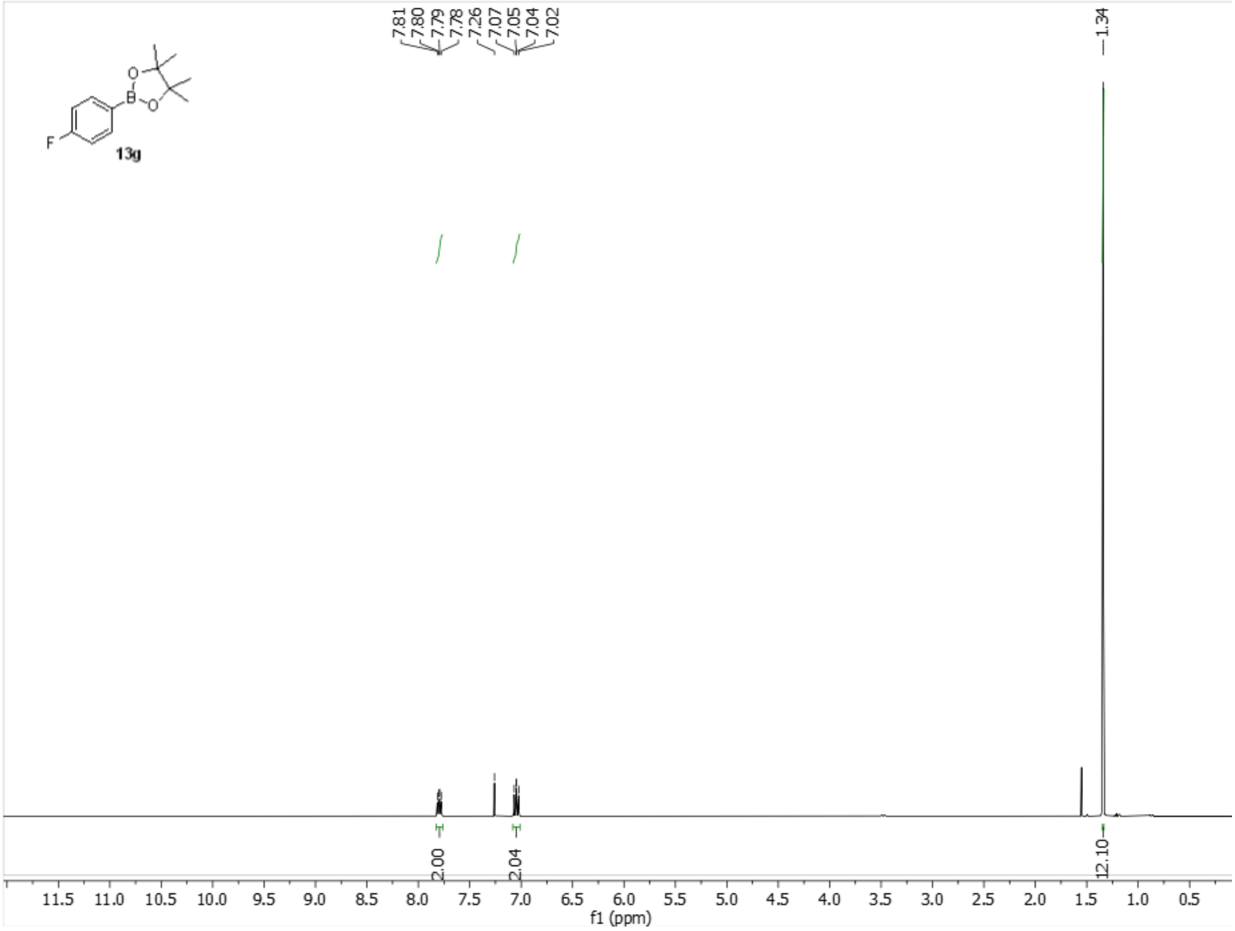

^{13}C NMR (101 MHz, $CDCl_3$)

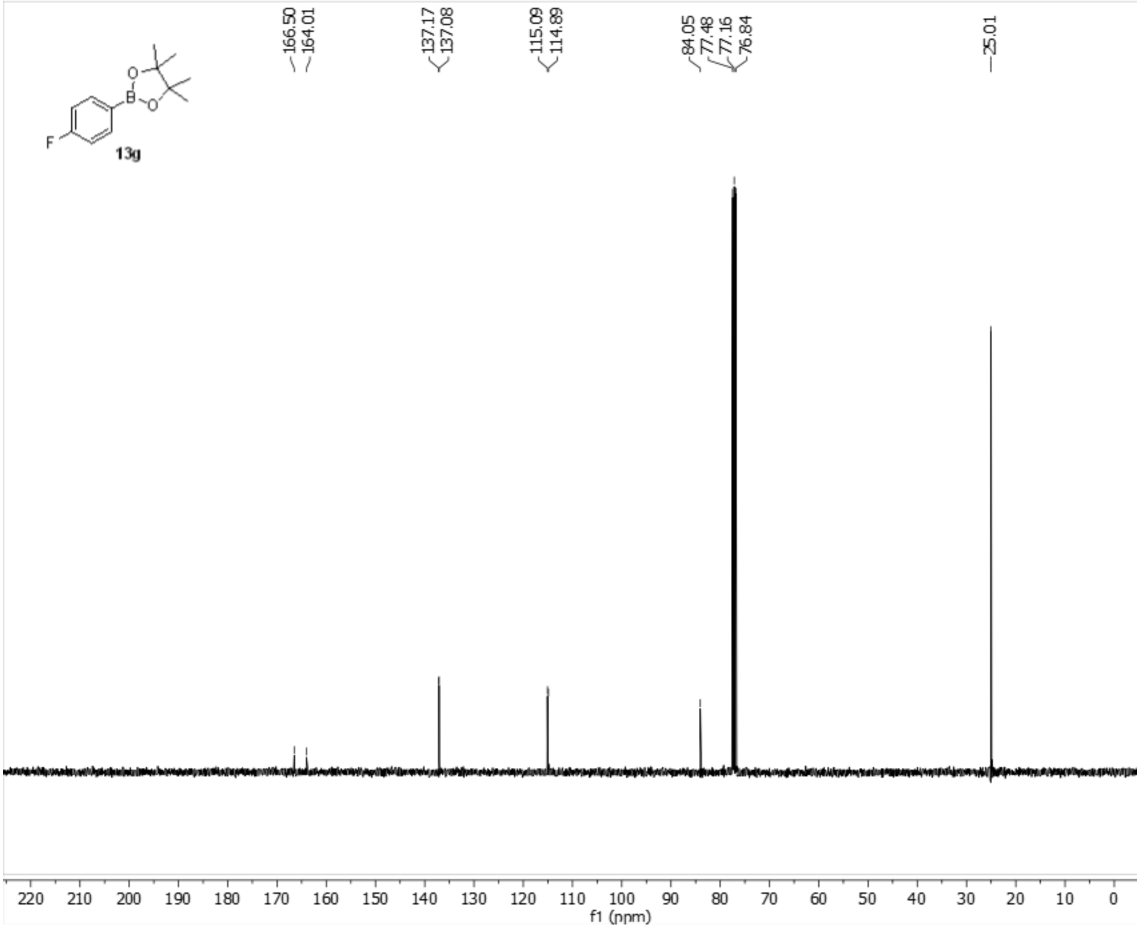

^{11}B NMR (128 MHz, $CDCl_3$)

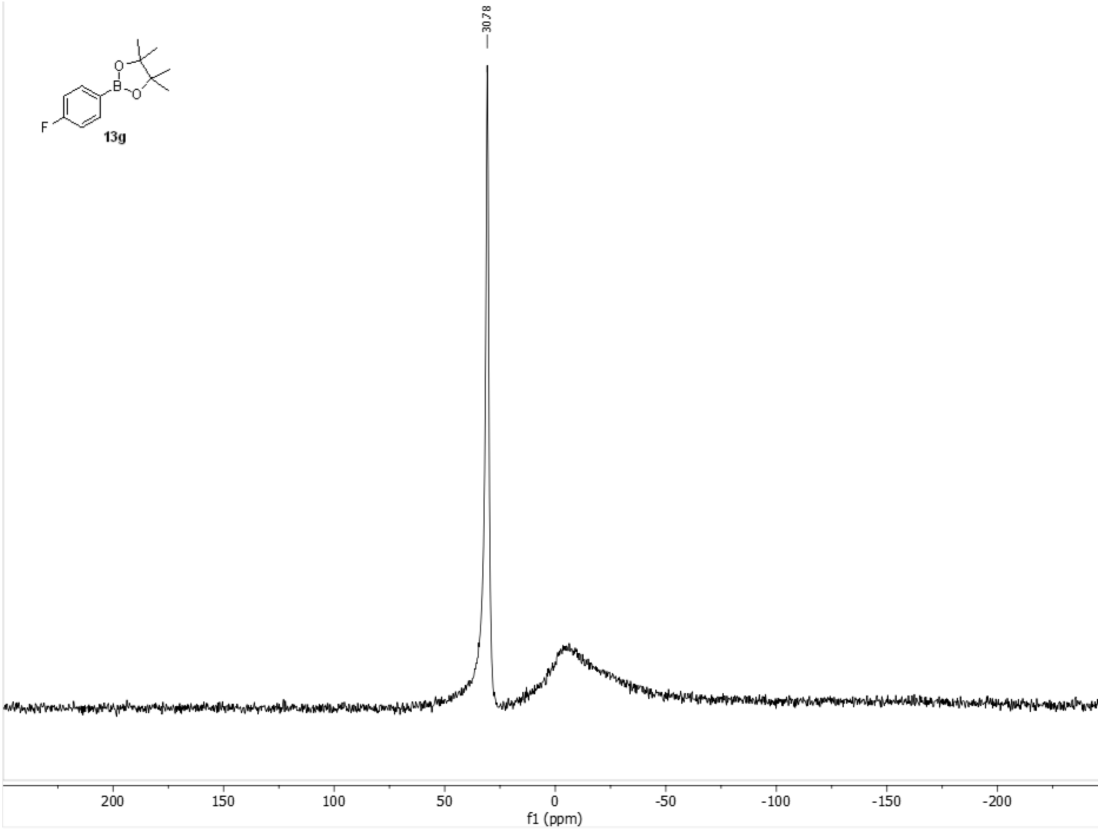

^{19}F NMR (376 MHz, $CDCl_3$)

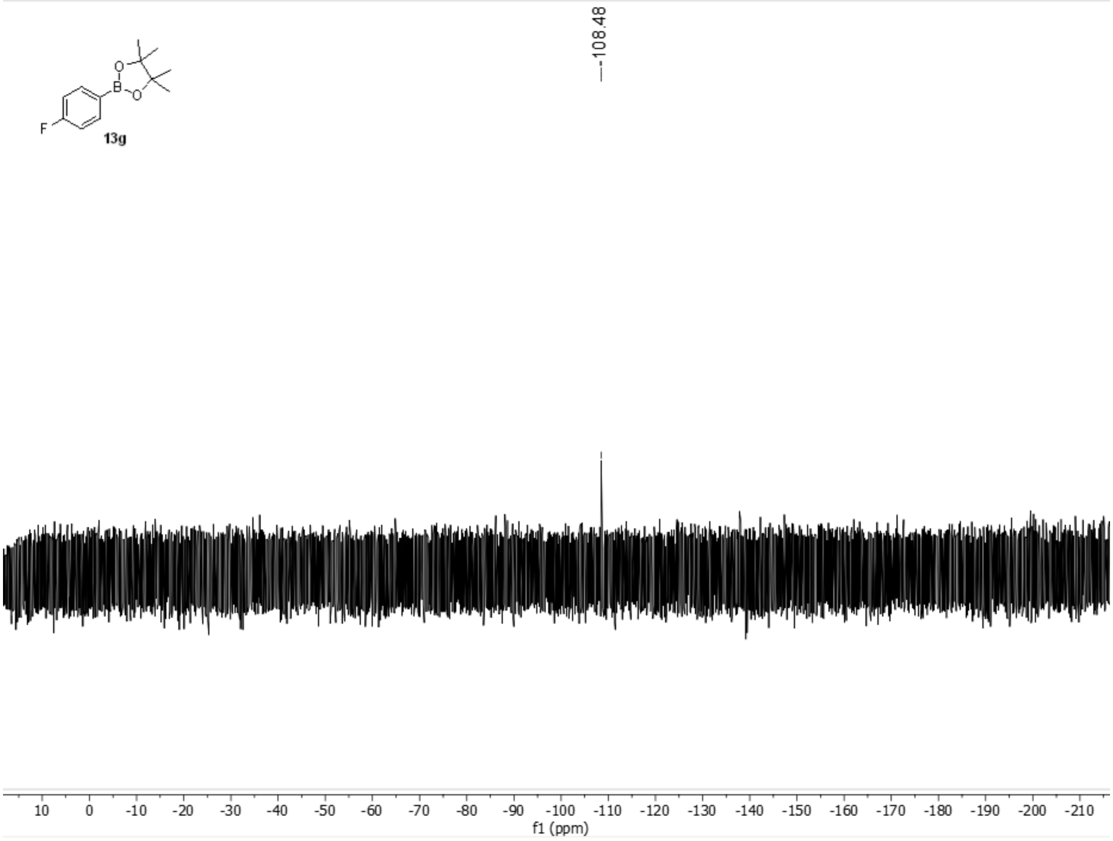

2-(4,4,5,5-Tetramethyl-1,3,2-dioxaborolan-2-yl)phenol 13j

^{1}H NMR (400 MHz, $CDCl_3$)

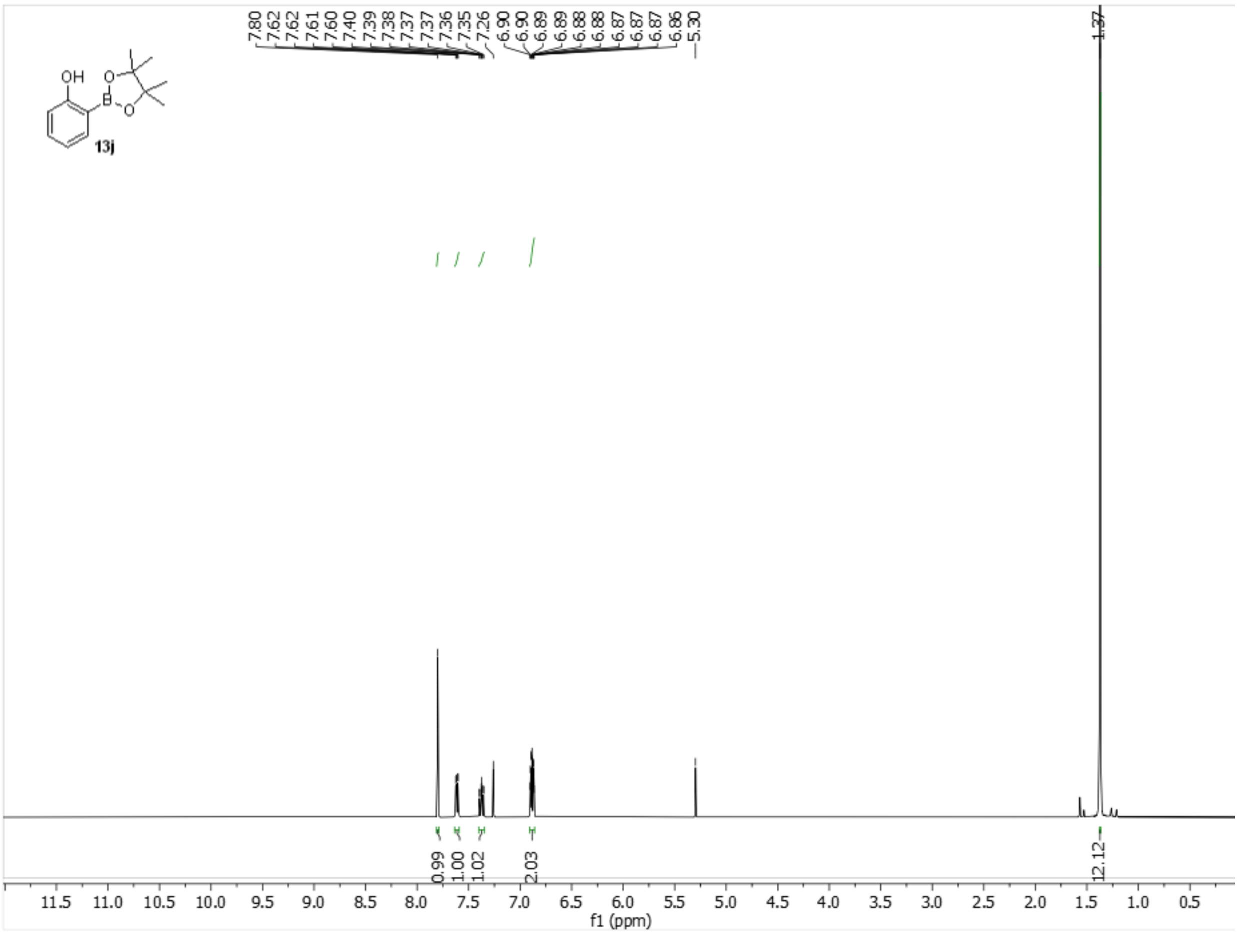

^{13}C NMR (101 MHz, $CDCl_3$)

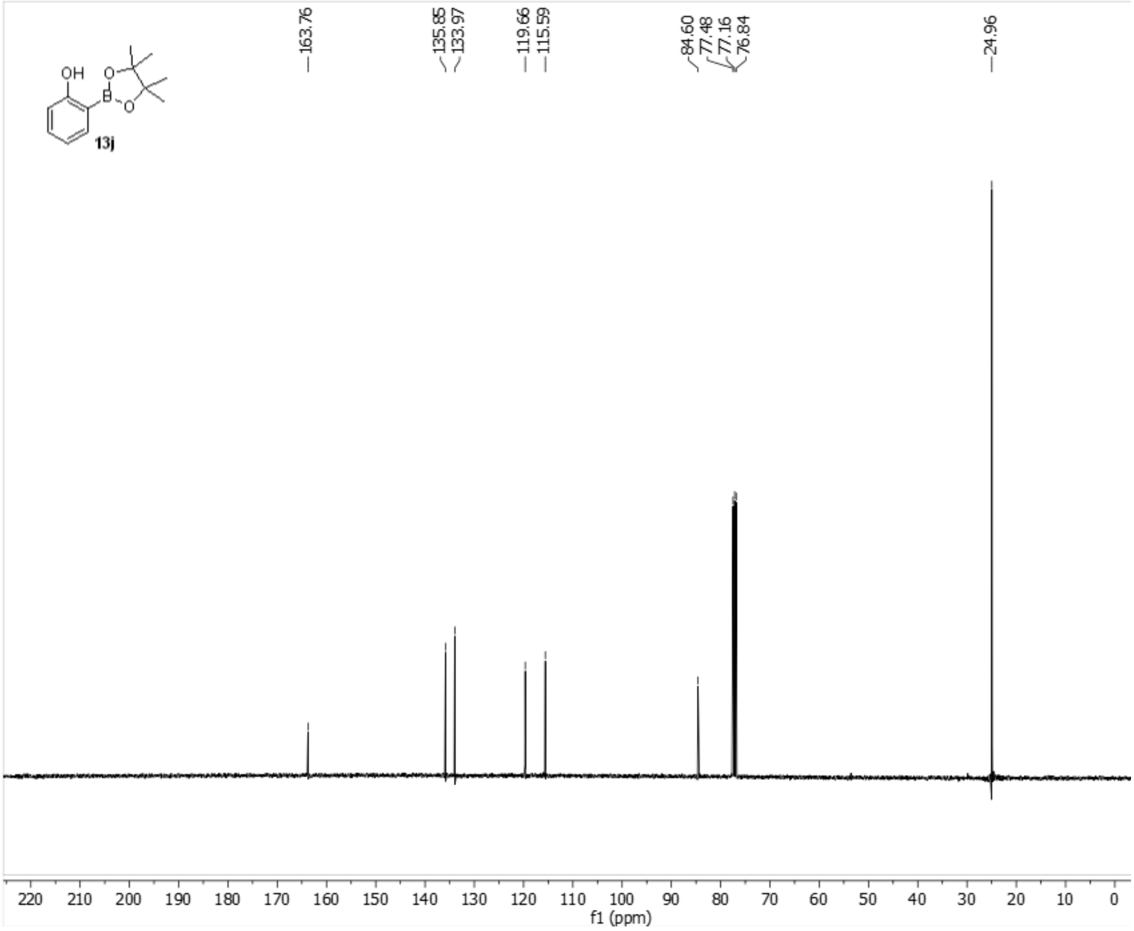

^{11}B NMR (128 MHz, $CDCl_3$)

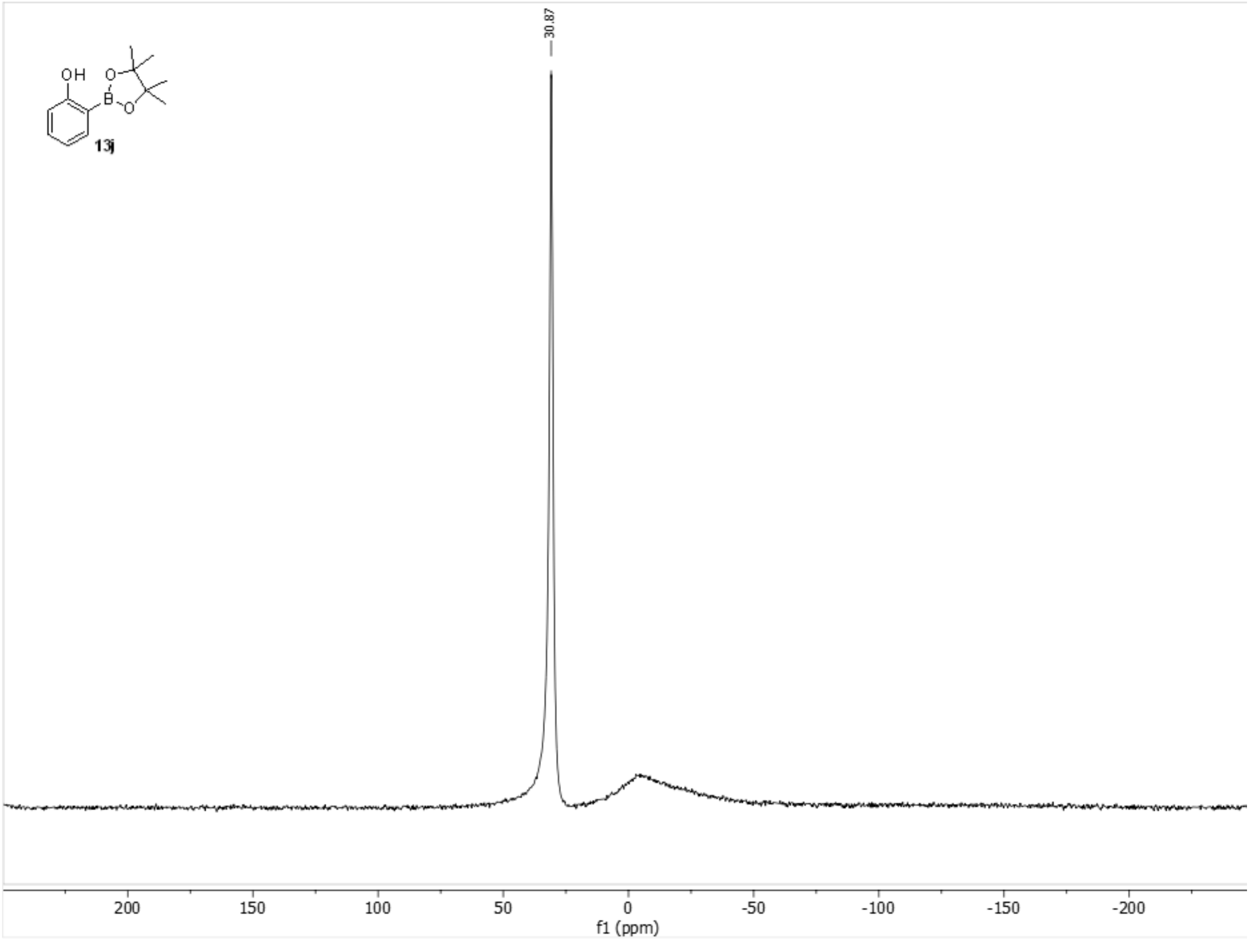

2-(2-Methoxyphenyl)-4,4,5,5-tetramethyl-1,3,2-dioxaborolane 13k

^{1}H NMR (400 MHz, $CDCl_3$)

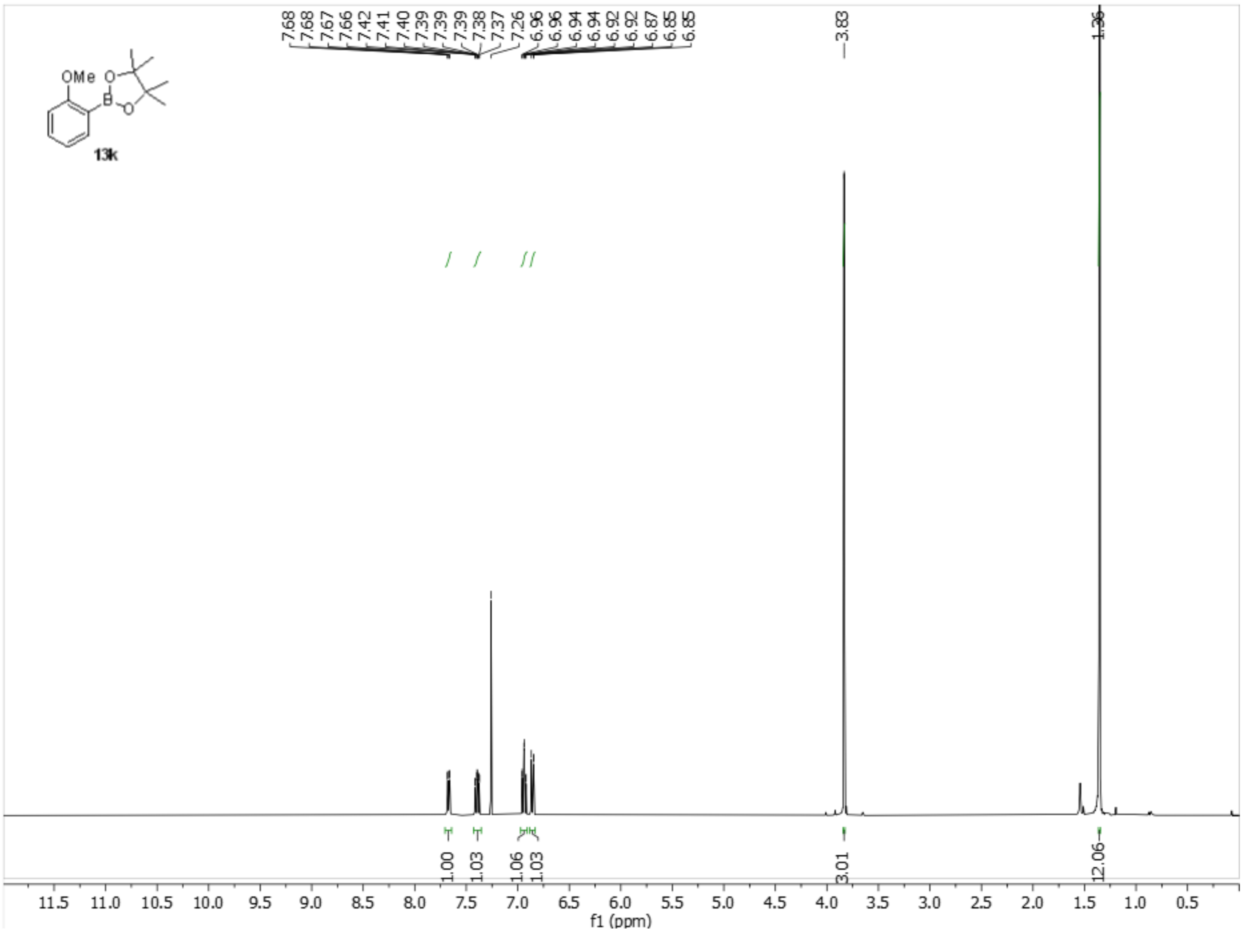

^{13}C NMR (101 MHz, $CDCl_3$)

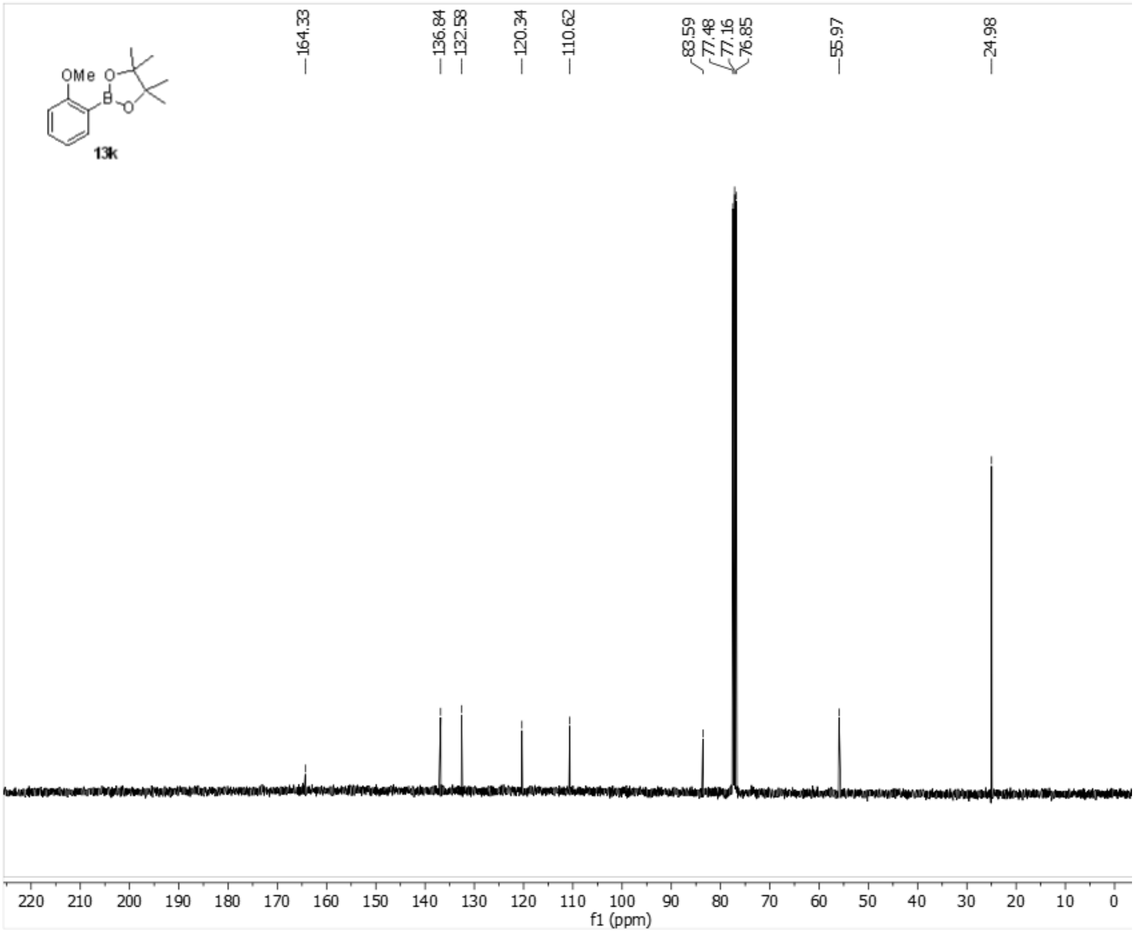

^{11}B NMR (128 MHz, $CDCl_3$)

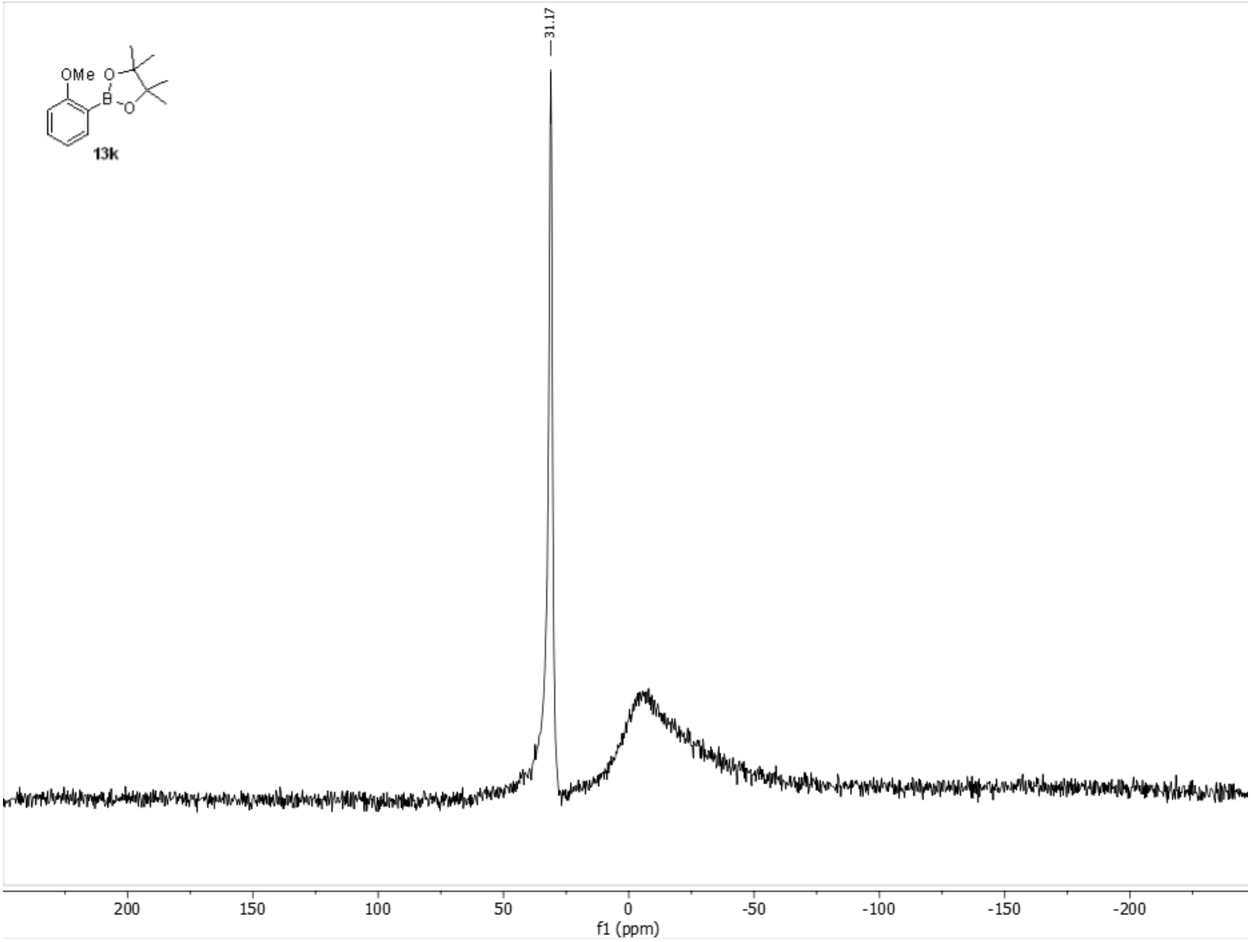

4,4,5,5-Tetramethyl-2-(*o*-tolyl)-1,3,2-dioxaborolane 13l

^{1}H NMR (400 MHz, $CDCl_3$)

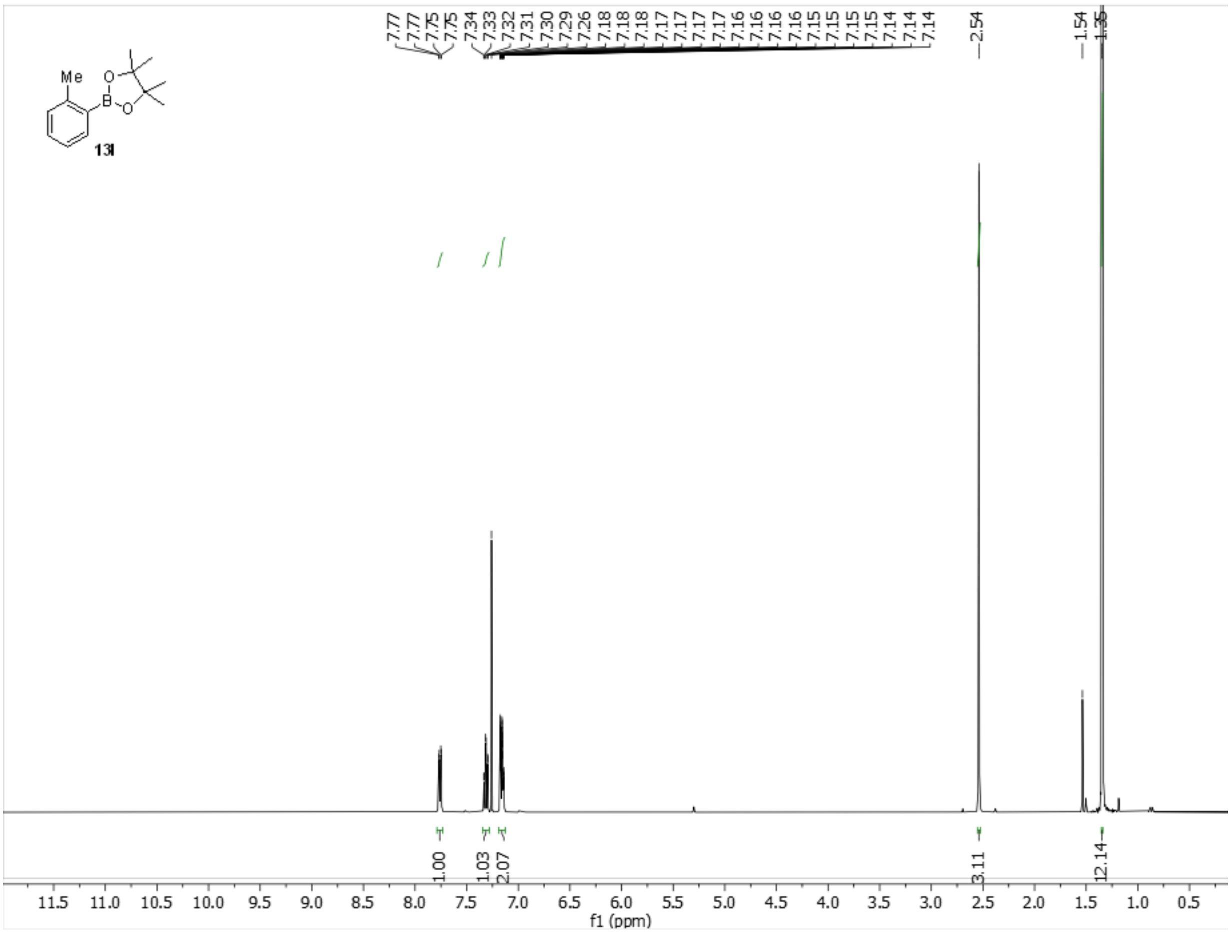

^{13}C NMR (101 MHz, $CDCl_3$)

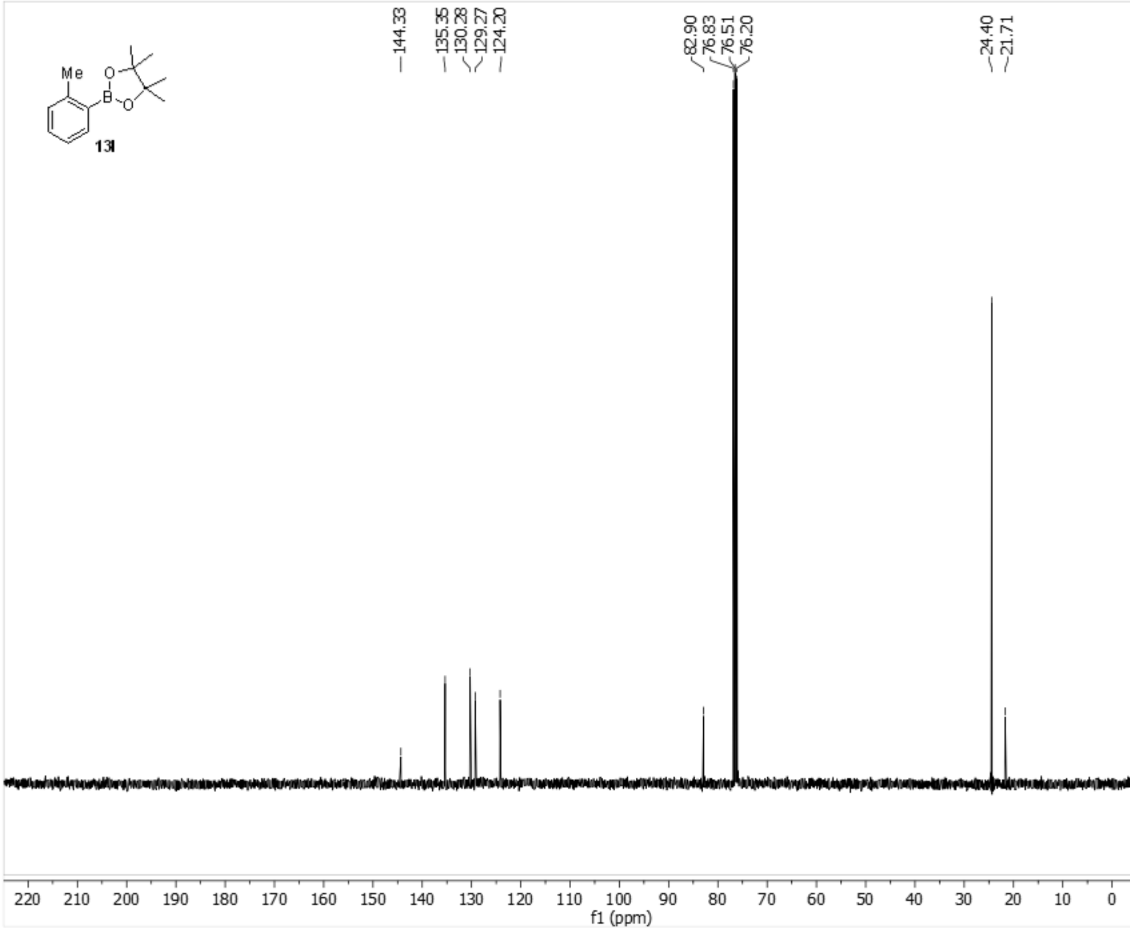

^{11}B NMR (128 MHz, $CDCl_3$)

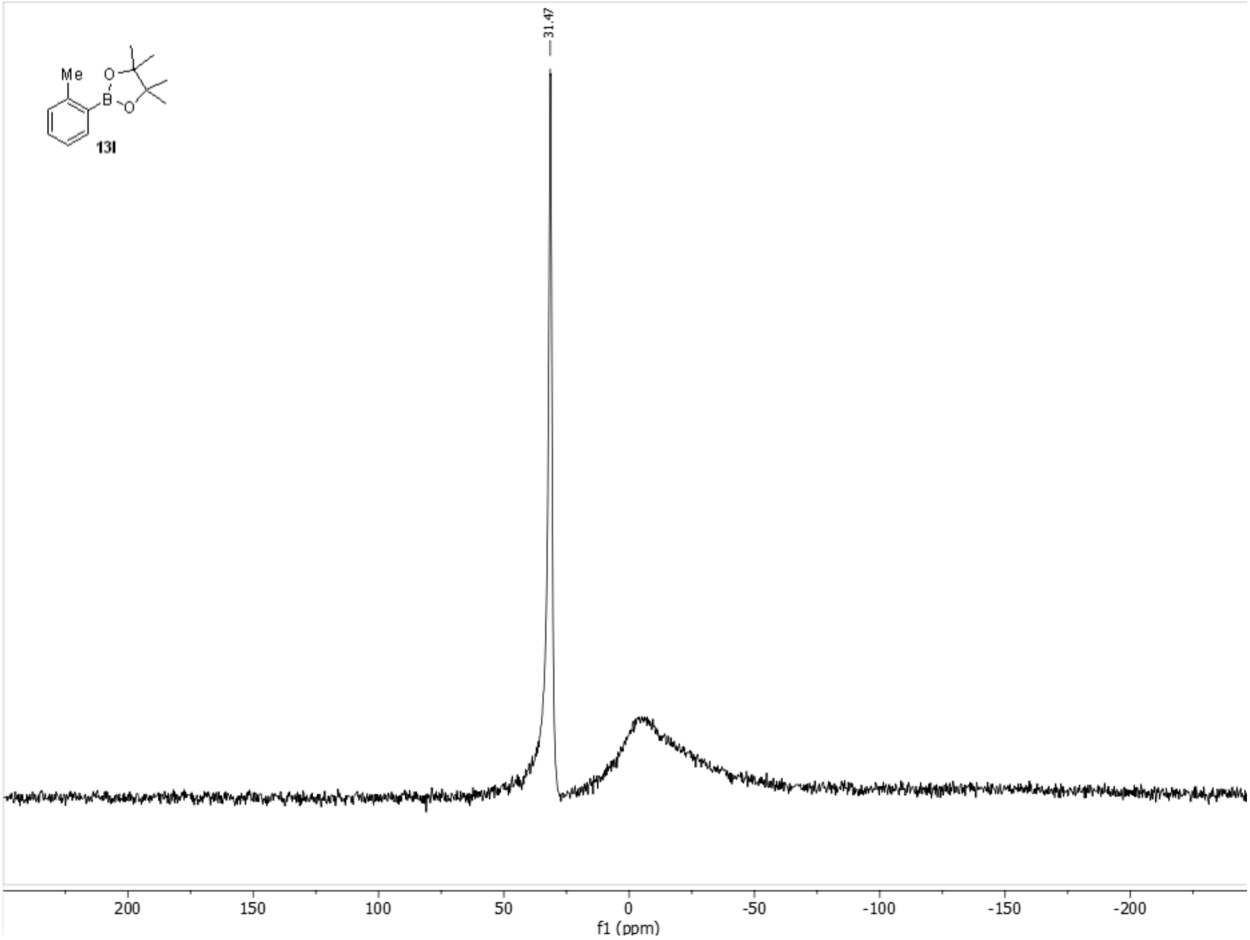

3-(4,4,5,5-Tetramethyl-1,3,2-dioxaborolan-2-yl)phenol 13m

^{1}H NMR (400 MHz, $CDCl_3$)

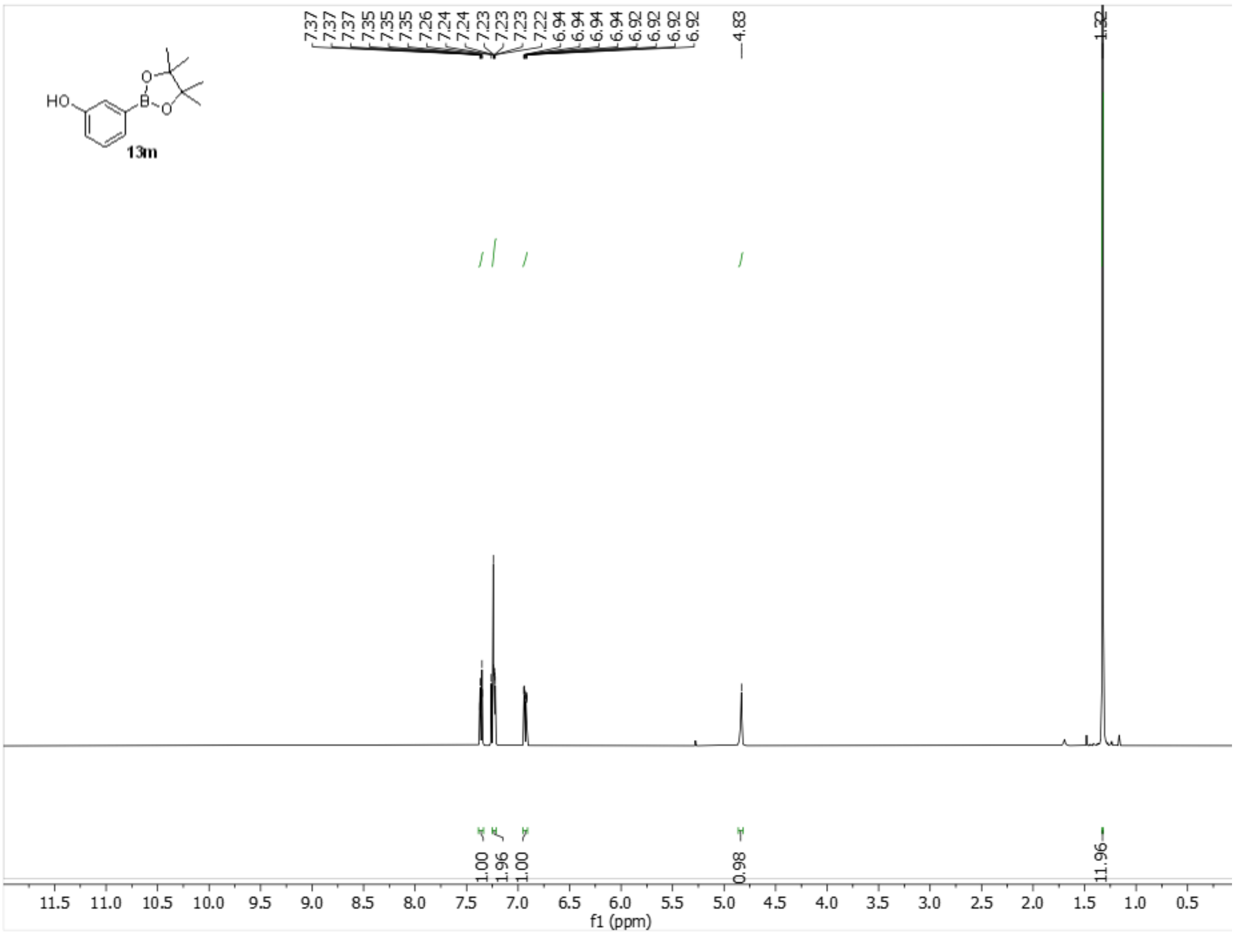

^{13}C NMR (101 MHz, $CDCl_3$)

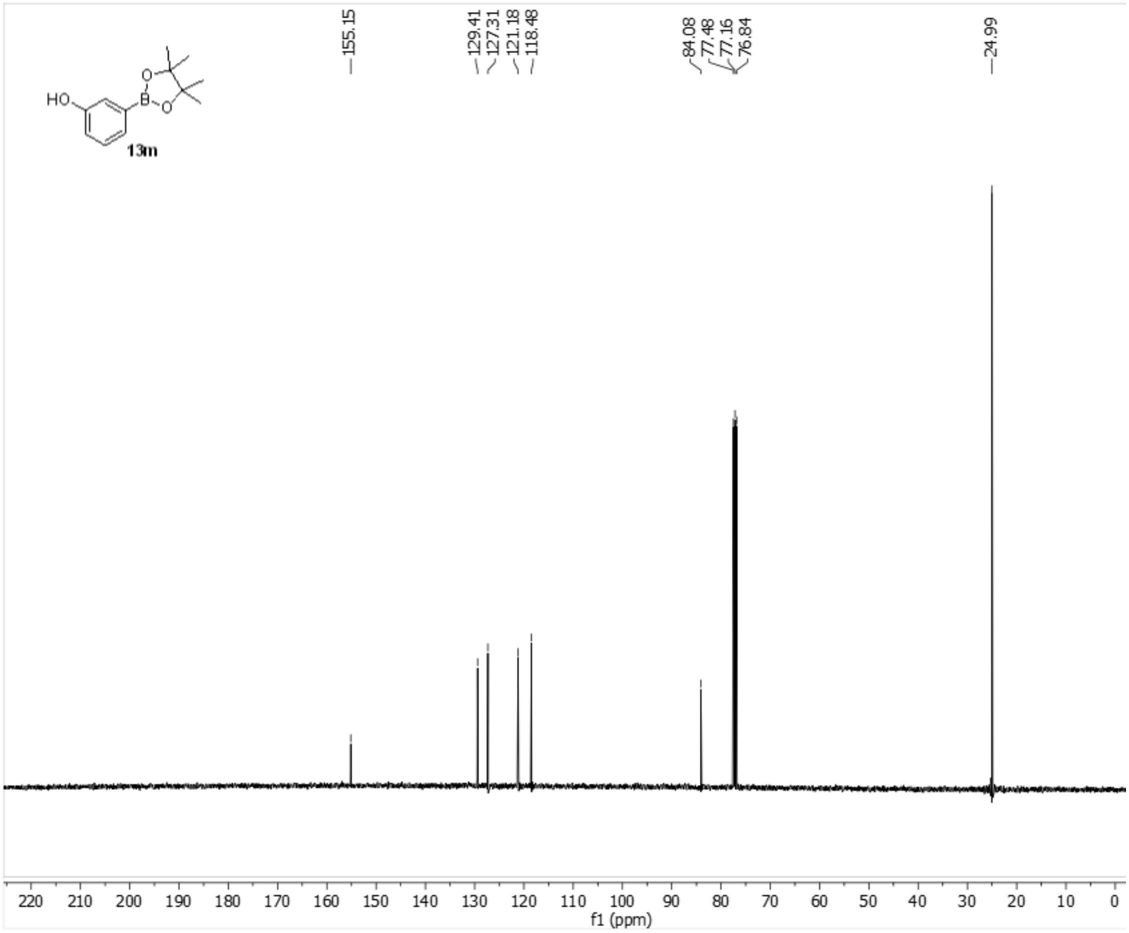

^{11}B NMR (128 MHz, $CDCl_3$)

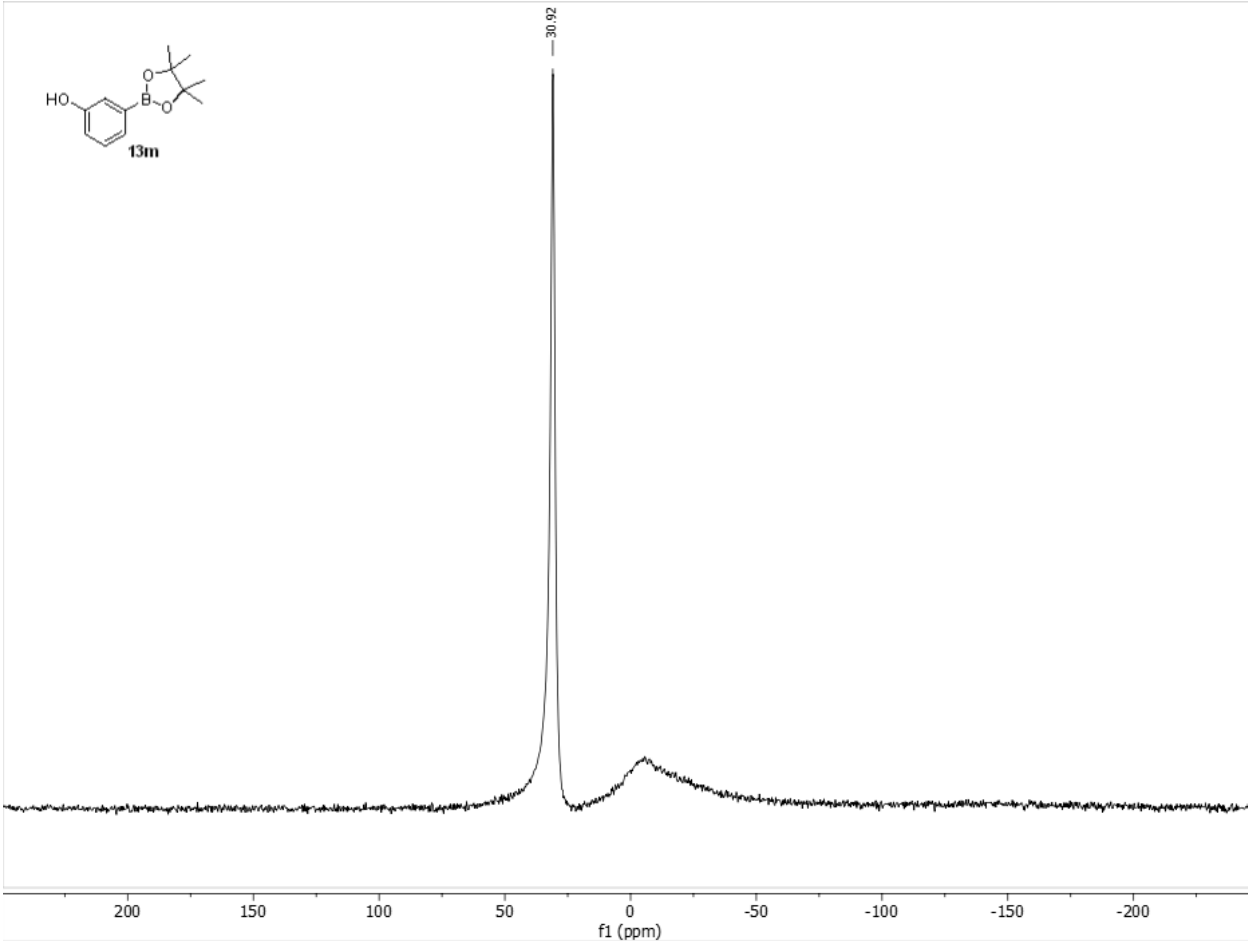

2-(3-Methoxyphenyl)-4,4,5,5-tetramethyl-1,3,2-dioxaborolane 13n

^{1}H NMR (400 MHz, $CDCl_3$)

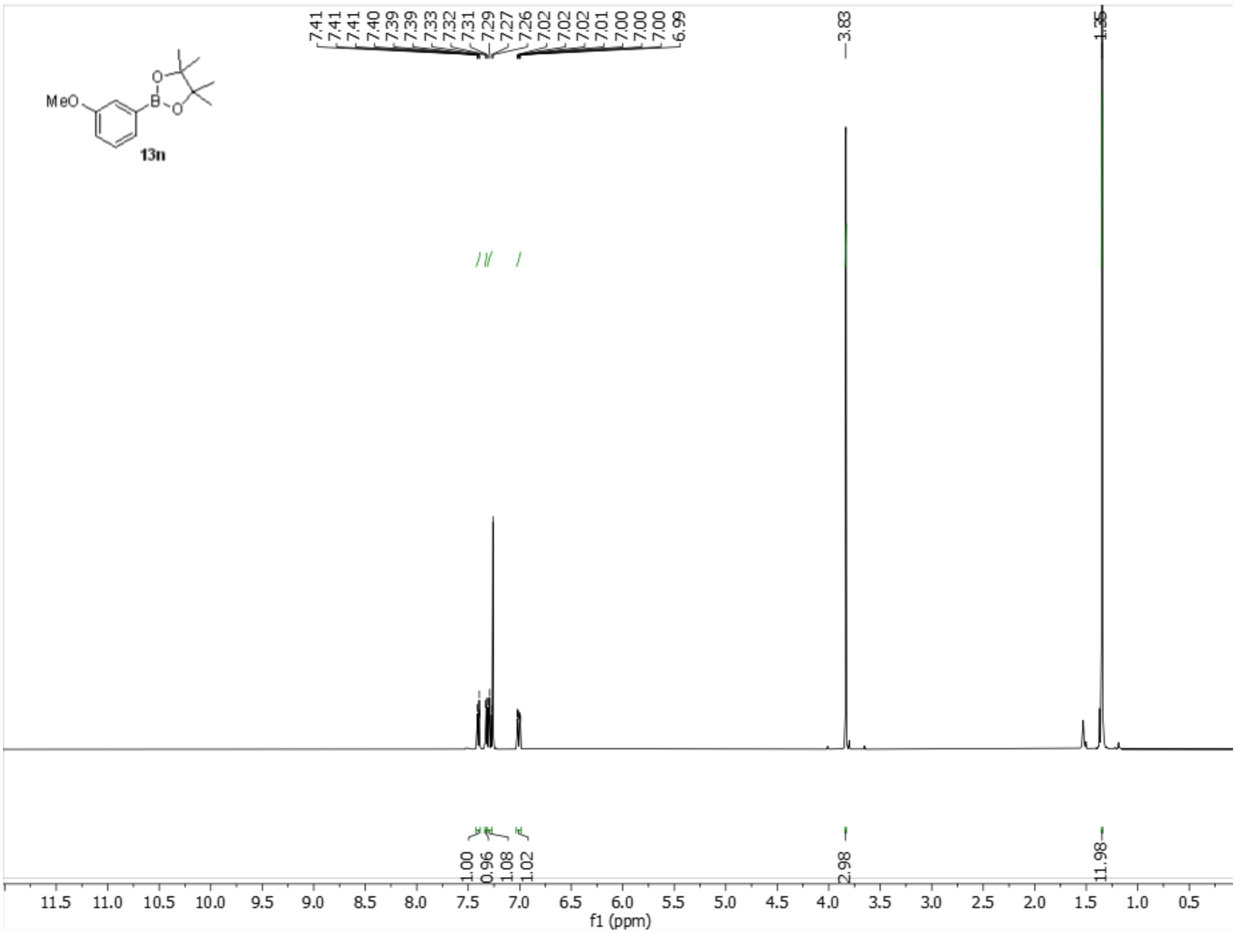

^{13}C NMR (101 MHz, $CDCl_3$)

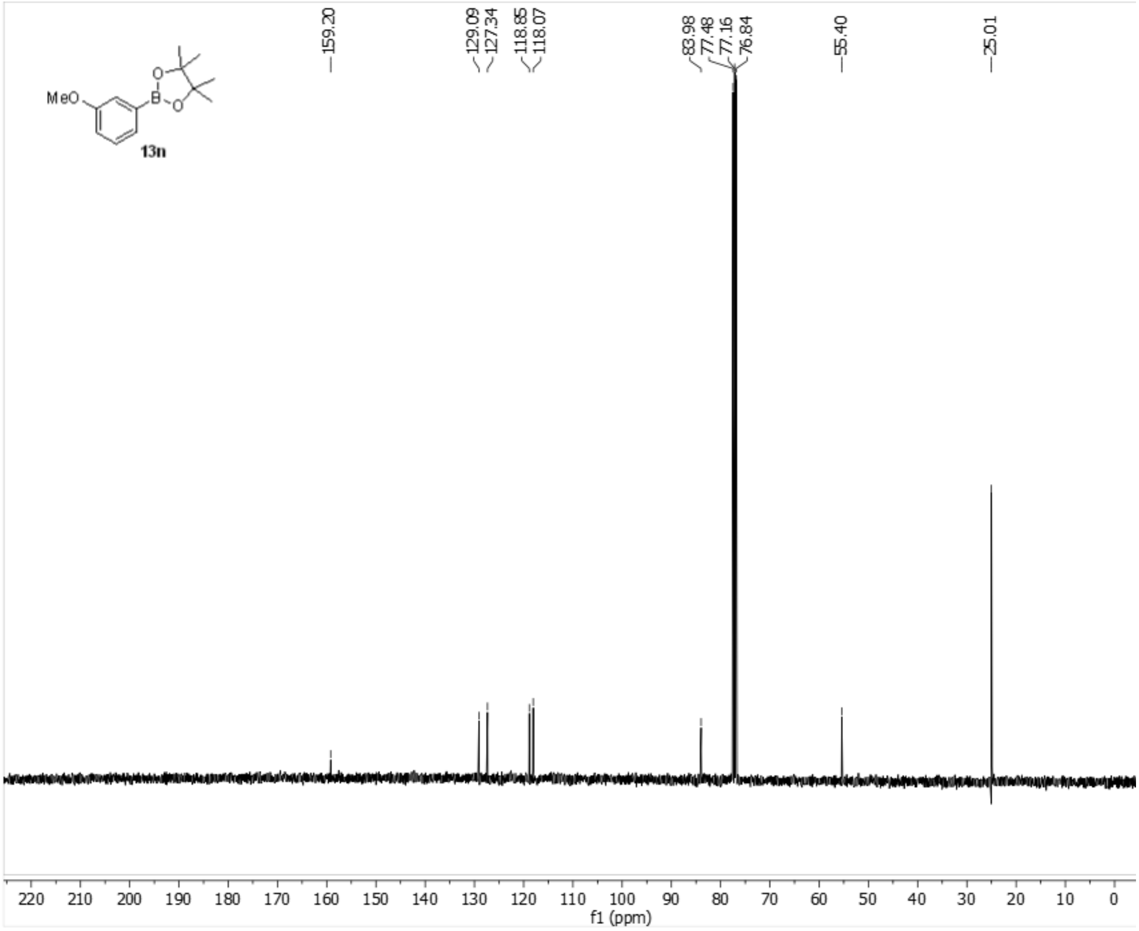

^{11}B NMR (128 MHz, $CDCl_3$)

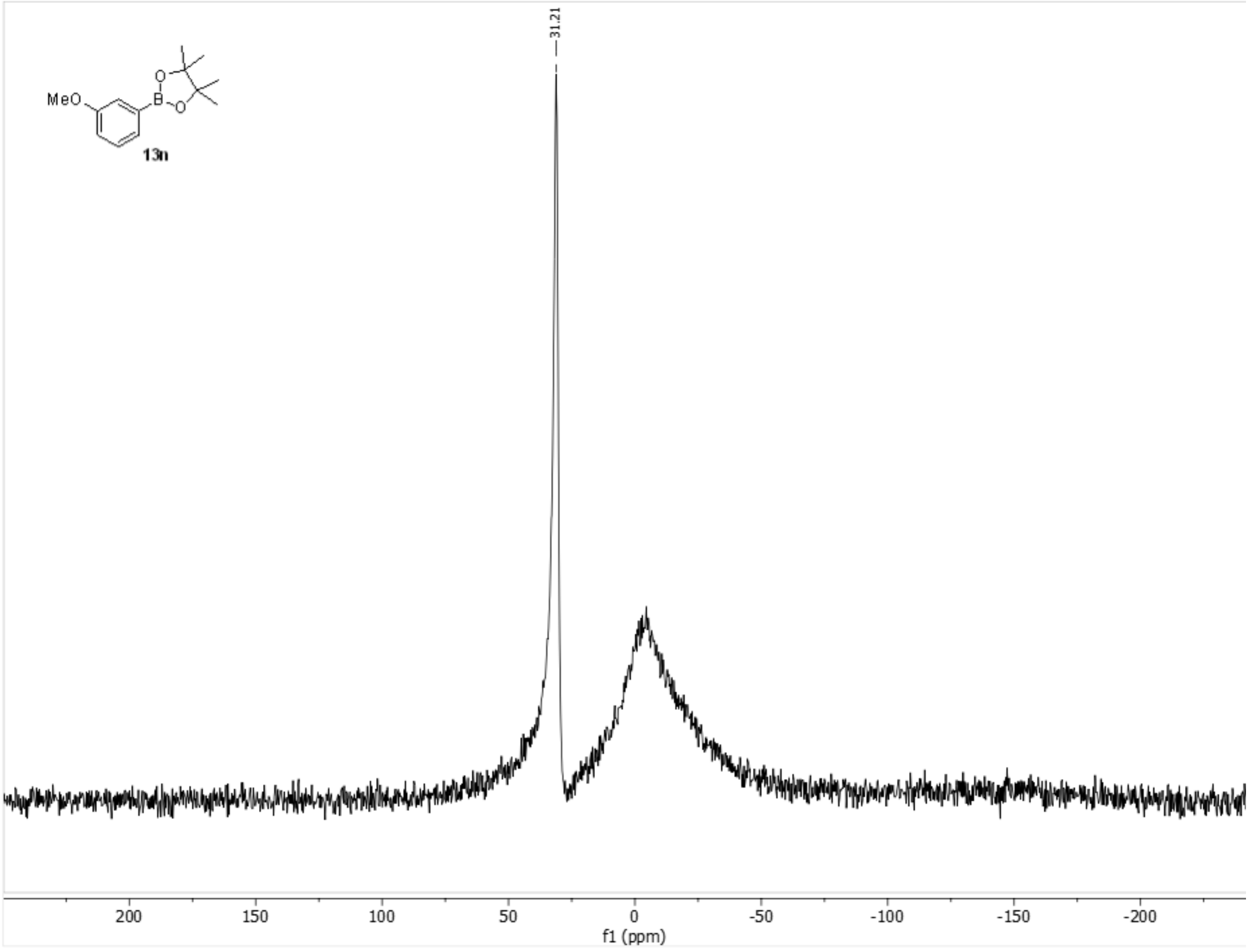

4,4,5,5-Tetramethyl-2-(*m*-tolyl)-1,3,2-dioxaborolane 13o

^{1}H NMR (400 MHz, $CDCl_3$)

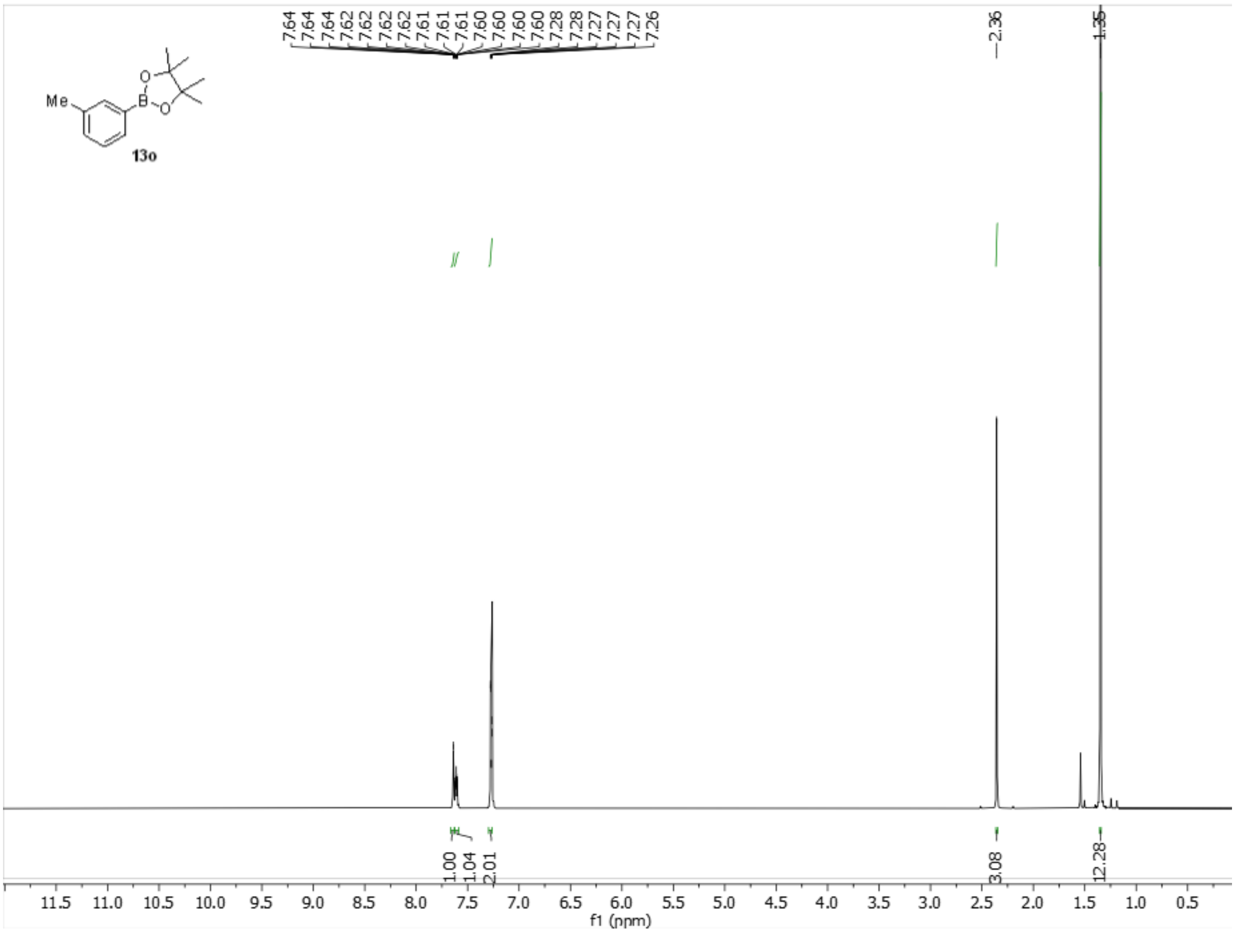

^{13}C NMR (101 MHz, $CDCl_3$)

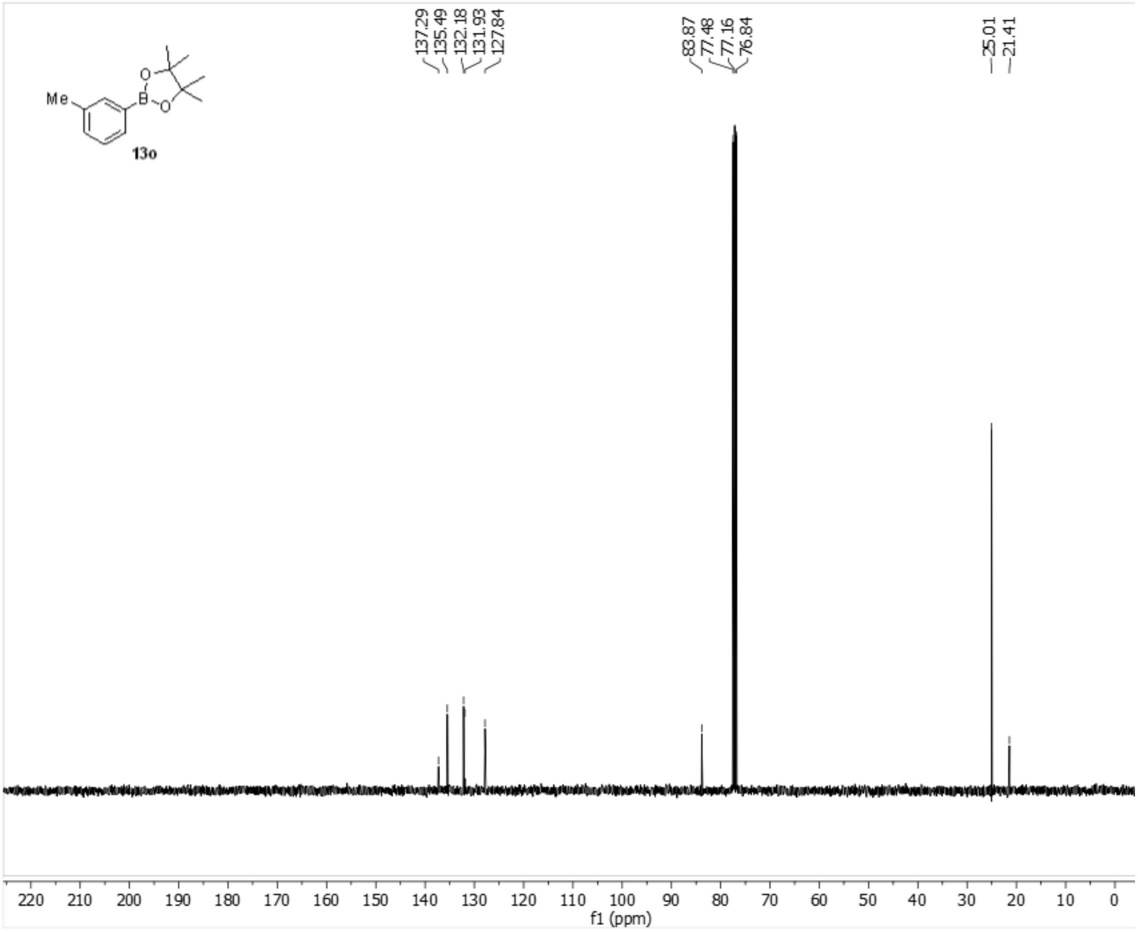

^{11}B NMR (128 MHz, $CDCl_3$)

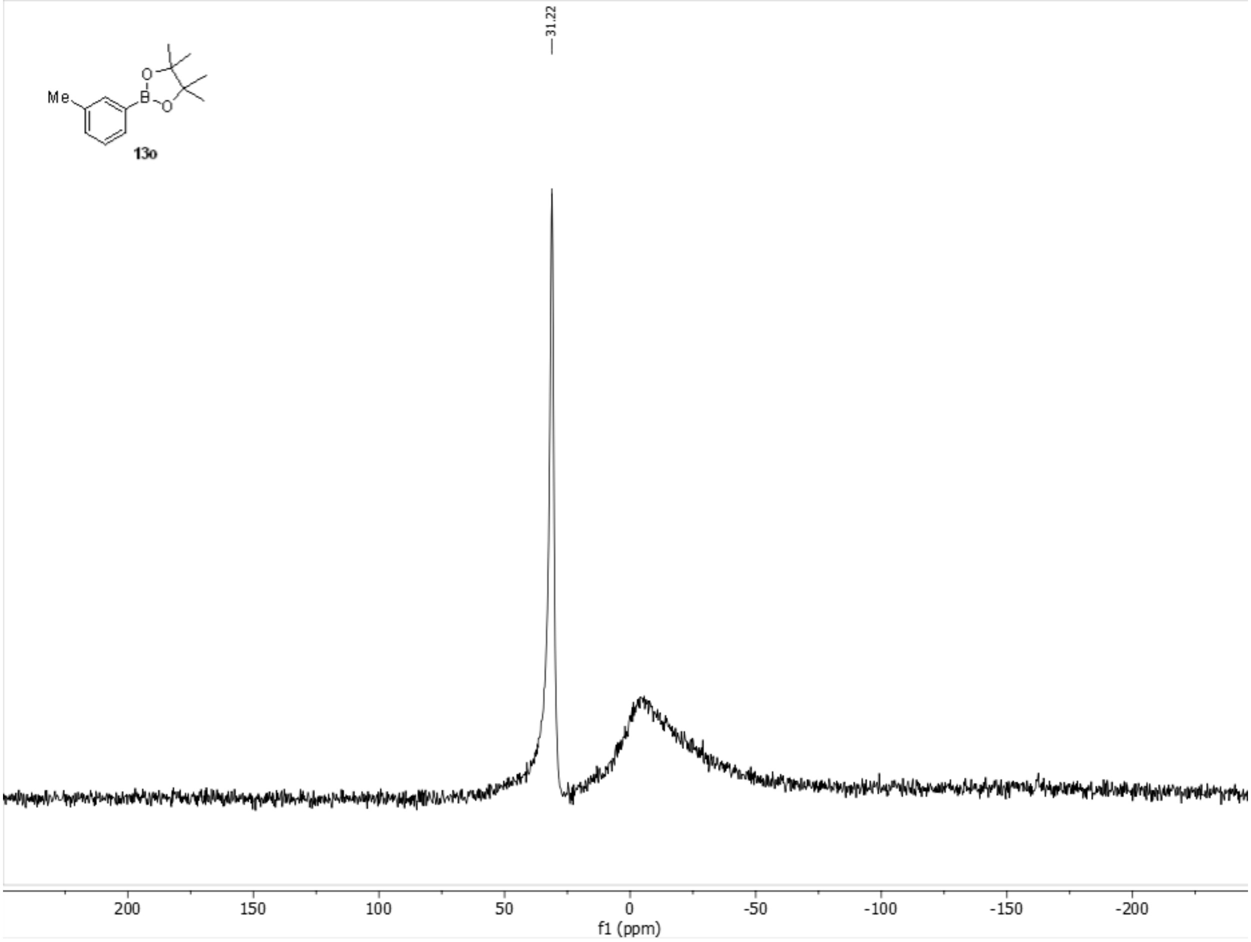

2-(3,5-Dimethylphenyl)-4,4,5,5-tetramethyl-1,3,2-dioxaborolane 13p

^{1}H NMR (400 MHz, $CDCl_3$)

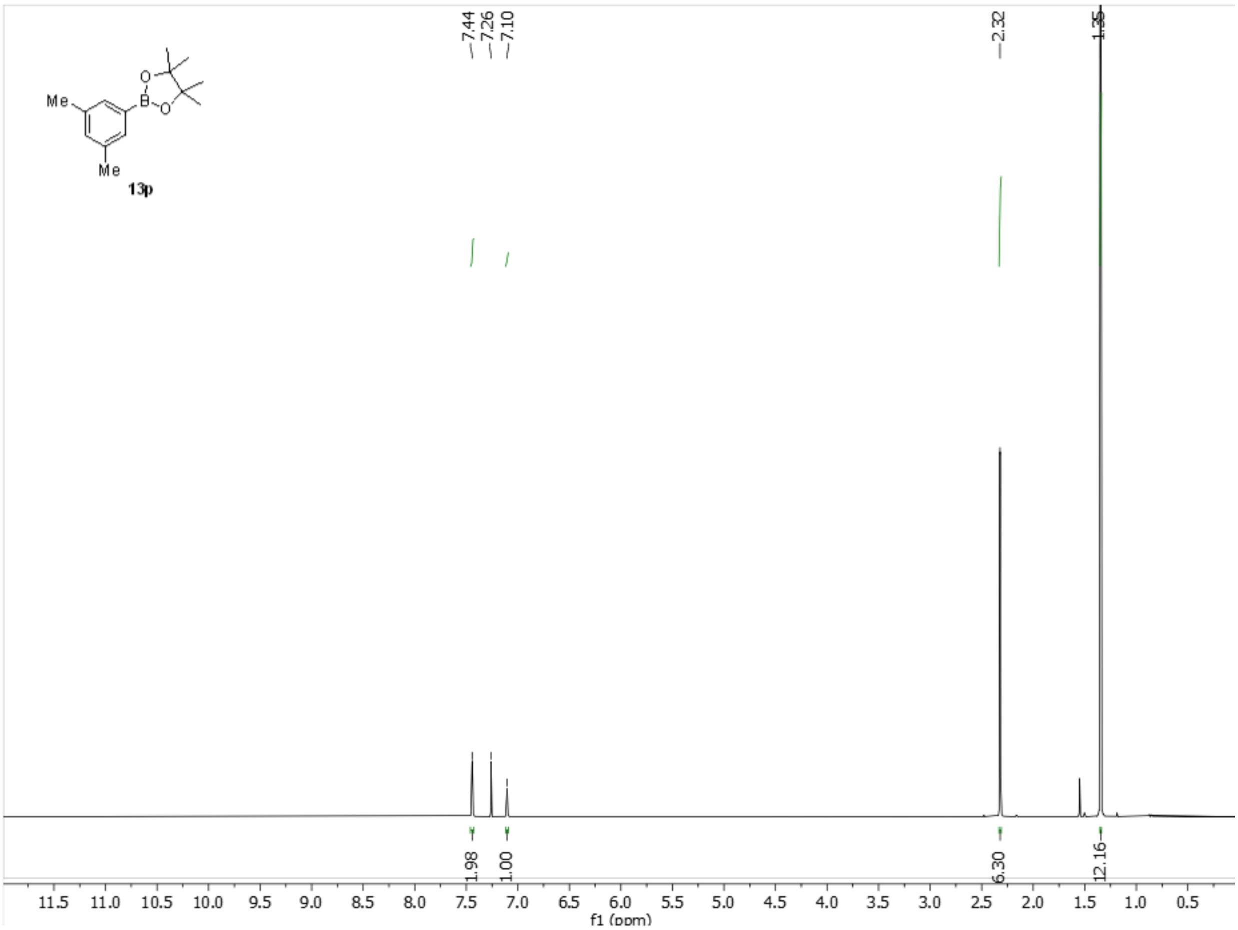

^{13}C NMR (101 MHz, $CDCl_3$)

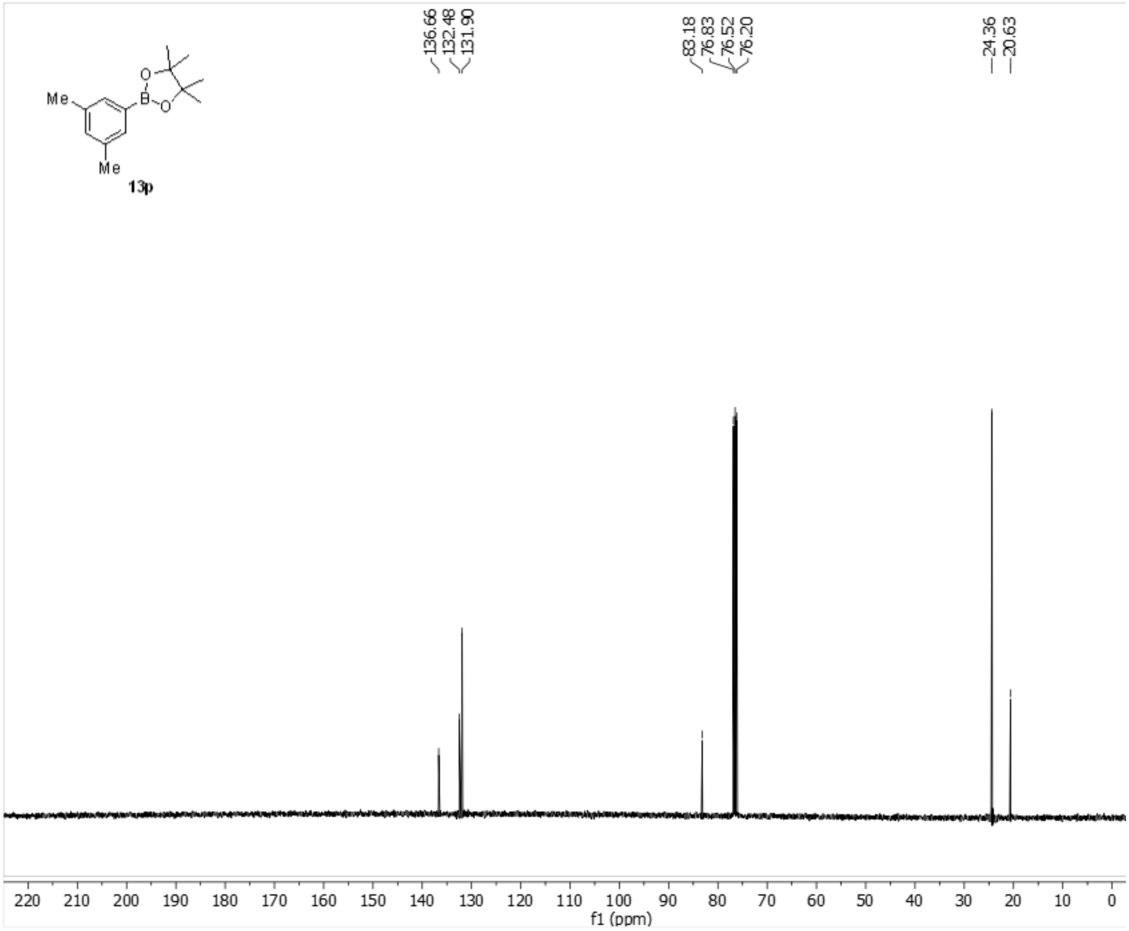

^{11}B NMR (128 MHz, $CDCl_3$)

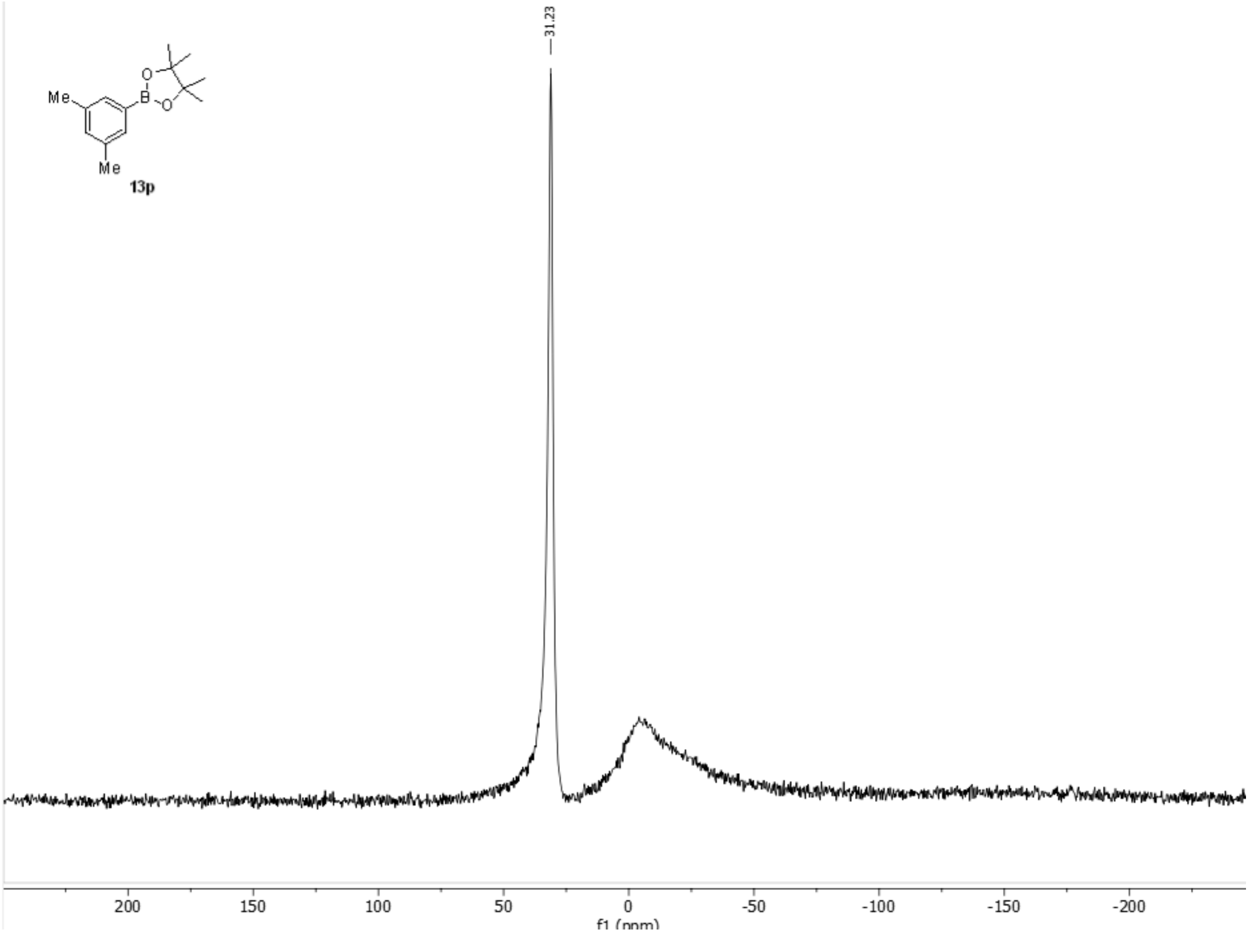

2-(3-Fluoro-4-methoxyphenyl)-4,4,5,5-tetramethyl-1,3,2-dioxaborolane 13q

^{1}H NMR (400 MHz, $CDCl_3$)

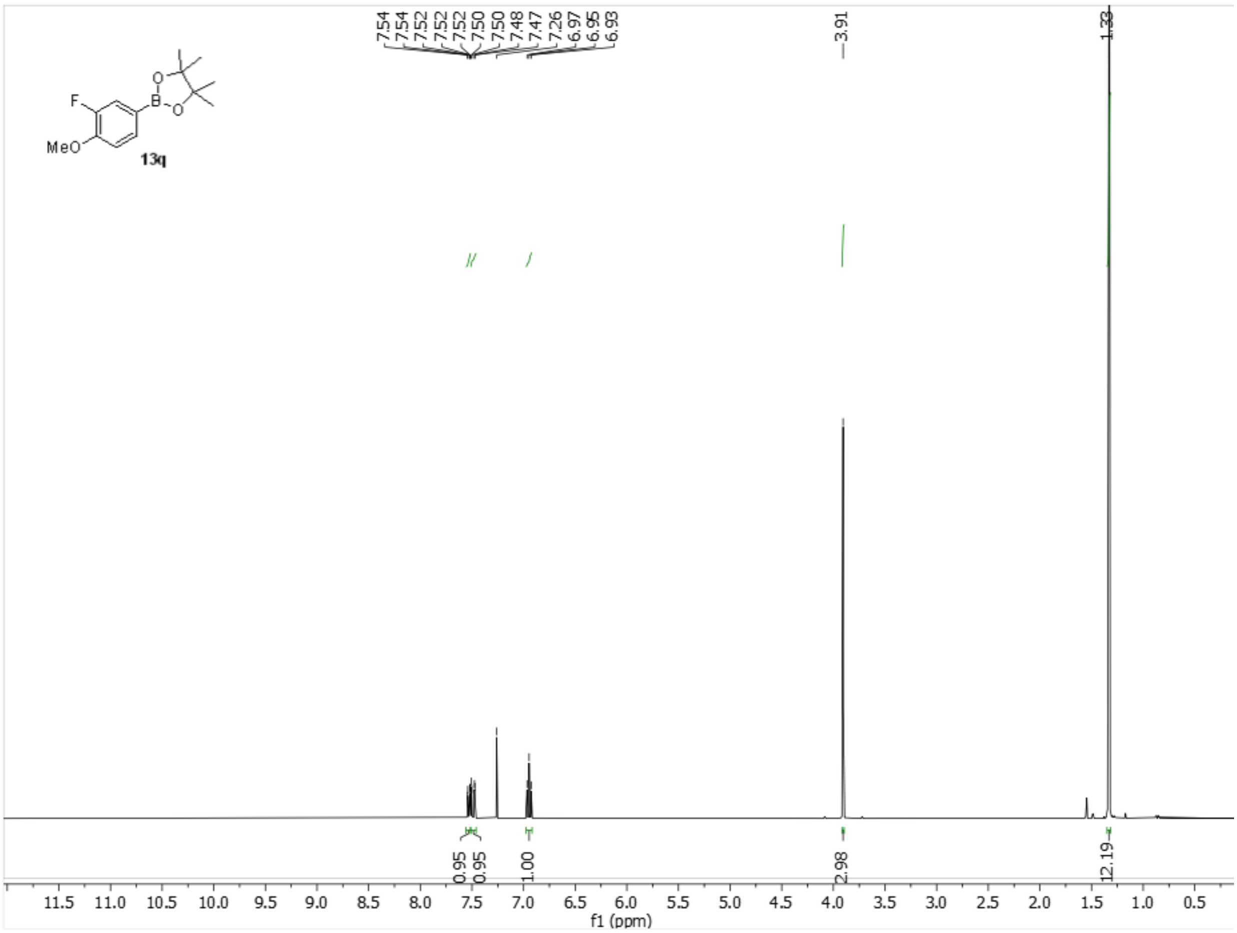

^{13}C NMR (101 MHz, $CDCl_3$)

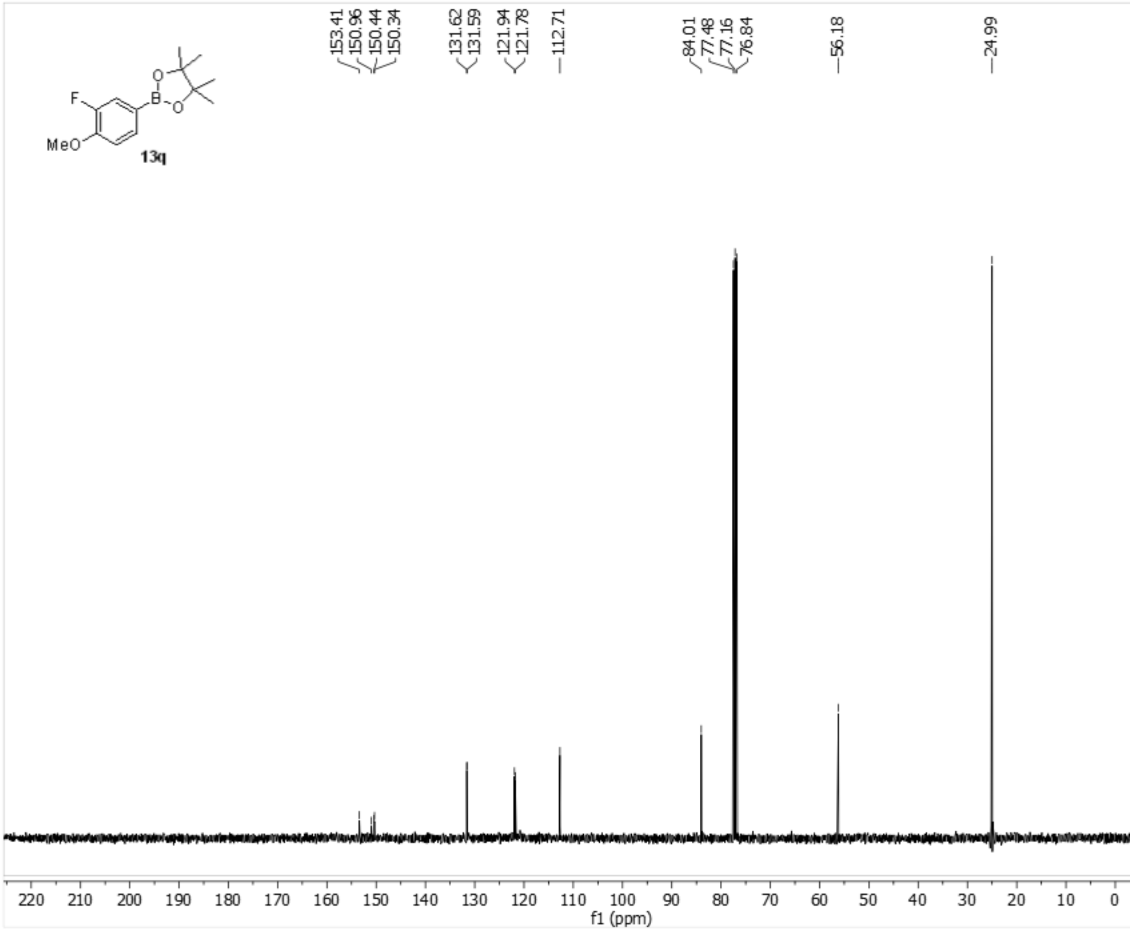

^{11}B NMR (128 MHz, $CDCl_3$)

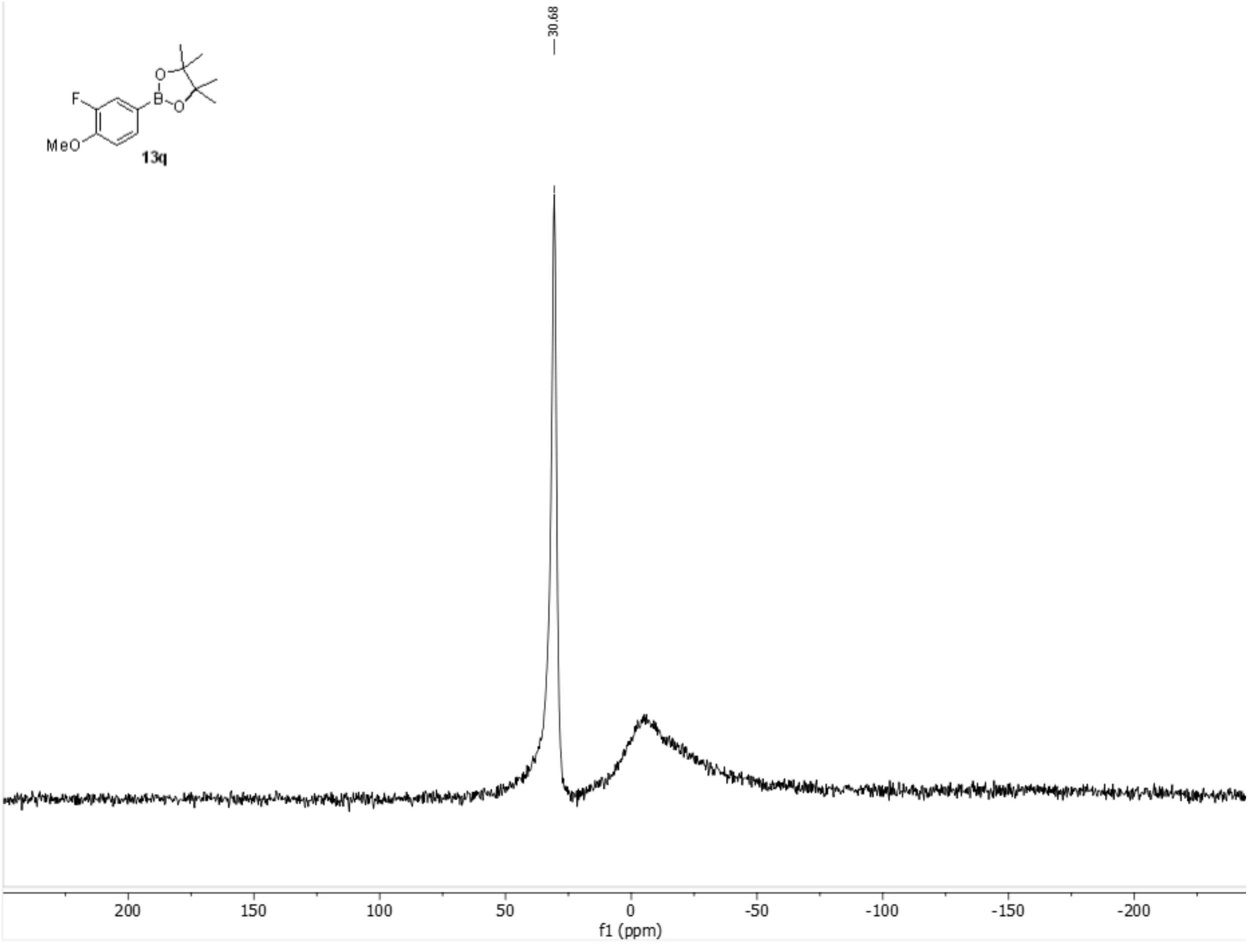

^{19}F NMR (376 MHz, $CDCl_3$)

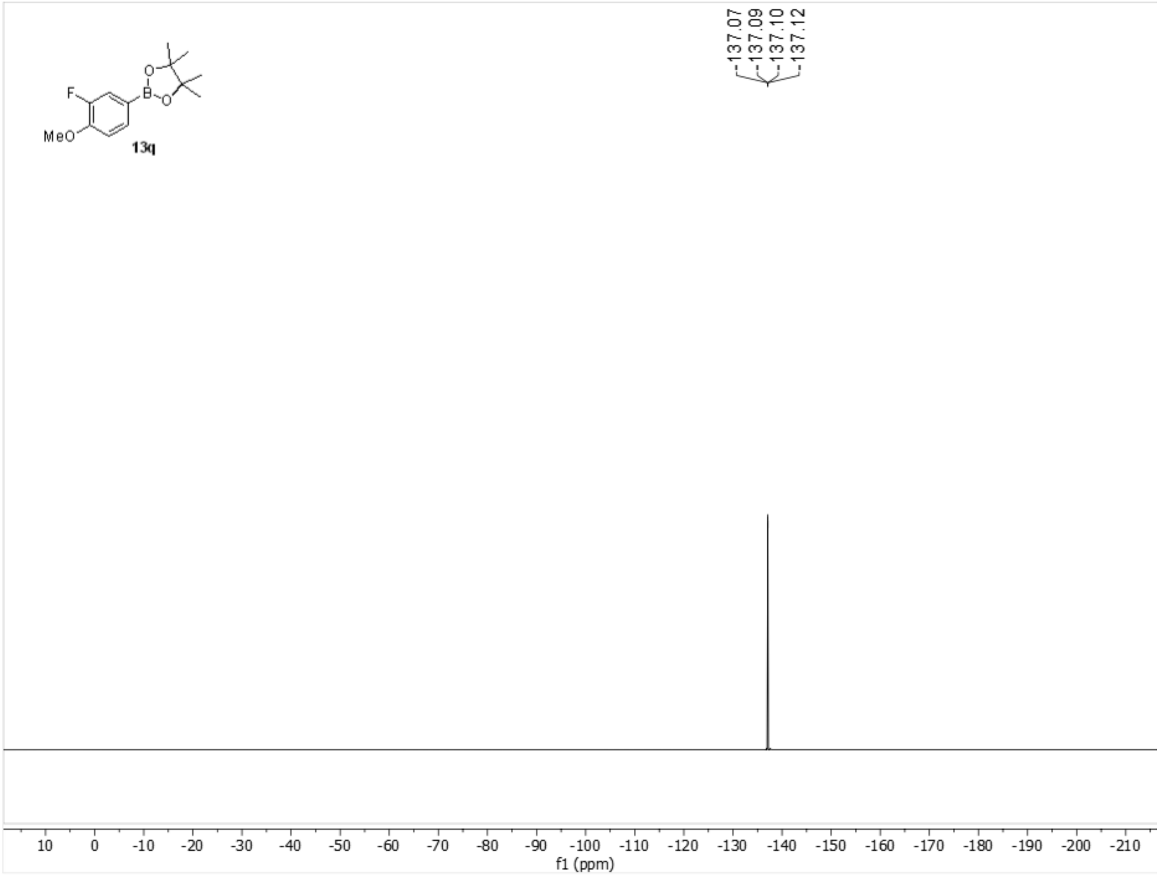

^{19}F NMR (376 MHz, $CDCl_3$) - expanded

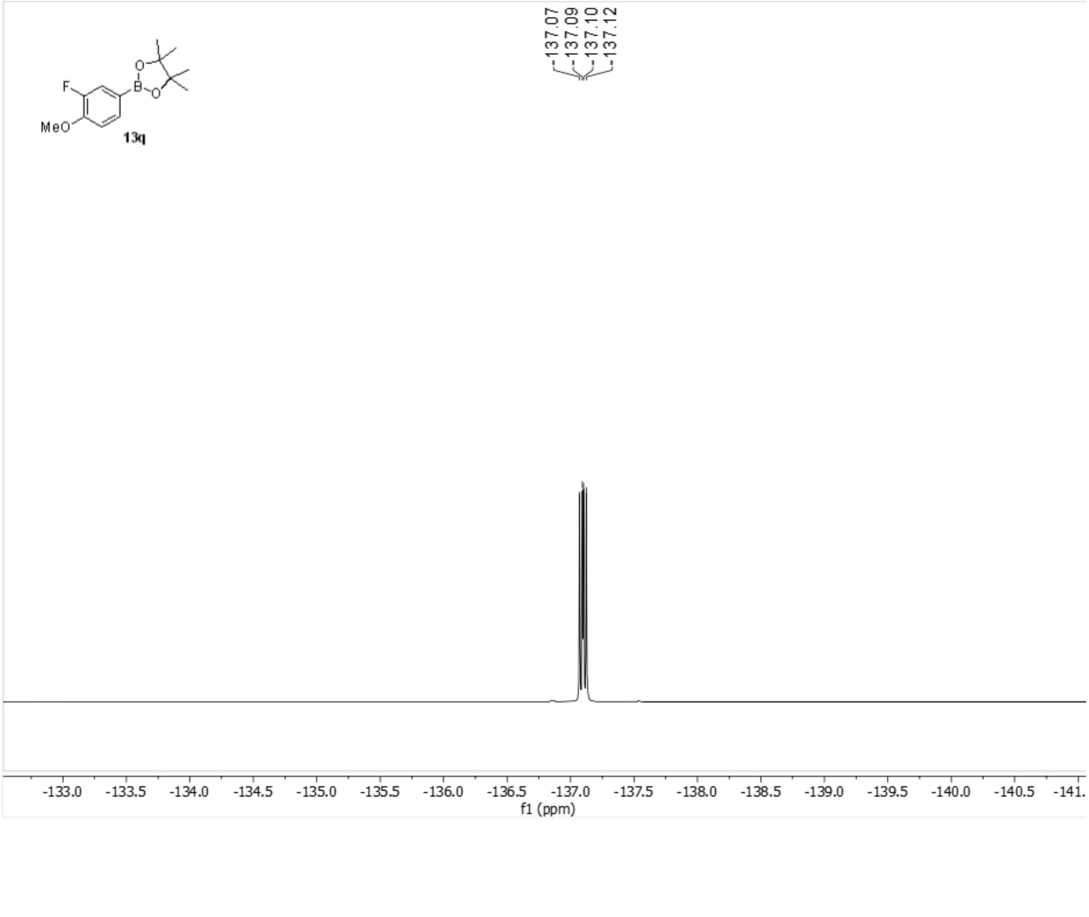

1-Methyl-5-(4,4,5,5-tetramethyl-1,3,2-dioxaborolan-2-yl)-1*H*-indole 13r

^{1}H NMR (400 MHz, $CDCl_3$)

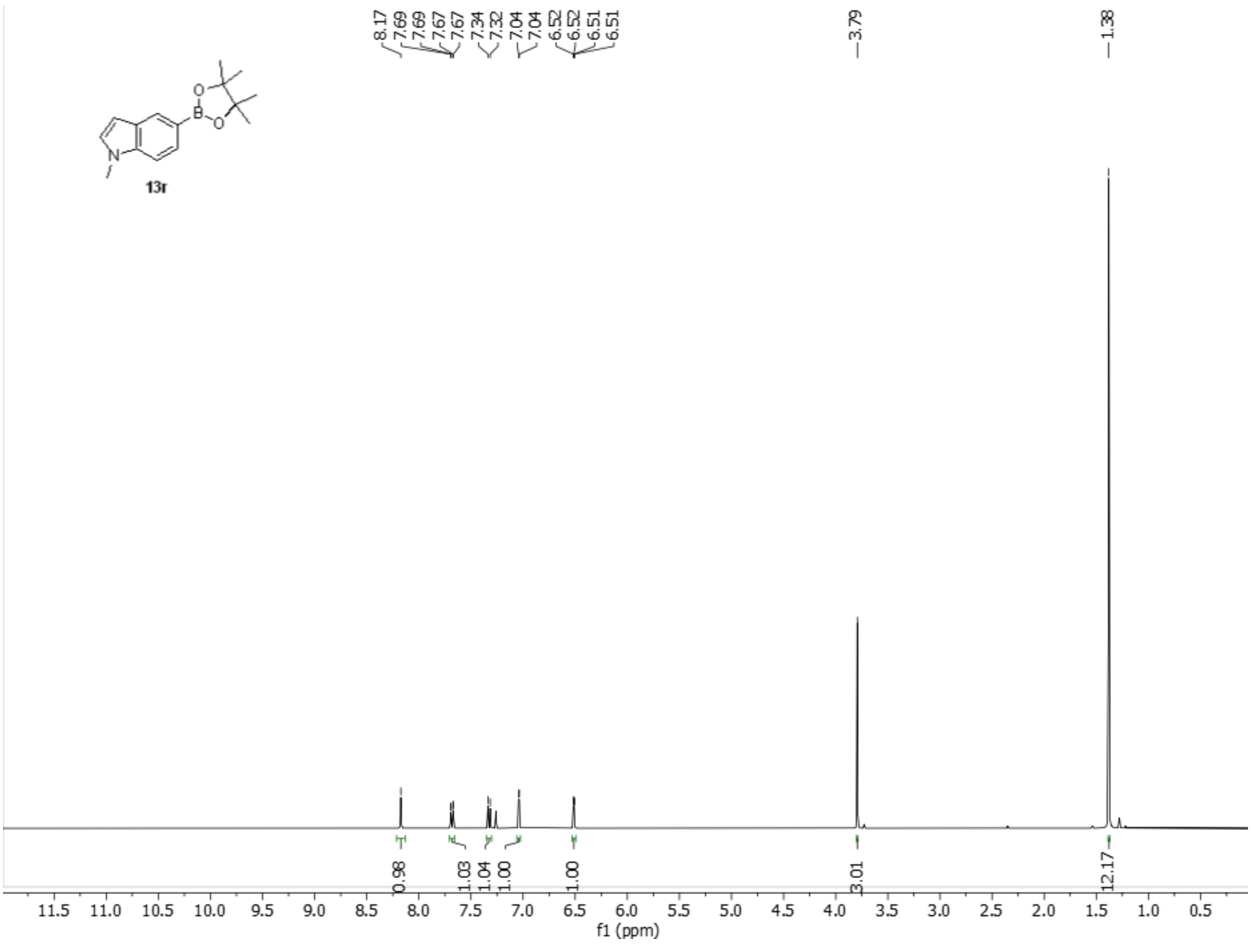

^{13}C NMR (101 MHz, $CDCl_3$)

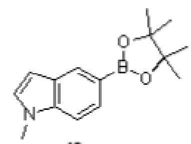

13r

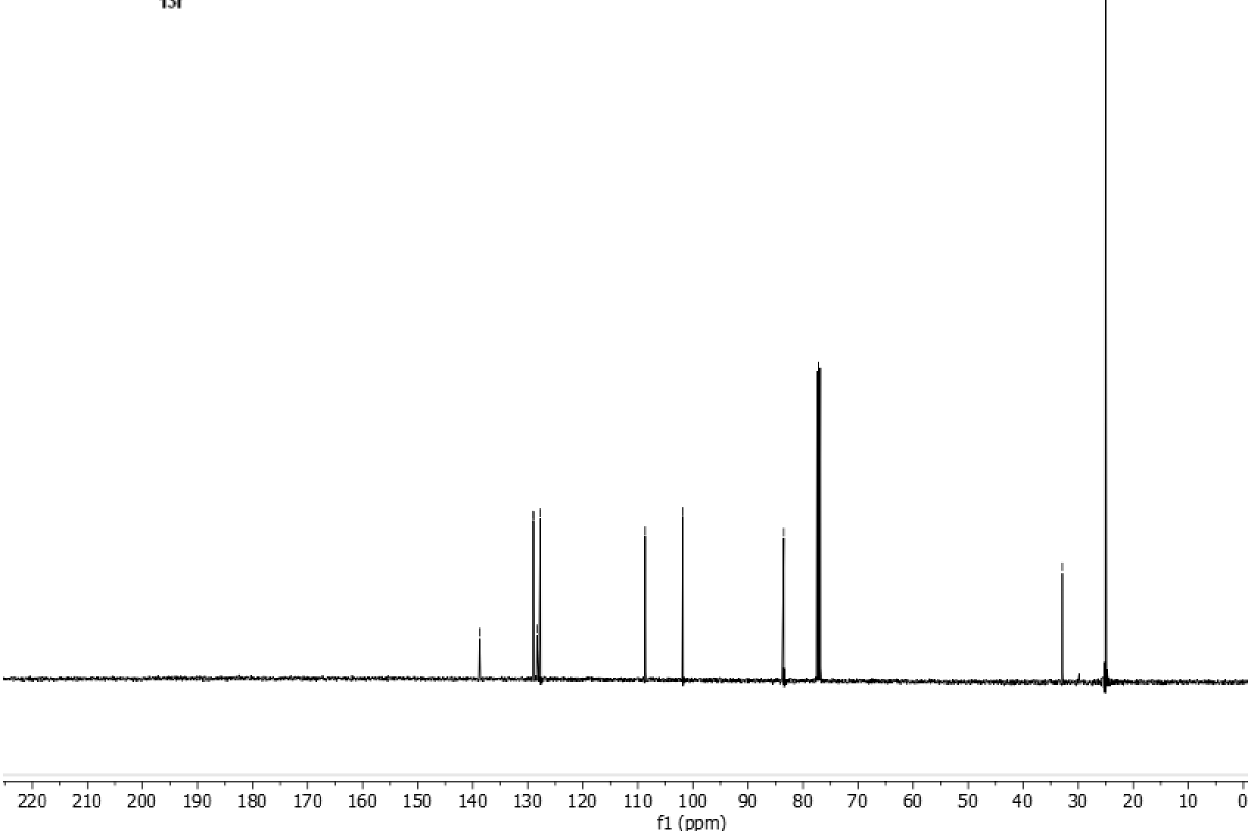

^{11}B NMR (128 MHz, $CDCl_3$)

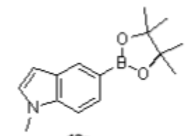

13r

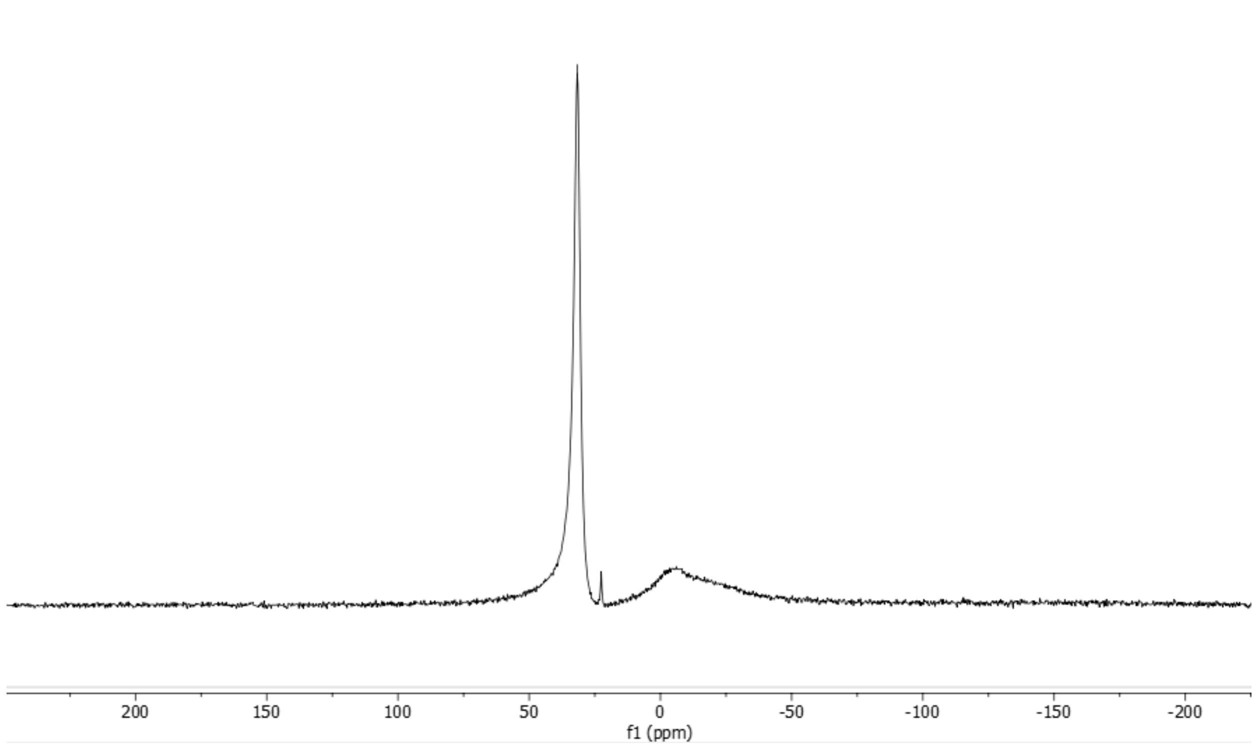

2-(4-Iodophenyl)-4,4,5,5-tetramethyl-1,3,2-dioxaborolane 13s

^{1}H NMR (400 MHz, $CDCl_3$)

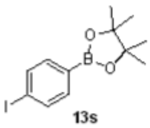

13s

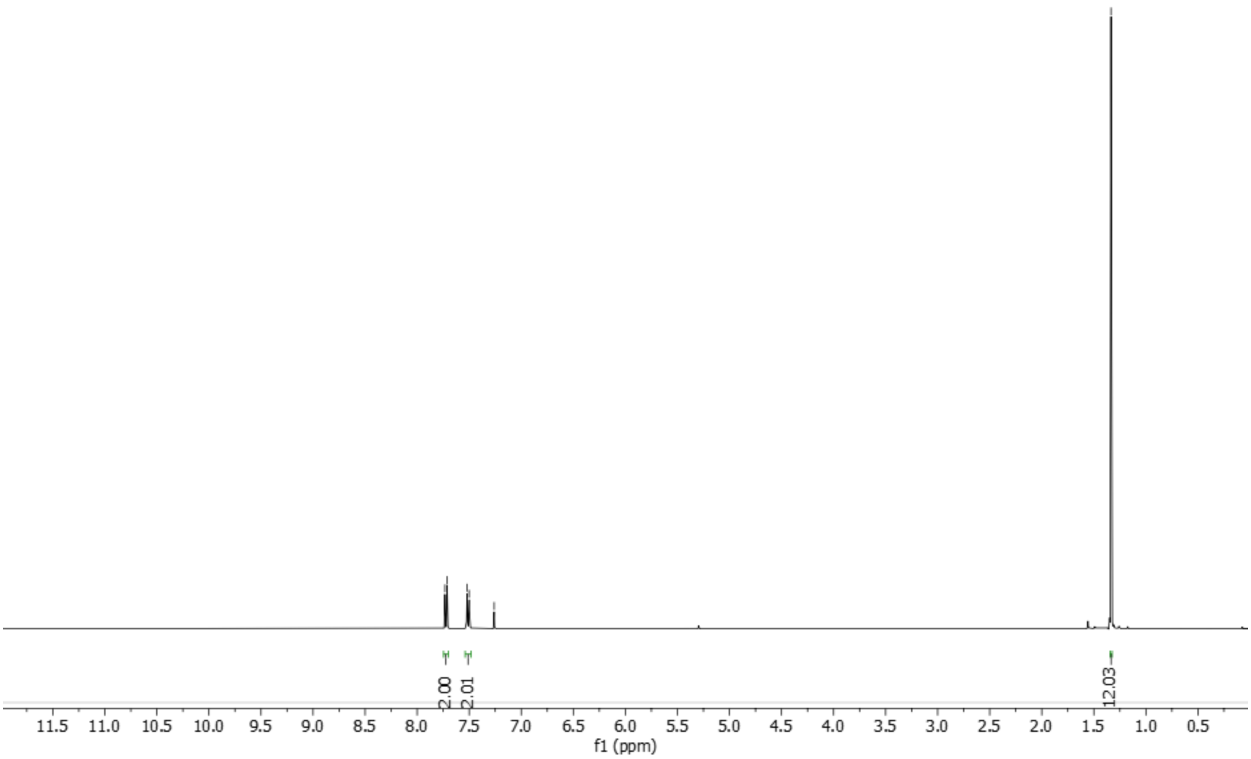

^{13}C NMR (101 MHz, CDCl$_3$)

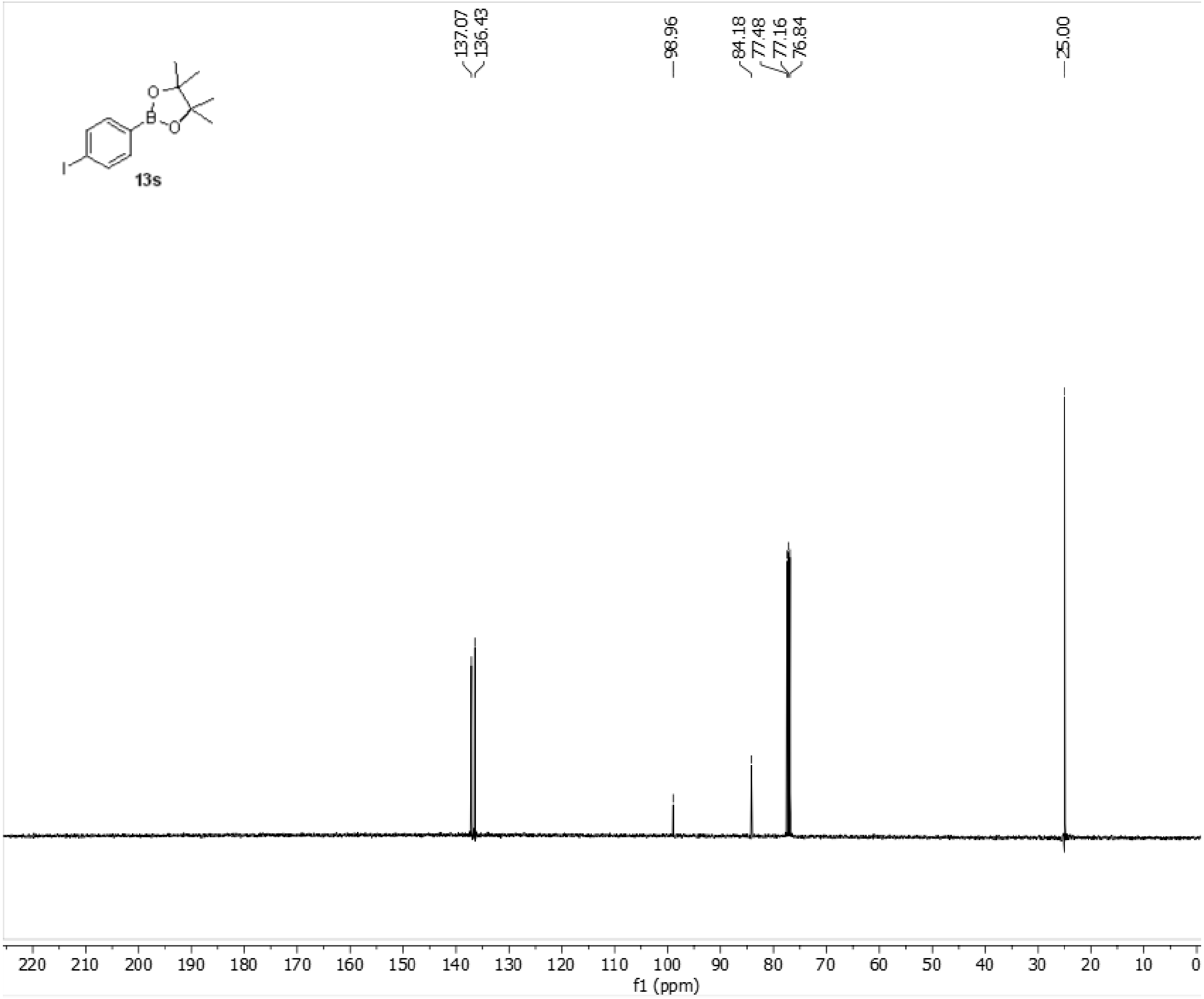

^{11}B NMR (128 MHz, $CDCl_3$)

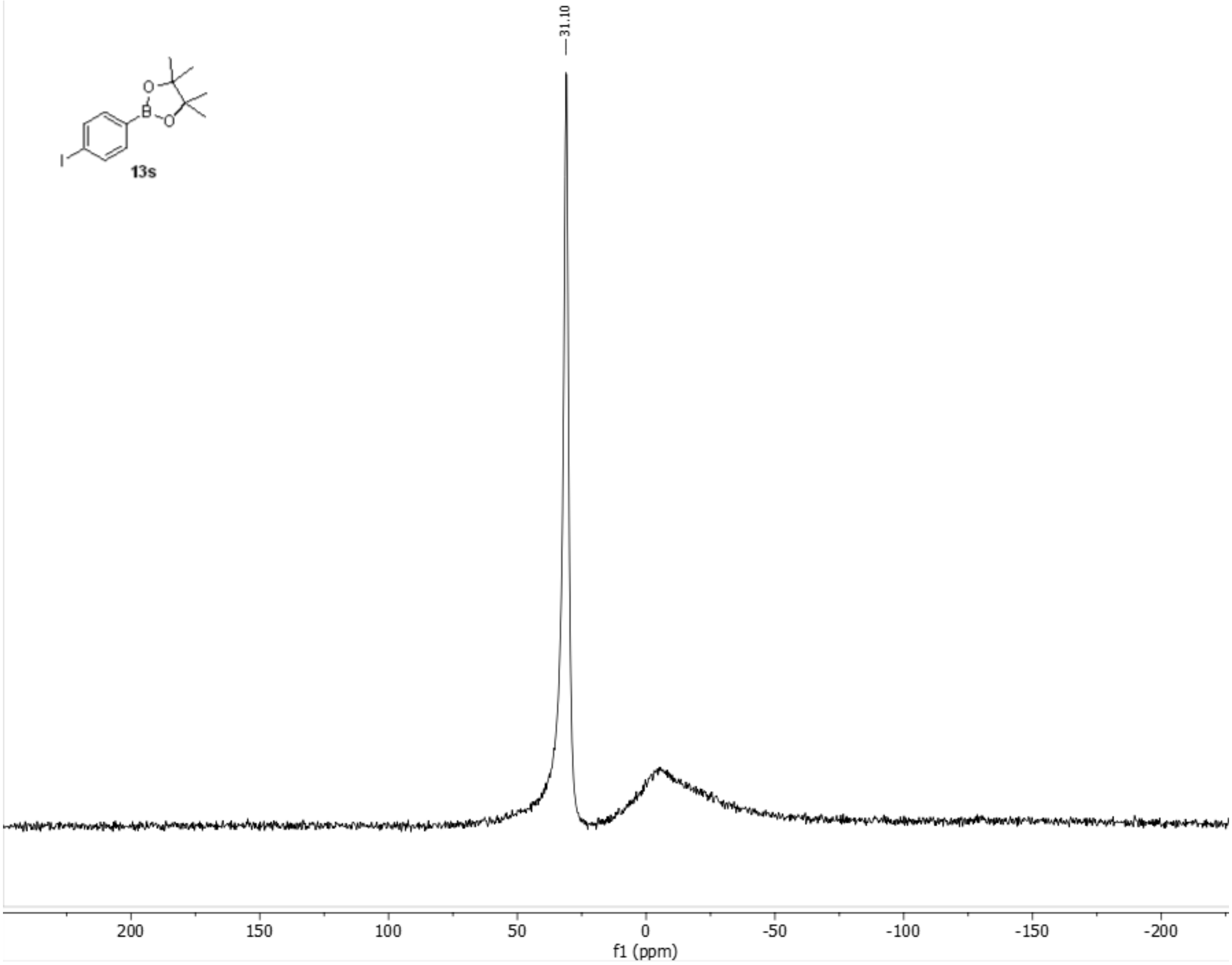

4,4,5,5-Tetramethyl-2-(4-(methylthio)phenyl)-1,3,2-dioxaborolane 13t

^1H NMR (400 MHz, $CDCl_3$)

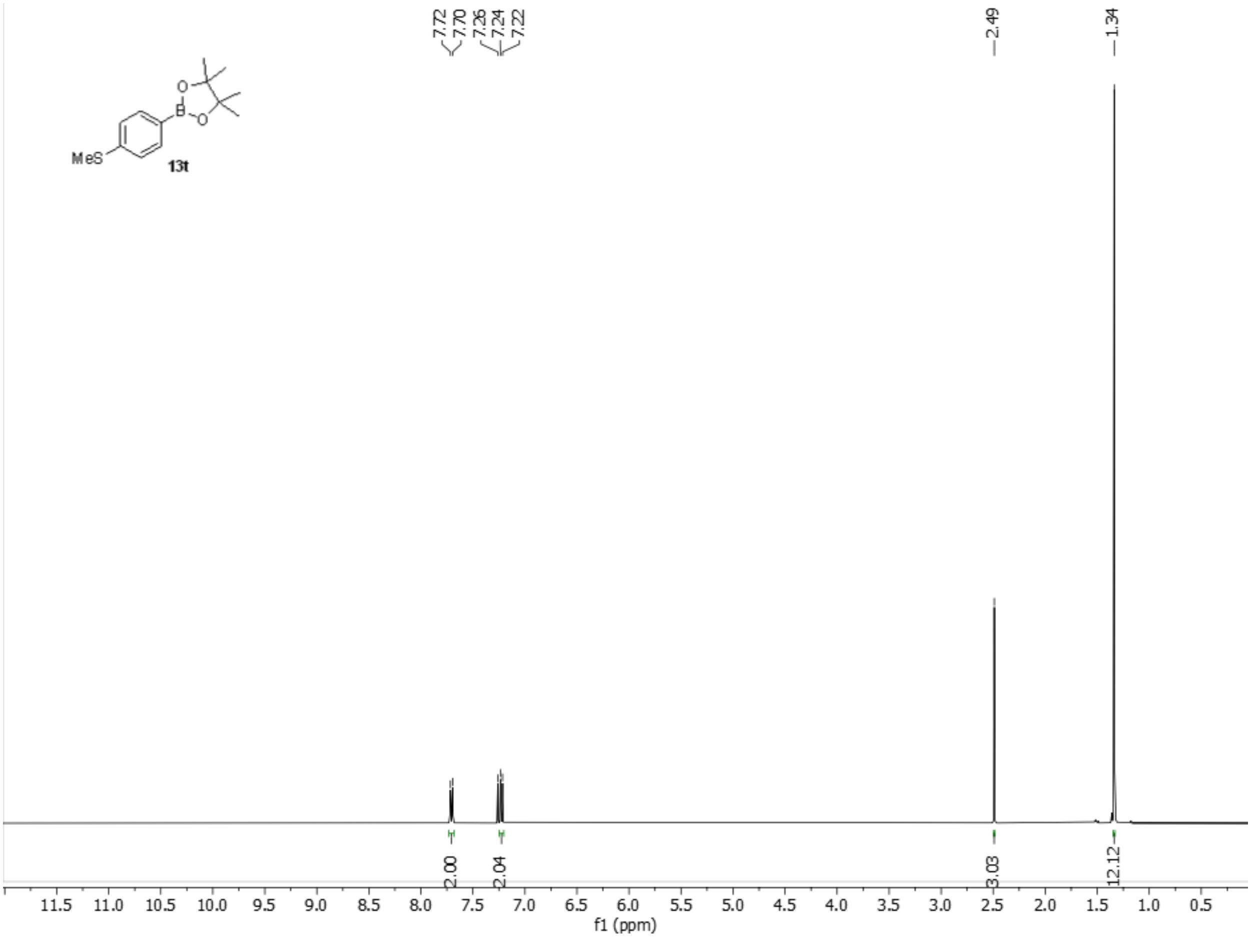

^{13}C NMR (101 MHz, $CDCl_3$)

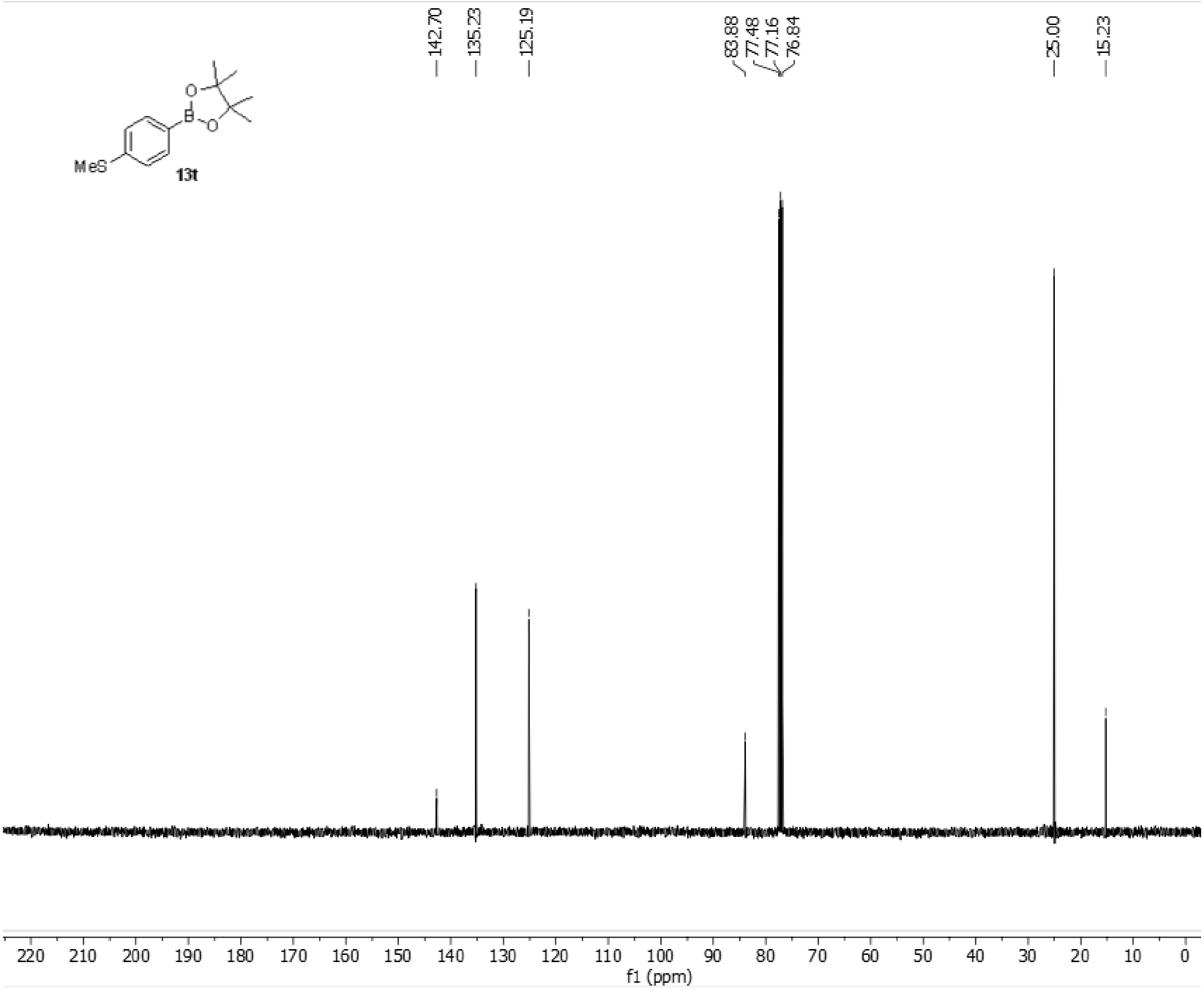

^{11}B NMR (128 MHz, $CDCl_3$)

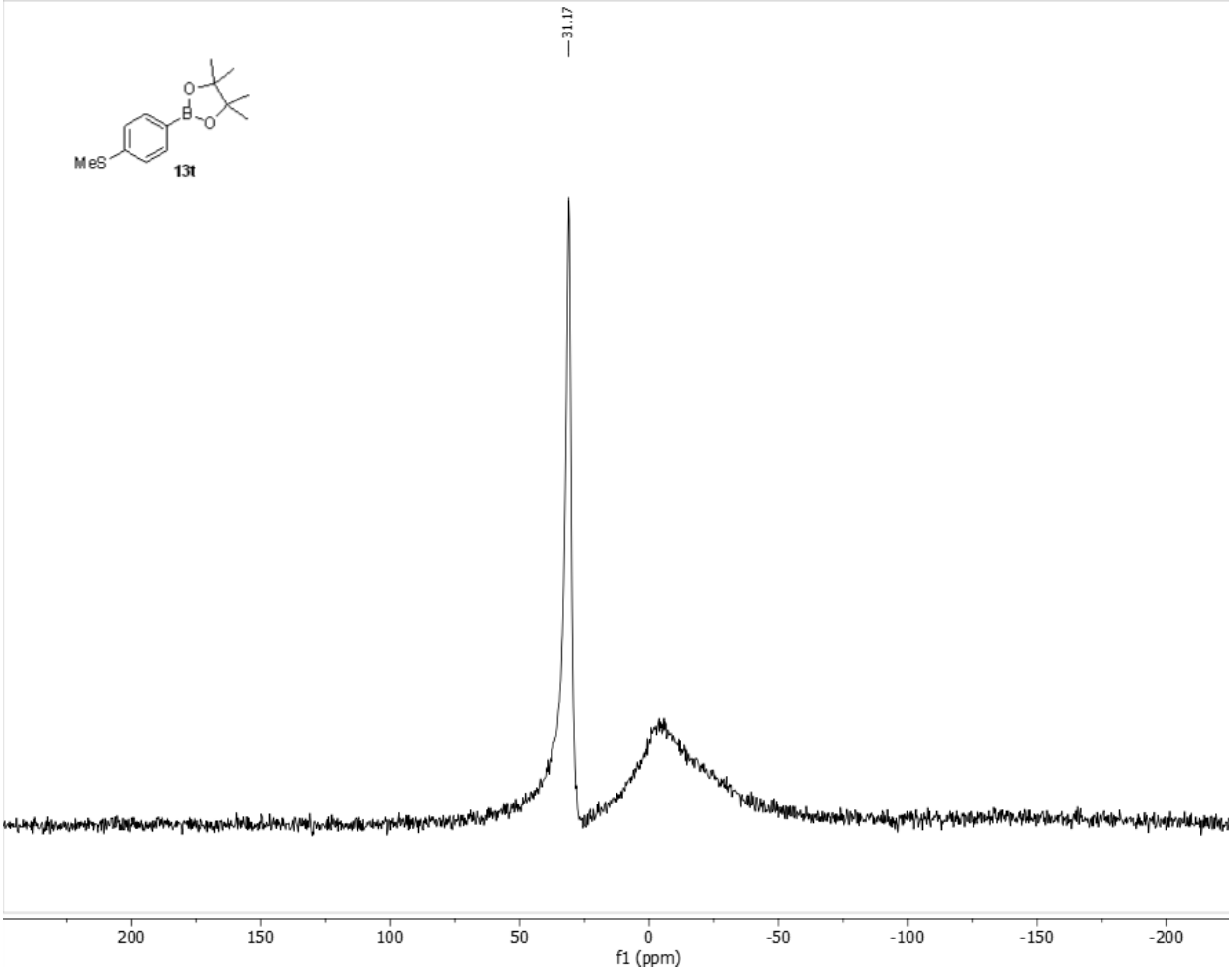

2-(4-Methoxyphenyl)-5,5-dimethyl-1,3,2-dioxaborinane 14a

^{1}H NMR (400 MHz, $CDCl_3$)

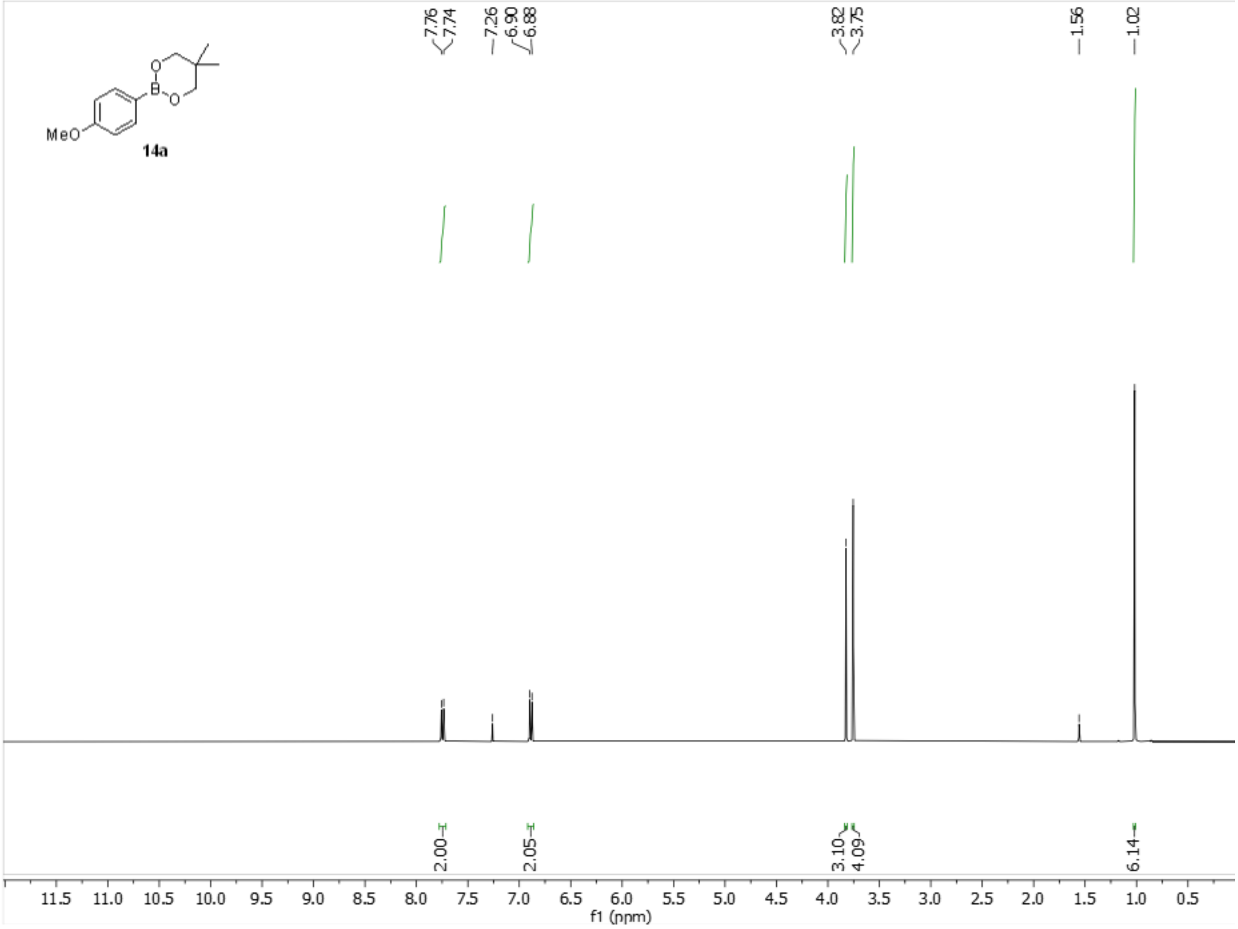

^{13}C NMR (101 MHz, $CDCl_3$)

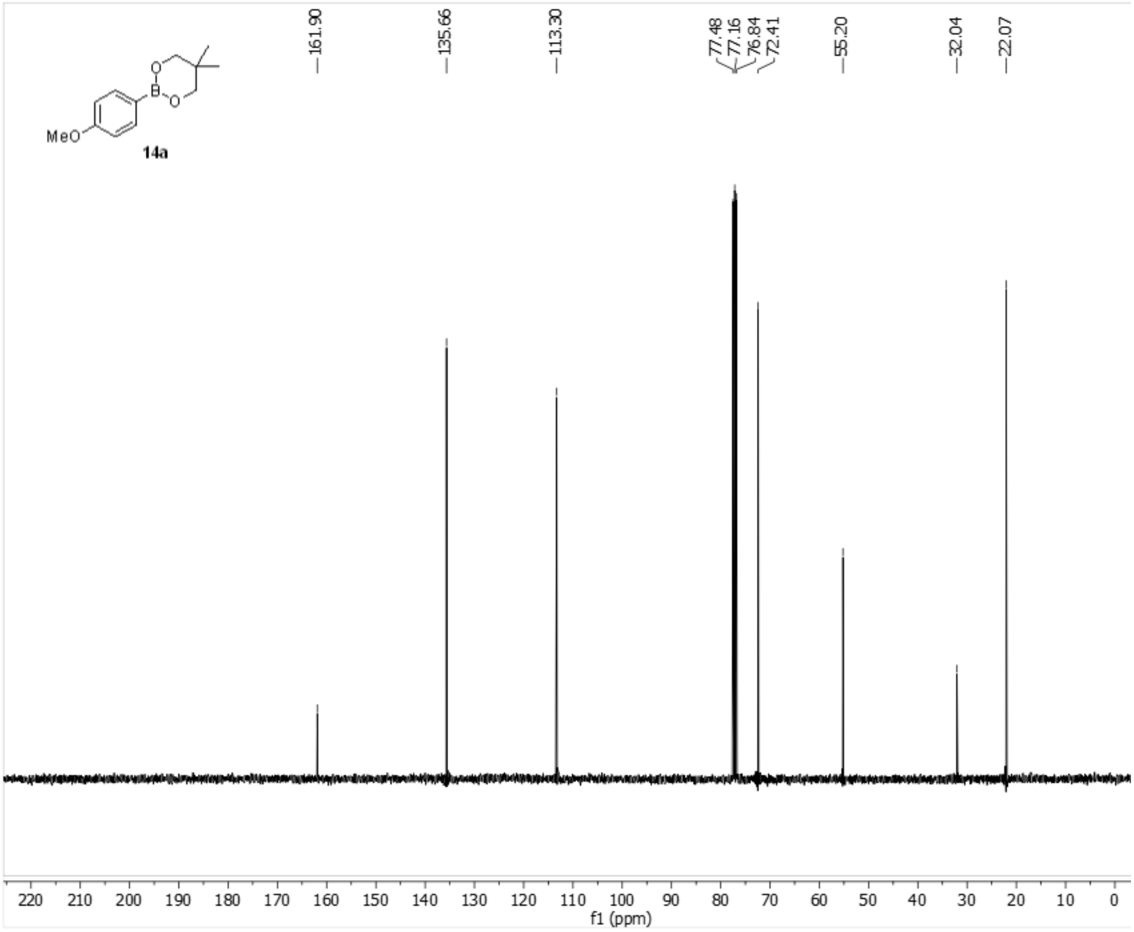

^{11}B NMR (128 MHz, $CDCl_3$)

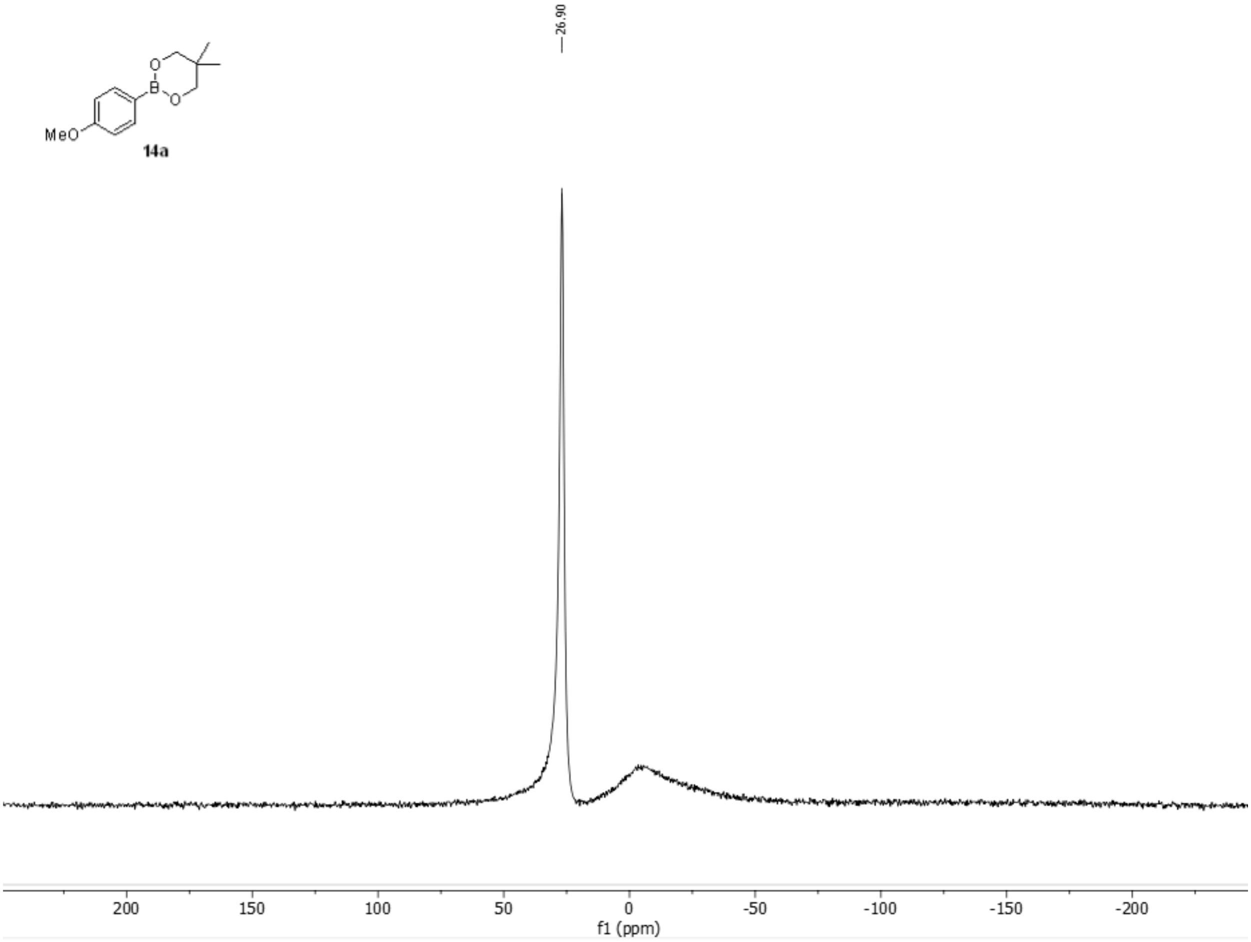

5,5-Dimethyl-2-(*p*-tolyl)-1,3,2-dioxaborinane 14b

^{1}H NMR (400 MHz, $CDCl_3$)

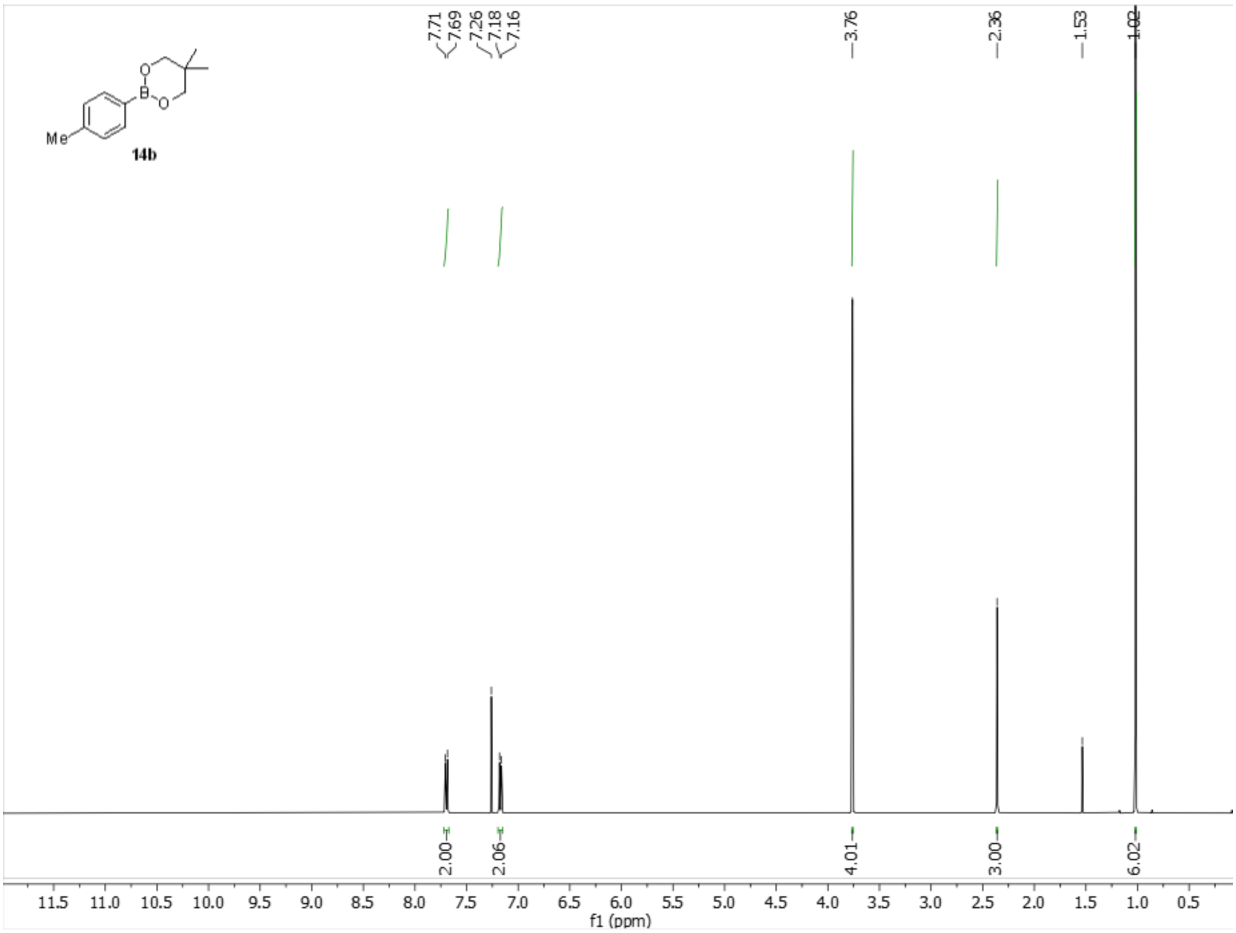

^{13}C NMR (101 MHz, $CDCl_3$)

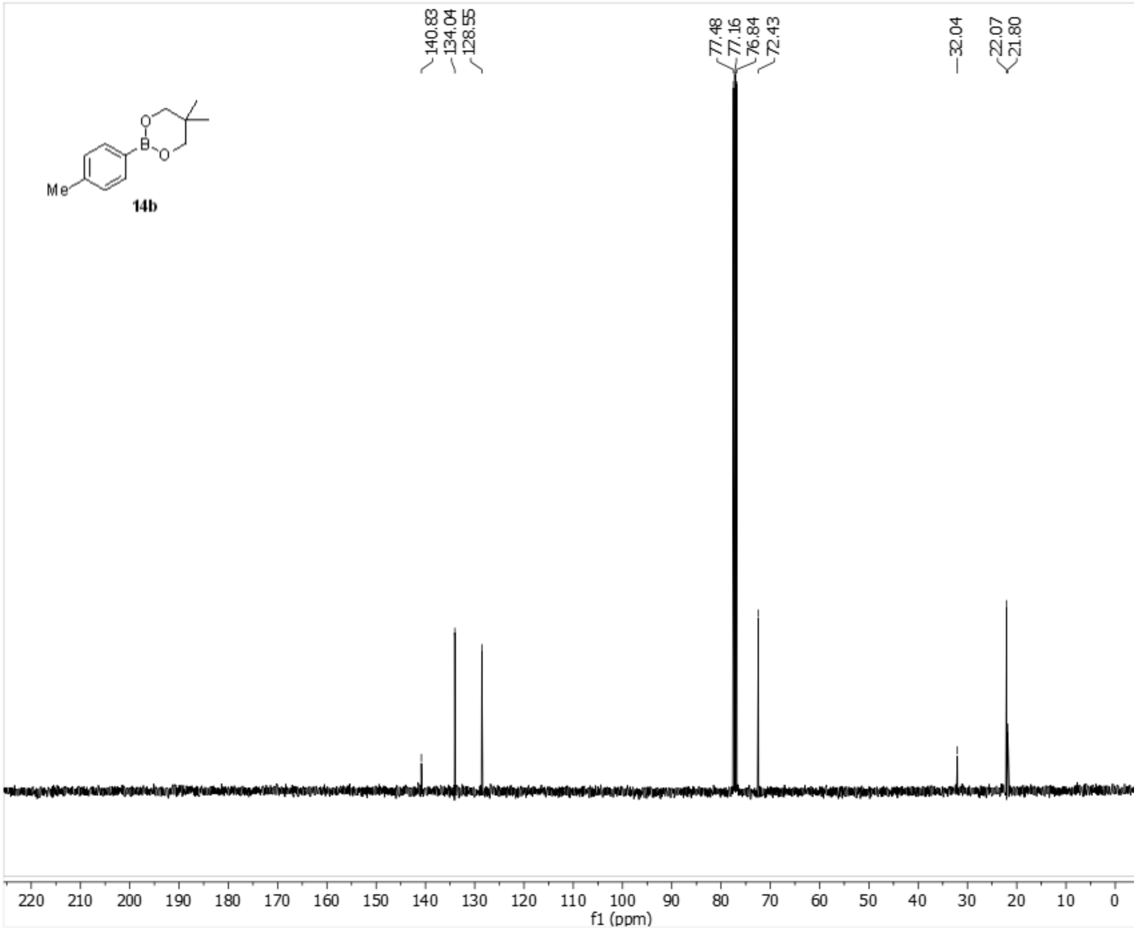

^{11}B NMR (128 MHz, $CDCl_3$)

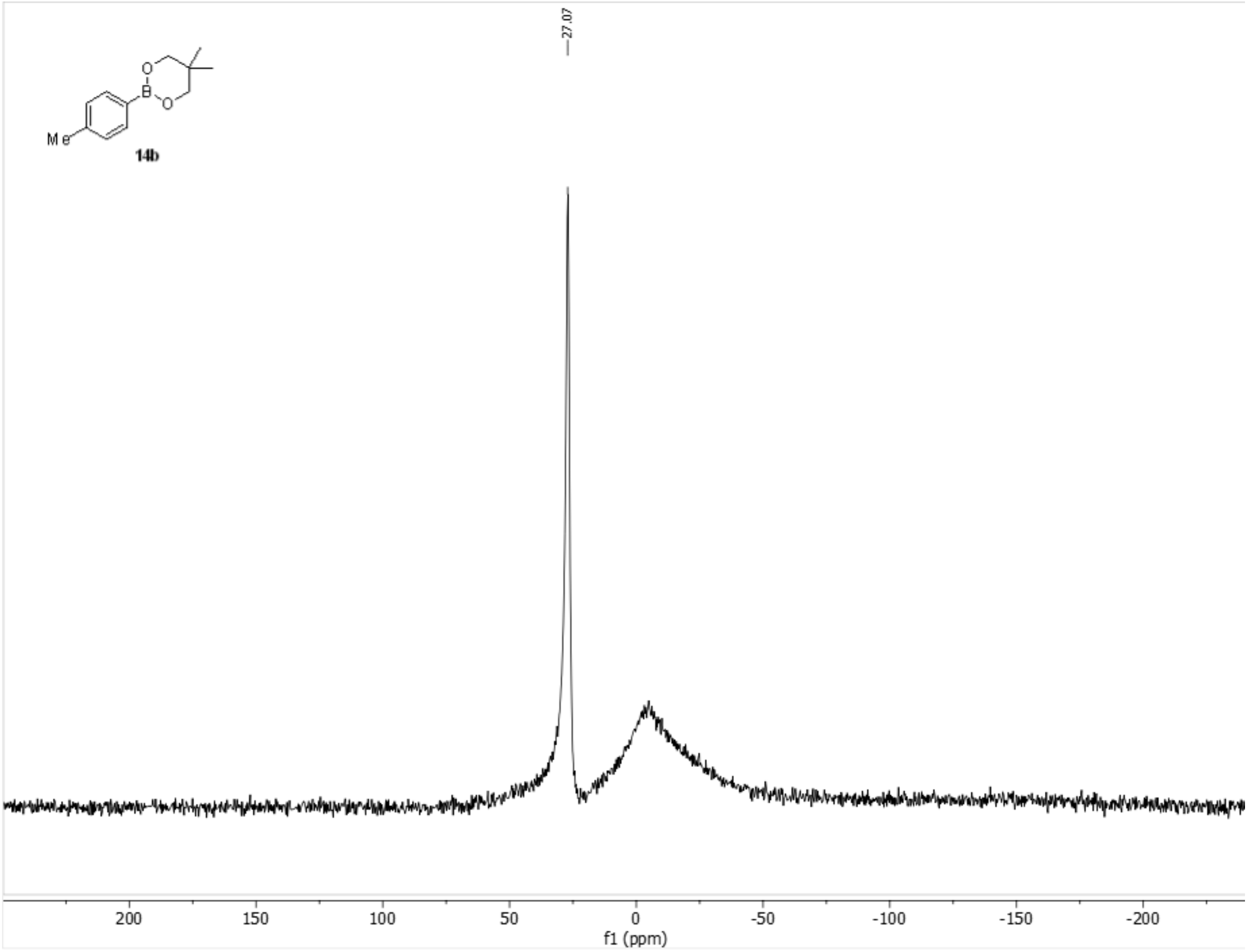

2-([1,1'-Biphenyl]-4-yl)-5,5-dimethyl-1,3,2-dioxaborinane 14c

^{1}H NMR (400 MHz, $CDCl_3$)

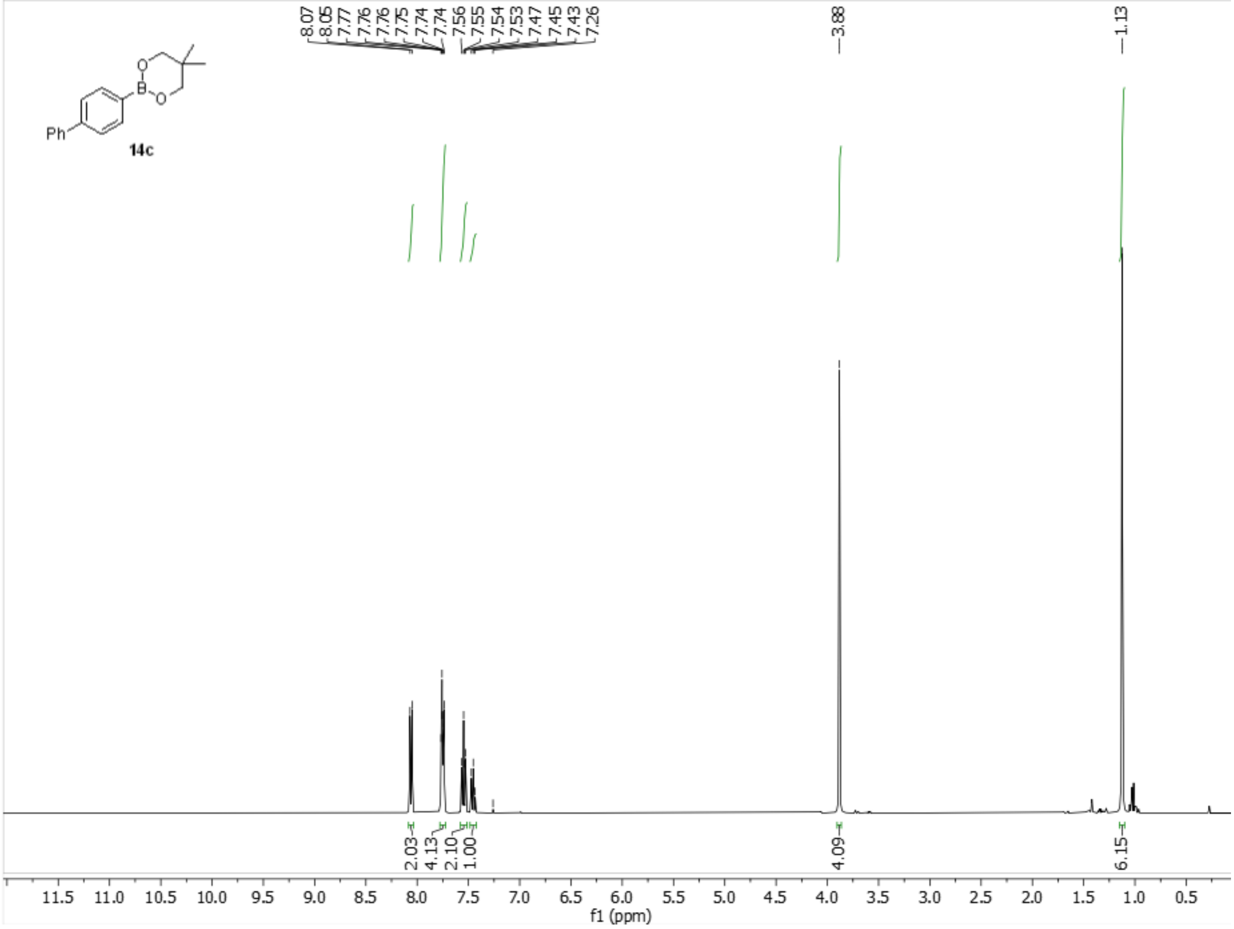

^{13}C NMR (101 MHz, $CDCl_3$)

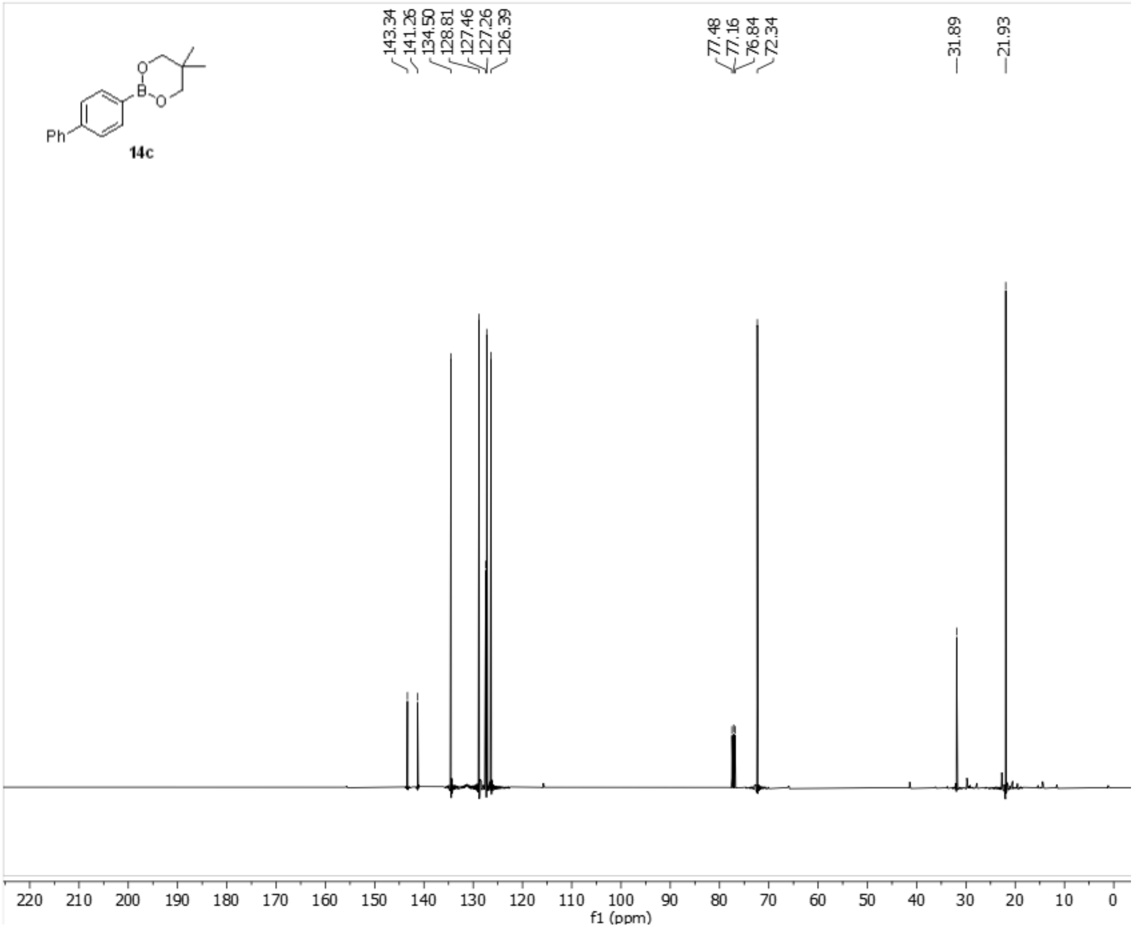

^{11}B NMR (128 MHz, $CDCl_3$)

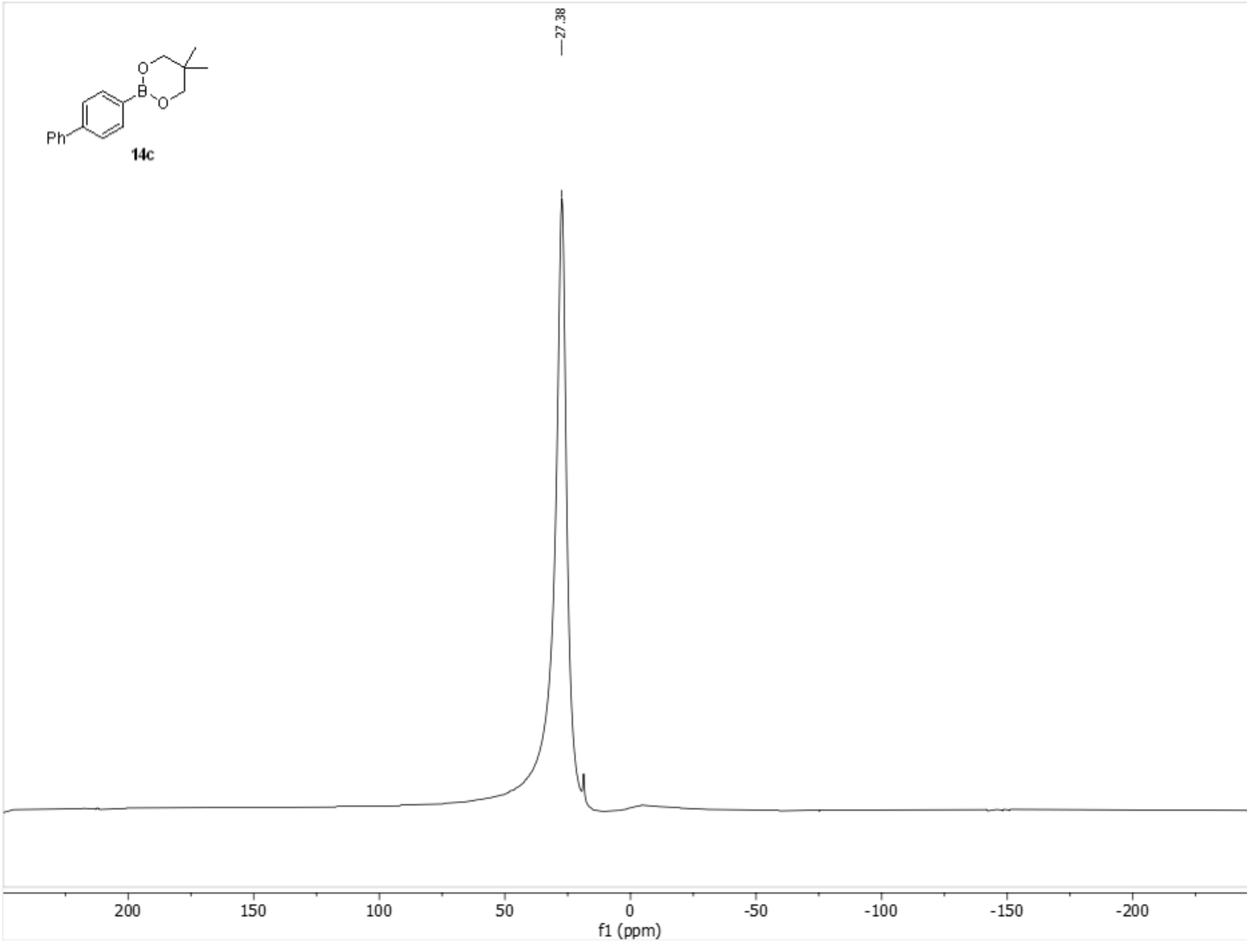

2-(4-(*Tert*-butyl)phenyl)-5,5-dimethyl-1,3,2-dioxaborinane 14d

^{1}H NMR (400 MHz, $CDCl_3$)

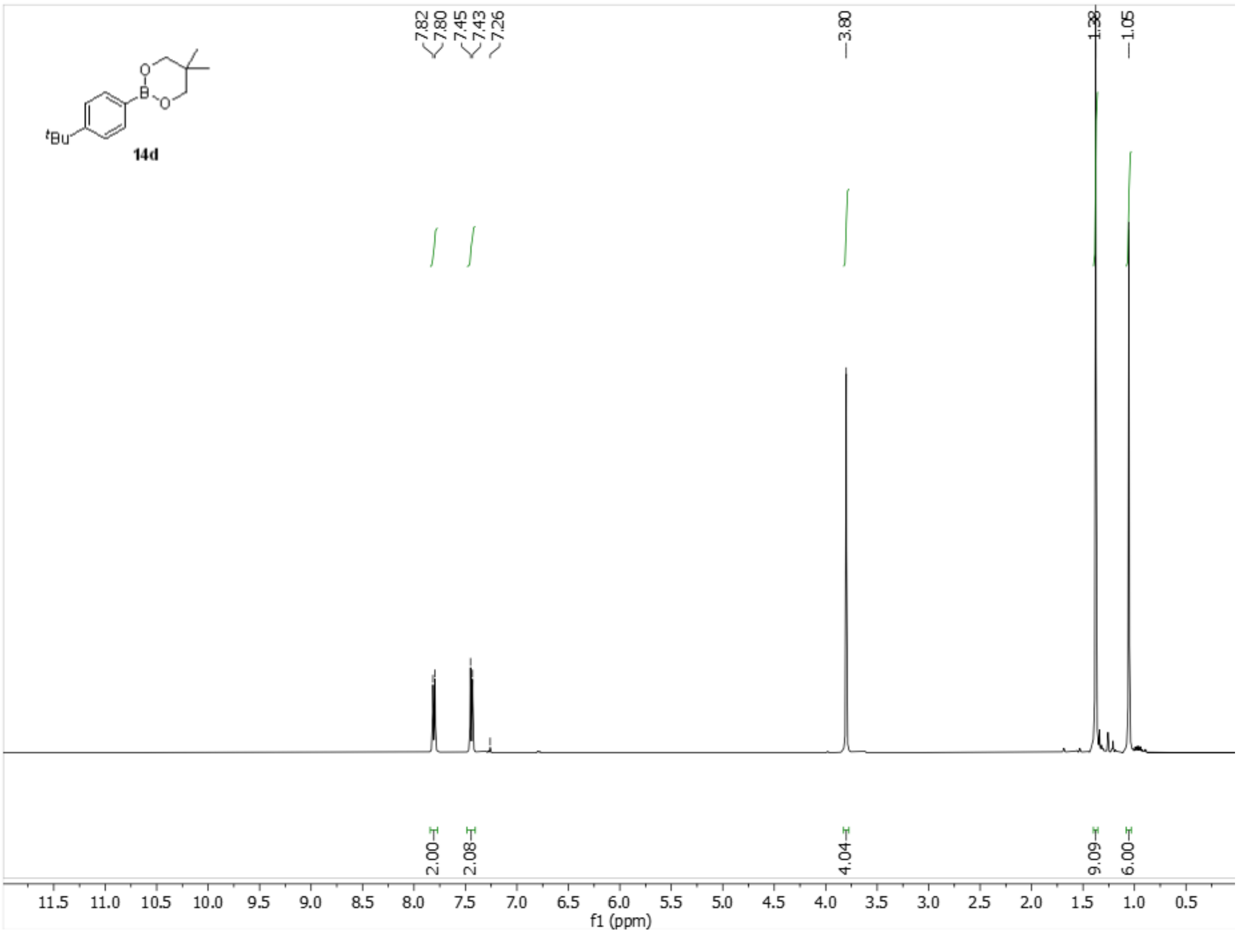

^{13}C NMR (101 MHz, $CDCl_3$)

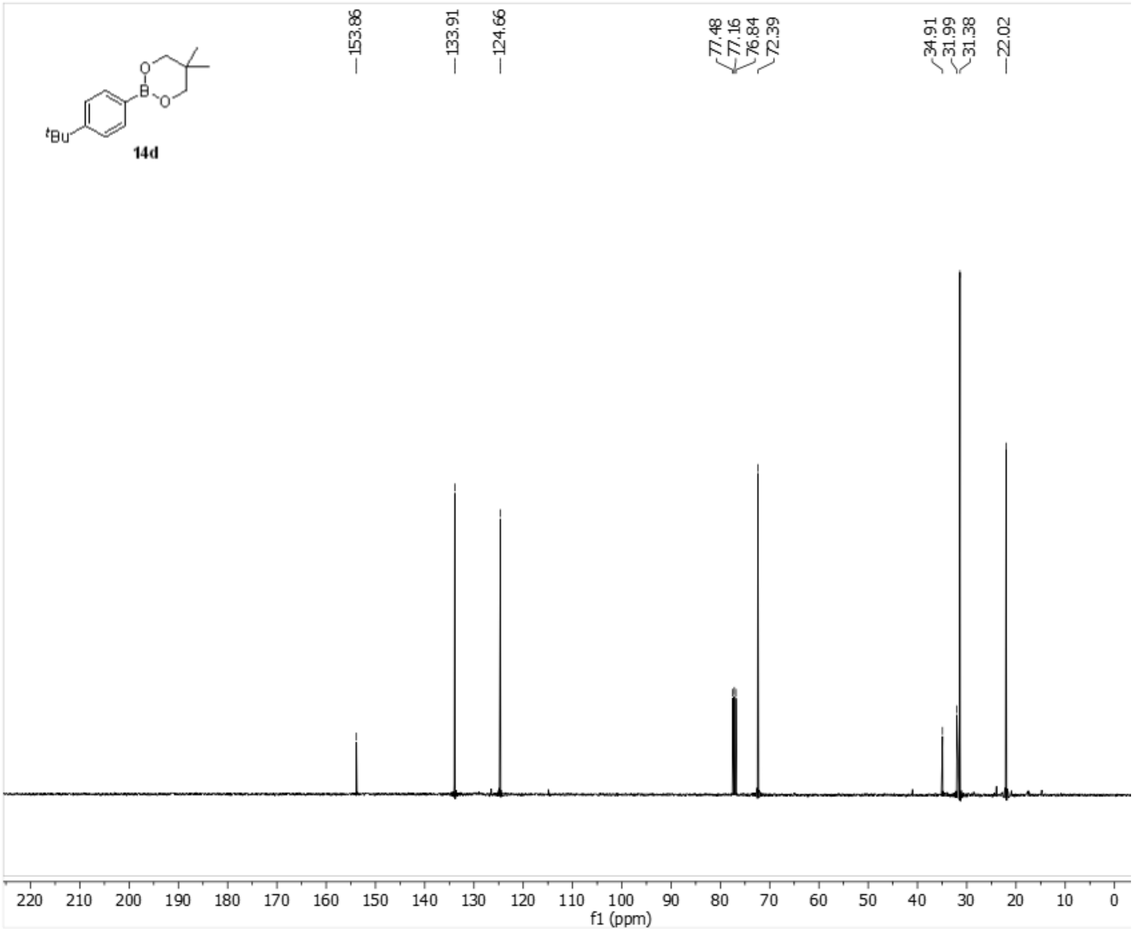

^{11}B NMR (128 MHz, $CDCl_3$)

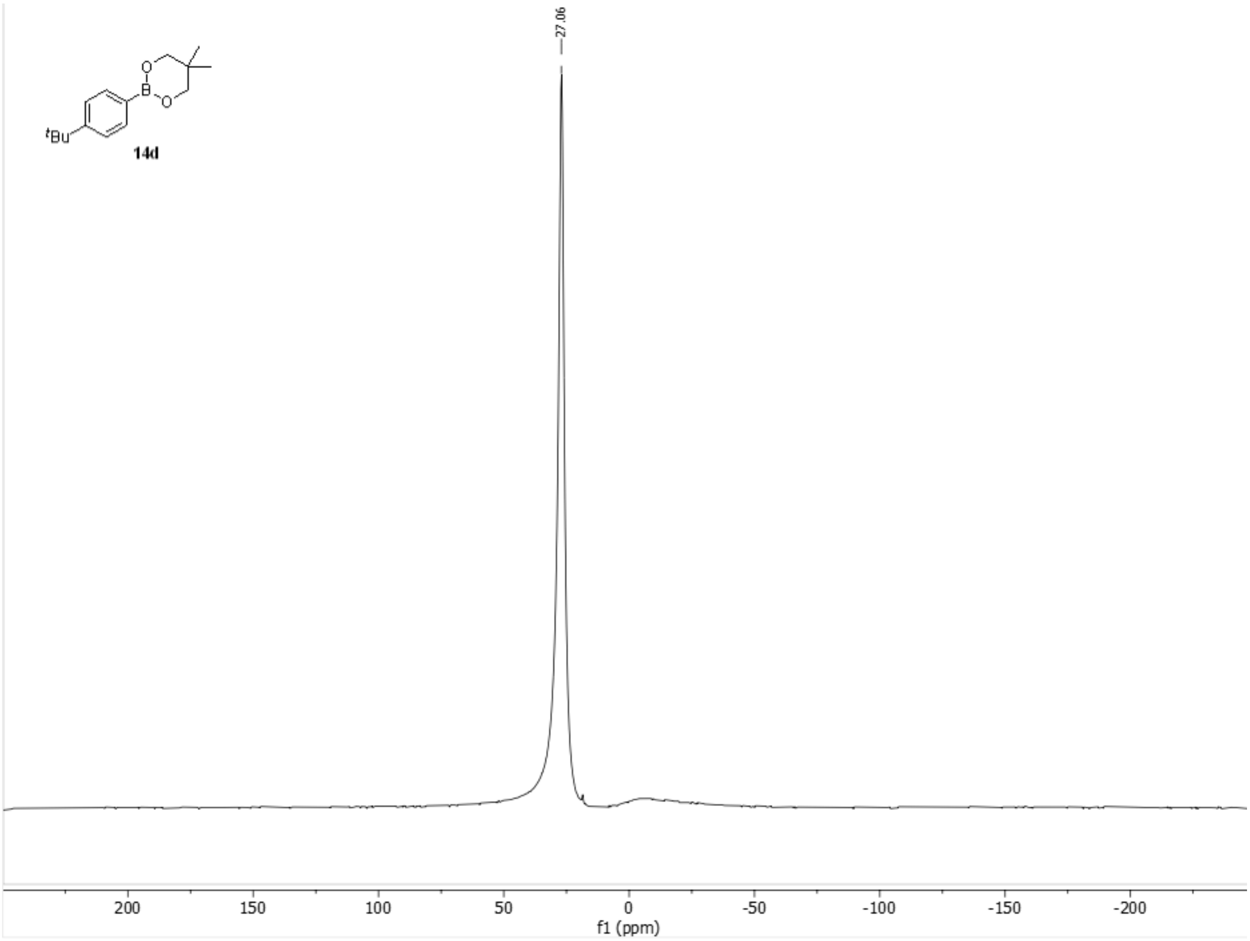

2-(4-Chlorophenyl)-5,5-dimethyl-1,3,2-dioxaborinane 14e

^{1}H NMR (400 MHz, $CDCl_3$)

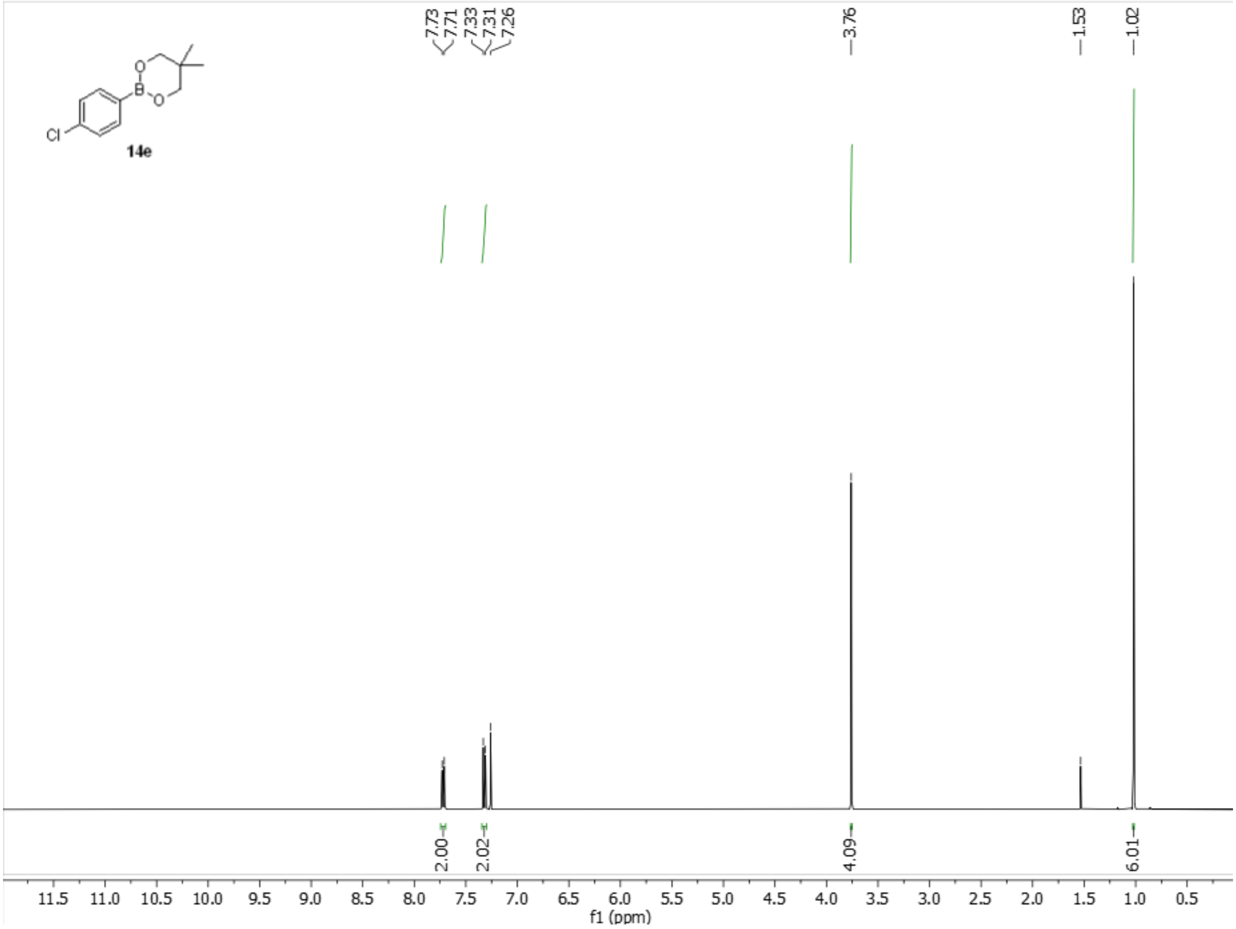

^{13}C NMR (101 MHz, $CDCl_3$)

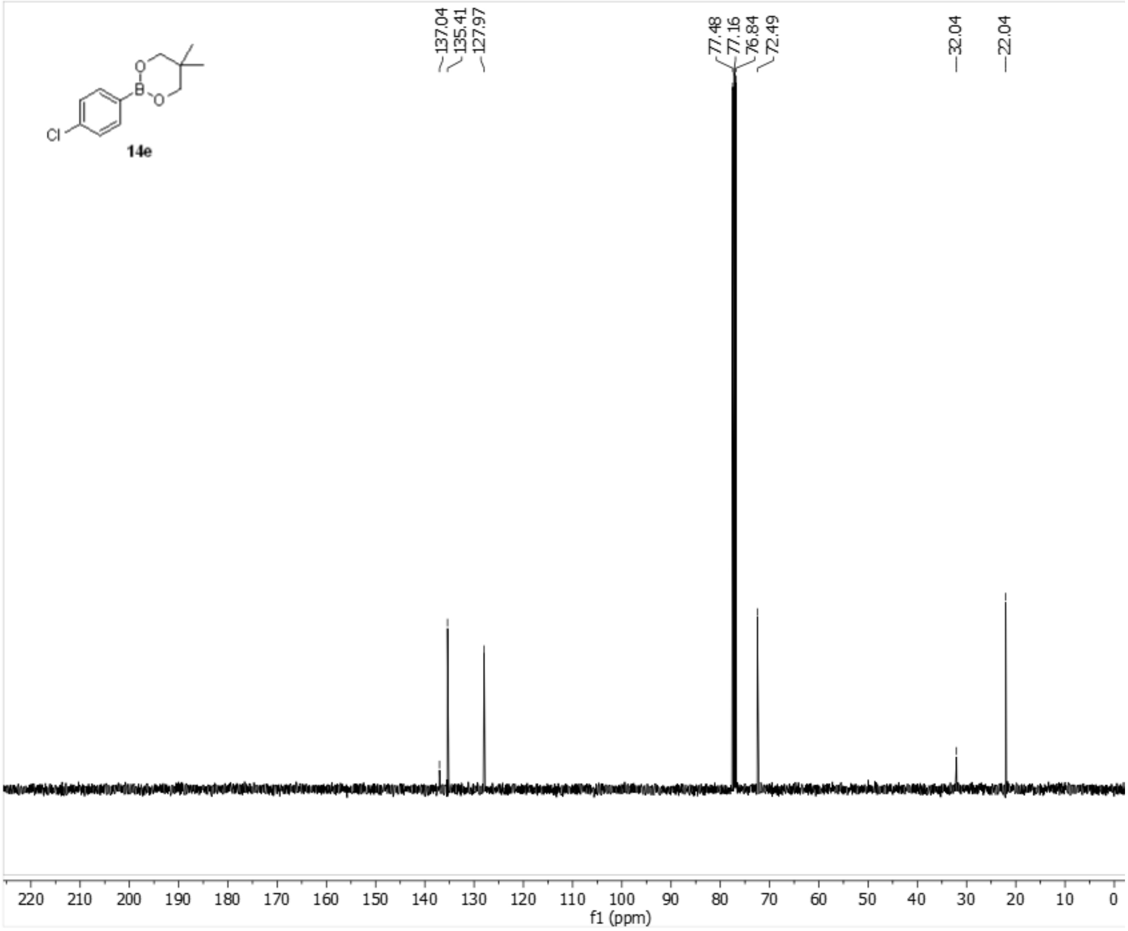

^{11}B NMR (128 MHz, $CDCl_3$)

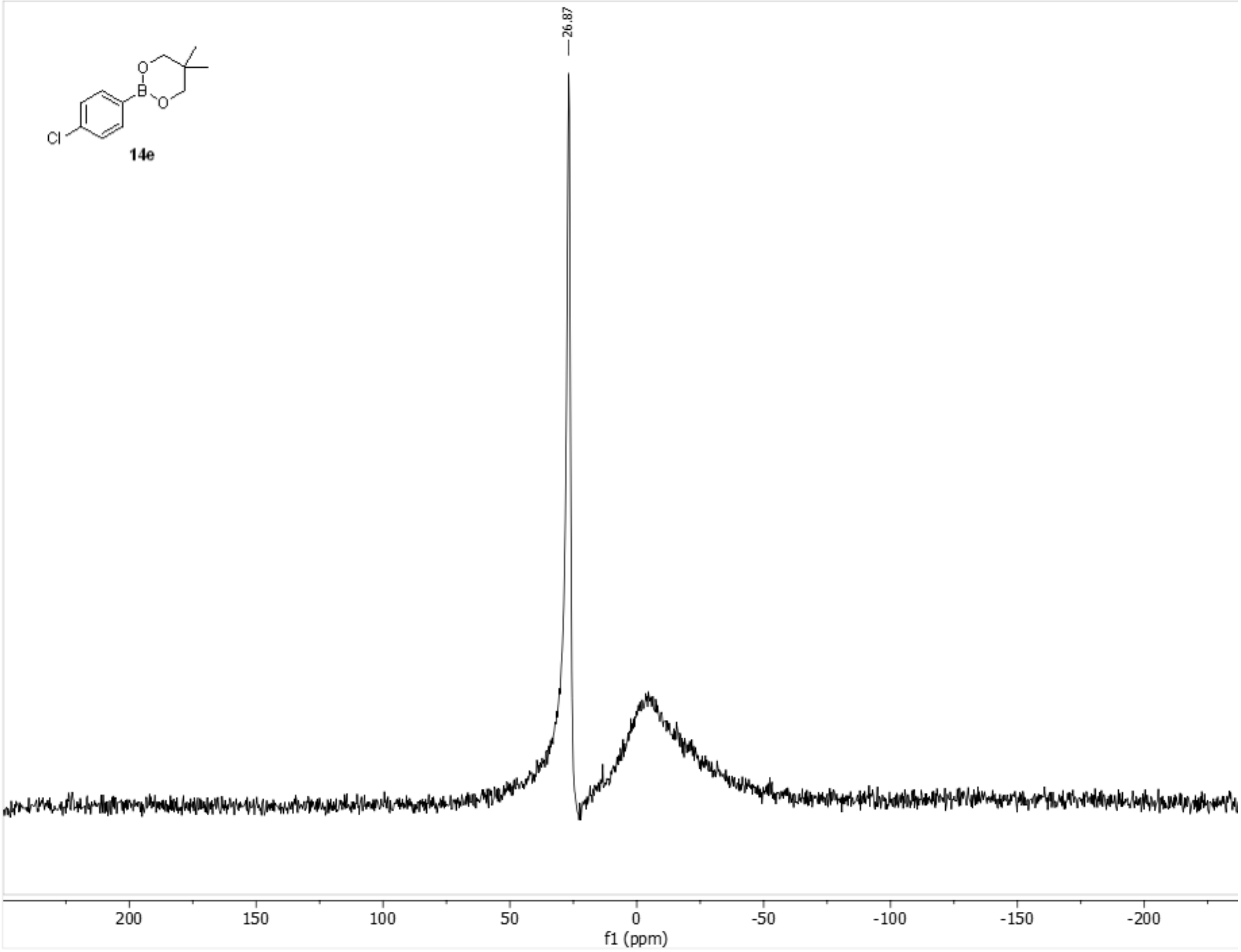

2-(4-Fluorophenyl)-5,5-dimethyl-1,3,2-dioxaborinane 14f

^{1}H NMR (400 MHz, $CDCl_3$)

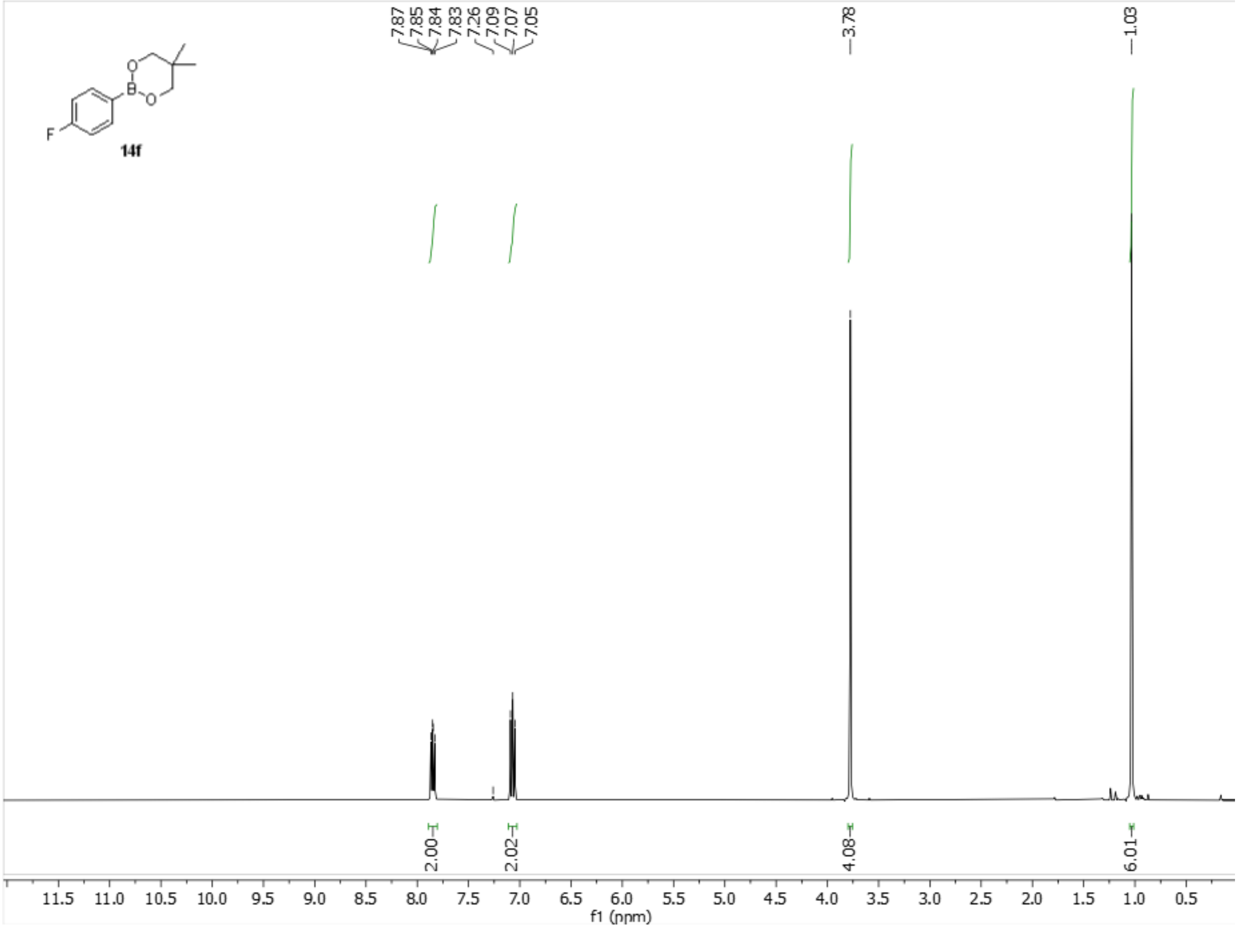

^{13}C NMR (101 MHz, $CDCl_3$)

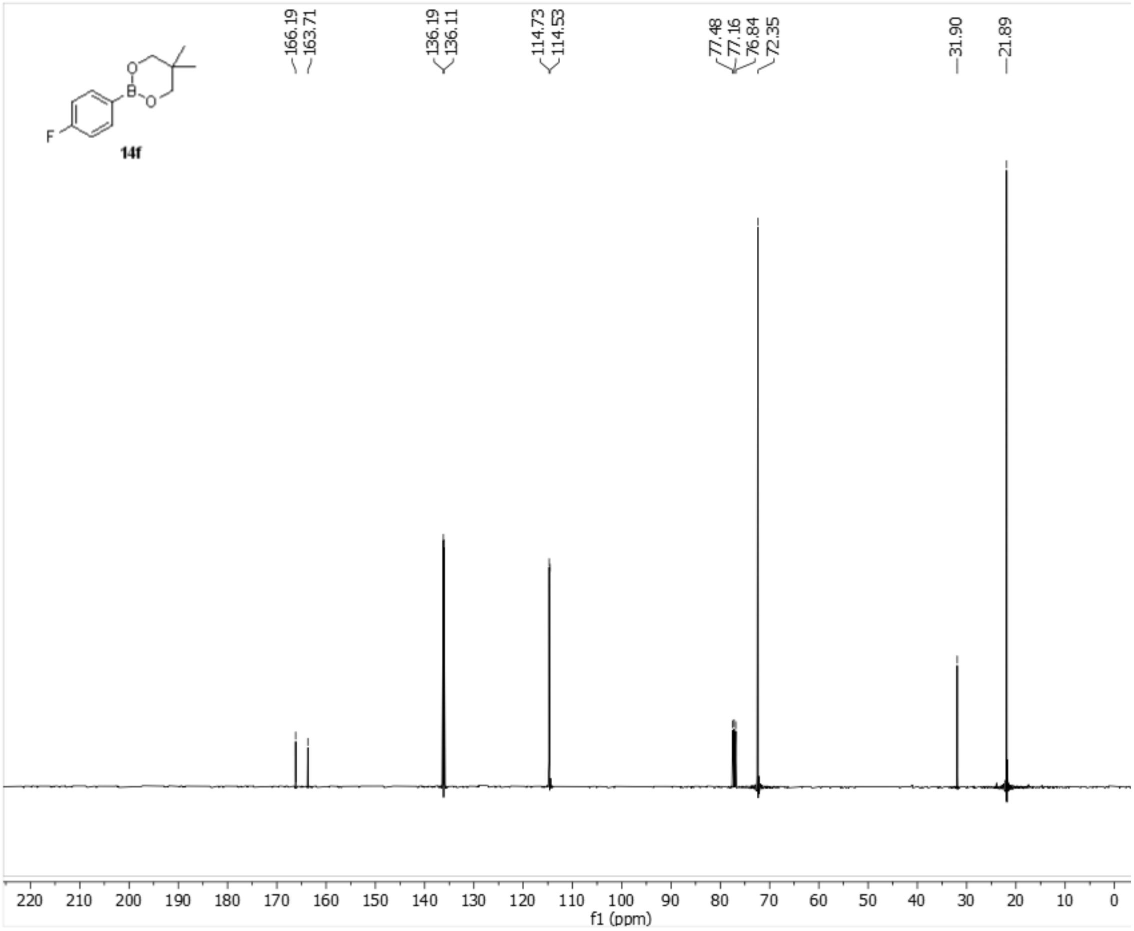

^{11}B NMR (128 MHz, $CDCl_3$)

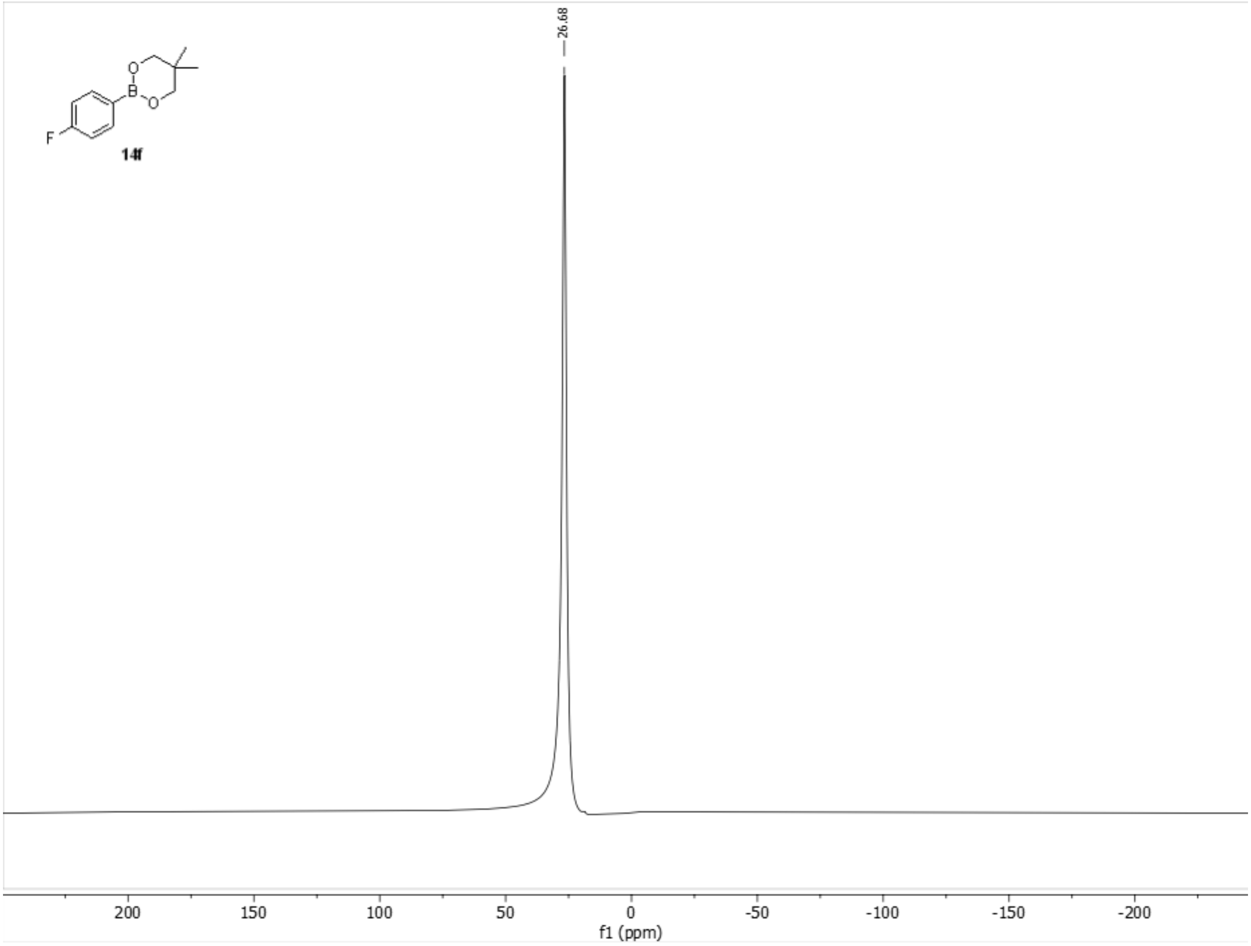

^{19}F NMR (376 MHz, $CDCl_3$)

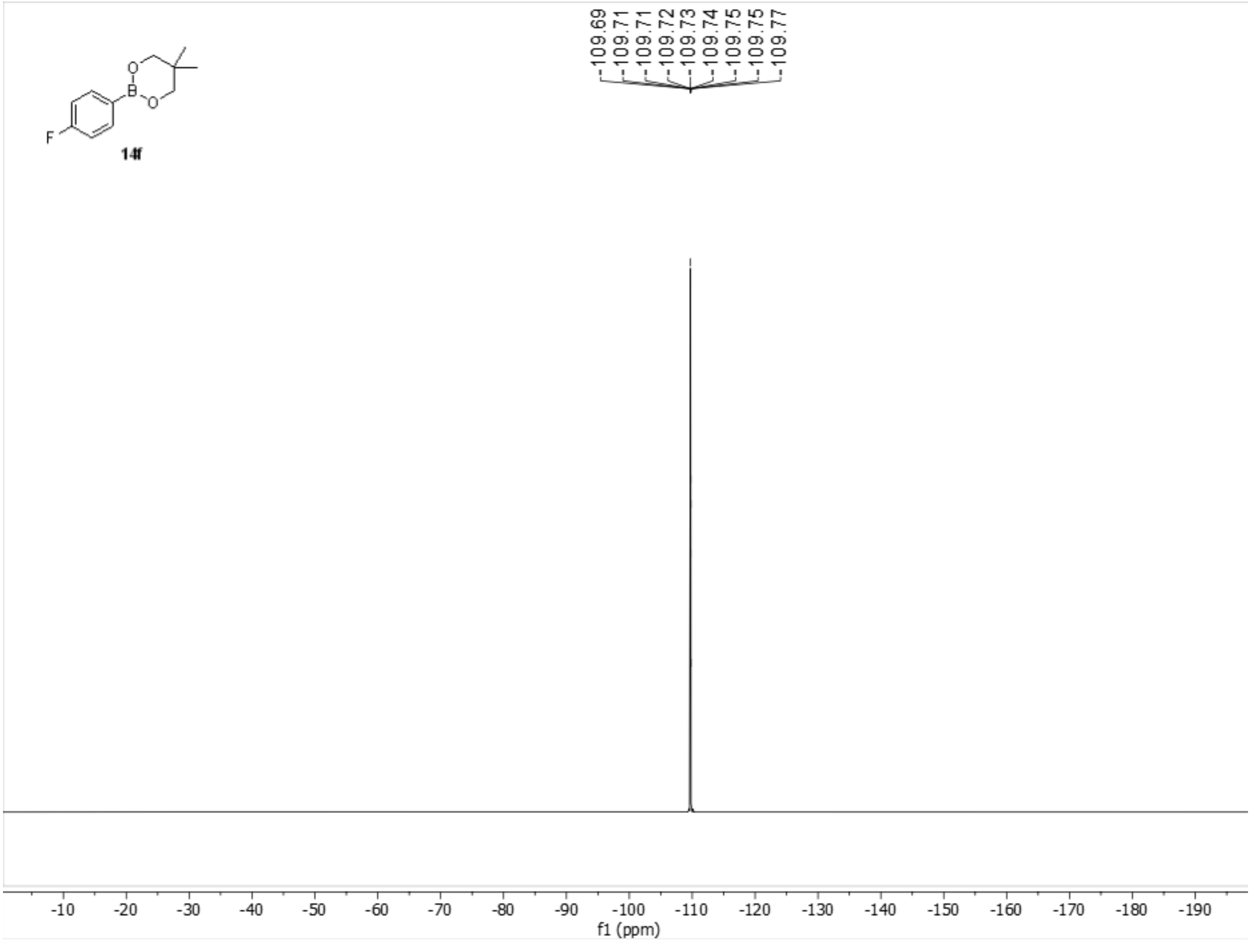

^{19}F NMR (376 MHz, $CDCl_3$) - expanded

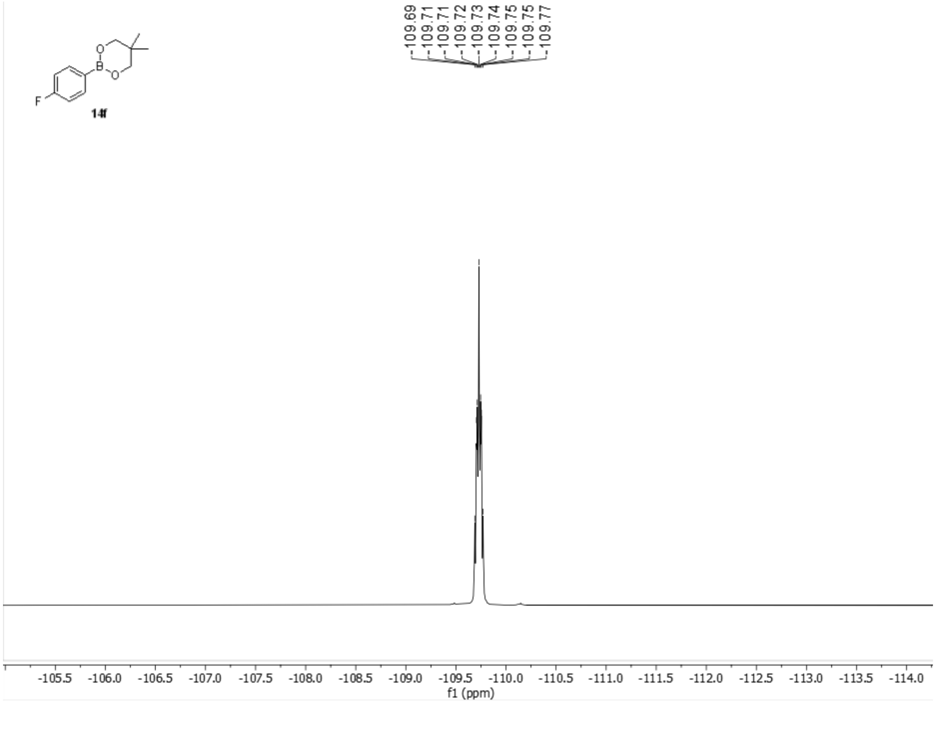

2-(4-Bromophenyl)-5,5-dimethyl-1,3,2-dioxaborinane 14g

^{1}H NMR (400 MHz, $CDCl_3$)

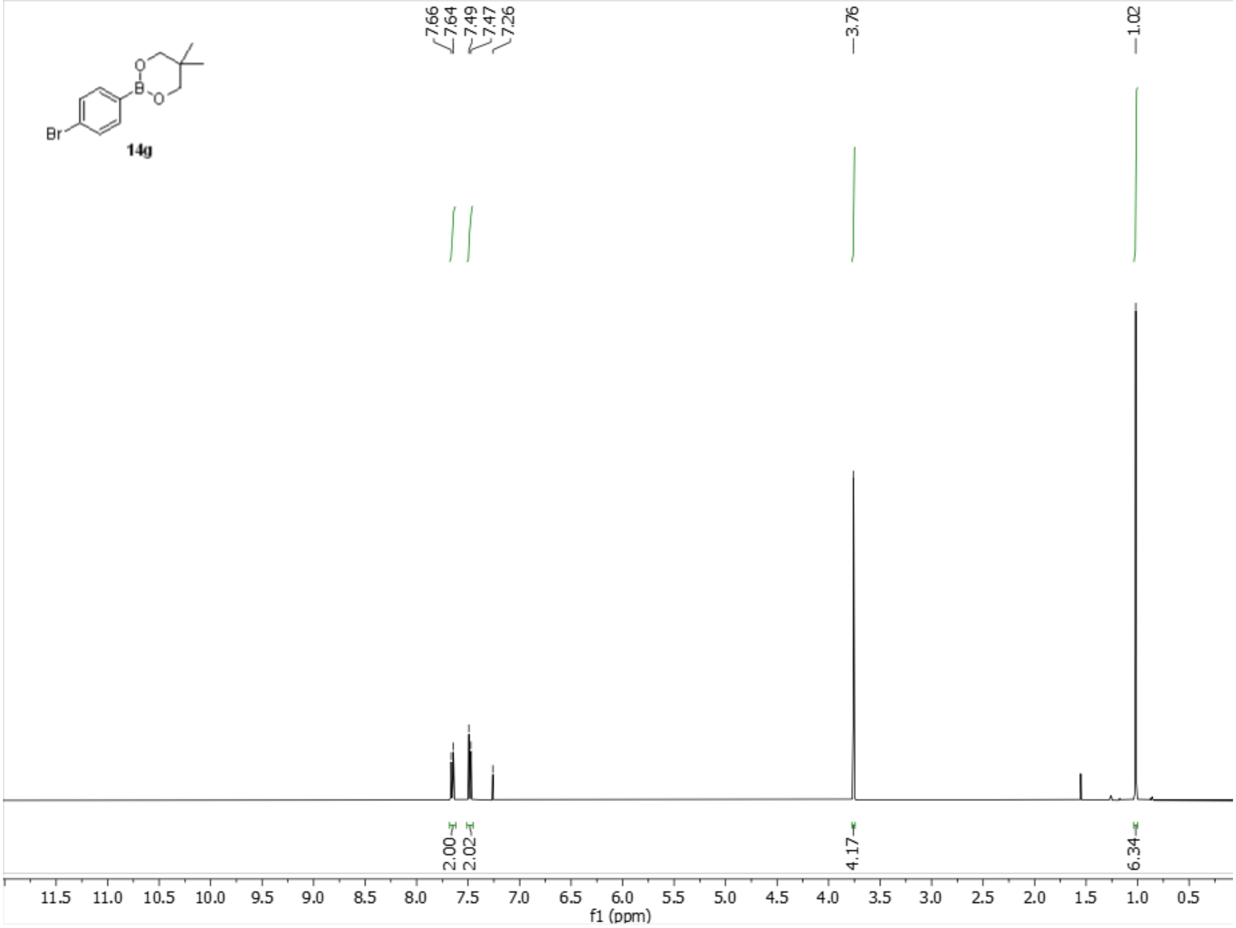

^{13}C NMR (101 MHz, $CDCl_3$

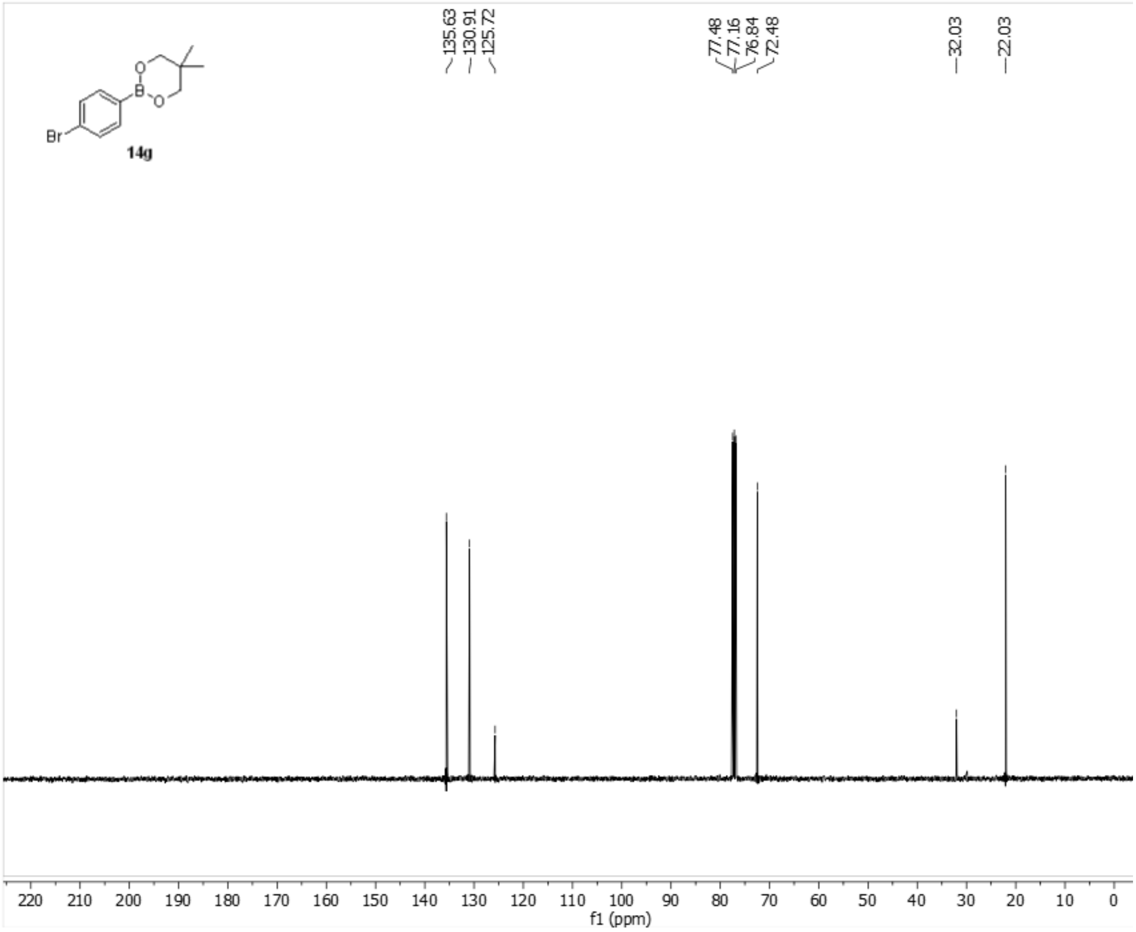

^{11}B NMR (128 MHz, $CDCl_3$)

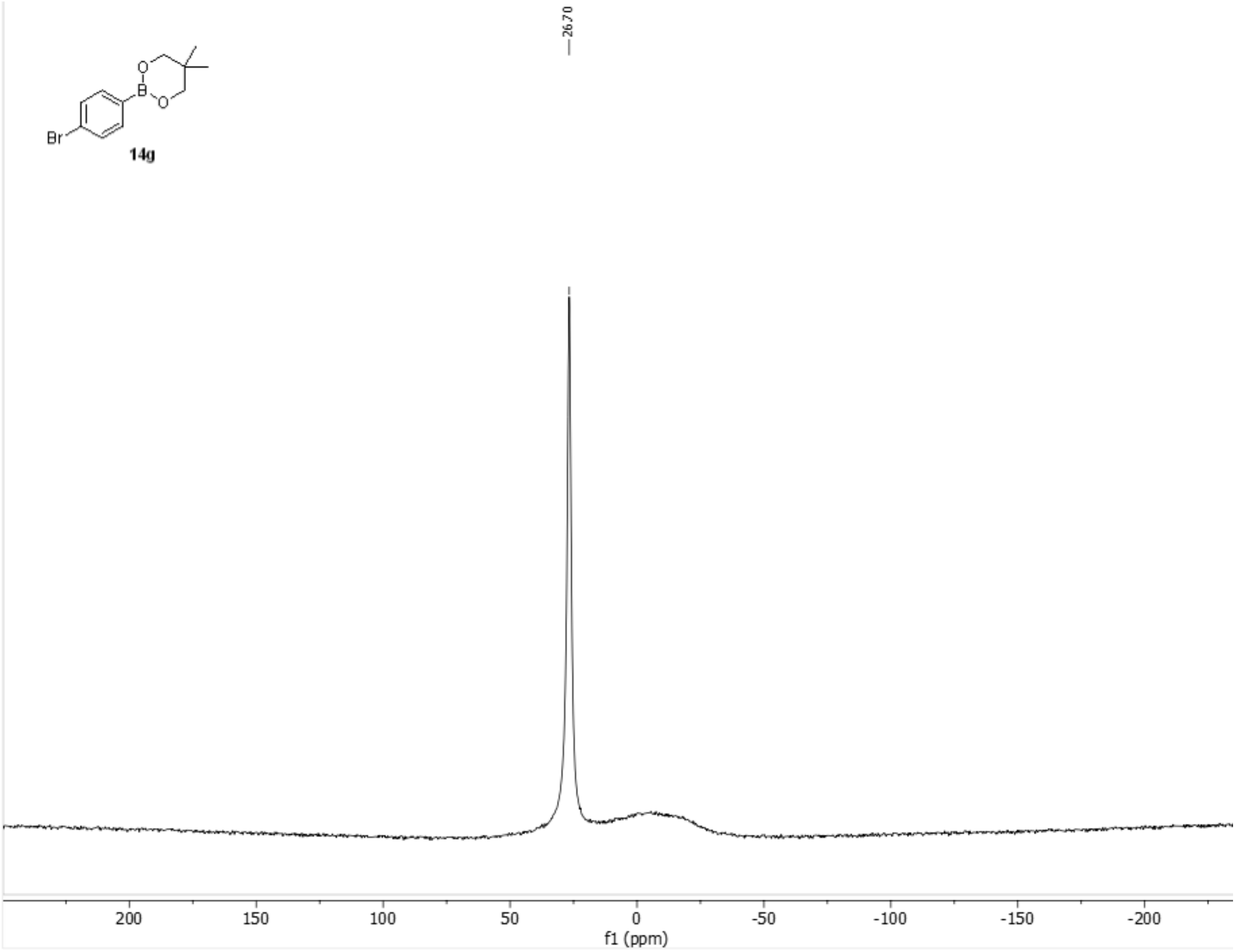

5,5-Dimethyl-2-(4-(trifluoromethyl)phenyl)-1,3,2-dioxaborinane 14h

^1H NMR (400 MHz, $CDCl_3$)

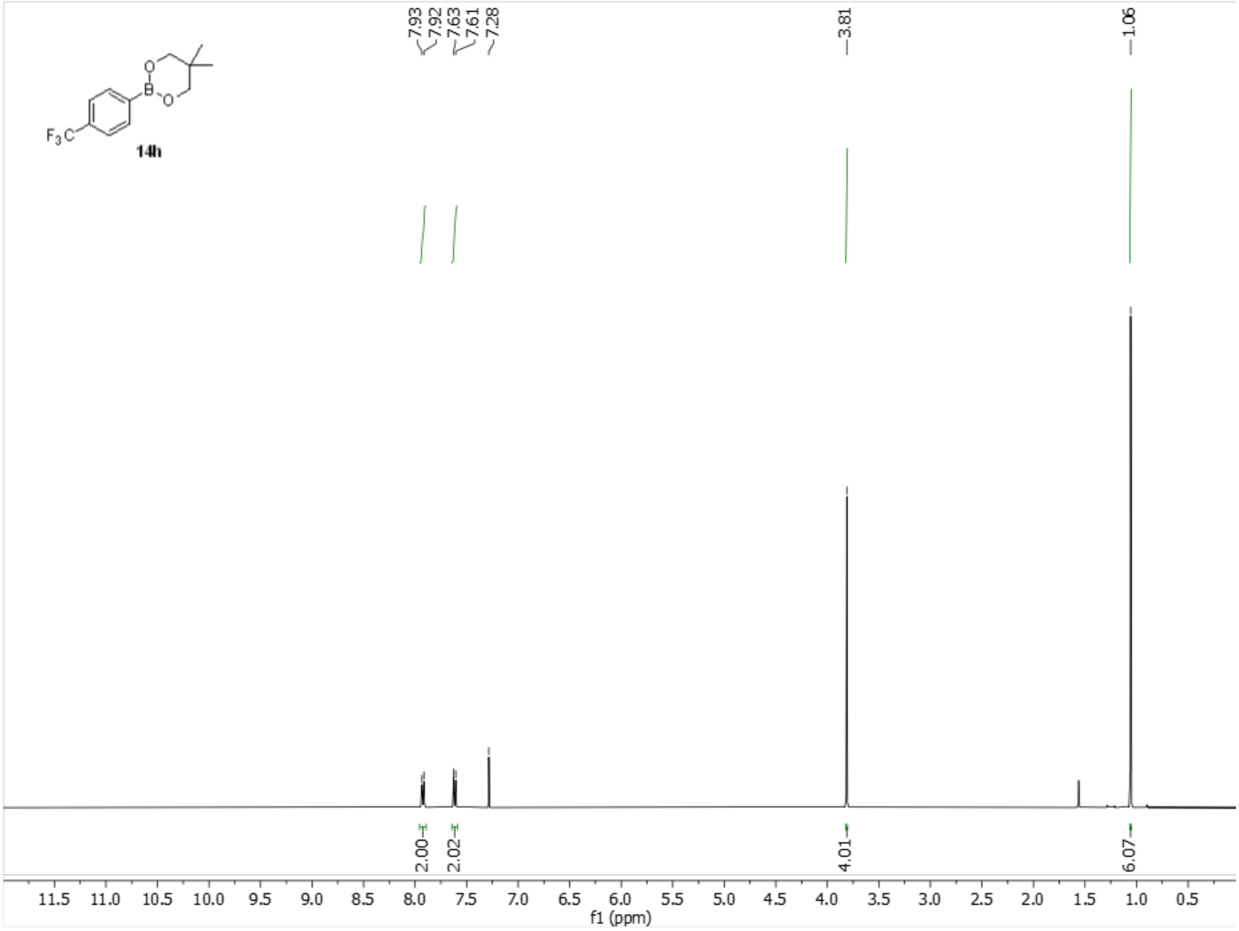

^{13}C NMR (101 MHz, $CDCl_3$)

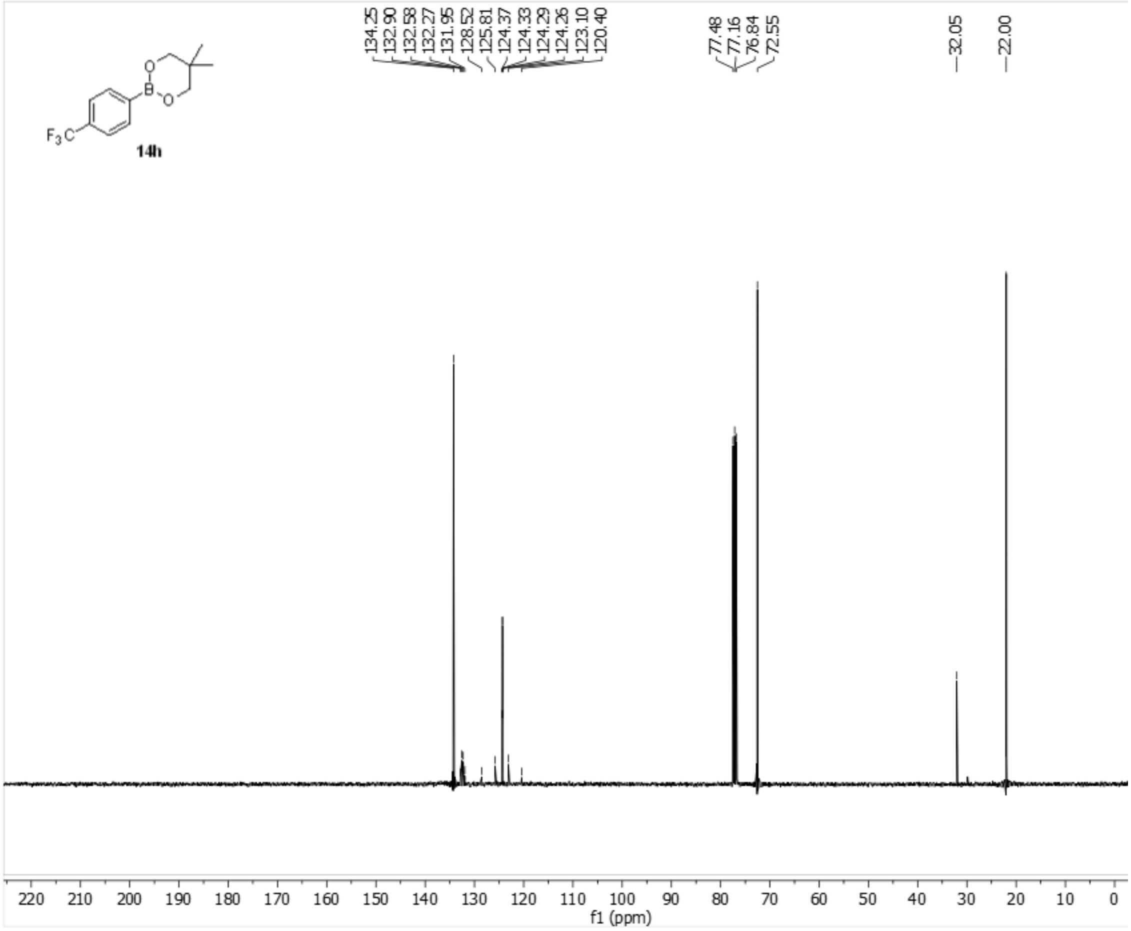

^{11}B NMR (128 MHz, $CDCl_3$)

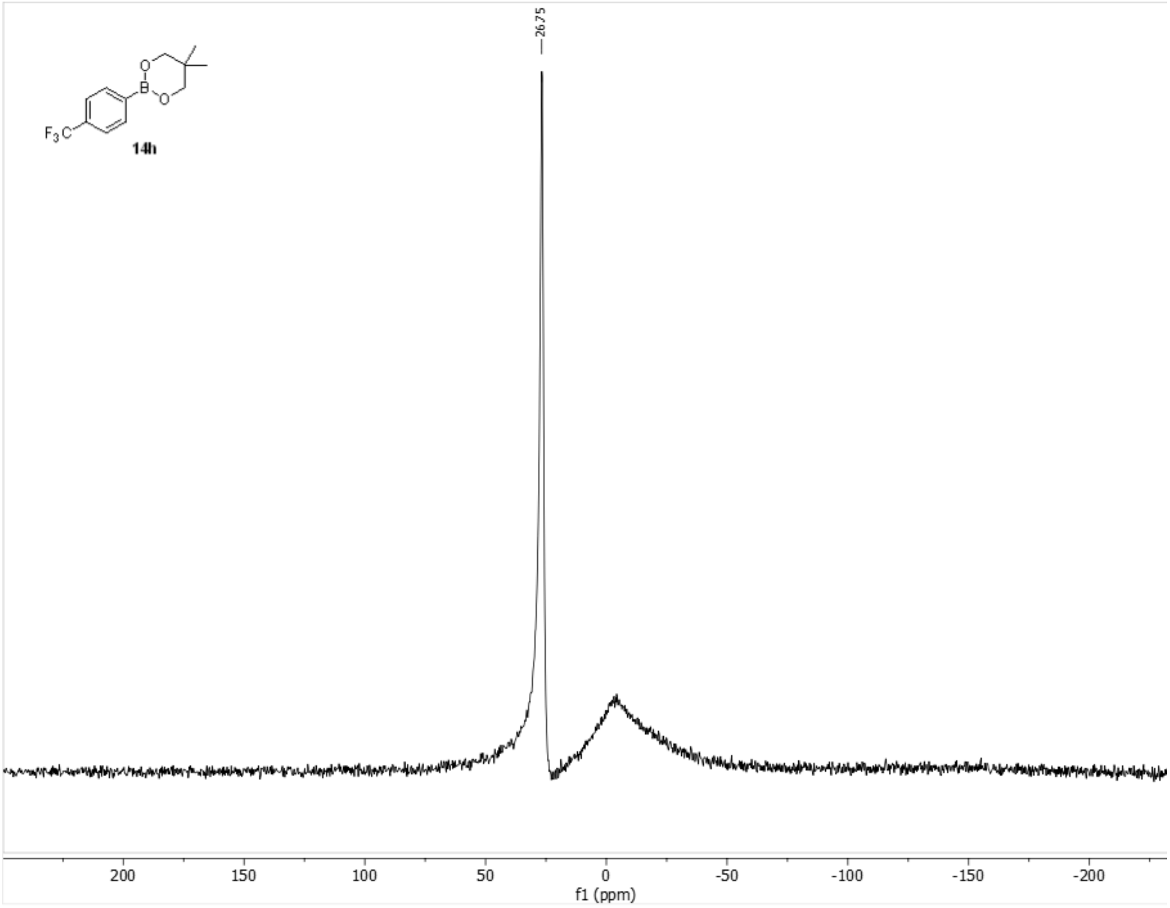

^{19}F NMR (376 MHz, $CDCl_3$)

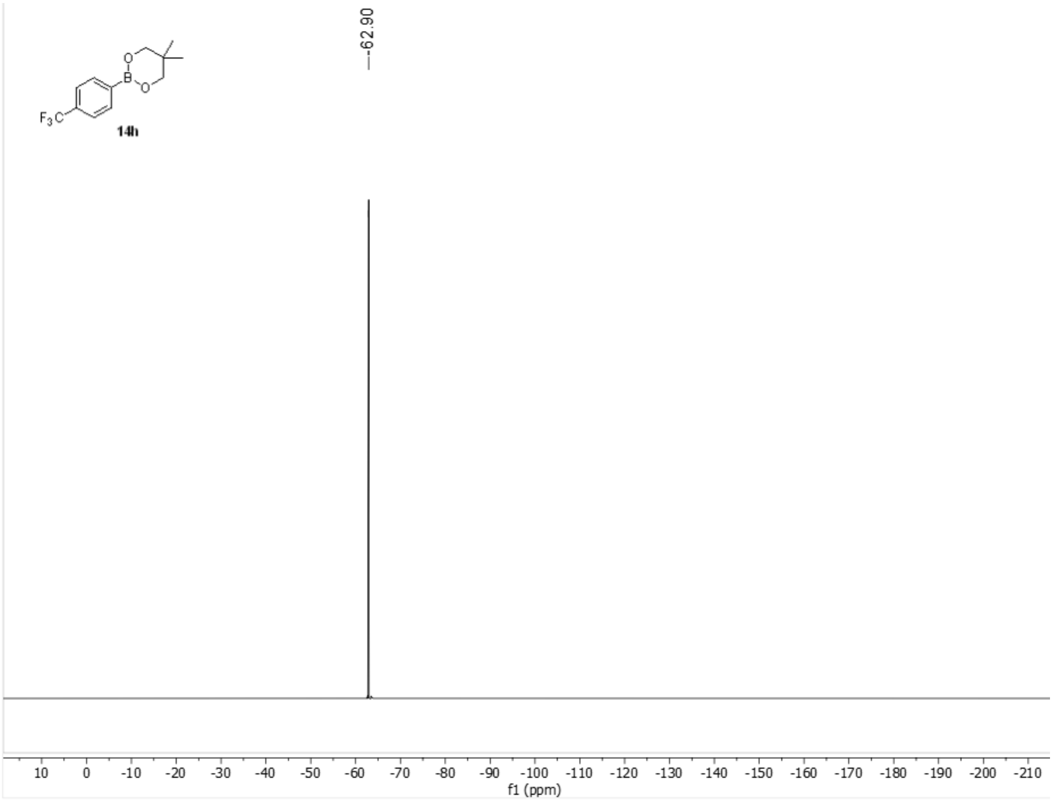

2-(4-(Benzyloxy)phenyl)-5,5-dimethyl-1,3,2-dioxaborinane 14i

^{1}H NMR (400 MHz, $CDCl_3$)

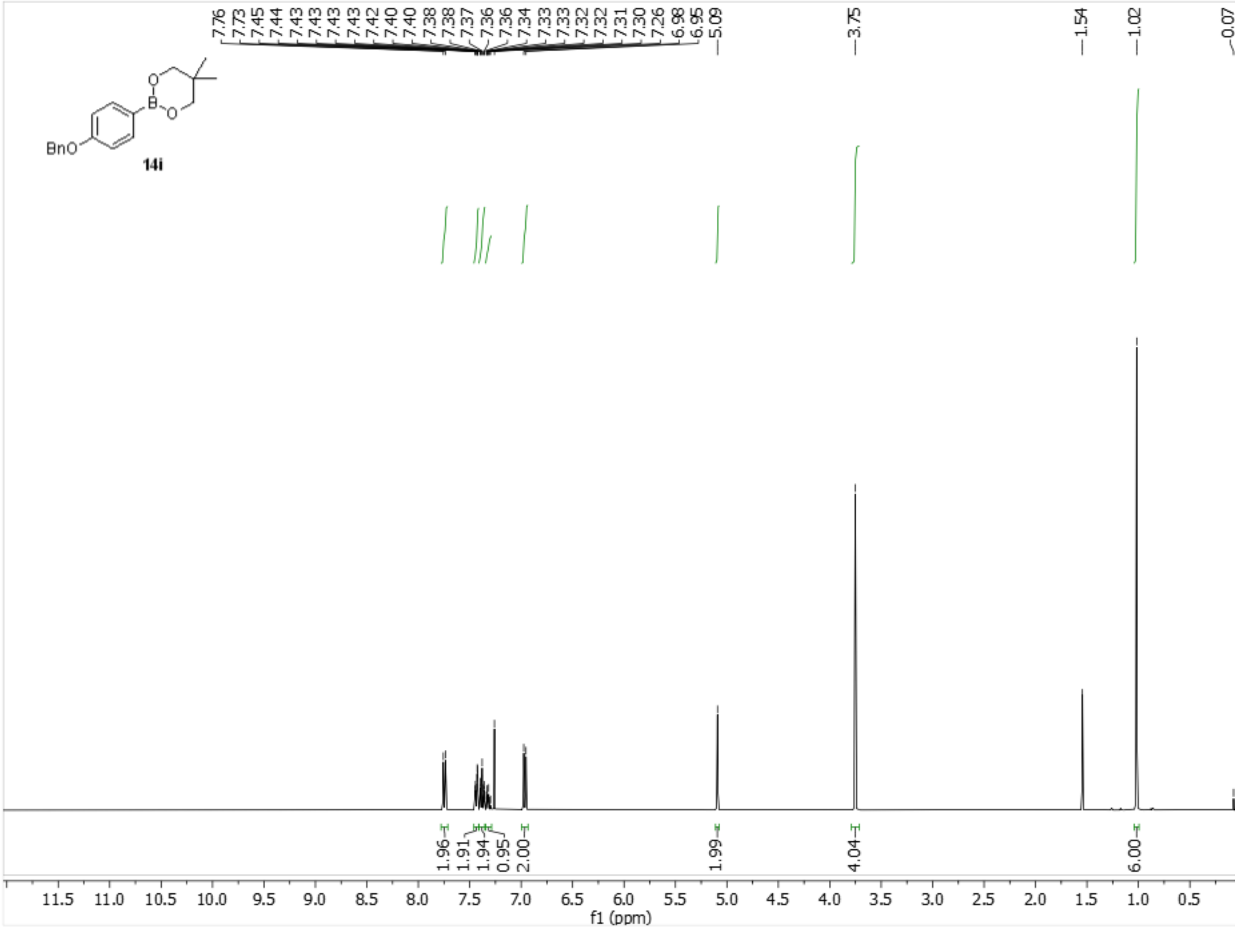

^{13}C NMR (101 MHz, $CDCl_3$)

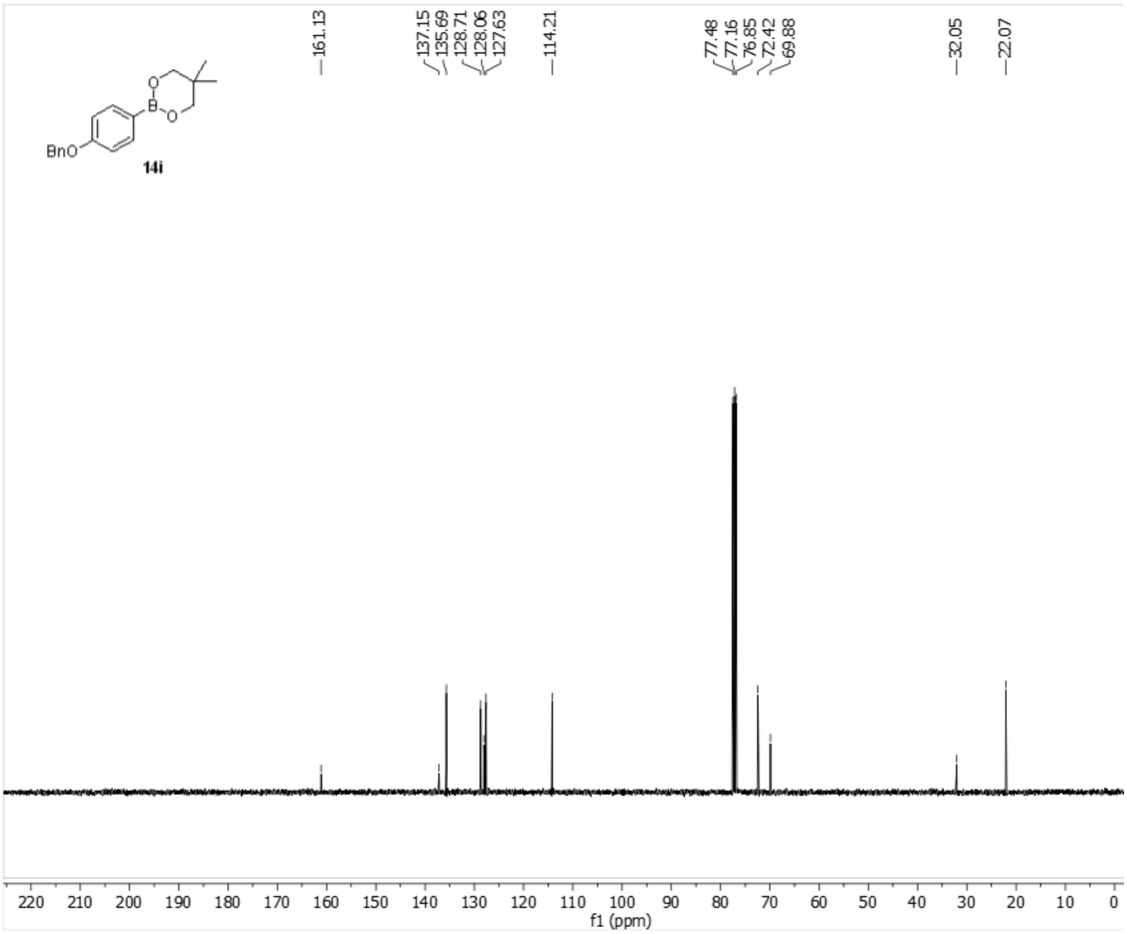

^{11}B NMR (128 MHz, $CDCl_3$)

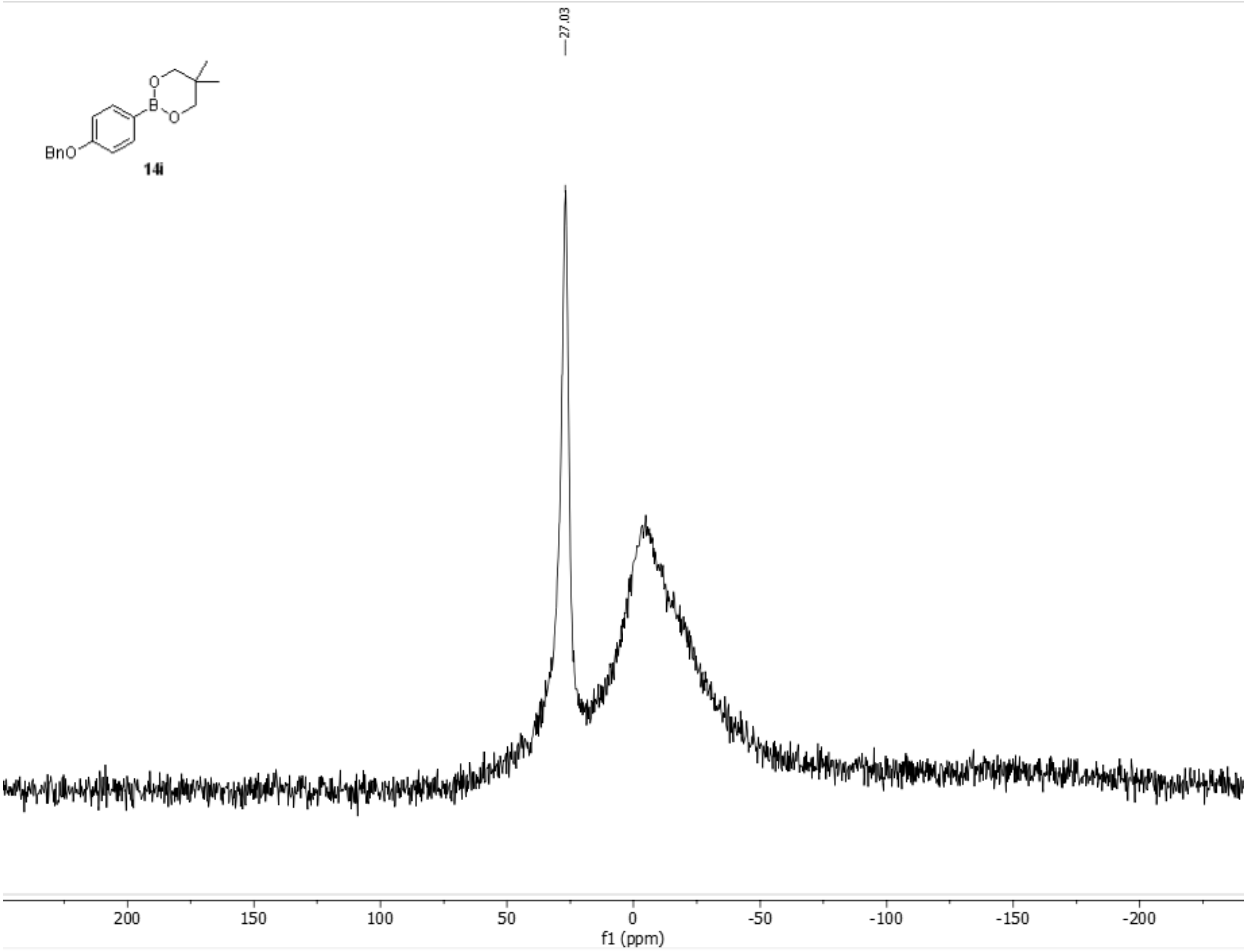

5,5-Dimethyl-2-(*o*-tolyl)-1,3,2-dioxaborinane 14j

^{1}H NMR (400 MHz, $CDCl_3$)

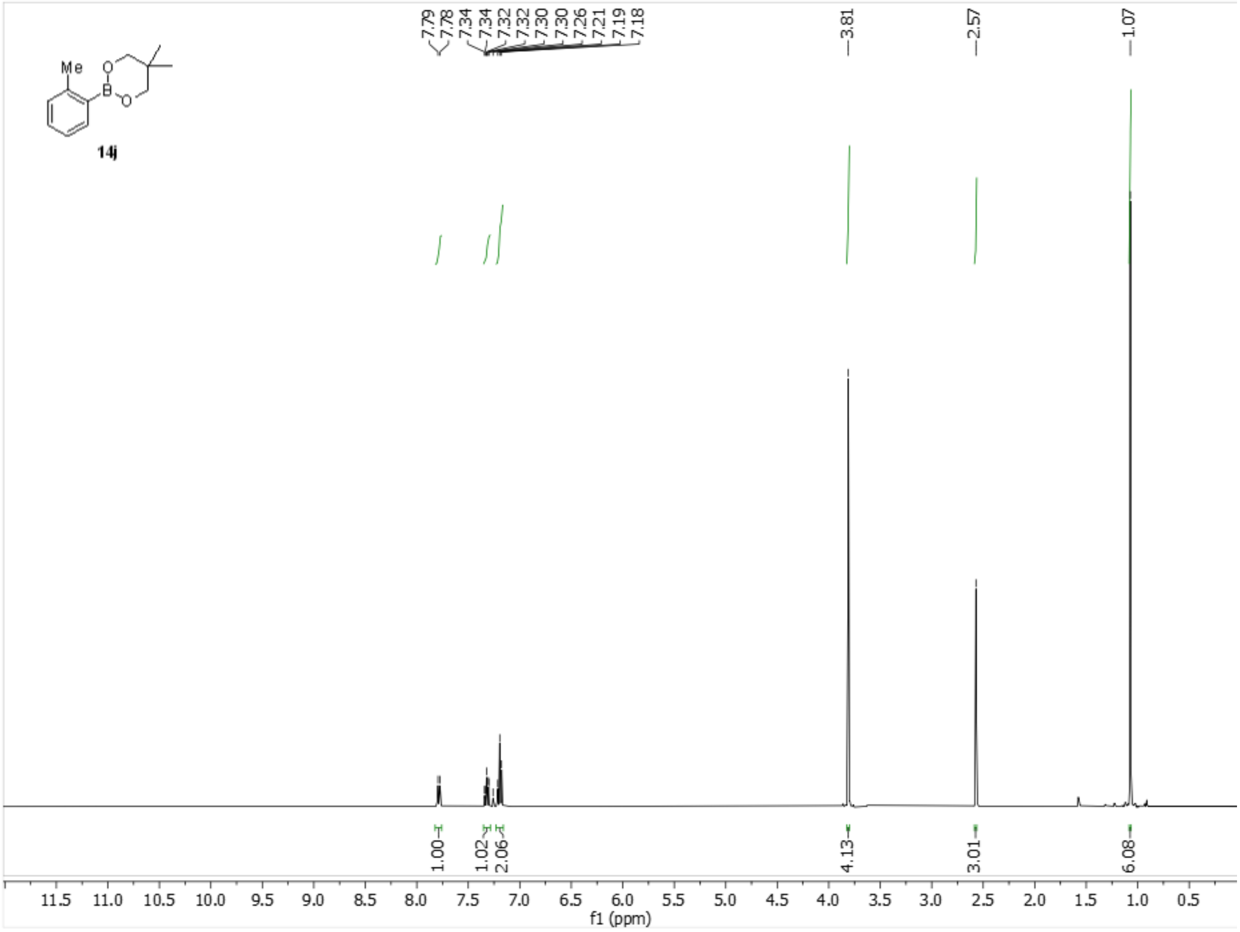

^{13}C NMR (101 MHz, $CDCl_3$)

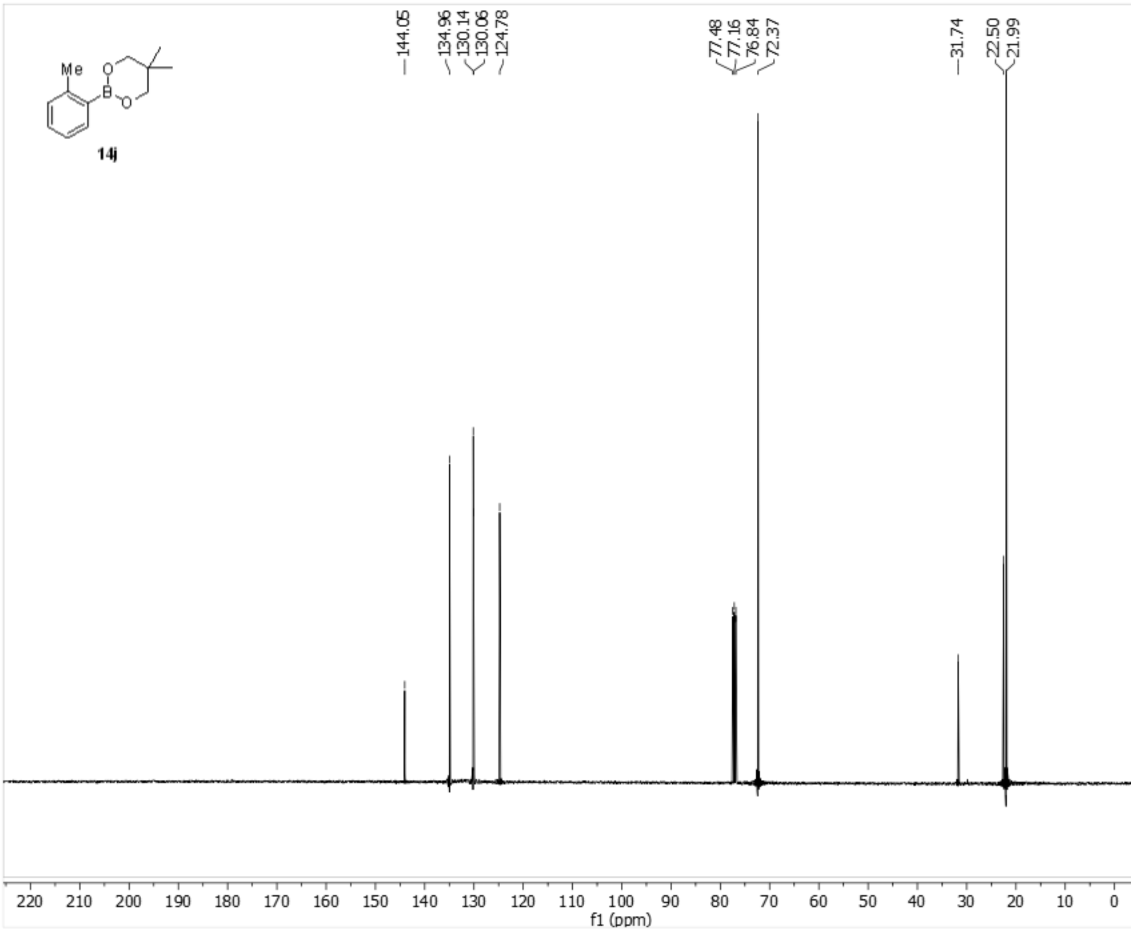

^{11}B NMR (128 MHz, $CDCl_3$)

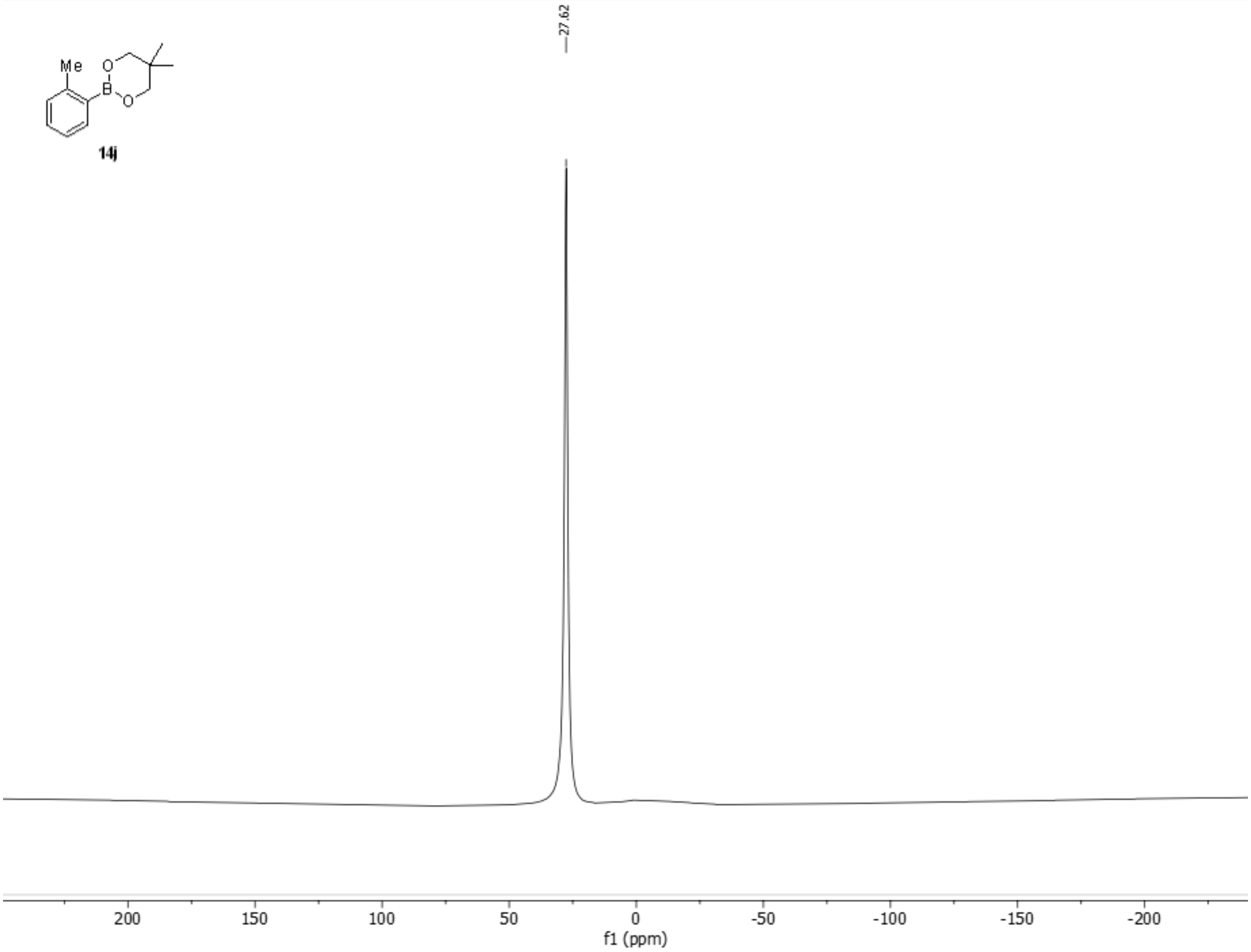

5,5-Dimethyl-2-(*m*-tolyl)-1,3,2-dioxaborinane 14k

^{1}H NMR (400 MHz, $CDCl_3$)

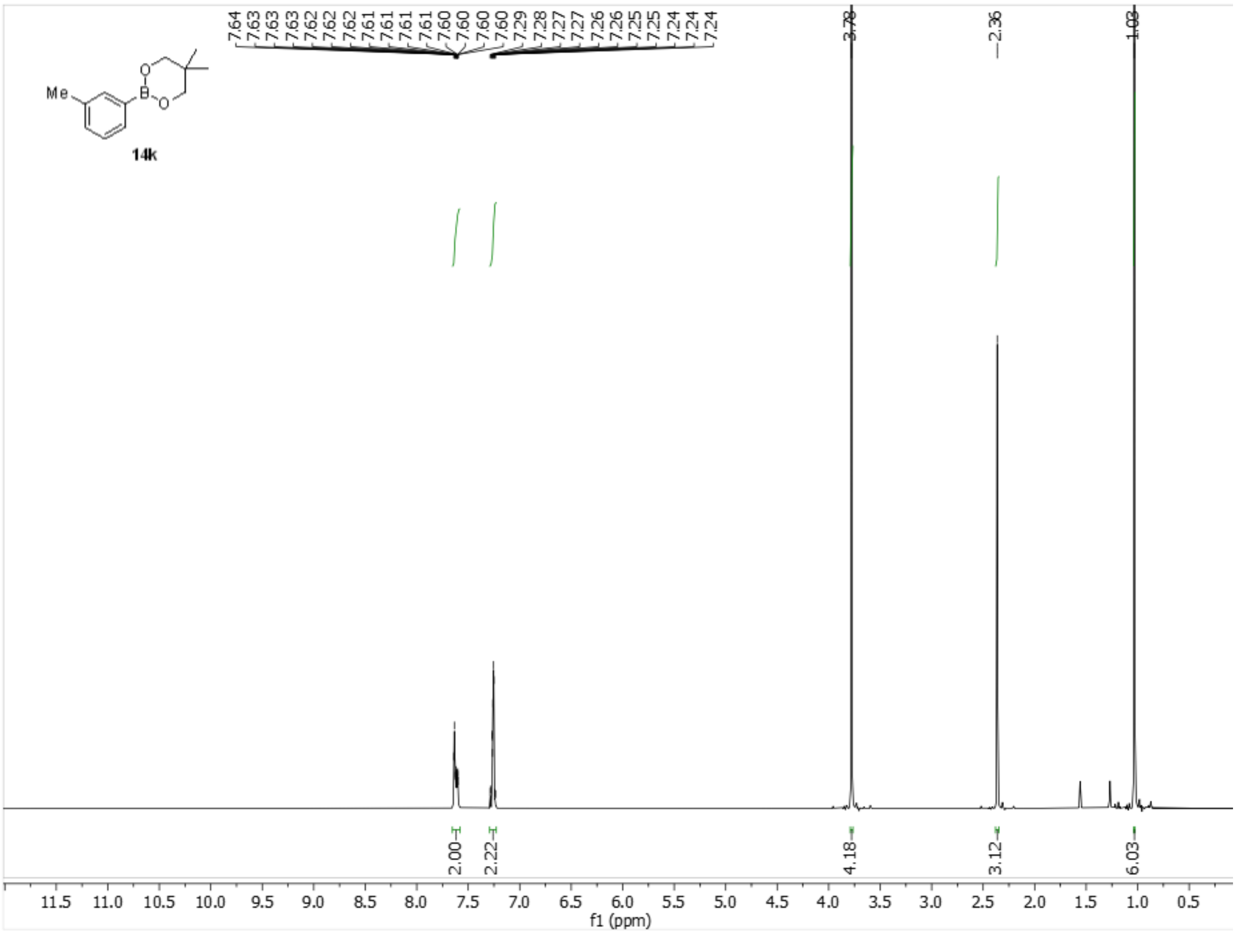

^{13}C NMR (101 MHz, $CDCl_3$)

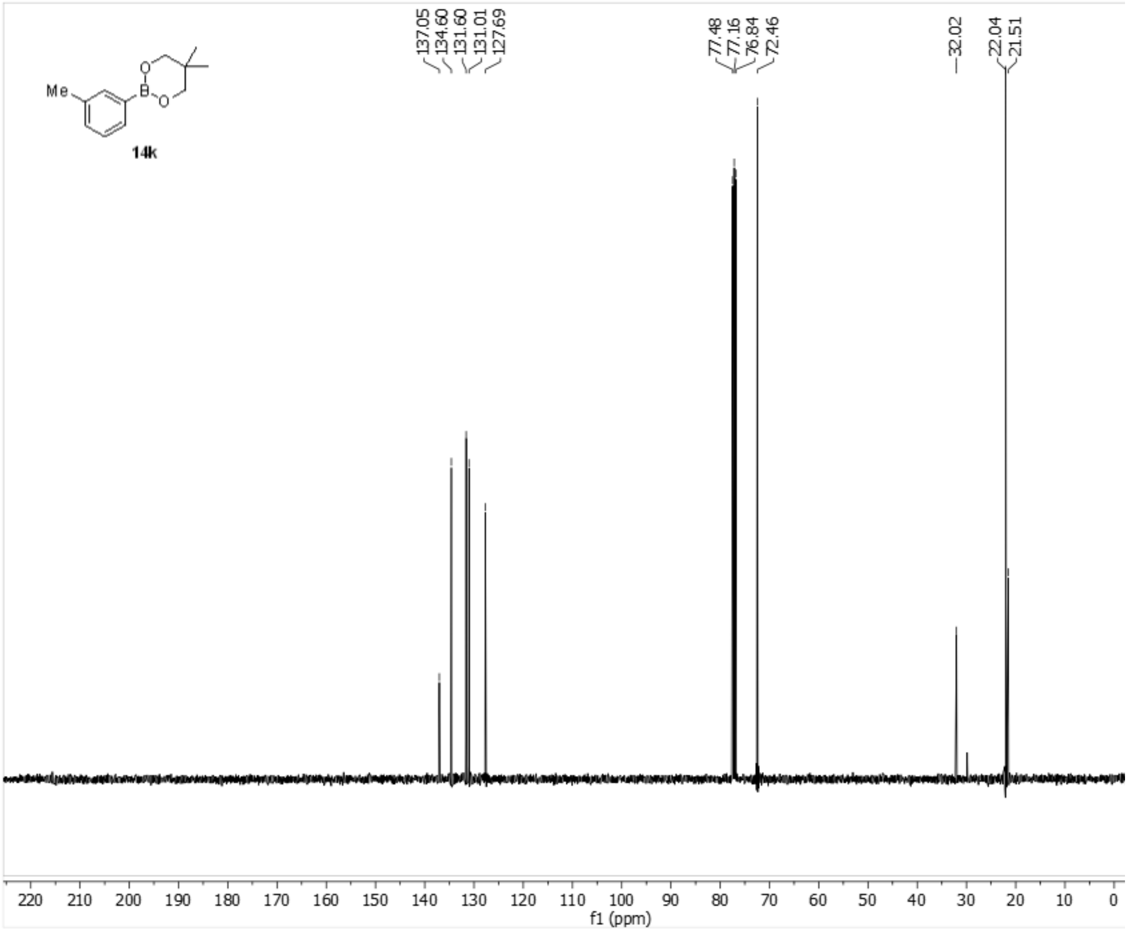

^{11}B NMR (128 MHz, $CDCl_3$)

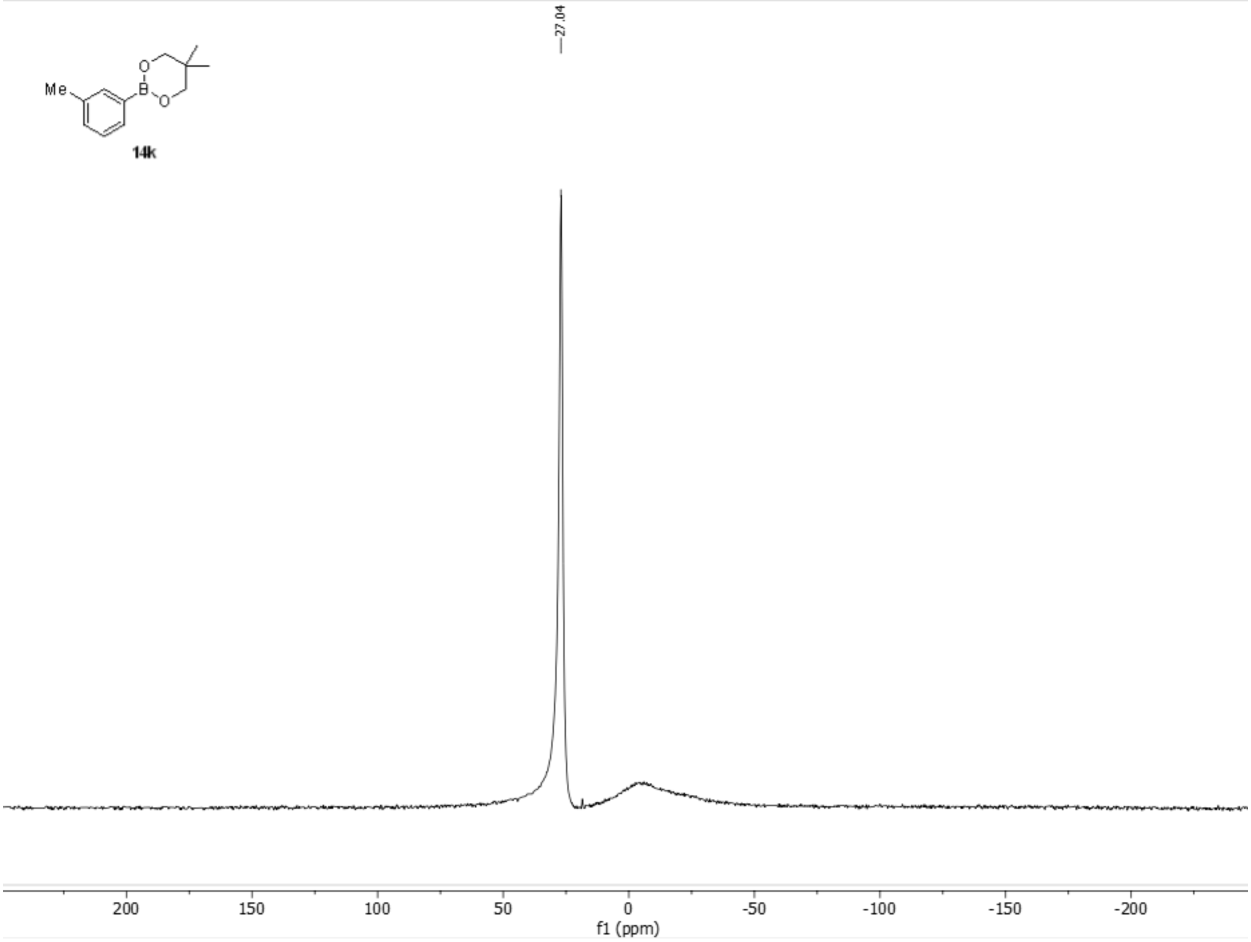

2-(3-Methoxyphenyl)-5,5-dimethyl-1,3,2-dioxaborinane 14l

^{1}H NMR (400 MHz, $CDCl_3$)

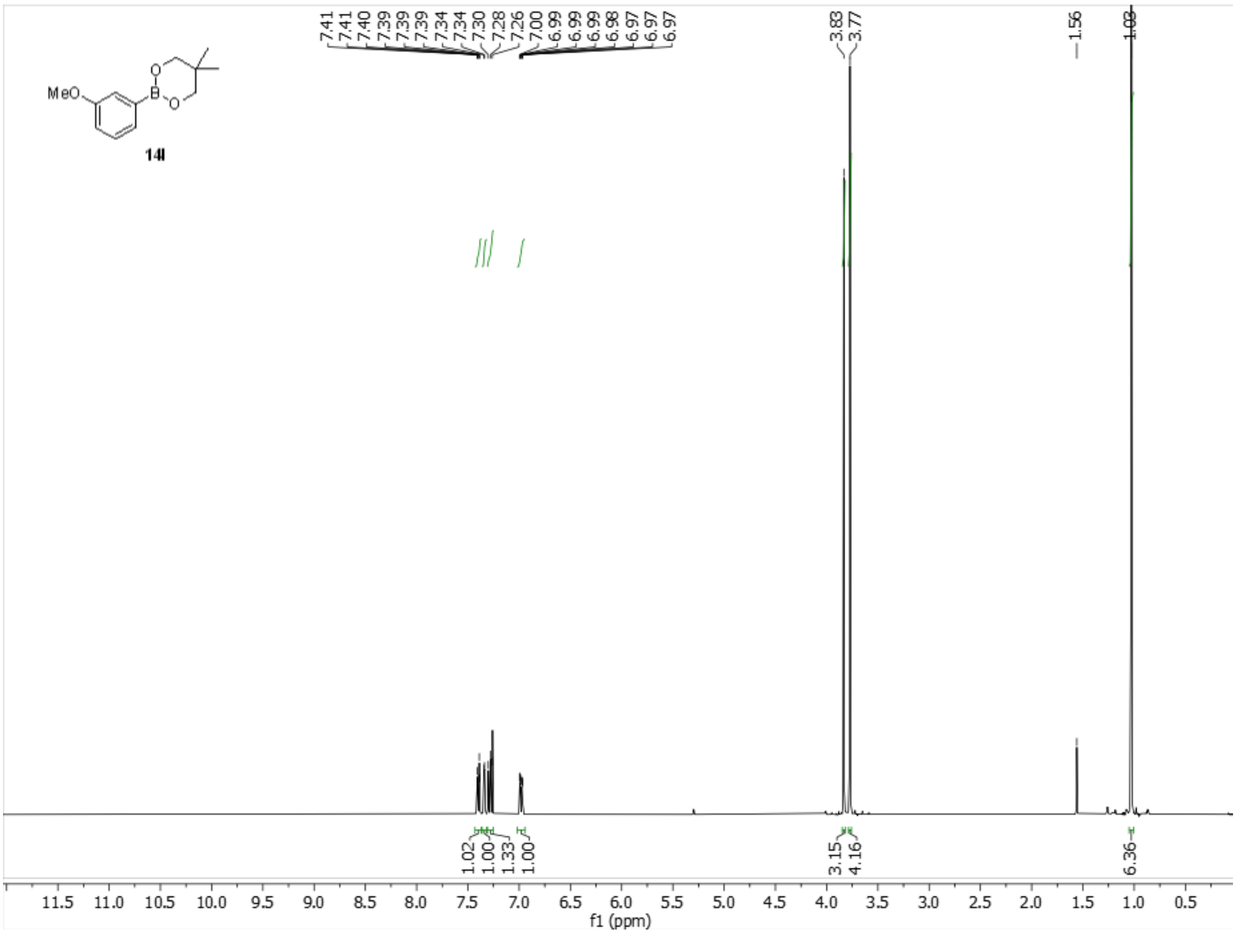

^{13}C NMR (101 MHz, $CDCl_3$)

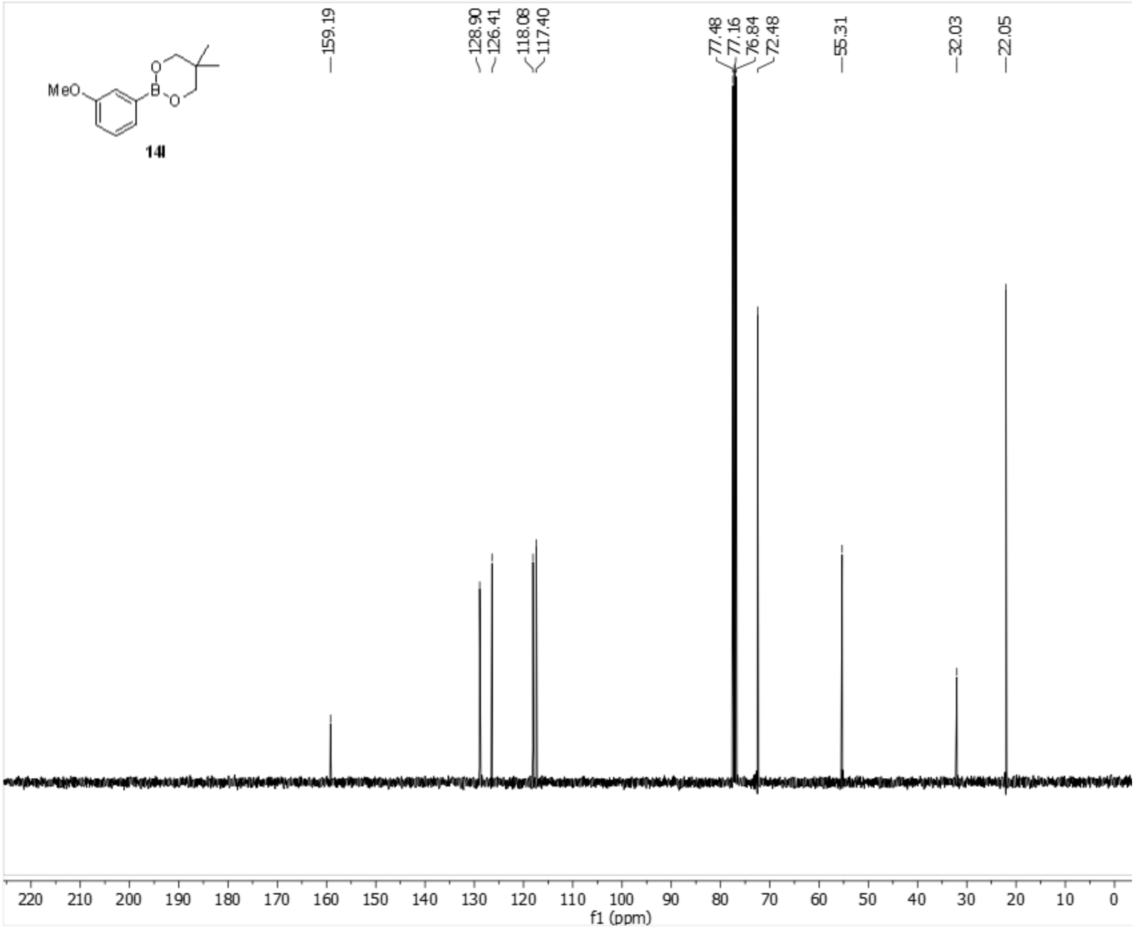

^{11}B NMR (128 MHz, $CDCl_3$)

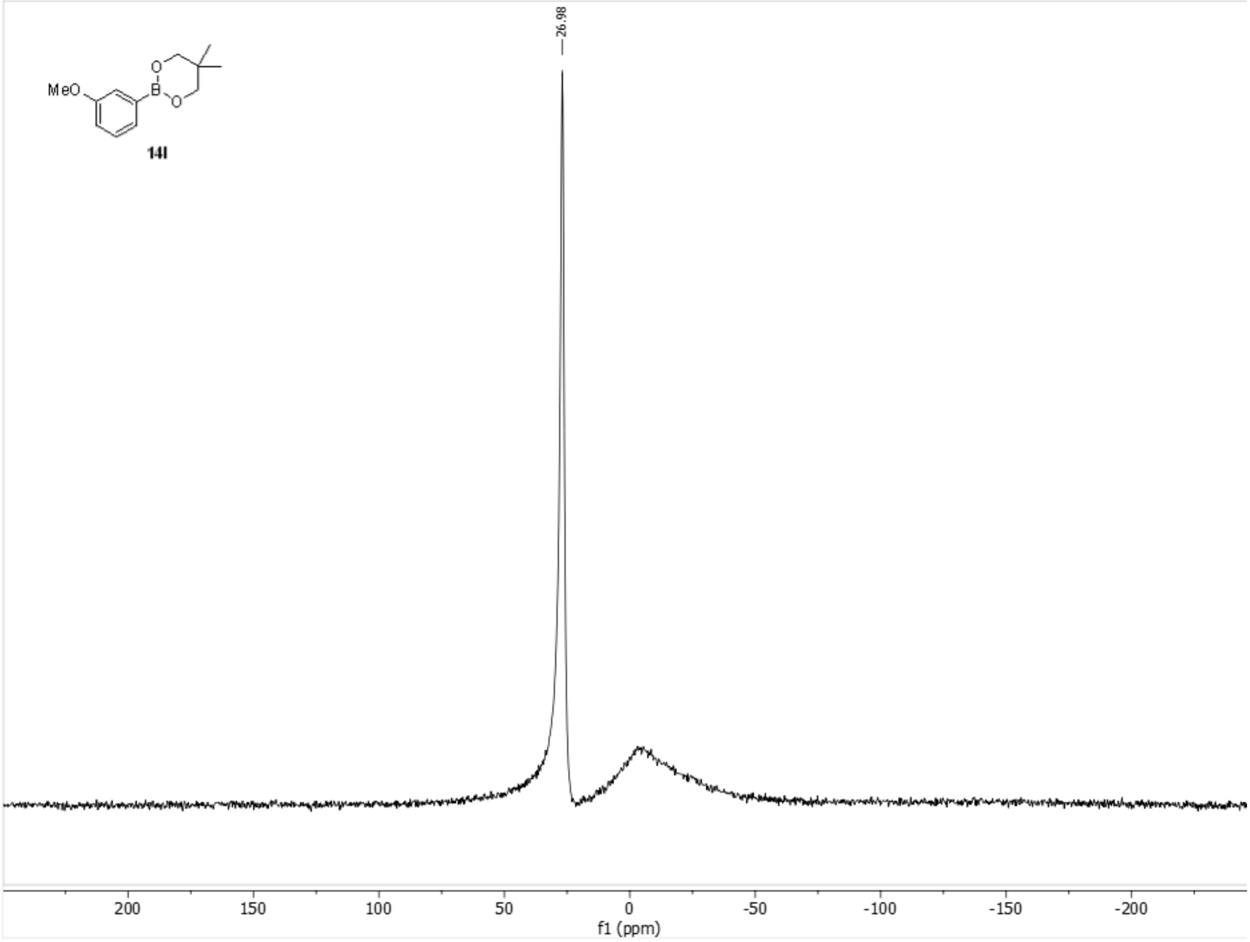

1-(4-Methoxyphenyl-2,6-d_2)piperidine *d*-16

^{1}H NMR (400 MHz, $CDCl_3$)

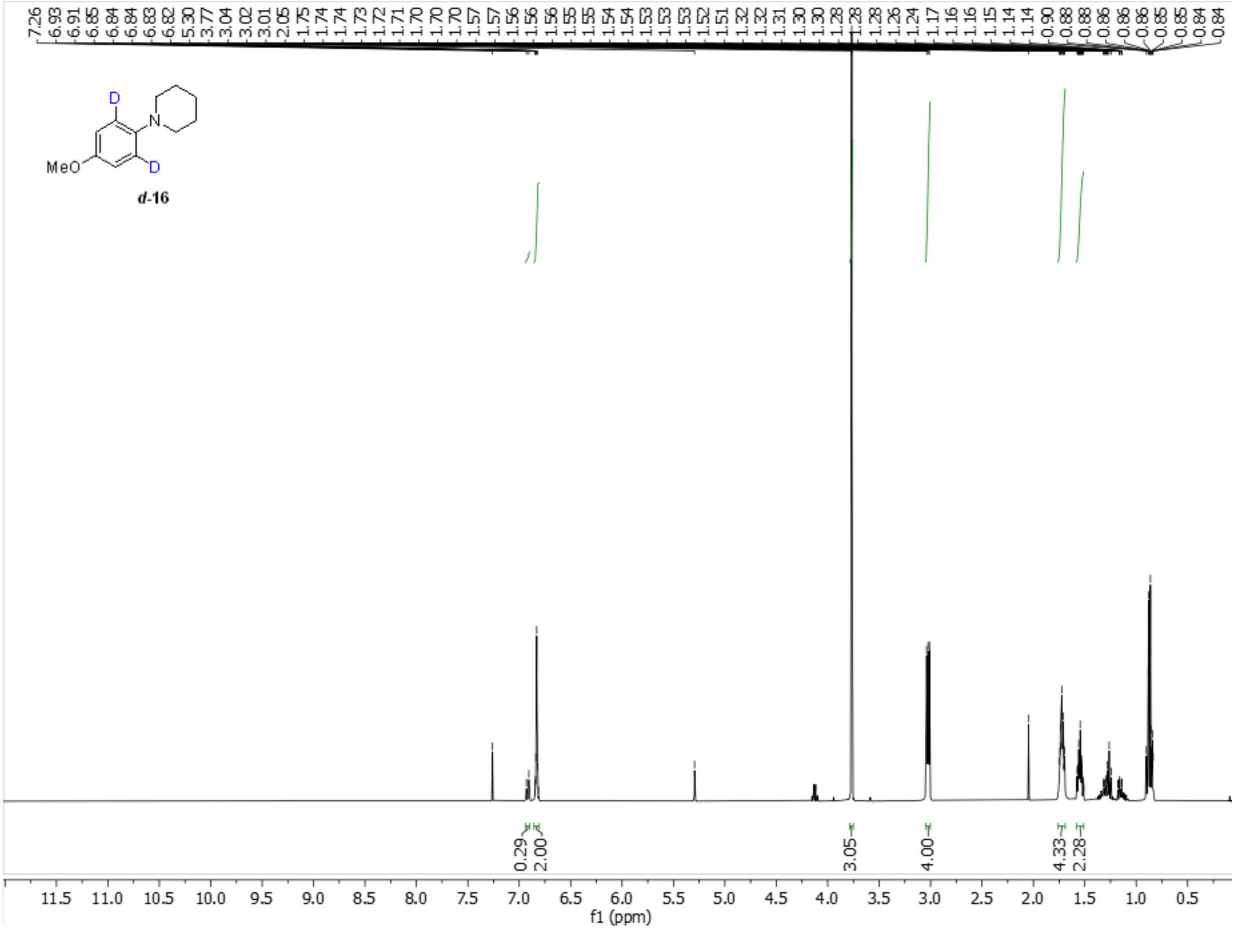

^{13}C NMR (101 MHz, $CDCl_3$)

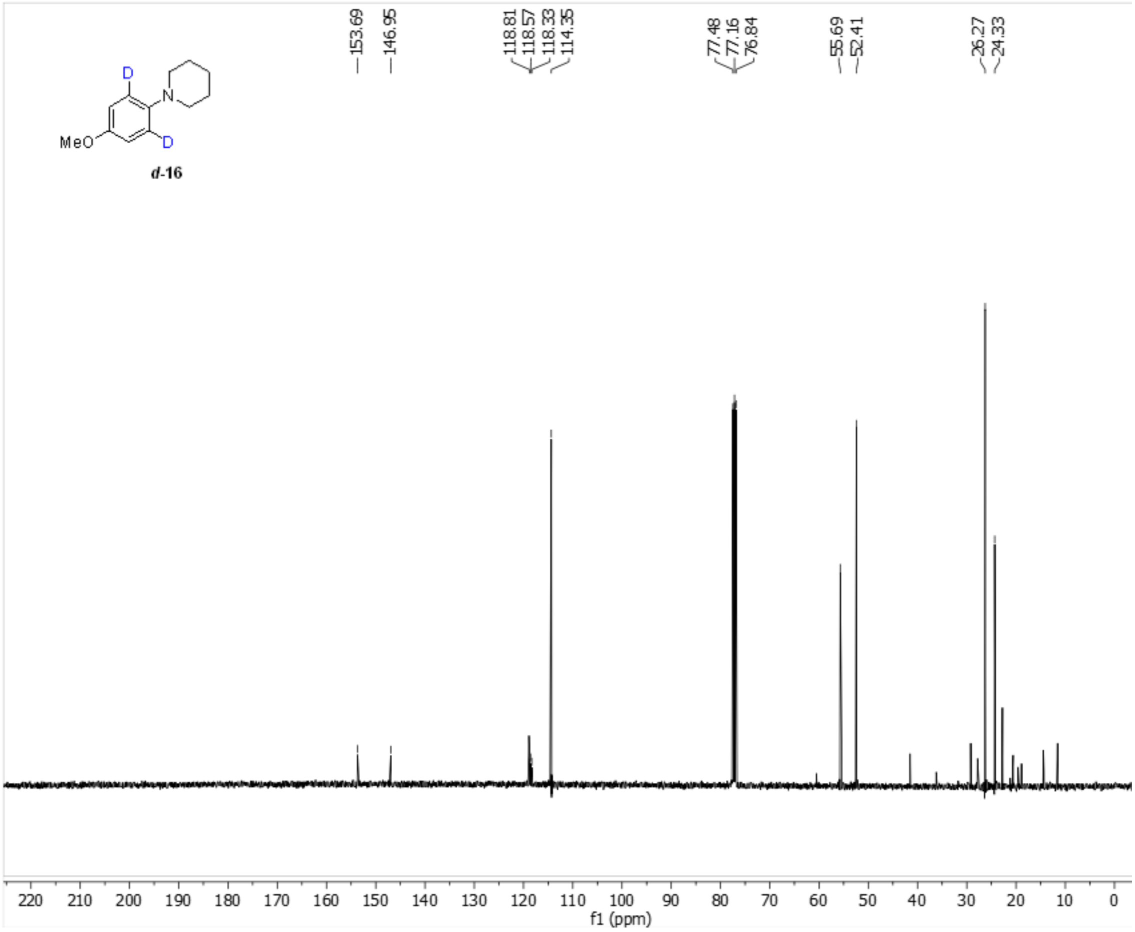

4-Methoxy-4'-nitro-1,1'-biphenyl-2,6-*d*$_2$ *d*-18

^{1}H NMR (400 MHz, CDCl$_3$)

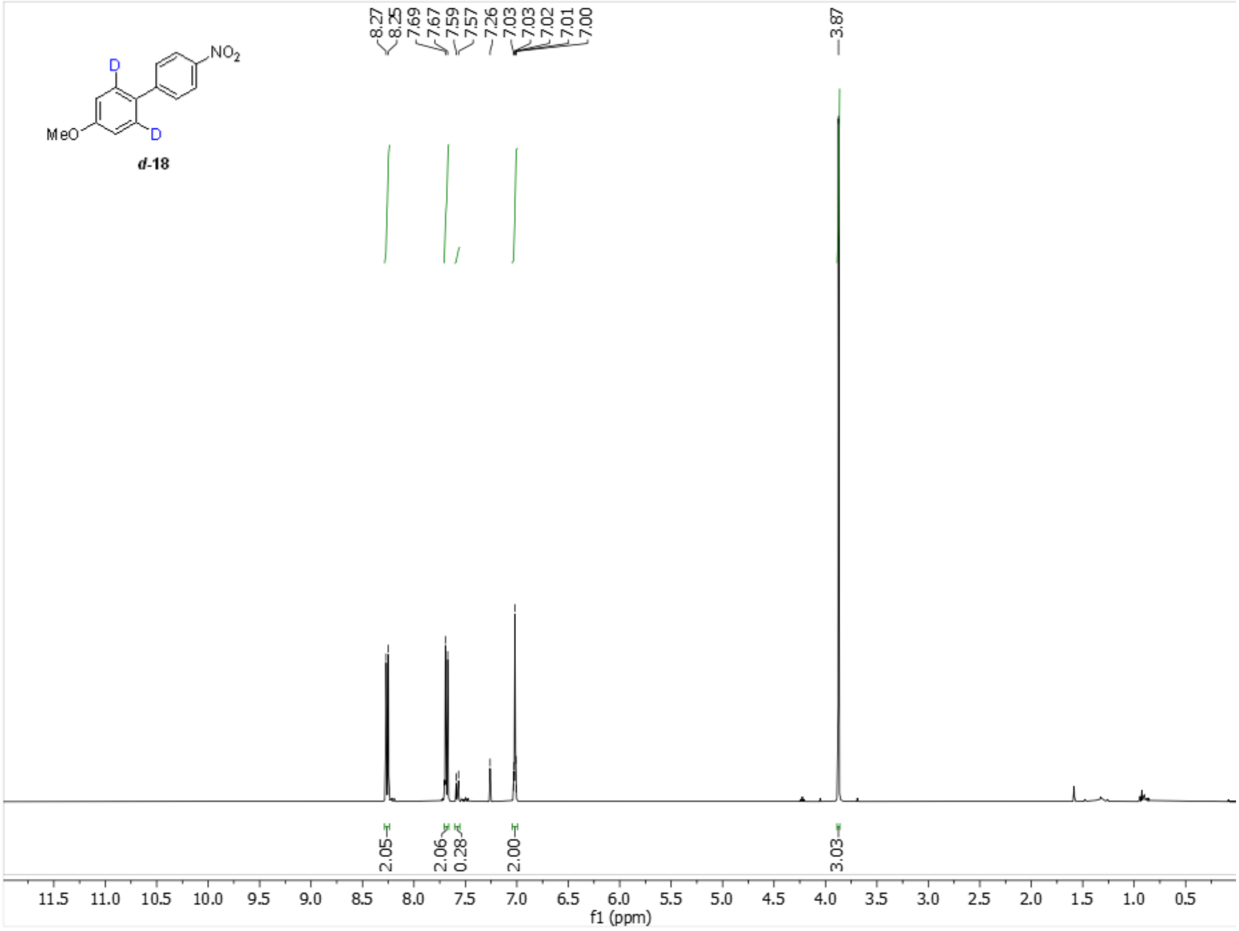

^{13}C NMR (101 MHz, $CDCl_3$)

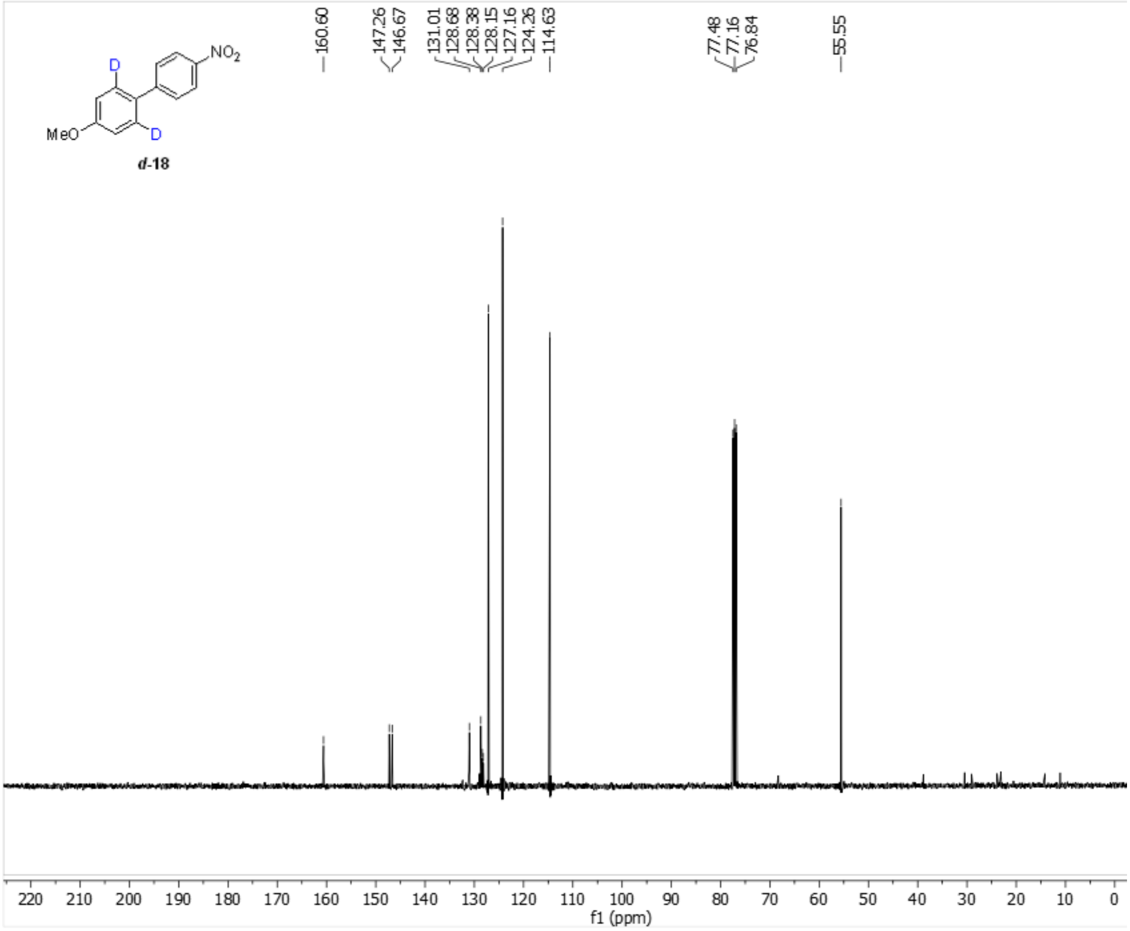

N-(1-(1H-Benzo[d][1,2,3]triazol-1-yl)ethyl)-4-bromoaniline S13

^1H NMR (400 MHz, DMSO)

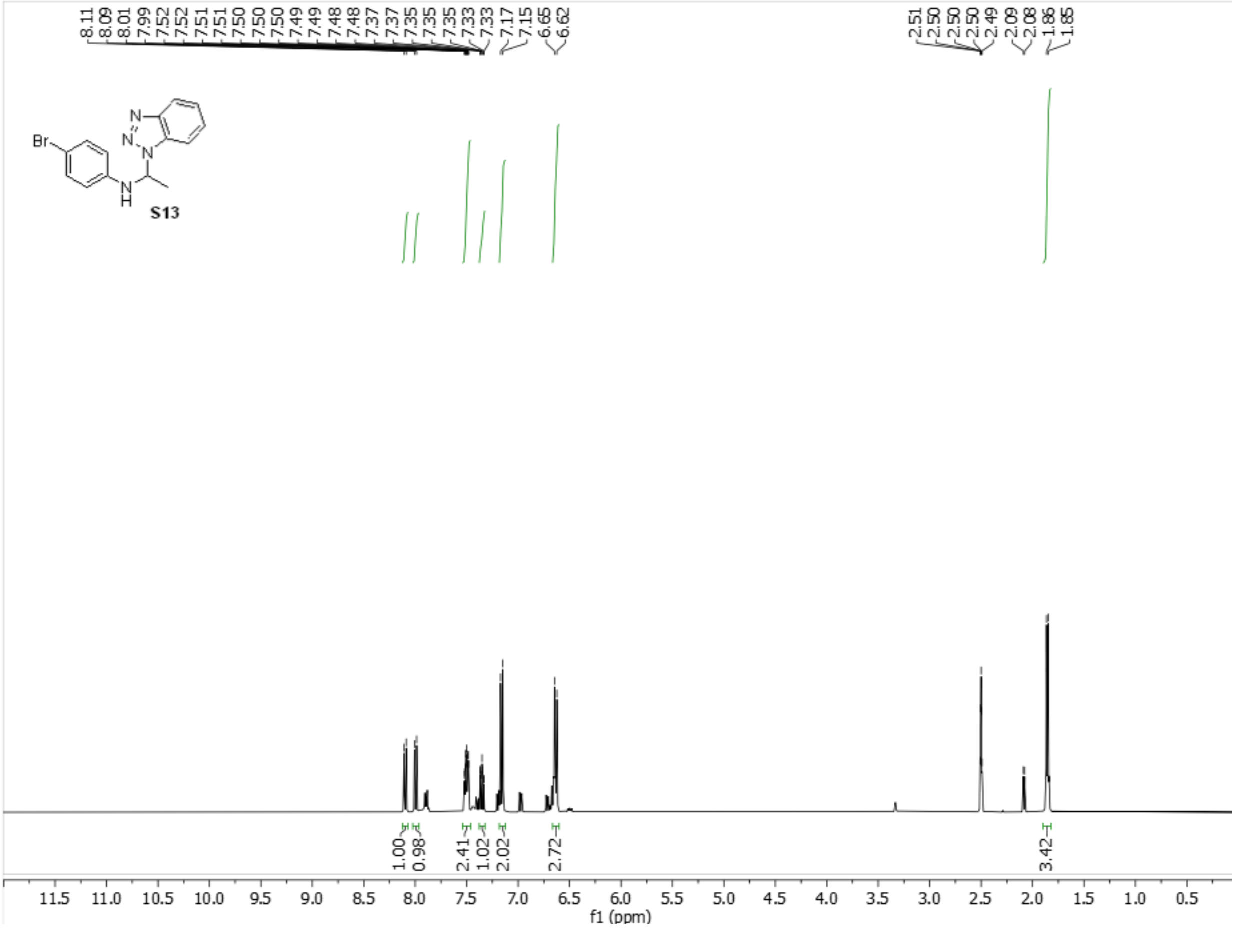

^{13}C NMR (101 MHz, DMSO)

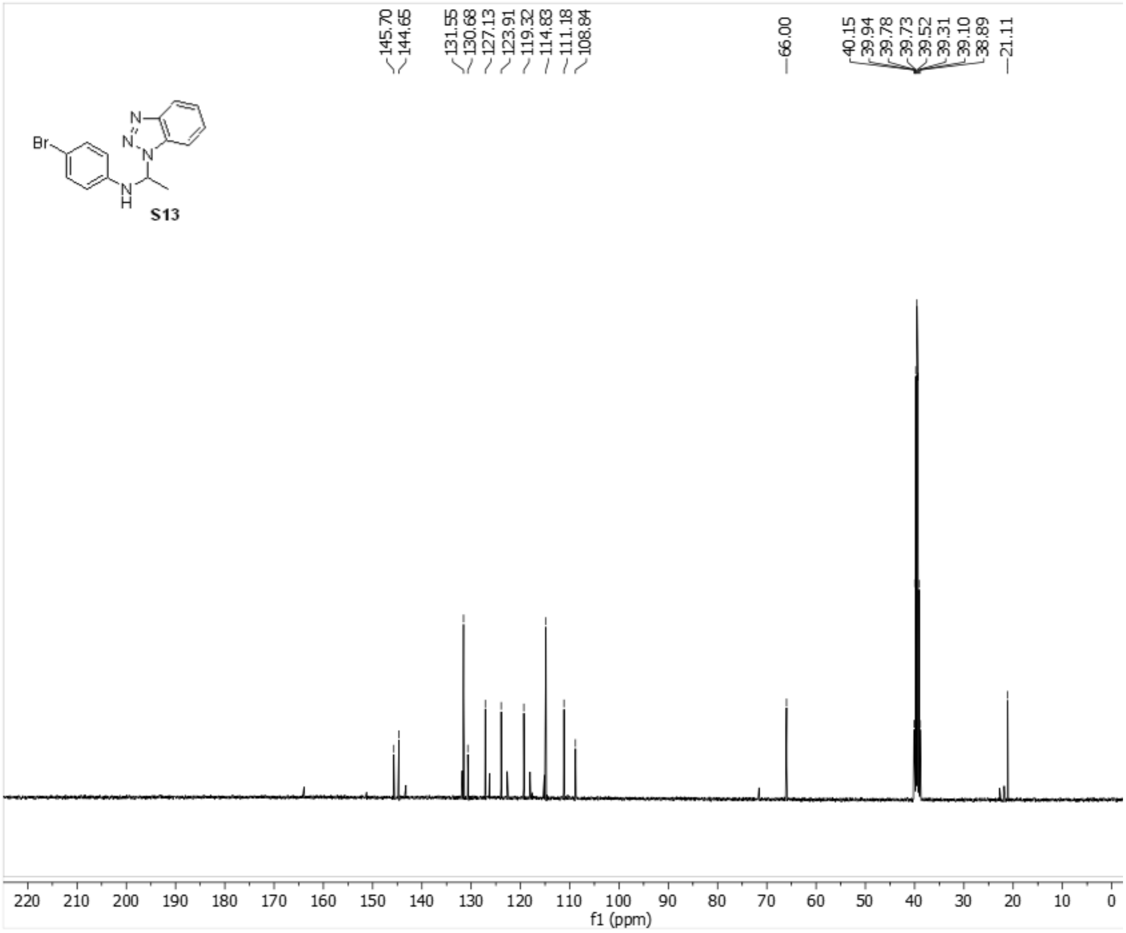

***N*-((2*S**,4*R**)-6-Bromo-2-methyl-1,2,3,4-tetrahydroquinolin-4-yl)formamide S14**

^{1}H NMR (400 MHz, DMSO)

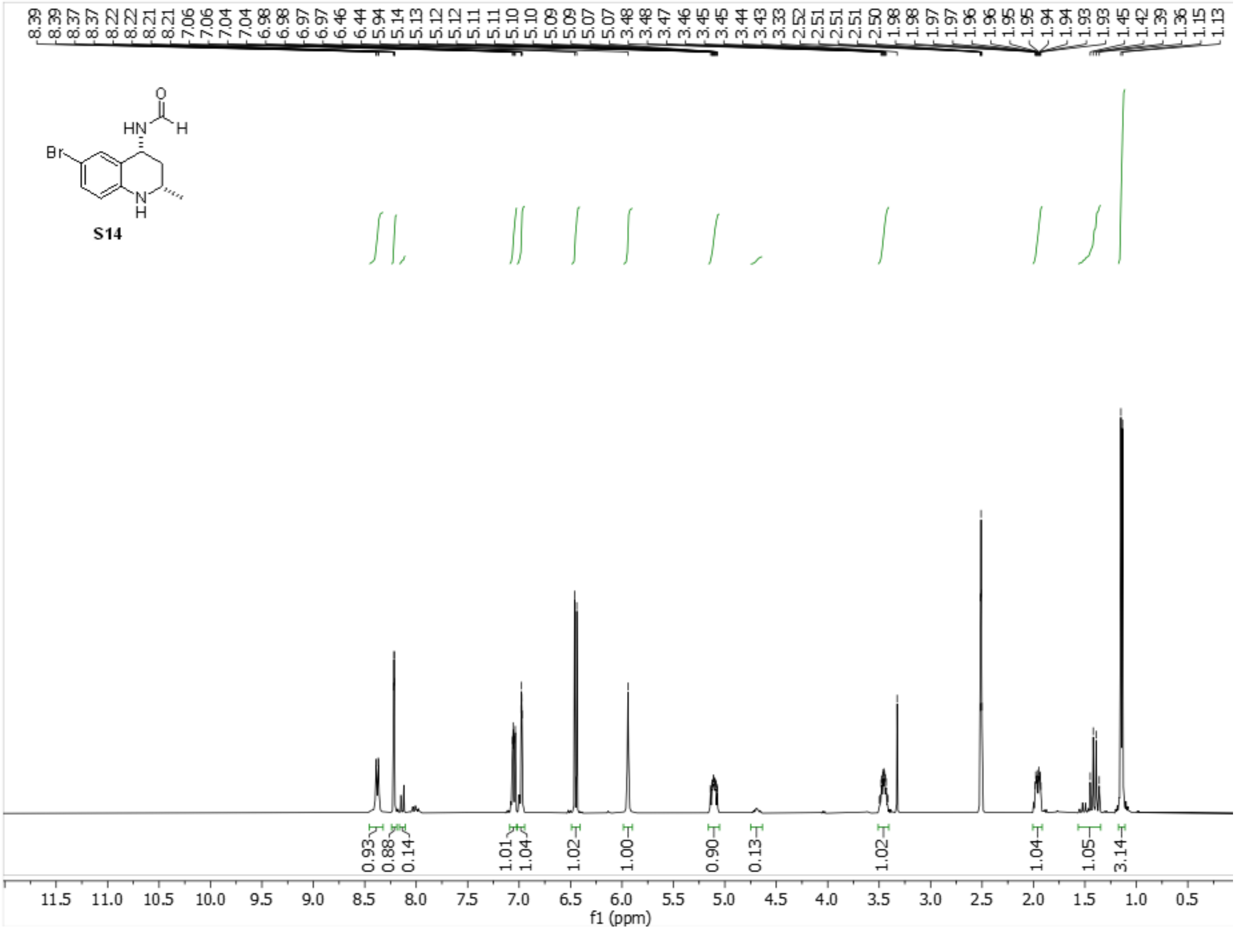

^{13}C NMR (101 MHz, DMSO)

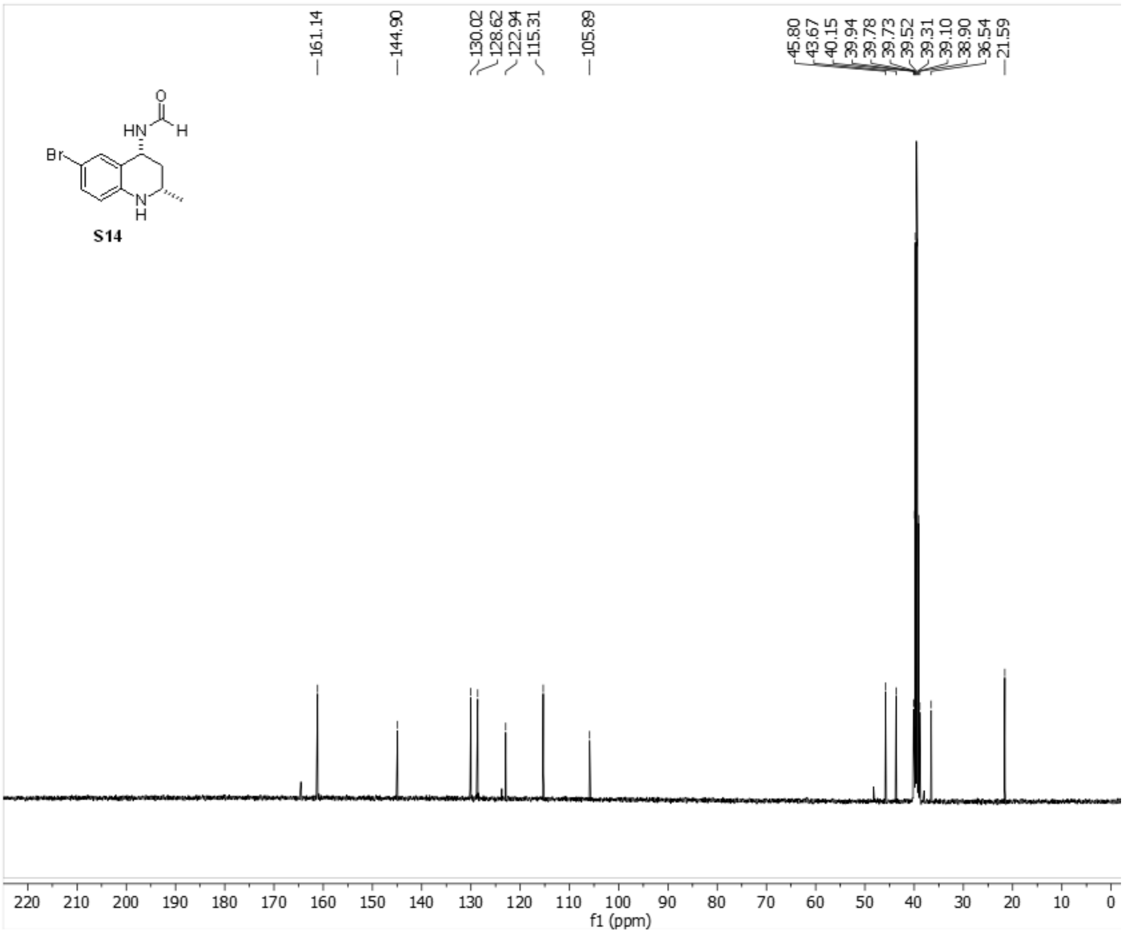

_N_-((2_S_*,4_R_*)-1-Acetyl-6-bromo-2-methyl-1,2,3,4-tetrahydroquinolin-4-yl)formamide S15

^{1}H NMR (400 MHz, DMSO)

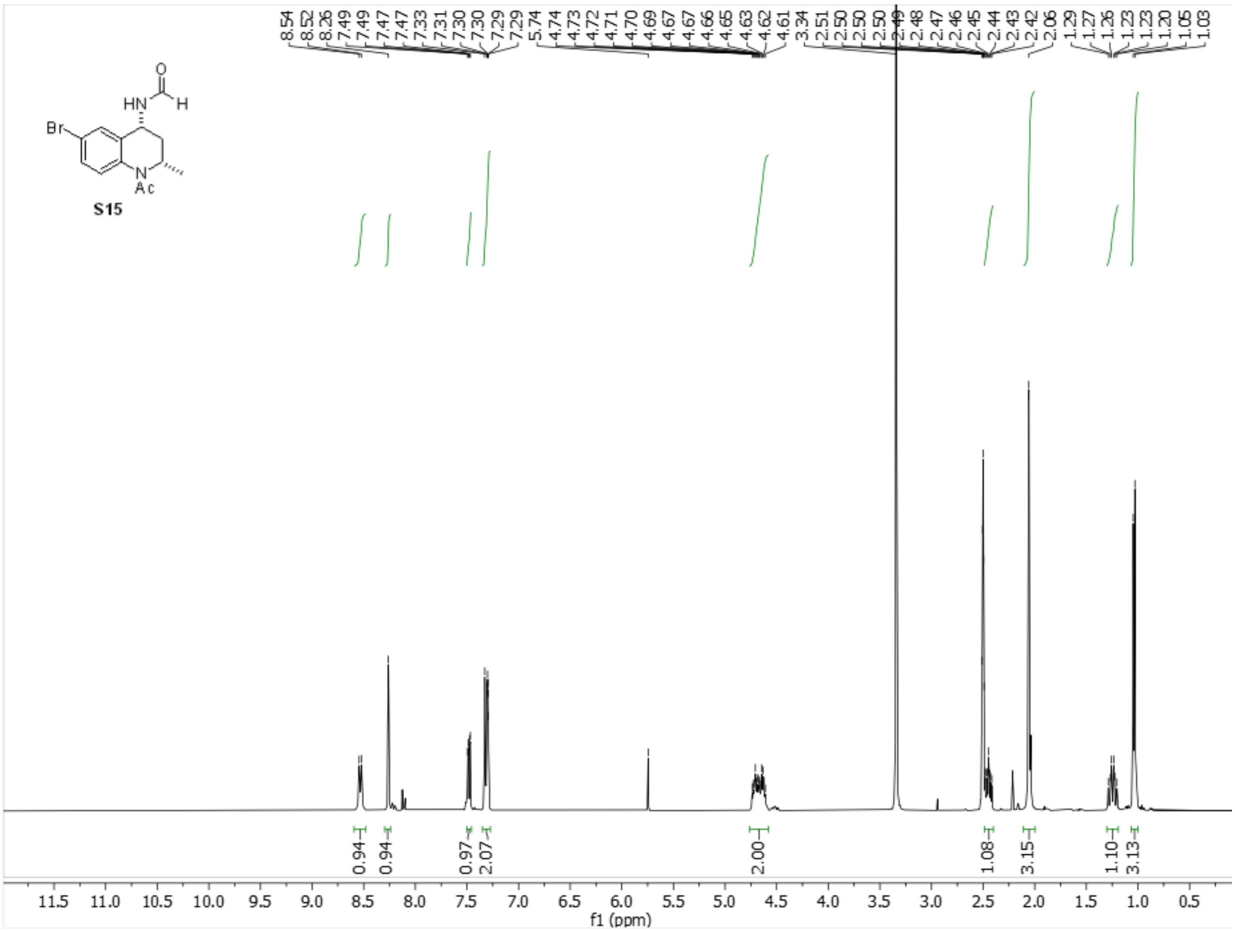

^{13}C NMR (101 MHz, $CDCl_3$)

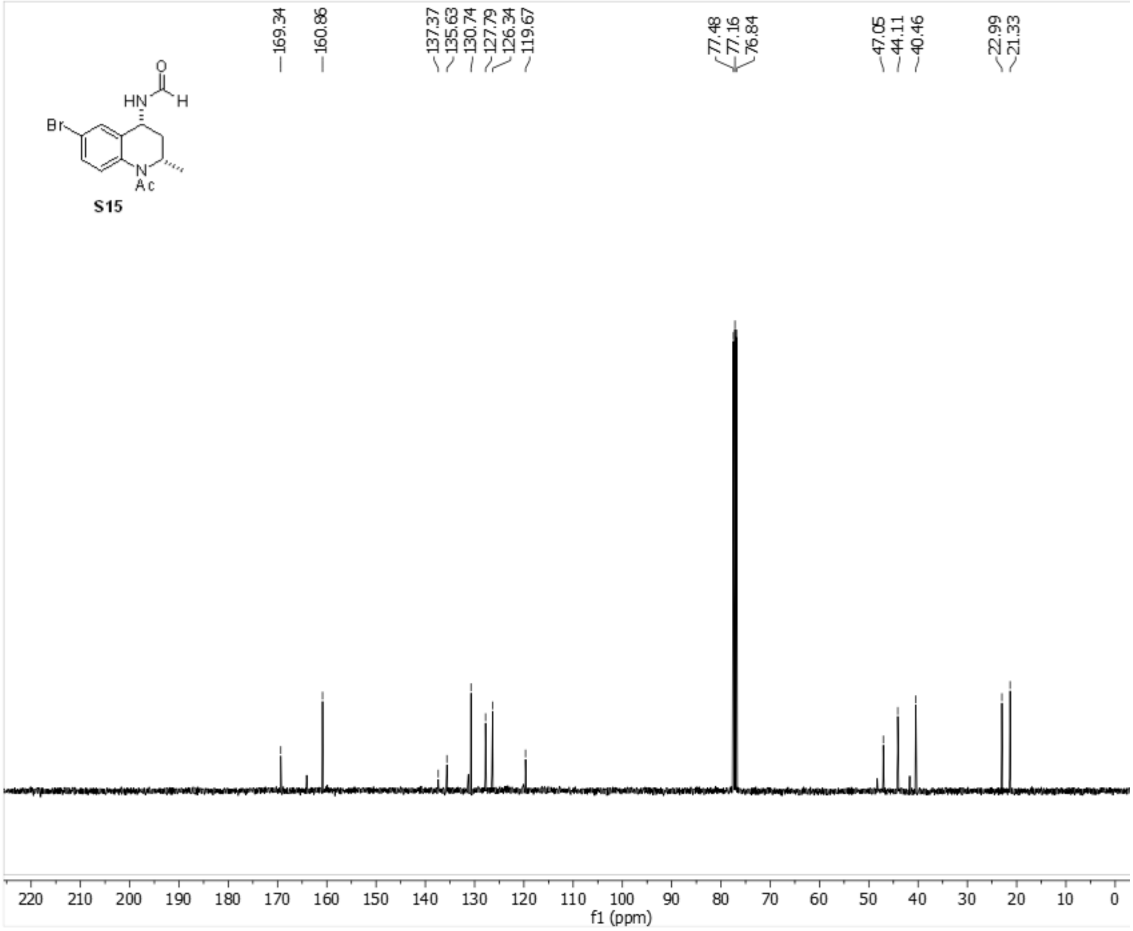

1-((2*S,4*R**)-4-Amino-6-bromo-2-methyl-3,4-dihydroquinolin-1(2*H*)-yl)ethan-1-one 19**

^{1}H NMR (400 MHz, $CDCl_3$)

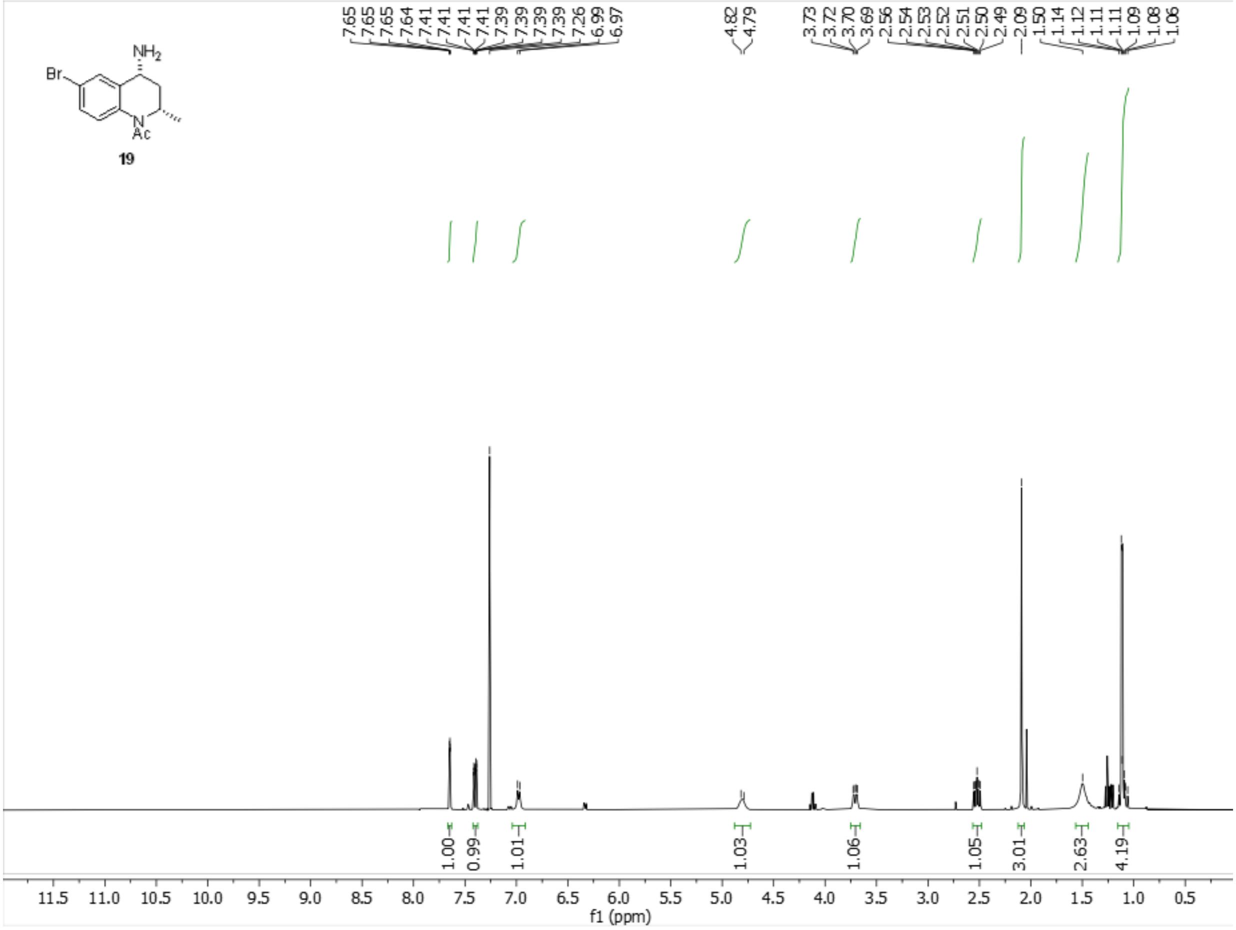

^{13}C NMR (101 MHz, $CDCl_3$)

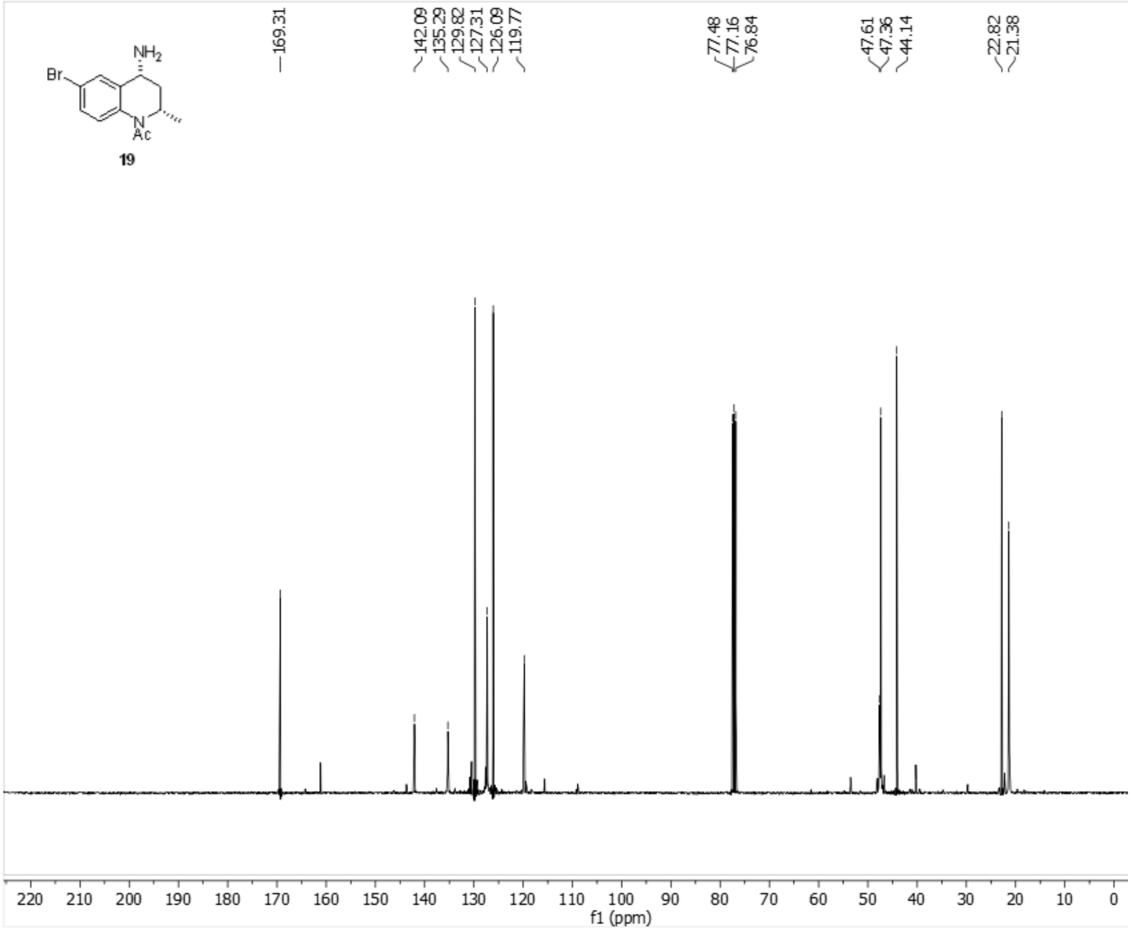

1-((2*S,4*R**)-6-Bromo-4-((4-chlorophenyl-2,6-d_2)amino)-2-methyl-3,4-dihydroquinolin-1(2*H*)-yl)ethan-1-one *d*-20**

^{1}H NMR (400 MHz, $CDCl_3$)

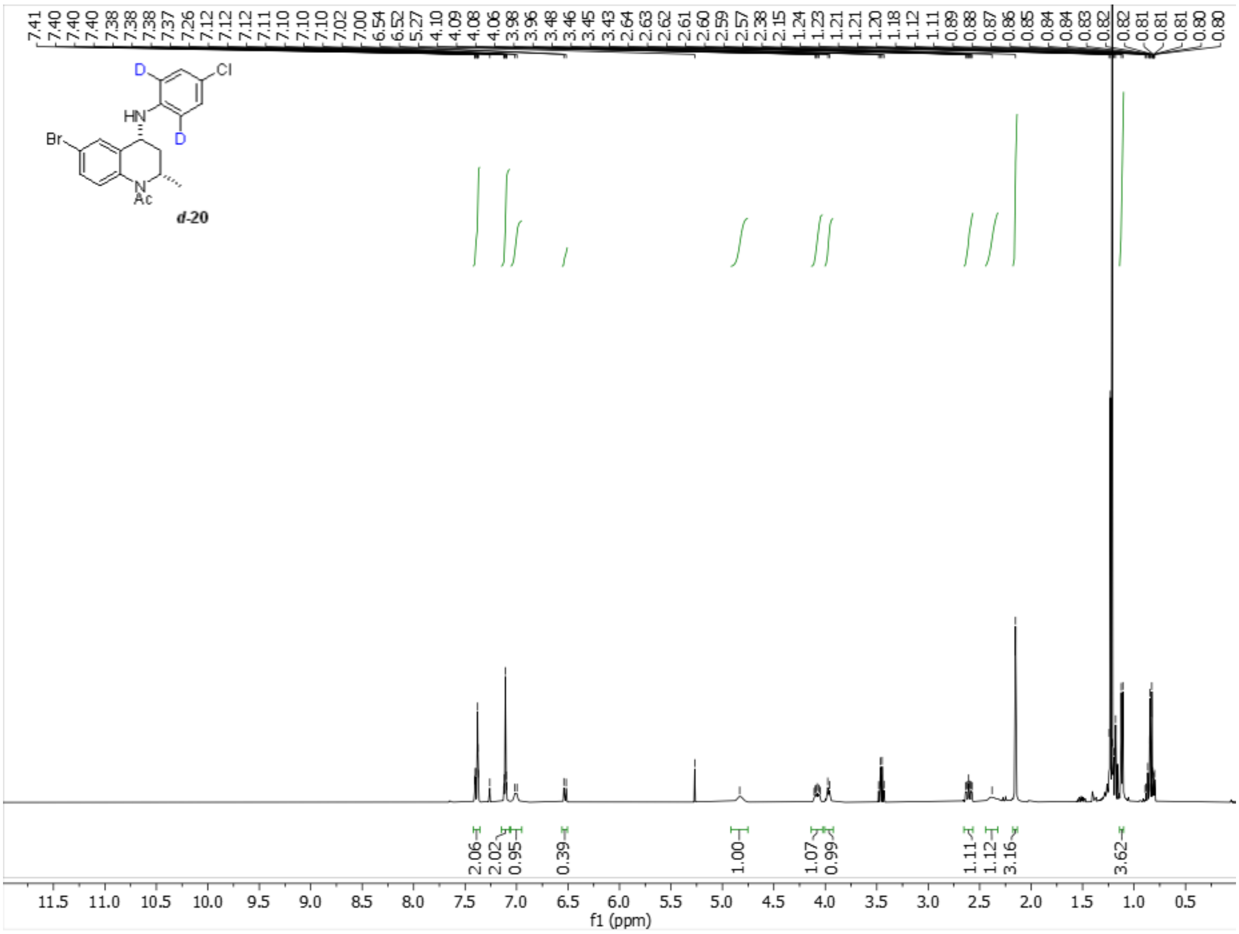

^{13}C NMR (101 MHz, $CDCl_3$)

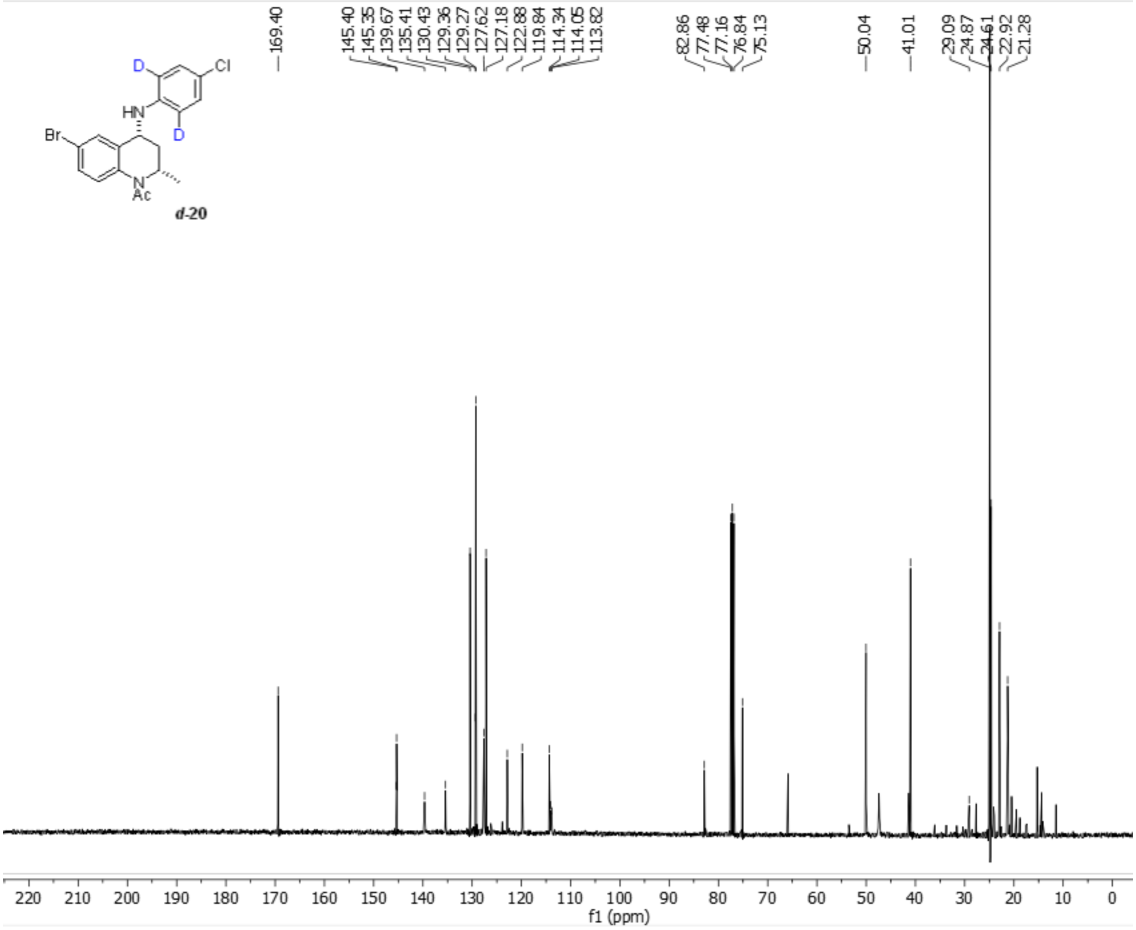

1-((2*S,4*R**)-4-((4-Chlorophenyl-2,6-*d*$_2$)amino)-2-methyl-6-(2-methylphenyl-6-*d*)-3,4-dihydroquinolin-1(2*H*)-yl)ethan-1-one *d*-21**

^{1}H NMR (400 MHz, CD_3CN)

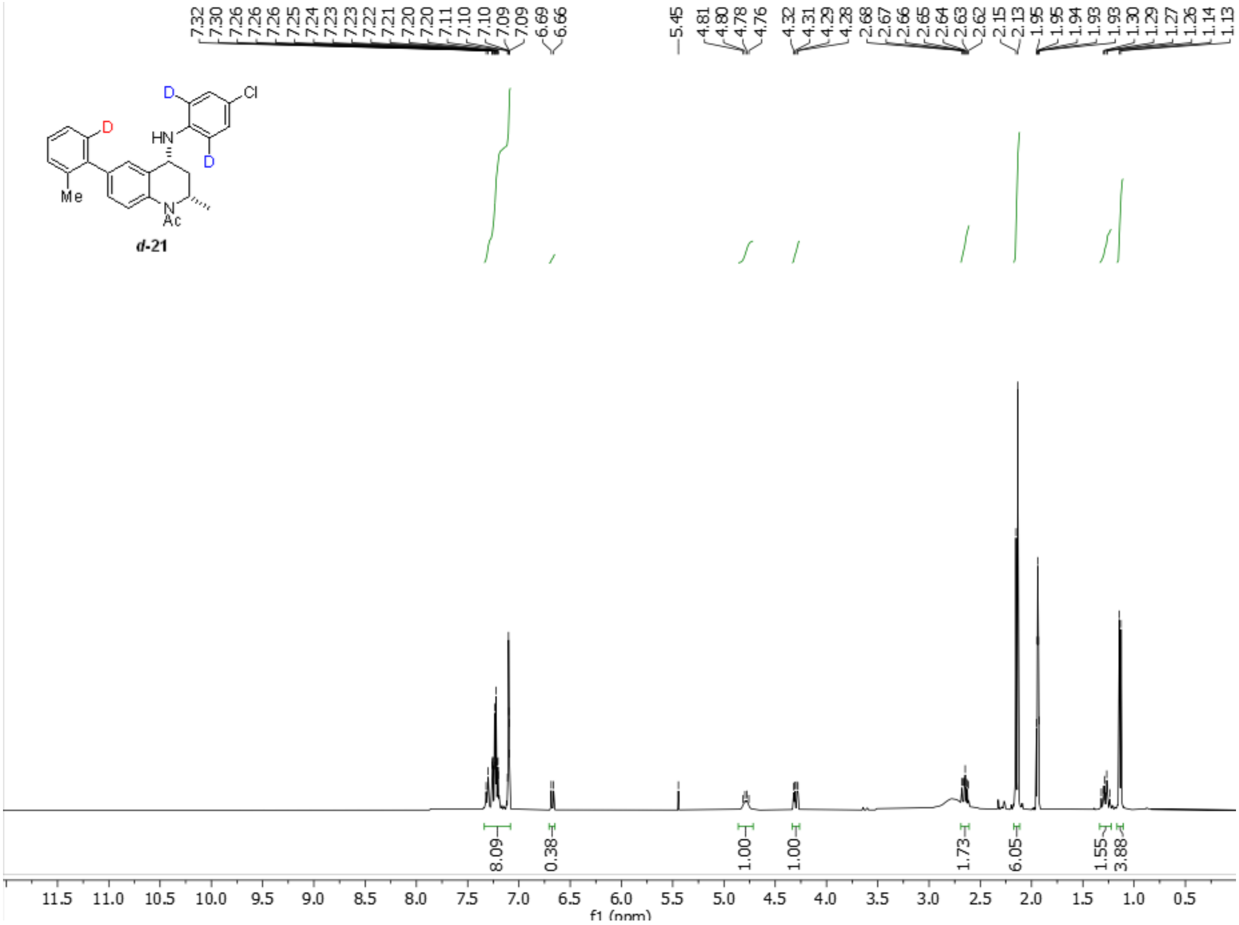

^{13}C NMR (101 MHz, CD_3CN)

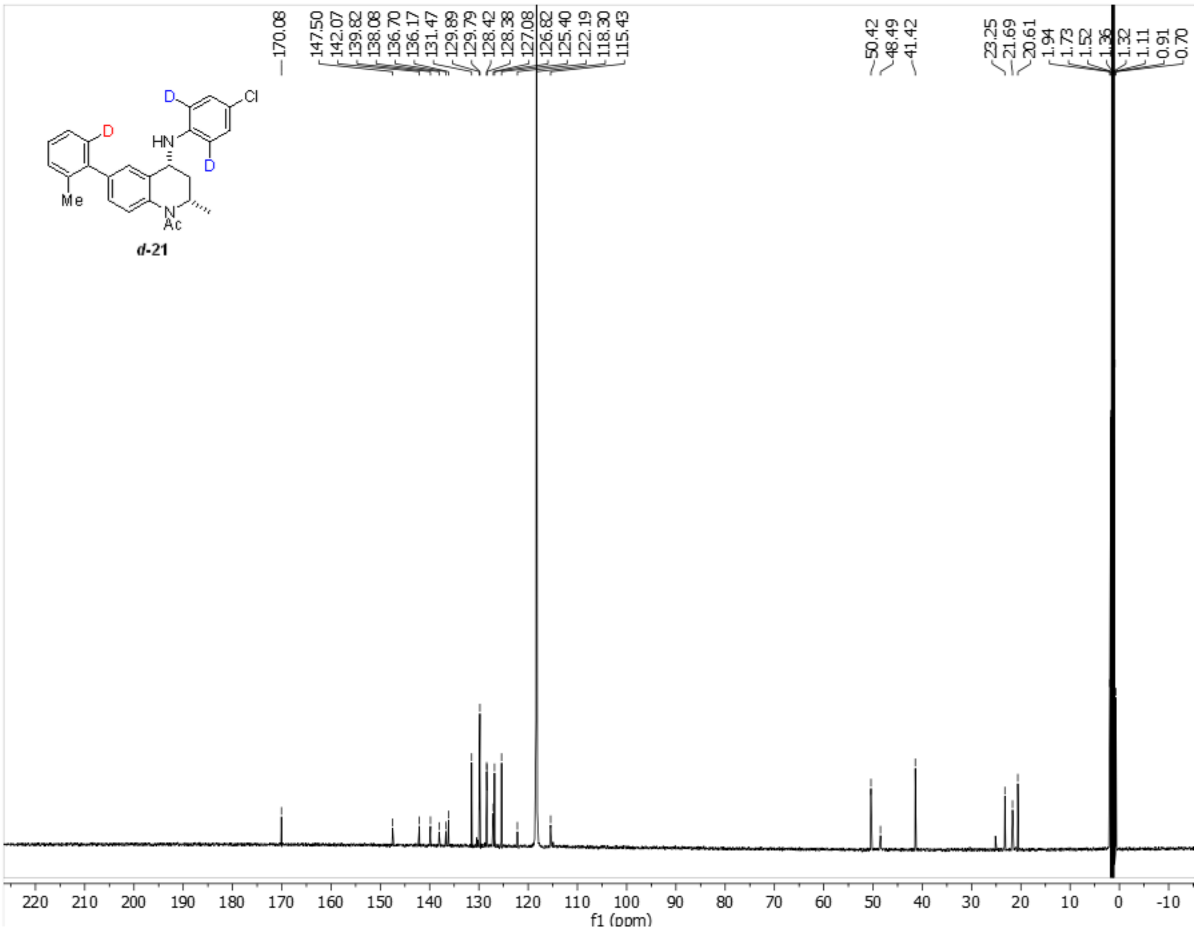

1.11.2 Cartesian coordinates (Å) for all Optimised Structures

Manuscript Scheme 2

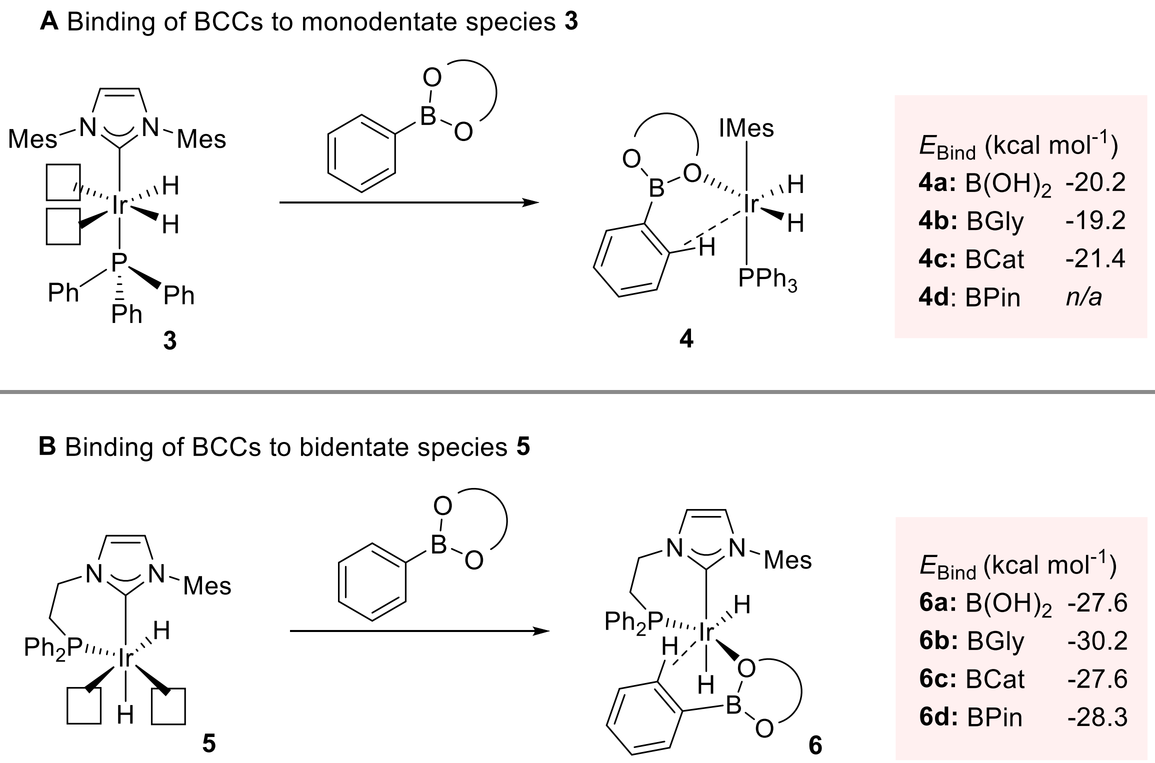

Free Catalyst 3

C	3.7256400	2.7731880	0.3053090
C	2.6316180	3.5719360	0.3699020
N	3.2781030	1.4626980	0.1966720
N	1.5393050	2.7249280	0.2993250
C	0.1291110	2.9894410	0.2817270
C	-0.4302910	3.7796420	-0.7336460
C	-0.6689170	2.3506050	1.2557270
C	-1.8147650	3.9242440	-0.7479570
C	-2.0564810	2.5185480	1.1753110
C	-2.6469450	3.2940730	0.1830420

H	-2.2626250	4.5301400	-1.5320550
H	-2.6810330	2.0401390	1.9252050
C	4.1226890	0.3027980	0.0869510
C	4.6520420	-0.0175670	-1.1657230
C	4.3601150	-0.4648180	1.2301280
C	5.4630800	-1.1474180	-1.2485260
C	5.1772480	-1.5840480	1.0919670
C	5.7421200	-1.9387400	-0.1340120
H	5.8843820	-1.4182990	-2.2130580
H	5.3783340	-2.1953890	1.9678770
C	3.7398010	-0.1085880	2.5457960
H	3.8847470	0.9466730	2.7967850
H	2.6582830	-0.2856260	2.5301910
H	4.1610190	-0.7064650	3.3541860
C	4.3434790	0.8149200	-2.3718840
H	3.2644680	0.9426660	-2.5063180
H	4.7721270	1.8194480	-2.2962710
H	4.7426760	0.3570460	-3.2768360
C	6.6460690	-3.1271820	-0.2433310
H	6.3458250	-3.9273130	0.4358560
H	6.6658040	-3.5289160	-1.2576160
H	7.6751640	-2.8587040	0.0163210
C	-4.1344340	3.4227000	0.0887780
H	-4.4398750	4.4543610	-0.0987400
H	-4.5206150	2.8202800	-0.7410320
H	-4.6273090	3.0799140	0.9996110
C	-0.0745030	1.5875470	2.4118920
H	0.1338660	0.5051500	2.2283450

H	0.8745080	2.0129200	2.7450710
H	-0.7750850	1.5691430	3.2490060
C	0.4235110	4.4113330	-1.7889020
H	1.1798810	3.7156900	-2.1641940
H	-0.1830500	4.7404620	-2.6326010
H	0.9573240	5.2904740	-1.4154330
C	1.9275010	1.4227480	0.1927310
Ir	0.3718850	0.1005840	0.0988250
H	1.2509160	-1.1631860	0.2956080
H	0.6718170	-0.2637220	-1.3818680
P	-1.5703810	-1.2070570	-0.1087110
C	-1.3876920	-3.0161240	-0.2108670
C	-0.3091410	-3.5360760	-0.9339960
C	-2.3167370	-3.8896420	0.3623250
C	-0.1672410	-4.9091690	-1.0861010
H	0.4189950	-2.8613580	-1.3752410
C	-2.1650260	-5.2639440	0.2134760
H	-3.1576850	-3.4973600	0.9267720
C	-1.0931660	-5.7740740	-0.5106810
H	0.6707550	-5.3053440	-1.6493900
H	-2.8871650	-5.9363720	0.6639480
H	-0.9773690	-6.8465090	-0.6246960
C	-2.5219900	-0.7381150	-1.5925970
C	-2.3907840	0.5680410	-2.0734890
C	-3.4161990	-1.6123120	-2.2155870
C	-3.1556380	1.0014930	-3.1497190
H	-1.6812880	1.2471170	-1.6028340
C	-4.1734870	-1.1787010	-3.2978510

H	-3.5172510	-2.6327920	-1.8568600
C	-4.0487860	0.1274830	-3.7618760
H	-3.0470600	2.0174540	-3.5168480
H	-4.8625420	-1.8628330	-3.7812020
H	-4.6417960	0.4608620	-4.6067740
C	-2.7454240	-0.9322180	1.2673130
C	-4.0237320	-0.4023560	1.0751340
C	-2.3075160	-1.2026780	2.5700800
C	-4.8459480	-0.1439500	2.1685990
H	-4.3791570	-0.1873670	0.0717310
C	-3.1321890	-0.9480180	3.6580030
H	-1.3185480	-1.6278760	2.7298830
C	-4.4027980	-0.4124430	3.4586250
H	-5.8389060	0.2637150	2.0085680
H	-2.7872450	-1.1719340	4.6620040
H	-5.0471830	-0.2131280	4.3080670
H	4.7764040	3.0091730	0.3351610
H	2.5390000	4.6398000	0.4723960

Phenylboronic acid 10

C	2.6327820	0.0000030	-0.0000060
C	1.9360830	1.2049790	0.0001300
C	0.5467880	1.2014680	0.0001440
C	-0.1748450	-0.0000070	0.0000070
C	0.5467950	-1.2014740	-0.0001380
C	1.9360930	-1.2049760	-0.0001360
H	3.7185720	0.0000090	-0.0000100
H	2.4789900	2.1451260	0.0002350

H	0.0024080	2.1415040	0.0002640
H	0.0024260	-2.1415170	-0.0002510
H	2.4790020	-2.1451210	-0.0002450
B	-1.7284870	-0.0000020	0.0000150
O	-2.3704100	1.2108370	-0.0002340
H	-3.3272240	1.1579360	-0.0007070
O	-2.3704230	-1.2108340	0.0002450
H	-3.3272370	-1.1579230	0.0005400

Phenylboronic acid ethylene glycol ester (PhBGly) 11

C	-2.6829520	-1.2054730	0.0120150
C	-1.2938990	-1.2027110	0.0115400
C	-0.5739230	0.0000280	-0.0000240
C	-1.2939390	1.2027330	-0.0115700
C	-2.6829980	1.2054490	-0.0120040
C	-3.3788910	-0.0000230	0.0000220
H	-3.2262120	-2.1451720	0.0213100
H	-0.7500730	-2.1431560	0.0200720
H	-0.7501560	2.1432030	-0.0201400
H	-3.2262840	2.1451320	-0.0212890
H	-4.4645820	-0.0000450	0.0000460
B	0.9682050	0.0000250	-0.0000060
O	1.7221170	-1.1453120	0.0225060
O	1.7221560	1.1453330	-0.0225110
C	3.0958700	0.7663250	0.0586970
H	3.5027980	1.1153470	1.0138660
H	3.6529390	1.2564750	-0.7444360
C	3.0958490	-0.7663550	-0.0586780

H	3.6528890	-1.2565050	0.7444780
H	3.5027740	-1.1154120	-1.0138320

Phenylboronic acid catechol ester (PhBCat)

C	3.8758050	-1.2061800	0.0000280
C	2.4875250	-1.2040950	0.0000650
C	1.7679510	0.0004150	0.0000200
C	2.4882760	1.2044160	-0.0000610
C	3.8765600	1.2056260	-0.0000990
C	4.5716920	-0.0004910	-0.0000520
H	4.4186530	-2.1458830	0.0000610
H	1.9454410	-2.1454120	0.0001260
H	1.9468210	2.1461020	-0.0000920
H	4.4199840	2.1449950	-0.0001630
H	5.6572080	-0.0008340	-0.0000810
B	0.2366340	0.0005320	0.0000630
C	-1.8461490	0.6961230	0.0000250
C	-3.0165720	1.4268700	0.0001190
C	-1.8457900	-0.6958040	-0.0001190
C	-4.2076920	0.6967700	0.0000330
H	-3.0042320	2.5101380	0.0002270
C	-3.0159350	-1.4270370	-0.0001500
C	-4.2073880	-0.6974960	-0.0001070
H	-5.1526740	1.2285850	0.0001100
H	-3.0030860	-2.5103070	-0.0004390
H	-5.1521430	-1.2297060	-0.0002170
O	-0.5519380	1.1468790	-0.0000310
O	-0.5516670	-1.1462600	0.0002740

Phenylboronic acid bound complex 4a

C	1.7999230	-4.2632490	0.8046930
C	0.4811820	-4.5430830	0.9174310
N	1.9024500	-2.9077610	0.5471040
N	-0.1914810	-3.3484810	0.7239120
C	-1.6164650	-3.1970210	0.7383660
C	-2.2413700	-2.6729840	1.8749330
C	-2.3225590	-3.5102090	-0.4311710
C	-3.6171310	-2.4302260	1.7968150
C	-3.6977520	-3.2856480	-0.4432820
C	-4.3635440	-2.7361130	0.6569110
H	-4.1220380	-2.0151220	2.6660470
H	-4.2584260	-3.5222140	-1.3443860
C	3.1453140	-2.2171910	0.3308950
C	3.7627160	-1.5878300	1.4174230
C	3.6699200	-2.1802420	-0.9619120
C	4.9342140	-0.8800730	1.1677230
C	4.8473210	-1.4576670	-1.1578700
C	5.4899340	-0.7981590	-0.1115710
H	5.4279190	-0.3769950	1.9957590
H	5.2733290	-1.4122830	-2.1571400
C	3.0007430	-2.9030240	-2.0911900
H	3.0805310	-3.9889900	-1.9753770
H	1.9315850	-2.6761470	-2.1521280
H	3.4529750	-2.6446920	-3.0493650
C	3.1657490	-1.6587380	2.7884720
H	2.1556370	-1.2353550	2.8025280

H	3.0793270	-2.6913270	3.1414670
H	3.7727240	-1.1116120	3.5107480
C	6.7558000	-0.0320410	-0.3408640
H	6.9944080	0.0485590	-1.4020070
H	6.6896390	0.9793440	0.0684260
H	7.6041810	-0.5158680	0.1522310
C	-5.8440040	-2.5088330	0.6253380
H	-6.1350260	-1.6347790	1.2117610
H	-6.2171160	-2.3818890	-0.3933150
H	-6.3758180	-3.3660730	1.0496650
C	-1.6059330	-3.9820960	-1.6572180
H	-0.8672830	-3.2421100	-1.9878470
H	-1.0559330	-4.9114240	-1.4848160
H	-2.3003680	-4.1504040	-2.4805670
C	-1.5049820	-2.4025750	3.1518150
H	-0.4225410	-2.4359920	3.0309240
H	-1.7671750	-1.4243210	3.5633290
H	-1.7777850	-3.1456970	3.9079360
C	0.6737950	-2.3151810	0.4893470
Ir	0.2324240	-0.2960380	0.3293320
H	1.7186010	-0.0405300	0.6955680
H	0.0773730	-0.3008130	1.8791610
P	-0.2316990	1.9963360	0.5112890
C	1.1447580	3.0551570	1.0840270
C	2.0763970	2.5157410	1.9775960
C	1.2601140	4.3989760	0.7117920
C	3.1016220	3.3031790	2.4859250
H	1.9962310	1.4743530	2.2734440

C	2.2917790	5.1816500	1.2180970
H	0.5483540	4.8354030	0.0175620
C	3.2134890	4.6362010	2.1048510
H	3.8169390	2.8730440	3.1794410
H	2.3739760	6.2208230	0.9179220
H	4.0175960	5.2491810	2.4976870
C	-1.5745100	2.3353330	1.7163970
C	-2.3757620	1.2943820	2.1929500
C	-1.8430280	3.6419460	2.1364370
C	-3.4259090	1.5530820	3.0681320
H	-2.1634410	0.2732950	1.8870260
C	-2.8905580	3.8989250	3.0114340
H	-1.2269540	4.4632040	1.7816900
C	-3.6852910	2.8556270	3.4779930
H	-4.0369250	0.7349280	3.4373070
H	-3.0858640	4.9162280	3.3331500
H	-4.5004360	3.0589200	4.1641210
C	-0.8459350	2.7977170	-1.0110520
C	-2.2139440	2.9810020	-1.2332490
C	0.0605410	3.1357260	-2.0234920
C	-2.6647060	3.5125700	-2.4381690
H	-2.9302820	2.7249830	-0.4575880
C	-0.3927670	3.6824350	-3.2154840
H	1.1255580	2.9798450	-1.8711720
C	-1.7562710	3.8722740	-3.4256530
H	-3.7282450	3.6569880	-2.5972720
H	0.3199640	3.9533060	-3.9875600
H	-2.1078190	4.2978550	-4.3597190

H	2.6710560	-4.8921960	0.8835480
H	-0.0396720	-5.4640440	1.1200760
C	-1.0957700	-0.0473120	-2.9260480
C	0.2472110	-0.2511120	-2.5748960
C	1.2935590	0.0996030	-3.4185930
C	1.0083450	0.6503990	-4.6643940
C	-0.3145650	0.8370670	-5.0527220
C	-1.3513770	0.4968180	-4.1916820
H	0.5171980	-0.8296750	-1.6745480
H	2.3204220	-0.0699530	-3.1099150
H	1.8167810	0.9230070	-5.3350100
H	-0.5376820	1.2585490	-6.0275590
H	-2.3811430	0.6619590	-4.4941570
B	-2.2678620	-0.3749250	-1.9643310
O	-1.9714680	-0.5315820	-0.6147820
H	-2.6819970	-0.8821750	-0.0606160
O	-3.5283120	-0.5020040	-2.4511700
H	-4.2044280	-0.6854330	-1.7938400

PhBGly bound complex 4b

C	1.7832490	-4.2395830	0.7709200
C	0.4861500	-4.5686850	0.5875440
N	1.8634830	-2.8609760	0.6669640
N	-0.1979310	-3.3843070	0.3751710
C	-1.6052530	-3.3460840	0.0886730
C	-2.5239700	-3.1385480	1.1202970
C	-2.0042010	-3.5679130	-1.2371370
C	-3.8808290	-3.1377270	0.7842140

C	-3.3671270	-3.5630870	-1.5162660
C	-4.3231410	-3.3513080	-0.5178760
H	-4.6108040	-2.9671680	1.5722680
H	-3.6936220	-3.7357130	-2.5394940
C	3.1128350	-2.1562430	0.7607590
C	3.5530650	-1.7174600	2.0115110
C	3.8749560	-2.0088270	-0.4043480
C	4.8004140	-1.0934940	2.0697960
C	5.1170700	-1.3936790	-0.2889390
C	5.5960270	-0.9270940	0.9371390
H	5.1627090	-0.7399400	3.0316960
H	5.7196100	-1.2582850	-1.1838430
C	3.3521590	-2.4782600	-1.7269380
H	3.2347900	-3.5666710	-1.7580270
H	2.3646940	-2.0526330	-1.9385380
H	4.0203130	-2.1903010	-2.5390590
C	2.7360890	-1.9477730	3.2448220
H	1.6896970	-1.6671030	3.0969730
H	2.7420070	-3.0048270	3.5320030
H	3.1268580	-1.3782970	4.0884380
C	6.9191800	-0.2308530	1.0158190
H	7.7069090	-0.8095600	0.5273440
H	6.8826090	0.7383870	0.5066770
H	7.2234570	-0.0501440	2.0473900
C	-5.7842090	-3.3764910	-0.8468480
H	-6.3948060	-3.0389630	-0.0079180
H	-6.0146590	-2.7476810	-1.7111720
H	-6.1113600	-4.3889710	-1.1015310

C	-0.9875840	-3.7907500	-2.3163380
H	-0.2281030	-3.0013830	-2.3231360
H	-0.4501570	-4.7346660	-2.1808810
H	-1.4589360	-3.8234560	-3.3001280
C	-2.1069610	-2.9154550	2.5427380
H	-1.0257690	-2.8290940	2.6573440
H	-2.5582330	-1.9997560	2.9374480
H	-2.4489820	-3.7353850	3.1811920
C	0.6419530	-2.3038700	0.4164720
Ir	0.0375990	-0.3207460	0.3842210
H	0.5471810	-0.2373460	1.8523840
H	-1.2308890	-0.8593580	1.0867100
P	-0.9762280	1.7854910	0.6261990
C	-0.8186390	2.5858300	2.2654060
C	-0.8258620	1.7703180	3.4019860
C	-0.7995800	3.9749510	2.4268770
C	-0.8059950	2.3321150	4.6722320
H	-0.8516080	0.6904470	3.2878100
C	-0.7703330	4.5328950	3.7000900
H	-0.8017290	4.6270460	1.5589850
C	-0.7727650	3.7142320	4.8237750
H	-0.8135400	1.6887850	5.5457030
H	-0.7480740	5.6116420	3.8123240
H	-0.7507300	4.1526390	5.8156560
C	-2.8022240	1.7403350	0.4068600
C	-3.4887390	0.5413440	0.1973520
C	-3.5324060	2.9330870	0.4812520
C	-4.8746130	0.5354410	0.0622080

H	-2.9430230	-0.3960430	0.1351600
C	-4.9132180	2.9255770	0.3433240
H	-3.0159700	3.8745850	0.6477960
C	-5.5880380	1.7251600	0.1343130
H	-5.3921770	-0.4054700	-0.0959380
H	-5.4656640	3.8571300	0.4034210
H	-6.6679770	1.7208420	0.0317520
C	-0.4437130	3.0444820	-0.5849730
C	-1.1426220	3.2276790	-1.7823770
C	0.7580230	3.7367680	-0.3888920
C	-0.6583190	4.0948490	-2.7570920
H	-2.0844110	2.7107610	-1.9466580
C	1.2259300	4.6172550	-1.3539240
H	1.3252030	3.5888220	0.5262090
C	0.5205310	4.7968430	-2.5412890
H	-1.2110780	4.2271850	-3.6813430
H	2.1517310	5.1570200	-1.1838920
H	0.8924750	5.4815170	-3.2962130
H	2.6549200	-4.8423290	0.9647790
H	-0.0182490	-5.5205320	0.5900150
C	1.7795910	1.0845350	-2.3678730
C	2.3032120	0.8231150	-1.0951910
C	3.4391530	1.4630520	-0.6194970
C	4.1043570	2.3669040	-1.4430580
C	3.6300320	2.6186250	-2.7273650
C	2.4746460	1.9928550	-3.1792220
H	1.9083770	0.0050420	-0.4683780
H	3.8101520	1.2386710	0.3752470

H	4.9975330	2.8695210	-1.0850560
H	4.1552280	3.3162890	-3.3715210
H	2.0891680	2.2167430	-4.1694620
B	0.4373050	0.5003390	-2.8367870
O	-0.4030570	-0.2389950	-1.9975910
O	-0.0993050	0.6708540	-4.0735160
C	-1.3365630	-0.0494150	-4.1279550
C	-1.6796550	-0.3413010	-2.6721500
H	-2.0879020	0.5639790	-4.6287680
H	-2.3516840	0.4056230	-2.2370030
H	-2.1017290	-1.3307570	-2.4946940
H	-1.1843170	-0.9614620	-4.7151930

PhBCat bound complex 4c

C	4.4240970	-0.5942610	3.3069770
C	3.0543400	-0.7008450	3.5036410
C	2.1574940	-0.5985270	2.4265680
C	2.6960550	-0.4037590	1.1483230
C	4.0614500	-0.2710570	0.9439150
C	4.9280930	-0.3640600	2.0287590
H	5.1010890	-0.6723840	4.1509040
H	2.6626880	-0.8439790	4.5062970
H	2.0373760	-0.3384520	0.2756500
H	4.4476380	-0.0978960	-0.0556280
H	5.9977960	-0.2588420	1.8767840
B	0.6559790	-0.5470030	2.7035620
C	-1.5203340	-0.1455100	2.4406350
C	-2.7587480	0.2352800	1.9756860

C	-1.2534100	-0.3540640	3.7865190
C	-3.7605910	0.3686600	2.9408540
H	-2.9396030	0.4243200	0.9228440
C	-2.2376860	-0.2338160	4.7460430
C	-3.5073940	0.1316260	4.2916430
H	-4.7546070	0.6680780	2.6262060
H	-2.0250800	-0.3975660	5.7952670
H	-4.3103010	0.2463210	5.0109690
O	-0.3405770	-0.3166070	1.7318980
O	0.0811160	-0.6231320	3.9572620
C	-1.9494550	-3.9940690	-1.5062710
C	-0.6367350	-4.3171280	-1.5361780
N	-2.0271970	-2.6519860	-1.1704430
N	0.0577250	-3.1635860	-1.2169090
C	1.4868490	-3.0674300	-1.1260390
C	2.2166900	-2.5679640	-2.2103060
C	2.0995670	-3.5059520	0.0576290
C	3.6060950	-2.5050390	-2.0700970
C	3.4857250	-3.4455460	0.1310410
C	4.2570830	-2.9471430	-0.9213880
H	4.1940520	-2.1185600	-2.9000610
H	3.9764390	-3.7597540	1.0492330
C	-3.2715610	-1.9564730	-0.9890990
C	-3.7473620	-1.1342090	-2.0184260
C	-3.9713600	-2.1500410	0.2047680
C	-4.9474000	-0.4619740	-1.8002650
C	-5.1831050	-1.4769330	0.3583460
C	-5.6821710	-0.6224980	-0.6226810

H	-5.3279300	0.1930360	-2.5801740
H	-5.7373060	-1.6083210	1.2844780
C	-3.4307010	-3.0266820	1.2929680
H	-3.5775740	-4.0889960	1.0707210
H	-2.3551520	-2.8801890	1.4332690
H	-3.9244990	-2.8192990	2.2437210
C	-3.0097490	-1.0081720	-3.3156350
H	-1.9708090	-0.7015430	-3.1667060
H	-2.9818270	-1.9643780	-3.8489830
H	-3.4909890	-0.2804470	-3.9705060
C	-6.9872160	0.0869470	-0.4325660
H	-7.2384780	0.1963860	0.6241570
H	-6.9775950	1.0799510	-0.8863530
H	-7.8062680	-0.4684290	-0.9006390
C	5.7490870	-2.8872210	-0.8124870
H	6.1662350	-2.0748180	-1.4122620
H	6.0709350	-2.7505020	0.2221770
H	6.2068970	-3.8151730	-1.1697400
C	1.2789510	-3.9926690	1.2121720
H	0.4738360	-3.2899660	1.4550780
H	0.7953170	-4.9512120	0.9977400
H	1.8942440	-4.1237960	2.1028820
C	1.5771640	-2.1556500	-3.5024920
H	0.4885140	-2.2016070	-3.4714370
H	1.8580580	-1.1339890	-3.7754010
H	1.9158730	-2.8022600	-4.3174850
C	-0.7852540	-2.1184510	-0.9801330
Ir	-0.1834980	-0.1735710	-0.6498120

H	-1.6322930	0.2805840	-0.9371600
H	0.0046000	-0.0710000	-2.1979440
P	0.4715450	2.0825790	-0.5544200
C	-0.7585290	3.2495930	-1.2359560
C	-1.5378030	2.8406900	-2.3239470
C	-0.9168450	4.5444230	-0.7319250
C	-2.4563090	3.7111370	-2.8966980
H	-1.4185160	1.8373320	-2.7217380
C	-1.8412570	5.4105460	-1.3055600
H	-0.3253060	4.8741240	0.1172330
C	-2.6115400	4.9961690	-2.3866320
H	-3.0535360	3.3845940	-3.7419080
H	-1.9596270	6.4115990	-0.9048130
H	-3.3324130	5.6742370	-2.8307050
C	2.0120930	2.4352220	-1.4737570
C	2.7738430	1.3795290	-1.9810020
C	2.4579200	3.7478720	-1.6604890
C	3.9655390	1.6285360	-2.6541280
H	2.4303170	0.3541150	-1.8548280
C	3.6456920	3.9950530	-2.3359100
H	1.8720050	4.5795770	-1.2785520
C	4.4023430	2.9360800	-2.8316390
H	4.5487330	0.7994440	-3.0440750
H	3.9820810	5.0162940	-2.4786510
H	5.3286600	3.1332290	-3.3605260
C	0.8003770	2.6810120	1.1392580
C	2.0979340	2.8388010	1.6329680
C	-0.2843380	2.8190400	2.0152610

C	2.3051790	3.1193380	2.9802150
H	2.9505440	2.7295160	0.9693650
C	-0.0739900	3.0993240	3.3586250
H	-1.2994180	2.7029410	1.6432890
C	1.2239040	3.2436870	3.8449640
H	3.3177450	3.2331930	3.3533330
H	-0.9245540	3.2000630	4.0258000
H	1.3893790	3.4577170	4.8954360
H	-2.8333180	-4.5809660	-1.6929210
H	-0.1321310	-5.2426910	-1.7578390

Free Catalyst 5

C	-5.0353710	1.1664580	-1.3613820
C	-3.8359690	1.7536080	-0.9572880
C	-2.9539980	1.1131650	-0.0904350
C	-3.3020930	-0.1651760	0.3788580
C	-4.4888250	-0.7969310	-0.0131610
C	-5.3378270	-0.1030080	-0.8751360
H	-3.5834440	2.7483540	-1.3155130
H	-6.2622100	-0.5825040	-1.1857130
C	-2.6580600	-1.3844060	2.4954200
C	-1.5175580	-1.9794320	2.9221220
H	-3.6198810	-1.2822520	2.9679160
H	-1.2970500	-2.5083950	3.8343750
N	-2.3877660	-0.8618960	1.2404570
N	-0.5822230	-1.8083010	1.9167420
C	-1.1009880	-1.1173760	0.8736110
C	0.8083620	-2.2572390	1.9366260

H	0.9513600	-2.8435350	2.8460100
H	0.9662140	-2.9296380	1.0876480
C	1.7870680	-1.0900080	1.9070070
H	2.7979380	-1.4599780	2.0983280
H	1.5487510	-0.3887410	2.7103770
P	1.8326580	-0.1638770	0.2978910
C	3.3146380	-0.7790720	-0.5522030
C	4.5333880	-0.7538860	0.1405790
C	3.2776020	-1.2432470	-1.8676880
C	5.6935070	-1.1996510	-0.4757390
H	4.5839250	-0.3657870	1.1550220
C	4.4445940	-1.6924870	-2.4774100
H	2.3401570	-1.2356270	-2.4120590
C	5.6485530	-1.6737920	-1.7844590
H	6.6338210	-1.1735390	0.0637200
H	4.4099240	-2.0534930	-3.4993190
H	6.5564660	-2.0230770	-2.2639830
C	2.1924960	1.5547620	0.7524210
C	3.0160440	2.3282650	-0.0742300
C	1.5607260	2.1541500	1.8490650
C	3.2234380	3.6709910	0.2114390
H	3.4957200	1.8764430	-0.9371990
C	1.7713280	3.4978030	2.1288460
H	0.8987590	1.5775010	2.4897530
C	2.6051080	4.2558950	1.3122590
H	3.8701620	4.2621240	-0.4273340
H	1.2887270	3.9524680	2.9870590
H	2.7719860	5.3042500	1.5341560

H	-5.5685870	-2.6297610	-0.2427500
H	-5.3174580	-2.1765830	1.4324220
H	-3.9799910	-2.8356220	0.4995180
C	-4.8506960	-2.1782510	0.4422640
H	-6.9470720	1.4249340	-2.3186860
H	-5.5661170	1.8427980	-3.3280710
H	-6.0631870	2.9302370	-2.0428160
C	-5.9543650	1.8757490	-2.3053990
H	-1.3602480	1.5361430	1.3273210
H	-1.8313800	2.9005780	0.3118960
H	-0.8394000	1.6874940	-0.3832100
C	-1.6932360	1.8165550	0.3281030
Ir	-0.0724880	-0.3084640	-0.7693070
H	0.4319030	-1.6683950	-1.3268270
H	0.6846310	0.3742240	-2.0586960

Phenylboronic acid pinacol ester 12

C	3.7752060	1.1921630	0.1786140
C	2.3860810	1.1893430	0.1769780
C	1.6654200	0.0000900	0.0000260
C	2.3859380	-1.1892540	-0.1769500
C	3.7750610	-1.1922300	-0.1786350
C	4.4712170	-0.0000730	-0.0000240
H	4.3186770	2.1214780	0.3180790
H	1.8416550	2.1195160	0.3142750
H	1.8413910	-2.1193580	-0.3142200
H	4.3184230	-2.1216070	-0.3181150
H	5.5569540	-0.0001360	-0.0000450

B	0.1214980	0.0001250	0.0000320
O	-0.6349490	-1.1257010	-0.2097540
O	-0.6350560	1.1258500	0.2097860
C	-2.0093620	-0.7742970	0.1020310
C	-2.0094880	0.7742490	-0.1020420
C	-2.9297250	-1.5389520	-0.8259730
H	-2.8618840	-2.6092870	-0.6201980
H	-3.9701630	-1.2341600	-0.6777830
H	-2.6692630	-1.3816650	-1.8728500
C	-2.2483570	-1.1798800	1.5491600
H	-3.2852770	-1.0097750	1.8505780
H	-2.0288180	-2.2429130	1.6663630
H	-1.5973310	-0.6246480	2.2288460
C	-2.2484260	1.1798320	-1.5491680
H	-3.2853290	1.0096960	-1.8506410
H	-2.0289160	2.2428720	-1.6663300
H	-1.5973560	0.6246350	-2.2288420
C	-2.9299330	1.5388230	0.8259370
H	-2.8621680	2.6091710	0.6202090
H	-3.9703490	1.2339620	0.6777130
H	-2.6694970	1.3815120	1.8728160

Phenylboronic acid bound complex 6a

C	-4.9329070	0.2726850	0.1892340
C	-4.1242520	0.6910820	1.2427200
C	-3.0189030	-0.0485030	1.6627640
C	-2.7330730	-1.2350830	0.9819740
C	-3.5444260	-1.7185340	-0.0508200

C	-4.6368550	-0.9447440	-0.4304010
H	-4.3364640	1.6371590	1.7343730
H	-5.2698410	-1.2931040	-1.2436680
C	-1.5271790	-2.9094850	2.3788290
C	-0.2704020	-3.4107270	2.4077370
H	-2.3946620	-3.1242250	2.9805780
H	0.1806670	-4.1559610	3.0416970
N	-1.5617520	-1.9857890	1.3482600
N	0.4246700	-2.7856390	1.3900950
C	-0.3521530	-1.8910670	0.7167500
C	1.8334890	-3.0159660	1.0815030
H	2.1655180	-3.8597790	1.6896540
H	1.9106380	-3.3181370	0.0335000
C	2.6932840	-1.7928710	1.3606520
H	3.7447130	-2.0510750	1.2122880
H	2.5859630	-1.4888280	2.4049200
P	2.3076440	-0.3474840	0.2511200
C	3.8449690	-0.1334570	-0.6995430
C	5.0384940	0.0999720	-0.0022340
C	3.8690010	-0.2315100	-2.0905620
C	6.2347970	0.2351760	-0.6917370
H	5.0308760	0.1914130	1.0815560
C	5.0729760	-0.0997900	-2.7763870
H	2.9427310	-0.3995560	-2.6286740
C	6.2525830	0.1334230	-2.0808930
H	7.1534600	0.4228770	-0.1466940
H	5.0853900	-0.1761610	-3.8581930
H	7.1885210	0.2394280	-2.6187410

C	2.2442610	1.0843850	1.3776130
C	2.7321380	2.3277090	0.9623600
C	1.5980770	0.9816910	2.6146890
C	2.6017540	3.4395210	1.7860290
H	3.2193400	2.4228130	-0.0034960
C	1.4685050	2.0963330	3.4354280
H	1.1904640	0.0284390	2.9445570
C	1.9759900	3.3253740	3.0239860
H	2.9933010	4.3972340	1.4606940
H	0.9748170	2.0032760	4.3972750
H	1.8833410	4.1931360	3.6681350
H	-3.8126890	-3.1234570	-1.6551500
H	-3.5171560	-3.8725240	-0.0950520
H	-2.1799730	-3.1362930	-0.9502590
C	-3.2452070	-3.0212260	-0.7276050
H	-7.0167320	0.5646200	-0.2679780
H	-5.9111670	1.4097520	-1.3400990
H	-6.1880890	2.0200130	0.2937480
C	-6.0708860	1.1120970	-0.2988750
H	-2.2099170	-0.2124920	3.6567040
H	-2.4129750	1.4447340	3.0814950
H	-1.0876200	0.4469120	2.4772650
C	-2.1410940	0.4337100	2.7753230
Ir	0.4009950	-0.5964140	-0.8397790
H	1.1088400	-1.7867020	-1.5625050
H	0.9896410	0.3521010	-2.0258660
C	-2.3090660	4.1042810	-0.9980850
C	-2.6006260	2.9603900	-1.7319160

C	-2.0365680	1.7210440	-1.4011980
C	-1.1795850	1.6816740	-0.2922480
C	-0.8729830	2.8158600	0.4461660
C	-1.4397580	4.0350430	0.0865260
H	-2.7546760	5.0537250	-1.2749350
H	-3.2708340	3.0209950	-2.5841040
H	-0.8648590	0.7157980	0.1464240
H	-0.2167390	2.7450420	1.3076300
H	-1.2084350	4.9284090	0.6576720
B	-2.3423360	0.4773640	-2.2676480
O	-1.5285100	-0.6436770	-2.0792600
O	-3.3340470	0.5186320	-3.1891220
H	-3.4469630	-0.2562130	-3.7428540
H	-1.7143540	-1.4246050	-2.6056060

PhenylBGly bound complex 6b

C	-4.8180980	0.5981310	0.2945910
C	-3.9810890	1.4957220	0.9522350
C	-2.8449410	1.0739320	1.6423580
C	-2.5645700	-0.2949620	1.6571290
C	-3.4116310	-1.2395370	1.0658090
C	-4.5261310	-0.7664270	0.3791470
H	-4.1935950	2.5604120	0.8981980
H	-5.1875550	-1.4816390	-0.1052850
C	-1.2886030	-0.9701140	3.6847590
C	-0.0441550	-1.4430300	3.9274090
H	-2.1183900	-0.7751650	4.3437050
H	0.4330680	-1.7445420	4.8450690

N	-1.3748470	-0.7610350	2.3189180
N	0.5923570	-1.5162850	2.7032280
C	-0.2098460	-1.0947790	1.6847070
C	1.9692580	-1.9617850	2.5081730
H	2.3179780	-2.3675090	3.4600170
H	1.9641600	-2.7822080	1.7856820
C	2.8824840	-0.8371500	2.0464560
H	3.9081350	-1.2095380	1.9874450
H	2.8816490	-0.0245310	2.7775860
P	2.4325340	-0.1618550	0.3689090
C	3.9276580	-0.4785590	-0.6186730
C	5.1454250	0.0650400	-0.1864090
C	3.8973820	-1.2746840	-1.7633290
C	6.3115390	-0.1836760	-0.8955320
H	5.1799860	0.6964810	0.6984980
C	5.0715110	-1.5250000	-2.4676180
H	2.9521710	-1.6850820	-2.1011770
C	6.2750720	-0.9812360	-2.0370290
H	7.2491540	0.2460250	-0.5601530
H	5.0420370	-2.1443280	-3.3574980
H	7.1876190	-1.1750980	-2.5903530
C	2.3980290	1.6465770	0.6087780
C	2.8735120	2.5042000	-0.3883770
C	1.7944360	2.1909780	1.7480970
C	2.7777030	3.8817440	-0.2284960
H	3.3280180	2.0931230	-1.2848500
C	1.6991790	3.5691830	1.9037260
H	1.3966860	1.5387420	2.5231270

C	2.1984160	4.4161580	0.9184890
H	3.1608360	4.5392580	-1.0013100
H	1.2389820	3.9804130	2.7963520
H	2.1343690	5.4916230	1.0442710
H	-3.7864600	-3.2895250	0.5392260
H	-3.2859980	-3.0560780	2.2091420
H	-2.0905010	-2.9399360	0.9317190
C	-3.1281990	-2.7047910	1.1840400
H	-6.9380880	0.7606360	-0.0394400
H	-5.9815830	0.6473320	-1.5079480
H	-6.0113400	2.1577690	-0.5898460
C	-5.9951350	1.0708730	-0.4992390
H	-1.9839700	1.9764860	3.4035340
H	-2.1987800	3.0813450	2.0440160
H	-0.8918680	1.8936740	2.0301010
C	-1.9365240	2.0569870	2.3123030
Ir	0.4714440	-0.8946580	-0.3564240
H	1.1006550	-2.3200340	-0.3582780
H	1.0014400	-0.7690260	-1.8935180
C	-2.5426050	3.2154790	-2.6089680
C	-2.7820840	1.8478020	-2.6558440
C	-2.0697680	0.9586580	-1.8387080
C	-1.1136440	1.5042010	-0.9721100
C	-0.8526190	2.8656300	-0.9237000
C	-1.5734420	3.7244500	-1.7490820
H	-3.1037910	3.8866180	-3.2502260
H	-3.5264790	1.4522190	-3.3405730
H	-0.6298640	0.8898380	-0.1925840

H	-0.1079930	3.2542950	-0.2363050
H	-1.3801400	4.7918180	-1.7186010
B	-2.2992410	-0.5533510	-1.9658500
O	-1.4982120	-1.4982210	-1.3077670
O	-3.2022910	-1.1521240	-2.7842320
C	-1.7242500	-2.7708860	-1.9583520
C	-3.0801480	-2.5718170	-2.6242960
H	-0.9122730	-2.9306840	-2.6746050
H	-1.7044940	-3.5637560	-1.2096800
H	-3.1528730	-3.0519090	-3.6007300
H	-3.9071810	-2.9241780	-1.9981880

PhenylBCat bound complex 6c

C	-4.3289760	1.8572790	0.4682280
C	-3.2188500	2.6089620	0.8462560
C	-2.1603600	2.0501130	1.5620930
C	-2.2430210	0.6943540	1.8897710
C	-3.3724660	-0.0824050	1.5989570
C	-4.3958810	0.5204920	0.8723930
H	-3.1558340	3.6533350	0.5499210
H	-5.2687190	-0.0730510	0.6092910
C	-1.0154630	-0.0035750	3.9421420
C	0.1391610	-0.6626980	4.1944110
H	-1.7610410	0.4072780	4.6024470
H	0.6052310	-0.9460480	5.1234960
N	-1.1343770	0.0751410	2.5661460
N	0.6894350	-0.9736730	2.9657660
C	-0.0803850	-0.5237180	1.9338710

C	1.9597580	-1.6705800	2.7822700
H	2.2946240	-1.9979770	3.7684840
H	1.7791540	-2.5690360	2.1852040
C	3.0135340	-0.7860420	2.1337540
H	3.9759410	-1.3036400	2.1417880
H	3.1405730	0.1304310	2.7151250
P	2.6183950	-0.3546400	0.3681350
C	3.9012740	-1.2386720	-0.5724180
C	5.2473330	-0.9622860	-0.2950610
C	3.5794230	-2.1999400	-1.5305770
C	6.2523760	-1.6383020	-0.9714030
H	5.5111050	-0.2053560	0.4401790
C	4.5922560	-2.8790510	-2.2015760
H	2.5377980	-2.4023410	-1.7539640
C	5.9244910	-2.5997650	-1.9246430
H	7.2917700	-1.4142530	-0.7576630
H	4.3360730	-3.6251450	-2.9458600
H	6.7113100	-3.1279060	-2.4523480
C	3.0476960	1.4111670	0.2206220
C	3.6279440	1.8984670	-0.9553240
C	2.6980180	2.3107680	1.2341760
C	3.8831410	3.2573590	-1.0968100
H	3.8874510	1.2122070	-1.7559140
C	2.9525940	3.6695490	1.0878750
H	2.2266590	1.9544920	2.1472720
C	3.5525290	4.1430770	-0.0753910
H	4.3452700	3.6243770	-2.0067880
H	2.6878140	4.3564800	1.8849700

H	3.7614330	5.2017530	-0.1855290
H	-4.2444370	-2.0464180	1.5193910
H	-3.8045230	-1.5100090	3.1391620
H	-2.5519100	-2.0388510	2.0272790
C	-3.4958070	-1.4926130	2.0879560
H	-5.4986050	1.9330290	-1.3276890
H	-5.2423830	3.5084000	-0.5715580
H	-6.3936510	2.3612140	0.1204070
C	-5.4192250	2.4517150	-0.3669230
H	-0.0319880	2.3812680	1.7128710
H	-0.9603320	3.0502670	3.0445920
H	-0.9870120	3.8473150	1.4697780
C	-0.9760370	2.8744910	1.9634800
Ir	0.4968430	-0.7088260	-0.1406960
H	0.8160980	-2.2217040	0.0538980
H	0.9427930	-0.8995340	-1.6972420
C	-1.5456380	3.4919680	-3.1649360
C	-2.1556560	2.2709500	-2.9061120
C	-1.6561760	1.4102440	-1.9187070
C	-0.5307010	1.8328010	-1.1964140
C	0.0921930	3.0448210	-1.4493640
C	-0.4192810	3.8762070	-2.4431620
H	-1.9437230	4.1440740	-3.9346960
H	-3.0272690	1.9703400	-3.4800450
H	-0.1979370	1.2837990	-0.2977360
H	0.9541790	3.3484740	-0.8642860
H	0.0588130	4.8283840	-2.6485270
B	-2.2937990	0.0471630	-1.6677530

C	-2.4664220	-2.0735860	-0.9905530
C	-2.2695140	-3.3303760	-0.4656960
C	-3.5934580	-1.7277440	-1.7228530
C	-3.2954880	-4.2518910	-0.6851040
H	-1.3715040	-3.5786550	0.0886430
C	-4.6148220	-2.6301510	-1.9402540
C	-4.4426870	-3.9062850	-1.3998300
H	-3.1947650	-5.2576910	-0.2941900
H	-5.4938910	-2.3595510	-2.5120690
H	-5.2167110	-4.6497840	-1.5510410
O	-1.6431330	-0.9611950	-0.9244130
O	-3.4908140	-0.4272440	-2.1569620

PhenylBPin bound complex 6d

C	-4.6542660	-1.9608530	0.6826600
C	-3.4799560	-2.6569470	0.9746630
C	-2.3928270	-2.6606460	0.1050390
C	-2.4967490	-1.9068950	-1.0681800
C	-3.6735830	-1.2494590	-1.4331570
C	-4.7381840	-1.2846230	-0.5342500
H	-3.4095570	-3.2202780	1.9018040
H	-5.6605480	-0.7711800	-0.7951950
C	-1.2708150	-2.7198300	-3.0596240
C	-0.1278650	-2.3857650	-3.6999830
H	-2.0148210	-3.4699640	-3.2707320
H	0.3301670	-2.7849190	-4.5897120
N	-1.3812960	-1.8680650	-1.9744870

N	0.4230870	-1.3348290	-2.9932490
C	-0.3312340	-0.9926100	-1.9061300
C	1.7057290	-0.7216080	-3.3224720
H	2.0027580	-1.0985650	-4.3032320
H	1.5618340	0.3576950	-3.4183320
C	2.7701740	-1.0438450	-2.2858740
H	3.7386670	-0.6706570	-2.6273090
H	2.8674650	-2.1265340	-2.1742050
P	2.4189690	-0.2701400	-0.6312000
C	3.7831370	0.9269270	-0.4578430
C	5.0973320	0.4391500	-0.4489280
C	3.5622420	2.3025220	-0.3911780
C	6.1689450	1.3165710	-0.3679540
H	5.2830300	-0.6316470	-0.4902670
C	4.6411490	3.1787130	-0.3161440
H	2.5456530	2.6802790	-0.3871120
C	5.9410370	2.6889680	-0.3029070
H	7.1823350	0.9304670	-0.3527140
H	4.4621870	4.2471750	-0.2633580
H	6.7793190	3.3743320	-0.2394140
C	2.7896420	-1.5764950	0.5829250
C	3.3509240	-1.2398250	1.8192910
C	2.4046540	-2.9002060	0.3447360
C	3.5480010	-2.2174460	2.7862610
H	3.6344920	-0.2113480	2.0229200
C	2.5996130	-3.8751070	1.3165470
H	1.9430730	-3.1786580	-0.5999170
C	3.1768510	-3.5353740	2.5364320

H	3.9921400	-1.9482730	3.7386280
H	2.3052380	-4.9005270	1.1180550
H	3.3378130	-4.2977860	3.2911050
H	-4.7169170	0.0037750	-2.8370110
H	-3.8091660	-1.3050990	-3.5808780
H	-2.9515720	0.0930520	-2.9661360
C	-3.7949010	-0.5746570	-2.7652450
H	-6.7297920	-1.6549600	1.1762400
H	-5.6048870	-1.2284580	2.4622760
H	-5.9367210	-2.9185170	2.1243810
C	-5.7927410	-1.9433540	1.6545370
H	-1.0738070	-4.3068850	-0.3156740
H	-1.1938270	-3.8917420	1.3955990
H	-0.2522910	-2.8817660	0.2909100
C	-1.1675650	-3.4727550	0.3887990
Ir	0.3455420	0.4811470	-0.4625690
H	0.7490460	1.4447890	-1.6187130
H	0.9194070	1.5961510	0.5787900
C	-0.3638870	0.0713930	4.7072370
C	-0.9861550	0.9656470	3.8436560
C	-1.1020970	0.6821550	2.4780480
C	-0.5727730	-0.5305630	2.0123630
C	0.0757950	-1.4181350	2.8613210
C	0.1750420	-1.1144350	4.2159090
H	-0.2846940	0.3040830	5.7639160
H	-1.3749370	1.9053220	4.2238320
H	-0.7509940	-0.8362470	0.9685740
H	0.4997110	-2.3399820	2.4758030

H	0.6743190	-1.8053380	4.8880360
B	-1.6484810	1.7235880	1.4842970
O	-1.7227150	1.4723270	0.1048940
O	-2.0352110	2.9795950	1.8081450
C	-2.4256270	2.6458340	-0.4831680
C	-2.1316750	3.7524790	0.5727320
C	-1.8765630	2.9171700	-1.8615560
H	-2.0782170	2.0740330	-2.5261090
H	-2.3602590	3.8005370	-2.2879580
H	-0.8003400	3.0894780	-1.8449060
C	-3.8943710	2.2703350	-0.5306380
H	-4.4859450	3.0753580	-0.9720210
H	-4.0283030	1.3770510	-1.1366090
H	-4.2867500	2.0639640	0.4681180
C	-0.7855510	4.4293300	0.3712470
H	-0.8036210	5.0957620	-0.4945480
H	-0.5486240	5.0271300	1.2529330
H	0.0125180	3.6947220	0.2320870
C	-3.2340120	4.7763560	0.7340240
H	-2.9460380	5.5125450	1.4862490
H	-3.4064570	5.3090630	-0.2053660
H	-4.1713170	4.3205950	1.0521390
